# 2025 ACVIM Forum Research Abstract Program

**DOI:** 10.1111/jvim.70258

**Published:** 2025-10-17

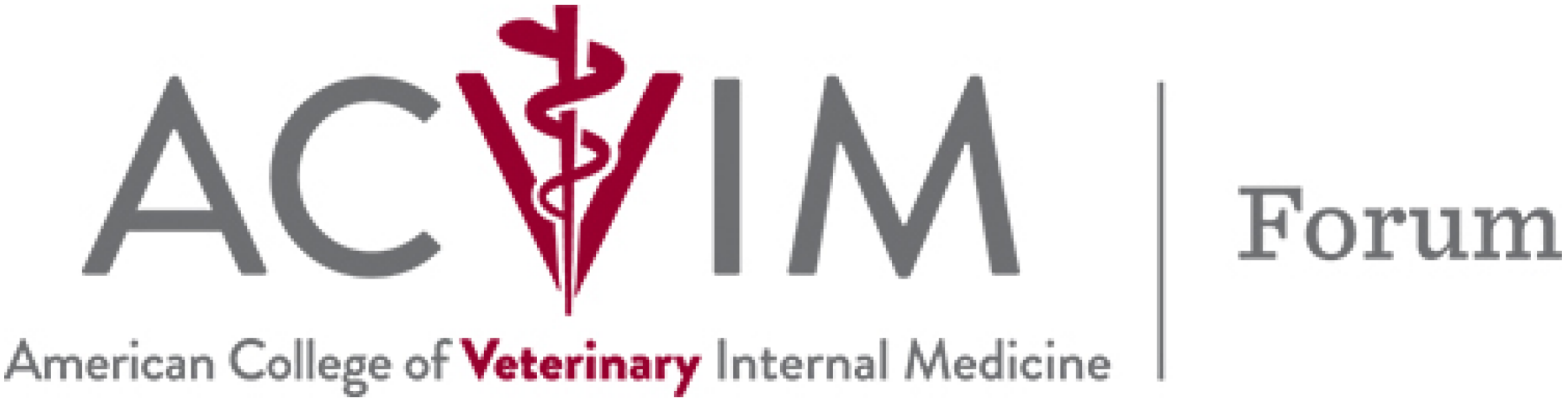

The American College of Veterinary Internal Medicine (ACVIM) Forum and the Journal of Veterinary Internal Medicine (JVIM) are not responsible for the content or dosage recommendations in the abstracts. The abstracts are not peer reviewed before publication. The opinions expressed in the abstracts are those of the author(s) and may not represent the views or position of the ACVIM. The authors are solely responsible for the content of the abstracts.

**2025 ACVIM Forum**

**June 18–October 31, 2025**

**Research Abstract Oral Program**

**Index of Abstracts**

**Table:** 

Title	Sequence Number	Award Eligible	Award Winner
**CARDIOLOGY**			
Hemodynamic Effects of Non‐ionic Contrast Prior to Patent Ductus Arteriosus Occlusion and Pulmonic Balloon Valvuloplasty	C01	Y	
Coadministration of Omecamtiv Mecarbil and Pimobendan: Observation of Safety and Efficacy (The COMPOSE Study)	C02	Y	2025 ACVIM Cardiology Pacemaker Resident Research Abstract Award
Comparing Two‐Dimensional Crescentic Ellipsoid Model Variants in Estimating Three‐Dimensional Echocardiographic Right Ventricular Volume in Dogs	C03	Y	
Segmental Left Ventricular Systolic Excursion in Cats with Hypertrophic Cardiomyopathy	C04	Y	
The Use of Contrast‐Enhanced Echocardiography in the Evaluation of Feline Left Ventricular Wall Thickness	C05	Y	
Quality of Life Assessment in Dogs Undergoing Mitral Transcatheter Edge‐to‐Edge Repair with the V‐Clamp Device	C06	Y	
Evaluation of Mitral Valve Leaflet Morphology in Healthy Beagle Dogs Using Three‐dimensional Transesophageal Echocardiography	C07	Y	2025 ACVIM Cardiology Pacemaker Resident Research Abstract Award
Echocardiographic Evaluation of Aortic Root Size in Cats with Hypertrophic Cardiomyopathy	C08	Y	2025 ACVIM Resident Research Abstract Award
Demographic, Clinical and Echocardiographic Characteristics of Dilated Cardiomyopathy in Dogs Eating Hydrolyzed Diets	C09	Y	
Bionote Point‐Of‐Care Quantitative NT‐Probnp Assay for Differentiating Stages of Preclinical Disease in Dogs and Cats	C10	Y	
Canine Hindlimb Function and Perfusion Following Femoral Arterial Ligation or Repair for Transcatheter Vascular Occlusion	C11	Y	
Accuracy of Echocardiographic Estimates of Mitral and Aortic Annular Area in Dogs with Mitral Regurgitation	C12	Y	
Effects of Zenalpha® (Medetomidine/Vatinoxan) on Blood Pressure, Echocardiographic Measurements, and Cardiac Rhythm in Healthy Dogs	C13	Y	2025 ACVIM Resident Research Abstract Award
Formalin and Aminocaproic Acid, but Not Yunnan Baiyao, Demonstrate Viscoelastic Procoagulant Effects in Healthy Horses	C14	Y	2025 ACVIM Resident Research Abstract Award
Detection of Congestive Heart Failure by Left Atrial Booster Strain in Cats with Cardiomyopathy	C15		
Development of a Second‐generation Vascular Occluder for Patent Ductus Arteriosus Occlusion in Dogs	C16		
Renin‐angiotensin‐aldosterone System Activation in Dogs with Moderate to Severe Hypochloremia and Congestive Heart Failure	C17		
Canine Cardiomyopathy and Taurine Deficiency in Multiple Breeds	C18		
**EQUINE**			
Insulin/Glucose Response to Varying Feeding Methods (Mare's Milk, Milk Replacer, Direct Nursing) in Foals	E01	Y	
Incretin Response to Varying Feeding Methods (Mare's Milk, Milk Replacer, Direct Nursing) in Foals	E02	Y	
The Hypothalamic‐pituitary‐adrenal Gland Axis Response to a Vasopressin Stimulation Test in Critically Ill Foals	E03	Y	
Effect of Ertugliflozin on Glucose and Insulin Dynamics in Healthy Horses Receiving Dexamethasone	E04	Y	2025 ACVIM Resident Research Abstract Award
Prolonged Wireless Measurement of Intragastric pH in Foals	E05	Y	2025 ACVIM Resident Research Abstract Award
Investigation of Potential Causes of Equine Fecal Water Syndrome	E06	Y	
Duodenal Macrophages and Their Role in Equine Inflammatory Bowel Disease (IBD): A Preliminary Study	E07	Y	
Investigation of Cardiac Troponin I, Arrhythmia Burden and Echocardiographic Variables in Apparently Healthy Neonatal Foals	E09	Y	
Effects of Hospitalization on Recumbency, Cortisol, and Heart Rate Variability in Horses	E10	Y	
Pulmonary Disposition and Pharmacokinetics of Ceftiofur Sodium After Nebulization in Adult Horses	E11	Y	
Antimicrobial Prescription Practices to Equine Patients at the Western College of Veterinary Medicine (2019–2023)	E12	Y	
The Neutrophil‐to‐Lymphocyte Ratio and Its Association with Outcome in Equine Colitis	E13	Y	
The Predictive Ability of Blood‐based Biomarkers to Detect bacteremia in Hospitalized Neonatal Foals	E14	Y	
Evaluation of a Lateral Flow Assay for Failure of Passive Transfer Diagnosis in Crias/Foals	E15	Y	
Hemoperfusion with a Novel Cytokine Adsorption Device Reduces Markers of Systemic Inflammation in LPS‐treated Horses	E16		
Formalin and Aminocaproic Acid, but Not Yunnan Baiyao, Demonstrate Viscoelastic Procoagulant Effects in Healthy Horses	E17	Y	
Evaluation of Two POC Measurement Systems for ACT and aPTT in Equine Blood	E18		
Blood Leukocyte Telomere Length Dynamics in the First Year of Life in Thoroughbreds	E19		
Prospective Categorisation of Early Maternal Care Style in Thoroughbred Broodmares	E20		
Computed Tomographic Detection and Management of Pneumolabyrinth in Three Horses with Temporohyoid Osteoarthropathy	E21		
Diagnostic Modalities for the Identification of Metastatic Neoplasia: A Clinical Series of 40 Cases	E22		
Progesterone Administration Decreases Airway Obstruction in Severe Equine Asthma	E23		
Poor Agreement Between Resting Tracheal Mucus Scores and Bronchoalveolar Lavage Cytology in Racing Thoroughbreds	E24		
**FOOD ANIMAL INTERNAL MEDICINE**			
Dual‐Center Retrospective Review of 204 Transfusions in Camelids	F01	Y	2025 ACVIM Resident Research Abstract Award
Pharmacokinetics of Lidocaine and its Metabolites Following Constant Rate Infusion in Healthy Goats	F02	Y	
Impact of Nebulizer Device on Aerosol Drug Delivery in Ruminants	F03	Y	
Pharmacokinetics of Famotidine in Goats: Subcutaneous and Intravenous Administration	F04		
The Effect of Antigen Characteristics on Camelid Immunoglobulin Isotype Responses	F05	Y	
Bacteremia Is Uncommon in Systemically Ill Goats Presented to a Tertiary Referral Hospital	F06	Y	
Cerebrospinal Nematodiasis in 8 Goats (2011‐2024)	F07	Y	
A Retrospective Look at the Efficacy of Proctoclysis in Hospitalized Swine	F08		
**NEUROLOGY**			
Case‐Control Investigation of Clinician‐Determined Variables in Progressive Myelomalacia After Disc Herniation in Dogs	N01	Y	
Mechanical Sensory Threshold in Neurologically Normal French Bulldogs with Congenital Vertebral Malformations	N02	Y	
Clinical and Imaging Findings in Fourteen Dogs with Surgically Treated Tethered Cord Syndrome	N03	Y	
Caretaker‐Reported Quality of Life, Functionality, and Complications Associated with Assistive Mobility Cart Use in Animals	N04	Y	
Breed Predispositions for Canine Otitis Media with Intracranial Extension	N05	Y	
Dissolution Characteristics of Split and Crushed Levetiracetam Extended‐Release Tablets in Comparison with Immediate‐Release Formulation	N06	Y	2025 ACVIM Resident Research Abstract Award
Treatment and Prognosis for Canine Calvarial Multilobular Tumor of Bone: 19 Cases (2012‐2024)	N07	Y	
Spinal‐only Meningomyelitis of Unknown Etiology: Clinical Presentation, Diagnosis, Treatment, and Outcome in 23 Dogs	N08	Y	2025 ACVIM Resident Research Abstract Award
Lipemia Interference in Automated Immunoassay Measurement of Plasma Phenobarbital Concentration	N09	Y	
Owner Observed Sleep Disturbances Between Cavalier King Charles Spaniels With and Without Symptomatic Chiari‐like Malformation	N10		
Platelet‐to‐Lymphocyte Ratio as a Potential Biomarker in Dogs with Idiopathic Epilepsy	N11		
The Relationship Between Cognition and Urinalysis Abnormalities in Aging Dogs	N12		
**NUTRITION**			
Increasing Dietary Fiber Intake Improves Fecal and Clinical Activity Scores in Dogs with Chronic Enteropathy	NM01	Y	2025 ACVIM Resident Research Abstract Award
Exploring Veterinary Professionals’ Strategies for Communicating and Documenting Canine Obesity	NM02		
Validation of a Food Frequency Questionnaire (FFQ) for Use in Dog Nutrition Research	NM03		
Home‐Prepared Diets for Companion Dogs Are Made from Diverse Ingredients and Few Are Balanced	NM04		
**SMALL ANIMAL INTERNAL MEDICINE — NEPHROLOGY/UROLOGY**			
Evaluation of Three Urinary Biomarkers for the Detection of Meloxicam‐induced Kidney Injury in Cats	NU01	Y	
Urinary Calcium Excretion and Bone Turnover in Dogs with Calcium Oxalate Urolithiasis: Preliminary Results	NU02	Y	
Effect of Sedation on Kidney Function Markers in Cats with Chronic Kidney Disease	NU03	Y	
Serum Triglyceride and Total Cholesterol in Dogs with Calcium Oxalate Urolithiasis	NU04	Y	
Investigating the Microbiome of the Canine Struvite Urolith and Its Link to the Oral Cavity	NU05		
Incidence of Acute Kidney Injury in Dogs Treated with Extracorporeal Shockwave Lithotripsy	NU06		
Early Characterisation of Autophagy and Antioxidant Response in Progressive Feline Chronic Kidney Disease	NU07		
Age, Signalment, and Renal Markers Affect Risk of Increased Cystatin B in Dogs and Cats	NU08		
Urinary Ammonia Excretion is Impaired in Dogs with Kidney Disease	NU09		
The Circulating and Intrarenal Renin‐angiotensin(‐aldosterone) Systems in Cats with Renal Ischemia‐induced Chronic Kidney Disease	NU10		
Circulating Renin‐angiotensin‐aldosterone System Markers in Cats with Non‐hypertensive Chronic Kidney Disease or Systemic Arterial Hypertension	NU11		
Cats with a Subcutaneous Ureteral Bypass Device locked with Saline or t‐EDTA	NU12		
Subcutaneous Ureteral Bypass Explantation in 21 Cats (2015‐2023): Outcomes and Owner Perceptions	NU13		
**ONCOLOGY**			
Minimally Invasive Sampling for Transcriptomic Profiling of the Immune Tumor Microenvironment in Canine B‐cell Lymphoma	O01	Y	
Optimization of Low‐Volume Blood Biopsy in Feline Cancer	O02	Y	
Comparison of Pooled vs. Single Samples Measuring Urine Protein: Creatinine Ratio in Dogs Receiving Toceranib Phosphate	O03	Y	2025 ACVIM Resident Research Abstract Award
MicroRNAs as Prognostic Markers of Canine Splenic Hemangiosarcoma	O04		
Validating Antibodies for Cross‐species Applications in Murine, Human, and Canine Osteosarcoma	O05		
Adoptive Cell Therapy with Chemotherapy Improves Canine Osteosarcoma Outcomes Compared to Standard of Care Chemotherapy	O06		
Plasma Extracellular Vesicles in Non‐Cancerous Bone Disease: Implications for Osteosarcoma Liquid Biopsy	O07		
Relationship Between Lactate Dehydrogenase and Substage in Dogs Diagnosed with Multicentric Large B‐cell Lymphoma	O08		
Organoid‐based Platform for Predicting Anticancer Drug Susceptibility in Animals	O09		
**SMALL ANIMAL INTERNAL MEDICINE — CARDIOLOGY**			
ENaC Blockade Enhances Circadian Variation in Systolic Blood Pressure Independent of Diabetic Status	SC01		
**SMALL ANIMAL INTERNAL MEDICINE — ENDOCRINOLOGY**			
Determining the Prevalence of Hypersomatotropism in a North American Cohort of Diabetic Cats	EN01	Y	
Dehydroepiandrosterone Sulfate (DHEAS) as a Potential Biomarker of the Etiology of Canine Cushing's Syndrome	EN02		
Evaluation of Hypoglycemic and Hyperglycemic Events in Non‐diabetic Dogs Using a Flash Glucose Monitoring System	EN03		
Autoimmune Polyglandular Syndrome Type II in Dogs: A Review of 51 Cases	EN04		
Pharmacodynamics of Insulin Lispro Assessed by Use of the Isoglycemic Clamp Method in Healthy Dogs	EN05		
Prior Obesity as a Risk Factor of Canine Diabetes Mellitus: a Pair‐matched 1:3 Case‐control Study	EN06		
**SMALL ANIMAL INTERNAL MEDICINE — GASTROENTEROLOGY**			
Analytical Validation of a Point‐of‐Care Assay for Pancreatic Lipase in Dogs and Cats	GI01		
Calcifediol Supplementation Safely Increases 25‐hydroxyvitamin D Concentrations in Dogs with Protein‐losing Enteropathy	GI02	Y	
Gastrointestinal Ulcers and Masses Diagnosed by Video Capsule Endoscopy in 26 Dogs	GI03	Y	
Effect of Low Dose Azithromycin on the Fecal Microbiome and Fecal Bile Acids in Dogs	GI04	Y	2025 ACVIM Resident Research Abstract Award
Evaluation of an Outpatient Protocol for the Management of Acute Pancreatitis in Dogs	GI05	Y	
Tolerability of At‐Home Nasoesophageal and Nasogastric Tube Feedings in Dogs and Cats	GI06	Y	
Establishment and Characterization of Canine Pancreatic Organoids	GI07		
Viable Bacteria Isolated from Lyophilized Stool Used for Fecal Microbiota Transplantation	GI08		
Localization of Glucose Transporter 2 RNA in Small Intestine of Dogs with Chronic Inflammatory Enteropathy	GI09		
Exploring the Gut Microbiome of Ferrets: Associations with Health Status and Microbial Composition	GI10		
Effect of Amoxicillin‐Clavulanate Treatment on the Fecal Lipidome in Healthy Adult Cats	GI11		
Microbiome and Liver Enzymes in Dogs with Chronic Enteropathies Receiving Fecal Microbiota Transplantation	GI12		
Correlation Between Serum Cobalamin Concentration, Histopathologic Changes, and Outcome in Cats with Chronic Enteropathy	GI13		
Blood Reticulocyte Hemoglobin Concentration as a Marker of Iron‐deficient Erythropoiesis in Canine Chronic Inflammatory Enteropathy	GI14		
Parasite and Pathogen Incidence and Frequency of Testing in a Companion Animal Stool Bank	GI15		
Safety, Tolerability, and Pharmacokinetics of a Canine Monoclonal Antibody for Treatment of Chronic Inflammatory Enteropathy	GI16		
Daily Monitoring of Fecal Microbiota Transplantation Response in Dogs: Preliminary Insights from the Owners' Perspective	GI17		
Retrospectively Characterizing Fecal Microbiota in Dogs with Acute Diarrhea using qPCR, Metagenomics, and Machine Learning	GI18		
Concurrent Management of Behavioral and Gastrointestinal Disorders in Dogs with Early‐life Trauma Improves Clinical Outcomes	GI19		
Serum and Fecal Bile Acid Profiles in Dogs with Chronic Enteropathy	GI20		
Comparison of Gallbladder Volume and Emptying Between Treated Hypothyroid Dogs and Healthy Dogs	GI21		
Outcome of Protein‐losing Enteropathy in Pugs in First‐opinion Practice: 69 Cases	GI22		
Serum Cobalamin and Methylmalonic Acid Concentrations in Emaciated Dogs with Long‐term Inadequate Nutrition	GI23		
**SMALL ANIMAL INTERNAL MEDICINE — HEMATOLOGY**			
Risk Factors Associated with Lipemic Plasma in a Cohort of Healthy Canine Blood Donors	HM01	Y	
Plasma Plasminogen Activator Inhibitor (PAI‐1) Activity in Dogs with Immune‐mediated Hemolytic Anemia	HM02	Y	2025 ACVIM Resident Research Abstract Award
Evaluation of the Ability of Babesia rossi to Induce Neutrophil Extracellular Trap Formation	HM03		
The Use of Cold Stored Platelets to Provide Hemostasis in Dogs with Thrombocytopenia	HM04		
Estimation of Red Blood Cell Indices in Dogs and Cats Based on Artificial Intelligence	HM05		
Evaluation of Cytokine Expression for Predicting Outcome and Adverse Effects in Canine Immune‐Mediated Hemolytic Anemia	HM06		
Identification of a Novel Common Red Blood Cell Antigen Lacking in a Boykin Spaniel	HM07		
**SMALL ANIMAL INTERNAL MEDICINE — HEPATOLOGY**			
Do Serum Pro‐inflammatory and Pro‐coagulant Biomarkers in Dogs with Gallbladder Mucoceles Predict Disease Progression?	HP01	Y	2025 ACVIM Resident Research Abstract Award
Safety of Chronic Copper‐Restricted Diet (0.11 mg/100 kcal) in Dogs with Congenital Portosystemic‐Vascular‐Anomalies	HP02		
Lower Risk for Liver Copper Accumulation in Dogs Fed ≤0.24 mg Copper /100 kcal	HP03		
Relative Exchangeable Copper and Ultrafiltered Urine Copper‐to‐Creatinine ratio for Diagnosis of Copper‐Associated Hepatitis in Dogs	HP04		
**SMALL ANIMAL INTERNAL MEDICINE — IMMUNOLOGY**			
Plasma ADAMTS13 Activity in Dogs with Severe Thrombocytopenia and Presumed Immune Thrombocytopenia	IM01	Y	2025 ACVIM Resident Research Abstract Award
Canine Autoimmune Lymphoproliferative Syndrome in Four Sibling Boerboel Puppies	IM02		
Antioxidant Effects of Gastric Acid Suppressants on Free Radicals from Ex Vivo Dog Neutrophils	IM03		
Defining the Nasal Transcriptome in Cats with Chronic Rhinitis	IM04		
Bulk RNA Sequencing of Blood from Dogs with Immune‐mediated Polyarthritis Reveals Diverse Inflammatory Pathway Activation	IM05		
**SMALL ANIMAL INTERNAL MEDICINE — INFECTIOUS DISEASE**			
Castellaniella spp. as a Potential Emerging Pathogen in Dogs	ID01	Y	
Agreement between Collection Techniques for Detection of Histoplasma Antigen in the Urine of Dogs	ID02	Y	
Negative or Low Levels of Antibodies in 27 Dogs with Clinical Leishmaniosis	ID03	Y	
Outbreak of Leptospirosis in Dogs from Los Angeles County, California, USA, 2021	ID04	Y	2025 ACVIM Resident Research Abstract Award
Vector‐borne infections in dogs with immune‐mediated hemolytic anemia, thrombocytopenia, or polyarthritis in Northern California	ID05	Y	
Canine Blastomycosis: A Retrospective Study of 126 Cases in Tennessee	ID06		
Performance of a Proprietary Silicon Surface Multiplex Immunoassay for Ehrlichia spp Antibody Detection in Dogs	ID07		
Use of Acute Phase Proteins in Cats with Feline Infectious Peritonitis	ID08		
Atovaquone Resistance M121I Polymorphism in Babesia gibsoni Isolates from Dogs	ID09		
Comparison of Gastrointestinal Parasite Frequency in Feral and Pet Cats in Houston, Texas	ID10		
Molnupiravir Pharmacokinetics Study in Cats with Naturally Occurring Feline Infectious Peritonitis	ID11		
Does Virulence Vary Amongst ‘Candidatus Mycoplasma haemominutum’ Isolates within a Geographical Region?	ID12		
Comparison of Two In‐House Screening Tests for Anaplasma and Ehrlichia spp. Antibody Detection in Dogs	ID13		
Field Validation of an Anaplasma and Ehrlichia spp. Antibody Screening Test in Dogs	ID14		
Echinococcus multilocularis in 3 dogs in Boulder County, Colorado	ID15		
Plasma and Complement Proteins Are Essential for the Antimicrobial Activity of Canine Platelet Lysate	ID16		
**SMALL ANIMAL INTERNAL MEDICINE — NUTRITION**			
Temporal Variability of the Canine Fecal Microbiome	SNM01		
Defining the Core Microbiome in Healthy Cats	SNM02		
**SMALL ANIMAL INTERNAL MEDICINE — OTHER**			
Do Owners Know Best? Clinical & Laboratory Findings in Reportedly Healthy Geriatric Cats	OT01		
Associations of Physical Factors with Gait Speed in Healthy Dogs as a Potential Frailty Evaluation	OT02		
The Effects of Intraoperative Intraperitoneal Fluid Administration on Perfusion Parameters in pregnant Queens Undergoing Ovariohysterectomy	OT03		
Assessment of Peptacetobacter hiranonis' In Vitro Ability to Convert Primary into Secondary Bile Acids	OT04		
**SMALL ANIMAL INTERNAL MEDICINE — PHARMACOLOGY**			
Effect of Inhaled Albuterol on Whole Blood Potassium and Plasma Insulin Concentrations in Cats	P01		
Therapeutic Drug Monitoring of Itraconazole in Dogs with Blastomycosis	P02		
Comparison of Cyclosporine Pharmacokinetics and Pharmacodynamics in Blood, Plasma, Liver, and Small Intestine in Dogs	P03		
**SMALL ANIMAL INTERNAL MEDICINE — RESPIRATORY**			
Using Histologic Examination to Determine If Chronic Bronchitis Is a Cause of Bronchomalacia in Dogs	R01	Y	
Prevalence of Tracheal Collapse (CTCS), Congenital Portosystemic Shunts (CPSS), or Both in Yorkshire Terriers	R02		
Unraveling Canine Infectious Respiratory Disease: Nasal Microbiome Differences in Healthy vs. Symptomatic Dogs	R03		
Effect of Chest Confirmation on Measured Expiratory Volumes and Lung Mechanics in Healthy Dogs	R04		

**Research Abstract Poster Program**

**Index of Abstracts**

**Table:** 

Presenter	Title	Sequence Number	Award Eligible	Award Winner
**CARDIOLOGY**				
Amanda Bentley	Predominant Mitral Valve Morphology in Dogs at Different Stages of Myxomatous Mitral Valve Disease	C19		
Ritu Verma	Transcriptomic Insights into Hypertrophic Cardiomyopathy in Feline Hearts	C20		
Soyoung Jeung	A Retrospective Study on the Therapeutic Potential of Mesenchymal Stem Cells in Early‐Stage MMVD	C21	Y	
Joanna Kaplan	Unraveling the Genetics of Feline Hypertrophic Cardiomyopathy: A Multiomics Study	C22		
Kentaro Kurogochi	Incidence and Risk Factors of Postoperative New‐Onset Atrial Fibrillation in Dogs Undergoing Mitral Valve Repair	C23		
Ta‐Li Lu	Comparative Efficacy of Injectable Moxidectin and Melarsomine Protocols for Canine Heartworm Treatment	C24		
Eve Hanks	Application of a Novel MicroRNA Platform to Cardiac Disease Diagnoses in Companion Animals	C25		
Dylan Deprospero	Effect of Moderate to Severe Hypochloremia on Survival in Dogs with Stable Congestive Heart Failure	C26	Y	
Marco Mazzarella	Breed Differences in Left Ventricular Early Inflow‐Outflow Index in Dogs with Myxomatous Mitral Valve Disease	C27		
Katherine Murphy	Agreement of Resting Respiratory Rate Measurements and Those Obtained by the Maven Collar in Dogs	C28		
Eve Hanks	Critically Appraised Topic: What is the Clinical Potential for MicroRNAs as Cardiac Biomarkers in Dogs?	C29		
Ha Suk Nam	Hemoglobin, Albumin, Lymphocyte, and Platelet Score as a Predictor of Short‐Term Mortality in Canine CHF	C30		
Taylor Reiter	Asymptomatic Arrhythmias in Aged, Apparently Healthy Dogs Screened for a Large, Multi‐center Clinical Trial	C31		
Mutsuki Umezawa	Evaluation of Dose‐dependent Effects of Dapagliflozin on Urinary Glucose and Plasma Ketone Bodies in Dogs	C32		
Laura Machado Ribas	Association of MicroRNAs with Sudden Death in Boxer Dogs with Arrhythmogenic Right Ventricular Cardiomyopathy	C33		
Jaechun Cho	Etiologies and Outcomes of Pericardial Effusion in Small‐Breed Dogs: Emphasis on Cardiogenic Causes	C34		
Peter Constable	Comparison of Two Digital Electrocardiographs and Their Associated Computer‐Based Analysis Programs for Recording Dog Electrocardiograms	C35		
Alexandria Goodyear	Computer‐Generated 3D Models in Veterinary Student Education: Improving Radiographic Interpretation of Canine Congenital Heart Disease	C36		
Autumn Harris	Renin‐angiotensin‐aldosterone System Metabolites and Enzymes in Dogs with Active and Chronic Congestive Heart Failure	C37		
Kentaro Kurogochi	The T‐wave Peak‐end to QT Ratio in Doberman Pinschers with Subclinical Dilated Cardiomyopathy	C38		
Corine Lavigne	Copeptin Hormone Concentrations in Dogs with Heart Disease and Its Relationship with Antidiuretic Hormone	C39		
Corine Lavigne	The Effect of Hypochloremia and Medications on Outcomes in Cats with Stable Congestive Heart Failure	C40		
Ilana Levinzon	Comparison of Holter Monitoring and AliveCor for Arrhythmia Detection in Scottish Deerhounds	C41		
Gerard O'Leary	Evaluating NXSCOPE for Generating PCG Cardiology Reports in Healthy and Cardiac‐Disordered Canines	C42		
Victor Rivas	A Novel Cardiac Troponin‐I Missense Variant Is Associated with Familial Hypertrophic Cardiomyopathy in Golden Retrievers	C43		
**EQUINE**				
Morgan Askins	Serum Total Adiponectin Variation over 24 Hours in Grazing Horses with and Without Insulin Dysregulation	E26		
Laszlo Hunyadi	Poor Air Quality in Texas is Associated with Asthma Phenotype in Horses	E28		
Gustavo Ferlini Agne	Comparison of Equine Antimicrobial Susceptibility Based on Minimum Inhibitory Concentration and Pharmacokinetic‐Pharmacodynamic Indices for Enrofloxacin	E29		
Kimberly Hallowell	Prevalence of Insulin Dysregulation in Sport Horses as Determined by Traditional and Stall‐Side Testing	E30		
Emily Sundman	Case Series of Nutritional Secondary Hyperparathyroidism in Horses in Hawaii	E31		
Francesca Freccero	Echocardiography in Hypoxemic Neonatal Foals: Preliminary Results	E32		
Emma Stapley	Comorbidities Associated with a Diagnosis of Pituitary Pars Intermedia Dysfunction in Primary Care Practice	E33		
Amjad Khan	Aerosolized Equid Alphaherpesvirus 1 and ‐4 Viral Particles Detection at an Equine Care Facility	E34		
Daniela Luethy	Preliminary Evaluation of an Immunoturbidometric Assay for Measurement of Total Adiponectin Concentration in Equids	E35		
Marcio Costa	Fecal and Intestinal Microbiota Profiles in Horses with Different Types of Surgical Colic	E36		
Ernesto Rojas‐Sanchez	Evaluation of Two Immunochromatographic Point of Care Tests for Detection of Coccidioides Antibodies in Horses	E37		
Lara Jimenez	The Role of Adrenal Activation in the Pathogenesis of Equine Metabolic Syndrome	E38		
Lisa Katz	The Response of Cathepsin B to Exercise and Training in Thoroughbred Racehorses	E39		
Hamid Tavanaeimanesh	Complex Arrhythmias in One Exercising ECG in Thoroughbred Racehorses Are Not Associated with Lifetime Performance	E40		
Wenqing Wang	Leukocyte Numbers and Morphological Changes in the Hindgut of Horses with Pituitary Pars Intermedia Dysfunction	E41		
Lisa Zimmer	Evaluating Equine Resting Behavior in Response to Housing Changes: Impact of Age and Adaptation Effect	E42		
Linda Paul	Pharmacokinetics of S‐Adenosylmethionine in Horses Using a Crossover Study Design with Three Dosing Regimens	E43		
Khadija Ayadi	Bronchodilatory Effect of Inhaled Lidocaine in Horses with Severe Asthma	E44		
Marcio Costa	Detection of Biomarkers Indicators of Intestinal Health Among the Equine Fecal Microbiota Using Quantitative PCR	E45		
Eva Buschmann	First Experience with Radiofrequency Ablation to Reduce Recurrence Risk of Atrial Fibrillation in Horses	E46		
Serena Ceriotti	Effect of Omeprazole Treatment on the Pharmacokinetics of Orally Administered Flunixin Meglumine in Adult Horses	E47		
Bethanie Cooper	Investigation of MARCKS Protein as a Novel Therapeutic Target to Decrease Nets in Equine Asthma	E48		
Daniela Espinosa	Antimicrobial Susceptibility of Bacterial Isolates Recovered from Sick Neonatal Foals	E49		
Olivia Jacob	Safety, Immunogenicity, and Protective Efficacy of an Equine Rotavirus B Vaccine	E50		
Daniel Jean	Antimicrobial Use in Pediatric Patients with Neonatal Infections in an Equine Teaching Hospital in 2023	E51		
Audrey Johnston	Establishing Metabolic Parameters in Mules	E52		
Ninja Karikoski	Changes in Serum Basal Insulin Concentration During Estrous Cycles in Healthy Mares	E53		
Rachel Lemcke	Endocrine Disorder Classifications and Clinical Sign Frequencies using Static and Dynamic Testing Among Horse Breeds	E54		
Rachel Lemcke	Frequency of Endocrine‐associated Clinical Signs in Equids with Different Risk Levels for Hyperinsulinemia‐associated Laminitis	E55		
Caitrin Lowndes	A Randomized, Placebo‐controlled Crossover Study of Bexagliflozin Treatment in Horses with Naturally‐occurring Insulin Dysregulation	E56		
Caitrin Lowndes	Decreased Adiponectin and Increased Leptin: Adiponectin are Associated with Insulin Dysregulation and Laminitis in Light‐breed Horses	E57		
Caroline Mckinney‐Aguirre	Placental‐derived Extracellular Vesicles Enhance in vitro Equine Intestinal Epithelial Repair	E58		
Sydney Mordoh	Effects of IL‐8 on Neutrophil Extracellular Traps (NETs) in Healthy Equine Neutrophils	E59		
Piper Norton	Clinical and Histological Chronological Characterization of Equine Nasopharyngeal Cicatrix Syndrome in 6 Horses in Texas	E60		
Elaine Norton	Epidemiological Evaluation of Equine Metabolic Syndrome in Arabian Horses and Their Subgroups	E61		
Hanna Sfraga	Post‐mortem Characterization of Subclinical Inflammation of Nuchal Bursa and Ligament in Horses	E62	Y	
Breanna Sheahan	Horses with Undifferentiated Colitis Exhibit Downregulation of a Choline Transporter Gene in Colon and Rectum	E63		
Gunther Van Loon	Orthodromic Atrioventricular Re‐entry Bradycardia and Tachycardia in Horses Due to an Accessory Pathway	E64		
**FOOD ANIMAL INTERNAL MEDICINE**				
Marcio Costa	Fecal Microbiota Dynamics in Calves During the First 24 Days in a Veal Unit	F09		
Eloi Guarnieri	Insights from Hong Kong's First Ruminant Ambulatory Service	F10		
Jennifer Halleran	Influence of Sample Collection Site on Blood Glucose Measurements in Goats	F11		
Grace Malla	The Pharmacokinetics and Pilot Pharmacodynamics of Subcutaneous Levamisole through a Combination Product in Domestic Goats	F12		
Lanie Phillips	Impact of Storage Duration on Erythrocyte Health and Biochemical Analytes in Caprine Blood Products	F13		
Joe Smith	The Pharmacodynamics and Pharmacokinetics of Acepromazine in Domestic Goats	F14		
Luisa De La Cuadra Rojas	The Effects of Polyclonal IgY Supplementation on the Health of Pre‐weaned Dairy Calves	F15		
Grace Jakes	The Impact of Stress on Bovine Pulmonary Mucosal Immunity	F16		
**NEUROLOGY**				
Stephanie Chang	Post‐surgical Outcomes in Paraplegic Dogs with Asymmetrical Loss of Nociception Secondary to Intervertebral Disc Herniation	N13	Y	
Katherine Simon	The Relationship Between Owner Assessments of Cognition and Quality of Life in Senior Companion Dogs	N14		
Stephanie Fonseca	Quantification of Effective Implant Size on CT for Cortical Stainless Steel Screws	N15	Y	
Joshuah Klutzke	Objective Assessment of a Novel Hearing Protection Device in Dogs	N16	Y	
Jungmin Lym	Evaluation of Hemoglobin, Albumin, Lymphocyte, and Platelet Score for Predicting Short‐term Mortality in Canine MUE	N17		
**NUTRITION**				
Brielle Gordon	Effects of Dietary Medium Chain Fatty Acids on Feline Renal Function	NM05		
Kiran Panickar	Changes in Circulating MicroRNAs and Their Potential Beneficial Effects in Canines Fed Dietary Vitamin D	NM06		
Camila Torelli	Serum Vitamin D Metabolites in Diabetic Dogs	NM07		
**ONCOLOGY**				
Karin Allenspach	Assessing Individual Variation in Chemoradiation Responses in Canine Bladder Cancer Organoids	O10		
Yunho Jeong	Hypoxia Enhances Vasculogenic Mimicry Formation in Canine Mammary Tumor Cell Lines	O11		
Keon Kim	Circulating Progranulin: A Promising Biomarker for Diagnosis and Prognostic Prediction in Canine Tumors	O12		
Jeongmin Lee	3D Organoid‐Based Drug Sensitivity Analysis for Personalized Chemotherapy in Canine Sarcoma	O13		
Yiyu Li	Retrospective Analysis of Canine Splenic Tumors: 54 Cases	O14		
Andre Meneses	Innovative Development of Organoids from Canine Mastocytomas: A Novel In Vitro Model Background	O15		
Yoon‐Ho Suh	Auranofin Induces Apoptosis and Contributes to Paraptosis‐Like Cell Death in Malignant Canine Mammary Tumor Cells	O16		
Brian Hansford	Environmental Chemical Exposures, Early DNA Damage, and Multicentric Lymphoma in Dogs	O17		
Jeffrey Bryan	Chimeric Antigen Receptor T Cells Can Be Tracked in vivo and Induce remission in Dogs	O18		
Christine Chang	Bacterial Extracellular Vesicles Decrease Viability of Canine Osteosarcoma Cells and Modulate T Cell Immune Response	O19		
Maria Dashek	Innovative Bioanalytical Luminescent Assays for Advancing Veterinary Oncology Therapeutics	O20		
Aleksandar Masic	Pharmacokinetic & Safety Assessment of a Novel Anti‐CD20 mAb	O21		
**SMALL ANIMAL INTERNAL MEDICINE—CARDIOLOGY**				
Manlik Kwong	First Step Toward Precision Veterinary Clinical Decision‐support Development Incorporating Physiological and Electronic Health Records	SC02		
Seung‐Bum Cho	CD40‐CD40 Ligand‐Mediated Cardiorenal Injury in Canine Myxomatous Mitral Valve Disease	SC03		
Jae Hun Kim	Impact of Pulmonary Hypertension for Hepatobiliary System: Retrospective Study in 33 Dogs	SC04		
**SMALL ANIMAL INTERNAL MEDICINE—ENDOCRINOLOGY**				
Álan Pöppl	Endogenous ACTH as an Aid for Primary Hypoadrenocorticism Diagnosis After Glucocorticoid Treatment Beginning	EN07		
Maxime Derre	Free Thyroxine Concentration by Chemiluminescent Immunoassay and Equilibrium Dialysis in Euthyroid Dogs with Nonthyroidal Illness	EN08	Y	
Álan Pöppl	Hypersomatotropism Low‐Prevalence Among Diabetic Cats from Porto Alegre City in Southern Brazil: Preliminary Report	EN09		
Chaeyeon Park	Risk Factors Associated with Elevated Serum Specific Canine Pancreatic Lipase in Dogs with Hypercortisolism	EN10		
Yunjin Sung	Machine Learning Algorithms as a Screening Tool for Hypercortisolism in Dogs	EN11		
Priscila Viau	Evaluation of Serum Insulin Measurement in Dogs: A New Perspective	EN12		
Viviani De Marco	Dapagliflozin as Once‐daily Therapy for Feline Diabetes Mellitus	EN13		
Arielle Johnson‐Pitt	Serological Evidence of Pancreatic Inflammation in Dogs with Diabetes Mellitus	EN14		
Florence Leduc	Comparison of Thyroid Stimulating Hormone Measurements Between Chemiluminescent Immunoassay and Bulk Acoustic Wave Assay	EN15	Y	
**SMALL ANIMAL INTERNAL MEDICINE—GASTROENTEROLOGY**				
Chih‐Chun Chen	Fecal Concentrations of Carbohydrates in Cats with Chronic Enteropathy	GI24		
Albert Jergens	Duodenum Mucosa Genome‐Wide DNA Methylation Profiling in Canine Inflammatory Enteropathy	GI25		
Joonghyun Song	Clinical Efficacy and Safety of Fexuprazan in Canine Chronic Gastritis	GI26		
Ricardo Duarte	AI‐Assisted Meta‐Analysis on the Use of Hypoallergenic Diets for the Treatment of Canine Chronic Enteropathies	GI27		
Abby Goldberg	Post‐mortem Evaluation of Esophageal Diameters in Dogs and Cats: A Descriptive Study in 352 Animals	GI28	Y	
Emily Moore	Evaluation of Immune System Components in Dogs with Protein‐losing Enteropathy versus Healthy	GI29	Y	
Marine Rullier	Fecal Microbiota Transplant as an Adjunctive Treatment of Canine Chronic Pruritic Dermatitis	GI30		
Elisa Scarsella	Description and Insights of the Feline and Canine Gut Mycobiome	GI31		
**SMALL ANIMAL INTERNAL MEDICINE—HEMATOLOGY**				
Hyunjin Kim	Postoperative Thrombocytosis and Hypercoagulability Based on Thromboelastography Over 28 Days After Splenectomy	HM08		
Jaewoo Park	Platelet Function Analysis Using the PFA‐200 in Geriatric Dogs	HM09		
Clare Timothy	Indications for Out‐patient Packed Red Blood Cell Transfusion in Dogs: 43 Cases (2014‐2024)	HM10		
Allison Collier	Comparing Anti‐globulin Enhanced Major Crossmatch Test Methods in Cats	HM12		
**SMALL ANIMAL INTERNAL MEDICINE—HEPATOLOGY**				
Sharon Center	Coyote Liver Copper Compared to Dogs Implicates Excessive Dietary Copper Supplementation in Commercial Dog Food	HP05		
Rommaneeya Leela‐Arporn	Transcriptomic Comparison of Liver FNAs and Biopsies in Healthy Cats	HP06	Y	
Paige Albers	Signalment, Clinicopathologic, and Imaging Findings in Dogs with Cirrhotic and Non‐cirrhotic Causes of Portal Hypertension	HP07	Y	
Floris Dröes	Serum and Fecal Bile Acid Profiles in Dogs with Liver Disease	HP08		
**SMALL ANIMAL INTERNAL MEDICINE—IMMUNOLOGY**				
Sangyoon Choi	Enhanced Anti‐Inflammatory Effects of 3D‐Cultured Feline Adipose Tissue Derived Mesenchymal Stem Cells	IM06		
Eunbi Lee	Improved Anti‐inflammatory Effects of Canine Adipose Tissue‐Derived Mesenchymal Stem Cells Using 3D Culture	IM07		
Lynna Li	Leptospirosis Vaccine Responses Are Maintained in a Cohort of Alaskan Sled Dogs Aged 8‐14	IM08		
Ga‐Hyun Lim	Assessment of NK Cell Activity for Immunity Evaluation in Dogs	IM09		
Kevin Gooch	Disease Impact on Prednisolone Pharmacokinetic and Pharmacodynamic Parameters in Dogs with Immune‐mediated Diseases or Lymphoma	IM10	Y	
**SMALL ANIMAL INTERNAL MEDICINE—INFECTIOUS DISEASE**				
Pablo Jimenez Castro	Giardia Duodenalis in US Dogs and Cats: Differences in Positive Proportion and Zoonotic Potential	ID17		
Peyton Wagner	Identifying Dogs at Risk of Antimicrobial Resistant Infections with Machine‐learning: Escherichia coli Bacteriuria in Dogs	ID18		
Pablo Jimenez Castro	Lungworm Species Typing Using Molecular Diagnostics in an International Sample Cohort	ID19		
Peyton Wagner	Machine Learning Tools to Identify Cats with Bacterial Cystitis When Exhibiting Lower Urinary Tract Signs	ID20		
Emily Teng	Baylisascaris procyonis (Raccoon Roundworm) Detection in Dogs and Cats from the United States and Canada	ID21	Y	
Petra Cerna	Assessing Fecal Lipidome in Cats Recovering from Feline Infectious Peritonitis After Antiviral Therapy	ID22		
Izumi Kida	Development of Direct Whole‐genome Sequencing of Feline Coronavirus for Understanding Pathogenesis and Drug Resistance	ID23		
Meera Surendran‐Nair	Validation of an In‐House Diagnostic Screening Test for Borrelia burgdorferi Antibodies in Naturally Exposed Dogs	ID24		
Graham Bilbrough	Performance of a Silicon Surface Multiplex Immunoassay for Anaplasma spp. Antibody Detection in Dogs	ID25		
Jean Carlos Alvarado Colon	Effects of Molnupiravir on Clinical Remission and Calicivirus Shedding in Cats with Chronic Gingivostomatitis	ID26		
Sarah Eusebi	Diagnostic Application of Machine Learning Models for Predicting Feline Infectious Peritonitis	ID27		
Ingo Schäfer	Resistance Against Allopurinol in Three Dogs Infected with Leishmania Infantum	ID28		
**SMALL ANIMAL INTERNAL MEDICINE—NEPHROLOGY/UROLOGY**				
Kerrigan Fleming	Investigating Serum Growth Differentiation factor‐8 Concentrations in Cats with Early Chronic Kidney Disease	NU14		
Andre Meneses	Renal Cortical Thickness to Aortic Diameter Ratio: A Novel Ultrasound Marker for Feline Kidney Disease	NU15		
Josh Chang	Retrospective Study of Cystoscopic‐guided Laser Ablation of Intramural Ectopic Ureters in Male Dogs	NU16	Y	
William Whitehouse	Evaluation of Oxidative Stress in Nonazotemic Cats with Increased Symmetric Dimethylarginine Concentrations	NU17		
Luciano Giovaninni	Quantitative and Qualitative Analysis of Renal Proteinuria in 25 Dogs with Spontaneous Chronic Kidney Disease	NU18		
Seungju Kang	Elevated Trimethylamine N‐Oxide Levels in Canine Chronic Kidney Disease: TMAO as a Potential Renal Biomarker	NU19		
Victória Gomes	Urinary Calcium and Urinary Magnesium in Cats: A Validation Study	NU20		
Nuttha Hengtrakul	The Co‐occurrence Between Nephrocalcinosis and Nephrolithiasis in Feline Kidneys	NU21		
Matheus Leite	Urinary Calcium, Magnesium and Phosphorus in Dogs: A Validation Study	NU22		
Cory Penn	Assessment of an Evolved Deep Learning Artificial Intelligence Algorithm for Canine/feline Urine Sediment Samples	NU23		
Mary Lewis	Deep Learning Algorithm for Evaluation of Less Common Crystals and Spermatozoa in Canine/Feline Urine Sediment	NU24		
Ignacio Lopez	Modulation of Fecal Microbiota in Dogs with Chronic Kidney Disease Supplemented with Probiotics	NU25		
Jessica Quimby	Sparse Sampling Pharmacokinetics of Pregabalin in Cats with Chronic Kidney Disease	NU26		
Danielle Santos	Acid‐base and Electrolyte Disorders in Dogs with Leptospirosis: Preliminary Results	NU27		
Kate Sarkan	Electrophoretic Urine Protein Banding Patterns in Healthy Cats and Cats with Kidney Disease	NU28		
**SMALL ANIMAL INTERNAL MEDICINE—NUTRITION**				
Deborah Linder	Establishing a Reference Interval for Postprandial Triglyceride Concentrations in Healthy Adult Dogs	SNM03	Y	
Monique Paludetti	Case Description of Canine Gallbladder Mucocele: Retrospective Study of 60 Cases	SNM04		
**SMALL ANIMAL INTERNAL MEDICINE—OTHER**				
Kiyeon Son	Analysis of Beta‐Adrenergic Receptor Expression in Canine Splenic Hemangiosarcoma	OT05		
Hyunyoung Cho	Delineating the Fecal Microbiome of Healthy Domestic Short‐Hair Cats in South Korea	OT06		
Sydney Holland	Participant Compliance with Scheduled Activities in the Test of Rapamycin in Aging Dogs (TRIAD)	OT07		
Matthew Jackson	Dogs and Cats have Differential Immunometabolic Responses to Lipopolysaccharide in Cultured Circulating Cells	OT08		
Matthew Peloquin	Multiomic Analysis of Canine Aging Uncovers Conserved Aging Pathways	OT09		
Arielle Johnson‐Pitt	Owner‐Reported Health Conditions in UK Miniature Schnauzers	OT10		
**SMALL ANIMAL INTERNAL MEDICINE—PHARMACOLOGY**				
Kate Kukanich	Fluoroquinolones Are a Risk Factor for Cranial Cruciate Ruptures in Retrievers	P04		
**SMALL ANIMAL INTERNAL MEDICINE—RESPIRATORY**				
Hye‐In Park	Comparison of Conventional Oxygen Therapy and High‐flow Nasal Cannula in Dogs with Non‐cardiogenic Pulmonary Edema	R05		
Jennifer Weng	Soft Palate Defects as a Cause of Chronic Nasal Signs in 3 Cats	R06	Y	

## 1 ABSTRACT C01: Hemodynamic Effects of Non‐Ionic Contrast Prior to Patent Ductus Arteriosus Occlusion and Pulmonic Balloon Valvuloplasty

### 1.1 **Alix Saavedra** ^1^; James Karnia^2^, DVM, DACVR; Stacey Leach^3^; Laura Nafe^2^, DVM, MS, DACVIM (SAIM); Anna McManamey^2^, DVM, DACVIM‐Cardiology; Kelly Wiggen^2^, DVM, DACVIM‐Cardiology

#### 1.1.1 ^1^Cardiology Resident, University of Missouri, Columbia, MO, USA; ^2^University of Missouri, Columbia, MO, USA; ^3^Missouri Veterinary Cardiology, LLC., Columbia, MO, USA

**Background:** The hemodynamic effects of non‐ionic contrast (iohexol) administration on invasive pressure measurements in dogs have not been evaluated.

**Hypothesis/Objective:** To evaluate effects of iohexol on invasive pressure assessment in dogs undergoing patent ductus arteriosus (PDA) occlusion or balloon valvuloplasty (BVP) for pulmonic stenosis (PS). The authors hypothesized there would not be a statistically or clinically relevant difference in measurements obtained pre‐ and post‐iohexol administration.

**Animals:** Client owned dogs undergoing PDA occlusion (*n* = 15) or BVP (*n* = 15).

**Methods:** Prospective observational study. Invasive pressures for PDA (main pulmonary artery (MPA) and aorta) and PS dogs (right ventricle and MPA) were obtained. Heart rate and direct peripheral blood pressure (BP) were recorded simultaneously. Pressures acquired prior to contrast injection (t0), 60–90 seconds post‐injection (t1), and 5 min post‐injection (t2). The mean pressure difference between time points was calculated, including peak‐to‐peak gradient (P2P) for PS dogs.

**Results:** There was a significant mean difference for the following: PDA group: diastolic and mean arterial pressure in the aorta between t1 and t0 (*p* = 0.019, 0.003) and diastolic BP between t2 and t0 (*p* = 0.020); in the PS group: MPA systolic BP between t1 and t0 (*p* = 0.02). There were wide 95% tolerance limits for most measurements, including P2P change between t1‐0 and t2‐0.

**Conclusions and Clinical Importance:** Although mean pressure change was generally not statistically significant, tolerance intervals suggest large changes can occur in some animals. Wide 95% tolerance limits for P2P could lead to misinterpretation of surgical success intraoperatively. Ideally, invasive pressure measurements should be obtained prior to contrast administration.

## 2 ABSTRACT C02: Coadministration of Omecamtiv Mecarbil and Pimobendan: Observation of Safety and Efficacy (The COMPOSE Study)

### 2.1 **Phillip Lawson** ^1^, II; Victor Rivas^2^, MS, PhD; Kentaro Kurogochi^2^, DVM, PhD; Caitlin Hargrove^2^, DVM; Sandra Losa^2^, PhD, MBA; Aida Gonzalez^2^; Megan Leber^2^, BS; Sarah Holdt^3^, VMD, DACVIM (Cardiology); Bill Tyrrell^3^, DVM, DACVIM (Cardiology); Joshua Stern^2^, DVM, PhD, DACVIM (Cardiology)

#### 2.1.1 ^1^Cardiology Resident, CVCA—Cardiac Care for Pets; ^2^Stern Lab/College of Veterinary Medicine, North Carolina State University, Raleigh, NC, USA; ^3^CVCA

**Background:** Inotropic support is a mainstay of congestive heart failure therapy and an area of novel drug discovery. Omecamtiv mecarbil (OM) is a sarcomere modulator that targets the catalytic S1 domain of cardiac myosin. Pimobendan and OM support systolic function through two specific mechanisms of the actin‐myosin binding complex; however, their synergistic action is unknown.

**Hypothesis/Objectives:** We hypothesize that combination therapy of OM and pimobendan will be safe and effective in increasing canine myocardial systolic function.

**Animals:** 6 healthy adult purpose‐bred dogs

**Methods:** A single‐blinded, placebo‐controlled, crossover pharmacodynamic study was carried out. Five echocardiographic timepoints were compared. On day 1, each dog received vehicle (echo 1) followed by intravenous pimobendan bolus (0.15 mg/kg; echo 2) followed by a 30‐day washout period. Vehicle was repeated (echo 3) followed by OM (0.25 mg/kg IV bolus and 0.25 mg/kg/h IV CRI × 15 min; echo 4), followed by the final treatment with dual therapy (OM 0.25 mg/kg/h IV CRI plus pimobendan at 0.15 mg/kg IV).

**Results:** Change in echocardiographic values from same‐day vehicle control were calculated, normality tested and compared with the corresponding parametric (one‐way ANOVA and Tukey's multiple comparisons test) or non‐parametric equivalent (Friedman's and Dunn's multiple comparisons test). Left ventricular ejection fraction was significantly increased (*p* < 0.006) with dual therapy compared to OM or pimobendan.

**Conclusions and Clinical Importance:** Dual therapy was well‐tolerated and improved systolic function compared to pimobendan or OM alone. Future studies in dogs with cardiovascular disease are indicated.

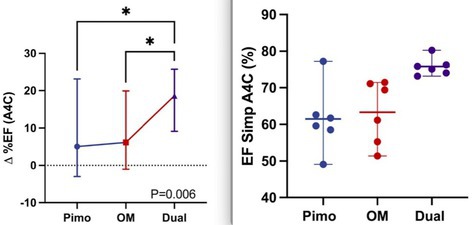

## 3 ABSTRACT C03: Comparing Two‐Dimensional Crescentic Ellipsoid Model Variants in Estimating Three‐Dimensional Echocardiographic Right Ventricular Volume in Dogs

### 3.1 **Robert Ciardullo** ^1^; Brittany Tagg^2^; Shana Mintz^3^, DVM, DACVIM (Cardiology); Romain Pariaut^4^, DVM, DACVIM (Cardiology), DECVIM‐CA (Cardiology); Weihow Hsue^3^, DVM, DACVIM (Cardiology)

#### 3.1.1 ^1^Resident, Cornell University, Ithaca, NY, USA; ^2^Student, Cornell University, Ithaca, NY, USA; ^3^Assistant Professor, Clinical Sciences, Cornell University, Ithaca, NY, USA; ^4^Associate Professor, Clinical Sciences, Cornell University, Ithaca, NY, USA

**Background:** Determining right ventricular (RV) volume typically requires three‐dimensional imaging due to its complex shape. The crescentic ellipsoid model (CEM) offers a two‐dimensional alternative, employing area‐ or linear‐based formulas (Figure 1) with further variations based on echocardiographic views (Figure 2).

**Hypothesis/Objectives:** To identify which CEM variant best agrees with real‐time three‐dimensional echocardiography (RT3D) as a reference standard and to assess within‐day reproducibility.

**Animals:** Sixty‐seven client‐owned dogs (23 normal, 44 with right‐sided heart diseases) underwent echocardiograms, with 20 normal dogs receiving a repeat exam.

**Methods:** Prospective method comparison study. Body weight‐indexed end‐diastolic (iEDV) and end‐systolic volumes (iESV) were calculated across eight CEM variants. Agreement with RT3D was assessed using concordance correlation coefficients (rc) and Bland‐Altman analysis, while within‐day reproducibility was evaluated using intraclass correlation coefficients (ICC) and reproducibility coefficients.

**Results:** The area‐ and linear‐based CEMs using RV parameters from the left apical four‐chamber view and cardiac diameter from the right parasternal short‐axis view (CEM_A4C‐RPS and LCEM_A4C‐RPS, respectively) were the only methods to achieve moderate agreement with RT3D (rc > 0.90). The CEM_A4C‐RPS showed no significant systematic bias for iEDV (median of the differences [95% confidence interval]: 0.09 [0.00–0.13]), while LCEM_A4C‐RPS displayed no significant systematic bias for iEDV (0.03 [−0.02 to 0.08]) and iESV (0.04 [−0.01 to 0.12]). Both methods demonstrated good reproducibility for iEDV and iESV (ICC > 0.75), with iESV reproducibility significantly greater than that of methods using RV parameters from the right parasternal long‐axis view.

**Conclusions and Clinical Importance:** The CEM_A4C‐RPS and LCEM_A4C‐RPS provide clinically practical RV volume estimates.

**Figure:**
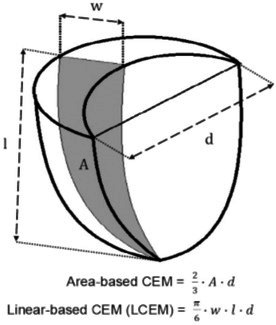
**Figure 1**: Crescentic ellipsoid model (CEM) formulas. A, right ventricular cross‐sectional area; w, right ventricular basilar width.

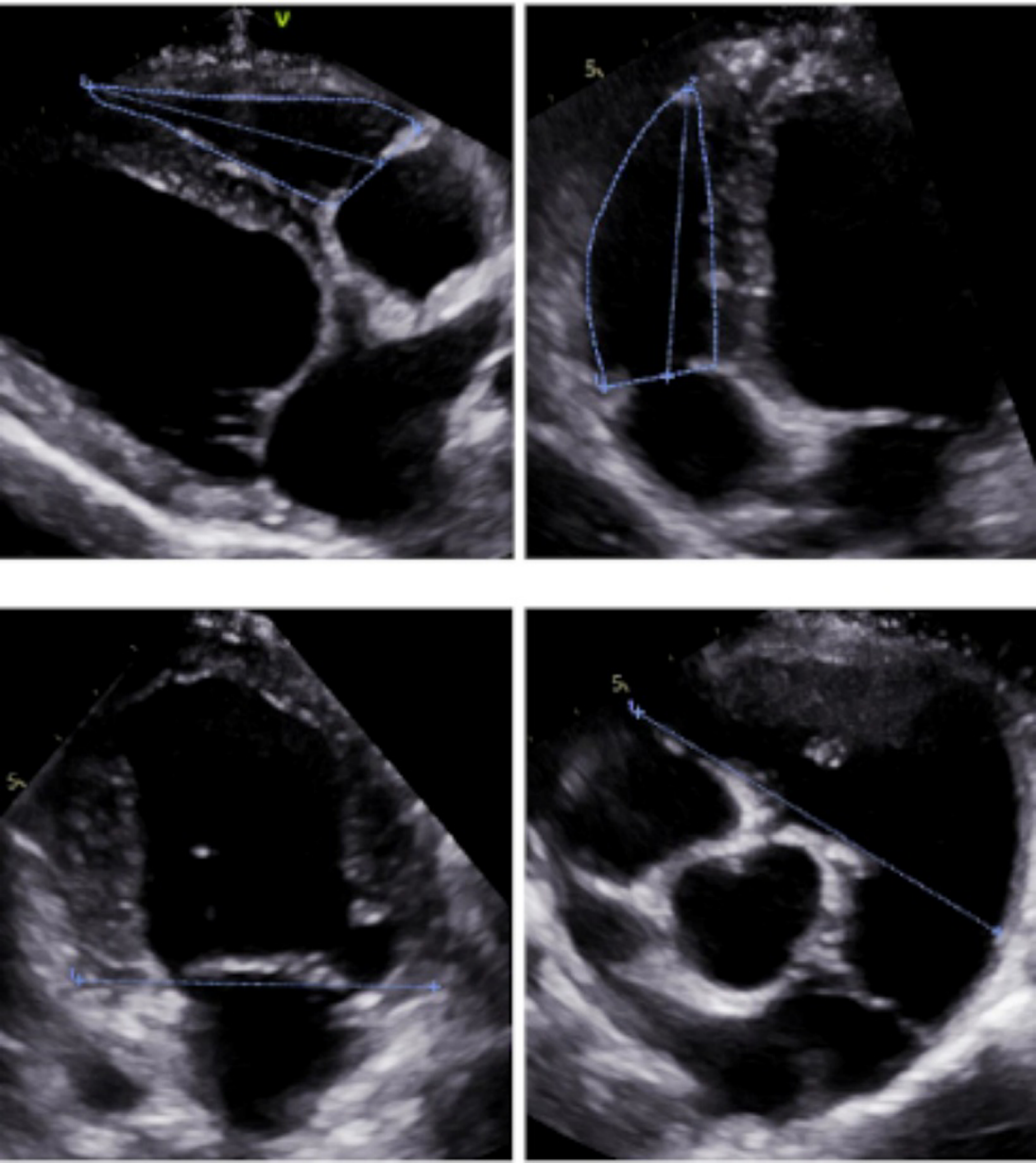

## 4 ABSTRACT C04: Segmental Left Ventricular Systolic Excursion in Cats with Hypertrophic Cardiomyopathy

### 4.1 **Eve Lo** ^1^; David Connolly^2^, BSc, BVetMed, PhD, CertVC, CertSAM, DECVIM (Cardiology), MRCVS; Virginia Luis Fuentes^2^, MA, VetMB, PhD, CertVR, DVC, MRCVS, DACVIM (Cardiology), DECVIM‐CA (Cardiology)

#### 4.1.1 ^1^Resident in Cardiology, Royal Veterinary College, London, UK; ^2^Professor in Veterinary Cardiology, Royal Veterinary College, London, UK

**Background:** Left ventricular (LV) systolic dysfunction is associated with a poor prognosis in cats with hypertrophic cardiomyopathy (HCM). Echocardiographic strain techniques are used in people to demonstrate regional wall motion abnormalities, but these techniques require optimal image quality and advanced software. We demonstrate the use of M‐mode echocardiography to evaluate regional radial wall motion in cats.

**Hypothesis/Objectives:** In cats with HCM stage B2 and C, LV free wall (LVFW) systolic excursion (SE) is lower than septal SE.

**Animals:** 75 client‐owned cats, including 12 healthy cats and 63 cats with HCM (stage B1, *n* = 24; stage B2, *n* = 20 and stage C, *n* = 19).

**Methods:** One observer reviewed right parasternal short‐axis cineloops from our echocardiographic records. Healthy cats and cats with HCM with normal sinus rhythm and acceptable image quality were included. Left ventricular short‐axis images at the papillary muscle level were divided into 3 septal and 3 LVFW myocardial segments. Anatomical M‐mode was used for measuring the SE of each radial segment.

**Results:** Mean systolic excursion of all six segments was progressively lower comparing stage B1, B2, and stage C cats (*p* < 0.0001). In stage B2 cats the SE in the LVFW and septum were similar (*p* = 0.608), whereas stage C cats had lower SE in the LVFW (2.8 mm ± 1.2 mm) than the septum (4.0 mm ± 1.3 mm; *p* < 0.0083).

**Conclusions and Clinical Importance:** Regional systolic dysfunction is present even in subclinical stages of HCM, and progressively worsens with advancing stage of HCM. This effect is more marked in the LVFW.

## 5 ABSTRACT C05: The Use of Contrast‐Enhanced Echocardiography in the Evaluation of Feline Left Ventricular Wall Thickness

### 5.1 **Emily Javery** ^1^; Saki Kadotani^2^; Carl Toborowsky^2^; Sumana Parbhakar^2^; Todd Sumerfield^2^; Kimberly Selting^2^; Ryan Fries^2^

#### 5.1.1 ^1^Cardiology Resident, University of Illinois at Urbana‐Champaign, Champaign, IL, USA; ^2^University of Illinois, Champaign, IL, USA

**Background:** Repeatable and reliable measurements of the feline left ventricular (LV) wall are difficult to obtain due to irregularities of the endocardium, false tendons, asymmetric hypertrophy, and prominent papillary muscles. Contrast‐enhanced echocardiography (C‐Echo) is utilized in human medicine to optimize measurements of the LV wall and is more accurate, repeatable, and reliable than standard echocardiography (S‐Echo).

**Hypothesis/Objectives:** Compare C‐Echo and S‐Echo measurements of LV wall thickness (LVWT) and determine repeatability, reproducibility, and reliability for each modality.

**Animals:** Fifty client‐owned cats; LVWT <5 mm (*n* = 30), LVWT 5–6 mm (*n* = 8), and LVWT > 6 mm (*n* = 12).

**Methods:** Prospective observational study. All cats underwent S‐Echo by two observers and C‐Echo by one observer after intravenous injection of 0.2–0.4 mL contrast agent (Lumason, sulfur hexafluoride lipid‐type‐A microspheres). Methods were compared using Bland‐Altman and Passing‐Bablok regression. Inter‐ and intraoperator reproducibility were quantified using intraclass correlation (ICC) and 95% repeatability/reproducibility coefficients (RC).

**Results:** Method comparison between S‐Echo and C‐Echo demonstrated significant differences in LVWT for all segments. Globally, the median difference in LVWT between S‐Echo and C‐Echo was 0.56 mm (range, −1.4 to 4.9 mm; *p* < 0.0001) and regression showed a proportional bias of 1.21 mm (95% CI 1.11–1.32). Excellent intra‐ and interoperator reproducibility was observed for S‐Echo and C‐Echo based on ICC 0.89–0.97 and 0.87–0.94, respectively with lower RCs for C‐Echo (0.28 mm–0.61 mm) compared with S‐Echo (0.89–1.03 mm).

**Conclusions and Clinical Importance:** Compared with S‐Echo, C‐Echo yields lower LVWT measurements, excellent visualization of the LV wall, and superior repeatability and reproducibility.

## 6 ABSTRACT C06: Quality of Life Assessment in Dogs Undergoing Mitral Transcatheter Edge‐to‐Edge Repair With the V‐Clamp Device

### 6.1 **Hannah Melhorn** ^1^; Katie Abbott‐Johnson^2^; Emily Karlin^3^; John Rush^3^; Brianna Potter^2^; Lisa Freeman^3^; I‐Jung Chi^3^

#### 6.1.1 ^1^Cardiology Resident, Tufts University, Medford, MA, USA; ^2^Veterinary Teaching Hospital, Colorado State University, Fort Collins, CO, USA; ^3^Cummings School of Veterinary Medicine at Tufts University, North Grafton, MA, USA

**Background:** Owner‐perceived quality of life (QoL) is important in dogs with advanced heart disease. The effect of transcatheter edge‐to‐edge repair (TEER) on QoL is unknown.

**Hypothesis/Objectives:** Quality of life as determined by the Functional EvaluaTion of Cardiac Health (FETCH) questionnaire and biochemical parameters will improve following TEER of the mitral valve.

**Animals:** Thirty‐seven client‐owned dogs undergoing TEER at two centers.

**Methods:** Dogs were prospectively enrolled after obtaining informed consent. Owners completed a FETCH questionnaire with mean weekly respiratory rate (RR) and cough frequency at baseline, 2–4 weeks (recheck 1) and 2–4 months (recheck 2) post TEER. Baseline NT‐proBNP and serum biochemistry values were compared between timepoints. Echocardiography was performed at baseline and at recheck 2. Preliminary results were analyzed using Wilcoxon signed‐rank test (between 2 time points) or Friedman test (between 3 timepoints); Spearman correlations were used to test potential associations between echocardiographic measurements and FETCH scores.

**Results:** FETCH scores improved from baseline at recheck 1 (*p* = 0.04) and recheck 2 (*p* = 0.02; overall *p* < 0.001; *n* = 20). TEER resulted in smaller left ventricular internal dimension in diastole (LVIDdN; *n* = 21, *p* = 0.001) and left atrium to aorta ratio (LA:Ao; *n* = 21, *p* = 0.01). LVIDdN and LA:Ao were positively correlated with FETCH scores at recheck 2 (*r* = 0.75, *p* < .001; *r* = 0.67, *p* = 0.002, respectively). Respiratory rate decreased following TEER (*p* = 0.001, *n* = 19). Serum potassium remained within the reference range but increased over time (*p* < 0.001, *n* = 14). NT‐proBNP results are pending.

**Conclusions and Clinical Importance:** Cardiac health‐related QoL, RR, and left heart dimensions were improved after TEER with the canine V‐Clamp device.

## 7 ABSTRACT C07: Evaluation of Mitral Valve Leaflet Morphology in Healthy Beagle Dogs Using Three‐Dimensional Transesophageal Echocardiography

### 7.1 **Marta Karn** ^1^; Brianna Potter^2^; Lalida Tantisuwat^2^; Lance Visser^2^; E. Orton^2^; Brian Scansen^2^

#### 7.1.1 ^1^Cardiology Resident, Colorado State University, Fort Collins, CO, USA; ^2^Colorado State University, Fort Collins, CO, USA

**Background:** Description of the normal subleaflet anatomy of the mitral valve (MV) in dogs using three‐dimensional (3D) transesophageal echocardiography (TEE) has not been well described. With advancements in MV interventions, understanding leaflet morphology becomes increasingly important.

**Hypothesis/Objectives:** To describe the subleaflet anatomy of the MV in apparently healthy beagle dogs. Variation in the subleaflet anatomy was hypothesized, with posterior leaflet anatomy expected to vary more than the anterior.

**Animals:** Twenty healthy beagle dogs.

**Methods:** Prospective, descriptive study. Comprehensive TEE studies were performed and 3D images acquired. Studies were evaluated and MV anatomy was classified as classic or atypical. Classic MV anatomy was defined as a non‐scalloped anterior leaflet and a posterior leaflet with only three scallops. Atypical anatomy referred to patients with accessory scallops and/or dichotomous posterior leaflet scallops.

**Results:** Classic MV anatomy was observed in 45% (9/20) of dogs. Accessory scallops were present in 45% (9/20) of dogs. The anterior leaflet comprised an average of 24.9% (±3.3%) of the mitral annular circumference. Variation in scallop size was observed, with the percentage of annular circumference occupied by each scallop ranging from P1: 19.2–29.7%, P2: 19.4–32.1%, P3: 16.6–27.7%. Which scallop was largest varied among dogs, with P1 being the largest in 11/20 (55%), P2 in 6/20 dogs (30%), and P3 in 3/20 dogs (15%).

**Conclusions:** Variations in subleaflet morphology are present in apparently healthy dogs, with most variations noted in the posterior leaflet. Accessory and dichotomous posterior scallops are common in healthy beagle dogs.

## 8 ABSTRACT C08: Echocardiographic Evaluation of Aortic Root Size in Cats With Hypertrophic Cardiomyopathy

### 8.1 **Eline Nijveldt** ^1^; Karsten Schober^2^, DVM, PHD, DECVIM‐CA; Vedat Yildiz^3^

#### 8.1.1 ^1^Resident in Cardiology and Interventional Medicine, College of Veterinary Medicine, The Ohio State University, Columbus, OH, USA; ^2^Professor; Cardiology and Interventional Medicine Section Head, Cardiology, College of Veterinary Medicine, The Ohio State University, Columbus, OH, USA; ^3^Center for Biostatistics, The Ohio State University, Columbus, OH, USA

**Background:** In addition to left ventricular hypertrophy (LVH), enlargement of the mitral valve is commonly observed in hypertrophic cardiomyopathy (HCM) in cats. Whether aortic root size (ARS) is increased in HCM is unknown.

**Hypothesis/Objectives:** To determine ARS in cats with HCM and compare ARS to control cats and cats with hyperthyroidism (HT4) and systemic hypertension (SH). We hypothesized that cats with HCM have increased ARS.

**Animals:** 180 client‐owned cats.

**Methods:** Retrospective echocardiographic observational study. Ten two‐dimensional echocardiographic variables characterizing ARS were measured (linear dimension of the aortic valve (AV), the sinus of Valsalva (SV), the sinotubular junction (STJ), the ascending aorta (AAo), and the sinus height (SH) in systole and diastole). Results were compared using standard univariate and multivariate statistical tests for group comparison.

**Results:** 130 cats with HCM, 15 with HT4, and 15 with SH were studied. Univariate analysis revealed a difference (*p* < 0.01) between HCM and control only for the STJ measured in diastole and systole (median, 7.44 vs. 7.03 mm and 7.70 vs. 6.90 mm, respectively), the SV in systole (9.99 vs. 9.67 mm), and the AV in systole (7.06 vs. 6.66 mm). However, multivariable analysis only found age (*p* < 0.001), body weight (BW, *p* < 0.001), and presence of SH (*p* < 0.001) but not HCM and HT4 associated with ARS.

**Conclusions and Clinical Importance:** Cats with HCM do not have aortic root enlargement beyond that expected by BW and aging while ARS is consistently increased in cats with SH. These data are helpful differentiating causes of LVH in cats by echocardiography.

## 9 ABSTRACT C09: Demographic, Clinical, and Echocardiographic Characteristics of Dilated Cardiomyopathy in Dogs Eating Hydrolyzed Diets

### 9.1 **Bradley Whelchel** ^1^; Yiru Ma^2^; John Rush^3^, DVM, MS, DACVIM (Cardiology), DACVECC; Lisa Freeman^3^, DVM, PhD, DACVIM (Nutrition)

#### 9.1.1 ^1^Cardiology Resident, Cummings School of Veterinary Medicine at Tufts University, North Grafton, MA, USA; ^2^Cummings School of Veterinary Medicine at Tufts University, North Grafton, MA, USA; ^3^Professor, Cummings School of Veterinary Medicine at Tufts University, North Grafton, MA, USA

**Background:** Diet‐associated dilated cardiomyopathy (DCM) can occur in dogs eating high‐pulse diets, and diet change can lead to clinical and echocardiographic improvements. There have been anecdotal reports of DCM in dogs eating hydrolyzed diets.

**Hypothesis/Objectives:** To determine the prevalence and characteristics of dogs with DCM eating a variety of hydrolyzed diets.

**Animals:** 2,222 dogs diagnosed with DCM.

**Methods:** Medical records of dogs diagnosed with DCM at a nationwide cardiology specialty practice between May 2018 and October 2023 were retrospectively reviewed. Demographic variables were compared between dogs eating hydrolyzed diets vs. non‐hydrolyzed diets. Clinical and echocardiographic data were recorded.

**Results:** Twenty‐eight dogs (1.3%) were eating hydrolyzed diets prior to DCM diagnosis. The most common breeds were pit bull (*n* = 6), English bulldog (*n* = 4), and Doberman pinscher (*n* = 4). Arrhythmias were seen in 17/28 dogs, and 17/28 dogs had congestive heart failure. Age (media*n* = 9.4 years; *p* = 0.47), sex (19 male, 9 females; *p* = 0.97), and body weight (media*n* = 33.2 kg; *p* = 0.52) of dogs eating hydrolyzed diet were not statistically different from dogs eating non‐hydrolyzed diets. Fourteen of the twenty‐eight dogs had follow‐up echocardiograms. For the five dogs that changed diet, there was a significant reduction in left atrial to aortic ratio (LA/Ao) at the follow‐up exam (*p* = 0.04), whereas there was no significant change in LA/Ao in the nine dogs that did not change diet (*p* = 0.72).

**Conclusions and Clinical Importance:** Dilated cardiomyopathy can occur in dogs eating hydrolyzed diets. The role of diet is unclear and warrants further investigation.

## 10 ABSTRACT C10: Bionote Point‐of‐Care Quantitative NT‐Probnp Assay for Differentiating Stages of Preclinical Disease in Dogs and Cats

### 10.1 **Samantha Sotillo** ^1^; Emily Javery^2^, DVM; Saki Kadotani^2^, DVM, DACVIM (Cardiology); Sumana Prabhakar^2^, DVM; Todd Sumerfield^2^, DVM; Carl Toborowsky^2^, VMD, DACVIM (Cardiology); Ryan Fries^2^, DVM, DACVIM (Cardiology), DECVIM‐SA (Cardiology)

#### 10.1.1 ^1^Resident in Veterinary Cardiology, Thrive Pet Healthcare Specialists North Scottsdale, Scottsdale, AZ, USA; ^2^University of Illinois, Urbana, IL, USA

**Background:** Quantitative N‐terminal pro‐brain natriuretic peptide (NT‐proBNP) is useful in combination with examination findings to stage preclinical cardiac disease in dogs and cats. Quantitative point‐of‐care (POC) NT‐proBNP assays have not been evaluated for this purpose.

**Objectives:** Assess reliability of Bionote POC quantitative NT‐proBNP to differentiate between ACVIM stages B1 and B2 in myxomatous mitral valve disease dogs and hypertrophic cardiomyopathy cats. The ACVIM consensus statement was used to differentiate stages, and B2 cats required a left atrial diameter > 17 mm.

**Animals:** A total of 96 dogs and 53 cats with stage B1 (51 dogs, 31 cats) or B2 (45 dogs, 22 cats) cardiac disease.

**Methods:** Animals with echocardiographic evidence of cardiac disease and no comorbidities were prospectively enrolled. Bionote POC quantitative NT‐proBNP was assess for all animals.

**Results:** Stage B2 animals had higher (*p* = 0.003) median NT‐proBNP concentrations (cat, 886 pmol/L; dog, 1206 pmol/L) compared with B1 (cat, 199 pmol/L; dog, 539 pmol/L). Receiving operating characteristics (ROC) analysis indicated excellent discriminatory ability in dogs (AUC = 0.96, *p* < 0.0001) and good in cats (AUC = 0.83, *p* < 0.0001). Based on Youden index, a cutoff in dogs of > 888 pmol/L achieved a sensitivity of 87% and specificity of 94%, while a cutoff of > 1004 pmol/L achieved a specificity of 100%. A cutoff in cats of > 342 pmol/L achieved a sensitivity of 86% and specificity of 74%, while a cutoff > 1332 pmol/L achieved a specificity of 100%.

**Conclusions and Clinical Importance:** POC quantitative NT‐proBNP is useful for disease staging preclinical cardiac disease in dogs and cats.

## 11 ABSTRACT C11: Canine Hindlimb Function and Perfusion Following Femoral Arterial Ligation or Repair for Transcatheter Vascular Occlusion

### 11.1 **Seth Bowden** ^1^; Kursten Pierce^2^, DVM, DACVIM (Cardiology); Bruce Keene^2^, DVM, MSc, DACVIM (Cardiology); Chin‐Chieh Yang^3^, DVM; Amanda Valentino^2^, MS, RVT; Alejandra Mondino, DVM, Msc, PhD; Natasha Olby^2^, Vet MB, PhD, MRCVS, DACVIM (Neurology)

#### 11.1.1 ^1^Cardiology Resident, North Carolina State University, Raleigh, NC, USA; ^2^North Carolina State University, Raleigh, NC, USA; ^3^Université de Montréal, Montreal, QC, Canada

**Background:** Transcatheter occlusion is the treatment of choice for patent ductus arteriosus (PDA) in dogs. Following PDA occlusion, it is commonplace to ligate the femoral artery at the arterial puncture site. Ligation appears to be clinically well‐tolerated by patients, however, there are no published data comparing femoral artery ligation to repair.

**Hypothesis/Objectives:** Repairing the femoral artery results in improved hindlimb perfusion and less hindlimb dysfunction when compared to ligation.

**Animals:** Eleven client‐owned dogs (> 5 kg) underwent transcatheter device occlusion of left‐to‐right shunting PDA. Five dogs underwent femoral arterial repair, and 6 dogs underwent arterial ligation.

**Methods:** Orthopedic examination, static posturography, thigh girth measurement, creatinine kinase, infrared thermography, and vascular ultrasound were performed at baseline, 1 day, 10–14 days, and 90 days post‐procedure.

**Results:** Diagnostic results were reviewed in a blinded fashion. The changes in outcome variables were compared over time using mixed effect models. There was significantly increased blood flow to the distal limb on vascular ultrasound in patients with arterial repair. Of the ligation group, partial return of femoral artery flow was observed in 44.4% of dogs at 90 days post‐ligation. The remainder of the diagnostics performed did not reveal clinically significant differences between groups at postop timepoints (Day 1, Day 10–14, Day 90).

**Conclusion and Clinical Importance:** In the short‐term, femoral artery ligation appears to be well tolerated with no clinically appreciable deficits in hindlimb perfusion and function in dogs undergoing PDA occlusion. It is uncertain whether clinically significant differences occur long‐term for these patients.

## 12 ABSTRACT C12: Accuracy of Echocardiographic Estimates of Mitral and Aortic Annular Area in Dogs with Mitral Regurgitation

### 12.1 **Riley Ellis‐Reis** ^1^; Lance Visser^2^; I‐Jung Chi^3^; Brian Scansen^2^

#### 12.1.1 ^1^Cardiology Resident, Colorado State University, Fort Collins, CO, USA; ^2^Colorado State University, Fort Collins, CO, USA; ^3^Tufts University, Medford, MA, USA

**Background:** Echocardiographic estimates of mitral (MAA) and aortic annular area (AAA) are used to calculate regurgitant volume. The accuracy of MAA and AAA in dogs with degenerative mitral valve disease (DMVD) are unknown.

**Hypothesis/Objectives:** To determine the accuracy of echocardiographic estimates of MAA and AAA compared to planimetered annular area using cardiac computed tomography (CCT) in dogs with DMVD.

**Animals:** Twenty dogs with ACVIM stage B2 DMVD.

**Methods:** Observational study on dogs that underwent echocardiography and CCT on the same day. Echocardiographic mid‐diastolic MAA was estimated from a right parasternal long‐axis (RPLx) 4‐chamber and left apical 4‐chamber (AP4C) view. Echocardiographic early systolic AAA was estimated from a RPLx outflow and a left cranial (LCr) view. Annular area was estimated using the formula: diameter (leaflet hinge‐points) 2 × 0.785. Pearson's correlation coefficients and Bland‐Altman's method were used to compare CCT and echocardiography.

**Results:** Significant associations were identified when comparing AAA_RPLx (*r* = 0.87, 95% CI=0.70, 0.95) and AAA_LCr (*r* = 0.94, 95% CI = 0.85, 0.98) to AAA_CCT and when comparing MAA_RPLx4C (*r* = 0.86, 95% CI=0.68, 0.94) and MAA_AP4C (*r* = 0.90, 95% CI=0.77, 0.96) to MAA_CCT. Bias and limits of agreement for AAA_RPLx (−0.29 [−0.80, 0.21] cm2) and AAA_LCr (−0.25 [−0.63, 0.13] cm^2^) were smaller compared to MAA_RPLx4C (−0.71 [−2.06, 0.63] cm2) and MAA_AP4C (−1.14 [−2.24, −0.05] cm^2^). All comparisons exhibited significant (*p* < 0.001) fixed bias.

**Conclusions/Clinical Importance:** Echocardiographic estimates of annular area, particularly MAA, underestimated annular size compared to CCT. Indices of regurgitant volume utilizing MAA might be inaccurate in dogs with DMVD.

## 13 ABSTRACT C13: Effects of Zenalpha® (Medetomidine/Vatinoxan) on Blood Pressure, Echocardiographic Measurements, and Cardiac Rhythm in Healthy Dogs

### 13.1 **Kiersten Forsyth** ^1^; Daniel Hogan^2^, DVM, DACVIM (Cardiology); Luis Dos Santos^3^, DVM, PhD, DACVIM (Cardiology); Hsin‐Yi Weng^4^, BVM, MPH, PhD

#### 13.1.1 ^1^Cardiology Resident, College of Veterinary Medicine, Purdue University, West Lafayette, IN, USA; ^2^Professor, Cardiology, Department of Veterinary Clinical Sciences, College of Veterinary Medicine, Purdue University, West Lafayette, IN, USA; ^3^Assistant Professor, Cardiology, Department of Veterinary Clinical Sciences, College of Veterinary Medicine, Purdue University, West Lafayette, IN, USA; ^4^Associate Professor, Clinical/Analytical Epidemiology, Department of Comparative Pathobiology, College of Veterinary Medicine, Purdue University, West Lafayette, IN, USA

**Background:** The effect of Zenalpha® (medetomidine/vatinoxan) on echocardiographic parameters and cardiac rhythm in healthy adult dogs has not been evaluated.

**Hypothesis/Objectives:** To determine the effects of Zenalpha® on blood pressure, echocardiographic parameters, and cardiac rhythm in healthy dogs.

**Animals:** Twenty‐one healthy, adult, client‐owned dogs

**Methods:** Dogs were enrolled in a prospective, pre‐test/post‐test study. Echocardiography, blood pressure, and 1‐h Holter monitor were performed at baseline and repeated after sedation with Zenalpha® (1 mg medetomidine/m^2^). Multiple echocardiographic parameters (M‐mode, 2D, volumetric‐SMOD, Doppler, and tissue Doppler), blood pressure, and an arrhythmic burden score were assessed using pairwise comparisons between baseline and sedation.

**Results:** Compared to baseline, Zenalpha® sedation was associated with significant changes in the following parameters: reduction in blood pressure (*p* < 0.001), prolongation of PR (*p* < 0.001), and QT (*p* < 0.001), reduction in HR (*p* < 0.001), and reduction in eight systolic function parameters: EF (MM,2D,SMOD), %FS (MM,2D), ESVI (SMOD), SVI (SMOD), %LVAS (2D), all *p* < 0.001; and an increase in arrhythmic burden score (*p* = 0.006). Thirteen of 21 dogs (62%) developed or had worsening of AV nodal conduction on Holter while only 2/21 (9.5%) developed or had worsening atrial or ventricular ectopic activity. Of all measured echocardiographic systolic function parameters, 12/21 (57%) dogs had two or more outside of reference range with Zenalpha® sedation. Of the parameters EF‐SMOD, ESVI, %FS, and %LVAS, 5/21 (24%) had two or more outside of reference range.

**Conclusions and Clinical Importance:** Sedation with Zenalpha® can result in altered ECG and echocardiographic measurements, which should be considered when used for echocardiographic studies.

## 14 ABSTRACT C14: Coronary Ostial Eccentricity, Aortic Rotation, and Cusp Symmetry in Dogs Evaluated by Cardiac Computed Tomography

### 14.1 **Cassidy Coats** ^1^; Brian Scansen^2^, DVM, MS, DACVIM (Cardiology)

#### 14.1.1 ^1^Cardiology Resident, Colorado State University, Fort Collins, CO, USA; Professor, Clinical Sciences, Colorado State University, Fort Collins, CO, USA

**Background:** Coronary ostia may vary in location within the aortic cusps. Brachycephalic dogs have an increased risk for anomalous coronary artery anatomy (aCAA). The aim of this study was to evaluate aortic root rotation and asymmetry of aortic cusp size and their association with aCAA in dogs.

**Hypothesis/Objectives:** Coronary ostia are not centrally located within the aortic cusps and this eccentricity is greater in brachycephalic dogs. Aortic root rotation and aortic cusp asymmetry are associated with aCAA.

**Animals:** 105 dogs with cardiac computed tomography including 22 normal, 19 PS with aCAA, and 64 PS with normal CAA.

**Methods:** Retrospective, observational study. Each coronary ostial opening was determined as an angle relative to aortic cusp midpoint. Symmetry of the aortic cusps was determined by planimetered area. Rotation of the aortic root was calculated as deviation of the midpoint of the non‐coronary cusp from the plane of the interatrial septum.

**Results:** The left coronary ostium of dogs had a median eccentricity of 19.5 degrees (95% CI: 14.3–22.5) toward the left‐right commissure; no difference was observed between brachycephalic and non‐brachycephalic dogs (*p* = 0.3). The area of the cusps were not different in dogs with aCAA as compared to dogs with normal CAA (*p* > 0.3). Aortic root rotation was greater in brachycephalic dogs (16.7 ± 20.3 degrees) than in non‐brachycephalic dogs (6.1 ± 15.7; *p* = 0.01).

**Conclusions and Clinical Importance:** Brachycephalic dogs have aortic root rotation. Presence of aCAA is not related to asymmetry of the aortic cusps. The pathoetiology of aCAA remains uncertain.

## 15 ABSTRACT C15: Detection of Congestive Heart Failure by Left Atrial Booster Strain in Cats with Cardiomyopathy

### 15.1 **Kei Sato** ^1^; Kensuke Nakamura^2^, DVM, PhD, DAiCVIM (Cardiology); Sei Kawamoto^3^, DVM; Mei Sugawara^3^, DVM, PhD; Kazuyoshi Sasaoka^3^, DVM, PhD; Nozomu Yokoyama^4^, DVM, PhD; Noboru Sasaki^5^, DVM, PhD; Keitaro Morishita^2^, DVM, PhD, DAiCVIM (Internal Medicine); Mitsuyoshi Takiguchi^6^, DVM, PhD, DAiCVIM (Internal Medicine)

#### 15.1.1 ^1^PhD Student, Hokkaido University, Sapporo, Japan; ^2^Associate Professor, Veterinary Clinical Science, Hokkaido University, Sapporo, Japan; ^3^Assistant Professor, Veterinary Teaching Hospital, Hokkaido University, Sapporo, Japan; ^4^Assistant Professor, Veterinary Clinical Science, Hokkaido University, Sapporo, Japan; ^5^Lecturer, Veterinary Teaching Hospital, Hokkaido University, Sapporo, Japan; ^6^Professor, Veterinary Clinical Science, Hokkaido University, Sapporo, Japan

**Background:** Left atrial (LA) strain is a valuable indicator of cardiac disease severity in humans and dogs. However, booster pump function cannot be assessed in many cats due to their high heart rates, limiting its clinical application.

**Hypothesis/Objectives:** To evaluate the feasibility of assessing booster pump function using high frame rate strain echocardiography in cats and its clinical utility.

**Animals:** Forty‐one client‐owned cats with hypertrophic or restrictive cardiomyopathy phenotypes.

**Methods:** Cross‐sectional study. Cats were classified as stage B or C according to the American College of Veterinary Internal Medicine consensus. Strain analysis images were obtained from the left apical four‐chamber view, with the sector width minimized to enhance the frame rate. Diagnostic performance for detecting stage C was assessed using receiver operating characteristic (ROC) curves.

**Results:** Booster pump function was successfully evaluated in 35 of 41 cats with 88–429 frames per second. ROC analysis showed that reservoir strain (area under the curve [AUC] 1.00; cutoff 12.0%; sensitivity 100%; specificity 100%) and booster strain (AUC 1.00; cutoff 6.51%; sensitivity 100%; specificity 100%) outperformed conventional LA size indices, including LA diameter (AUC 0.995; cutoff 17.7 mm; sensitivity 100%; specificity 94.1%) and LA to aortic root ratio (AUC 0.985; cutoff 1.89; sensitivity 100%; specificity 92.3%), in detecting stage C.

**Conclusions and Clinical Importance:** Booster pump function could be assessed in many cats using high frame rate strain echocardiography, demonstrating its value in identifying stage C. Further studies are needed to verify its clinical usefulness in predicting the risk of heart failure and thromboembolism.

## 16 ABSTRACT C16: Development of a Second‐Generation Vascular Occluder for Patent Ductus Arteriosus Occlusion in Dogs

### 16.1 **Min Su Kim** ^1^; Darcy Adin^2^, DVM, DACVIM (Cardiology); Michael Aherne^3^, MVB, GradDipVetStud, MS, MANZCVS (Small Animal Surgery), DACVIM (Cardiology); Dongmin Sihn^4^, DVM; Kyoung‐a Youp^5^, DVM, MS; Hakyoung Yoon^6^, DVM, PhD; Daekyu Oh^7^, DVM, MS; Nuri Lee^8^, DVM, MS; Seongyong Park^9^, DVM, MS; Daeyun Seo^10^, DVM, PhD; Seongsoo Lim^10^, DVM; Beomkwan Namgoong^10^, DVM; Heesung Umh^10^, DVM, MS; Nanju Lee^10^, DVM; Isong Kim^10^, DVM; Jihwan Kang^10^, DVM; Cheyoun Kim^10^, DVM; Seunghun Heo^10^, DVM; Hayoung Shin^10^, DVM

#### 16.1.1 ^1^Professor, College of Veterinary Medicine and Research Institute of Veterinary Science, Seoul National University, Seoul, Republic of Korea; ^2^Professor, College of Veterinary Medicine, University of Florida, Gainesville, FL, USA; ^3^College of Veterinary Medicine, University of Florida, Gainesville, FL, USA; ^4^Ilsan Animal Medical Center, Goyang‐si, Gyeonggi‐do, Republic of Korea; ^5^Korea Animal Medical Center, Seowon‐gu Cheongju‐si, Republic of Korea; ^6^Jeonbuk Animal Medical Center, College of Veterinary Medicine, Jeonbuk National University, Jeonju, Republic of Korea; ^7^Nowon N Animal Medical Center, Seoul, Republic of Korea; ^8^Yeeun Animal Medical Center; ^9^Gaeyang Sky Animal Medical Center; ^10^College of Veterinary Medicine, Seoul National University, Seoul, Republic of Korea

**Background:** We previously described a new canine patent ductus arteriosus (PDA) occlusion system in 2021. Dogs treated with the first‐generation device were monitored for residual flow over three years. Residual flow persisted in about 30% of cases. Subsequently, a second‐generation device was developed to address the issue of residual flow, based on a morphologic study of PDAs using CT. The device can be secured at the pulmonary ostium and is available with various waist diameter sizes.

**Hypothesis/Objectives:** To describe the use of a second‐generation occluder to close PDA.

**Animals:** Thirteen dogs with PDA from July 2024 to December 2024.

**Methods:** We retrospectively evaluated echocardiographic recordings in 13 dogs that underwent PDA occlusion using the second‐generation vascular occlude at 1‐ and 30‐days post‐occlusion. Occluder size and residual flow were recorded. Occluder was chosen by the sizes of pulmonary ostium and ampulla.

**Results:** Mean body weight was 3.70 kg (2.28–7 kg). All dogs had a PDA classified as type 2a, with a mean pulmonary ostium size of 2.7 mm (1.8–3.82 mm) by echocardiography. Occluder sizes used included 6 mm (*n* = 2), 7 mm (*n* = 2), 8 mm (*n* = 5), 9 mm (*n* = 3), and 10 mm (*n* = 1). Complete occlusion of the PDA was achieved, with no residual flow detected at both 1 day and 1‐month post‐procedure in all 13 dogs.

**Conclusions and Clinical Importance:** This second‐generation PDA occluder appear to result in more complete ductal occlusion without residual flow when compared to the first‐generation occluder.

**Figure:**
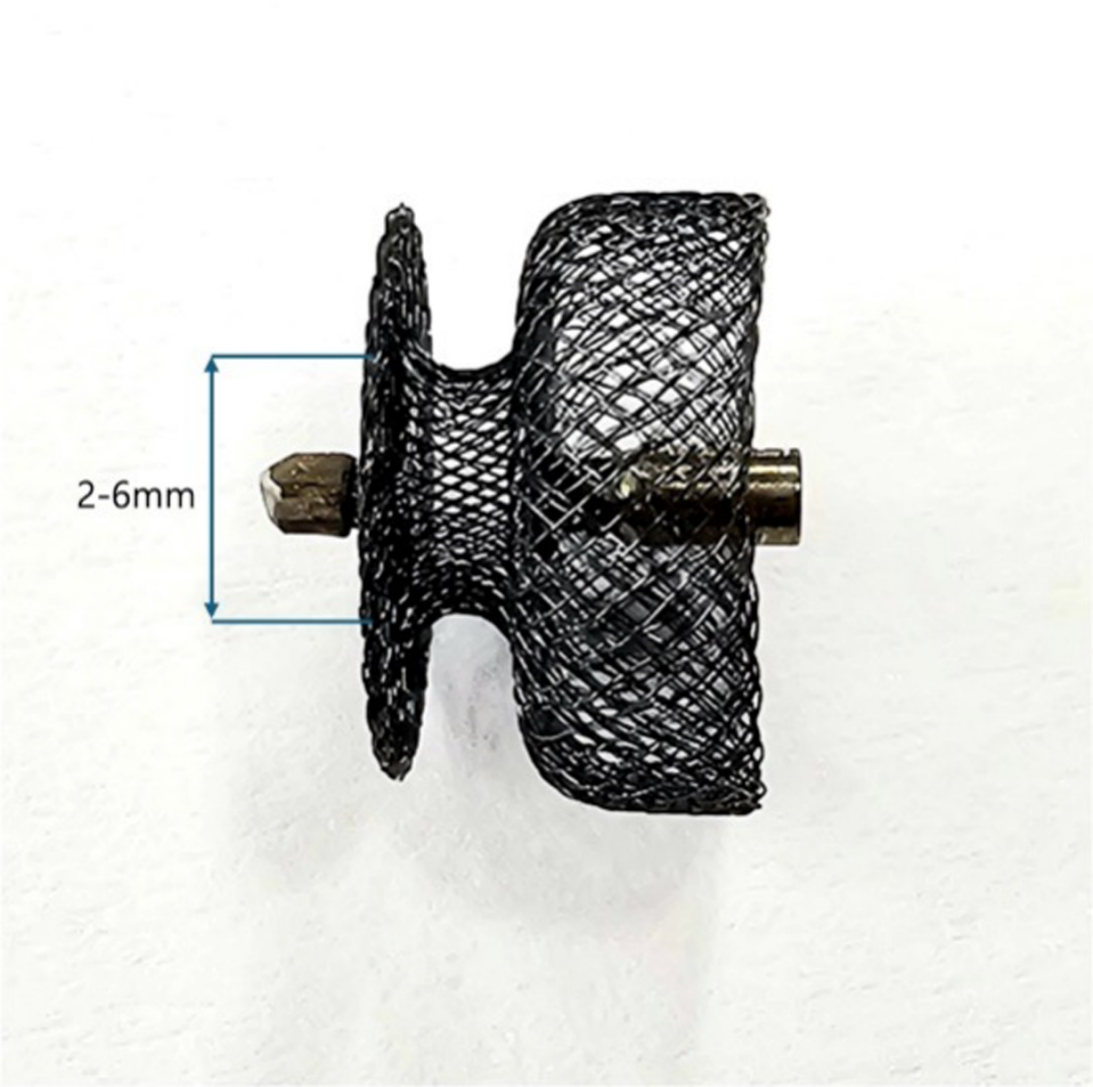
**Figure 1** Second‐generation vascular occluder

## 17 ABSTRACT C17: Renin‐Angiotensin‐Aldosterone System Activation in Dogs With Moderate to Severe Hypochloremia and Congestive Heart Failure

### 17.1 **Darcy Adin** ^1^; Oliver Domenig^2^, PhD; Autumn Harris^3^, DVM, DACVIM (SAIM)

#### 17.1.1 ^1^Clinical Professor, Cardiology, University of Florida, Gainesville, FL, USA; ^2^Attoquant Diagnostics, Vienna, Austria; ^3^Associate Professor, North Carolina State University, Raleigh, NC, USA

**Background:** Hypochloremia is associated with poor outcomes in people with congestive heart failure (CHF) and advanced heart disease in dogs, but etiology is uncertain.

**Hypothesis/Objectives:** We hypothesized that dogs with serum chloride concentrations ([Cl‐]) < 100 mEq/L would show greater renin‐angiotensin‐aldosterone system (RAAS) activation than dogs with [Cl‐] ≥ 100 mEq/L.

**Animals:** Eighty‐nine dogs with CHF.

**Methods:** Prospective study. RAAS metabolites and enzymes were quantified by liquid chromatography‐mass spectrometry/mass‐spectroscopy using previously validated methods and compared between dogs with [Cl‐] ≥ or < 100 mEq/L using Mann–Whitney test. Comparison of the proportion of dogs with active (pulmonary edema or effusions requiring intravenous furosemide) and stable (no edema or effusions and receiving oral furosemide or torsemide) CHF in each group was tested with Fisher's exact test. Significance was set at *p* < 0.05.

**Results:** Sixteen dogs had [Cl‐] < 100 (all degenerative valve disease) and 73 CHF dogs had [Cl‐] ≥ 100 (55 degenerative valve disease, 15 DCM, 3 congenital). Angiotensin II (*p* = 0.002), angiotensin III (*p* = 0.002), angiotensin 1–5 (*p* = 0.0005), and angiotensin IV (*p* = 0.002) were higher in dogs with [Cl‐] < 100 compared to dogs with [Cl‐] ≥ 100. No group differences were found for angiotensin I, angiotensin 1–7, aldosterone, angiotensin‐converting enzyme (ACE) activity, or ACE2 activity. Approximately half of the dogs with [Cl‐] < 100 had active CHF (44%) and half had stable CHF (56%), although the proportion of dogs with active CHF was greater in the group with [Cl‐] < 100 (*p* = 0.01).

**Conclusions and Clinical Importance:** Dogs with moderate to severe hypochloremia have greater RAAS activation compared to dogs with [Cl‐] ≥ 100 mEq/L, which might contribute to poor outcomes.

## 18 ABSTRACT C18: Canine Cardiomyopathy and Taurine Deficiency in Multiple Breeds

### 18.1 **Mikkel Bjerknaes** ^1^; Karin Kriström^2^, DECVIM (Cardiology); Jens Häggstrom^2^, DECVIM (Cardiology), PhD; Anna Tidholm^3^, DECVIM (Cardiology), PhD; Paul Pion^4^, DACVIM (Cardiology); Ingrid Ljungvall^2^, DECVIM (Cardiology), PhD

#### 18.1.1 ^1^Resident, Swedish University of Agricultural Sciences, Uppsala, Sweden; ^2^Swedish University of Agricultural Sciences, Uppsala, Sweden; ^3^Anicura Albano, Danderyd, Sweden; ^4^Veterinary Information Network, Davis, CA, USA

**Background:** Taurine deficiency (TD) and its association with a dilated cardiomyopathy phenotype (DCMp) is well described in cocker spaniel breeds. Taurine is currently considered a conditionally essential canine amino acid, and consequences of TD in the general dog population may be underappreciated.

**Objectives:** To retrospectively examine the prevalence of low taurine concentrations in dogs tested for TD, the prevalence of low taurine concentration in dogs with an echocardiographic diagnosis of a DCMp, and, furthermore, to describe dog characteristics and clinical findings in dogs diagnosed with low taurine concentrations.

**Animals:** 310 client‐owned dogs evaluated at the cardiology units of three animal hospitals in Sweden between 2018 and 2024.

**Methods:** Retrospective study of dogs that, following echocardiographic examination and at the clinician's discretion, had EDTA‐plasma analysed for taurine concentrations, because of ventricular systolic dysfunction, and/or a DCMp.

**Results:** Low taurine concentrations (below reference ranges) were found in 78/241 (32%) of dogs screened for TD. Dogs from 29 breeds, of which 55/78 (71%) were non‐cocker spaniel breeds, presented with low taurine concentrations. Of 148/217 (68%) dogs diagnosed with a DCMp and tested for TD, 68 (46%) had low taurine concentrations, yielding a cumulative period prevalence of 31% for dogs with DCMp. Low concentrations were found in 45/68 (66%) of TD tested dogs diagnosed with DCMp and congestive heart failure.

**Conclusion:** Nearly half of the taurine tested dogs with a DCMp had low taurine concentrations, and low concentrations were observed in approximately 30 breeds. The impact of TD in dogs should be further explored.

## 19 ABSTRACT C19: Predominant Mitral Valve Morphology in Dogs at Different Stages of Myxomatous Mitral Valve Disease

### 19.1 **Amanda Bentley** ^1^, DVM; Saki Kadotani^2^; Carl Toborowsky^2^; Ryan Fries^2^

#### 19.1.1 ^1^Cardiology Specialty Intern, University of Illinois; ^2^University of Illinois

**Background:** Myxomatous mitral valve disease (MMVD) is the most commonly acquired heart disease in dogs. Clinical symptoms and signs of cardiac remodeling on echocardiography are surrogates for disease severity. Other findings, such as mitral valve morphology, are not routinely considered, yet may play a crucial role in disease progression. Echocardiographic classification of mitral valve morphology is established in human medicine but has not been evaluated in canine MMVD.

**Objectives:** To compare the predominant mitral valve morphology (billow, prolapse, prolapse‐flail, or isolated flail) and leaflets affected (anterior, posterior, both) in dogs with MMVD ACVIM stages B1, B2, and C.

**Animals:** Dogs (*n* = 1108) with an echocardiographic diagnosis of MMVD ACVIM B1 (595), B2 (314), and C (199) weighing 1–15 kg.

**Methods:** Echocardiographic studies were retrospectively evaluated. Affected leaflets were identified from the transthoracic right parasternal and left apical four chamber views and Barlow's 1985 classification was used to determine the predominant morphology.

**Results:** Single anterior leaflet involvement was more common in stages B1 (79%) and B2 (77%) than C (53%) (*P* < 0.004). Billow significantly decreases across stages B1 (79%), B2 (17%), and C (5%) (*P* < 0.0001). Prolapse was significantly more common in stages B2 (47%) and C (32%), than B1 (15%) (*P* < 0.0001) as was isolated flail B2 (10%), C (8%), B1 (1%) (*P* < 0.0001). Prolapse‐flail significantly increases across stages B1 (6%), B2 (25%), and C (56%) (*P* < 0.0001).

**Conclusions and Clinical Importance:** Morphologic features of the MV vary significantly across stages of MMVD. These features may impact progression and treatment recommendations.

## 20 ABSTRACT C20: Transcriptomic Insights Into Hypertrophic Cardiomyopathy in Feline Hearts

### 20.1 **Ritu Verma** ^1^, MS; Megan Allan^2^; Jeff Caswell^3^, DVM, PhD; Sonja Fonfara^4^, DVM, PhD; Jessica Joshua^5^, PhD; Shari Raheb^6^, DVM, DAVCIM; Jeremy Simpson^7^, PhD

#### 20.1.1 ^1^PhD Student, University of Guelph; ^2^Student, Clinical Studies, University of Guelph; ^3^Professor, Pathobiology, University of Guelph; ^4^Professor, Clinical Studies, University of Guelph; ^5^Pathobiology, University of Guelph; ^6^Assistant Professor, Clinical Studies, University of Guelph; ^7^Associate Professor, Human Health and Nutrition Science, University of Guelph

**Background:** Hypertrophic cardiomyopathy (HCM) is the most common heart disease in cats and has a largely unknown pathogenesis.

**Hypothesis/Objectives:** The objective was to analyze transcriptomic (mRNA) differences in the hearts of male cats with HCM and healthy male cats of similar age to identify dysregulated genes and pathways.

**Animals:** 7 left ventricle (LV) and 7 left atrium (LA) samples from male cats with HCM (mean age 10 years, range 2–15 years), and 5 LV and 5 LA samples from male adult cats (mean age 7 years, range 2–12 years) without cardiac disease were collected immediately after their death.

**Methods:** RNA sequencing was performed, followed by differentially expressed gene (DEG) analysis using Ingenuity Pathway Analysis (IPA) and Gene Set Enrichment Analysis (GSEA). DEGs were identified with a log2 fold change (log2Fc) of ±0.585 and a false discovery rate (FDR) < 0.05 in IPA. For GSEA, pathways with an FDR < 0.25 were considered significant.

**Results:** The HCM myocardium showed region‐specific pathway dysregulation that might be involved in hypertrophic remodeling. In the HCM LV, angiogenesis, mitochondrial function, and glucose metabolism pathways were inhibited, and inflammation and collagen catabolism were activated. In the HCM LA, autophagy and proteolysis pathways were suppressed, while calcineurin‐NFAT signaling, a pathway involved in hypertrophic growth, and apoptosis pathways were activated.

**Conclusions and Clinical Relevance:** The observed pathways are chamber‐specific and reveal insights into the molecular mechanisms involved in HCM pathogenesis. This may allow the identification and subsequent modulation of signaling cascades involved in disease processes.

## 21 ABSTRACT C21: A Retrospective Study on the Therapeutic Potential of Mesenchymal Stem Cells in Early‐Stage MMVD

### 21.1 **Soyoung Jeung** ^1^, DVM, MS, PhD; Jaegon Ah^2^, DVM, MS; Sung‐Soo Kim^2^, DVM, MS, PhD

#### 21.1.1 ^1^Director of the Department of Internal Medicine and Stem Cell Center, VIP Animal Medical Center; ^2^President, Internal Medicine, VIP Animal Medical Center

**Background:** In veterinary medicine, research on mesenchymal stem cell (MSC) therapy has predominantly focused on advanced myxomatous mitral valve disease (MMVD) cases (stage C or higher) and heart failure. However, studies evaluating its therapeutic potential in early‐stage MMVD patients remain limited, emphasizing the need for further investigation.

**Objectives:** This study investigated the efficacy and safety of MSCs for early‐stage MMVD (stage B1).

**Animals:** Sixteen dogs diagnosed with MMVD ACVIM stage B1 (control: *n* = 10; MSC: *n* = 6).

**Methods:** This retrospective study included a treatment group that received monthly intravenous administrations of allogeneic MSCs derived from gonadal tissue at a dose of 1×10^6^ cells/kg for at least five sessions. Data were analyzed at baseline and at a 1‐year endpoint. Efficacy was assessed through echocardiography, thoracic radiography, NT‐proBNP levels, and the duration from stage B1 diagnosis to stage B2 progression. Safety was evaluated using physical examinations, blood tests, imaging studies, and monitoring for adverse events.

**Results:** After 1 year, the control group showed worsening echocardiographic parameters, while MSC‐treated dogs exhibited minimal changes. Significant differences were observed in left atrial diameter (0.48±2.86 mm vs. 2.97± 2.38 mm) and E‐velocity (−0.08±0.16 m/s vs. 0.26±0.30 m/s) between the MSC and control groups (*p* < 0.05). Additionally, the median duration to MMVD stage B2 progression was 1467 days in the MSC group compared to 730 days in the control group (*p* < 0.05).

**Conclusions and Clinical Importance:** MSC therapy significantly delayed MMVD stage B1 progression, enabling dogs to prolong disease duration without requiring cardiac medication.

**Figure:**
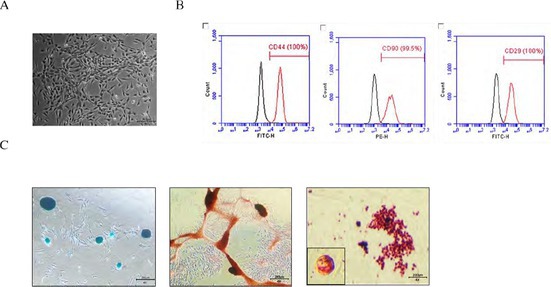
**Figure 1. MSC preparation Characterization and differentiation of canine gonadal tissue‐derived MSCs**. The morphology of canine gonadal tissue‐derived MSCs under light microscopy, where confluent cells exhibit the typical spindle‐like shape characteristic of MSCs with a scale bar representing 200 μm **(A)**. The expression of surface markers of canine gonadal tissue‐derived MSCs is depicted, with the left, middle, and right panels corresponding to the expressions of CD44, CD90, and CD29, respectively, as measured by flow cytometry **(B)**. The chondrogenic, osteogenic, and adipogenic differentiation of canine gonadal tissue‐derived MSCs, with the left panel showing chondrocytes differentiated from MSCs stained with alcian blue, the middle panel displaying osteoblasts originated from MSCs stained with alizarin red S, and the right panel exhibiting adipocytes differentiated from MSCs stained with Oil red O **(C)**.

**Figure:**
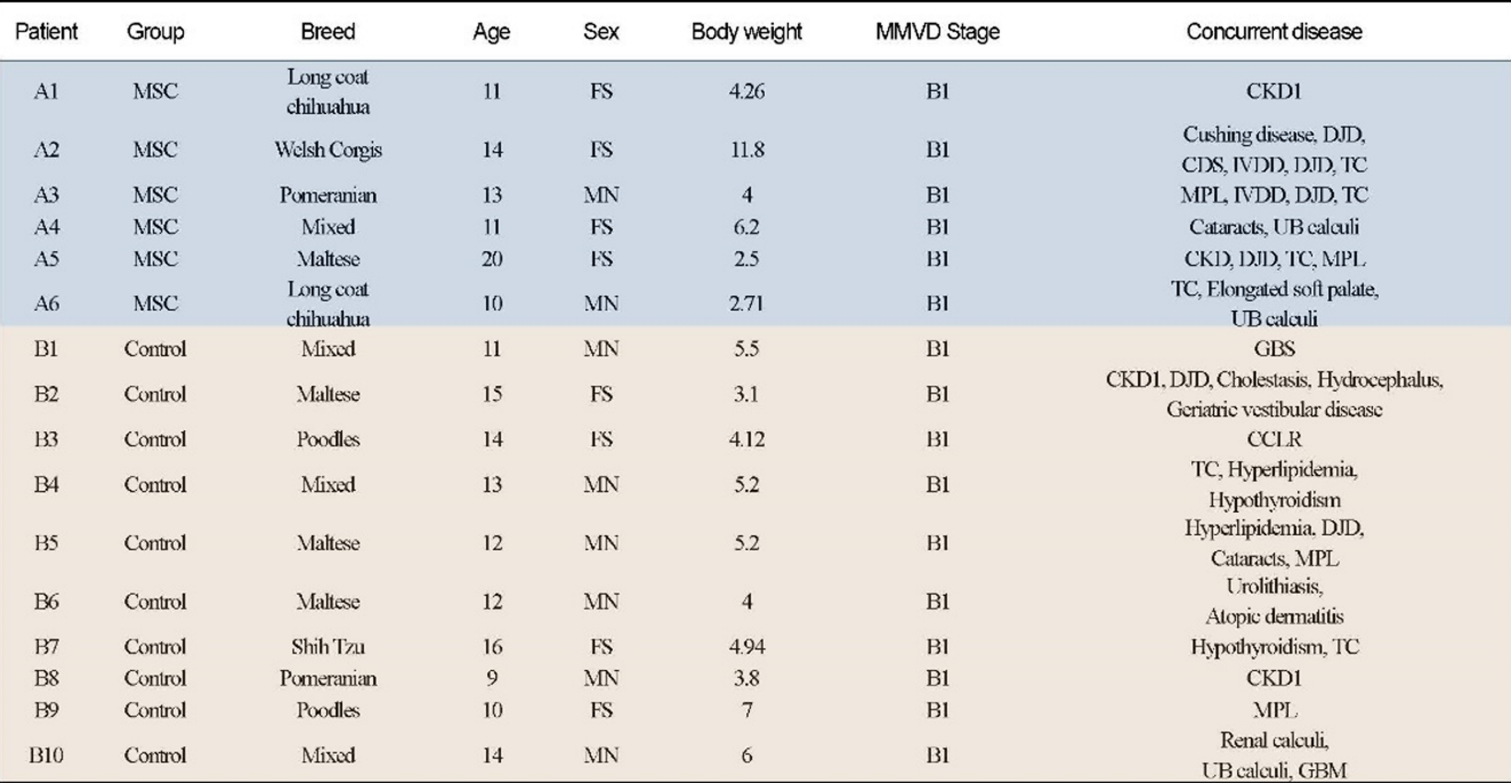
**Table 1. Study population**. Patients’ information.

**Figure:**
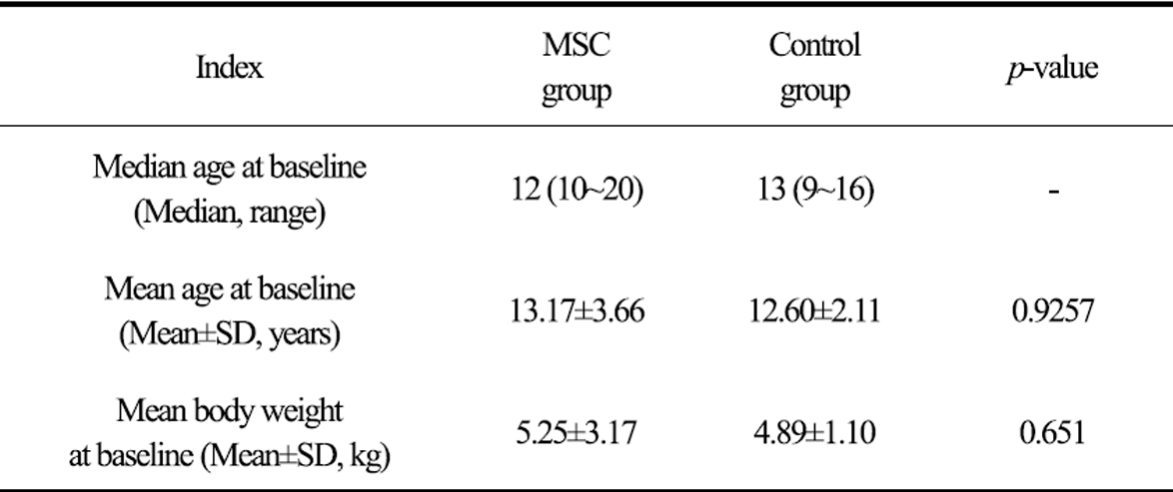
**Table 2. Study population**. Age and body weight at baseline.

**Figure:**
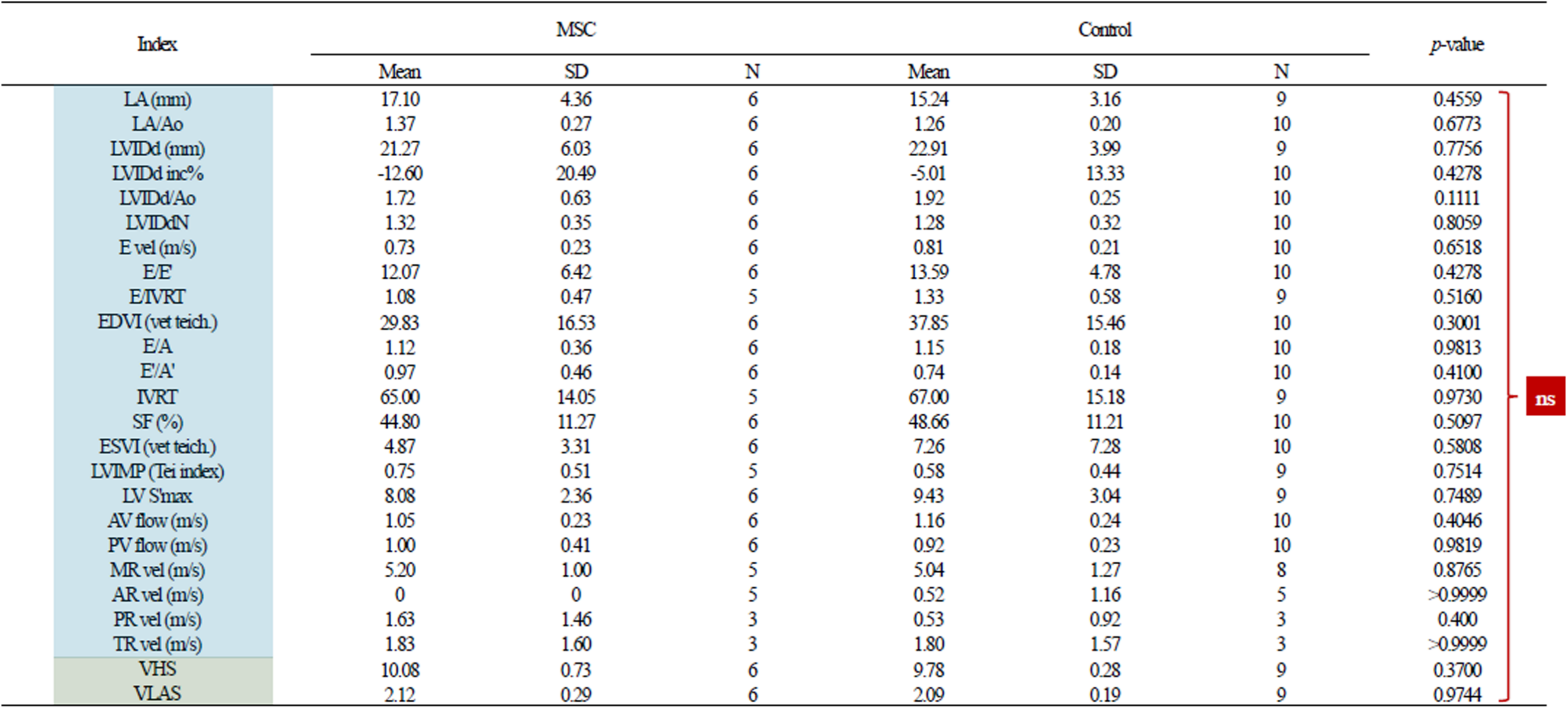
**Table 3. Therapeutic potential of MSC therapy in early‐stage MMVD**. Comparison imaging parameters changes between **the MSCs and control groups at baseline**.

**Figure:**
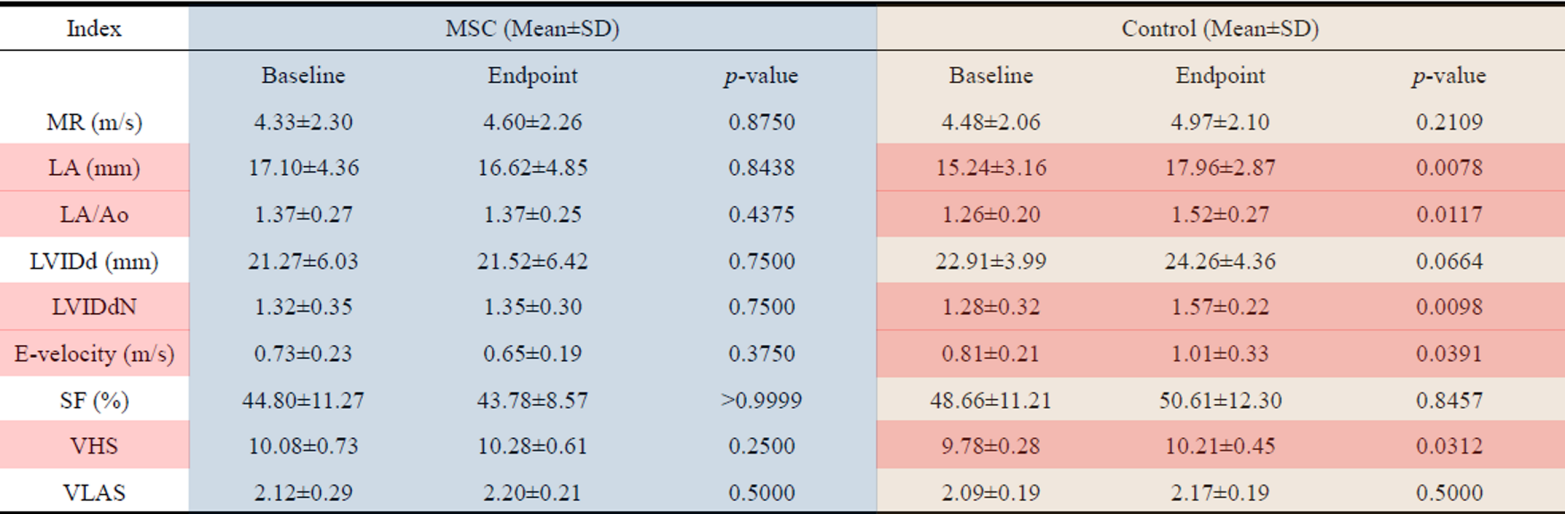
**Table 4. Therapeutic potential of MSC therapy in early‐stage MMVD**. Changes in **imaging parameters** between endpoint and baseline **in respective groups**.

**Figure:**
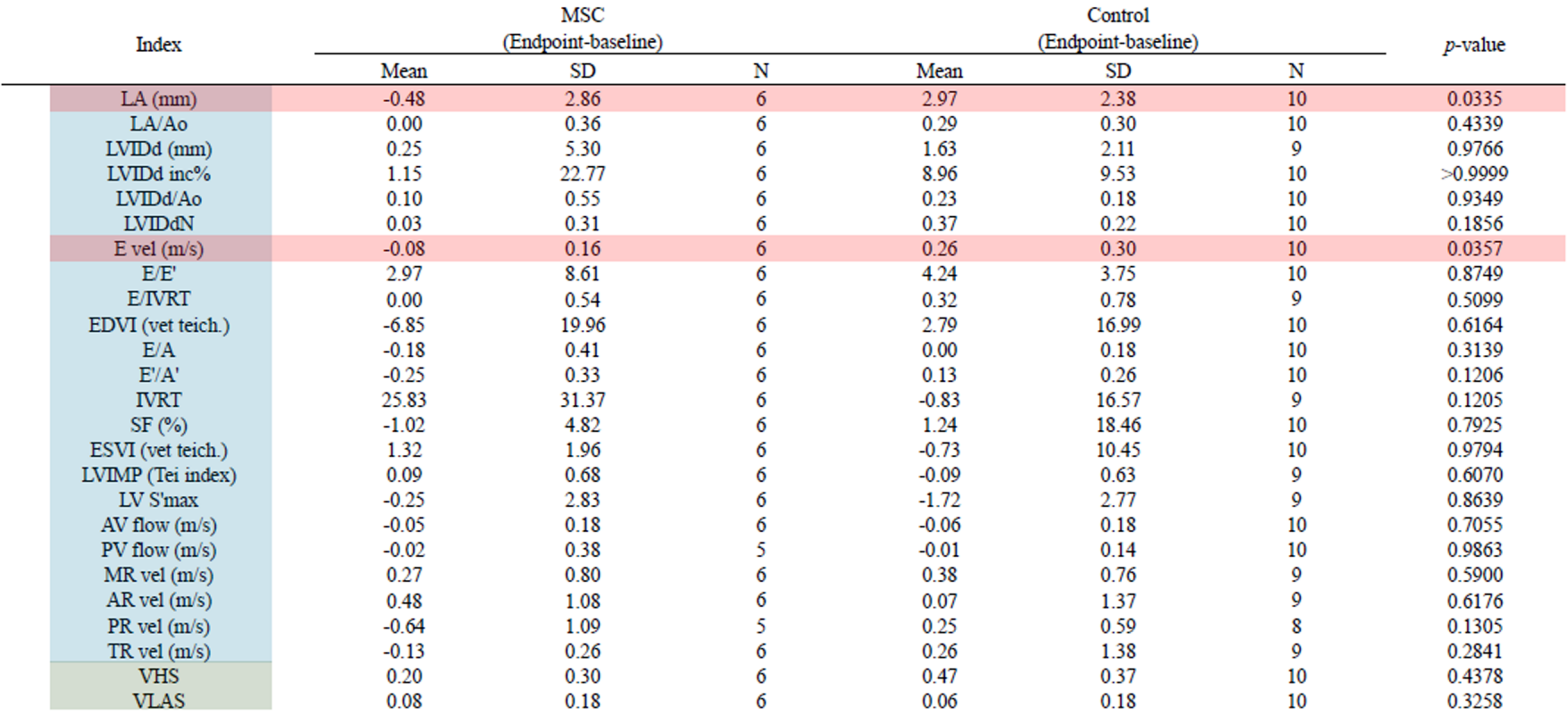
**Table 5. Therapeutic potential of MSC therapy in early‐stage MMVD**. Comparisons imaging parameters changes between endpoint and baseline **in the MSCs and control groups**.

**Figure:**
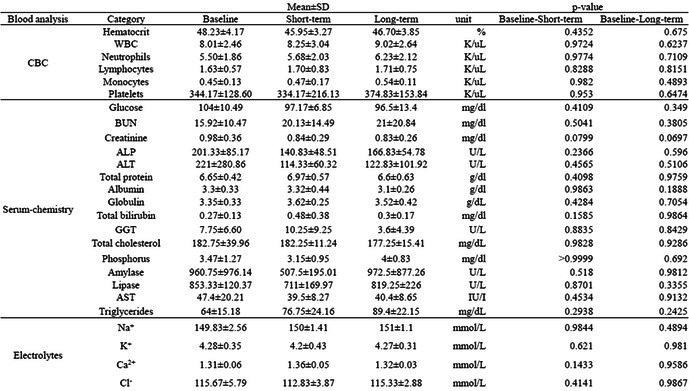
**Table 6. Safety assessment of MSC therapy in early‐stage MMVD**. Blood analysis in the **MSC group** during the evaluation period.

**Figure:**
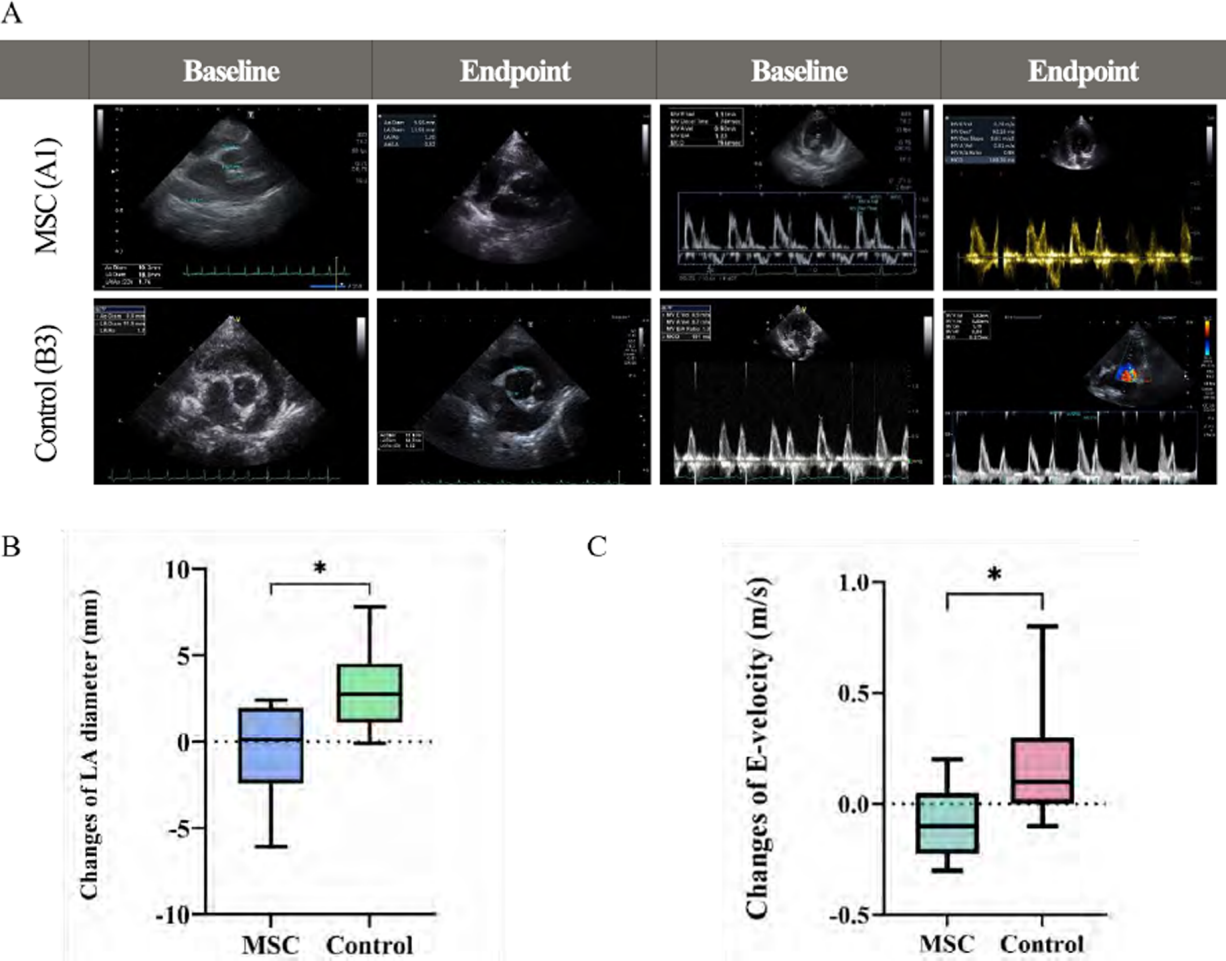
**Figure 2. Therapeutic potential of MSC therapy in early‐stage MMVD** **Comparative analysis of echocardiographic changes in canine MSC and control groups**. The comparison of changes in echocardiography between endpoint and baseline in the MSC therapy and control groups **(A)**. Patient A1 demonstrated improvement from 18 mm to 11.9 mm in LA diameter before (baseline) and after (endpoint) MSC therapy, respectively, with the E‐peak decreasing from 1.1 m/s to 0.8 m/s post‐treatment. In contrast, patient B3, part of the control group, exhibited an increase in LA diameter from 11.9 mm to 14.7 mm after one year, with the E‐velocity rising from 0.9 m/s to 1 m/s. The changes in left atrium diameter between endpoint and baseline after canine gonadal tissue‐derived MSC therapy, showing differences of 0.48±2.86 mm in the MSC group and 2.97±2.38 mm in the control group, indicating a statistically significant difference (*p* < 0.05) (B). The changes in E‐velocity between endpoint and baseline after canine gonadal tissue‐derived MSC therapy, with the E‐velocity changes of −0.08±0.16 m/s in the MSC group and 0.26±0.30 m/s in the control group, showing a statistically significant difference between them (*p* < 0.05) (C).

**Figure:**
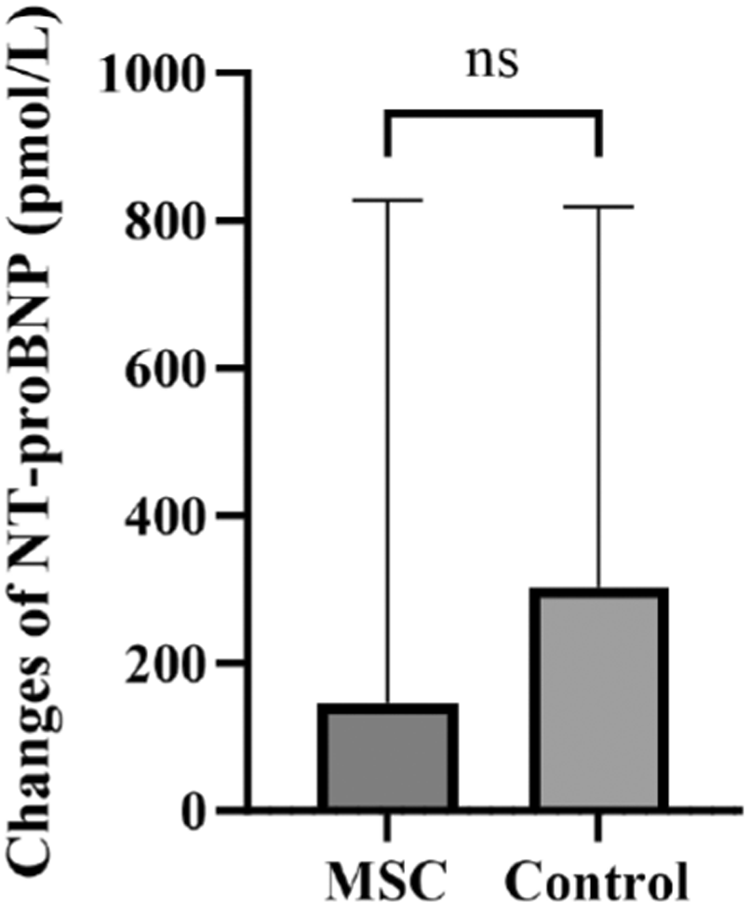
**Figure 3. Therapeutic potential of MSC therapy in early‐stage MMVD**. **The changes in NT‐proBNP between endpoint and baseline after canine gonadal tissue‐derived MSC therapy**. The mean changes in serum NT‐proBNP were 146.80±608.72 pmol/L and 304.14±476.75 pmol/L in the MSC therapy and control groups, respectively, with no statistically significant difference between them.

**Figure:**
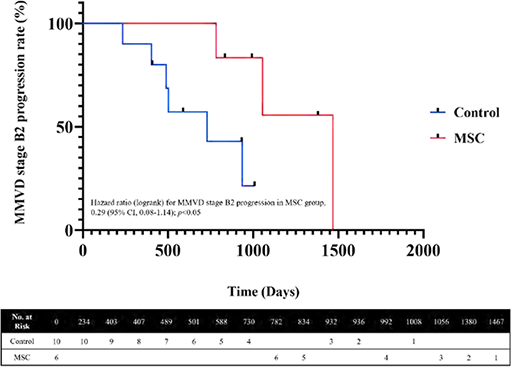
**Figure 4. Therapeutic potential of MSC therapy in early‐stage MMVD**. **Kaplan‐Meier analysis of MMVD progression from B1 to B2 after canine gonadal‐derived MSC therapy**. The median progression duration was 730 days for the control group and 1467 days for the MSC therapy group. The significance level for MMVD stage progression analysis (*p* < 0.05).

**Figure:**
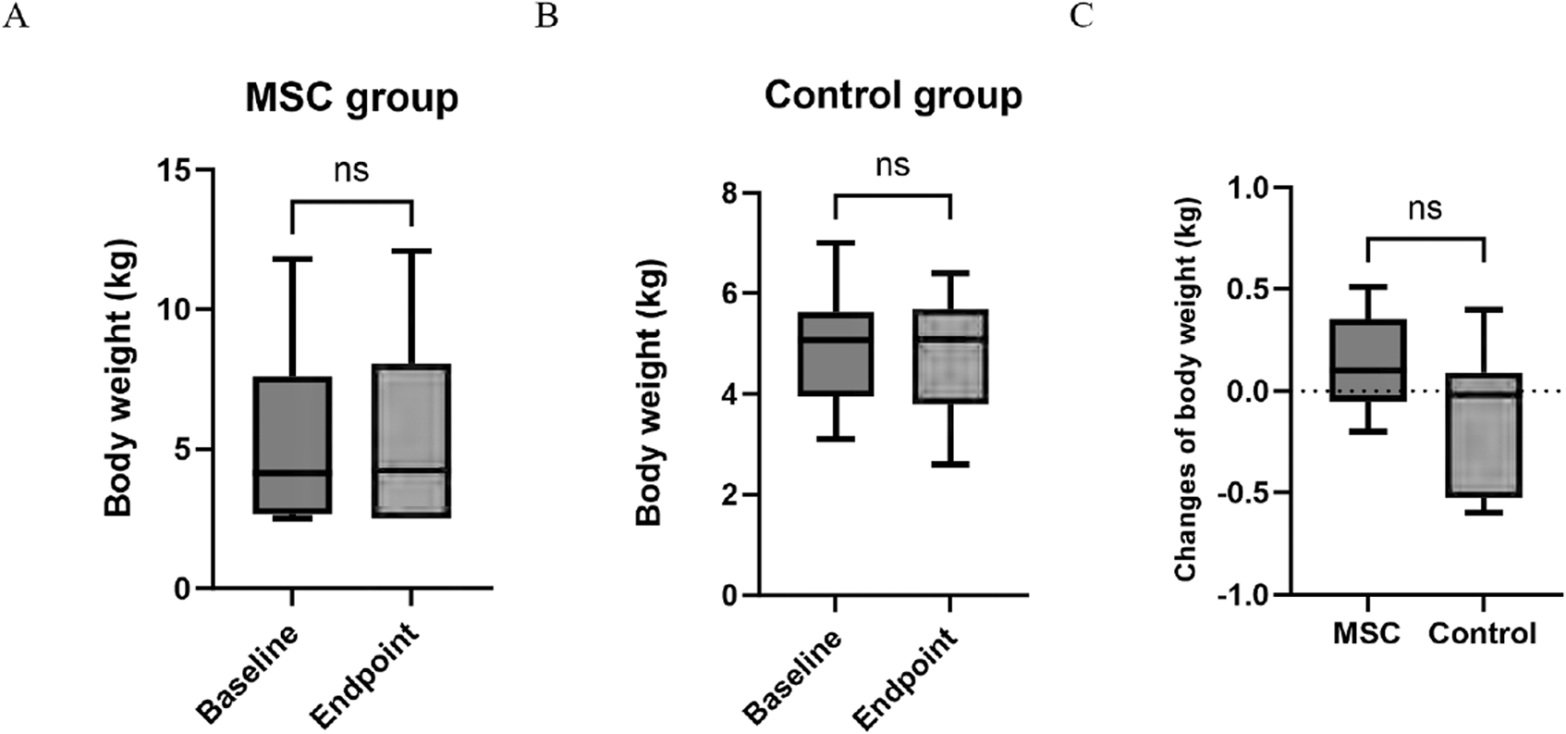
**Figure 5. Safety assessment of MSC therapy in early‐stage MMVD**. **Evaluation of MSC safety in canine patients with one‐year weight changes**. In both the MSC therapy and control groups, weight changes over the 1‐year period were minimal, with the MSC group showing a slight increase (A) and the control group a slight decrease (B). These changes were not statistically significant within each group (A, B) or between the groups (C).

## 22 ABSTRACT C22: Unraveling the Genetics of Feline Hypertrophic Cardiomyopathy: A Multiomics Study

### 22.1 **Joanna Kaplan** ^1^, PhD, DACVIM (Cardiology); Samantha Harris^2^, PhD; Katherine Meurs^3^, DVM, PhD, DACVIM (Cardiology); Victor Rivas^3^, PhD; Joshua Stern^3^, DVM, PhD, DACVIM (Cardiology); Michael Vandewege^3^, PhD; Jalena Wouters^4^

#### 22.1.1 ^1^Assistant Professor of Cardiology, School of Veterinary Medicine, University of California–Davis; ^2^College of Medicine–Tucson, University of Arizona; ^3^College of Veterinary Medicine, North Carolina State University; ^4^School of Veterinary Medicine, University of California–Davis

**Background:** The genetics of feline hypertrophic cardiomyopathy are poorly understood and limited genetic discoveries remain breed or family specific.

**Hypothesis/Objectives:** We aimed to identify novel causative or disease modifying variants in a large cohort of cats reflective of the general cat population. In a second cohort, we sought to characterize transcriptomic differences between HCM‐affected cats and healthy controls.

**Animals:** Cohort1 consisted of 109 HCM‐affected and 29 control cats ≥ 10 years old. Cohort2 consisted of 27 HCM‐affected and 15 control cats euthanized for cardiac or non‐cardiac‐related causes, respectively.

**Methods:** DNA was isolated from blood samples and submitted for paired‐end WGS at ~30× coverage (cohort1). Standard pipelines were employed for variant calling across sequenced cats. In cohort2, immediately flash‐frozen left ventricular (LV), interventricular septal (IVS), and left atrial (LA) tissues were submitted for stranded mature RNA sequencing at 50 million reads/sample.

**Results:** No single or combination of variants of high, moderate, or modifying impact were identified in genome‐wide analysis to cause or modify disease severity of HCM. Several rare high and moderate impact variants in genes associated with human HCM were detected in diseased cats. A total of 70, 111, and 57 DEGs were upregulated and 8, 52, and 130 DEGs were downregulated in LVPW, IVS, and LA tissue, respectively, in HCM‐affected cats compared to controls.

**Conclusions:** Similar to humans, the genetic etiology of feline HCM remains unknown in a high proportion of cases. Transcriptomics revealed molecular signatures that may help identify novel HCM biomarkers or drug targets in future investigations.

## 23 ABSTRACT C23: Incidence and Risk Factors of Postoperative New‐Onset Atrial Fibrillation in Dogs Undergoing Mitral Valve Repair

### 23.1 **Kentaro Kurogochi** ^1^, DVM, PhD; Masako Nishiyama^2^, DVM; Shimon Furusato^2^, DVM; Yasuyuki Nii^2^, DVM, PhD; Masami Uechi^2^, DVM, PhD

#### 23.1.1 ^1^Research Fellow, North Carolina State University; ^2^JASMINE Veterinary Cardiovascular Medical Center

**Background:** Postoperative new‐onset atrial fibrillation (POAF) is a potential complication following cardiac surgery. However, information on its occurrence in veterinary practice remains limited.

**Hypothesis/Objectives:** To describe the characteristics of POAF following mitral valve repair (MVR) in dogs and to identify potential preoperative clinical risk factors associated with its occurrence.

**Animals:** Dogs that underwent MVR between January 2020 and December 2023.

**Methods:** This is a retrospective cohort study. Dogs with preoperative atrial fibrillation or those that died intraoperatively were excluded. The duration of POAF was documented, and potential risk factors of POAF were investigated based on preoperative clinical parameters.

**Results:** A total of 1,580 cases were included. POAF was observed in 100 cases, categorized as the POAF group, while the remaining 1,480 cases were classified as non‐POAF group. The duration of POAF lasted less than 24 h in 88 cases (88%), and only three cases (3%) persisted for over one month postoperatively. Possible independent risk factors for POAF included being male (risk ratio [RR], 1.74; 95% confidence interval [CI], 1.12–2.70, *P* = 0.008), body weight (RR, 1.17; 95% CI, 1.04–1.32, *p* < 0.001), and left ventricular end‐diastolic internal diameter normalized to body weight (RR, 1.11; 95% CI, 1.02–1.21, *P* = 0.018).

**Conclusions and Clinical Importance:** POAF occurred in 6.3% of canine MVR patients. Among these dogs, 88% returned to sinus rhythm within 24 h postoperatively. Male, greater body weight, and preoperative left ventricular enlargement may be associated with the development of POAF.

## 24 ABSTRACT C24: Comparative Efficacy of Injectable Moxidectin and Melarsomine Protocols for Canine Heartworm Treatment

### 24.1 **Ta‐Li Lu** ^1^, DVM, DAiCVIM (Cardiology); Clarke Atkins^2^, DVM, DACVIM (Internal Medicine, Cardiology); Chao‐Yi Chang^3^, DVM; Chun‐Ting Chang^3^, DVM; Tzu‐Hsiang Wu^3^, DVM; Yu‐Hsuan Yang^3^, DVM; Tsz‐Yun Bin^4^

#### 24.1.1 ^1^Superintendent, Chuan Animal Hospital; ^2^Jane Lewis Seaks Distinguished Professor, Emeritus, College of Veterinary Medicine, North Carolina State University; ^3^Chuan Animal Hospital; ^4^Technician, Chuan Animal Hospital

**Background:** Canine heartworm (HW) disease requires symptomatic treatment and, ideally, parasite clearance. The American Heartworm Society recommends a three‐dose melarsomine protocol (AHS). An extended‐release injectable moxidectin protocol (MOX) offers a simpler, less expensive, and potentially equally effective alternative. Comparative studies, however, are limited.

**Hypothesis/Objectives:** The MOX protocol is comparable to the AHS‐3 protocol in clearing HW infections.

**Animals:** With owner consent, 24 client‐owned HW antigen‐positive Taiwanese dogs were randomized into two groups of 12.

**Methods:** Prospective, single‐blind, randomized 360‐day trial, comparing MOX (moxidectin 0.5 mg/kg (Proheart 12®, Zoetis), doxycycline, and prednisone) to AHS (three‐dose melarsomine 2.5 mg/kg (Imiticide®, Boehringer Ingelheim), doxycycline, and prednisone). Moxidectin was repeated at 6‐months to HW antigen‐positive MOX dogs. Treatment was considered effective when a dog tested HW antigen‐negative. Data are presented as mean (SD). The statistical comparison of the time to antigen negativity between groups was conducted using the log‐rank test.

**Results:** Eleven dogs (91.67%) in each group achieved antigen‐negativity within 360 days. The mean time to HW‐negativity was 127 days (median 104; range 89–242 days) and 154 days (median 130; range 86–313; *P* > 0.05) in the AHS and MOX groups, respectively. Three MOX dogs, HW‐positive at 180‐days, required a second moxidectin injection and 1 remained antigen‐positive at Day 360. One AHS dog died suddenly on Day 108. Other than this post‐arsenical death, no significant adverse events were noted in either group.

**Conclusions and Clinical Importance:** The MOX protocol is comparable to the AHS‐3 protocol, showing promise as an alternative to arsenical therapy for canine HW.

## 25 ABSTRACT C25: Application of a Novel MicroRNA Platform to Cardiac Disease Diagnoses in Companion Animals

### 25.1 **Eve Hanks** ^1^, BVMS, CertAVP (EM), PhD, MRCVS; Robert Coultous^2^, BVSc, MSc, PhD, AFHEA, MRCVS; Paul Capewell^3^, PhD; Javier Palarea‐Albaladejo^4^, PhD; Joanna Dukes‐McEwan^5^, BVMS (Hons), MVM, PhD, DVC, DECVIM‐CA (Cardiology), FRCVS

#### 25.1.1 ^1^CEO, MI:RNA Ltd; ^2^Chief Scientific Officer, MI:RNA Ltd; ^3^School of Molecular Biosciences, University of Glasgow; ^4^University of Girona; ^5^University of Liverpool

**Background:** Cardiac disease is common and challenging to diagnose in primary care. MicroRNA diagnostics could fill this unmet clinical need.

**Hypothesis/Objectives:** In companion animals, to answer the question: can analysis of a blood sample by a novel microRNA‐based platform, when compared to the current gold standard of echocardiography, effectively distinguish between animals with cardiac disease and healthy controls?

**Animals:** One hundred and fifty‐nine client‐owned animals; 52 cats (34 hypertrophic cardiomyopathy (HCM) and 18 controls) and 107 dogs (36 myxomatous mitral valve disease (MMVD), 30 dilated cardiomyopathy (DCM) and 41 controls).

**Methods:** A prospective case‐control study was performed to obtain blood samples, surplus to clinically indicated testing, from cats with HCM, dogs with DCM or MMVD and healthy cat and dog controls. Cardiac diagnoses were made by a cardiology specialist after a cardiac evaluation including echocardiography. Blood samples were sub‐analysed by diagnostic group for the expression profile of 15 microRNA markers using machine learning algorithms. Model classification accuracy was assessed and sensitivity and specificity for each pathology was reported with 95% confidence intervals.

**Results:** For HCM, diagnostic sensitivity was 0.89 [0.74–0.96] and specificity was 0.77 [0.52–0.92]. For diagnosis of DCM, sensitivity was 0.87 [0.70–0.95] and specificity was 0.59 [0.43–0.72]. For MMVD, diagnostic sensitivity was 0.83 [0.69–0.91] and specificity was 0.92 [0.78–0.97].

**Conclusions and Clinical Importance:** This proof‐of‐concept study demonstrates the capability of a novel microRNA platform to identify companion animals with cardiac disease and its potential for use in primary care.

## 26 ABSTRACT C26: Effect of Moderate to Severe Hypochloremia on Survival in Dogs With Stable Congestive Heart Failure

### 26.1 **Dylan DeProspero** ^1^, DVM; Autumn Harris^2^, DACVIM (SAIM); Penelope Reynolds^3^, PhD; Darcy Adin^4^

#### 26.1.1 ^1^Cardiology Resident, College of Veterinary Medicine, University of Florida; ^2^College of Veterinary Medicine, North Carolina State University; ^3^College of Medicine, University of Florida; ^4^College of Veterinary Medicine, University of Florida

**Background:** Serum chloride concentrations (s[Cl]) can be low in congestive heart failure (CHF). Although likely multifactorial, hypochloremia < 100 mEq/L is associated with poor outcomes in people.

**Hypothesis/Objectives:** We hypothesized that hypochloremia would be associated with poor survival in dogs with CHF.

**Animals:** A total of 292 stable CHF dogs were included (229 degenerative valve disease, 31 dilated cardiomyopathy, 21 congenital heart disease, 6 pulmonary hypertension, and 5 arrhythmogenic cardiomyopathy).

**Methods:** Single institutional retrospective study from 2014–2024. Biochemical variables, medications, and outcome data were extracted from medical records at the first recheck after initial CHF episode. Exclusion criteria were active CHF, absence of s[Cl] on bloodwork, point‐of‐care bloodwork, and clinically important vomiting or diarrhea. Dogs were grouped by s[Cl] < 100 mEq/L. Risk of death associated with s[Cl] < 100 mEq/L, biochemical variables, and medications was evaluated with Cox regression analysis and survival was assessed using test Kaplan‐Meier analysis using log‐rank sum.

**Results:** Median s[Cl] was 106.5 mEq/L (range 85.0–119.0). Serum [Cl] < 100 mEq/L was associated with risk of death (hazard ratio 2.649; CI 1.265–5.311), with significant survival difference between dogs with s[Cl] < 100 mEq/L (115 days) and dogs with s[Cl] ≥ 100 mEq/L (196 days) (*P* = 0.0116). Medications and other biochemical variables were not associated with risk of death or outcome.

**Conclusions and Clinical Importance:** Moderate to severe hypochloremia (s[Cl] < 100 mEq/L) in dogs with stable CHF is associated with shorter survival times compared to s[Cl] ≥ 100 mEq/L. These findings support efforts to increase s[Cl] levels above 100 mEq/L in dogs with CHF.

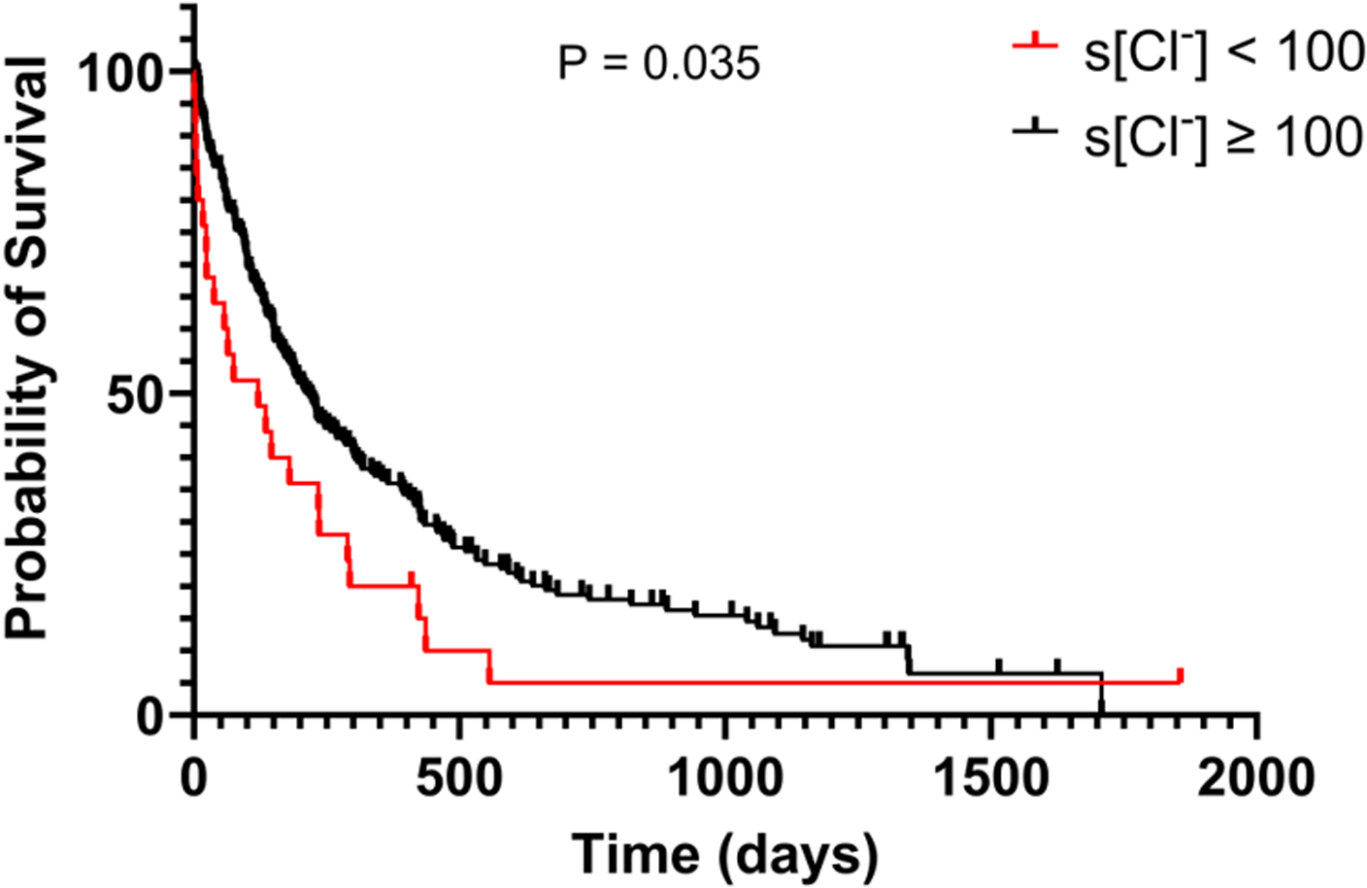

## 27 ABSTRACT C27: Breed Differences in Left Ventricular Early Inflow‐Outflow Index in Dogs With Myxomatous Mitral Valve Disease

### 27.1 **Marco Mazzarella** ^1^, DVM, MScR, MRCVS; Gavin McAulay^2^, BVetMed, DVC, CertVC, CertEM (IntMed), MRCVS; Matthew Butler^3^, BSc, MBChB, PhD; Rebecca Foster^4^, BSc, PhD; Natalie Finch^5^, BVSc, PhD, DECVIM‐CA (Internal Medicine); Jade Ward^6^; Wasutorn Yangwanitset^7^; Metaxia Tsakiroglou^7^; Melanie Hezzell^8^, MA, VetMB, PhD, CertVDI, CertVC, PhD, MRCVS, DACVIM (Cardiology)

#### 27.1.1 ^1^PhD Student, University of Bristol; ^2^Brighton Veterinary Referrals; ^3^Consultant Senior Lecturer, Renal Group, University of Bristol; ^4^Associate Professor of Microvascular Medicine, Renal Group, University of Bristol; ^5^Head of Renal Medicine, Bristol Veterinary School, University of Bristol; ^6^Research Technician, Bristol Veterinary School, University of Bristol; ^7^PhD Student, Bristol Veterinary School, University of Bristol; ^8^Professor of Veterinary Cardiology, Bristol Veterinary School, University of Bristol

**Background:** Myxomatous mitral valve disease (MMVD) is the commonest heart disease in dogs. The left ventricular early inflow‐outflow index (LVEIO) is a Doppler estimate of mitral regurgitation severity, validated in human patients. The calculation includes aortic velocity time integral (VTI) measurements, which are affected by cardiac geometry. Geometric differences exist in the hearts of Cavalier King Charles spaniels (CKCS) compared to other breeds, which might affect LVEIO.

**Hypothesis/Objective:** In dogs with stage B1 MMVD, LVEIO is higher in CKCS compared to non‐CKCS.

**Animals:** 34 privately owned dogs with stage B1 MMVD (21 CKCS and 13 non‐CKCS) enrolled in a longitudinal study of MMVD.

**Methods:** Retrospective cross‐sectional study. Stage B1 MMVD was diagnosed as per ACVIM consensus statement. LVEIO was calculated as the ratio between mitral inflow E wave maximum velocity and aortic VTI. Data distribution was tested using the Shapiro‐Wilk test. Correlations were tested using Pearson's or Spearman's *ρ* test. Groups (CKCS vs non‐CKCS) were compared using unpaired Student's *t*‐test or Mann‐Whitney tests as appropriate. Data are reported as median (minimum–maximum).

**Results:** E wave velocity (*p* = 0.69) and aortic VTI (*p* = 0.055) were not different between groups (Figure 1). There was no correlation between LVEIO and body weight in CKCS (*p* = 0.35, *ρ*=−0.22) or non‐CKCS (*p* = 0.49, *ρ*=−0.21). LVEIO was significantly higher in CKCS (5.42 (3.06–7.98)) vs non‐CKCS dogs (4.67 (3.04–11.45); *p* = 0.029).

**Conclusions and Clinical Importance:** LVEIO can be easily obtained and might provide further information about MMVD severity in dogs. However, a breed‐specific range for CKCS should be considered for more accurate assessment.

**Figure:**
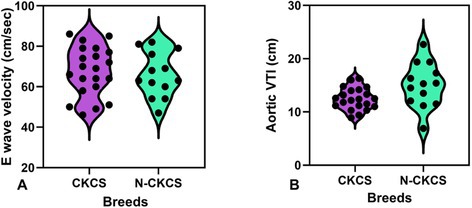
**Figure 1** Violin plot of E wave velocity (A) and aortic velocity time integral (VTI) (B) for Cavalier King Charles Spaniels (CKCS) and other breeds (N‐CKCS). Means E wave velocities are 67.67 cm/sec (±12.12) and 66 cm/sec (±11.11), respectively. Means aortic VTI are 12.47 cm (±2.03) and 15.04 cm (±4.17), respectively. Each dot represents an individual dog.

## 28 ABSTRACT C28: Agreement of Resting Respiratory Rate Measurements and Those Obtained by the Maven Collar in Dogs

### 28.1 **Katherine Murphy** ^1^, DVM; Darcy Adin^2^; Carolina Domingues^3^; Eduardo Benjamin^4^; Rebecca Leigh^5^

#### 28.1.1 ^1^Small Animal Rotating Intern, College of Veterinary Medicine, University of Florida; ^2^Clinical Professor of Cardiology, Chief Medical Officer and Assistant Dean of Small Animal Hospitals, College of Veterinary Medicine, University of Florida; ^3^Research and Partnerships Specialist, Maven Pet; ^4^Clinical Assistant Professor, Cardiology, College of Veterinary Medicine, University of Florida; ^5^Veterinary Student, College of Veterinary Medicine, University of Florida

**Background:** Resting respiratory rate (RRR) monitoring is essential in dogs with advanced cardiac disease to monitor for control of congestive heart failure. The Maven Pet collar system offers a feature measuring RRR in pets.

**Hypothesis/Objective:** To describe the agreement between Maven Pet Smart Collar‐obtained and manually counted RRR measurements in dogs.

**Animals:** 18 apparently healthy, student and staff‐owned dogs.

**Methods:** Dogs were fitted for a Maven Pet Smart Collar. Owners were trained to video record their dogs at home to document the RRR for later analysis. Owners were asked to collect an aggregate of at least 28 one‐minute videos. Videos were collected after the dogs were resting for at least 15 min. Videos were used for analysis if they were within one minute from a collar‐obtained RRR. Bland‐Altman analysis was used to assess agreement between Maven Pet Smart Collar‐obtained RRR and RRR measured by the investigators from video recordings.

**Results:** Data was collected from 18 dogs (*n* = 120 instances). Relative to manually counted RRR, bias for the Maven Pet Smart Collar system was −0.75 breaths/minute (95% limits of agreement −8.28 to 6.79 breaths/min).

**Conclusions and Clinical Importance:** The Maven Pet Smart Collar system closely agrees with manually counted RRR, with relatively narrow limits of agreement. The Maven Pet Smart Collar could be considered for monitoring RRR in dogs, especially in dogs with poorly compliant owners because it is automated. Clinicians will need to consider the limits of agreement in interpretation.

**Figure:**
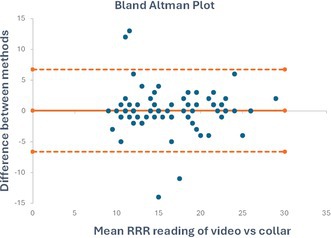
**Figure 1** Bland‐Altman Plot illustrating the difference in measurements between the manually counted resting respiratory rate and the Maven Pet collar system. The solid orange line illustrates the mean bias and the dotted orange lines represent the upper and lower 95% limits of agreement.

## 29 ABSTRACT C29: Critically Appraised Topic: What Is the Clinical Potential for MicroRNAs As Cardiac Biomarkers in Dogs?

### 29.1 **Eve Hanks** ^1^, BVMS, CertAVP (EM), PhD, MRCVS; Robert Coultous^2^, BVSc, MSc, PhD, AFHEA, MRCVS; Alexandra Raftery^3^, MVM, MA, VetMB, DECEIM, MRCVS

#### 29.1.1 ^1^CEO, MI:RNA Ltd; ^2^Chief Scientific Officer, MI:RNA Ltd; ^3^University of Glasgow

**Background:** Cardiac disease is common in dogs but diagnosis and monitoring require specialist imaging. Cardiac biomarkers can provide key information in the primary care setting. MicroRNAs are novel biomarkers with application to cardiac disease.

**Objectives/Hypothesis:** Does measurement of microRNA biomarkers show potential for diagnosis of myocardial disease with respect to (1) presence and (2) severity when compared with echocardiography?

**Methods:** This was a critically appraised topic using PICO methodology. A search strategy was developed (PubMed; January 2007–December 2024). Studies were included describing microRNAs associated with naturally occurring cardiac disease in dogs. Exclusion criteria were defined (no gold standard comparison, secondary source, case report). Manuscripts were reviewed and data extracted (condition, population, methods and results). Study quality was assessed and recommendations made (GRADE). Studies were sub‐categorised by pathology.

**Results:** From 76 studies retrieved, 61 were excluded (22 not relevant, 35 experimental, 4 review articles). Fifteen met the inclusion criteria (14 case‐control; one longitudinal case series). Six studies evaluated ACVIM staged myxomatous mitral valve disease (MMVD) with differences in microRNA expression between A and B and B and C/D disease. Heterogeneity in microRNAs prohibited evidence synthesis. Remaining studies demonstrated the application of microRNA profiling to a range of cardiac pathologies (unstaged MMVD, pulmonic stenosis, dilated cardiomyopathy).

**Conclusions and Clinical Importance:** There is strong evidence in favour of the potential for microRNAs being a useful future addition to canine cardiac diagnostics particularly for MMVD. Further work determining microRNA panels with optimal discriminatory power is needed.

## 30 ABSTRACT C30: Hemoglobin, Albumin, Lymphocyte, and Platelet Score as a Predictor of Short‐Term Mortality in Canine CHF

### 30.1 **Ha Suk Nam** ^1^, DVM; Jayeon Park^2^; Yeon Chae^3^, PhD; Hakhyun Kim^4^; Byeong‐Teck Kang^4^; Taesik Yun^4^

#### 30.1.1 ^1^Veterinary Student, Laboratory of Veterinary Internal Medicine, College of Veterinary Medicine, Chungbuk National University; ^2^Laboratory of Veterinary Internal Medicine, College of Veterinary Medicine; ^3^Clinical Professor, Laboratory of Veterinary Internal Medicine, College of Veterinary Medicine; ^4^Professor, Laboratory of Veterinary Internal Medicine, College of Veterinary Medicine

**Background:** Myxomatous mitral valve disease (MMVD) is the most common heart disease in dogs. Reliable prognostic indicators for predicting survival time in dogs with congestive heart failure (CHF) remain limited. Recently, the hemoglobin, albumin, lymphocyte, and platelet (HALP) score has received attention as a novel prognostic indicator for short‐term mortality in humans, which has not been studied in veterinary medicine.

**Hypothesis/Objective:** This study aimed to evaluate the utility of the HALP score as a prognostic indicator for short‐term mortality in dogs with CHF due to MMVD.

**Animals:** Fifty‐four dogs diagnosed with CHF secondary to MMVD.

**Methods:** The HALP score was calculated using the following formula: hemoglobin (g/L)×albumin (g/L)×lymphocytes (/L)/platelets (/L). This study evaluated survival outcomes at six, nine, and twelve months.

**Results:** The HALP score was significantly higher in the survival group than in the non‐survival group at both six‐month and nine‐month mortality (*p* < 0.01). Receiver operating characteristic curve analysis revealed that the HALP score had good diagnostic accuracy, with area under the curve values exceeding 0.7 for predicting both six‐month and nine‐month mortality. An optimal cut‐off HALP score of 11.13 showed a sensitivity of 44.44% and a specificity of 94.44% for predicting six‐month mortality. Kaplan‐Meier survival analysis demonstrated that dogs with higher HALP scores had significantly longer survival times than those with lower scores.

**Conclusion and Clinical Importance:** The HALP score could be a valuable prognostic indicator for predicting short‐term mortality in dogs diagnosed with CHF due to MMVD.

## 31 ABSTRACT C31: Asymptomatic Arrhythmias in Aged, Apparently Healthy Dogs Screened for a Large, Multi‐Center Clinical Trial

### 31.1 **Taylor Reiter** ^1^, DVM; Elizabeth Malcolm^2^; Aliya McCullough^3^; Janice O’Brien^3^; Audrey Ruple^3^; Jake Ryave^2,4^; Kate Creevy^2^

#### 31.1.1 ^1^Clinical Research Intern, Dog Aging Project; ^2^Texas A&M University; ^3^Virginia‐Maryland College of Veterinary Medicine; ^4^Dog Aging Project

**Background:** Clinically relevant arrhythmias can occur in dogs without overt clinical signs. Arrhythmias have been previously established as important exclusionary criteria from the Test of Rapamycin in Aging Dogs (TRIAD).

**Hypothesis/Objective:** Describe the nature and prevalence of clinically significant arrhythmias and concurrent abnormalities at screening within the TRIAD target population.

**Animals:** Reportedly healthy dogs ≥ 7 years of age and 20–55 kg (*n* = 247) assessed for eligibility within the multi‐center TRIAD clinical trial.

**Methods:** Cross‐sectional assessment of dogs qualified for in‐person screening examination after medical record review. Examination included systolic arterial blood pressure, physical examination, five‐minute electrocardiogram, complete echocardiogram, CBC, chemistry, urinalysis, heartworm serology for all dogs with Chagas IFA testing for dogs in Texas. Holter monitoring was introduced to evaluate ventricular ectopy (VE) identified during screening that did not warrant antiarrhythmic therapy.

**Results:** 38/235 (16%) screened dogs (18 F/S; 20 M/C) had VE and 24/38 (63%) had a Holter performed. Of those 24 dogs, 8 remained eligible and 16 were excluded from enrollment in the TRIAD study based on the complexity of arrhythmias on Holter. Exclusionary arrhythmias found on Holter included ventricular tachycardia (6/16), ventricular couplets/triplets (3/16), and ≥ 50 VPCs in 24 hrs (7/16). Concurrent findings in some dogs excluded with arrhythmias included neoplasia, cardiac disease, positive Chagas titer, and biochemical changes.

**Conclusions and Clinical Importance:** Clinically significant VE is common within this age group of medium‐to‐large dogs, even when seemingly healthy upon records review. When VE is encountered upon physical examination, Holter monitoring can reveal increased arrhythmia severity.

## 32 ABSTRACT C32: Evaluation of Dose‐Dependent Effects of Dapagliflozin on Urinary Glucose and Plasma Ketone Bodies in Dogs

### 32.1 **Mutsuki Umezawa**, DVM; Kensuke Orito; Ryo Yoshimoto; Raiki Shinozaki; Haruka Yoshimura; Yoko Fujii

#### 32.1.1 Azabu University

**Background:** Dapagliflozin is a sodium‐glucose cotransporter‐2 inhibitor used in the treatment of human patients with heart failure. Despite its potential applications in veterinary medicine, no studies have evaluated its pharmacodynamics in dogs.

**Hypothesis/Objectives:** To evaluate the dose‐dependent effects of single dosing of dapagliflozin on pharmacodynamics, diuresis and ketogenesis in dogs.

**Animals:** Five clinically healthy dogs.

**Methods:** Each dog received all treatments (single oral dose of dapagliflozin at 0.1, 0.3, or 1.0 mg/kg, and placebo) in a randomized sequence, with a 14‐day washout period between each treatment. Urine and blood samples were collected at predefined time points (0, 1, 2, 3, 4, 5, 6, 9, 12, and 24 h post‐administration) to evaluate urine volume; plasma glucose, ketone bodies, and electrolytes (sodium, chloride, and potassium); and urine glucose and electrolytes.

**Results:** 24‐h urinary glucose excretion was significantly increased in the 0.3 mg/kg group compared to the 0.1 mg/kg group (*p* = 0.02). However, no significant difference in 24‐h urinary glucose excretion was observed between 0.3 mg/kg and 1.0 mg/kg groups. Plasma ketone body concentration was significantly higher in the 1.0 mg/kg group compared to the placebo group at 4, 5, 6, 12, and 24 h post‐administration (*P* < 0.05). No significant differences were observed among the four groups (placebo, 0.1 mg/kg, 0.3 mg/kg, and 1.0 mg/kg) in urine volume, plasma glucose and electrolytes, and urinary electrolytes.

**Conclusions and Clinical Importance:** Single oral dose dapagliflozin increased urinary glucose excretion with a dose‐dependent increase observed up to 0.3 mg/kg, and induced ketogenesis in dogs.

## 33 ABSTRACT C33: Association of MicroRNAs With Sudden Death in Boxer Dogs With Arrhythmogenic Right Ventricular Cardiomyopathy

### 33.1 **Laura Machado Ribas** ^1^, DVM; Jasmine Aggarwal^2^; Kerstin Muner^3^, DVM, MSc; Andrea Pires dos Santos^4^, DVM, MSc, PhD, DACVP (Clinical); Luis Dos Santos^5^, DVM, MSc, PhD, DACVIM (Cardiology)

#### 33.1.1 ^1^Graduate Student, Purdue University; ^2^Veterinary Student, Purdue University; ^3^Graduate Student, Comparative Pathobiology, Purdue University; ^4^Associate Professor, Comparative Pathobiology, Purdue University; ^5^Assistant Professor, Veterinary Clinical Sciences, Purdue University

**Background:** Arrhythmogenic right ventricular cardiomyopathy (ARVC) is a common condition in Boxer dogs associated with sudden death. Clinical diagnosis relies on the detection of ventricular arrhythmias but does not predict disease severity. Circulating microRNAs have been identified as predictive factors of recurrent arrhythmias in people, but studies in dogs are still lacking.

**Hypothesis:** MicroRNAs are differentially expressed (DE) in Boxers that manifested sudden death compared to those that died from other causes.

**Animals:** Cardiac samples from Boxer dogs that experienced sudden death due to ARVC (SCD; *n* = 4), non‐cardiac‐related death (NCD; *n* = 4), and healthy mixed‐breed dogs (CTRL; *n* = 4).

**Methods:** Cross‐sectional study. Total RNA was extracted using a commercial kit and subjected to small RNA sequencing and qPCR validation. Fold regulation > 1.5 in either direction and *p* < 0.05 were considered for all comparisons.

**Results:** A total of 88 miRNAs with the highest fold changes between groups were selected for validation using customized qPCR panels. Upon validation, in SCD vs CTRL, 13 miRNAs were DE, while nine were DE in NCD vs CTRL. On qPCR, miR‐885 and miR‐31 were downregulated in SCD vs NCD, though in sequencing only miR‐208a was downregulated. Pathways regulated by the DE miRNAs include the MAPK, FoxO, TNF, and PI3K‐Akt.

**Conclusions and Clinical Importance:** MicroRNAs may help stratify Boxers with a higher risk of sudden death. Some of the pathways linked to these microRNAs have been described in human ARVC and may provide insights into the pathophysiology and assist in developing new targeted therapies.

**Figure:**
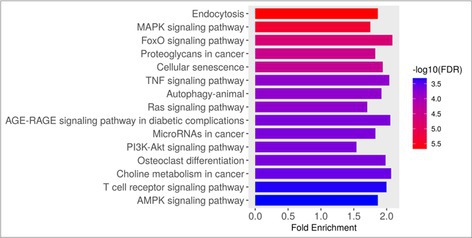
KEGG pathway enrichment analysis based on target genes of differentially expressed miRNAs in ARVC. The target genes were predicted through miRDB Target Mining, and the list of the genes was then submitted to shinyGO for gene‐set enrichment. The *y*‐axis lists the enriched pathways, while the *x*‐axis represents fold enrichment values.

## 34 ABSTRACT C34: Etiologies and Outcomes of Pericardial Effusion in Small‐Breed Dogs: Emphasis on Cardiogenic Causes

### 34.1 **Jaechun Cho** ^1^, DVM, MS; Chul Park^2^; Mihyun Choi^3^, PhD; Eunji Lee^3^, MS; Jayeon Ryu^3^; Doyun Kim^3^, PhD; Minsuk Kim^4^, PhD; Chuleui Park^3^

#### 34.1.1 ^1^PhD Student, Bon Animal Medical Center/Jeonbuk National University; ^2^Professor, Jeonbuk National University; ^3^Bon Animal Hospital; 4Jeonbuk National University

**Background:** Pericardial effusion (PE) arises from various etiologies. Previous studies have predominantly focused on large‐breed dogs, with limited research on PE in small breeds.

**Hypothesis/Objectives:** This study aimed to investigate the causes of PE in small‐breed dogs and compare clinical characteristics, echocardiographic findings, and survival times across different etiologies.

**Animals:** Thirty‐two client‐owned small‐breed dogs (≤ 10 kg) diagnosed with PE, drawn from a referral hospital population between 2021 and 2024, were retrospectively reviewed.

**Methods:** This retrospective case series analyzed clinical, echocardiographic, and survival data. Dogs were categorized into three groups based on etiology: cardiogenic, neoplastic, and idiopathic. Kaplan‐Meier survival analysis compared survival times among the three groups. Additionally, clinical and echocardiographic parameters were compared between cardiogenic and non‐cardiogenic groups.

**Results:** Cardiogenic PE accounted for 37.5% (12/32) of cases, neoplastic 46.9% (15/32), and idiopathic 15.6% (5/32). Among cardiogenic cases, 8 dogs were diagnosed with left atrial rupture and 4 with right‐sided congestive heart failure. All cardiogenic cases survived to hospital discharge, with the shortest post‐discharge survival time being 6 days. Mean survival times were 31.5 days (cardiogenic), 120 days (neoplastic), and 210 days (idiopathic), with significant differences among groups (log‐rank *P* value < 0.05). Body weight, sex, age, blood pressure, lactate levels, syncope episodes, vomiting episodes, and echocardiographic findings were compared between cardiogenic and non‐cardiogenic cases. Among echocardiographic findings, the LA/Ao ratio and LVIDDN showed statistically significant differences between the two groups.

**Conclusions and Clinical Importance:** Cardiogenic PE represents a notable proportion of PE cases in small‐breed dogs, including both left atrial rupture and right‐sided congestive heart failure. While all cardiogenic cases survived hospitalization, their overall survival times remained significantly shorter compared to other groups. These findings highlight the need for echocardiographic evaluation to better consider cardiogenic causes and guide treatment in small‐breed dogs.

**Figure:**
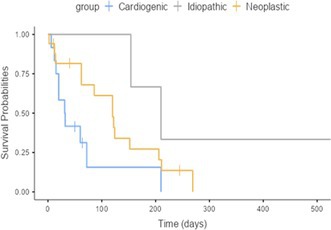
**Figure 1** Kaplan‐Meier curve showing the survival differences among cardiogenic, neoplastic, and idiopathic pericardial effusion groups. Vertical bars indicate censored data (*P* < 0.05).

**Figure:**
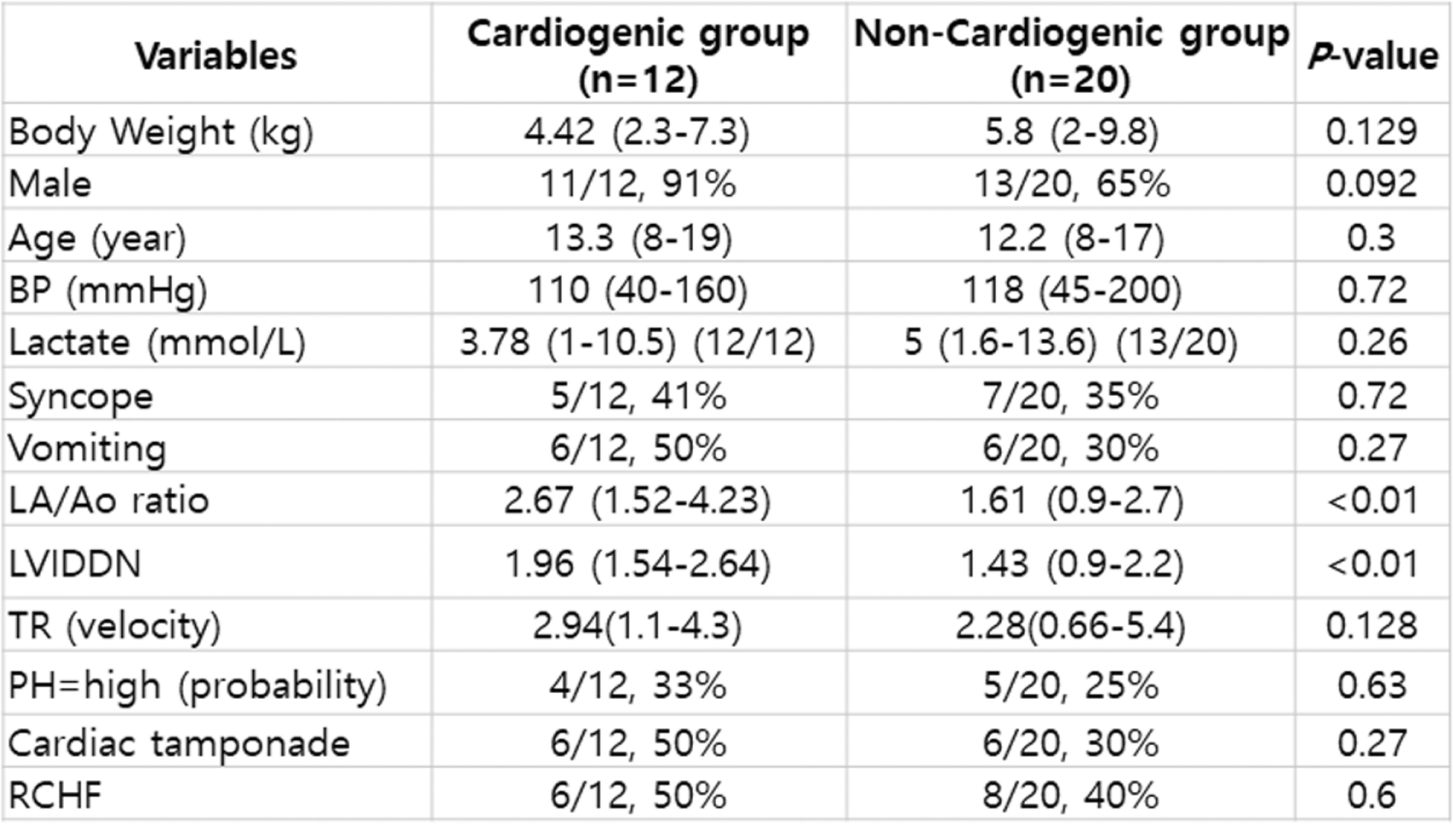
**Table 1** Comparison of clinical signs and echocardiographic indicators between cardiogenic and non‐cardiogenic groups.

## 35 ABSTRACT C35: Comparison of Two Digital Electrocardiographs and Their Associated Computer‐Based Analysis Programs for Recording Dog Electrocardiograms

### 35.1 **Peter Constable** ^1^, BVSc (Hons), MS, PhD, DACVIM (LAIM, Nutrition‐Hon); Tabitha Jones^2^; Christina Wilson^2^; Micah Hernandez^3^; Thomas Jacob^3^; Stuart Nelson^2^

#### 35.1.1 ^1^Professor and Dean, College of Veterinary Medicine, University of Illinois Urbana‐Champaign; ^2^Iditarod Trail Sled Dog Race; ^3^Dextronix, Inc.

**Background:** Small but consistent differences exist for human electrocardiogram (ECG) measurements obtained using digital electrocardiographs and their associated computer‐based analysis programs. We hypothesized that more substantive measurement differences exist between digital electrocardiographs when computer‐based ECG measurements, using software designed and optimized to analyze human ECGs, are obtained from dogs.

**Objectives:** To compare measured ECG intervals and amplitudes using a new wireless veterinary electrocardiograph (VET‐ECG, Dextronix Inc) to those obtained using a reference electrocardiograph designed for human use (Pagewriter XLi M1790A, Hewlett‐Packard Co). To complete a methods comparison study to develop ECG reference intervals for endurance‐trained dogs using the new wireless electrocardiograph.

**Animals:** Convenience sample of 122 healthy endurance‐trained sled dogs.

**Methods:** Six‐lead ECG recordings (leads I, II, III, aVR, aVL, and aVF) were obtained simultaneously using wireless and reference electrocardiographs with dogs in right lateral recumbency. Measurements for the two electrocardiographs were compared using Wilcoxon's signed‐rank test, Deming regression, and Bland‐Altman plots.

**Results:** Differences (*P* < 0.001) were identified in measured median values between wireless and reference electrocardiographs for global ECG intervals and durations, and most lead amplitudes. The differences, expressed as a percentage of the reference electrocardiograph median value, were P‐wave duration (−15.3%), PQ interval (−10.0%), QRS duration (−6.5%), QT interval (−20.2%), and lead II P‐ (−3.0%), Q‐ (+3.9%), R‐ (+15.6%), and T‐wave (+21.2%) amplitude.

**Conclusions and Clinical Importance:** Clinically significant differences in computer‐based measurements were identified when dog ECGs were obtained using two digital electrocardiographs. Reference intervals for dog ECGs may differ depending on the electrocardiograph and its computer‐based analysis program.

## 36 ABSTRACT C36: Computer‐Generated 3D Models in Veterinary Student Education: Improving Radiographic Interpretation of Canine Congenital Heart Disease

### 36.1 **Alexandria Goodyear** ^1^, MS; Lauren Markovic^2^, DVM, DACVIM (Cardiology), FACVIM (Interventional Cardiology); Sherry Clouser^3^, EdD; Brad Gilleland^4^

#### 36.1.1 ^1^Veterinary Student, University of Georgia; ^2^Assistant Professor, Small Animal Medicine and Surgery, University of Georgia; ^3^University of Georgia; ^4^Medical Illustrator, University of Georgia

**Background:** Thoracic radiographic interpretation of congenital heart disease (CHD) is challenging for veterinary students, particularly when translating three‐dimensional (3D) anatomic structures into two‐dimensional (2D) images. This study evaluates the effectiveness of virtual 3D models in enhancing student interpretation of thoracic radiographs.

**Hypothesis:** 3D models will improve students’ spatial and anatomical understanding of healthy and CHD radiographs compared to traditional 2D methods.

**Animals:** Three client‐owned dogs diagnosed with patent ductus arteriosus, pulmonary valve stenosis, and a healthy control were enrolled.

**Methods:** Computed tomography angiography datasets were used to create 3D cardiac models using Materialise software. Virtual models were overlaid onto each corresponding dog's thoracic radiographs. A workshop was conducted with third‐ and fourth‐year veterinary students, divided into two interactive learning groups; 2D traditional methods vs. virtual 3D models with radiographic overlays. Pre‐ and post‐workshop multiple‐choice tests and Likert scale surveys assessed the objective and subjective radiographic interpretation and CHD understanding.

**Results:** Thirty students participated in the workshop. Mean overall pre‐test score was 67.9, and mean post‐test score was 87.5 (*p* < 0.001). Mean percentage increase in pre to post workshop test scores was 16.8 and 22.1 in the 2D and 3D groups, respectively; however, the difference in score improvement between the groups was not significant (*p* = 0.42). Feedback was generally positive with participants preferring 3D over 2D resources.

**Conclusions and Clinical Importance:** Both traditional and 3D methods improved students’ ability to interpret thoracic radiographs in dogs. Participants preferred 3D resources, highlighting their potential as a valuable adjunct tool in veterinary education.

## 37 ABSTRACT C37: Renin‐Angiotensin‐Aldosterone System Metabolites and Enzymes in Dogs With Active and Chronic Congestive Heart Failure

### 37.1 **Autumn Harris** ^1^, DVM, DACVIM (SAIM); Oliver Domineg^2^, PhD; Yang Qu^3^, MS, PhD; Alexis Cooper^4^; Darcy Adin^5^, DVM, DACVIM (Cardiology)

#### 37.1.1 ^1^Associate Professor of Nephrology‐Urology, North Carolina State University; ^2^Attoquant Diagnostics; ^3^Research Associate, Department of Clinical Sciences, North Carolina State University; ^4^Department of Small Animal Clinical Sciences, University of Florida; ^5^Clinical Professor of Cardiology, Small Animal Chief Medical Officers, Department of Small Animal Clinical Sciences, University of Florida

**Background:** The renin‐angiotensin‐aldosterone system (RAAS) is activated in congestive heart failure (CHF), but the relative contributions of advanced heart disease and diuretics are unknown. Additionally, the influence of parenteral furosemide and active CHF are unknown.

**Hypothesis/Objectives:** Our objective was to evaluate RAAS metabolites and enzyme activities between dogs with active and stable naturally occurring CHF. We hypothesized that RAAS variables would be significantly higher in active CHF dogs.

**Animals:** Forty active (pulmonary edema or effusions on exam requiring intravenous furosemide) CHF (38 degenerative valve disease (DVD), 1 dilated cardiomyopathy (DCM), 1 congenital heart disease), and 49 stable (receiving oral furosemide or torsemide without pulmonary edema or effusions on exam) CHF (33 DVD, 14 DCM, 2 congenital) dogs were included.

**Methods:** Prospective study. RAAS metabolites and enzyme activities were quantified by liquid chromatography‐mass spectrometry/mass‐spectroscopy using previously validated methods. Groups were compared using Wilcoxon rank sum test.

**Results:** Angiotensin II (*P* < 0.0001), III (*P* < 0.0001), IV (*P* = 0.0001), 1–5 (*P* < 0.0001), 1–7 (*P* = 0.007), were higher in dogs with active CHF compared to dogs with stable CHF. There was no significant difference in angiotensin I, aldosterone, angiotensin‐converting enzyme (ACE), and ACE2 between active and stable CHF dogs.

**Conclusions and Clinical Importance:** Most RAAS metabolites were higher in dogs with active CHF compared to dogs with stable CHF supporting global activation. Potential reasons include greater potency of intravenous furosemide compared to oral diuretics and sympathetic stimulation associated with active CHF. These results support judicious use of intravenous furosemide and consideration of active vs. chronic CHF in future studies.

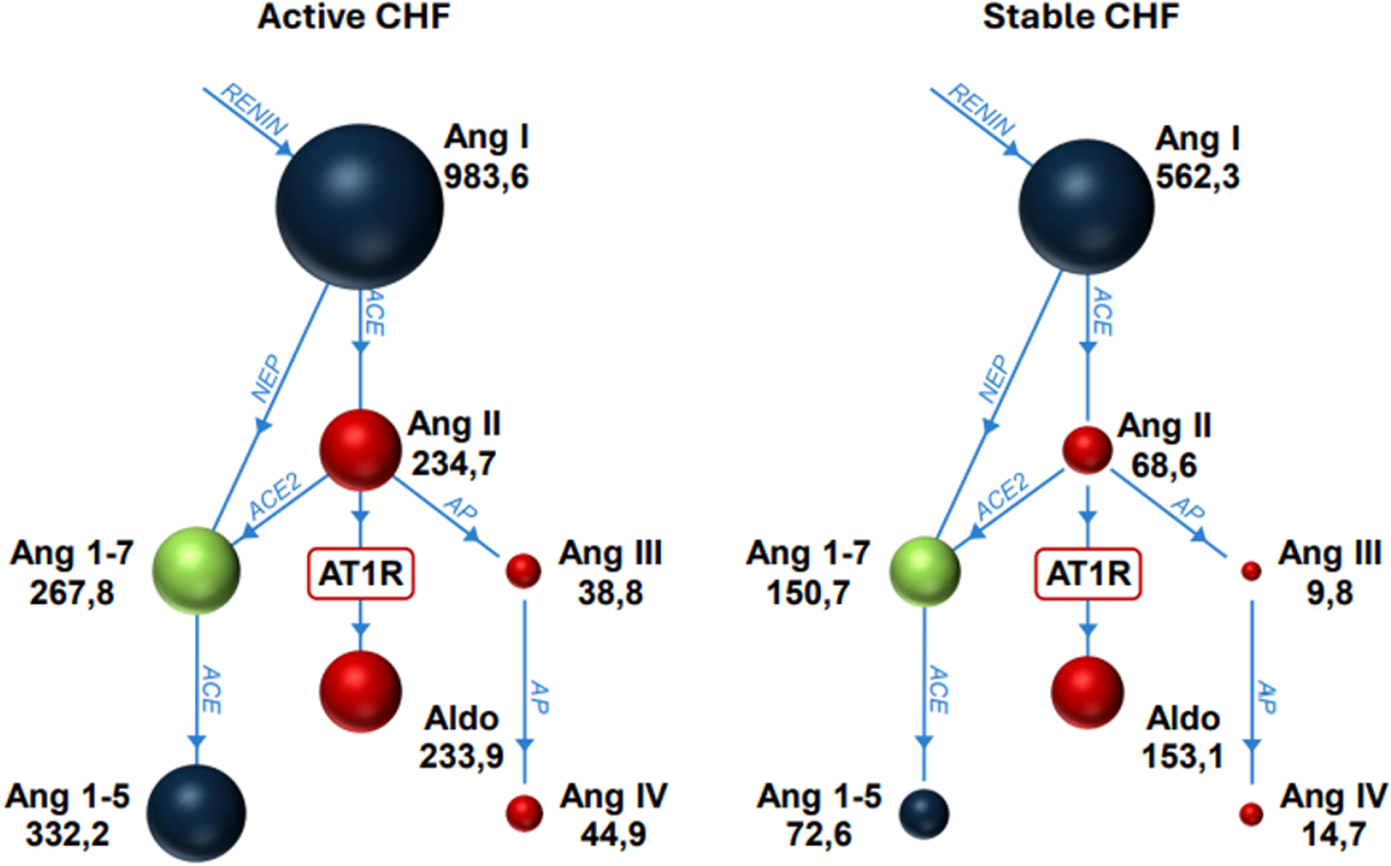

## 38 ABSTRACT C38: The T‐Wave Peak‐End to QT Ratio in Doberman Pinschers With Subclinical Dilated Cardiomyopathy

### 38.1 **Kentaro Kurogochi** ^1^, DVM, PhD; Yu Ueda^2^, DVM, PhD, DACVECC; Kathryn Meurs^2^, DVM, PhD, DACVIM (Cardiology); Joshua Stern^2^, DVM, PhD, DACVIM (Cardiology)

#### 38.1.1 ^1^Research Fellow, North Carolina State University; ^2^North Carolina State University

**Background:** The T‐wave peak‐to‐end to QT ratio (TpTe:QT) is an electrocardiographic marker of myocardial repolarization dispersion that is not influenced by the RR interval.

**Hypothesis/Objectives:** The authors hypothesized that TpTe:QT is prolonged in Doberman Pinschers (DPs) with dilated cardiomyopathy (DCM).

**Animals:** Thirty‐five client‐owned DPs.

**Methods:** This retrospective cross‐sectional study included clinically healthy DPs that are PDK4 and/or TTN genotype positive as the controls, and DPs diagnosed with subclinical DCM (no history of congestive heart failure) as the DCM+ group. Dogs receiving antiarrhythmic drugs were excluded. Using ambulatory electrocardiograms, QT interval, QTc, TpTe, TpTe:QT, and corrected TpTe were measured as repolarization markers. Risk ratios for identifying the DCM phenotype, and interrelationships among RR interval, DCM phenotype, and each marker, were evaluated.

**Results:** Twelve dogs were classified as DCM+ group, and 23 were classified as controls. TpTe:QT was significantly higher in the DCM+ group compared to controls (risk ratio 1.23), with negligible to weak correlation identified to RR interval. TpTe and corrected TpTe were also elevated in the DCM+ group (risk ratios 1.04 and 1.06, respectively); however, these values were significantly influenced by RR interval.

**Conclusions and Clinical Importance:** TpTe:QT is a potential marker for distinguishing Doberman Pinschers with subclinical DCM from those that are normal yet genetically at risk. Its independence from heart rate variation underscores its potential utility in clinical practice. TpTe:QT is a simple, non‐invasive, and readily available test that may predict subclinical DCM in DPs.

## 39 ABSTRACT C39: Copeptin Hormone Concentrations in Dogs With Heart Disease and Its Relationship With Antidiuretic Hormone

### 39.1 **Corine Lavigne** ^1^, DVM; Darcy Adin^2^, DVM, DACVIM (Cardiology); Courtney Hanner^3^; Alexis Cooper^3^; Rebeca Castro^4^; Autumn Harris^5^, DVM, DACVIM (SAIM)

#### 39.1.1 ^1^Small Animal Rotating Intern, University of Florida; ^2^Clinical Professor of Cardiology, Small Animal Chief Medical Officers, Department of Small Animal Clinical Sciences, University of Florida; ^3^University of Florida; ^4^North Carolina State University; ^5^Associate Professor of Nephrology‐Urology, Department of Clinical Sciences, North Carolina State University

**Background:** Antidiuretic hormone (ADH) is upregulated in dogs with cardiac disease, which might indicate renin‐angiotensin‐aldosterone system (RAAS) activation and predispose to electrolyte‐free water retention. However, measurement of ADH is impractical in a clinical setting. Copeptin is co‐secreted with ADH, making it a potentially clinically applicable biomarker of ADH secretion in dogs with heart disease.

**Hypothesis/Objectives:** We hypothesized that dogs with heart disease would have higher copeptin concentrations than healthy dogs and that copeptin concentrations would positively correlate with ADH.

**Animals:** The study population comprised 19 healthy, 20 preclinical, and 20 dogs with congestive heart failure (CHF).

**Methods:** Prospective study. Serum chloride concentration ([Cl^−^]), plasma ADH, and serum copeptin were measured and compared between groups using the Kruskal‐Wallis test. Agreement between ADH and copeptin was assessed with Bland‐Altman analysis. Relationships between copeptin, age, ADH, serum sodium concentration, [Cl^−^], osmolality, and use of renin‐angiotensin‐aldosterone system inhibitors were explored using multivariable linear regression.

**Results:** There were no significant differences in copeptin concentrations between healthy (median 54.9 pg/mL; range 0.5–196.1), preclinical (median 43.6 pg/mL; range 0.5–131.4), and CHF (median 60.5 pg/mL; range 0.5–997.8) dogs. Relative to ADH, copeptin showed a negative proportional bias of −87.8 pg/mL with wide limits of agreement (−421.8–246.2). Linear regression showed significant influence of age and [Cl^−^] on copeptin concentrations.

**Conclusions and Clinical Importance:** Copeptin measured by ELISA does not agree with ADH concentrations in healthy dogs and dogs with cardiac disease, and should not be used as a surrogate marker. Copeptin concentrations are associated with age and [Cl^−^].

## 40 ABSTRACT C40: The Effect of Hypochloremia and Medications on Outcomes in Cats With Stable Congestive Heart Failure

### 40.1 **Corine Lavigne** ^1^, DVM; Darcy Adin^2^, DVM, DACVIM (Cardiology); Natasha Gurdyal^3^; Penny Reynolds^3^, PhD; Autumn Harris^4^, DVM, DACVIM (SAIM)

#### 40.1.1 ^1^Small Animal Rotating Intern, University of Florida, Gainesville, FL, USA; ^2^Clinical Professor of Cardiology, Small Animal Chief Medical Officer, Department of Small Animal Clinical Sciences, University of Florida, Gainesville, FL, USA; ^3^University of Florida, Gainesville, FL, USA; ^4^Associate Professor of Nephrology and Urology, Department of Clinical Sciences, North Carolina State University, Raleigh, NC, USA

**Background:** Hypochloremia is the most common electrolyte abnormality in cats with acute congestive heart failure (CHF). Although associated with poor outcomes in people, its impact on survival in cats with CHF is unclear.

**Hypothesis/Objectives:** We hypothesized that hypochloremia, but not medication use, would be associated with outcomes in cats with stable CHF.

**Animals:** 86 client‐owned cats

**Methods:** Retrospective study. Records of 178 cats (2014–2024) were reviewed. Cats with stable CHF (> 1 week post‐hospitalization) and chemistry panels including serum chloride concentrations (s[Cl^‐^]) from the UF Laboratory were included; those with significant gastrointestinal disease were excluded. Data included clinical, echocardiographic, medication, and outcome variables. Cats were categorized as normochloremic (≥ 112 mEq/L) or hypochloremic (< 112 mEq/L). Group comparisons were made using Mann‐Whitney U or Fisher's exact tests. Correlation between loop diuretic dosage and s[Cl^‐^] was assessed with Spearman's test. Survival was analyzed using Kaplan‐Meier and Cox regression. Pimobendan survival effects were also evaluated, and % CHF time on pimobendan was calculated to assess potential confounding.

**Results:** Median s[Cl^‐^] was 115.3 mEq/L (range 100.0–121.6). Hypochloremia was associated with increased risk of death (HR 4.584; CI 1.327–14.94) and shorter survival (159 vs. 416 days, *P* < 0.0001). Pimobendan was also associated with higher risk of death (HR 2.227; CI 1.220–4.112) and shorter survival (210 vs. 559 days, *P* = 0.014), even when excluding cats with LVOTO (*P* = 0.004).

**Conclusions and Clinical Importance:** Hypochloremia and pimobendan use are risk factors for death in CHF cats. Strategies to normalize s[Cl^‐^] may improve outcomes.

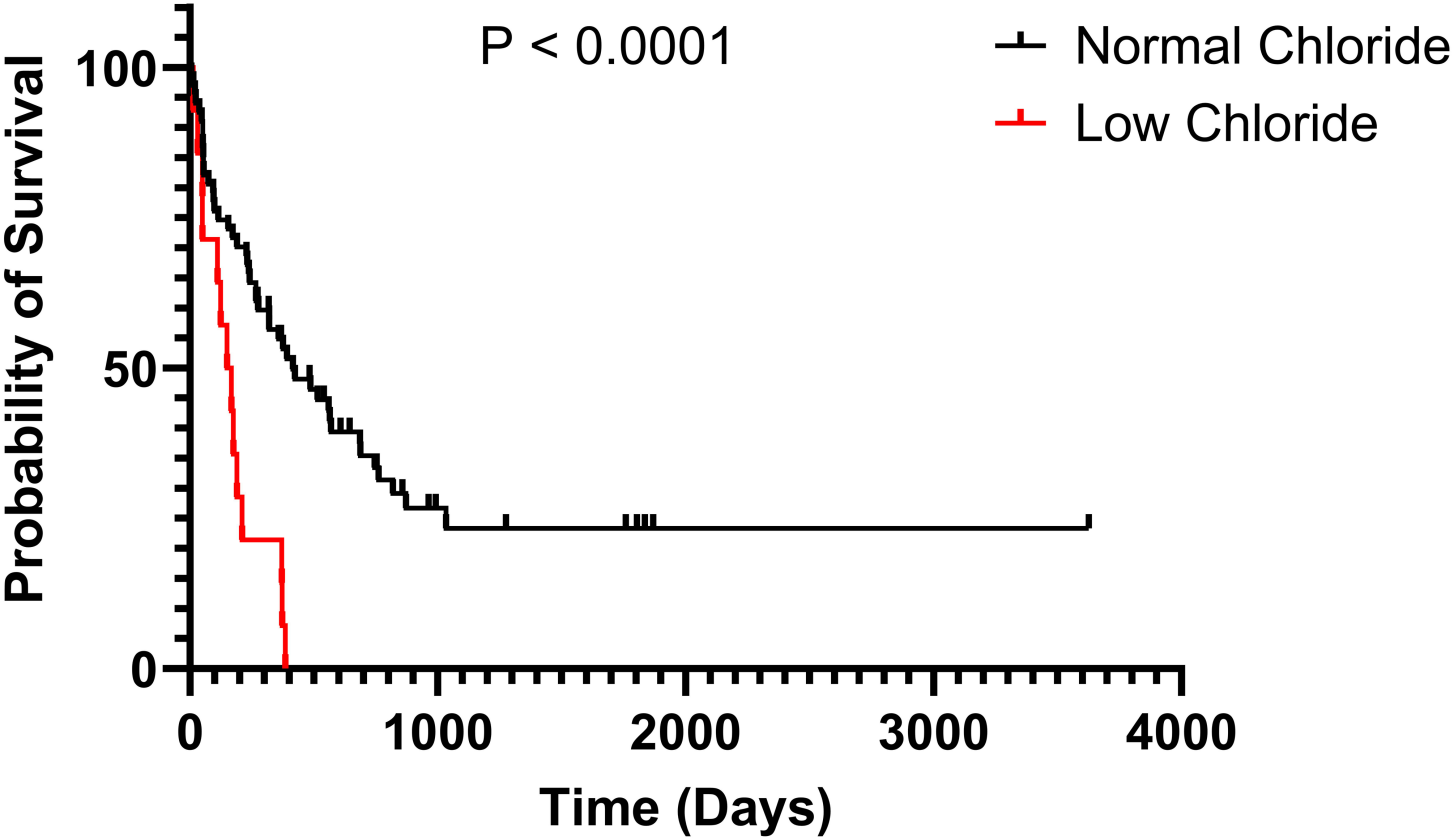

## 41 ABSTRACT C41: Comparison of Holter Monitoring and AliveCor for Arrhythmia Detection in Scottish Deerhounds

### 41.1 **Ilana Levinzon** ^1^, DVM; Miranda Levin^2^; Meg M. Sleeper^3^, DVM, DACVIM (Cardiology)

#### 41.1.1 ^1^Cardiology Research Intern, Department of Small Animal Clinical Sciences, College of Veterinary Medicine, University of Florida, Gainesville, FL, USA; ^2^Research Chair, Scottish Deerhound Club of America; ^3^Clinical Professor; Cardiology Service Chief, Department of Small Animal Clinical Sciences, College of Veterinary Medicine, University of Florida, Gainesville, FL, USA

**Background:** Scottish Deerhounds have a high prevalence of cardiac disease, representing 24% of deaths in UK populations with dilated cardiomyopathy particularly common. While Holter monitoring remains the gold standard for arrhythmia detection, normal reference intervals have not been established for this breed, and the utility of smartphone‐based ECG devices (AliveCor) remains unexplored.

**Hypothesis/Objectives:** We hypothesized that AliveCor recordings can serve as a screening tool for arrhythmia detection in dogs.

**Animals:** 44 client‐owned Scottish Deerhounds recruited by United States breed club

**Methods:** We recorded 24‐h Holter data to establish reference intervals and arrhythmia patterns in 44 Deerhounds. In a subset of 20 dogs, we compared AliveCor recordings to Holter data. Abnormalities were defined as > 100 ventricular or atrial premature complexes on Holter (Dutton et al. 2022) and any arrhythmia on AliveCor. Each dog completed two consecutive 5‐minute AliveCor recordings during sleep, rest, and post‐exercise (10 min per activity), with additional recordings if needed (179 recordings; median 7 per dog; range: 4–32).

**Results:** In 20 dogs with paired recordings, Holter identified abnormalities in 10 (50%) and AliveCor in 9 (45%), showing 85% agreement (k=0.7, *p* = 0.002). AliveCor demonstrated 80% sensitivity and 90% specificity when referencing Holter. Agreement varied by activity, ranking highest during rest, 85% (k=0.7, *p* = 0.001), post‐exercise, 75% (k=0.5, *p* = 0.019), and sleep, 70% (k=0.4, *p* = 0.025).

**Conclusions and Clinical Importance:** The frequency of arrhythmias in healthy Scottish Deerhounds was notable. AliveCor monitor appears to be a useful screening tool for dogs to prioritize testing with Holter monitoring.

## 42 ABSTRACT C42: Evaluating NXSCOPE for Generating PCG Cardiology Reports in Healthy and Cardiac‐Disordered Canines

### 42.1 Gerard O’Leary, PhD

#### 42.1.1 Postdoctoral Fellow, University of Toronto, Toronto, ON, Canada

**Background:** NXSCOPE is a stethoscope attachment combining AI‐driven phonocardiography (PCG) analytics, with patient contexts taken from automated transcription of veterinarian‐client conversations. Timely detection of canine cardiac anomalies is paramount, as subtle changes can significantly affect treatment strategies.

**Hypothesis/Objectives:** We hypothesized that NXSCOPE's integrated approach would produce accurate PCG cardiology reports to serve as a non‐invasive initial diagnostic tool for healthy and cardiac‐disordered canines.

**Animals:** Twenty client‐owned dogs (10 healthy, 10 with diagnosed cardiac conditions) were enrolled from a single veterinary hospital.

**Methods:** During routine exams, NXSCOPE recorded heart sounds and transcribed client‐veterinarian dialogues via a built‐in microphone, automatically extracting patient profiles (e.g., breed, age) to enhance diagnostic accuracy. PCG data were analyzed for murmurs, arrhythmias, and other acoustic anomalies, and findings were compared with echocardiographic assessments. Key vitals (heart rate, temperature, respiratory rate) were also captured and integrated into automated cardiology reports.

**Results:** PCG findings correlate strongly with echocardiographic results. Murmur and arrhythmia, detection sensitivity are possible and are compared with ECG findings. Clinicians report improved workflow efficiency and diagnostic confidence due to integrated patient data and real‐time acoustic analyses.

**Conclusions and Clinical Importance:** NXSCOPE offers a comprehensive, non‐invasive tool for canine cardiac evaluation, enhancing diagnostic accuracy by merging patient profile data with PCG analytics. Its potential to streamline veterinary workflows and support earlier interventions may lead to improved outcomes in canine cardiology.

## 43 ABSTRACT C43: A Novel Cardiac Troponin‐I Missense Variant Is Associated With Familial Hypertrophic Cardiomyopathy in Golden Retrievers

### 43.1 **Victor Rivas** ^1^, MS, PhD; Dayna Goldsmith^2^, DVM, DACVP; Michael Vandewege^3^, PhD; Ronald Li^3^, DVM, MVetMed, PhD, DACVECC; Sandra Losa^3^, MBA, PhD; Megan Leber^3^; Panchan Sitthicharoenchai^3^, MS, DVM, PhD, DACVP; Kim Hawkes^4^, DVM, DACVIM (Cardiology); Jennifer Davies^2^, MVSc, DVM, DACVP; Carolyn Legge^2^, MVSc, DVM, DACVP; Sarah Revell^5^, DVM; Joshua Stern^6^, DVM, PhD, DACVIM (Cardiology)

#### 43.1.1 ^1^Post‐Doctoral Scholar and Veterinary Student, College of Veterinary Medicine, North Carolina State University, Raleigh, NC, USA; ^2^University of Calgary, Calgary, AL, Canada; ^3^North Carolina State University, Raleigh, NC, USA; ^4^Pulse Veterinary Cardiology; ^5^Highview Animal Clinic Ltd.; ^6^Associate Dean for Research and Graduate Studies, Department of Clinical Sciences, North Carolina State University, Raleigh, NC, USA

**Background:** Hypertrophic cardiomyopathy (HCM) is a naturally occurring disease characterized by left ventricular (LV) thickening that commonly affects humans and cats, but rarely dogs. 1,500 and three genetic mutations explain HCM in humans and cats, respectively. To date, no variants have been described in dogs. HCM in a golden retriever (GR) family was identified following sudden cardiac death of three puppies.

**Hypothesis/Objectives:** An underlying deleterious sarcomeric variant that changes protein structure and/or function explains HCM in this GR family.

**Animals:** A whole‐genome association study (WGAS) population of three HCM‐affected puppies and 11 echocardiographically confirmed normal nuclear members (*n* = 14). 2,771 previously sequenced unphenotyped dogs. A validation cohort of 45 HCM‐unaffected GRs. Additional LV tissues from five control dogs.

**Methods:** A WGAS assuming an autosomal‐recessive mode of inheritance (P_Allelic_‐value < 0.024). Allele frequencies of variants were calculated in the unphenotyped cohort of previously WGSed samples and subsequently interrogated in the phenotyped GR population via PCR. LV tissue immunofluorescence of candidate protein(s) was performed for the HCM‐affected and ‐unaffected groups.

**Results:** WGAS identified a single segregating missense mutation in Cardiac Troponin‐I (TNNI3). This variant was not observed in the unphenotyped cohort and only one carrier was identified in the validation population GRs. Immunofluorescent staining of LV tissues did not reveal obvious aberrant protein localization and/or expression at the sarcomeric level.

**Conclusions and Clinical Importance:** This variant represents the first‐ever reported pathogenic HCM variant in any canine species; its identification holds promise for establishing translational models, genetic screening, and early disease prevention.

## 44 ABSTRACT E01: Insulin/Glucose Response to Varying Feeding Methods (Mare's Milk, Milk Replacer, Direct Nursing) in Foals

### 44.1 **Sarah Mazur** ^1^; Laura Hostnik^2^; Hannah Kinsella^2^; Ahmed Kamr^2^; Thomas Bills^2^; Celine Bartish^2^; Ramiro Toribio^2^

#### 44.1.1 ^1^Equine Internal Medicine Resident, The Ohio State University, Columbus, OH, USA; ^2^The Ohio State University, Columbus, OH, USA

**Background:** Variation in feeding methods are common in critically ill neonatal foals. However, the effect of feeding methods on energy regulation has not been investigated in foals and could be relevant to nutritional management.

**Hypothesis/Objectives:** Our objective was to investigate blood glucose (BG) and serum insulin responses in healthy foals to bolus feeding mare's milk (MM), mare's milk replacer (MMR), and lactated Ringer's solution (LRS; control), with direct nursing (DN) from the dam. We hypothesized that DN would enhance the glucose and insulin responses compared to bolus feeding responses.

**Animals:** Twenty‐eight healthy Standardbred foals, less than 96 h of age, were included.

**Methods:** Each foal was randomly assigned to one of four treatment groups: bolus feeding 500 ml MM, MMR, or LRS via nasogastric tube, or DN from the mare for 5 min, each after a 1‐h fast. Blood samples were collected for BG and serum insulin measurements up to 180 min.

**Results:** Area under the curve (AUC) values for BG and insulin were significantly larger in bolus‐fed groups compared to DN and control. Time to maximal BG was longer for DN compared to MM and MMR, and DN maintained higher BG throughout the study period. Insulin concentrations peaked later in the DN group compared to other groups.

**Conclusions:** Our results suggest that while suckling is not necessary for maintaining energy regulation, the DN group demonstrated a more gradual peak in BG and insulin concentrations compared to bolus‐fed groups, and sustained higher BG concentrations for a longer time.

## 45 ABSTRACT E02: Incretin Response to Varying Feeding Methods (Mare's Milk, Milk Replacer, Direct Nursing) in Foals

### 45.1 **Sarah Mazur** ^1^; Laura Hostnik^2^, DVM, MS, DACVIM; Hannah Kinsella^2^; Ahmed Kamr^2^; Hannah Kinsella^2^; Thomas Bills^2^; Celine Bartish^2^; Ramiro Toribio^2^

#### 45.1.1 ^1^Equine Internal Medicine Resident, The Ohio State University, Columbus, OH, USA; ^2^The Ohio State University, Columbus, OH, USA

**Background:** Investigations of the enteroinsular axis (EIA) in neonatal foals suggest that imbalances in incretin responses in critically ill foals may contribute to mortality. Therefore, the impact of feeding method may have clinical relevance.

**Hypothesis/Objectives:** Our objective was to compare the incretin response (glucose‐dependent insulinotropic polypeptide [GIP], glucagon‐like peptide‐1 [GLP‐1], glucagon‐like peptide‐2 [GLP‐2]) in healthy foals to bolus feeding mare's milk (MM), mare's milk replacer (MMR), and lactated Ringer's solution (LRS; control), with direct nursing (DN) from the dam. We hypothesized that DN would enhance the incretin response compared to other groups.

**Animals:** Twenty‐eight healthy Standardbred foals, less than 96 h of age, were included.

**Methods:** Each foal was randomly assigned to one of four treatment groups: bolus feeding 500 ml MM, MMR, or LRS via nasogastric tube, or DN from the mare for 5 min after a 1‐h fast. Blood samples were collected for incretin measurements up to 180 min.

**Results:** The GIP area under the curve (AUC) was larger in the MMR group compared to control. The GLP‐2 AUC was larger in the MM group compared to DN and control, with DN and control larger compared to MMR. Time to maximum incretin concentration (GIP, GLP‐2) was shorter for MM and MMR compared to DN. There were no differences in GLP‐1 AUC among groups. Incretin concentrations appeared to vary with age (0–48 h vs. 48–96 h).

**Conclusions:** The results suggest that the incretin response in neonatal foals is variable amongst feeding methods and may be influenced by age.

## 46 ABSTRACT E03: The Hypothalamic‐Pituitary‐Adrenal Gland Axis Response to a Vasopressin Stimulation Test in Critically Ill Foals

### 46.1 **Myriah Albrecht** ^1^; Nimet Browne^2^; Teresa Burns^3^; Katarzyna Dembek^4^; Javier Perez Quesada^4^

#### 46.1.1 ^1^Equine Internal Medicine Resident, The Ohio State University, Columbus, OH, USA/Hagyard Equine Medical Institute, Lexington, KY, USA; ^2^Hagyard Equine Medical Institute, Lexington, KY, USA; ^3^The Ohio State University, Columbus, OH, USA; ^4^North Carolina State University, Raleigh, NC, USA

**Background:** Sepsis remains the leading cause of death in foals. Hypothalamic‐pituitary‐adrenal gland axis (HPAA) dysfunction, a common complication of sepsis, is associated with decreased survival and can be diagnosed with a vasopressin (AVP) stimulation test.

**Hypotheses/Objectives:** Administration of exogenous AVP will increase systemic ACTH and cortisol concentrations in foals, and this response will be attenuated in critically ill compared to healthy foals.

**Animals:** 14 healthy and 43 hospitalized foals: 19 septic (sepsis score > 12), 24 sick non‐septic (SNS; not critically ill)

**Methods:** A prospective, randomized repeated measures design was utilized to assess HPAA function and compare ACTH and cortisol concentrations at 0‐, 15‐, 30‐, 60‐, and 90‐ min following administration of 5 IU of AVP in healthy, SNS, and septic foals.

**Results:** ACTH and cortisol concentrations increased at 15 and 30 min in all groups compared to baseline (*p* < 0.01). ACTH concentration was lower in septic (150±96 pg/ml) and SNS (110±88 pg/mL) compared to healthy foals (214±127 pg/mL) 15 min after AVP administration (*p* < 0.05). Healthy foals had lower cortisol concentrations at 0‐, 15‐, 60‐, and 90‐min compared to septic foals (*p* < 0.05). Fold increase in ACTH was lower in septic (3.9±3.3) compared to SNS (4.6±3.2) and healthy foals (7.9±3.3) (*p* < 0.01). There was no difference in fold increase of cortisol between groups (*p* > 0.05).

**Conclusion and Clinical Importance:** An IV AVP stimulation test can be used to evaluate the HPAA in critically ill foals. ACTH response to AVP appears to be a more sensitive indicator of HPAA dysfunction in ill foals than cortisol response.

## 47 ABSTRACT E04: Effect of Ertugliflozin on Glucose and Insulin Dynamics in Healthy Horses Receiving Dexamethasone

### 47.1 **Florence Dupuis‐Dowd** ^1^; Emily Berryhill^2^; Philip Kass^2^

#### 47.1.1 ^1^Large Animal Internal Medicine Resident, University of California‐Davis, Davis, CA, USA; ^2^University of California‐Davis, Davis, CA, USA

**Background:** Sodium‐glucose cotransporter 2 (SGLT‐2) inhibitors have proven their efficacy in managing insulin dysregulation (IR) and limiting hyperinsulinemia‐associated laminitis, but the effect of SGLT‐2 inhibitors on corticosteroid induced IR is unknown.

**Hypothesis/Objectives:** To evaluate the effect of ertugliflozin, a SGLT‐2 inhibitor, on insulin and glucose dynamics in healthy horses with concurrent dexamethasone administration. We hypothesized that dexamethasone would cause IR and that coadministration of ertugliflozin would mitigate that effect.

**Animals:** Seven healthy research horses (four geldings and three mares).

**Methods:** Utilizing a randomized crossover design, horses received dexamethasone (0.06 mg/kg IV q48h) (DEX group), or ertugliflozin (0.05 mg/kg PO q24h) plus dexamethasone (SGLT‐2 group) for 7 days. Oral sugar (OST) and insulin tolerance (ITT) tests were performed before and after treatment, and daily serum insulin and glucose were measured during each treatment block. Data were analyzed with mixed effect linear models; *p* value < 0.05 was considered significant.

**Results:** Daily insulin concentrations were lower in the SGLT‐2 group compared to the DEX group (*p* < 0.001) throughout treatment. Glucose was lower in the SGLT‐2 group on days 3 and 5 (*p* < 0.001). The glucoseAUC and Δ insulinT60‐0 from the OST were higher in both groups after treatment (p≤0.01, *p* = 0.014, respectively), without difference between groups. Glucose concentrations decreased less during the ITT post‐treatment in the SGLT‐2 group compared to DEX (*p* < 0.001).

**Conclusions and Clinical Importance:** Co‐administration of ertugliflozin lowered serum insulin in horses administered dexamethasone but did not reduce IR. Ertugliflozin may reduce the risk of hyperinsulinemia associated laminitis in equine patients receiving corticosteroids.

## 48 ABSTRACT E05: Prolonged Wireless Measurement of Intragastric pH in Foals

### 48.1 **Evelyn Hodgson** ^1^; Tallia‐Rume Romano^2^; Allison Stewart, BVSc (Hons I) MS DACVIM (LAIM) DACVECC PhD MANZCVS^3^; Stephanie Bond, BVSc (Hons) BAnimSci PhD^4^; Jessica Wise, BVetBio/BVSc DVStud MANZCVS DECEIM^5^

#### 48.1.1 ^1^Resident in Equine Internal Medicine, University of Queensland, Brisbane, Australia; ^2^Undergraduate Student, School of Veterinary Science, University of Queensland, Brisbane, Australia; ^3^Senior Lecturer and Specialist in Equine Internal Medicine and Emergency and Critical Care, School of Veterinary Science, University of Queensland, Brisbane, Australia; ^4^Lecturer in Veterinary Anatomy, School of Veterinary Science, University of Queensland, Brisbane, Australia; ^5^Specialist in Equine Internal Medicine, School of Veterinary Science, University of Queensland, Brisbane, Australia

**Background:** The pathogenesis of gastric ulceration is not well understood in foals and the relationship with gastric acidity requires further investigation. A wireless capsule, designed for intraesophageal pH monitoring in humans, has been adapted to measure intragastric pH in adult horses.

**Hypothesis/Objectives:** To determine the feasibility of wireless intragastric pH measurement in foals. We hypothesized that the capsules would remain attached for ≥ 48 h.

**Animals:** Eight healthy foals aged 24 to 98 days.

**Methods:** Prospective interventional study. Capsules were attached to the glandular mucosa under gastroscopic guidance using a hemostasis clip and pH was continuously recorded. Gastroscopy was performed when detachment was suspected based on sustained pH ≥ 8, or after 7 days, to assess capsule attachment.

**Results:** The capsules remained attached for 7 days in 6/8 foals (75%), with detachment in 2/8 foals after 5 days. The mean of the mean pH was 3.2±0.6, the mean median pH was 3.0±0.7, and the mean percentage of time the pH was less than 4 (%tpH < 4) was 74.0±16.2%. As age increased, median pH significantly decreased (*r* = ‐0.75, *p* = 0.033) and %tpH < 4 significantly increased (*r* = 0.72, *p* = 0.046). There was no significant correlation between age and attachment duration (*r* = ‐0.23, *p* = 0.58).

**Conclusion and Clinical Importance:** This technique provides a non‐invasive wireless alternative for prolonged measurement of intragastric pH in foals. The effect of age on pH provides additional insight into pathophysiology and warrants further exploration.

## 49 ABSTRACT E06: Investigation of Potential Causes of Equine Fecal Water Syndrome

### 49.1 **Allison Boehm** ^1^; Camille Stewart^2^; Anna Bracken^2^; Valeria Scorza^2^; Gabriele Landolt^2^

#### 49.1.1 ^1^Resident, Colorado; ^2^Colorado State University, Fort Collins, CO, USA

**Background:** Fecal water syndrome (FWS) is the passing of nearly normal feces preceded or followed by a volume of liquid. While FWS is not life‐threatening, it poses a significant burden to horses and people managing them. While many potential causes have been hypothesized few studies have been conducted to identify its cause, and results have been inconsistent or non‐reproducible.

**Hypothesis/Objectives:** The objective is to identify risk factors that may predispose horses to FWS and determine the shedding prevalence of *Salmonella enterica*, *Clostridium difficile*, equine coronavirus, *Neorickettsia risticii*, *Lawsonia intracellularis*, *Cryptosporidium* spp. and *Giardia*.

**Animals:** Fecal samples were collected from 25 horses with FWS and 26 unaffected horses. 117 owners completed a questionnaire including 72 owners of horses with FWS and 45 owners of control horses.

**Methods:** A case‐control study was conducted to look for shared factors between affected horses. Fecal samples were analyzed by PCR and immunofluorescent antibody testing (IFA) to determine shedding frequencies and questionnaire data was compared between groups by chi‐square testing.

**Results:** Data suggests horses with FWS were more likely diagnosed with comorbidities compared to controls. Administration of 3 or more supplements, recent non‐steroidal anti‐inflammatory drug use, and prior use of antibiotics was observed more frequently in horses with FWS. No differences were observed in prevalence of infectious agent shedding.

**Conclusions and Clinical Importance:** Results suggest FWS may be more frequently encountered in horses with certain management practices. While no infectious agent shedding differences were observed, serial testing may be needed to detect intermittent shedding.

## 50 ABSTRACT E07: Duodenal Macrophages and Their Role in Equine Inflammatory Bowel Disease (IBD): A Preliminary Study

### 50.1 **Aurélie Amar** ^1^; Guillaume St‐Jean^2^; Marie‐Odile Benoit‐Biancamano^2^; Nanny Wenzlow^3^; Daniel Jean^4^

#### 50.1.1 ^1^ACVIM‐LA Resident, University of Montreal, Montreal, QC, Canada; ^2^Pathology and Microbiology, Faculty of Veterinary Medicine, University of Montreal, Montreal, QC, Canada; ^3^School of Veterinary Medicine, Texas Tech University, Lubbock, TX, USA; ^4^Clinical Sciences, Faculty of Veterinary Medicine, University of Montreal, Montreal, QC, Canada

**Background:** Intestinal macrophages are key regulators of gastrointestinal homeostasis and inflammation in several species, but their role in equine IBD remains poorly understood.

**Objectives:** The study aimed to 1) Compare macrophage count between duodenal biopsies and their full thickness counterpart, 2) Quantify duodenal macrophages in horses with IBD.

**Animals:** Five horses were examined postmortem and 30 horses (with and without IBD) underwent duodenal biopsies at the equine hospital (CHUV).

**Methods:** Full‐thickness and endoscopic biopsies of the duodenum were obtained postmortem. Duodenal biopsies were performed in 30 horses by gastroduodenoscopy with endoscopic forceps. Tissues were stained immunohistochemically to identify M2 macrophages (CD204) and pan‐macrophages (Iba‐1). Histomorphometric analysis quantified macrophages within the lamina propria of villi (a), the deep lamina propria (b) and the sub‐cryptal area (c). Agreement between biopsies and full‐thickness tissues was assessed with intra‐class correlation, while T‐tests compared macrophage count between IBD and non‐IBD horses.

**Results:** Data revealed that biopsies tend to underestimate CD204 and Iba‐1 positive macrophages compared to full‐thickness tissues. T‐tests indicates significant differences in macrophage count between horses with and without IBD. IBD horses showed significantly higher number of Iba‐1 positive macrophages in the lamina propria of villi (t=3.44, *p* = 0.003) and in all three lamina propria layers combined (a+b+c; t=2.34, *p* = 0.028), compared to horses without IBD.

**Conclusions and Clinical Importance:** IBD horses have significantly higher numbers of duodenal mucosal macrophages, which highlights their potential importance in the diagnostic process and clinical management.

## 51 ABSTRACT E09: Investigation of Cardiac Troponin I, Arrhythmia Burden, and Echocardiographic Variables in Apparently Healthy Neonatal Foals

### 51.1 **Hanna Sfraga** ^1^; Elizabeth Williams Louie^2^, DVM, DACVIM (LAIM); Callum Donnelly^3^, BVSc, PhD, DACT, DACVIM (LAIM); Katharyn Mitchell^3^, BVSc, DVM‐PhD, DACVIM (LAIM)

#### 51.1.1 ^1^Veterinarian, Cornell University, Ithaca, NY, USA; ^2^PhD Student, Cornell University, Ithaca, NY, USA; ^3^Assistant Professor, Cornell University, Ithaca, NY, USA

**Background:** Cardiac troponin I ([cTnI]) is a biomarker for myocardial insult, and reference intervals have been established for adult horses. In neonatal foals, [cTnI], arrhythmia burden, and echocardiographic variables are not well characterized.

**Hypothesis/Objectives:** To establish [cTnI] reference intervals, arrhythmia burden, and echocardiographic variables in healthy foals.

**Animals:** Five University owned foals.

**Methods:** Blood was collected at 0, 6, 12, 18, 24, 48, 72, and 120 h of age, and stored at ‐80°C. Complete echocardiograms were performed at 24 h, 5 days, and 21 days. An ECG was recorded for 24 h, beginning at 12 h of age. Plasma [cTnI] was measured (iSTAT‐1, Abbott Laboratories).

**Results:** Four of five foals had [cTnI] outside the reference interval for adult horses (< 0.06 ng/mL). The [cTnI] peaked at 6 h with a mean (standard deviation) of 0.16 (0.09); decreased at 12, 18, and 24 h: 0.11 (0.07), 0.08 (0.11), and 0.06 (0.06) respectively. An ECG was recorded in 4/5 foals. Analysis revealed supraventricular and ventricular arrhythmias. Echocardiographically, foals had prominent right hearts, changes in tissue Doppler variables compared to adults, pulmonary artery flow acceleration and turbulence, and the ductus arteriosus remained open on day 5 in all foals and was closed at 21 days.

**Conclusions and Clinical Importance:** Arrhythmia burden and [cTnI] in neonatal foals is higher compared to adults. The ductus arteriosus remained open in all foals on day 5 indicating that further investigation to characterize the timing of ductus closure is needed. Five additional foals will be investigated in spring 2025.

## 52 ABSTRACT E10: Effects of Hospitalization on Recumbency, Cortisol, and Heart Rate Variability in Horses

### 52.1 **Andrea Frei** ^1^; Ana Pacheco^2^; Bernadette Stang^2^

#### 52.1.1 ^1^Resident, Large Animal Internal Medicine, Carlson College of Veterinary Medicine at Oregon State University, Corvallis, OR, USA; ^2^Carlson College of Veterinary Medicine at Oregon State University, Corvallis, OR, USA

**Background:** Resting behaviors and stress parameters in hospitalized horses remain poorly characterized.

**Objectives:** To compare recumbency duration, salivary cortisol, and heart rate variability (HRV) in healthy horses across two hospital stall environments and their home environment.

**Animals:** Six healthy mares from a teaching herd.

**Methods:** A case‐control study in which each horse served as its own control. Horses were monitored for three days at home before spending three days in each of the two hospital stalls: HS‐1 (standard indoor stall) and HS‐2 (outdoor‐facing stall with reduced monitoring, added exercise, and nighttime darkness). Recumbency was recorded, salivary cortisol was measured by ELISA, and HRV (SDNN and RMSSD) was assessed via ECG. A mixed‐effects model evaluated environment and day as fixed effects, with horse as a random effect. Pairwise comparisons were adjusted for multiple testing.

**Results:** Recumbency decreased in both hospital environments (HS‐1: ‐99.3 min, *p* = 0.02; HS‐2: ‐92.9 min, *p* = 0.03). Salivary cortisol increased in both hospital environments (HS‐1: +0.04 μg/dL, *p* = 0.02: HS‐2: +0.08 μg/dL, *p* < 0.01). HRV parameters increased in both hospital settings (HS‐1 SDNN: +0.04, *p* = 0.01; HS‐1 RMSSD +0.04, *p* < 0.01; HS‐2 SDNN: +0.07, *p* < 0.01; HS‐2 RMSSD: +0.04, *p* < 0.01). No significant differences were found between HS‐1 and HS‐2.

**Conclusions and Clinical Importance:** Hospitalization increased cortisol and reduced recumbency duration, indicating higher stress and decreased resting behavior. Unexpected HRV increases warrant further study. Environmental modifications in HS‐2 did not mitigate these effects.

## 53 ABSTRACT E11: Pulmonary Disposition and Pharmacokinetics of Ceftiofur Sodium After Nebulization in Adult Horses

### 53.1 **Lana Dedecker** ^1^; Serena Ceriotti^2^, DVM, MS, PhD, DACVIM (LAIM); Mariano Mora^3^, LV, MS, PhD, DACVIM (LAIM); Heather Knych^4^, DVM, PhD, DACVCP; Erin Groover^5^, DVM, DACVIM (LAIM); Kara Lascola^6^, DVM, MS, PhD, DACVIM (LAIM)

#### 53.1.1 ^1^Resident, Large Animal Internal Medicine, Auburn University, Auburn, AL, USA; ^2^Clinical Lecturer, Auburn University, Auburn, AL, USA; ^3^Assistant Professor, Auburn University, Auburn, AL, USA; ^4^Professor, University of California, Davis, CA, USA; ^5^Clinical Professor, Auburn University, Auburn, AL, USA; ^6^Professor, Auburn University, Auburn, AL, USA

**Background:** Ceftiofur sodium is commonly nebulized to treat lung infections in horses. Plasma and pulmonary pharmacokinetics have been described in foals but not in adult horses.

**Hypothesis/Objectives:** This study described pulmonary and plasma pharmacokinetics of desfuroylceftiofur sodium acetamide (DCA) following nebulized and intravenous (IV) administration of ceftiofur sodium in adult horses. It was hypothesized that 1) DCA would be detected in pulmonary epithelial lining fluid (PELF) and exceed minimal inhibitory concentrations (MIC) for gram‐positive (0.12 μg/ml) and most gram‐negative (2 μg/ml) bacteria; 2) nebulization would result in lower systemic (plasma) DCA exposure compared to IV administration.

**Animals:** Six healthy adult university research horses.

**Methods:** A single dose of ceftiofur sodium (2.2 mg/kg) was administered IV or nebulized using a Flexineb® standard medication cup in a randomized cross‐over protocol. Serial plasma samples were collected, and bronchoalveolar lavage was performed and processed to obtain PELF at 1.5, 8, and 20 h. Concentrations of DCA in both plasma and PELF were determined via high pressure liquid chromatography and used for noncompartmental pharmacokinetic analysis. Aerosolized particle size was quantified and rate of nebulization (ml/min) recorded.

**Results:** In PELF, DCA concentrations at 8 h exceeded 0.12 μg/ml and 2 μg/ml in 6/6 and 5/6 horses after nebulization and 3/6 and 2/6 after IV, respectively (Figure 1). Plasma pharmacokinetics are summarized in table 1. Median particle diameter was 4.066 μm with 60% between 1–5 μm.

**Conclusions:** Nebulized administration of ceftiofur sodium results in PELF DCA concentrations above MIC for pathogens encountered in equine bacterial pneumonia.

**Figure:**
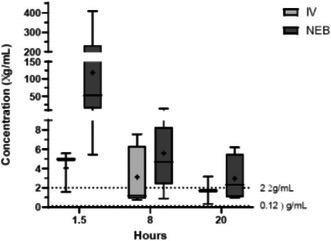
**Figure 1** Box‐plot depicting concentrations of desfuroylceftiofur sodium acetamide (DCA) in the pulmonary epithelial lining fluid (PELF) after nebulization using a standard medication cup (NEB) and intravenous administration (IV) of ceftiofur sodium. Boxes represent interquartile ranges, lines represent median values, and mean values, indicated by +. Note the different scale on the Y axis. For each time point horses were excluded due to PELF DCA concentrations falling below the lower limit of quantification (LOQ) with corresponding adjustments in sample size (n) as follows: NEB 1.5h *n* = 6, 20h *n* = 4; IV 1.5h *n* = 3, 8h *n* = 5, 20h *n* = 3.

**Figure:**
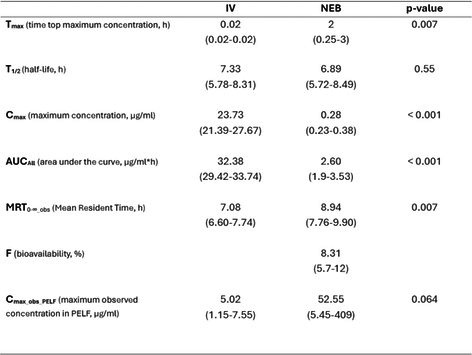
**Table 1.** Median (range) for relevant plasma and pulmonary epithelial lining fluid (PELF) pharmacokinetic variables after administration of a single dose ceftiofur sodium at 2.2 mg/kg IV or nebulized with standard medication cup. IV = intravenous administration NEB = nebulized administration

## 54 ABSTRACT E12: Antimicrobial Prescription Practices to Equine Patients at the Western College of Veterinary Medicine (2019–2023)

### 54.1 **Lorena Santos Bernal** ^1^; Joseph Rubin^2^, DVM, PhD; Fabienne Uehlinger^3^, DVM, DACVIM (LAIM), PhD

#### 54.1.1 ^1^Large Animal Internal Medicine Resident, University of Saskatchewan, Saskatoon, SK, Canada; ^2^Professor, Veterinary Microbiology, Western College of Veterinary Medicine, Saskatoon, SK, Canada; ^3^Associate Professor, Large Animal Clinical Sciences, Western College of Veterinary Medicine, Saskatoon, SK, Canada

**Background:** Antimicrobial resistance is a global problem affecting humans and animals. Of particular concern is resistance to the third‐generation cephalosporins which are critically important to both human and animal health. As antimicrobial resistance follows usage, identifying prescribing patterns is essential for its control. To date, there is a lack of information regarding antimicrobial prescribing practices in equine patients.

**Hypothesis/Objectives:** To describe antimicrobial prescribing practices to equine patients at the Western College of Veterinary Medicine's Large Animal Clinic in Saskatoon, Canada, from 2019 to 2023.

**Animals:** Any horse treated with at least one antimicrobial.

**Methods:** Electronic medical records and client invoices from January 1st, 2019, to December 31st, 2023, were retrieved, and those with antimicrobial billing codes were reviewed. Data including signalment, antimicrobial prescribed, indication, whether a treatment protocol was specified, and whether bacterial culture and sensitivity were performed to guide treatment decisions were extracted.

**Results:** A total of 1,629 records were identified. To date, 1,709 prescriptions from 738 records have been evaluated. The most prescribed antimicrobials were trimethoprim‐sulfamethoxazole (578/1,709; 33.8%), gentamicin (552/1,709; 32.3%), penicillin‐gentamicin combination (231/1,709; 13.5%), and ceftiofur (198/1,709; 11.6%). Regarding ceftiofur, (32/198, 16.2%) of prescriptions were accompanied by culture and sensitivity testing, and (75/198; 38%) were prescribed for musculoskeletal conditions.

**Conclusions and Critical Importance:** This is the first study in Western Canada investigating equine antimicrobial prescription practices. These preliminary results highlight the lack of an evidence‐based approach to antimicrobial prescription, and the frequent usage of third‐generation cephalosporins in equine patients due to their broad‐spectrum activity.

## 55 ABSTRACT E13: The Neutrophil‐to‐Lymphocyte Ratio and Its Association with Outcome in Equine Colitis

### 55.1 **Amanda Samuels** ^1^; Celine Bartish^2^, MS; Payton Kelly^2^, BS; Ahmed Kamr^2^, PhD; Toribio Ramiro^3^, DVM, PhD, DACVIM (LAIM)

#### 55.1.1 ^1^Resident, School of Veterinary Medicine, The Ohio State University, Columbus, OH, USA; ^2^The Ohio State University, Columbus, OH, USA; ^3^Professor, The Ohio State University, Columbus, OH, USA

**Background:** Acute colitis can rapidly become life‐threatening often requiring aggressive medical intervention to reduce the risk of non‐survival. Recognizing horses at a higher risk of non‐survival early in hospitalization is critical for providing appropriate therapeutics and making informed clinical decisions. In humans with ulcerative colitis, the neutrophil‐to‐lymphocyte ratio (NLR) reflects disease severity and in foals, a low NLR is associated with non‐survival.

**Hypothesis/Objectives:** NLR at admission may have prognostic utility for identifying horses that are at increased risk for non‐survival. The objectives of this study: (1) define the NLR at admission in horses diagnosed with colitis and compare them to healthy horses; (2) determine whether the NLR at admission differs between surviving and non‐surviving horses with colitis.

**Animals:** 400 hospitalized horses > 1‐year‐old of any breed and sex: 300 horses diagnosed with colitis and 100 healthy horses.

**Methods:** Retrospective multicenter study. Medical records of horses presenting to four equine referral hospitals were reviewed. Horses were included if they had complete hemograms, final diagnosis of colitis, and outcome data. Data was analyzed by non‐parametric and univariate analysis.

**Results:** The median NLR was similar in healthy horses (median [95% confidence interval]), 2.2 [1.7–2.5]) compared to healthy horses (2.1 [1.7–2.4]). The NLR was lower in surviving horses (1.9 [1.6–2.4]) compared to non‐surviving horses (2.6 [1.4–3.1]). The difference was not significant. Work is ongoing to define the NLR for varying etiologies of colitis.

**Conclusions:** The NLR at admission in horses diagnosed with colitis is not associated with non‐survival.

## 56 ABSTRACT E14: The Predictive Ability of Blood‐Based Biomarkers to Detect Bacteremia in Hospitalized Neonatal Foals

### 56.1 **Amanda Samuels** ^1^; Kelly Hanlon^2^, B.S; Ahmed Kamr^3^, PhD; Niamh Collins^4^, MVB MSc Cert EM DECEIM; Ramiro Toribio^2^, DVM, PhD, DACVIM (LAIM)

#### 56.1.1 ^1^Resident, School of Veterinary Medicine. The Ohio State University, Columbus, OH, USA; ^2^The Ohio State University, Columbus, OH, USA; ^3^Post‐Doctoral Visiting Fellow, The Ohio State University, Columbus, OH, USA; ^4^Veterinarian, Scone Equine Hospital, Scone, Australia

**Background:** Early and accurate identification of septicemia in neonatal foals will improve survival rates. In human medicine, the neutrophil‐to‐lymphocyte ratio (NLR), neutrophil‐to‐monocyte ratio (NMR), monocyte‐to‐lymphocyte ratio (MLR), and plasma cell‐free (cfDNA) concentrations have been used for early identification of bacteremic patients.

**Hypothesis/Objectives:** To determine the utility of the NLR, NMR, MLR, and plasma cfDNA to predict a positive blood culture, and to evaluate the diagnostic performance of these parameters at admission to discriminate between gram‐positive or gram‐negative infection.

**Animals:** 391 hospitalized foals < 5 days old. Inclusion criteria (1) CBC and a blood culture obtained aseptically at admission; (2) documentation of either discharge or non‐survival.

**Methods:** Retrospective. Logistic regression analysis generated predictive models and Area Under the Curve (AUC) was calculated to determine the discriminative ability of independent risk factors and composite models. Plasma concentration of cfDNA was measured using fluorometry.

**Results:** Plasma cfDNA, IgG, NLR, and WBC were identified as independent predictors of positive blood culture and used to generate the composite model (AUC: 0.806 95% CI: 0.665–0.826). Plasma cfDNA, IgG, and neutrophil counts were identified as independent predictors of gram‐negative bacteremia and used to generate the composite model (AUC: 0.807 95% CI: 0.669–0.945). Monocyte counts and age were identified as independent predictors of gram‐positive bacteremia and used to generate the composite model (AUC: 0.670 95% CI: 0.57–0.76). Complete results are in Table 1.

**Conclusions:** We generated composite models that could aid in the identification of bacteremic foals at admission. Models are being validated in the clinic.

**Table:** **Table 3.** Area under the curve characteristics for prediction of blood culture status in hospitalized foals

	AUC	SE	95% CI	Youden Index	% Sensitivity	% Specificity
**Positive blood culture in all hospitalized foals**						
NLR	0.582	0.03	0.521–0.644	< 3.09	49%	68%
TS (g/dL)	0.599	0.04	0.518–0.68	< 4.35	48%	78%
WBC (×10^9^/L)	0.623	0.03	0.562–0.684	< 6.18	61%	61%
cfDNA (ng/mL)	0.670	0.06	0.539–0.789	> 4.67	75%	55%
IgG (mg/dL)	0.639	0.03	0.574–0.704	< 330	44%	81%
Age (h)	0.650	0.02	0.591–0.700	> 23.0	76%	50%
Monocytes (×10^9^/L)	0.590	0.04	0.515–0.665	< 0.02	63%	53%
Neutrophils (×10^9^/L)	0.631	0.04	0.571–0.692	< 4.42	59%	63%
Lymphocytes (×10^9^/L)	0.604	0.03	0.545–0.663	< 1.05	57%	58%
Model 1	0.806	0.05	0.707–0.906	> 0.27	90%	59%
**Gram‐negative blood culture in all hospitalized foals**						
Neutrophils (×10^9^/L)	0.624	0.04	0.536–0.711	< 4.42	60%	63%
PCV (%)	0.656	0.05	0.549–0.762	> 42	47%	77%
cfDNA (ng/mL)	0.682	0.08	0.519–0.843	> 9.06	53%	81%
IgG (mg/dL)	0.730	0.03	0.651–0.870	< 330	60%	81%
Model 2	0.807	0.07	0.669–0.945	< 0.30	82%	80%
**Gram‐positive blood culture in all hospitalized foals**						
Age (h)	0.632	0.04	0.559–0.705	> 23	77%	50%
Monocytes (×10^9^/L)	0.640	0.05	0.534–0.734	< 0.19	62%	60%
WBC (×10^9^/L)	0.610	0.04	0.529–0.690	< 6.34	56%	61%
Neutrophils (×10^9^/L)	0.620	0.04	0.536–0.695	< 7.0	80%	41%
L‐lactate (mmol/L)	0.630	0.04	0.543–0.711	< 3.25	64%	62%
Model 3	0.670	0.05	0.570–0.760	> 0.27	70%	60%
**Polymicrobial blood culture in all hospitalized foals**						
Age (h)	0.764	0.04	0.687–0.841	> 26	64%	71%
NLR	0.730	0.06	0.600–0.860	< 2.66	76%	71%
IgG (mg/dL)	0.702	0.07	0.552–0.851	< 500	69%	73%

Model 1: logit(P)=2.234+(ln(1.146)×cfDNA)−(ln(0.999)×IgG)−(ln(0.864)×NLR)−(ln(0.862)×WBC)

Model 2: logit(P)=1.286+(ln(1.016)×age)−(ln(0.998)×IgG)

Model 3: logit(P)=0.933+(ln(1.081)×cfDNA)−(ln(0.998)×IgG)−(ln(0.846)×Neutrophils)

WBC = white blood cell; NLR = neutrophil‐to‐lymphocyte ratio; PCV = packed cell volume; TS = total solid; cfDNA = cell‐free DNA

## 57 ABSTRACT E15: Evaluation of a Lateral Flow Assay for Failure of Passive Transfer Diagnosis in Crias/Foals

### 57.1 **Taylor Burdette** ^1^; Toby Pinn‐Woodcock^2^, DVM, DACVIM; Tacy Stokol^3^, BVSc, PhD, DACVP

#### 57.1.1 ^1^Resident, LAIM, Cornell; ^2^Associate Clinical Professor, Department of Population Medicine and Diagnostic Services, Animal Health Diagnostic Center, Cornell University; ^3^Professor, Department of Population Medicine and Diagnostic Sciences, Cornell University

**Background:** Serum immunoglobulin G (IgG) measurement is used for assessing passive transfer of immunity in foals and crias, with concentrations < 800 mg/dL and < 1000 mg/dl indicating failure of transfer of passive immunity (FTPI), respectively.

**Hypothesis/Objectives:** Evaluate the accuracy and precision of a stall‐side lateral flow assay (LFA) for FTPI diagnosis in foals and crias.

**Animals:** Serum from 50 foals and 38 crias were analyzed with the LFA and “gold standard” laboratory immunoturbidometric assay (ITA).

**Methods:** Associations between assays, median assay concentrations and the difference between assays were determined using Spearman rank correlation (Rsp), Wilcoxon signed‐rank test, and a Bland‐Altman plot, respectively. Precision was determined by calculating the coefficient of variation (CV) of 5 replicate values of 3 different IgG concentrations. Accuracy for FTPI diagnosis was calculated as the percentage of neonates with LFA results below designated cut‐offs.

**Results:** There was moderate to good correlation between IgG LFA and ITA concentrations in foals (Rsp=0.740, *p* = 0.001) and crias (Rsp=0.809, *p* < 0.001). However, compared to the ITA, median LFA IgG concentrations were significantly lower in foals (LFA: 799 mg/dL, ITA: 1036 mg/dL, *p* = 0.002; median bias: −198 mg/dL) and higher in crias (LFA: 3000 mg/dL; ITA: 850 mg/dL, *p* ≤ 0.001; median bias: 1155 mg/dL). Replicate analysis with the LFA yielded high variation (CV: 27–49% in foals, 42–66% in crias). Of 16 foals and 23 crias with ITA concentrations below the FTPI cut‐off, 88% of foals and 48% of crias would have an FTPI diagnosis with the LFA.

**Conclusion/Clinical Importance:** The stall‐side LFA should not be used to diagnose FTPI in foals or crias.

## 58 ABSTRACT E16: Hemoperfusion With a Novel Cytokine Adsorption Device Reduces Markers of Systemic Inflammation in LPS‐Treated Horses

### 58.1 **Kallie Hobbs** ^1^; Bethanie Cooper^2^, DVM; Megan Burke^3^, DVM, DACVS, DAVECCS; Yu Ueda^4^, DVM, PhD, DACVECCS; Mary Sheats^5^, DVM, PhD, DACVIM (LAIM)

#### 58.1.1 ^1^Assistant Professor, Texas A&M; ^2^Graduate Student, North Carolina State University; ^3^Associate Professor, North Carolina State University; ^4^Assistant Professor, North Carolina State University; ^5^Full Professor, North Carolina State University

**Background:** Sepsis is a common condition in adult and neonatal horses. Despite substantial advances in medical management, sepsis continues to be a leading cause of illness and death in these patients. Hemoperfusion through a novel plasma cytokine adsorption device (CytoSorb®) has shown promise as an adjunctive therapy in humans with sepsis.

**Hypothesis:** LPS‐treated horses hemoperfused through a novel cytokine adsorption device (VetResQ) will have lower levels of systemic inflammatory markers compared to sham‐filtration.

**Animals:** Six healthy, university‐owned horses. IACUC: 23‐110.

**Methods:** 6 horses received 60 ng/kg LPS, IV as a bolus then 60 ng/kg IV as a CRI over 1 h. Systemic inflammation was confirmed by clinical signs and neutropenia. In a cross over model, hemoperfusion was performed for 4 h with either a sham or polymer column. Blood was collected at 5 time points over a 72 hr period for flow cytometry analysis of neutrophils and 10 timepoints for cytokine multiplex analysis.

**Results:** In an *in vivo* LPS‐induced model of systemic inflammation, column‐ vs sham‐treated horses showed more rapid normalization of leukocyte counts, stabilization of reactive oxygen species and increased early neutrophil apoptosis (*p* = 0.01). Systemic cytokines were not significantly different between column and sham treatment.

**Conclusions:** These results provide proof‐of‐concept for hemoperfusion as a potential treatment to mitigate deleterious LPS‐induced immune responses in adult horses. Further research is needed to determine the anti‐inflammatory mechanism(s) of hemoperfusion therapy.

## 59 ABSTRACT E17: Formalin and Aminocaproic Acid, but Not Yunnan Baiyao, Demonstrate Viscoelastic Procoagulant Effects in Healthy Horses

### 59.1 **Ana Nobrega** ^1^; Ina Mersich^2^; Rebecca Bishop^2^, DVM, MS, DACVS (LA); Scott Austin^2^, MS, DVM, DACVIM (LA); Anne Barger^2^; Sofia Rivera Maza^2^; Pamela Wilkins^2^, DVM, PhD, DACVIM (LA), DACVECC

#### 59.1.1 ^1^Resident, Equine Internal Medicine, University of Illinois,; ^2^University of Illinois

**Background:** Procoagulant and antifibrinolytic drugs, such as formalin, aminocaproic acid (ACA), and Yunnan Baiyao (YB), are used to treat bleeding/coagulopathy in horses. Limited research supports efficacy in horses.

**Hypothesis/Objectives:** Formalin, ACA, or YB would alter viscoelastic coagulation in healthy horses.

**Animals:** Seven healthy mares from university research herd.

**Methods:** Prospective, randomized block design with a 2‐week washout between treatments. Formalin and ACA were administered slowly IV in 1 L saline. YB was administered by nasogastric tube. CBC, fibrinogen, and point‐of‐care (POC) viscoelastic testing were performed 1 hr before (T0), 1 hr after treatment (T1), and at recommended redosing interval (T2; formalin 24 hrs, ACA 6 hrs, YB 8 hrs). The effect of time was determined by one‐way repeated measures ANOVA or Friedman test with post‐hoc t‐test or Wilcoxon test based on normality.

**Results:** Formalin and ACA, but not YB, showed significant effects. Formalin resulted in significant increases in A20 (*p* = 0.01), MCF (*p* = 0.005), LI45 (*p* = 0.006), and platelet count (*p* = 0.04) over time. ACA resulted in significant increases in A10 (*p* = 0.01), A20 (*p* < 0.001), MCF (*p* < 0.001), and platelet count (*p* < 0.001), as well as a significant decrease in fibrinogen (*p* = 0.001). There was no statistically significant effect of YB for any measured parameters. The ACA treatment group demonstrated significant differences between individual time points for A20 (T0 vs T1, *p* = 0.026; T0 vs T2, *p* = 0.047) and MCF (T0 vs T1, *p* = 0.016).

**Conclusions and Clinical Importance:** These results suggest that formalin and ACA promote clot formation and stability in healthy horses. Further study in horses with clinical coagulopathy is indicated.

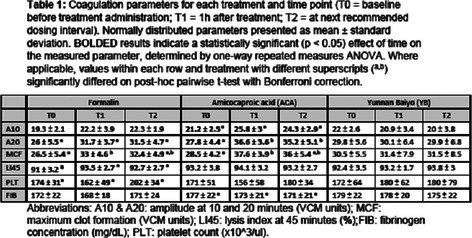

## 60 ABSTRACT E18: Evaluation of Two POC Measurement Systems for ACT and aPTT in Equine Blood

### 60.1 **Kallie Hobbs** ^1^; Yu Ueda^2^, DVM, PhD, DACVECCS; Mary Sheats^2^, DVM, PhD, DACVIM (LAIM); Megan Burke^2^, DVM, DACVS, DACVECCS

#### 60.1.1 ^1^Assistant Professor, Texas A&M; ^2^North Carolina State University

**Background:** There is a lack of information on performance of point of care (POC) device measurement of activated clotting time (ACT) and activated partial thromboplastin time (aPTT) concentration in horses.

**Hypothesis:** Measurement of ACT and aPTT with two point of care analyzers, Vet‐Tube (ACT POC) or Coag DX (aPTT POC), in heparinized and non‐heparinized equine blood will be accurate and precise.

**Animals:** Thirty healthy university‐owned horses. IACUC 23‐254.

**Methods:** Whole blood samples (10 mL) without anticoagulant were collected from 30 horses. Fifteen of the 30 horses had an additional 10 mL sample collected that was treated with 7 μl of heparin. ACT and aPTT were measured for all samples using the Vet‐Tube (ACT POC), Coag DX (aPTT POC) and conventional stationary analyzers (Medtronic ACT or Stago Chemistry) within 30 min of collection.

**Results:** Samples were analyzed using Pearson correlation and Bland Altman analysis. ACT POC and stationary analyzer values showed moderate correlation, acceptable bias and a low coefficient of variation; and were in good agreement for both non‐heparinized (*n* = 30, *p* = 0.64) and heparinized samples (*n* = 15, *p* = 0.43). aPTT POC and stationary analyzer values showed strong correlation but high bias and a high coefficient of variation. The aPTT POC and stationary chemistry analyzer values were in poor agreement for non‐heparinized samples (*n* = 30, *p* = 0.0002) but had good agreement for heparinized samples (*n* = 15, *p* = 0.03).

**Conclusions:** ACT POC measurement is accurate and precise and could present a useful low‐cost option for patients with coagulopathy or receiving heparin therapy. aPTT POC measurement may need further optimization.

## 61 ABSTRACT E19: Blood Leukocyte Telomere Length Dynamics in the First Year of Life in Thoroughbreds

### 61.1 **Alexandra Moss** ^1^; Megan Power^2^; John Browne^3^; Orlagh O’Reilly^4^; Emmeline Hill^5^; Lisa Katz^6^, DVM, MS, PhD, DACVIM, DECEIM, DECVSMR, MRCVS, CertUT

#### 61.1.1 ^1^Clinical Assistant Professor in Equine Medicine, University of Nottingham; ^2^Postdoctoral Research Fellow, School of Biology and Environmental Science, University College Dublin; ^3^Chief Technical Officer, School of Agriculture and Food Science, University College Dublin; ^4^University College Dublin; ^5^Professor in Genetics, School of Agriculture and Food Science, University College Dublin; ^6^Professor in Equine Medicine, School of Veterinary Medicine, University College Dublin

**Background:** Telomeres maintain genomic integrity, shortening as an animal ages. Telomere length (TL) dynamics are proposed as welfare markers since stress accelerates telomere attrition (TA) rate while exercise lengthens sequences. This requires understanding normal TL dynamics at all stages of life. TA rate is greatest during high growth periods, slowing after maturation has been reached, although lengthening has also been reported to occur. TL dynamics have not been investigated during rapid growth in horses.

**Hypothesis:** TL dynamics differ between young horses and adults.

**Animals:** Privately‐owned Thoroughbred mare‐foal dyads (*n* = 34) from one farm.

**Methods:** A prospective cohort study was performed. Venous blood was collected from mare‐foal dyads every 3 months from birth for 12 months (T0–T4), stored at −80C until analysis. DNA was extracted from blood leukocytes and relative TL (RTL) assessed by RT‐qPCR. The eHBB was the single copy gene (S) used, and RTL presented as a calibrated normalised T/S ratio (QBASE+ software), transformed into z‐scores for analysis. Linear mixed models were fitted with covariates (foal sex, birth weight/mare body condition score [BCS], dam/sire age).

**Results:** Mare RTL did not change between timepoints. Foal RTL at T0 was not associated with mare RTL. Foal RTL was greater at T1 (*p* = 0.017) and T4 (*p* < 0.001) compared to T0. None of the covariates significantly affected foal TL.

**Conclusions and Clinical Importance:** These results demonstrate that telomere lengthening occurs in developing horses. Understanding TL dynamics including potential parental and environmental influences is fundamental to interpreting the effects of stress and exercise on TA rate.

## 62 ABSTRACT E20: Prospective Categorisation of Early Maternal Care Style in Thoroughbred Broodmares

### 62.1 **Alexandra Moss** ^1^; Orlagh O’Reilly^2^; Amy Rand^3^; Siobhan Mullan^4^; Kathryn Mitchell^5^, DVM, PhD, BVSc, DACVIM (LAIM); Emmeline Hill^6^; Michael Salter‐Townshend^7^; Lisa Katz^8^, DVM, MS, PhD, DACVIM, DECEIM, DECVSMR, MRCVS, CertUT

#### 62.1.1 ^1^Clinical Assistant Professor in Equine Medicine, University of Nottingham; ^2^University College Dublin; ^3^School of Veterinary Medicine, University College Dublin; ^4^Professor in Animal Welfare and Behavioural Medicine, School of Veterinary Medicine, University College Dublin; ^5^Assistant Professor, Section Chief, Section of Large Animal Internal Medicine, Department of Clinical Sciences, Cornell University; ^6^Professor in Genetics, School of Agriculture and Food Science, University College Dublin; ^7^Assistant Professor in Statistics, School of Mathematics and Statistics, University College Dublin; ^8^Professor in Equine Medicine, School of Veterinary Medicine, University College Dublin

**Background:** The quality of early maternal care style (MCS) has long‐lasting influences on a neonate's subsequent stress response and behavioural phenotype, with poor MCS linked to poor stress adaptation later in life.

**Hypothesis:** Early MCS can be determined in Thoroughbreds by categorising protective and nursing behaviours, validated through tests assessing maternal bond (separation) and fearfulness (novel object).

**Animals:** Privately‐owned Thoroughbred broodmares (*n* = 36, 4–23 years old) from one farm.

**Methods:** A prospective cohort study was performed. Ethogram and qualitative behavioural (QBA) assessments of mare‐foal interactions were quantified over the first 2 weeks after parturition. Behavioural and physiological measurements (salivary cortisol, heart rate variability) were taken from broodmares during a 3‐minute mare‐foal separation test and a 5‐minute novel object test.

**Results:** Ethogram and QBA results were significantly correlated (*r* = 0.41, *p* = 0.01; Pearson's correlation), with 3 principal components responsible for 56.4% of variation in behaviours (Principal component analysis). Two distinct clusters corresponding to high and low MCS phenotypes were identified (k‐means clustering), with successful nursing (*p* = 0.02) and distancing down (*p* = 0.0037) ethogram behaviours differing significantly between phenotypes. Probit regression analysis confirmed MCS phenotypes to be reliably predicted by the maternal bond and fearfulness tests, with a high area under the ROC curve (0.87).

**Conclusions and Clinical Importance:** Ethogram/QBA‐based assessments of maternal care in Thoroughbreds provide robust MCS phenotypes. This has applications for studies on the effect of early MCS on the stress response in equine neonates later in life, aiming to improve welfare and attenuate stress‐related diseases.

## 63 ABSTRACT E21: Computed Tomographic Detection and Management of Pneumolabyrinth in Three Horses with Temporohyoid Osteoarthropathy

### 63.1 **Camilla Jamieson** ^1^; Jack Jarvis^2^; Ori Nagasaki^2^; Masahiro Murakami^3^, BVSc, PhD, DACVR; Ana Pinto^4^, DVM, PhD, DACVR

#### 63.1.1 ^1^Clinical Assistant Professor, Large Animal Emergency Medicine, Purdue University; ^2^Resident, Diagnostic Imaging, College of Veterinary Medicine, Purdue University; ^3^Assistant Professor, Diagnostic Imaging, College of Veterinary Medicine, Purdue University; ^4^Associate Professor, Diagnostic Imaging, College of Veterinary Medicine, Purdue University

**Introduction:** Pneumolabyrinth is recognized in humans and a dog, as a condition characterized by presence of air within the vestibule of the inner ear, following traumatic injury, perilymphatic fistula or otitis. Pneumolabyrinth is associated with severe vestibular ataxia, and successfully treated with anti‐inflammatories and antibiotics, resulting in clinical and radiographic resolution.

**Case 1:** 8‐year‐old AQHA mare, presented for acute onset right cranial nerve VII lesions, without vestibular ataxia, underwent standing head CT that identified grade 3 temporohyoid osteoarthropathy (THO). Eight weeks following successful partial ceratohyoidectomy, she re‐presented with grade IV/V vestibular ataxia. Repeat head CT revealed progressive otitis media and discrete regions of air accumulated within the vestibule of the inner ear, or pneumolabyrinth, see Figure 1.

**Case 2:** 4‐year‐old AQHA mare presented for rapidly progressive vestibular ataxia and right cranial nerve VII deficits underwent a standing head CT. Grade 3 THO was identified, with otitis media and pneumolabyrinth.

**Case 3:** 17‐year‐old Haflinger mare presented for a 5‐day history of grade IV/V vestibular ataxia and left CN VII signs. Standing head CT revealed grade 3 THO, otitis media and pneumolabyrinth.

**Management:** All cases were treated with a taper‐course of oral prednisolone, trimethoprim‐sulfamethoxazole, and 4+ weeks of strict stall rest.

In all three cases, vestibular ataxia resolved within 14 days. Case 1 received a follow up head CT at the resolution of vestibular ataxia, confirming resolution of the pneumolabyrinth.

**Conclusions:** Pneumolabyrinth should be considered in horses with THO presenting with vestibular ataxia, and can resolve with medical management, similar to reports in other species.

**Figure:**
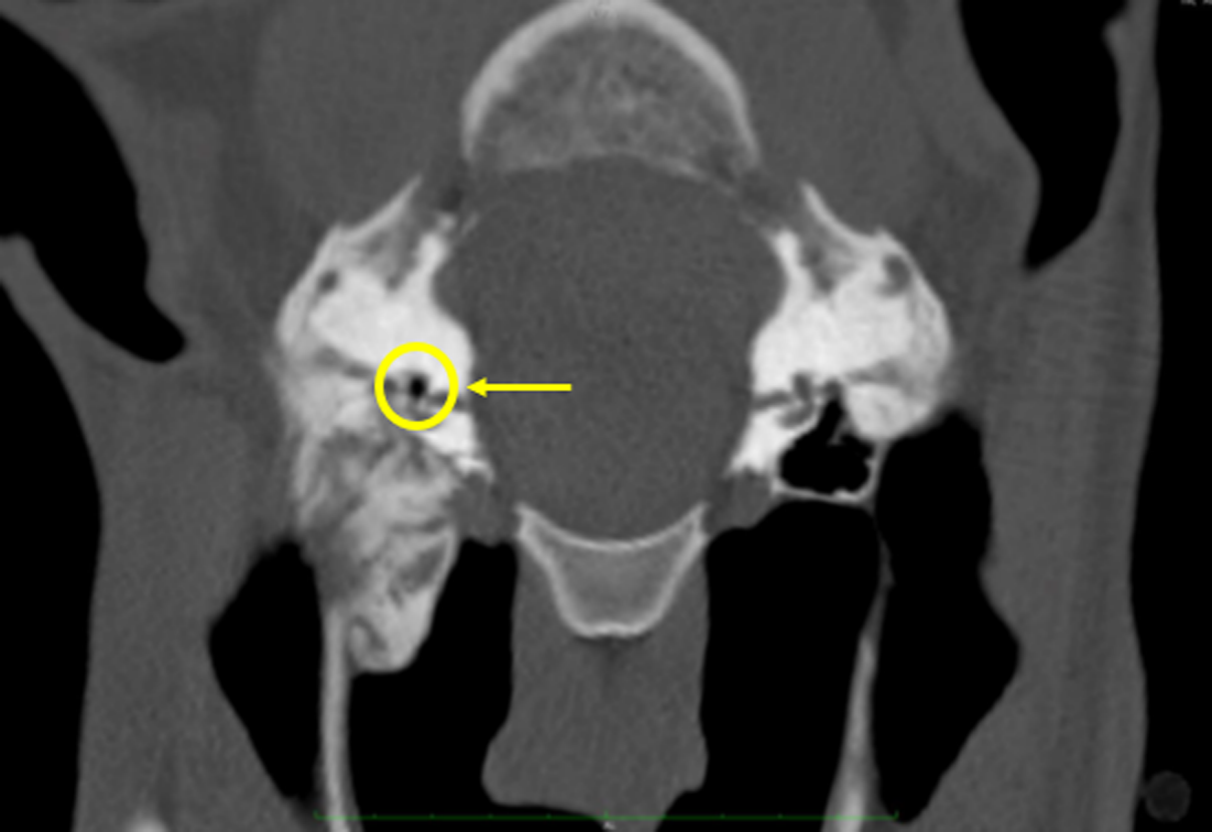
**Figure 1. CT Image of right sided THO with pneumolabyrinth.** Transverse images in bone windowing at the level of the middle ear: Right sided Grade 3 temporohyoid osteoarthropathy, otitis media, and pneumolabyrinth, highlighted by the yellow arrow.

## 64 ABSTRACT E22: Diagnostic Modalities for the Identification of Metastatic Neoplasia: A Clinical Series of 40 Cases

### 64.1 **Isabelle Desjardins** ^1^; Eve Ramery^2^, DACVP, DECVP, PhD; Marta David^3^, DVM

#### 64.1.1 ^1^Assistant Professor Equine Internal Medicine, VetAgro Sup, University of Lyon; ^2^Assistant Professor in Clinical Pathology, Clinical Pathology, VetAgro Sup, University of Lyon; ^3^ECEIM Resident, Equine Department, VetAgro Sup

**Background:** Diagnosis of metastatic neoplasia is often challenging and belated.

**Hypothesis/Objectives:** To assess the success rate of ante‐mortem identification of metastatic neoplasia.

**Animals:** Hospitalized equines between 2009–2024.

**Methods:** Retrospective clinical study. Inclusion criteria were the identification of a primary neoplasm with a final ante‐ or post‐mortem diagnosis of metastatic malignancy, with at least one other site or organ affected. Clinical data, nature of tumour and diagnostic methods were recorded.

**Results:** Among the 40 cases with metastatic tumour(s), lymphoma (*n* = 13, 32.5%) and squamous cell carcinoma (*n* = 9, 22.5%) were the most common. Median age was 15.5‐year‐old (3–29). Ante‐mortem diagnosis was achieved in 77.5% (31/40) of cases. Masses were observed on the body surface in 32% (13/40) and were palpated rectally in 9/40 (22.5%). A single imaging technique per anatomical site (endoscopy, ultrasound, or radiography) was required in 30% of cases (12/40), two modalities in 42.5% (17/40), three in 25% (10/40) and four for a single horse (2.5%). Neoplastic characterisation was performed using biopsy histology (21/27; 77.8%), body fluid cytology (8/33; 24.2%), blood smear cytology (3/14; 21.4%), fine‐needle aspiration of the mass (8/13; 61.5%) and/or cytology of biopsy imprint (3/3; 100%). On average, 2.25 modalities were required/horse to characterise the primary neoplasm. An equivalent examination on the same site (cytology or histology) had to be repeated 2 times in 4 cases and 3 times in 1 case, before tumour cells could be identified.

**Conclusion‐Clinical Importance:** Combining several sampling sites and techniques considerably increases the probability of detecting and characterizing neoplastic cells.

## 65 ABSTRACT E23: Progesterone Administration Decreases Airway Obstruction in Severe Equine Asthma

### 65.1 **Sophie Mainguy‐Seers** ^1^; Justine Herlemont^2^, DVM; Jean‐Pierre Lavoie^3^, DMV, DACVIM; Mathilde Leclère^4^, DVM, DACVIM (LAIM), PhD

#### 65.1.1 ^1^Assistant Professor in Equine Internal Medicine, Université de Montréal; ^2^Intern, Université de Montréal; ^3^Honorary Professor, Université de Montréal; ^4^Full Professor, Université de Montréal

**Background:** Asthma symptoms can vary during the menstrual cycle in women. Similarly, airway obstruction is attenuated during the luteal phase in mares affected by severe asthma (SEA), a phenomenon that could be related to the relaxing effect of progesterone on bronchial smooth muscle.

**Objectives:** To evaluate the effects of progesterone on airway obstruction and inflammation in SEA.

**Animals:** 4 mares and 4 geldings with SEA from a research herd.

**Methods:** In a randomized blinded cross‐over study with a 3‐week washout period, the effect of a 10‐day daily oral altrenogest treatment (0.044 mg/kg) was compared to that of dexamethasone (0.06 mg/kg). Lung function, tracheal mucus accumulation and airway neutrophilia were assessed before and after treatment. Data were analyzed with two‐way ANOVAs and Dunnett's corrections.

**Results:** Both treatments improved pleural pressure, pulmonary resistance and elastance overtime (*p* < 0.0001), although dexamethasone was more effective (treatment group difference (*p* < 0.05) and time‐treatment group interaction (*p* < 0.05); pleural pressure reduction from 30.7±7.7 to 21.9±8.8 cm H_2_O with progesterone (*p* = 0.02) and from 30.0±11.3 to 8.5±1.5 cm H_2_O with dexamethasone (*p* < 0.0001)). The response was similar between mares and geldings. The mucus accumulation and airway neutrophilia remained unchanged.

**Conclusions and Clinical Importance:** The partial relief of airway obstruction by progesterone suggests its potential use as an adjunct therapy in SEA mares that display respiratory distress fluctuation during the estrus cycle. Whether altrenogest modifies the lung function and performance of healthy and mild asthmatic mares require investigation.

## 66 ABSTRACT E24: Poor Agreement Between Resting Tracheal Mucus Scores and Bronchoalveolar Lavage Cytology in Racing Thoroughbreds

### 66.1 **Carla Olave** ^1^; Katy Ivester^2^, DVM, PhD, DACVS; Laurent Couëtil^2^, DVM, PhD, DACVIM

#### 66.1.1 ^1^Equine Internal Medicine Specialist, The Equine Hospital, Jockey Club of Saudi Arabia; ^2^Purdue University

**Background:** Equine asthma is diagnosed by a combination of clinical signs and demonstration of inflammation upon bronchoalveolar (BAL) cytology and/or endoscopic examination of the airway. The degree of agreement between cytology and endoscopic mucus scores in resting horses is controversial.

**Hypothesis/Objectives:** Endoscopic tracheal mucus score is a poor predictor of BAL cytology in Thoroughbred racehorses at time points distant from strenuous exercise.

**Animals:** 124 Thoroughbred racehorses housed at racetracks in the USA.

**Methods:** BAL cytology, mucus scores, and exercise‐induced pulmonary hemorrhage (EIPH) scores were collected during two prospective‐randomized trials from horses during non‐racing days. Generalized linear mixed models (GLMM) were constructed to determine if tracheal mucus scores predicted BAL inflammatory cell proportions, controlling for age and EIPH scores. Cohen's kappa was calculated to determine agreement between mucus score and BAL cytology in the diagnosis of asthma. Significance was set at *p* < 0.05.

**Results:** BAL Neutrophil proportions were inversely related to mucus score (*p* < 0.0001) but positively related to EIPH score (*p* < 0.0001) and age (*p* < 0.0001). BAL mast cell proportions were not related to mucus score (*p* = 0.58). BAL eosinophil proportions were positively related to the EIPH score (*p* < 0.0001) and negatively related to age and mucus score (*p* < 0.0001). No agreement was observed between mucus score and BAL cytology (κ≤0.02)

**Conclusions and Clinical Importance:** Endoscopic mucus score is not a good predictor of lower airway inflammation when not performed shortly after racing. A positive relationship was observed between the EIPH score and neutrophilic and eosinophilic inflammation. Further investigation of the relationship between EIPH and airway inflammation is warranted.

## 67 ABSTRACT E26: Serum Total Adiponectin Variation Over 24 h in Grazing Horses With and Without Insulin Dysregulation

### 67.1 **Morgan Askins** ^1^, MS; Amanda Adams^2^; Patricia Harris^3^; Erica Jacquay^2^; Brittany Kerley^2^; Margaret McClendon^2^; Andrew van Eps^4^

#### 67.1.1 ^1^Graduate Research Assistant, M.H. Gluck Equine Research Center, Department of Veterinary Science, University of Kentucky; ^2^University of Kentucky; ^3^Waltham Petcare Science Institute; ^4^University of Pennsylvania

**Background:** Serum insulin varies in response to pasture nutrient content across the day in grazing horses; therefore, sample timing can greatly influence results. Total adiponectin is a potentially useful marker of insulin dysregulation (ID) and laminitis risk, but its temporal variability in grazing horses is unknown.

**Hypothesis/Objectives:** To examine temporal changes in serum total adiponectin over a 24‐h grazing period in horses.

**Animals:** Twelve adult horses (mean±s.d. 19±3 yrs) pre‐classified as ID (*n* = 6) or non‐ID (NID; *n* = 6) using oral sugar tests

**Methods:** Horses were housed on pasture in late summer. Blood was collected every 2 h for 24 h and analyzed for insulin (Tosoh AIA) and total adiponectin (turbidimetric immunoassay). Data were analyzed non‐parametrically.

**Results:** Pasture non‐structural carbohydrate (NSC) varied from 7.5–15.4% (dry matter basis). The median [interquartile range] total adiponectin (μg/mL) was higher in NID (10.5 [6.7–13]) compared to ID (4.8 [3.9–6.5]) horses (*P* < 0.001) and varied minimally across the day (median [IQR] within‐horse total range 0.8 [0.7–1] for NID and 0.6 [0.5–0.8] for ID; within‐horse coefficient of variation 2.6%[2–3.6] for NID and 3.5% [2.6–5.9] for ID). In contrast, insulin (μIU/mL) varied widely over the day (median [IQR] within‐horse total range 16 [3.6–19.7] for NID and 168 [122–235] for ID; within‐horse coefficient of variation 17% [6.9–18.8] for NID and 32.3% [26.6–41.4] for ID). Initial adiponectin concentration negatively correlated with peak insulin (r = −0.70 [95% CI −0.91 to −0.17]; *P* = 0.02).

**Conclusions and Clinical Importance:** Factors causing variation in insulin concentrations in grazing horses in late summer had minimal effects on total adiponectin. The effect of pastures with higher NSC contents warrants evaluation.

## 68 ABSTRACT E28: Poor Air Quality in Texas Is Associated With Asthma Phenotype in Horses

### 68.1 **Laszlo Hunyadi** ^1^, DACVIM (LAIM); Sarah Cate Hyde^2^; Emily Sundman^2^, DABVP (Equine Practice)

#### 68.1.1 ^1^Associate Professor of Medicine, School of Veterinary Medicine, Texas Tech University; ^2^School of Veterinary Medicine, Texas Tech University

**Background:** Poor air quality has been reported repeatedly in equine facilities, including in stalls, stables, and indoor arenas. A substantial risk factor for the development of asthma in humans is poor air quality (AQ). To date limited information is available on the association between AQ and equine asthma.

**Hypothesis:** There is an association in horses between poor AQ and the prevalence of specific asthma phenotypes.

**Animals:** A total of 52 horses in Texas with clinical signs or history consistent with equine asthma were enrolled at four sites.

**Methods:** This study was a multi‐site, cohort study in horses with suspected asthma. Diagnosis of asthma was based on BALF cytology. A DustTrak Aerosol Monitor measured size segregated mass fraction concentrations at the horse's location. Particulate matter (PM) 2.5 results were converted to air quality index (AQI) measurements for four to five continuous days.

**Results:** One site had good AQI, and one site had AQI that was moderate to unhealthy for sensitive groups for the evaluation period. Two sites had AQIs in the unhealthy range for three of the evaluation days. A statistical difference by Chi‐square analysis for asthma phenotype by AQI was identified, with an overrepresentation of mastocytic asthma at sites with unhealthy AQI. Neutrophilic asthma was only identified at sites with unhealthy AQI.

**Conclusion and Clinical Importance:** The association between the occurrence of equine asthma and poor air quality is consistent with results found in humans. Veterinarians should provide housing recommendations for asthmatic horses when air quality is poor.

## 69 ABSTRACT E29: Comparison of Equine Antimicrobial Susceptibility Based on Minimum Inhibitory Concentration and Pharmacokinetic‐Pharmacodynamic Indices for Enrofloxacin

### 69.1 **Gustavo Ferlini Agne, Sr**.^1^, MV, MS, DACVIM (LAIM); Kandarp Patel^2^, BVSc&AH, PGDiplVPH, PhD (Veterinary Epidemiology); Nathan Slovis^3^, DVM, DACVIM, CHT; Darren Trott^4^, BSc (Hon), BVMS (Hon), PhD

#### 69.1.1 ^1^Senior Lecturer, Large Animal Internal Medicine, University of Adelaide; ^2^Senior Lecturer, Veterinary Public Health, Pathobiology, University of Adelaide; ^3^Director, McGee Critical Care and Medical Center, Hagyard Equine Medical Institute; ^4^Professor of Veterinary Microbiology, University of Adelaide

**Background:** Culture & susceptibility (C&S) and minimum inhibitory concentration (MIC) testing commonly guide antimicrobial selection, but incorporating pharmacokinetic‐pharmacodynamic (PK‐PD) indices better predicts *in vivo* efficacy. For fluoroquinolones, optimal bactericidal activity occurs at an area under the curve (AUC):MIC ratio ≥ 125 or maximum (peak) plasma concentration (Cmax):MIC ratio ≥ 10, enhancing bacterial killing and reducing resistance. Despite this, MIC‐based C&S results frequently guide treatment without PK‐PD consideration.

**Hypothesis/Objectives:** This study aimed to assess agreement between MIC‐based and PK‐PD‐derived susceptibility across enrofloxacin doses and routes. We hypothesized MIC‐based classifications would poorly align with PK‐PD predictions.

**Methods:** C&S and MIC results from clinical records were retrospectively reviewed. PK‐PD‐derived susceptibility was assessed using AUC and Cmax values from published enrofloxacin PK studies in adult horses. Agreement between MIC‐based and PK‐PD‐derived susceptibility was evaluated using descriptive and Cohen's kappa statistics.

**Results:** C&S testing classified 461 isolates (90.2%) as susceptible and 50 (9.8%) as resistant to enrofloxacin. Agreement between MIC‐based and PK‐PD‐derived susceptibility varied by dose and route. At 2.5 mg/kg, intragastric (IG) administration showed 0.11 agreement, while intravenous (IV) reached 0.36. At 5 mg/kg, IV agreement ranged from 0.25 to 1. Intramuscular administration achieved 0.64, and IG 0.82. At 7.5 mg/kg, the recommended per oral (PO) dose, agreement was 0.36.

**Conclusions and Clinical Importance:** MIC‐based susceptibility showed variable agreement with PK‐PD derived susceptibility, with only fair agreement for the recommended PO dose. Integrating PK‐PD indices for treating bacterial infections is crucial for optimizing efficacy and minimizing resistance against enrofloxacin in equine practice.

## 70 ABSTRACT E30: Prevalence of Insulin Dysregulation in Sport Horses as Determined by Traditional and Stall‐Side Testing

### 70.1 **Kimberly Hallowell** ^1^, DVM, DACVIM (LAIM); Katarzyna Dembek^2^, DVM, PhD, DACVIM (LAIM); Kelley Varner^3^, DVM, DACVAA; Caitlyn Horne^4^, DVM, DACVSMR; Raul Bras^5^, DVM, CJF; Andrew van Eps^6^, BVSc, PhD, DACVIM (LAIM); Lauren Schnabel^7^, DVM, PhD, DACVS, DACVSMR

#### 70.1.1 ^1^Postdoctoral Fellow, PhD Student, North Carolina State University, NC, USA; ^2^Assistant Professor, Department of Clinical Sciences, North Carolina State University, NC, USA; ^3^Clinical Veterinary Instructor, Department of Clinical Sciences, North Carolina State University, NC, USA; ^4^Assistant Clinical Professor, Molecular Biomedical Sciences, North Carolina State University, NC, USA; ^5^Department of Clinical Sciences, North Carolina State University, NC, USA; ^6^Professor, Department of Clinical Studies, School of Veterinary Medicine, University of Pennsylvania, PA, USA; ^7^Professor, Department of Clinical Sciences, North Carolina State University, NC, USA

**Background:** Insulin dysregulation (ID) is a risk factor for laminitis, but the prevalence has not been investigated in sport horses. New stall‐side assays offer convenience but need further validation.

**Objectives:** To determine the prevalence of ID and in sport horses, identify parameters associated with disease, and evaluate a stall‐side insulin assay.

**Animals:** To date, 50 sport horses presenting for orthopedic evaluation with no prior history of confirmed insulin dysregulation (additional 150 to be tested).

**Methods:** Cross‐sectional observational study. Signalment and morphometric data including body condition and cresty neck scores were collected. Insulin, leptin, and total adiponectin were measured at baseline. Horses were administered 0.15 mL/kg light karo syrup and insulin measurements repeated after 60 and 90 min. Stall‐side (Wellness Ready) insulin measurements were compared to a commercial laboratory (Tosoh Immunoassay). Simple logistic regression was used to identify predictors of ID. Agreement between insulin results was assessed with simple linear regression and a Bland‐Altman plot.

**Results:** No horses had increased basal insulin, but ID was confirmed in 12/50 (24%) with the oral sugar test. Univariate logistic regression identified no parameters associated with a diagnosis of ID. Simple linear regression showed significant correlation between tests (R^2^=0.64, *P* < 0.0001). Stall‐side insulin concentrations averaged 14.3 μU/mL higher than the laboratory (95% LoA −2.9 to 31.5 μU/mL).

**Conclusions and Clinical Importance:** ID is common in sport horses and dynamic insulin testing is necessary for diagnosis. Agreement between the stall‐side assay and laboratory was fair and warrants further evaluation.

**Figure:**
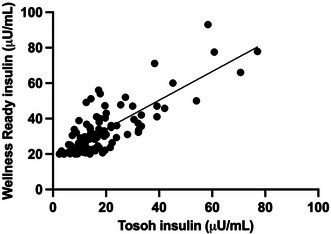
**Figure 1. Linear regression to assess correlation of equine insulin concentrations (*n* = 50) analyzed with a lateral flow assay (Wellness Ready) using whole blood and a Tosoh immunoassay using serum**. Insulin values were significantly correlated between assays (R^2^=0.64, *P* < 0.0001).

**Figure:**
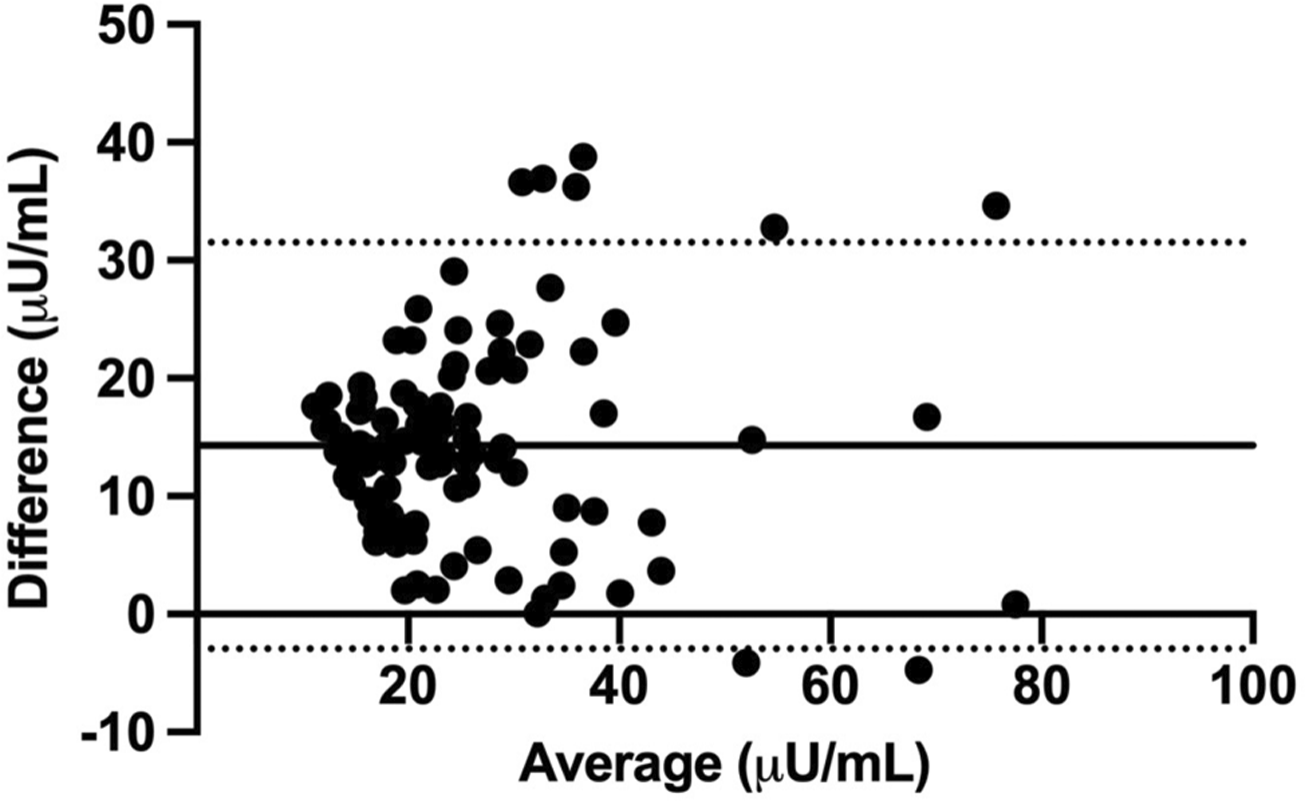
**Figure 2. Bland‐Altman plot of the difference between equine insulin concentrations for 50 paired samples determined with a lateral flow assay (Wellness Ready) using whole blood and Tosoh immunoassay using serum**. Solid black line represents mean bias (14.3 μU/mL) and black dashed lines indicate 95% limit of agreement (−2.9 to 31.5 μU/mL).

## 71 ABSTRACT E31: Case Series of Nutritional Secondary Hyperparathyroidism in Horses in Hawaii

### 71.1 **Emily Sundman** ^1^, DVM, DABVP (Equine Practice); Laszlo Hunyadi^2^, DACVIM (LAIM)

#### 71.1.1 ^1^Adjunct Professor, School of Veterinary Medicine, Texas Tech University; ^2^Associate Professor of Medicine, School of Veterinary Medicine, Texas Tech University

**Background:** Equine nutritional secondary hyperparathyroidism (NSHP, bighead disease) is a consequence of diets with abnormal calcium:phosphorus ratios. With the advent of fortified feeds and availability of legume forage, the disease has largely disappeared. The Hawaiian Islands are unique in that legume forage is unavailable and pastures have high oxalate concentrations.

**Objective:** This case series reported on the use of calcium supplementation in horses diagnosed with NSPH.

**Animals:** Seventeen adult horses imported from the continental U.S. to the Waiki’i region, Hawai’i that grazed on pastures with known oxalate‐rich kikuyu grass.

**Methods:** This was a cohort study. Plasma ionized calcium and parathyroid hormone concentrations at baseline and after eight months were evaluated. Purina® Outlast® was fed to Group 1 (eight horses) and Leg Up® Stomach pellets was fed to Group 2 (five horses) as calcium supplements.

**Results:** At screening 8/17 horses were normal and 9/17 horses were diagnosed with NSHP. In Group 1, 1 NSHP horse resolved, 4 NSHP horses remained affected, 2 non‐NSHP horses remained unaffected, and 1 non‐NSHP horse developed NSHP. In Group 2, 2 NSHP horses resolved, 1 NSHP horse remained affected, and 2 non‐NSHP horses remained unaffected. A statistically significant difference between treatment cohorts was found in post‐treatment ionized calcium (*p* value=0.0063) and parathyroid hormone (*p* value=0.0236) concentrations.

**Conclusions and Clinical Importance:** The results of this study demonstrate that NSHP remains a common disease in regions without consistent access to fortified feeds and with high oxalate grasses. Calcium supplementation may be effective in some horses, but continued monitoring is required.

## 72 ABSTRACT E32: Echocardiography in Hypoxemic Neonatal Foals: Preliminary Results

### 72.1 **Francesca Freccero** ^1^, DVM, PhD, DECEIM; Fernanda Timbó D’el Rey Dantas^2^, DVM, MSc; Elisa Celegato^3^; Giovanni Romito^4^, DVM, SMIPPV, MSc, PhD, DECVIM‐CA (Cardiology); Carolina Castagnetti^5^, DVM, PhD, DECAR; Aliai Lanci^6^, DVM, PhD; Jole Mariella^7^, DVM, PhD; Aurora Mannini^2^, DVM

#### 72.1.1 ^1^Associate Professor, Università di Bologna, Italy; ^2^PhD candidate, Dipartimento di Scienze Mediche Veterinarie, Università di Bologna, Italy; ^3^Undergraduate Student, Dipartimento di Scienze Mediche Veterinarie, Università di Bologna, Italy; ^4^Senior Assistant Professor, Università di Bologna, Italy; ^5^Full Professor, Dipartimento di Scienze Mediche Veterinarie, Università di Bologna, Italy; ^6^Junior Researcher, Università di Bologna, Italy; ^7^Associate Professor, Dipartimento di Scienze Mediche Veterinarie, Università di Bologna, Italy

**Background:** Hypoxemic newborn infants often show echocardiographic abnormalities. Regrettably, no echocardiographic data exists in hypoxemic neonatal foals (HYPO).

**Hypothesis/Objectives:** To compare selected echocardiographic parameters between HYPO, sick non‐hypoxemic (SICK), and healthy neonatal foals (HEALTHY).

**Animals:** 35 foals ≤5 days (12 HYPO, 8 SICK, and 15 HEALTHY)

**Methods:** Retrospective study on foals examined via transthoracic echocardiography within the first 5 days of life. A right parasternal short‐axis view was used to measure left ventricular diameters horizontally (LVD1) and vertically (LVD2) in diastole (LVD1_d_, LVD2_d_) and systole (LVD1_s_, LVD2_s_). Diastolic and systolic eccentricity indices were calculated from these diameters. Aortic diameters at the Valsalva sinus (AoD_sinus_) and sinotubular junction (AoD_STJ_) were measured in diastole from the left ventricular outflow tract view. Pulmonary artery systolic and diastolic diameters (PAD_d_, PAD_s_), pulmonary artery distensibility index, AoD_sinus_:PAD_d_, and AoD_STJ_:PAD_d_ ratios were also calculated. Presence of tricuspid regurgitation was noted. Parameters were compared using the Kruskal‐Wallis test with Bonferroni correction.

**Results:** Age and body weight showed no significant differences between groups. HEALTHY had larger LVD1_d_ than HYPO and SICK, and larger LVD2_d_ than SICK. Additionally, SICK had smaller AoD_sinus_ than HEALTHY. No other statistically significant differences were observed. Only two HEALTHY and one HYPO showed tricuspid regurgitation.

**Conclusions and Clinical Importance:** Echocardiographic signs of left ventricular volume depletion were observed in SICK and HYPO, with no significant differences between these two groups. Further studies with larger sample sizes and additional echocardiographic parameters, including those assessing right heart dimensions and function, are needed to better characterize HYPO.

**Table:** **Table 1.** Echocardiographic parameters in hypoxemic (HYPO), sick non‐hypoxemic (SICK), and healthy (HEALTHY) neonatal foals

Variable	Unit	HYPO	SICK	HEALTHY
pO_2_	mmHg	50.6 [40.3–55.8]^A^	73.2 [71.5–75.3]^B^	58.2–84.3*
Age	h	29 [6–102]	15 [9.25–21.5]	20 [17–23]
BW	kg	44 [42.5–44.5]	39 [37–45]	48 [44–49]
LVD1_d_	cm	5.05 [4.2–5.25]^A^	4.8 [4.3–5.3]^A^	5.5 [5.25–5.7]^B^
LVD2_d_	cm	4.5 [3.7–5.3]^AB^	4.25 [4.05–4.95]^AB^	5.3 [5.05–5.45]^A^
EI_d_		1.1 [1.05–1.15]	1.1 [1–1.15]	1.0 [1–1.1]
LVD1_s_	cm	2.9 [2.25–3.3]	2.85 [2.2–3.2]	3.0 [2.9–3.3]
LVD2_s_	cm	2.75 [1.95–3.4]	2.75 [2.05–3.3]	3.2 [2.95–3.4]
EI_s_		1.0 [0.9–1.05]	1.0 [0.95–1.15]	1.0 [0.9–1]
AoD_sinus_	cm	2.8 [2.7–3.15]^AB^	2.75 [2.55–2.9]^AB^	3.1 [2.95–3.35]^A^
AoD_STJ_	cm	2.05 [1.9–2.25]	1.75 [1.65–2.15]	2.1 [1.9–2.35]
PAD_d_	cm	1.8 [1.45–1.95]	1.6 [1.4–1.8]	1.7 [1.55–1.9]
PAD_s_	cm	2.4 [2–2.5]	2.05 [1.9–2.25]	2.2 [2.1–2.4]
PADI	%	26.15 [16.6–32.5]	22.3 [17.5–29.2]	21.8 [16.75–28.8]
AoD_sinus_/PAD_d_		1.6 [1.45–1.95]	1.7 [1.6–1.85]	1.9 [1.6–2.1]
AoD_STJ_/PAD_d_		1.15 [1–1.55]	1.25 [0.95–1.35]	1.3 [1.05–1.45]

Data are expressed as median [interquartile range].

Different superscript letters (A, B) in row indicate differences (*P* < 0.05) among groups after Bonferroni correction.

BW: body weight; EI: eccentricity index; PADI: pulmonary artery distensibility index

* Mean values for foals aged from 60 min to 4 days. From Wilkins PA, Otto CM, Baumgardner JE, et al. Acute lung injury and acute respiratory distress syndromes in veterinary medicine: consensus definitions: the Dorothy Russell Havemeyer Working Group on ALI and ARDS in veterinary medicine. *J Vet Emerg Crit Care*. 2007;17:333–339.

## 73 ABSTRACT E33: Comorbidities Associated With a Diagnosis of Pituitary Pars Intermedia Dysfunction in Primary Care Practice

### 73.1 **Emma Stapley** ^1^, DVM, DACVIM; Amanda Farr^2^, DVM, DABVP; Caroline Gillespie Harmon^2^, DVM, MS, DACVS (LA); Sarah Waxman^2^, DVM, MS, DACVS (LA); François‐René Bertin^3^, DVM, MS, PhD, FHEA, DACVIM (LAIM)

#### 73.1.1 ^1^PhD Student, Purdue University, IN, USA; ^2^Clinical Assistant Professor, Equine Community Practice, Purdue University, IN, USA; ^3^Associate Professor, Department of Veterinary Clinical Sciences, Purdue University, IN, USA

**Background:** Pituitary pars intermedia dysfunction (PPID) and Parkinson's disease (PD) are both alpha‐synucleinopathies linked to aging. However, comorbidities common to both conditions, independent of age, have not been studied.

**Objectives:** Identify comorbidities associated with PPID, independent of age, in a first‐opinion veterinary practice.

**Animals:** Medical records from 132 horses with PPID and 274 controls matched by age and breed

**Methods:** Variables associated with a diagnosis of PPID were evaluated with a Fischer exact test or Mann‐Whitney test depending on type. A backward stepwise model was then used with *p* < 0.2 to be considered for inclusion and *p* < 0.05 to be retained in the final multivariable logistic regression.

**Results:** Variables associated with a diagnosis of PPID included boarding at a boarding facility (*p* = 0.02), a high fecal egg count (*p* = 0.03), presence of hyperinsulinaemia‐associated laminitis (HAL, *p* < 0.0001), more common use of non‐steroidal anti‐inflammatory drugs (NSAID, *p* = 0.006), more common development of neurologic diseases (*p* = 0.03) and having more adverse medical events (7 [0–25] vs. 5 [0–47], *p* = 0.01). The results of the logistic regression indicated that boarding at a boarding facility, poor wound healing, HAL and use of NSAID were independent factors associated with a diagnosis of PPID.

**Clinical Importance:** Although age is the main risk factor for the development of PPID, additional factors seem associated with a diagnosis of the disease. As described in people with PD, chronic inflammation appears common in horses with synucleinopathies.

## 74 ABSTRACT E34: Aerosolized Equid Alphaherpesvirus‐1 and ‐4 Viral Particles Detection at an Equine Care Facility

### 74.1 **Amjad Khan, Sr**.^1^, DVM, MPhil, PhD; Edward Olajide^2^; Emma Hyde^2^; Nicolas Ciraulo^3^; Juliette Brun^4^; Lutz Goehring^5^, DVM, MS, PhD

#### 74.1.1 ^1^Postdoctoral Scholar for Infectious Diseases, University of Kentucky, KY, USA; ^2^Veterinary Science, University of Kentucky, KY, USA; ^3^Lincoln Memorial University, TN, USA; ^4^University of Rennes, France; ^5^Wright‐Markey Professor of Equine Infectious Diseases, Veterinary Science, University of Kentucky, KY, USA

**Background:** Recent outbreaks of equid alphaherpesvirus 1 (EHV‐1) in equine care facilities underscore significant gaps in infection control. Innovative surveillance strategies are needed for proactive surveillance.

**Objectives:** To evaluate a novel community surveillance approach for the detection of aerosolized EHV‐1 in equine care facilities.

**Animals:** There was no direct horse contact.

**Methods:** Observational study conducted at an equine care facility in Kentucky for 4 weeks (Summer 2024). We used a Bertin™ Coriolis compact for continuous air sampling at 200 L/min 6 hrs in a treatment area (daytime activity) and during nights in an occupied barn. As references, we sampled individual surfaces in close contact with the heads of horses once daily. Samples were analyzed using quantitative PCR.

**Results:** We collected equal numbers of 84 surface and 12 air samples from either the treatment (end of day) or the barn (mornings). In the treatment area, 2 and 5 air samples were found positive for EHV‐1 or ‐4, respectively. 5 EHV‐4 positive surfaces, and no EHV‐1 was detected. In the barn area, 4 and 6 air samples were positive for either EHV‐1 or ‐4, and 0 and 6 surfaces were positive for EHV‐1 and EHV‐4. Overall, the air sampling detection rate was significantly higher at night‐time sampling in stalls as compared to daytime in treatment areas with low‐scale surface contamination in both areas which could be related to density of population occupancy at night time.

**Conclusion:** Air sampling demonstrated a good detection rate for EHVs, highlighting its potential as a community monitoring tool in equine care facilities.

## 75 ABSTRACT E35: Preliminary Evaluation of an Immunoturbidometric Assay for Measurement of Total Adiponectin Concentration in Equids

### 75.1 **Daniela Luethy** ^1^, DVM, DACVIM (LAIM); Rose Nolen‐Walston^2^; Caitrin Lowndes^2^; Jeaneen Kulp^2^; Andrew Van Eps^2^

#### 75.1.1 ^1^Assistant Professor, Large Animal Medicine, New Bolton Center, University of Pennsylvania, PA, USA; ^2^New Bolton Center, University of Pennsylvania, PA, USA

**Background:** Total adiponectin is a marker of insulin dysregulation and laminitis risk. The reliability of the currently available immunoturbidometric total adiponectin (TA) assay, as well as natural TA variation, has not been established in equids.

**Hypothesis/Objectives:** 1) Determine markers of analytic validity for the TA assay; 2) Assess correlation between TA and high molecular weight adiponectin (HMWA); 3) Investigate variability of TA throughout the course of a day.

**Animals:** Serum samples from 207 client‐ and university‐owned mixed breed horses, ponies, and donkeys with and without insulin dysregulation.

**Methods:** Total adiponectin concentrations were determined using a turbidimetric immunoassay. Intra‐ and inter‐assay variability and recovery upon dilution were assessed. HMW adiponectin was measured using a previously‐validated ELISA. Spearman correlation was performed to assess the relationship between TA and HMWA. TA was measured every 2 h in 5 horses over a 24‐h period.

**Results:** Mean (range) intra‐assay coefficient of variation (CV%) was 2.4% (1.1–5.6%), and inter‐assay CV% was 16.3% (11.3–21.8%). Recovery upon dilution was 121.9% (75–140.6%). A strong correlation between TA and HMWA was observed (Spearman's ρ=0.90 [95% CI: 0.86–0.92], *p* < 0.001). Mean (range) within‐horse CV% for TA over 24 h was 10.4% (5.6–22.6%).

**Conclusion and Clinical Importance:** The turbidimetric immunoassay was sufficiently precise and reliable for measurement of TA in equids. TA correlated well with HMWA in this population. The variation in TA over 24 h in individual horses was likely insufficient to affect clinical interpretation, although more research in this area is warranted.

## 76 ABSTRACT E36: Fecal and Intestinal Microbiota Profiles in Horses With Different Types of Surgical Colic

### 76.1 **Marcio Costa** ^1^, DVM, DVSc, PhD; Julia Arantes^2^; Marília Ferreira^2^; Pedro Henrique Brito^2^; Caio Bustamante^2^; Camila Fernandes^3^; Carlos Augusto Valadão^3^; Renata Dória^2^

#### 76.1.1 ^1^Associate Professor, University of Montreal; ^2^Faculty of Animal Science and Food Engineering, University of São Paulo; ^3^School of Agricultural and Veterinary Studies, São Paulo State University

**Background:** Various factors influence the equine intestinal microbiota, including colic.

**Objectives:** To evaluate the fecal and intestinal microbiota of horses with colic undergoing exploratory celiotomy.

**Animals:** Thirty‐five horses with colic, categorized by lesion type: large colon volvulus (*n* = 11), displacement (*n* = 11), impaction (*n* = 6); small intestine volvulus (*n* = 5) and obstruction (*n* = 2). Twelve healthy horses from the same farms, sampled six months post‐surgery, served as controls.

**Methods:** Fecal samples were collected at admission and on days 1, 2, 3, and 6 months post‐surgery. Intestinal content was obtained from the duodenum, cecum and colon during surgery. Microbiota composition was analyzed via 16S rRNA sequencing (V4 region). ANOVA and PERMANOVA tests (*p* < 0.05) determined statistical significance.

**Results:** Fecal samples differed significantly from intestinal contents and between sampling days. Feces collected at D0 had lower richness in horses with small intestinal or strangulating lesions. Colic duration (> 24 vs. ≤24 h) also influenced fecal microbiota composition at D0. Several markers were associated with feces at D0 (e.g. *Escherichia* and *Akkermansia*) compared to healthy controls (e.g. Lachnospiraceae and *Fibrobacter*). By six months post‐surgery, fecal microbiota composition was indistinguishable from healthy controls, indicating microbiota recovery. In intestinal contents, microbiota richness, diversity, and composition varied by intestinal segment and lesion type.

**Conclusions and Clinical Importance:** The differences between fecal and intestinal content highlight the uniqueness of each segment during colic episodes. Future studies identifying microbial markers for specific colic types may enhance diagnosis and improve clinical decision‐making.

## 77 ABSTRACT E37: Evaluation of Two Immunochromatographic Point‐of‐Care Tests for Detection of *Coccidioides* Antibodies in Horses

### 77.1 **Ernesto Rojas‐Sanchez** ^1^, DVM, MPVM; Jane Sykes^2^, BVSc, PhD, MBA, GCPH, DACVIM (SAIM); Margaret Brosnahan^3^, DVM, PhD, DACVIM (LAIM); Rana Bozorgmanesh^2^, DVM, DACVIM (LAIM); Allen Bryan^2^, MD, PhD; Kelly Crucillo^2^, MS; Alina Dhalla^2^; Jennifer Cassano^2^, DVM, PhD

#### 77.1.1 ^1^PhD Epidemiology Student, University of California–Davis, Davis, CA, USA; ^2^University of California–Davis, Davis, CA, USA; ^3^Midwestern University, Glendale, AZ, USA

**Background:** Coccidioidomycosis (valley fever [VF]) is a life‐threatening and emerging disease of humans and animals. Currently available serologic tests (agar gel immunodiffusion [AGID]) take days to perform and require experience to perform and interpret. Validation of novel lateral flow assays (LFAs) for horses will allow for more rapid diagnosis and earlier treatment, yielding improved outcomes.

**Objectives:** To compare results of two novel lateral flow assays (LFAs) to AGID for detection of serum anti‐*Coccidioides* antibodies in horses.

**Animals:** Serum specimens from horses tested using AGID at the University of California–Davis Coccidioidomycosis Serology Laboratory (UCD‐CSL) from December 2024 to June 2025 (target 20 seropositive horses and 20 seronegative horses).

**Methods:** Serum specimens were tested using sōna *Coccidioides* antibody LFA (IMMY Diagnostics) and a recombinant chitinase‐1 LFA (Velox, Cactus Bio) according to manufacturers’ instructions. Investigators interpreting assay results were masked to AGID results. Assay agreement was determined using a kappa comparison. Sensitivity and specificity for each LFA were determined using AGID as the gold standard reference.

**Results:** For 14 specimens tested at the time of writing (Figure 1), results for the sōna LFA have agreed perfectly with AGID (kappa value 1.00, 100% agreement). The Velox LFA showed strong agreement (0.72, 85.7%). The sōna LFA had a 100% sensitivity and specificity. The Velox LFA had a 100% sensitivity and 75% specificity (Figure 1).

**Conclusions and Clinical Importance:** Preliminary data suggest that these LFAs may expedite diagnosis of equine coccidioidomycosis.

**Figure:**
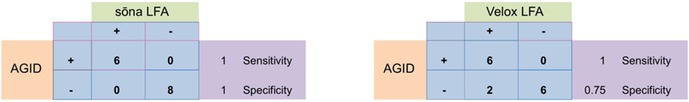
Figure 1. Evaluation of the results from sōna *Coccidioides* antibody LFA (IMMY diagnostics) and a recombinant chitinase 1 LFA (Velox, Cactus Bio) immunochromatographic point‐of‐care tests in comparison to the results from the agar gel immunodiffusion (AGID) test for detection of *Coccidioides* antigen in horses

## 78 ABSTRACT E38: The Role of Adrenal Activation in the Pathogenesis of Equine Metabolic Syndrome

### 78.1 **Lara Jimenez** ^1^, DVM, AFHEA, MRCVS; Morgan Ruth^2^, MA, VetMB, PhD, ECEIM, MRCVS; Tobias Warnken^3^, DVM, PhD, DECEIM, EBVS; Johanna Sonntag^4^, PhD; Scott Denham^5^; Natalie Homer^6^, PhD, FIBMS

#### 78.1.1 ^1^Equine Intern, University of Edinburgh; ^2^Lecturer, Department of Animal and Veterinary Sciences, Royal (Dick) School of Veterinary Science, Scotland's Rural College, Easter Bush Campus, University of Edinburgh; ^3^Senior Pipeline and Portfolio Strategy Manager Equine, Boehringer Ingelheim Vetmedica GmbH; ^4^Senior Scientist NID Research, Boehringer Ingelheim Vetmedica GmbH; ^5^Senior Bioanalytical Specialist/Deputy Core Manager, Mass Spectrometry Core, Edinburgh Clinical Research Facility, Queen's Medical Research Institute, University of Edinburgh; ^6^Senior Research Fellow, Mass Spectrometry Core, Edinburgh Clinical Research Facility, Queen's Medical Research Institute, University of Edinburgh

**Background:** In human obesity and insulin dysregulation (ID), adrenal activation often exacerbates metabolic dysfunction. However, limited information exists on equine obesity or equine metabolic syndrome (EMS).

**Hypothesis:** Obesity is associated with adrenal activation and EMS horses show an amplified adrenal response to an oral glycaemic challenge test.

**Animals:** Serum samples from a prior multicentred prospective case‐control study in the UK, Germany, and Sweden comprised of 31 healthy and 40 EMS‐diagnosed horses, both university and privately‐owned were utilized.

**Methods:** Serum was analysed by LC‐MS/MS for quantification of 18 steroids, pre and post the glycaemic test. Multivariate regression models and two‐way ANOVAs determined the relationship between steroids and obesity/EMS, and the effect of obesity/EMS on response to the test.

**Results:** The final model best predicting obesity (BCS > 6/9) was increased cortisol and decreased corticosterone and cortisone (*P* = 0.02, NPP 69.7%, PPV 70.83%). Decreased aldosterone was predictive of EMS (*P* = 0.01, NPV 66.7, PPV 80%). No good predictors of regional adiposity were identified. The glycaemic challenge test increased progesterone (*P* = 0.003) and decreased cortisone in all animals. Obesity was associated with an increased 11‐deoxycortisol response to glucose (*P* = 0.04). EMS animals showed a significant increase in cortisol (*P* = 0.02), corticosterone (*P* = 0.04), 11‐deoxycortisol (*P* = 0.03), and 11‐deoxycorticosterone (*P* = 0.03) compared to healthy animals.

**Conclusions:** The study concludes that obesity is linked with adrenal activation, with a compensatory change in cortisol to cortisone metabolism. EMS results in an exaggerated glucocorticoid response to a glycaemic challenge, both raising pathophysiological and clinical questions for further investigation.

## 79 ABSTRACT E39: The Response of Cathepsin B to Exercise and Training in Thoroughbred Racehorses

### 79.1 **Lisa Katz** ^1^, DVM, MS, PhD, DACVIM, DECEIM, DECVSMR; Gina Duggan^2^; Amy Byrne^2^; Emmeline Hill^2^, PhD; Alan Baird^2^, PhD

#### 79.1.1 ^1^Professor, Equine Internal Medicine, University College Dublin; ^2^University College Dublin

**Background:** Exercise positively impacts brain health. Exercise‐induced peripheral humoral factors (exerkines) are believed to mediate some of the effects due to ‘muscle‐brain crosstalk’. Cathepsin B (CTSB) is a muscle‐derived exerkine capable of crossing the blood‐brain barrier. In humans peripheral CTSB is associated with improved cognitive function. Most report CTSB to increase after exercise and training, although some found no change or a decrease in CTSB after training. This has not been investigated in horses.

**Hypothesis:** Peripheral CTSB increases following exercise and training in horses.

**Animals:** Privately‐owned 2‐year‐old Thoroughbred racehorses (*n* = 17) from one yard.

**Methods:** A prospective cohort study was performed. Horses were instrumented with an ECG‐GPS device. Venous blood was collected before (T0), 5 (T1) and 30 min (T2) after high intensity ridden exercise on an all‐weather gallop; 10/17 horses had repeat samples taken after 6–8 weeks of training. Plasma samples were measured neat in triplicate using a Horse CTSB quantitative sandwich ELISA.

**Results:** Peak heart rate, velocity and distance were not different during the tests before and after training. Intra‐ and inter‐assay coefficient of variations were 10% and 15%. Pre‐training T1 (3.99±2.1 pg/mL) and T2 (3.5±1.6 pg/mL) CTSB concentrations were greater than T0 (3.1±1.4 pg/mL; *p* = 0.03, repeated measures ANOVA). After training, CTSB concentrations at T0 (2.8±1.1 pg/mL), T1 (3.2±1.5 pg/mL) and T2 (3.0±1.5 pg/mL) were significantly lower than pre‐training values (*p* = 0.05, repeated measures ANOVA).

**Conclusions and Clinical Importance:** Plasma CTSB concentrations increase following exercise in horses but decrease following training. Additional research is required to understand how CTSB may interact with other neurotrophic factors.

## 80 ABSTRACT E40: Complex Arrhythmias in One Exercising ECG in Thoroughbred Racehorses Are Not Associated With Lifetime Performance

### 80.1 **Hamid Tavanaeimanesh** ^1^, DVM, DVSc; Mary Durando^2^; Cristobal Navas de Solis^3^; Sian Durward‐Akhurst^4^

#### 80.1.1 ^1^Postdoctoral Associate, Department of Veterinary Clinical Sciences, University of Minnesota, MN, USA; ^2^Equine Sports Medicine Consultants; ^2^New Bolton Center, School of Veterinary Medicine, University of Pennsylvania, PA, USA; ^3^Department of Veterinary Clinical Sciences, University of Minnesota, MN, USA

**Background:** Complex arrhythmias (CA) are common in racehorses with and without poor performance making interpretation challenging.

**Hypothesis/Objectives:** To determine whether CA in one exercising ECG are associated with lifetime racing performance.

**Animals:** ECGs collected over 6 months from 75 Thoroughbreds in race‐training.

**Methods:** ECGs during and 2 min after breezing were analyzed. When ≥ 1 ECG was available/horse, the ECG with more premature complexes (PCs) was used. Starts, placing third or better, placing below third, the highest Equibase speed figure and total and average earnings per start over the lifetime of the horse were obtained from equibase.com (starts were excluded due to multicollinearity). Logistic multivariable regression (covariates: age, sex) was performed using R. Significance was *p* < 0.05.

**Results:** Mean (SD) peak heart rate was 215+7/min. 25/75 horses had CA, and 64/75 horses had ≥ 1 PC during exercise. Sex, speed, peak heart rate, and age were not significantly different between horses with and without CA (*p* > 0.1). Performance metrics analyzed were not associated with CA in one exercising ECG (*p* > 0.2).

**Conclusions and Clinical Importance:** These preliminary results suggest that CA in one exercising ECG are not associated with common lifetime performance metrics. This helps infer potential mechanistic and performance implications. For example, CA may not be associated with performance or intrinsic to the horse (instead, dependent on external factors), CA may be sporadic in an individual, or training cardiac rhythm is not representative of racing rhythm. These potential explanations remain speculative; work is ongoing to evaluate associations between arrhythmias and future/current performance, and safety.

## 81 ABSTRACT E41: Leukocyte Numbers and Morphological Changes in the Hindgut of Horses With Pituitary Pars Intermedia Dysfunction

### 81.1 **Wenqing Wang** ^1^; Viviana Astudillo^2^, BVSc, MNR, PhD, DACVP; François‐René Bertin^3^, DVM, MS, PhD, FHEA, DACVIM (LAIM); Meaghan Broman^4^, DVM, PhD, DACVP

#### 81.1.1 ^1^PhD Candidate, The University of Queensland; ^2^Senior Lecturer/Specialist Anatomic Pathologist, Veterinary Pathology, The University of Queensland; ^3^Associate Professor, Veterinary Clinical Science, Purdue University; ^4^Assistant Professor, Comparative Pathobiology, Purdue University

**Background:** Pituitary pars intermedia dysfunction (PPID) is a prevalent dopaminergic degeneration in aging horses, mirroring the pathogenesis of Parkinson's disease (PD). Despite the known association between intestinal inflammation and PD via the gut‐brain axis, this association remains unexplored in PPID.

**Objective/Hypothesis:** Horses with PPID have more severe inflammatory histopathological changes in the large intestine.

**Samples:** Archived tissue (cecum, large colon, and small colon) from horses with PPID (pituitary grades 4 or 5/5, *n* = 6–10) and age‐matched controls (pituitary grade 1 or 2/5, *n* = 6–10) were reviewed.

**Methods:** Epithelium injury, crypt morphology, mucosal fibrosis, goblet cell counts, prevalence of ciliated protozoa and Lewy bodies were evaluated. Lymphocytes and plasma cells, eosinophils, neutrophils, and macrophages in the mucosa and submucosa were quantified across 8 randomly selected fields. Differences between the two groups within an intestinal segment or layer were compared by unpaired t‐test or Mann‐Whitney test depending on distribution.

**Results:** Horses with PPID had significantly higher number of lymphocytes and plasma cells (control vs. PPID: 54 [49–72] vs. 79 [64–88], *P* = 0.01) and macrophages (5 [4–7] vs. 8 [6–11], *P* = 0.004), more frequent Lewy bodies (10% vs. 66.7%, *P* = 0.04), and higher grade of fibrosis (0 [0–0] vs. 1 [0–1], *P* = 0.01) in cecum.

**Conclusions and Clinical Importance:** Chronic intestinal inflammation is associated with PPID mirroring findings in PD and highlighting the importance of gastrointestinal health in the management for PPID horses.

## 82 ABSTRACT E42: Evaluating Equine Resting Behavior in Response to Housing Changes: Impact of Age and Adaptation Effect

### 82.1 **Lisa Zimmer** ^1^; Urlike Auer^1^; Jessika‐M.V. Cavalleri^1^; Michael Leschnik^1^; Manolis Lyrakis^2^; Johannes Schrammel^1^; Dagmar S. Trachsel^1^

#### 82.1.1 ^1^Center for Equine Health and Research, Clinical Department for Small Animals and Horses, University of Veterinary Medicine Vienna, Vienna, Austria; ^2^Platform for Bioinformatics and Biostatistics, Department of Biological Sciences and Pathobiology, University of Veterinary Medicine Vienna, Vienna, Austria

**Background:** Changes in the immediate environment of horses such as relocation may disrupt their resting behavior, potentially impacting overall well‐being. These effects have not been evaluated via electroencephalography, electrocardiography and video monitoring so far.

**Hypothesis/Objectives:** To investigate the effects of changes in housing on the sleeping behavior in horses of different age groups.

**Animals:** Ten university‐owned horses aged 5–26 years.

**Methods:** Ten horses were enrolled in a longitudinal observational, prospective, cohort study. They were selected in five pairs and equipped with an EEG mask, ECG girth and video cameras were placed in two different housing settings to monitor each pair for eight nights. In setting 1, horses were housed in a large barn with direct contact with conspecifics. In setting 2, horses were individually housed in separate stalls in sight of each other. Each horse spent four consecutive nights in each setting. Sleeping patterns and lying behavior were evaluated via multinomial or binomial logistic regression mixed models, while sleep and lying duration were evaluated via linear mixed models.

**Results:** Distinct sleeping patterns were observed between the two settings. During the first night in each setting, the lowest amount of lying behavior and total sleep was recorded. Sleep and lying behavior increased after midnight. REM sleep occurred almost exclusively when horses were lying. Young horses (≤10 years) were more likely to sleep compared to older horses (> 17 years).

**Conclusions and Clinical Importance:** Relocation causes sleep alterations in horses, particularly during the first night. A distinct difference in sleep distribution is observed between young and older horses.

**Figure:**
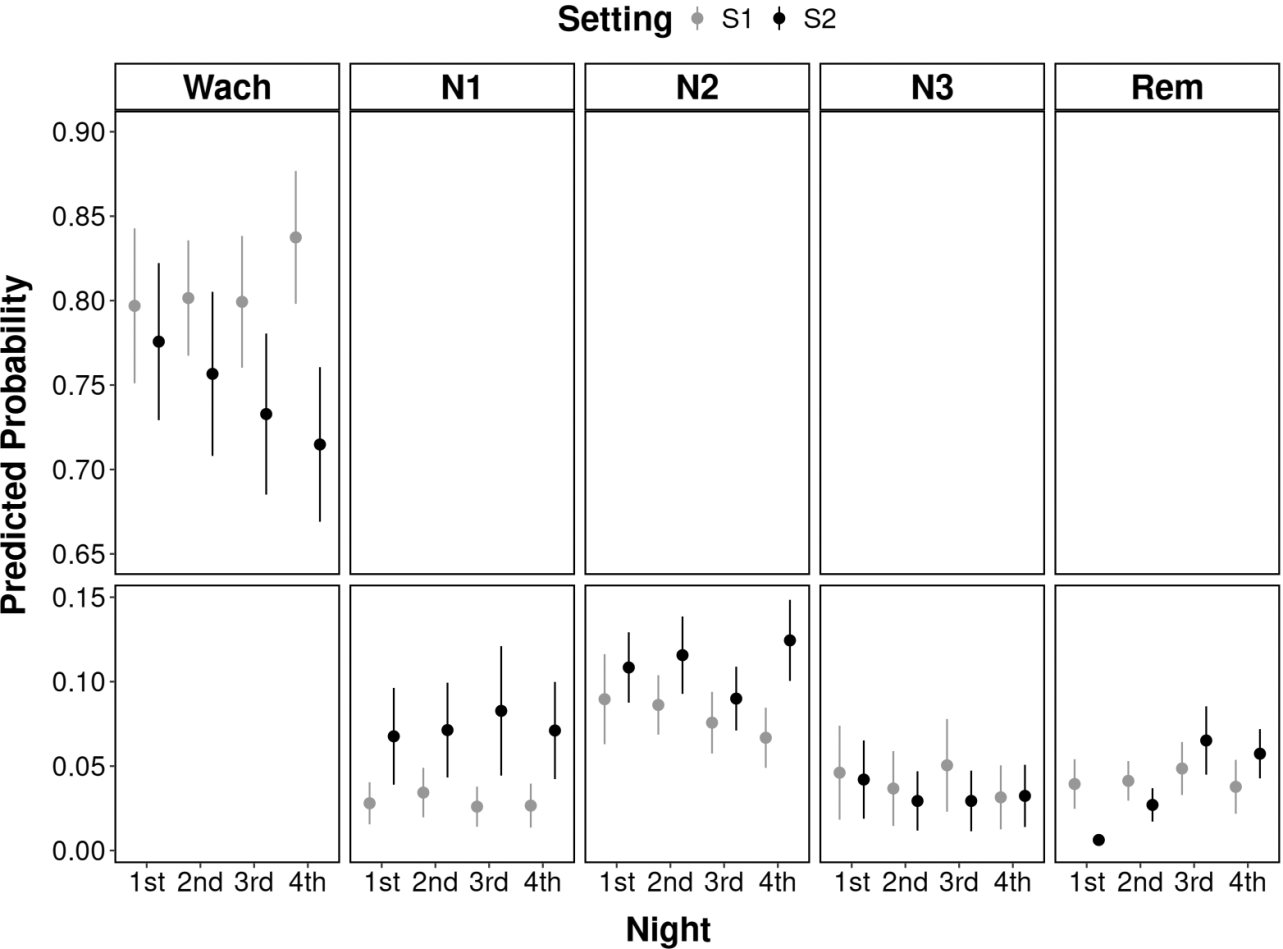
Plot 1. Probability of various sleep stages (Wach = Awake, N1, N2, N3, and REM) compared between settings (S1, S2) throughout different nights (1st–4th)

**Figure:**
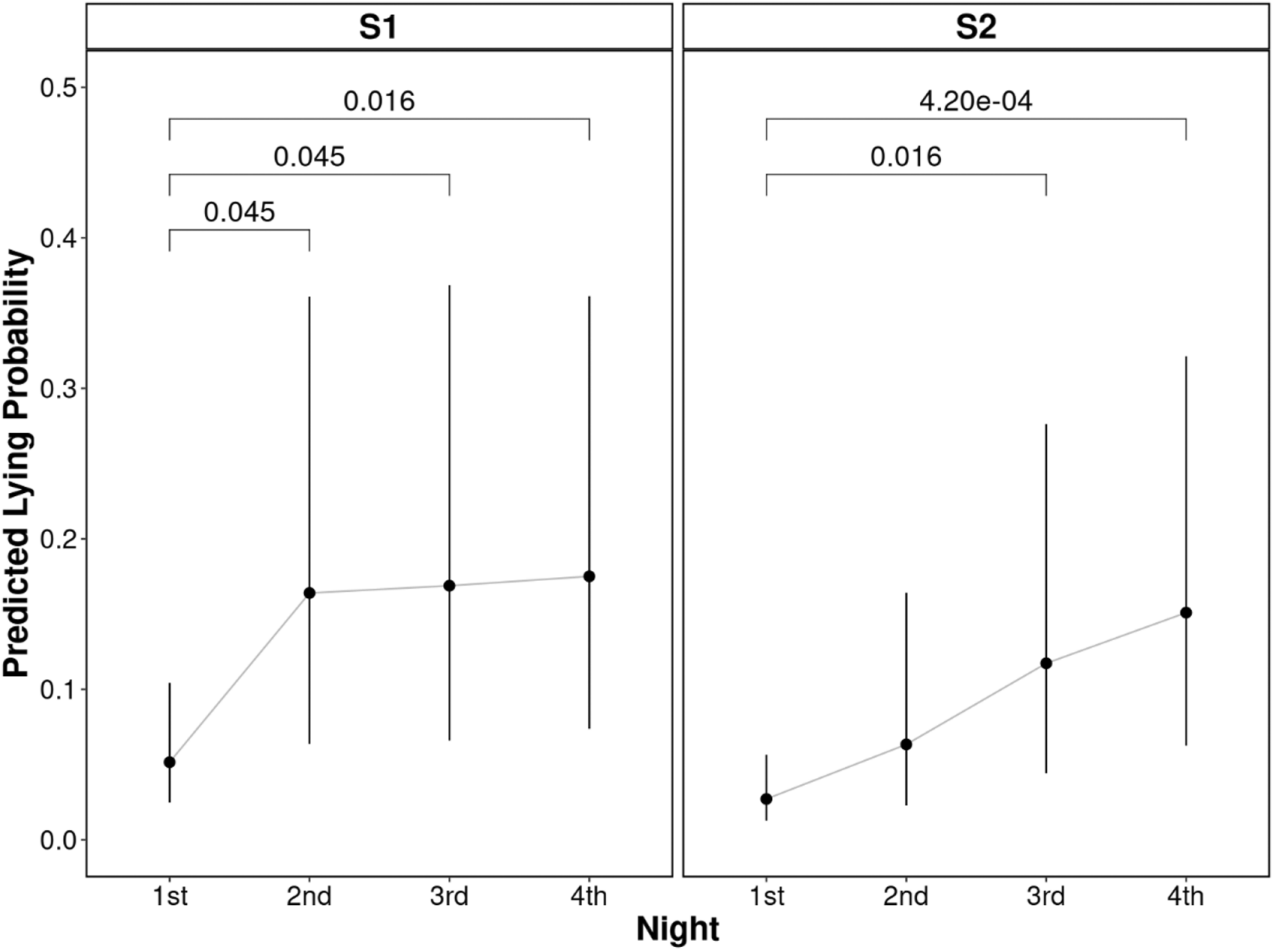
Plot 2. Lying probability in both settings (S1, S2) compared, during each night (1st–4th)

## 83 ABSTRACT E43: Pharmacokinetics of S‐Adenosylmethionine in Horses Using a Crossover Study Design With Three Dosing Regimens

### 83.1 **Linda Paul** ^1^, DVM, PhD, DACVIM (LAIM); Ethan Sutherland^2^, DVM; Carolyn Warner^2^, RVT, LVT; Rebekah Strunk^2^, MS; Robert Gillette^2^, DVM, MSE, DACVSMR; David Griffin^2^, DVM, MS

#### 83.1.1 ^1^Equine Commercial Medical Lead, Nutramax Laboratories, Inc.; ^2^Nutramax Laboratories, Inc.

**Background:** Supplementation of s‐adenosylmethionine (SAMe) can be useful in supporting liver health through augmentation of glutathione levels across many species. The pharmacokinetics of SAMe have yet to be reported in horses.

**Hypothesis/Objectives:** The objective of this study was to determine the pharmacokinetics of a proprietary formulation of SAMe (NMXSS75A; Nutramax Laboratories, Inc) in horses. The hypothesis was that plasma concentrations would be dose‐dependent and not affected by a small concentrate meal.

**Animals:** Six healthy adult mares from a research herd.

**Methods:** Crossover study design with a 7‐day washout period using three dosing regimens: S10 (10 g SAMe), S10 + FEED (10 g SAMe + 0.5 lb concentrate), and S5 (5 g SAMe). The SAMe formulation was administered via nasogastric intubation. Blood samples were collected within 1 h pre‐dose and at 0.5, 1, 2, 4, 6, 8, and 24 h post‐dose for each regimen.

**Results:** The maximum concentration (*C*_max_; *p* = 0.017), 24‐h area under the curve (AUC; *p* = 0.015) and time to maximum concentration (*T*_max_; *p* < 0.001) differed among groups. *C*_max_ (*p* = 0.017) and AUC (*p* < 0.001) were higher for S10 (*C*_max_: 1069±379 ng/mL; AUC: 3247±965 ng*h/mL) compared to S5 (619±253 ng/mL; 1460±553 ng*hr/mL). AUC (*p* < 0.001) was greater for S10 + FEED (2748±687 ng*h/mL) compared to S5. The *T*_max_ (*p* = 0.014) was prolonged for S10 + FEED (1.83±0.41 h) compared to S5 (0.92±0.20 h).

**Conclusions and Clinical Importance:** Plasma concentrations of SAMe were dose‐dependent and minimally affected by a small concentrate meal. This study provides plasma pharmacokinetic data for SAMe in horses to help guide its use in clinical practice.

## 84 ABSTRACT E44: Bronchodilatory Effect of Inhaled Lidocaine in Horses With Severe Asthma

### 84.1 **Khadija Ayadi** ^1^, DVM; Justine Herlemont^2^, DVM; Hélène Richard^2^; Sophie Mainguy‐Seers^2^, DVM, DAVCIM (LAIM), PhD; Mathilde Leclère^2^, DVM, DACVIM (LAIM), PhD

#### 84.1.1 ^1^Intern, Université de Montréal; ^2^Université de Montréal

**Background:** Inhaled lidocaine reduces bronchial hyperreactivity in humans and cats, and improves clinical scores and airway inflammation in asthmatic horses after a two‐week treatment. Its short‐term effect on bronchoconstriction in severe equine asthma (SEA) is unknown.

**Hypothesis:** Nebulized lidocaine induces transient bronchodilation.

**Animals:** Nine horses with SEA from the Equine Asthma Research Laboratory.

**Methods:** In a randomized, cross‐over study, nebulized 10% lidocaine (2 mg/kg), 0.9% saline, and salbutamol (1.6 μg/kg, metered‐dose inhaler) were administered during exacerbation. Lung function was recorded before and 5, 10, 30, and 60 min after administration. Data were analyzed using mixed‐effects models with Dunnett's corrections.

**Results:** Compared to baseline measurements, lidocaine nebulization improved pleural pressure at 10 and 30 min (from 33.6 (mean) ±31.0 (SD) to 21.8±16.6 and 18.6±18.5 cm H_2_O, *p* < 0.03) and pulmonary elastance at 30 min (from 3.4±3.9 to 1.6±1.9 cm H_2_O/L, *p* < 0.03), but did not affect resistance. Salbutamol improved lung function at 5, 10, and 30 min (pleural pressure: from 37.3±30.8 to 15.9±11.7, 12.3±4.4 and 12.8±4.4 cm H_2_O; resistance: from 2.3±1.1, to 1.0±0.3, 0.8±0.2 and 0.9±0.2 cm H_2_O/L/s; elastance: from 3.1±2.8 to 1.1±0.7, 0.9±0.5 and 1.1±0.5 cm H_2_O/L, *p* < 0.03). Saline had no significant effect.

**Conclusion and Clinical Importance:** Nebulized lidocaine induces transient bronchodilation, although to a lesser extent than salbutamol. The rapidity of the bronchodilation suggests that it is not solely due to the anti‐inflammatory properties reported with its prolonged use.

## 85 ABSTRACT E45: Detection of Biomarkers Indicators of Intestinal Health Among the Equine Fecal Microbiota Using Quantitative PCR

### 85.1 **Marcio Costa** ^1^, DVM, DVSc, PhD; Laurie Boucher^2^, DVM; Carolyn Arnold^3^; Luis Arroyo^4^; Marcio Costa^5^; Mathilde Leclere^6^; Jan Suchodoski^7^

#### 85.1.1 ^1^Associate Professor, University of Montreal; ^2^PhD student, University of Montreal; ^3^Texas Tech University; ^4^Department of Clinical Sciences, University of Guelph; ^5^Department of Veterinary Biomedical Sciences, University of Montreal; ^6^Department of Clinical Sciences, University of Montreal; ^7^Texas A&M

**Background:** DNA sequencing is commonly used to analyze microbiota composition but does not provide absolute bacterial quantification. The quantification of certain bacterial taxa has been used as proxies for dysbiosis in various species.

**Objectives:** To establish reference values for key fecal bacterial taxa using quantitative PCR (qPCR) as indicators of gut health in horses.

**Animals:** Fecal samples from 50 healthy horses and 30 horses diagnosed with colitis.

**Methods:** Thirteen bacterial taxa associated with gut health and dysbiosis were selected based on the current literature. qPCR assays were optimized for these taxa and total bacterial load. Each sample was analyzed in duplicate at a standardized DNA concentration (5 ng/μL). Group comparisons were performed using the Student's *t*‐test.

**Results:** Healthy horses had significantly higher levels of *Turicibacter* and Lachnospiraceae (*p*≤0.01), whereas horses with colitis exhibited significantly higher levels of *Escherichia coli*, *Fusobacterium*, and Enterobacteriaceae (*p*≤0.0001). No significant differences were observed for total bacterial load or other taxa, including Ruminococcaceae, *Akkermansia*, *Lactobacillus*, *Enterococcus*, *Fibrobacter*, *Faecalibacterium*, *Prevotella*, and *Streptococcus*. Reference values for each taxon in the studied population were established.

**Conclusions and Clinical Importance:** This rapid, quantitative approach enabled the detection of dysbiosis in a population of horses with colitis without requiring complete microbiota sequencing. Future studies applying this method may facilitate early microbiota‐based diagnostics for equine intestinal diseases and monitor microbiota recovery following antimicrobial therapy.

## 86 ABSTRACT E46: First Experience With Radiofrequency Ablation to Reduce Recurrence Risk of Atrial Fibrillation in Horses

### 86.1 **Eva Buschmann** ^1^, DVM; Glenn Van Steenkiste^2^, DVM, PhD; Ingrid Vernemmen^2^, DVM, PhD; Stijn Schauvliege^3^, DVM, PhD; Annelies Decloedt^4^, DVM, PhD; Mattias Duytschaever^5^, MD, PhD; Gunther van Loon^2^

#### 86.1.1 ^1^PhD Fellow, Ghent University; ^2^Internal Medicine, Reproduction and Population Medicine, Ghent University; ^3^Prof. Dr., Large Animal Surgery, Anaesthesia and Orthopaedics, Ghent University; ^4^Prof. Dr., Internal Medicine, Reproduction and Population Medicine, Ghent University; ^5^Prof. Dr., Cardiology, AZ Sint‐Jan

**Background:** Myocardial sleeves (MS) in the caudal vena cava and pulmonary veins are a potential trigger for initiation and perpetuation of atrial fibrillation (AF) in horses.

**Hypothesis/Objectives:** Isolation of these MS might reduce the arrhythmogenic substrate, thereby lowering the AF recurrence risk.

**Animals:** Six horses with recurrent AF. Horses had one (*n* = 4) or two (*n* = 2) recurrences, after a median of 91 [0.02–304] days post‐cardioversion.

**Methods:** After transvenous electrical cardioversion of AF, voltage mapping of the caudal vena cava was performed in all horses. In two horses, also pulmonary vein ostium I (*n* = 2), II (*n* = 1) and IV (*n* = 2) were mapped. Point‐by‐point, ablation index‐guided radiofrequency ablation was performed to isolate MS.

**Results:** Voltage mapping identified MS in the caudal vena cava (*n* = 5), ostium II (*n* = 1) and IV (*n* = 2), which were subsequently isolated, as confirmed by demonstrating entrance and exit block. To date, three horses remain in sinus rhythm with a follow‐up of 9–32 months post‐ablation. Three horses showed recurrence after 2 days, 6 months and 11 months. The horse that relapsed after 6 months underwent a second procedure to isolate ostium III and remains in sinus rhythm 2 months post ablation.

**Conclusions and Clinical Importance:** Isolation of MS reduced arrhythmogenic tissue. Isolating pulmonary vein ostia, especially ostium III, remains challenging. To date, 4 of 6 horses with recurrent AF remain in sinus rhythm but it remains difficult to demonstrate the additional benefit of ablation. This is the first study suggesting that ablation might benefit horses with AF, but more data are needed.

## 87 ABSTRACT E47: Effect of Omeprazole Treatment on the Pharmacokinetics of Orally Administered Flunixin Meglumine in Adult Horses

### 87.1 **Serena Ceriotti** ^1^, DVM, MS, PhD, DACVIM (LAIM); Giulia Memoli^2^, DVM; Michela Bullone^2^, DVM, PhD; Kara Lascola^3^, DVM, MS, DACVIM (LAIM); Luigi Intorre^2^, DVM, PhD, DECVPT; Valentina Meucci^2^, PharmD, PhD

#### 87.1.1 ^1^Clinical Lecturer, Auburn University; ^2^Universita’ degli Studi di Torino; ^3^Auburn University

**Background:** In humans, omeprazole‐induced gastric pH increase interferes with absorption of co‐administered oral drugs. Despite frequent co‐administration in horses, its effect on flunixin meglumine absorption remains uninvestigated.

**Hypothesis/Objectives:** The study aimed to compare flunixin pharmacokinetics in adult horses with and without 5‐day omeprazole treatment. We hypothesized that: I) omeprazole would increase gastric pH and reduce flunixin bioavailability; II) despite reduced bioavailability, flunixin would reach therapeutic plasma concentrations (> 0.75 μg/ml).

**Animals:** Six healthy adult geldings from a research herd.

**Methods:** Using a Latin‐square crossover design, horses received flunixin (1.1 mg/kg) via intravenous (IV) route, intragastric (IG) route, and intragastric route after 5 days of 4 mg/kg oral omeprazole (OM). Gastric pH was measured 24 h before flunixin administration in IG and OM protocols using a pH meter (pH700, Oaklon®). Plasma flunixin concentrations were quantified by High Performance Liquid Chromatography at 14 time points over 36 h and analyzed via non‐compartmental pharmacokinetics (Excel PKSolver®). Gastric pH and pharmacokinetic variables were compared between IG and OM protocols using paired *t*‐tests (*p* < 0.05). Results In the OM protocol, gastric pH was alkaline in all horses (7.61±0.42) and significantly higher (*p* = 0.0001) than in the IG protocol (2.99±1.34). Flunixin plasma concentration versus time data are reported in figure 1. Omeprazole significantly reduced flunixin bioavailability, maximum concentration, and overall drug exposure (Table 1).

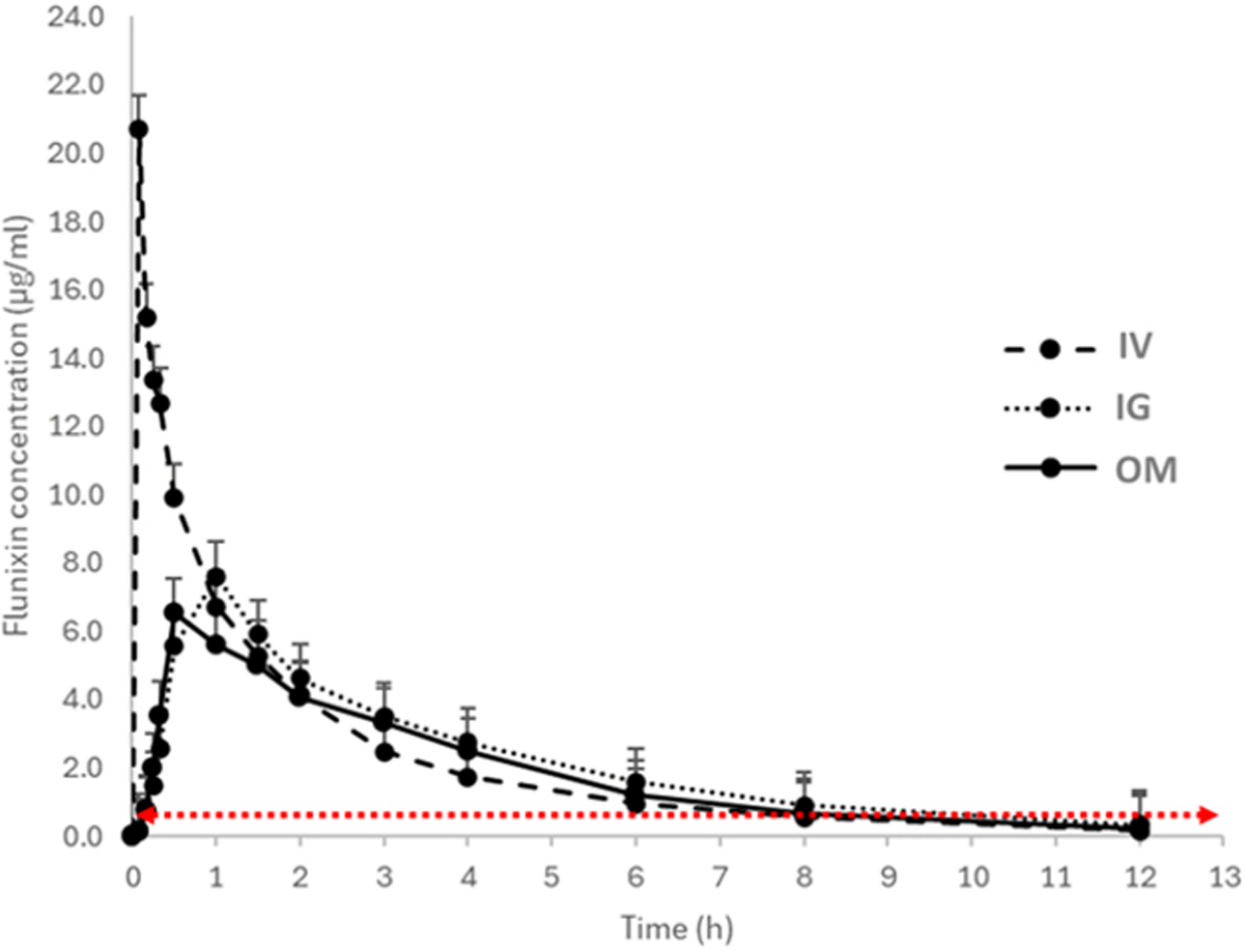

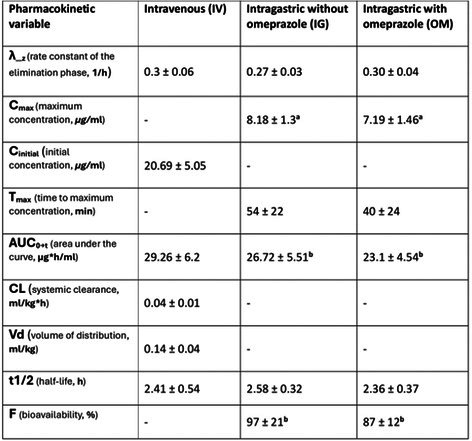

**Conclusions and Clinical Importance:** Omeprazole‐induced gastric pH elevation significantly reduced flunixin absorption but did not prevent plasma concentrations from reaching therapeutic levels for clinically relevant durations.

## 88 ABSTRACT E48: Investigation of MARCKS Protein as a Novel Therapeutic Target to Decrease Nets in Equine Asthma

### 88.1 **Bethanie Cooper** ^1^, DVM; Rosemary Bayless^2^, DVM, PhD, DACVIM; Eric Brooks^2^; Kate Hepworth^2^; Kallie Hobbs^3^; Katie Sheats^4^, DVM, PhD, DACVIM

#### 88.1.1 ^1^Graduate Research Assistant, North Carolina State University; ^2^North Carolina State University; ^3^Texas A&M; ^4^Associate Dean and Director of Professional Education and Admissions, North Carolina State University

**Background:** Severe equine asthma (sEA) is a chronic, inflammatory lower‐airway disease characterized by cough, increased respiratory rate and effort at rest, and elevated lower airway neutrophils. Neutrophils contribute to ongoing inflammation and tissue damage through the release of proteolytic enzymes, reactive oxygen species and neutrophil extracellular traps (NETs.) NETs are extruded strands of DNA intercalated with histones and enzymatic proteins. Given their role in inflammation and tissue damage, NETs represent a potential therapeutic target for sEA.

**Hypothesis:** NETs are increased in the lower airways of horses with sEA and inhibition of MARCKS with BIO‐11006 will attenuate NETosis in equine neutrophils.

**Animals:** 52 university‐owned teaching/research horses and client‐owned horses.

**Methods:** Cell free DNA (cfDNA) concentration was measured in BAL supernatant using the Qubit 4 fluorometer. Immunocytochemistry stained for two additional components of NETs: myeloperoxidase, and citrullinated‐histone 3. Peripheral blood neutrophils were isolated, pretreated with BIO‐11006 (MARCKS inhibitor) or RNS (control) and stimulated. CfDNA was quantified with SYTOX green.

**Results:** CfDNA was significantly (*p* < 0.05) higher in BAL supernatant from horses with sEA compared to healthy horses and horses with non‐neutrophilic asthma. Confocal microscopy revealed NETs in BAL cytospins from horses with sEA, but not in cytospins from healthy horses. BIO‐11006 significantly decreased A23187‐induced NET formation in peripheral blood neutrophils.

**Conclusions:** These findings support further investigation of NETs as a therapeutic target for sEA. Next steps include determining the effect of nebulized MARCKS‐inhibitor peptides on *in vivo* NETs in horses with sEA.

## 89 ABSTRACT E49: Antimicrobial Susceptibility of Bacterial Isolates Recovered from Sick Neonatal Foals

### 89.1 **Daniela Espinosa** ^1^, DVM; Luis Arroyo^2^, DVSc, PhD, DACVIM; Daniel Kenney^3^, VMD; Gabrielle Monteith^4^; Christopher Riley^5^, CertTeReo, BSc, BVSc (Hons), PGCIM, MA (Lead), MSc, PhD, DACVS, MANZCVS; Ashley Spencer^6^, SAIM

#### 89.1.1 ^1^MSc Student, Clinical Studies, Ontario Veterinary College, University of Guelph; ^2^Professor, Clinical Studies, Ontario Veterinary College, University of Guelph; ^3^Clinical Studies, Ontario Veterinary College, University of Guelph; ^4^Research Statistician, Clinical Studies, Ontario Veterinary College, University of Guelph; ^5^Professor and Chair, Clinical Studies, Ontario Veterinary College, University of Guelph; ^6^Assistant Professor, Clinical Studies, Ontario Veterinary College, University of Guelph

**Background:** Neonatal septicemia and its sequelae cause death and morbidity in foals. Clinicians require knowledge of geographically specific pathogens and their antibiotic resistance (AMR) patterns to guide treatment while awaiting antimicrobial susceptibility results. Data to guide the initial treatment of foals in Ontario are unavailable.

**Hypothesis/Objectives:** Pathogens and AMR and multidrug resistance (MDR) patterns are geographically unique. This study aimed to identify the bacterial species, AMR and MDR of isolates from sick foals in Ontario.

**Animals:** All foals < 1 mo hospitalized at Ontario Veterinary College during 2024.

**Methods:** This study was a prospective cohort study. Blood obtained on admission, 48 h and 72 h, and other samples, underwent aerobic and anerobic bacterial culture and identification. Antimicrobial susceptibilities were determined by minimum inhibitory concentrations. Logistic regression identified factors associated with MDR.

**Results:** Blood (84), umbilicus (5), and synovial fluid (7) submissions from 79 foals produced 99 isolates. *Escherichia coli* (21%), *Enterococcus* spp. (18%), *Streptococcus* spp. (17%), and *Staphylococcus* spp. (12%) were most common. AMR was highest for tetracyclines (72%; 67/93) and beta‐lactams (55%; 52/95). MDR rate was 54%; highest for *E. coli* (67%;14/21) and *Klebsiella* spp. (43%; 4/7) isolates. Antibiotic administration (*P* = 0.014), multiple antimicrobials (*P* = 0.002) and use of beta‐lactam antibiotics (*P* = 0.003) increased the odds of MDR.

**Conclusions:** The prevalence of MDR, particularly in *E. coli* and other endemic bacteria from sick neonatal foals in Ontario, preadmission antimicrobial use, and extended hospitalization‐associated MDR risk, emphasize the need for targeted stewardship.

## 90 ABSTRACT E50: Safety, Immunogenicity, and Protective Efficacy of an Equine Rotavirus B Vaccine

### 90.1 **Olivia Jacob** ^1^, BS; Ben Hause^2^, PhD; Lianne Eertink^3^, MS; Eric Bumgardner^4^, MS; Shalini Soni^5^, PhD; Ahsan Naveed^6^, DVM, PhD; Shavahn Loux^7^, PhD; Allen Page^8^, DVM, PhD; Dan Wang^8^, PhD; Emma Adam^9^, BVetMed (DVM), PhD, MRCVS, DACVIM, DACVS; Feng Li^10^, DVM, PhD

#### 90.1.1 ^1^Graduate Student, Gluck Equine Research Center, University of Kentucky; ^2^Chief Scientific Officer, Cambridge Technologies; ^3^Graduate Student, Veterinary Science, Gluck Equine Research Center, University of Kentucky; ^4^Research Scientist, Cambridge Technologies; ^5^Postdoctoral Scholar, Veterinary Science, Gluck Equine Research Center, University of Kentucky; ^6^Molecular Diagnostic Lab Manager II, University of Nebraska; ^7^Assistant Professor, Equine Physiology/Bioinformatics, Louisiana State University; ^8^Assistant Professor, Veterinary Science, Gluck Equine Research Center, University of Kentucky; ^9^Associate Professor, Equine Veterinary Outreach, Liaison/Research, Veterinary Science, Gluck Equine Research Center, University of Kentucky; ^10^Professor, William Robert Mills Chair in Equine Infectious Diseases, Veterinary Science, Gluck Equine Research Center, University of Kentucky

**Background:** Equine rotavirus B (ERVB) is an emerging pathogen that causes severe diarrhea in foals. Currently, there are no vaccines to prevent ERVB infection in horses.

**Hypothesis/Objectives:** This study is to develop a baculovirus‐based VP8 vaccine that protects foals against ERVB infection.

**Animals:** 15 healthy horses and 12 healthy pregnant mares were employed in this study.

**Methods:** In Study 1, horses were divided into two groups (10 vaccinated, 5 controls), with each group receiving two doses of vaccine or sham control respectively at 3 weeks apart. Serum samples were collected from these horses before and after immunization, which were assayed for VP8 antibody responses. Study 2 divided pregnant mares into two groups (6 vaccinated, 6 sham vaccinated), who were vaccinated during their 8th, 9th, and 10th month of gestation. On day 1 of life foals were orally challenged with ERVB. Colostrum and serial blood samples from foals were collected for measuring VP8 antibody titers in ELISA. Collected fecal samples were analyzed by RT‐qPCR to determine viral shedding.

**Results:** All vaccinated horses remained clinically normal, with no clinically relevant adverse reactions observed. In study 1, a robust VP8 antibody response was detected. In study 2, following viral challenge, 2 out of 6 foals from the vaccination group were completely protected, with the other 4 foals shedding lower quantities of the virus compared to infected foals from the sham vaccination group.

**Conclusions and Clinical Importance:** The baculovirus‐based VP8 vaccine is safe, immunogenic, and effective to protect foals from ERVB infection.

## 91 ABSTRACT E51: Antimicrobial Use in Pediatric Patients with Neonatal Infections in an Equine Teaching Hospital in 2023

### 91.1 **Daniel Jean** ^1^, DVM, DACVIM (LA), PhD; Fleur Béluze^2^, DVM; Maud de Lagarde^3^, DVM, DACVIM, PhD

#### 91.1.1 ^1^Equine Internal Medicine Professor, University of Montreal; ^2^Intern, Clinical Sciences, University of Montreal; ^3^Professor, Pathology and Microbiology, University of Montreal

**Background:** Antimicrobial judicious usage in veterinary and human medicine is in constant reflection in view of responsible management of antibiotic resistance while targeting ideal clinical effectiveness.

**Objectives:** Describe antimicrobial usage in equine pediatric patients, as well as clinical efficiency and associated short‐term prognosis.

**Animals:** Sixty equine pediatric patients.

**Methods:** The study included pediatric patients, 0–30 days of age, presented in the ICU at the University of Montreal's equine hospital (2023), that received an antimicrobial treatment. Data on patient characteristics, septic scores as well as antimicrobial use in our hospital medical data base were collected

**Results:** Most (70%) of the pediatric patients were up to one week old. Ceftiofur (74.6%) was used most often followed by penicillin‐gentamicin association (52.5%). The most common administered first‐line antibiotic was ceftiofur (63.3%) and 80% of foals received a first‐class antibiotic during hospitalization. A change of antibiotic was decided for 14 foals (23.3%) based on the antimicrobial susceptibility test (AST) results. The short‐term (end of hospitalization) survival rate was 83%. There is no correlation between the survival rate and the antibiotic therapy administered, nor between the survival rate and the septic score of the foals upon admission.

**Conclusion and Clinical Importance:** This study highlights the frequent use of highest‐priority critically important antimicrobials in pediatric patients at an equine teaching hospital. The AST results allowed for targeting antibiotic therapy in nearly one‐quarter of pediatric patients. These findings underscore the importance of strengthening antibiotic stewardship programs and providing alternatives to ceftiofur for treating septic neonatal foals.

## 92 ABSTRACT E52: Establishing Metabolic Parameters in Mules

### 92.1 **Audrey Johnston** ^1^, BS; Amy McLean^2^; Cynthia Xue^3^; Sarah Jacob^4^; Elaine Norton^5^

#### 92.1.1 ^1^Student, University of Arizona; ^2^University of California–Davis; ^3^Ross University; ^4^Michigan State University; ^5^University of Arizona

**Objective:** This project aimed to establish reference intervals for key metabolic biochemical measurements and assess the prevalence and impact of risk factors for metabolic syndrome in mules.

**Animals:** 159 mules and 31 horses (2–20 years old)

**Methods:** Phenotypic data included fasting insulin, triglycerides, ACTH, non‐esterified fatty acids (NEFA), and insulin post oral sugar test (INS‐OST), body condition score (BCS), and cresty neck score (CNS). Stepwise regression was used for model analysis with farm as a random effect and sex, age, CNS, BCS, and month as fixed effects. The percentage of phenotypic variation explained by each predictor was calculated from mixed model analyses. Significant differences between horses and mules were identified using pairwise comparisons with a Tukey‐corrected *p* value < 0.05. Parametric reference intervals were calculated using data from 117 mules (2–15 years old, BCS 4.5–7), applying Cook's distance for outlier detection.

**Results:** Across our cohort, 15% of the mules were obese and 32% had a CNS ≥ 3. CNS and sex significantly influenced most outcome variables, explaining 1–5% of the variation (R2), while farm accounted for 18–26%. Triglycerides were 1.4 times higher (95% CI 1.08–1.59, *p* = 0.007) and ACTH 1.3 times lower (95% CI 1.08–1.49, *p* = 0.003) in mules than horses. Mule‐specific reference intervals were established for ACTH (2.6–22.2 pg/mL; *p* < 0.001), insulin (2.6–18.0 μIU/mL; *p* < 0.001), triglycerides (11.6–98.6 mg/dL; *p* = 0.02), NEFA (≤0.44 mmol/L; *p* < 0.001), and INS‐OST (2.5–27.9 μIU/mL; *p* < 0.001).

**Conclusion and Clinical Importance:** These results provide essential insights into the metabolic health of mules and provides initial information to diagnose metabolic changes in mules.

## 93 ABSTRACT E53: Changes in Serum Basal Insulin Concentration During Estrous Cycles in Healthy Mares

### 93.1 **Ninja Karikoski** ^1^, DVM, PhD; Essi Lautala^2^, DVM; Maria Kareskoski^2^, DVM, PhD

#### 93.1.1 ^1^Senior Clinical Teacher, University of Helsinki, Finland; ^2^University of Helsinki, Finland

**Background:** Anecdotally, mares are at increased risk of laminitis during estrus; however, data on estrous cycle‐related changes in blood insulin concentration are limited.

**Hypothesis:** Serum basal insulin varies depending on estrous cycle phase.

**Animals:** 10 healthy, non‐pregnant riding school mares.

**Methods:** In this prospective cohort study, serum for basal insulin concentration was collected from each horse 9 times weekly (median number of days between samplings 7, range 5–15) during spring 2024. The estrous cycle was monitored on the same days using rectal examination and ultrasonography and categorized as estrus (follicles > 25 mm and endometrial edema) or diestrus (corpus luteum, no endometrial edema). Serum insulin concentration was analyzed with a commercial equine‐optimized ELISA (Mercodia equine insulin ELISA). Insulin values during and after the first ovulatory estrus were included in the statistical analysis.

**Results:** Serum basal insulin concentration varied within the horses. The median insulin concentrations in estrous and diestrus phases were 22.5 (min–max 6.8–87.5) μIU/mL and 15.2 (min–max 2.4–55.3) μIU/mL, respectively, and there was no difference between the phases (*p* = 0.397). One horse was classified as having insulin dysregulation (insulin > 71 μIU/mL) in one estrus sample and insulin concentration in the equivocal zone (49–70.9 μIU/mL) in another estrus sample. Two other horses had insulin concentration in the equivocal zone in one diestrus sample. None of the horses had signs of laminitis.

**Conclusions and Clinical Importance:** The estrous cycle phase was not associated with changes in serum basal insulin concentration. Therefore, the estrous cycle phase may not predispose mares to laminitis.

## 94 ABSTRACT E54: Endocrine Disorder Classifications and Clinical Sign Frequencies Using Static and Dynamic Testing Among Horse Breeds

### 94.1 **Rachel Lemcke** ^1^, BS, MS; Heather Broscious^2^; Steven Grubbs^2^, DVM, PhD, DACVIM

#### 94.1.1 ^1^Founder and Independent Veterinary Laboratory Consultant, Amwell Data Services LLC; ^2^Boehringer Ingelheim Animal Health USA

**Background:** Rates of endocrine disorders vary among breeds, though less is known about endocrine‐associated clinical sign (EACS) frequencies among breeds.

**Hypothesis/Objectives:** The objective of this research is to evaluate PPID classification and EACS frequencies for different breeds using pre‐ and post‐TRH stimulation ACTH values.

**Animals:** Arabians (*n* = 194), Thoroughbreds (*n* = 217), and Warmbloods (*n* = 371), ages 4 to 38, with EACS were tested for endocrine disorders using spring TRH stimulation testing by their veterinarian from 2016–2023.

**Methods:** Horses were considered PPID positive if spring basal ACTH levels were > 35 pg/mL (2016–2020) or > 30 pg/mL (2021–2023), or spring post‐TRH‐stimulated ACTH levels were > 110 pg/mL. EACS rates were determined per PPID classification using pre‐ and post‐TRH‐stimulation ACTH levels. PPID rates were analyzed using two‐way ANOVA; odds ratios were calculated per EACS.

**Results:** PPID classification among breeds using pre‐and post‐TRH stimulation ACTH values were similar (*p* > 0.9999). Using basal ACTH values, 21%–36% of horses were classified as PPID positive, increasing to 36–72 percent when post‐TRH stimulation ACTH values were utilized (*p* < 0.0001). Using post‐TRH stimulation ACTH levels for PPID classification instead of basal ACTH levels reclassified 18–39 percent of horses from PPID negative to PPID positive, and reclassified 3–5 percent from PPID positive to PPID negative (*p* = 0.0465). EACS frequencies significantly varied among breeds depending on pre‐ or post‐TRH stimulation levels (*p* < 0.0001). No odds ratios reached statistical significance (α=0.05; *p* = 0.1176‐> 0.9999; 95% CI included 1).

**Conclusions and Clinical Importance:** Different EACS frequencies were observed among these breeds, revealing opportunities to refine identification strategies for candidates for dynamic ACTH testing.

**Table:** **Table 1.** Categorizations of PPID using basal ACTH and post‐TRH stimulation ACTH values within three horse breeds

	Positive **on basal ACTH/**positive **on post‐TRH stim**.		Positive **on basal ACTH/**negative **on post‐TRH stim**.		Negative **on basal ACTH/**positive **on post‐TRH stim**.		Negative **on basal ACTH/**negative **on post‐TRH stim**.		
Breed	Qty horses	Perc. horses	Qty horses	Perc. horses	Qty horses	Perc. horses	Qty horses	Perc. horses	Total horses
Arabian	63	32.47	7	3.61	77	39.69	47	24.23	194
Thoroughbred	40	18.43	13	5.99	40	18.43	124	57.14	217
Warmblood	66	17.79	14	3.77	106	28.57	185	49.87	371

**Figure:**
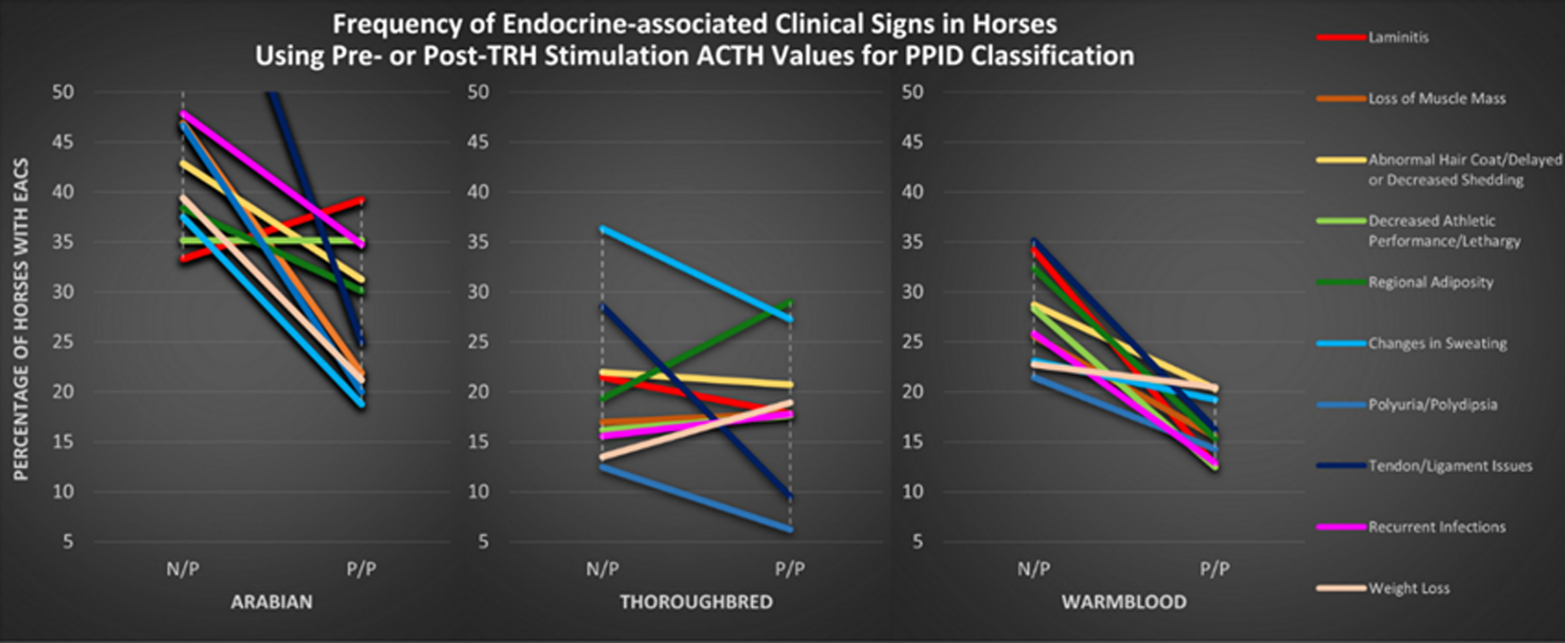
**Figure 1** Frequency of endocrine‐associated clinical signs (“EACS”) in Arabians, Thoroughbreds, and Warmbloods using pre‐ and post‐TRH stimulation ACTH values for PPID classifications. Horses classified as PPID positive on basal ACTH values and negative on post‐TRH stimulation ACTH values categorized as “N/P,” while horses positive on both pre‐ and post‐TRH stimulation ACTH values are categorizes and “P/P.” Omitted from the figure is the 75 percent value for N/P Arabian for tendon/ligament pathologies.

## 95 ABSTRACT E55: Frequency of Endocrine‐Associated Clinical Signs in Equids with Different Risk Levels for Hyperinsulinemia‐Associated Laminitis

### 95.1 **Rachel Lemcke** ^1^, BS, MS; Heather Broscious^2^; Steven Grubbs^2^, DVM, PhD, DACVIM

#### 95.1.1 ^1^Founder and Independent Veterinary Laboratory Consultant, Amwell Data Services LLC; ^2^Boehringer Ingelheim Animal Health USA

**Background:** Recent Equine Endocrinology Group guidelines differentiate risk levels for hyperinsulinemia‐associated laminitis (HAL) by insulin level. Understanding the prevalence of different insulin ranges and endocrine‐associated clinical signs (EACS) that correlate with an increased risk of HAL aids in successful management of insulin dysregulation.

**Hypothesis/Objectives:** The objective was to evaluate frequency of EACS with different ranges of insulin values.

**Animals:** Equids (*n* = 33,941), ages 1 to 45, including miniature donkeys (*n* = 188), ages 3 to 38, with EACS were tested for endocrine disorders by their veterinarian.

**Methods:** A retrospective analysis was performed on veterinarian‐provided data from a 2016–2023 study of equids with suspected endocrine disorders. Equids were considered PPID positive if spring basal, spring post‐TRH‐stimulated, or fall basal ACTH levels were > 35, > 110, or > 50 pg/mL, respectively, and resting insulin cutoff values were > 100 μIU/mL (“HIL”), > 45–100 μIU/mL (“MIL”), and 0–45 μIU/mL (“LIL”).

**Results:** One in four and one in ten equids evaluated had MIL and HIL, respectively, and nearly 58 percent of those equids had PPID. Laminitis and regional adiposity (“RA”) was reported in 40.78 and 43.09 percent in equids with HIL, respectively. In miniature donkeys with HIL, laminitis and RA rates were 63.16 and 78.95 percent, respectively, and RA rates were also elevated in 73.17 percent of miniature donkeys with MIL. Only 38.56 and 15.79 percent of equids and miniature donkeys with HIL, respectively, did not show either laminitis or RA.

**Conclusions and Clinical Importance:** Practitioners should continue to evaluate insulin levels in equids, despite any absence of RA or laminitis.

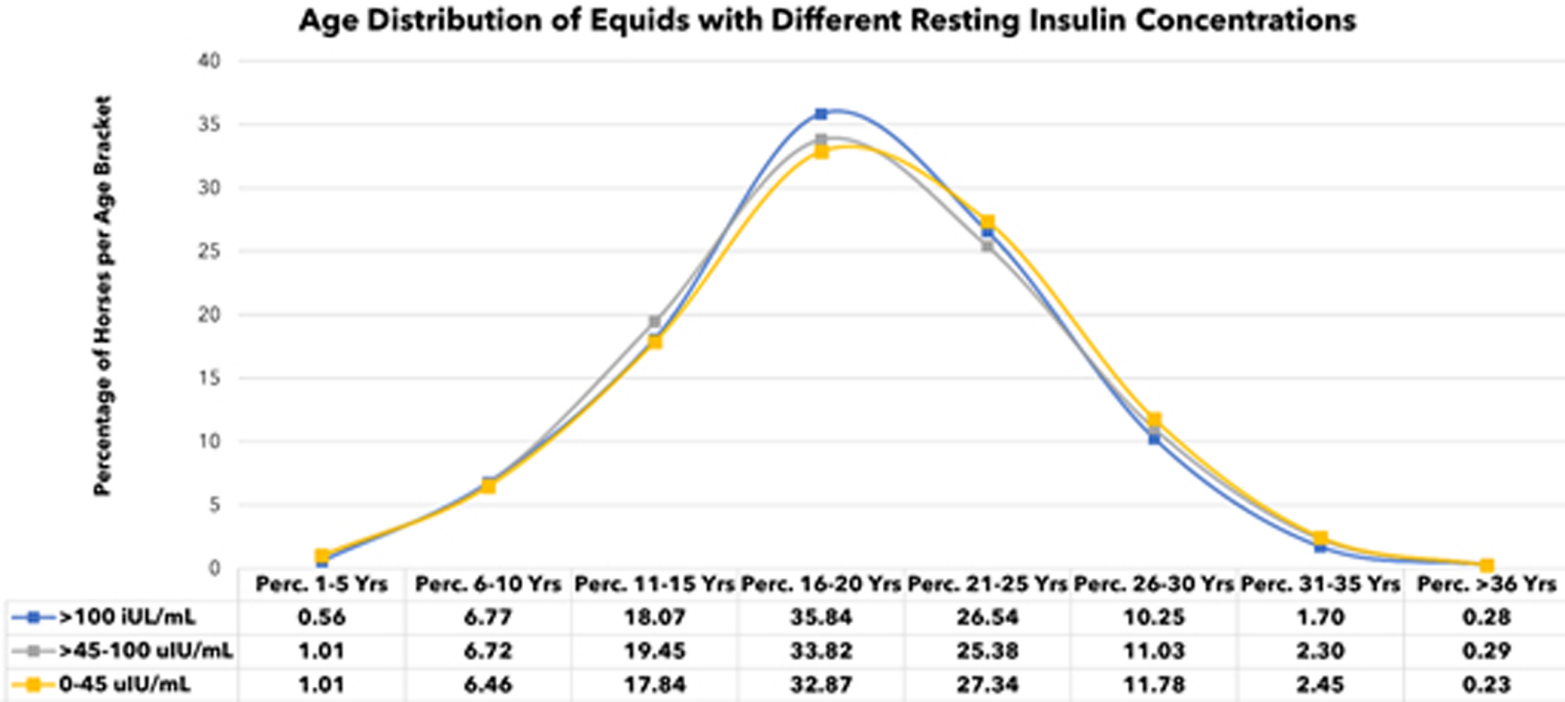

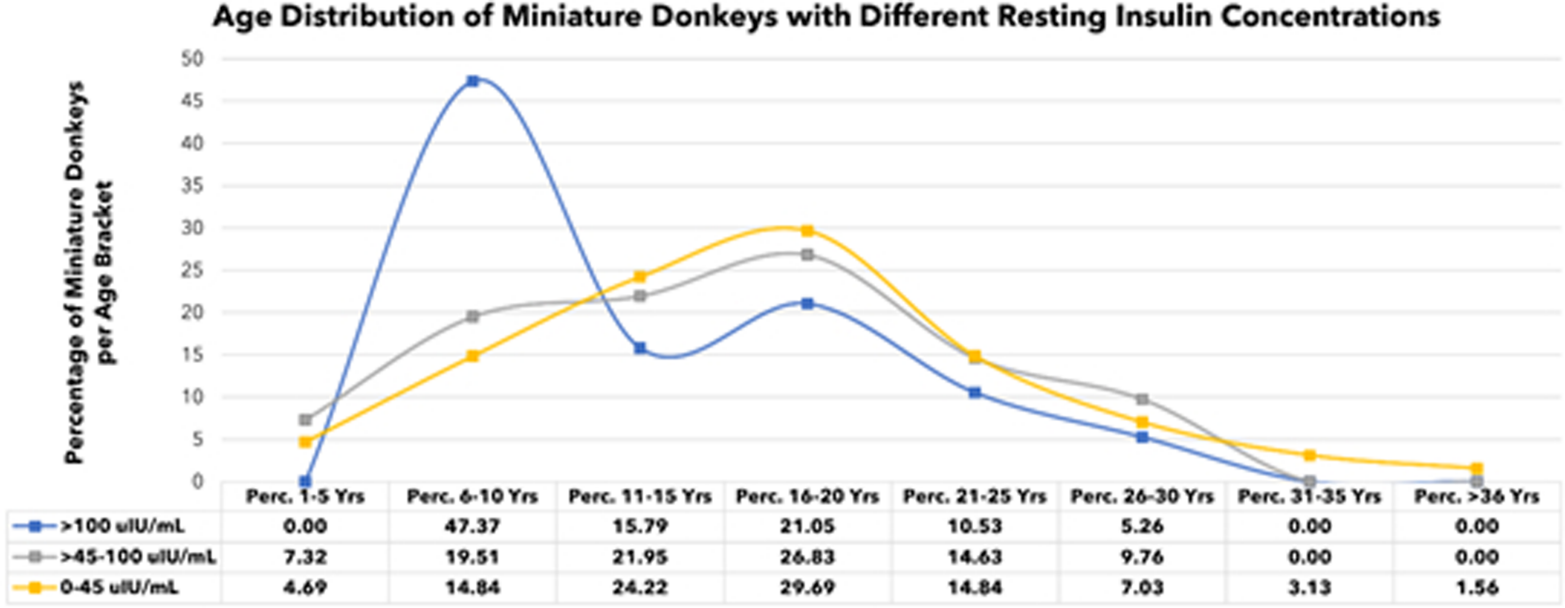

## 96 ABSTRACT E56: A Randomized, Placebo‐Controlled Crossover Study of Bexagliflozin Treatment in Horses With Naturally‐Occurring Insulin Dysregulation

### 96.1 **Caitrin Lowndes** ^1^, MVB; Daniela Luethy^2^; Darko Stefanovski^2^; Georgia Skelton^2^; Jeaneen Kulp^2^; Andrew Van Eps^2^

#### 96.1.1 ^1^Musculoskeletal Research Fellow, New Bolton Center, University of Pennsylvania; ^2^New Bolton Center, University of Pennsylvania

**Background:** A bexagliflozin formulation registered for cats could effectively treat insulin dysregulation (ID) in horses.

**Hypothesis/Objectives:** We hypothesized that the sodium‐glucose cotransporter 2 inhibitor bexagliflozin would reduce the insulin response to oral sugar tests (OST) in horses with ID.

**Animals:** Ten client‐owned Arabian horses (5–18 years; 442–571 kg) with naturally‐occurring ID (mean OST maximal insulin 88 [range 51–157] μIU/ml).

**Methods:** Randomized, blinded, placebo‐controlled crossover: 5 horses received placebo and 5 received 15 mg bexagliflozin once daily (~0.03 mg/kg; BEXA3) for 10 days. After a 4 day washout, treatments (swapped) were continued for 10 days followed by a 12 day washout. Then, all horses were treated with 30 mg bexagliflozin (~0.06 mg/kg; BEXA6) daily for 7 days. An OST (0.15 ml/kg Karo syrup) was performed at the beginning and end of each treatment period. Insulin (resting and OST) and triglycerides were compared (pre‐ vs. post‐treatment) using a mixed‐effects linear regression model.

**Results:** Bexagliflozin treatment was associated with a mean [95% confidence interval] reduction in resting insulin (BEXA3: −15[−29 to ‐1.4] μIU/mL; BEXA6: −21 [−35 to −6.5] μIU/ml); OST maximal insulin (BEXA3: −30 [−51 to −8.8] μIU/ml; BEXA6: −46[‐68 to −24] μIU/ml) and OST insulin area‐under‐the‐curve (BEXA3 −2562 [−4493 to −631] μIU/ml*min; BEXA6 −4154 [−6168 to −2139] μIU/ml*min); *P* < 0.001. Resting serum triglycerides slightly increased with BEXA3 (40 [31–49] mg/dl) and BEXA6 (52[43–61] mg/dl) compared to no treatment (32 [25–38] mg/dl); *P* < 0.001. No significant changes were associated with placebo (*P* > 0.05).

**Conclusions and Clinical Importance:** In this cohort, short‐term oral bexagliflozin treatment was effective for insulin control with only minor increases in serum triglycerides.

## 97 ABSTRACT E57: Decreased Adiponectin and Increased Leptin—Adiponectin are Associated With Insulin Dysregulation and Laminitis in Light‐Breed Horses

### 97.1 **Caitrin Lowndes** ^1^, MVB; Daniela Luethy^2^; Georgia Skelton^2^; Jeaneen Kulp^2^; Andrew Van Eps^2^

#### 97.1.1 ^1^Musculoskeletal Research Fellow, New Bolton Center, University of Pennsylvania; ^2^New Bolton Center, University of Pennsylvania

**Background:** Associations between hypoadiponectinemia, insulin dysregulation (ID) and laminitis risk have been established in ponies, but not horses. Leptin:adiponectin (L/A) is a potentially useful marker of ID and laminitis risk.

**Hypothesis/Objectives:** We hypothesized that light‐breed horses with ID and/or pre‐existing laminitis would have lower serum adiponectin and increased L/A compared to healthy horses.

**Animals:** 410 client‐ and university‐owned mixed light‐breed horses.

**Methods:** Cross‐sectional cohort study. Resting serum total adiponectin, leptin and insulin concentrations (unfasted) were measured in all horses. Oral sugar tests (OST, 0.15 ml/kg Karo syrup; *n* = 181) and forefoot radiographs (*n* = 137) were performed in a subset. Adiponectin, leptin and L/A were compared between horses with ID (resting or OST insulin > 30 μIU/mL; *n* = 320) and non‐ID horses (OST insulin < 30 μIU/ml; *n* = 90); and laminitic (> 2 degree radiographic distal phalangeal rotation; *n* = 56) vs. non‐laminitic horses (*n* = 81) using Mann‐Whitney tests.

**Results:** Median [interquartile range] serum total adiponectin (μg/ml) was lower (*p* < 0.001) in horses with ID (6.3 [4.8–8.8]) vs. non‐ID (8.3 [6.6–11.2]) and in laminitic (5.8 [3.6–8.4]) vs non‐laminitic (8.1 [6.4–10.3]) horses. Leptin (ng/mL) was higher (*p* < 0.001) in ID (13.8 [8.3–17.3]) vs non‐ID horses (5 [2.3–8.7]), but was not different between laminitic (10.1 [5.9–17.2]) and non‐laminitic horses (10.1 [4–15.4]); *p* = 0.44. L/A ratio was higher (*p* < 0.001) in horses with ID (1.9 [1.2–2.7]) vs. non‐ID (0.5 [0.2–1.2]) and in laminitic (1.9 [1.2–3.3]) vs non‐laminitic (0.96 [0.4–1.9]) horses.

**Conclusion and Clinical Importance:** Resting adiponectin and L/A ratio are potentially useful markers of metabolic health in light‐breed horses. Associations between adiponectin and laminitis warrant further investigation.

## 98 ABSTRACT E58: Placental‐Derived Extracellular Vesicles Enhance *In Vitro* Equine Intestinal Epithelial Repair

### 98.1 **Caroline McKinney‐Aguirre** ^1^, DVM, MPH, PhD, DACVIM (LA); Maryssa Ellis^2^; Victoria Weis^2^; Liara Gonzalez^1^

#### 98.1.1 ^1^Research Assistant Professor, College of Veterinary Medicine, North Carolina State University; ^2^Wake Forest School of Medicine

**Background:** Colic is the most common equine killer beyond old age. Severe colic results in intestinal epithelial barrier breakdown and no existent therapies directly enhance epithelial recovery. Equine placental extract (ePE) accelerates restitution and tight junction recovery (pending publication). Primed placental derived stem cells produce extracellular vesicles (EVs) that enhance epithelial healing in other species.

**Hypothesis:** Naïve, hypoxia‐primed, and LPS‐primed EVs will increase *in vitro* equine intestinal epithelial repair.

**Animals:** Jejunal enteroids were isolated from four hospitalized horses euthanized for non‐gastrointestinal causes. Healthy placental membranes from three university‐owned mares were processed for ePE and EV isolation.

**Methods:** Human placental stem cells (hPSC) were exposed to LPS, hypoxia (5% oxygen) or nothing (naïve) for 48 h (H). Media was ultra‐filtered and chromatographically fractionated to recover hEVs (35–350 nm). Equine jejunal monolayers were injured with 4H hypoxia and scratch wounding, and then apically treated with hEVs or PBS. Scratch margins were measured every 6H until closure. Scratch closure was analyzed using two‐way ANOVA and Tukey multiple comparison. Equine chorioallantois was chemically digested to create ePE, and then ultracentrifuged to collect EVs, as confirmed by transmission electron microscopy.

**Results:** LPS‐primed hEVs accelerate wound closure at 6H (control: 61.2%, LPS‐EV: 66.9%, *p* = 0.002). At 12H, LPS and hypoxia‐primed hEVs hasten wound closure (control: 82.5%, LPS‐EV: 92.2%, *p* = 0.003; hypoxia‐EV: 90.8%, *p* = 0.0011). EVs were successfully isolated from ePE.

**Conclusions and Clinical Importance:** Primed placental hEVs increase early epithelial restitution. ePE containing EVs may provide a targeted therapeutic to accelerate epithelial repair in equine colic patients.

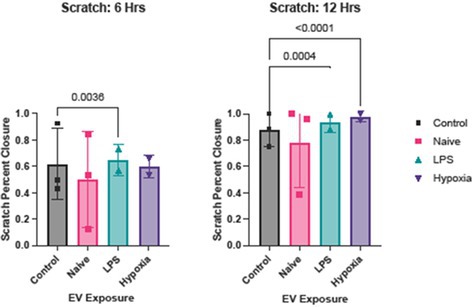

## 99 ABSTRACT E59: Effects of IL‐8 on Neutrophil Extracellular Traps (NETs) in Healthy Equine Neutrophils

### 99.1 **Sydney Mordoh** ^1^, DVM; Basel Abuaita^2^, PhD; Tony Catunda^3^, DVM, PhD; Britta Leise^4^, DVM, PhD, DACVS‐LA

#### 99.1.1 ^1^Equine Surgery Resident and PhD Candidate, School of Veterinary Medicine, Louisiana State University; ^2^Assistant Professor of Infectious Diseases, Pathobiological Sciences, School of Veterinary Medicine, Louisiana State University; ^3^Post‐Doctorate Researcher, Veterinary Clinical Sciences; ^4^Veterinary Clinical Sciences, School of Veterinary Medicine, Louisiana State University

**Background:** IL‐8 has been reported to induce Neutrophil Extracellular Trap formation in people, but its effect on equine neutrophils is unknown. Activation of NETs can occur through NOX2‐dependent or PAD4‐dependent pathways; however, specific activation of these pathways in the horse is not well understood.

**Hypothesis/Objectives:** IL‐8 will induce NETosis through the PAD4‐dependent pathway in neutrophils isolated from healthy horses.

**Animals:** Four healthy horses from the university research herd.

**Methods:** Isolated neutrophils were stimulated with known NET inducers (A213817, PMA, reIL‐8). NET formation was assessed through live‐cell imaging with cytotox green dye, western hybridization for citrullination of histone H3, and immunofluorescence of MPO/DAPI. For live cell analysis, number of NETs formed was determined for 7 h, and data were analyzed using two‐way repeated measure ANOVA with Tukey's *post hoc* comparison between groups. Band intensities and microscopic formation of NETs were subjectively evaluated and compared between groups.

**Results:** Stimulation with PMA resulted in NET formation (*p* < 0.05) starting at 4 h compared to reIL‐8 and media control groups when evaluated through live cell imaging. Strong band intensity was observed in neutrophils stimulated with A23817 for 6 h compared to PMA and media control. reIL‐8 stimulation induced NETosis in a concentration‐dependent manner, with mild increases in band intensity compared to PMA and media control.

**Conclusions and Clinical Importance:** While both pathways of NETosis can be induced in healthy equine neutrophils, IL‐8 appears to mildly stimulate NETosis through the PAD4‐dependent pathway. NETosis in clinical cases and assessment of NET inhibitors remain to be assessed.

## 100 ABSTRACT E60: Clinical and Histological Chronological Characterization of Equine Nasopharyngeal Cicatrix Syndrome in Six Horses in Texas

### 100.1 **Piper Norton** ^1^, DVM, DACVIM (LA); Haley Brown^2^, DVM; Catherine Minehan^2^, DVM; Karen Trainor^3^, DVM, MS, DACVP

#### 100.1.1 ^1^Owner, Texas Equine Medical Associates; ^2^Texas Equine Medical Associates; ^3^Innovative Vet Path

**Background:** Equine Nasopharyngeal Cicatrix Syndrome is a poorly understood condition resulting in permanent mucosal fibrosis of the laryngopharynx. Affected horses often have decreased performance and may require a permanent tracheostomy. This syndrome uniquely occurs in the Southeastern United States with horses in Texas being more commonly affected. The etiology has not yet been elucidated nor have the histological characteristics been described previously.

**Objective:** The objective is to describe six cases of Equine Nasopharyngeal Cicatrix Syndrome, symptomatically, endoscopically, and histologically from the acute to chronic stages over a period of ten weeks.

**Animals:** Six affected horses from one farm had endoscopic laryngopharyngeal biopsies performed at the onset, at 2 weeks post‐onset, and at 10 weeks post‐onset. One normal unaffected horse was sampled for comparison.

**Methods:** The horses were sedated, and lidocaine was liberally applied. Flunixin meglumine was administered intravenously. H&E‐stained and Masson's trichrome stained biopsies were examined, and immunohistochemistry was performed for lymphocytic and histiocytic markers.

**Results:** The most common clinical signs were clear nasal discharge (5/6) and ulceration of the common nasal meatus (6/6). Histologically, lymphoplasmacytic (6/6) and variable eosinophilic (2/6) inflammation was observed in addition to attenuation of the mucosal epithelium initially. Fibrosis of the lamina propria and loss of mucosal glands was noted over time.

**Conclusion:** Equine Nasopharyngeal Cicatrix Syndrome is an important differential diagnosis to consider in cases of clear nasal discharge, especially when numerous horses are affected on one farm. Characterization of the progression of this syndrome will lay the foundation for future studies.

## 101 ABSTRACT E61: Epidemiological Evaluation of Equine Metabolic Syndrome in Arabian Horses and Their Subgroups

### 101.1 **Elaine Norton** ^1^, DVM, MS, PhD, DACVIM (LA); Jennifer Hesser^2^; Audrey Johnston^2^; Brianna Young^2^

#### 101.1.1 ^1^Assistant Professor, University of Arizona; ^2^University of Arizona

**Objective:** Estimate prevalence and risk factors influencing equine metabolic syndrome (EMS) in Arabians and their subgroups.

**Animals:** 768 Arabians (205 geldings, 85 stallions, 476 mares) from 53 farms.

**Methods:** Phenotypic data included basal insulin, triglycerides, leptin, insulin post oral sugar test (INS‐OST), body condition score (BCS), and cresty neck score (CNS). Subgroups were defined as English (*n* = 145), Western (*n* = 87), leisure (*n* = 79), lesson (*n* = 37), halter (*n* = 68), broodmare (*n* = 266), breeding stallion (*n* = 25), or retired (*n* = 39). Prevalence was calculated across and within subgroups for binary outcome variables. Mixed model analyses were performed with biochemical measurements as quantitative outcome variables, farm as a random effect and sex, age, CNS, BCS, month, and subgroup as predictors. Pairwise comparisons identified significant differences between categorical predictors at Tukey‐corrected *p* values < 0.05.

**Results:** Across our cohort, 28% were obese, 57% had a CNS of ≥ 3, 16% had fasting hyperinsulinemia, 18% had post‐OST hyperinsulinemia, and 19% had hypertriglyceridemia. Obesity and post‐OST hyperinsulinemia were most common in broodmares (28%, 21%), retired (36%, 51%), and Western horses (39%, 18%). Breeding stallions had the highest prevalence of a CNS ≥ 3 (67%) but hypertriglyceridemia, hyperinsulinemia, and post‐OST hyperinsulinemia were absent. Retired horses had insulin concentrations 1.7–2.3 times higher than breeding stallions (*p* ≤ 0.001), broodmares (*p* = 0.006) or English horses (*p* ≤ 0.001). Across sex, stallions had 1.3–1.5 times lower INS‐OST (*p* ≤ 0.001) and 1.4 times lower leptin concentrations (*p* ≤ 0.001) compared to geldings and mares.

**Conclusion and Clinical Importance:** These results confirm that Arabians are a high‐risk breed for EMS with differences in prevalence and risk across subgroups and sex.

## 102 ABSTRACT E62: Post‐Mortem Characterization of Subclinical Inflammation of Nuchal Bursa and Ligament in Horses

### 102.1 **Hanna Sfraga** ^1^, DVM; Elena Demeter^2^, DVM, PhD, DACVP; Toby Pinn‐Woodcock^3^, DVM, DACVIM (LAIM); Cassandra Guarino^2^, BS, PhD; Marta Cercone^4^, DVM, PhD, DACVIM (LAIM)

#### 102.1.1 ^1^Veterinarian, Cornell University; ^2^Assistant Professor, Department of Population Medicine and Diagnostic Sciences, Cornell University; ^3^Assistant Clinical Professor, Department of Population Medicine and Diagnostic Sciences, Cornell University; ^4^Assistant Professor, Department of Clinical Sciences, Cornell University

**Background:** Nuchal bursitis is an uncommon, challenging cause of pain and decreased performance in horses. Radiographic identification of mineralization within the nuchal bursa and/or ligament is often considered incidental in the absence of swelling and pain. Recently, cases of nuchal bursitis associated with *Borrelia burgdorferi* infection have been reported.

**Hypothesis/Objectives:** To investigate the incidence of subclinical nuchal bursitis, characterize its histopathologic features and association with *Borrelia burgdorferi*.

**Animals:** Fifteen horses, 4–29 years old, (5 geldings, 10 mares, and various breeds).

**Methods:** Prospective descriptive cadaver study on a convenient population of horses in a *B. burgdorferi* endemic region. Research horses undergoing euthanasia and tissue donation without history or clinical signs of nuchal bursitis. Cranial nuchal bursa, synovial fluid and nuchal ligament were collected post‐mortem. The bursa and ligament were evaluated via histopathology scoring system, grading inflammation, edema, fibrosis, and mineralization (0‐normal to 18‐severe). Lyme Multiplex assay on serum and Borrelia burgdorferi PCR on bursa were performed.

**Results:** Gross abnormalities were found in four horses (calcification, abscess, synovial effusion, adhesions). On histopathology, lymphoplasmacytic and lymphohistiocytic inflammation was detected in four horses. Six horses showed other mild changes (edema, fibrosis, and mineralization). The overall histopathology score ranged from 1 to 7 (mean 2±1.65). Five horses had positive Lyme Multiplex assay antibody titers. All tissue samples were negative on *B. burgdorferi* PCR.

**Conclusion and Clinical Importance:** Our study suggests a high incidence of mild/moderate nuchal bursa and ligament histopathologic changes (67%), warranting further investigation of their causes and clinical implications.

## 103 ABSTRACT E63: Horses With Undifferentiated Colitis Exhibit Downregulation of a Choline Transporter Gene in Colon and Rectum

### 103.1 **Breanna Sheahan** ^1^, DVM, MS, PhD, DACVIM (LA); Lilly Haywood^2^; Morgan Jeter^3^

#### 103.1.1 ^1^Assistant Professor, Equine Medicine, North Carolina State University; ^2^North Carolina State University; ^3^Undergraduate, North Carolina State University

**Background:** Acute undifferentiated equine colitis has a high mortality rate. The pathophysiology of colitis at the epithelial level is incompletely understood. Transcriptomic analysis of intestinal tissues can identify cellular pathways altered in colitis. Rectal biopsies in live horses could represent a suitable substitute for colon.

**Objectives:**

1. Characterize the transcriptome of rectal tissue from clinically healthy horses and colitis cases.
2. Determine if downregulated SLC5A7, a gene encoding a high‐affinity sodium and chloride‐dependent choline transporter, CHT1, in colitis cases persists in a larger dataset and other intestinal tissues (right dorsal colon (RDC), right ventral colon (RVC), cecum).

**Animals:** 11 healthy horses and 5 horses with colitis. Rectal tissue was obtained on all horses, and RDC, RVC, and cecum were obtained in 7 healthy and 4 colitis cases.

**Methods:** An initial pilot RNA‐sequencing dataset was obtained in rectal tissues from 3 healthy horses and 3 colitis horses. Subsequent gene expression (qRT‐PCR) for SLC5A7 was performed on all samples. Immunofluorescence for CHT1 was performed on rectal tissue from healthy and colitis horses.

**Results:** Horses with colitis had a distinct transcriptomic signature as compared to healthy horses (Figure 1). SLC5A7 was significantly decreased in all tissues from colitis horses by RNA‐sequencing and qRT‐PCR (Figure 2). CHT1 immunofluorescence was decreased in colitis tissues.

**Significant Differentially Expressed Genes in Colitis Tissue:**

**Figure:**
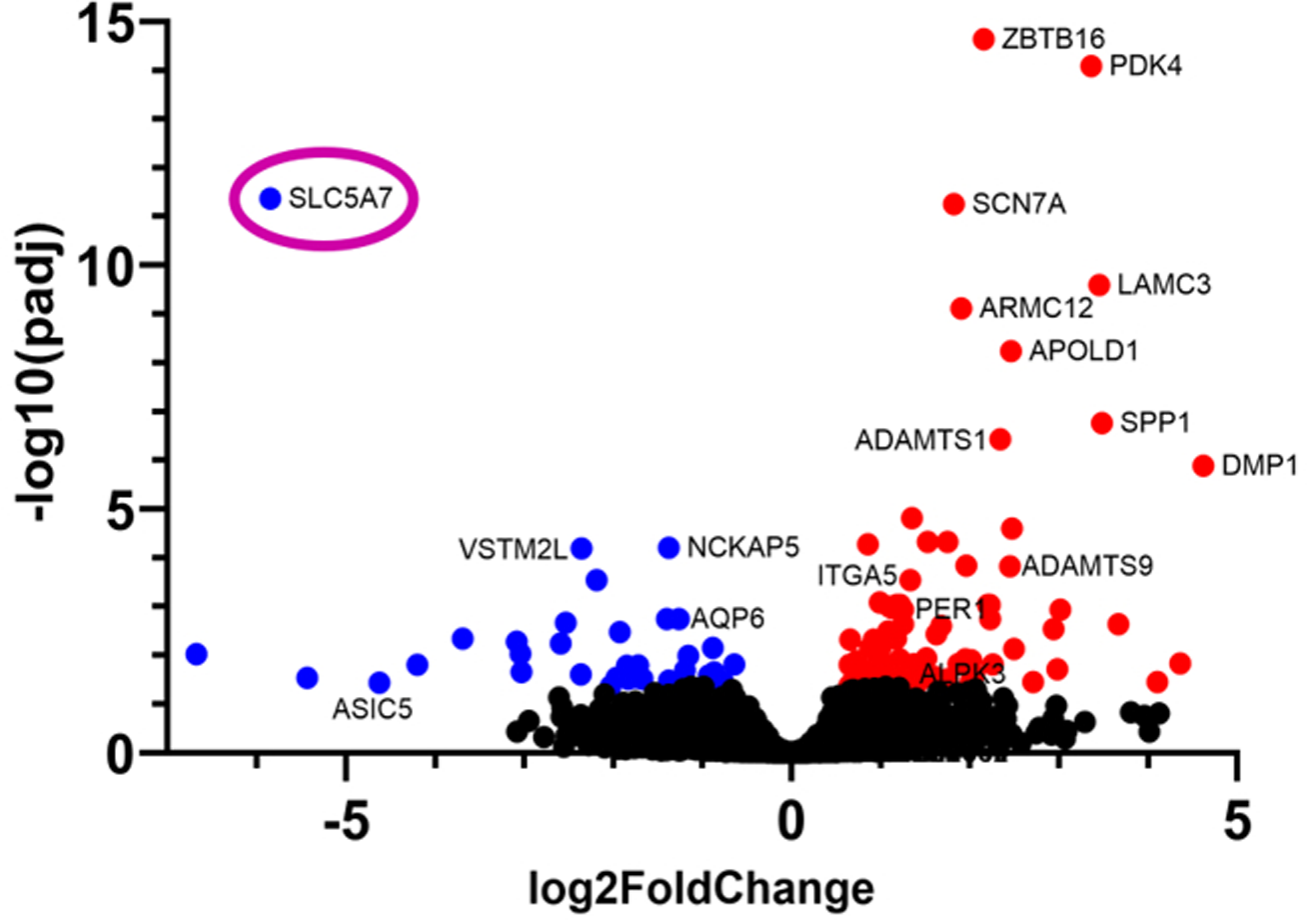
Figure 1. Rectal tissues from colitis cases exhibit multiple differentially expressed genes as compared to healthy horses. Significantly upregulated and downregulated genes in 3 colitis horses as compared to 3 healthy horses are identified by color (red and blue, respectively) based on an adjusted *p* value <0.05 and absolute log2 fold change > 1. A downregulated gene of interest, SLC5A7, is circled.

**Figure:**
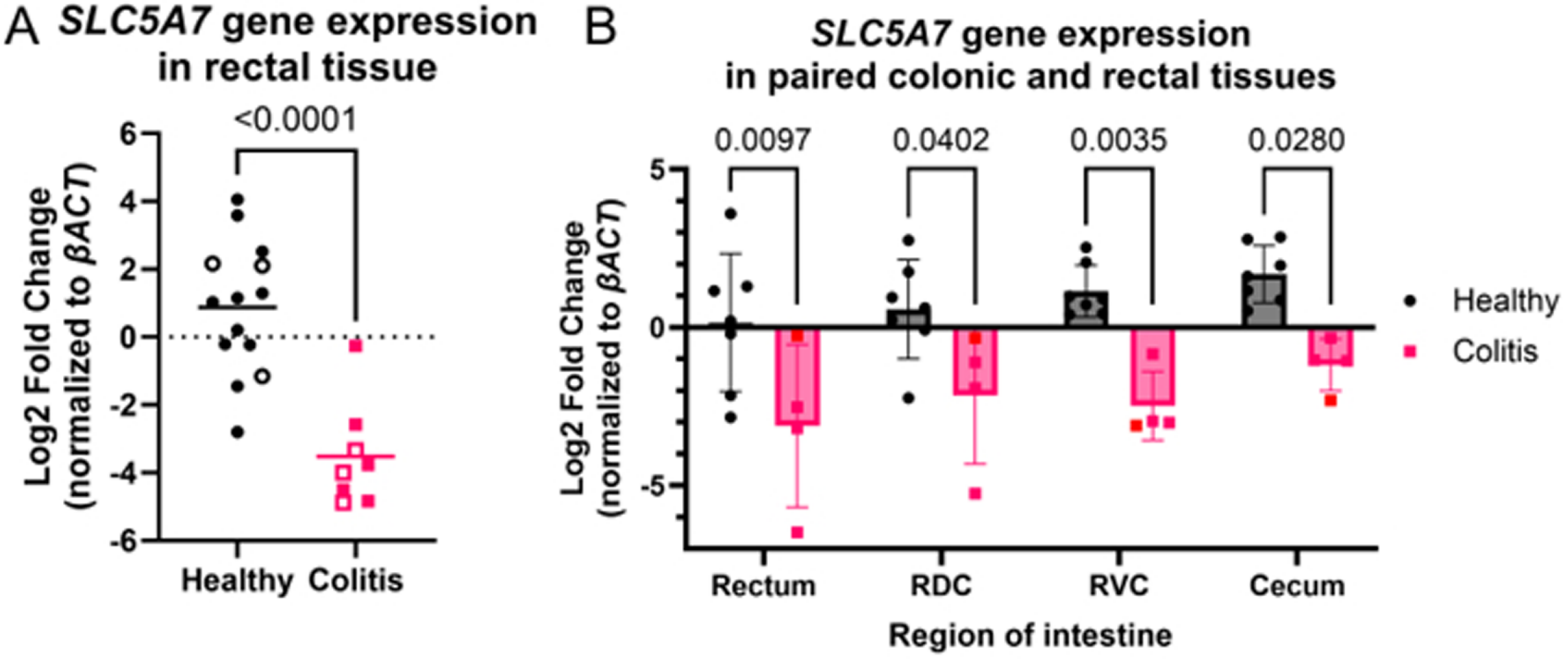
**Figure 2. SLC5A7 is broadly downregulated in colonic and rectal tissues from colitis cases**. (A) gene expression via qRT‐PCR of SLC5A7 is significantly decreased in rectal tissues of colitis cases in a larger data set. Clear circles/squares indicate validation of RNA‐sequencing results (same cases for both sequencing and qRT‐PCR). Significance (*p* < 0.05 determined by unpaired t‐text. (B) gene expression via qRT‐PCR of SLC5A7 is significantly decreases in rectal, RDC, RVC and cecal tissues from colitis cases (*n* = 4) as compared to clinically healthy horses (*n* = 7). Significance (*p* < 0.05) determined by two‐way ANOVA followed by Bonferroni's *post hoc* test.

**Conclusions and Clinical Importance:** Colitis cases demonstrated a shared transcriptome in rectal mucosa/submucosa distinct from clinically healthy horses. SLC5A7 is unexplored in equine colitis but the downregulation observed throughout the colon suggests it may be useful for future diagnostics or therapeutics.

## 104 ABSTRACT E64: Orthodromic Atrioventricular Re‐Entry Bradycardia and Tachycardia in Horses Due to an Accessory Pathway

### 104.1 **Gunther van Loon** ^1^, DVM, DECEIM, ECVDI (Assoc); Eva Buschmann^2^; Hans De Wilde^3^; Annelies Decloedt^2^; Mary Durando^4^; Charlotte Easton‐Jones^5^; Celia Marr^5^; Veronica Roberts^6^; Glenn Van Steenkiste^2^

#### 104.1.1 ^1^Head of Equine Cardioteam, Ghent and Equine Internal Medicine, Ghent University; ^2^Equine Cardioteam Ghent, Ghent University; ^3^Department of Cardiology, UZ Ghent; ^4^Equine Sports Medicine Consultants; ^5^Rossdales Equine Hospital and Diagnostic Centre; ^6^Bristol Veterinary School, University of Bristol

**Background:** Accessory pathways (AP) are muscular bundles connecting atria with ventricles, bypassing the atrioventricular (AV) node‐His‐Purkinje system. Anterograde AP conduction results in ventricular pre‐excitation. Retrograde conduction allows ventriculo‐atrial (VA) conduction, thereby creating a potential re‐entry circuit for orthodromic atrioventricular re‐entry tachycardia (OAVRT).

**Objectives:** Describe clinical and electrocardiographic findings of horses with retrograde AP conduction, and in one horse, mapping and radiofrequency ablation results.

**Animals:** Six horses with retrograde AP.

**Methods:** Records from 6 horses were reviewed.

**Results:** The resting electrocardiogram showed P’ waves in the ST segment with a fixed RP’ coupling interval in all horses. In 5 horses, these P’ waves were conducted back to the ventricles along the AV node, resulting in OAVRT with a median heart rate of 94 [74–145] beats per minute (bpm). All horses also showed P’ waves blocked at the AV node, resulting in orthodromic atrioventricular re‐entry bradycardia (OAVRB) due to sinus node resetting, with a median heart rate of 27 [21–37] bpm. In one horse, bi‐directional AP conduction also resulted in ventricular pre‐excitation. In one horse, 3D mapping identified a left atrial insertion of the AP by the shortest VA interval. Radiofrequency ablation without contact force was not successful in eliminating the AP.

**Conclusions:** Retrograde conducting AP resulted in OAVRT and OAVRB. Accessory pathways in these horses behaved differently compared to humans and small animals. Further research is necessary to elucidate AP behavior in horses, to evaluate the risk and effect on performance, and to assess the potential for treatment by ablation.

**Figure:**
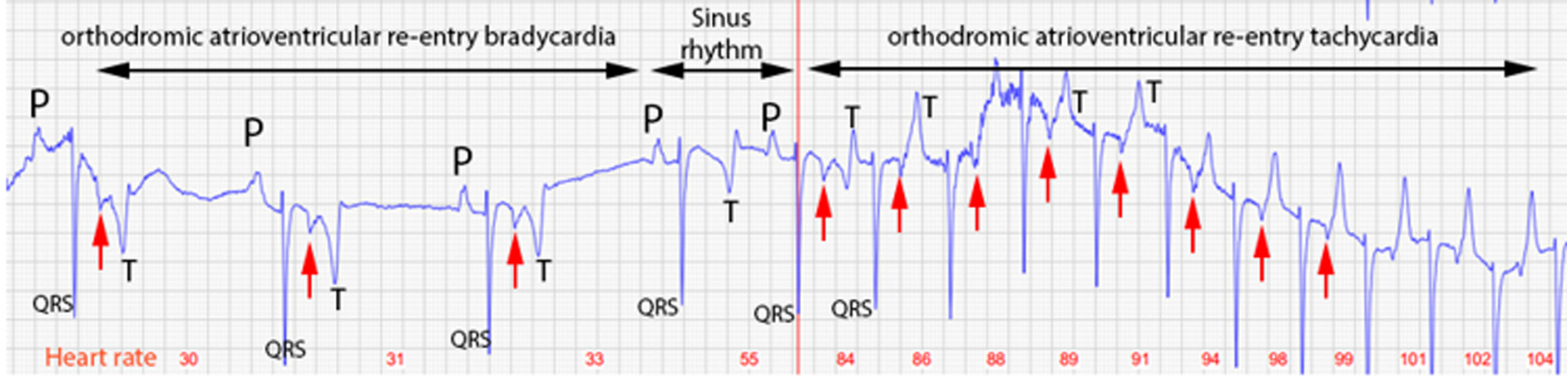
Electrocardiogram of a horse with an accessory pathway that conducts from ventricle to artium, shown by the retrograde P’ wave (red arrow). First, the retrograde conducted P’ waves are blocked at the atrioventricular node while they reset the sinus node, resulting in orthodromic atrioventricular re‐entry bradycardia (heart rate 31/min). Subsequently, during one cardiac cycle, no retrograde conduction occurs, showing a sinus rate of 55/min. After that, every retrograde P’ wave in immediately conducted to the ventricles, resulting in orthodromic atrioventricular re‐entry tachycardia, at a rate of 95/min.

## 105 ABSTRACT EN01: Determining the Prevalence of Hypersomatotropism in a North American Cohort of Diabetic Cats

### 105.1 **Lela Remington** ^1^; Kyle Martin^2^, DVM, MS, DACVS (Small Animal); Sarah Guess^3^, DVM, MS, DACVIM (SAIM); Annie Chen‐Allen^4^, DVM, MS, DACVIM (Neurology); Linda Martin^5^, DVM, MS, DACVECC; Tina Owen^6^, DVM, DACVS (Small Animal)

#### 105.1.1 ^1^Small Animal Internal Medicine Resident, Washington State University; ^2^Colorado Animal Specialty and Emergency; ^3^Assistant Professor, Internal Medicine, Washington State University; ^4^Washington State University; ^5^Associate Professor, Emergency Critical Care, Washington State University; ^6^Associate Professor, Emergent, Orthopedic & Soft Tissue Surgery, Pituitary Surgery, Washington State University

**Background:** Hypersomatotropism (HST) is a differential for persistent insulin resistance in cats with diabetes mellitus (DM). Serum IGF‐1 > 1000 ng/mL has been shown to have a high positive predictive value (95%) for the diagnosis of HST in cats. The prevalence of HST in diabetic cats has been previously described in cohorts outside of North America (14.9–24.8%).

**Hypothesis/Objectives:** To determine the prevalence of HST in a cohort of North American diabetic cats.

**Animals:** Fifty client‐owned cats with DM.

**Methods:** Prospective multicenter observational study. Sample size calculation was performed based on European prevalence data and assumptions included an overall prevalence of HST of approximately 25%±5% (SD). Diabetic cats were screened using fructosamine and sorted into “controlled” (C, ≤ 500 μmol/L) or “poorly‐controlled” (PC, > 500 μmol/L) groups. Study cats were also concurrently screened using IGF‐1. Advanced imaging (CT, MRI) was completed on all cats with an IGF‐1 > 1000 ng/mL.

**Results:** Nine of 50 cats had a IGF‐1> 1000 ng/mL. All (9/9) cats screened using advanced imaging met the criteria for a pituitary lesion. Majority (7/9) of the cats with pituitary lesions were castrated male domestic shorthair with an overall median age of 10.0 years (7.8–13.8). The positive predictive value of an IGF‐1 > 1000 ng/mL for a pituitary lesion was 100%.

**Conclusions and Clinical Importance:** This study determined a prevalence of 18% for HST in a small cohort of North American client‐owned diabetic cats.

## 106 ABSTRACT EN02: Dehydroepiandrosterone Sulfate (DHEAS) as a Potential Biomarker of the Etiology of Canine Cushing's Syndrome

### 106.1 **Rodolfo Oliveira Leal** ^1,5^; Inês Oliveira^2^; Patricia Lunet Marques^3,5^; Luísa Mateus^3,5^; Francesca Del Baldo^4^; MariaChiara Re^4^; Luís Capela^3,5^; Telmo Nunes^3^

#### 106.1.1 ^1^Invited Assistant Professor, Centre for Interdisciplinary Research in Animal Health (CIISA), Faculty of Veterinary Medicine, University of Lisbon; Lisbon, Portugal; ^2^HEV, Veterinary Teaching Hospital, Faculty of Veterinary Medicine, University of Lisbon, Portugal; ^3^Centre for Interdisciplinary Research in Animal Health (CIISA), Faculty of Veterinary Medicine, University of Lisbon; Lisbon, Portugal; ^4^Department of Veterinary Medical Sciences, Alma Mater Studiorum, University of Bologna, Ozzano Emilia, Italy; ^5^Associate Laboratory for Animal and Veterinary Sciences (AL4AnimalS), Lisboa, Portugal

**Background:** In humans, dehydroepiandrosterone sulfate (DHEAS) measurement is an alternative biomarker to endogenous ACTH (eACTH), commonly used to identify Cushing's Syndrome (CS) etiology, being normal/increased in pituitary‐dependent hypercortisolism (PDH) and decreased in adrenal‐dependent hypercortisolism (ADH). In dogs, information about DHEAS is scarce.

**Objectives:** To assess the role of DHEAS concentrations in determining CS etiology in dogs.

**Animals:** Thirty‐eight client‐owned cushingoid dogs with established diagnosis (PDH versus ADH) based on functional endocrine tests, eACTH measurement and diagnostic imaging.

**Methods:** Multicenter, retrospective, cross‐sectional study. Dogs were grouped according to CS etiology and thereafter based on sex and neutered status. DHEAS was measured with a species‐specific ELISA kit using stored serum samples (−80°C).

**Results:** Of the 38 dogs, 33/38 (87%) were diagnosed with PDH and 5/38 (13%) with ADH. There were 19 females and 19 males, of which 16 and 9 were neutered, respectively. Median DHEAS concentrations were significantly higher in intact males (2.45 ng/mL; interquartile range 1.02–7.80 ng/mL) than in neutered males (0.68 ng/mL; interquartile range 0.42–2.03 ng/mL; *p* = 0.017). Females showed a positive correlation between DHEAS and eACTH levels (*r* = 0.588; *p* = 0.008). ROC curve analysis demonstrated poor ability of DHEAS to differentiate PDH from ADH (AUC 0.430) and the Mann‐Whitney test showed no significant differences in DHEAS levels between these two causes (*p* = 0.645).

**Conclusions and Clinical Importance:** Despite some differences arising from sex and neutered status, DHEAS measurement does not appear to be a relevant biomarker for differentiating PDH from ADH in dogs with CS.

## 107 ABSTRACT EN03: Evaluation of Hypoglycemic and Hyperglycemic Events in Non‐Diabetic Dogs Using a Flash Glucose Monitoring System

### 107.1 **Rebecca Brisman** ^1^; Douglas Palma^2^, DVM, DACVIM (SAIM); Philip Fox^3^, DVM, DACVIM/DECVIM‐CA (Cardiology), DACVECC

#### 107.1.1 ^1^Small Animal Rotating Intern, Schwarzman Animal Medical Center; ^2^Specialist in Internal Medicine, The Schwarzman Animal Medical Center; ^3^Service Head of Cardiology, Cardiology, The Schwarzman Animal Medical Center

**Background:** Flash glucose monitoring systems (FGMS; FreeStyle Libre, Abbott) are convenient and useful devices intended to aid in and improve the management of diabetic patients. The FGMS is reportedly accurate for diabetic dogs with hyperglycemia and euglycemia but tends to underestimate glucose concentrations during hypoglycemia.

**Hypothesis/Objectives:** Assess to what extent hypoglycemia, hyperglycemia, and euglycemia are detected in healthy, non‐diabetic dogs using a FGMS.

**Animals:** Sixteen healthy hospital employee‐owned dogs.

**Methods:** Prospective, observational study. A FGMS was attached to 16 adult dogs to record at least 488 readings each for up to 14 days. Device‐measured interstitial glucose (IG) concentrations recorded the frequency of hypoglycemia, euglycemia, and hyperglycemia.

**Results:** 13,067 total IG samples were analyzed. During monitoring, 69% (11/16) had at least one hypoglycemic event (IG < 70 mg/dL), while 25% (4/16) had at least one hyperglycemic event (> 180 mg/dL). The mean percentage of total hypoglycemic and hyperglycemic readings per individual was 3% (range 0%–14%) and 0.8% (range 0%–8%), respectively. Marked hypoglycemia (IG < 55 mg/dL) and hyperglycemia (IG > 250 mg/dl) were identified at least one time in 56% (9/16) and 13% (2/16), respectively. When comparing body weight cohorts (2.5–20.5 kg vs 20.6–41.4 kg), the frequency of hypoglycemic events differed significantly between groups (*p* = 0.021), with the smaller dog cohort having a higher median frequency of hypoglycemia than the larger dog cohort.

**Conclusions and Clinical Importance:** Both hypoglycemia and hyperglycemia were recorded by FGMS in healthy dogs. These findings might inform treatment recommendations in diabetic patients and require further evaluation in a larger population.

## 108 ABSTRACT EN04: Autoimmune Polyglandular Syndrome Type II in Dogs: A Review of 51 Cases

### 108.1 **Yeon Joon Park** ^1^; Craig Breheny^2^, BVM&S DECVIM‐CA, SFHEA, MRCVS; Andre Kortum^3^, BVSc, MANZCVS (SAM), SFHEA, DECVIM, MRCVS; Niamh Duthie^4^; Maribel Navarro Espín^5^; Sandra Rosário^6^; Erin O’Connell^7^, BVSc, DECVIM‐CA, FHEA, MRCVS

#### 108.1.1 ^1^ECVIM Resident in Internal Medicine, University of Liverpool; ^2^Senior Lecturer in Emergency & Critical Care, The Royal (Dick) School of Veterinary Studies; ^3^Associate Teaching Professor in Small Animal Medicine, Dept of Veterinary Medicine, University of Cambridge; ^4^Small Animal Veterinary Rotating Intern, Queen Mother Hospital for Animals, The Royal Veterinary College; ^5^Small Animal Medicine Intern, University College Dublin; ^6^University of Liverpool; ^7^Senior Lecturer in Small Animal Internal Medicine, University of Liverpool

**Title:** Suspected Autoimmune Polyglandular Syndrome Type II in 51 Dogs

**Background:** Autoimmune polyglandular syndrome type II (APS‐II) is characterized by the co‐occurrence of two or three of the following: adrenal insufficiency, hypothyroidism, and diabetes mellitus (DM). While its clinicopathological features are well‐documented in humans, reports on APS‐II in dogs remain limited to individual case reports.

**Objectives:** To describe the clinical course of APS‐II in dogs.

**Animals:** Fifty‐one client‐owned dogs diagnosed with APS‐II based on clinicopathological criteria were included in this retrospective, observational cohort study.

**Methods:** Medical records from five university referral hospitals in Europe, from 2000 to 2024, were reviewed. Data included signalment, clinical signs, endocrine testing, and outcomes. Diagnosis was based on compatible clinical signs and confirmatory endocrine tests.

**Results:** Of 51 dogs, 19 (37%) had hypothyroidism and hypoadrenocorticism, 24 (47%) had hypothyroidism and DM, 7 (14%) had hypoadrenocorticism and DM, and 1 dog was diagnosed with all three. The most common initial diagnosis was DM (43%), followed by hypoadrenocorticism (33%) and hypothyroidism (23%). Median interval between the first and second diagnoses was 122 days (range 0–1636). Median age at first endocrinopathy diagnosis was 7 years (range 1–13). Median survival time was 41 months (range 1–145) from initial diagnosis with a median age of death of 12 years (range 1–15).

**Conclusions and Clinical Importance:** This study highlights the importance of recognizing polyendocrinopathies in dogs. Further research is needed to identify risk factors associated with APS‐II and how if differs from single endocrinopathies.

**Figure:**
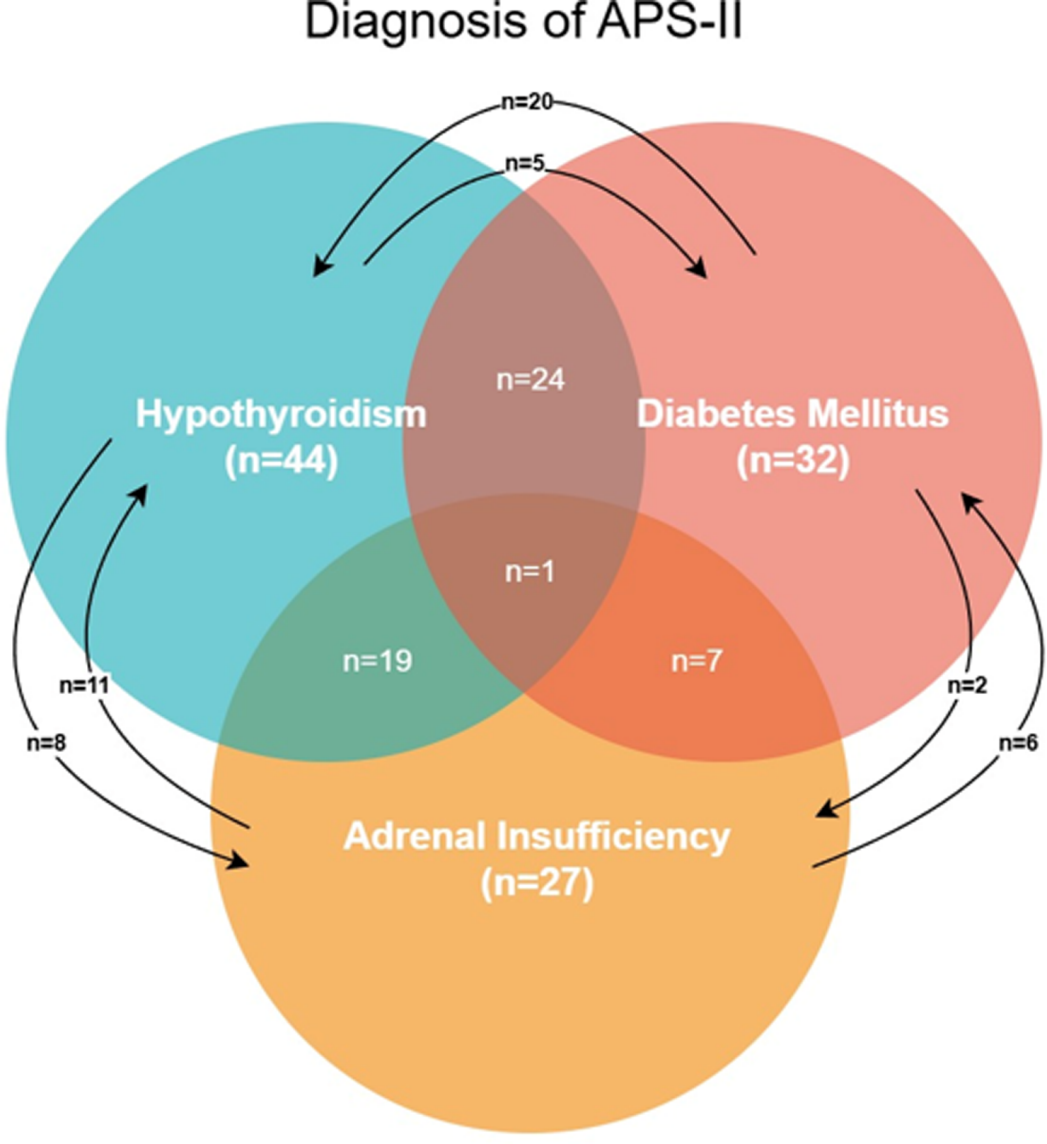
**Figure 1** A Venn diagram illustrating the distribution of endocrinopathies in dogs with autoimmune polyglandular syndrome type II (APS‐II). The arrows indicate the sequence of diagnosis.

## 109 ABSTRACT EN05: Pharmacodynamics of Insulin Lispro Assessed by Use of the Isoglycemic Clamp Method in Healthy Dogs

### 109.1 **Lauren Porter** ^1^; Chiquitha Crews^2^, MS; Chen Gilor^3^, DVM, DACVIM (SAIM), PhD

#### 109.1.1 ^1^Postdoctoral Associate, University of Florida; ^2^Biological Scientist II, University of Florida; ^3^Associate Professor, University of Florida

**Background:** Insulin lispro is a rapid acting insulin analog that is used as postprandial bolus insulin in humans. It may also be useful as a postprandial bolus insulin in dogs and has been suggested for use in the treatment of diabetic ketoacidosis (DKA), however, limited pharmacodynamic (PD) data exists.

**Objective:** To determine the PD of insulin lispro following subcutaneous injection in dogs.

**Animals:** Seven healthy, female spayed, two‐year‐old purpose‐bred beagles (median weight 8.9 kg [7.8–9.6]).

**Methods:** Each dog received 0.2 IU/kg insulin lispro subcutaneously. Isoglycemic clamps were performed by variable rate of intravenous glucose infusion. Blood glucose was measured by AlphaTrak3 every 5 min, and the glucose infusion rate (GIR) was adjusted to maintain baseline glycemic level. The GIR over time was used to create a time‐action curve. The data were evaluated for normality using the Shapiro‐Wilk Test. Data were not normally distributed and are therefore presented as median [range].

**Results:** Insulin lispro had a median onset of action of 10 min [10–15] and median duration of action of 150 min [115–300]. The median peak insulin action, defined as GIR max, was 19.1 mg/kg/min [12.9–26.9], and the median time to peak insulin action (time to GIR max) was 65 min [40–80].

**Conclusion:** The rapid onset and short duration of insulin lispro make it impractical as a postprandial insulin for dogs but suggest utility for managing DKA. Further studies are needed to evaluate the PD of insulin lispro in dogs with DKA, as disease state may alter its time‐action profile.

## 110 ABSTRACT EN06: Prior Obesity as a Risk Factor of Canine Diabetes Mellitus: A Pair‐Matched 1:3 Case‐Control Study

### 110.1 **John Flanagan** ^1^; Gabriel Kocevar^2^; Basile Reinert^2^; JoAnn Morrison^3^; Alexander German^4^

#### 110.1.1 ^1^Senior Research Scientist, Royal Canin; ^2^Seenovate; ^3^Mars Veterinary Health; ^4^University of Liverpool

**Background:** The association between obesity and diabetes mellitus (DM) in dogs remains controversial.

**Objective:** To study the potential association between prior obesity and DM in a case‐control study.

**Animals:** Electronic records from a large veterinary clinical database.

**Methods:** 6,611 diabetic dogs (cases) were identified, with 94% having at least 10 hospital visits over a defined monitoring period (median 6 y, range 4–9 y) prior to diagnosis. Three controls per case were selected using bootstrap matching, matched for breed, sex, neutered status and age. These controls had a similar frequency and duration of visit history. Body condition was categorised as underweight, ideal, overweight and obese. For statistical analysis, odds ratios (OR) were calculated, and univariate and multivariate logistic regression models were constructed.

**Results:** The odds of developing DM and body condition were significantly associated, with OR of 0.39 (0.15–0.83 95% CI), 2.05 (1.91–2.20) and 4.09 (3.79–4.41) for the underweight, overweight and obese categories, respectively, compared with the ideal category. There was an interaction between sex and body condition, whereby the odds of developing DM were greater in female compared with male dogs in obese condition, but greater in male compared with female dogs in ideal and overweight condition. Finally, there was a strong positive association between duration of time spent in obese condition and the OR for developing DM (Figure).

**Conclusions and Clinical Importance:** A strong temporal association exists between prior obesity and the development of DM in pet dogs. These results can help guide future research in this area.

**Figure:**
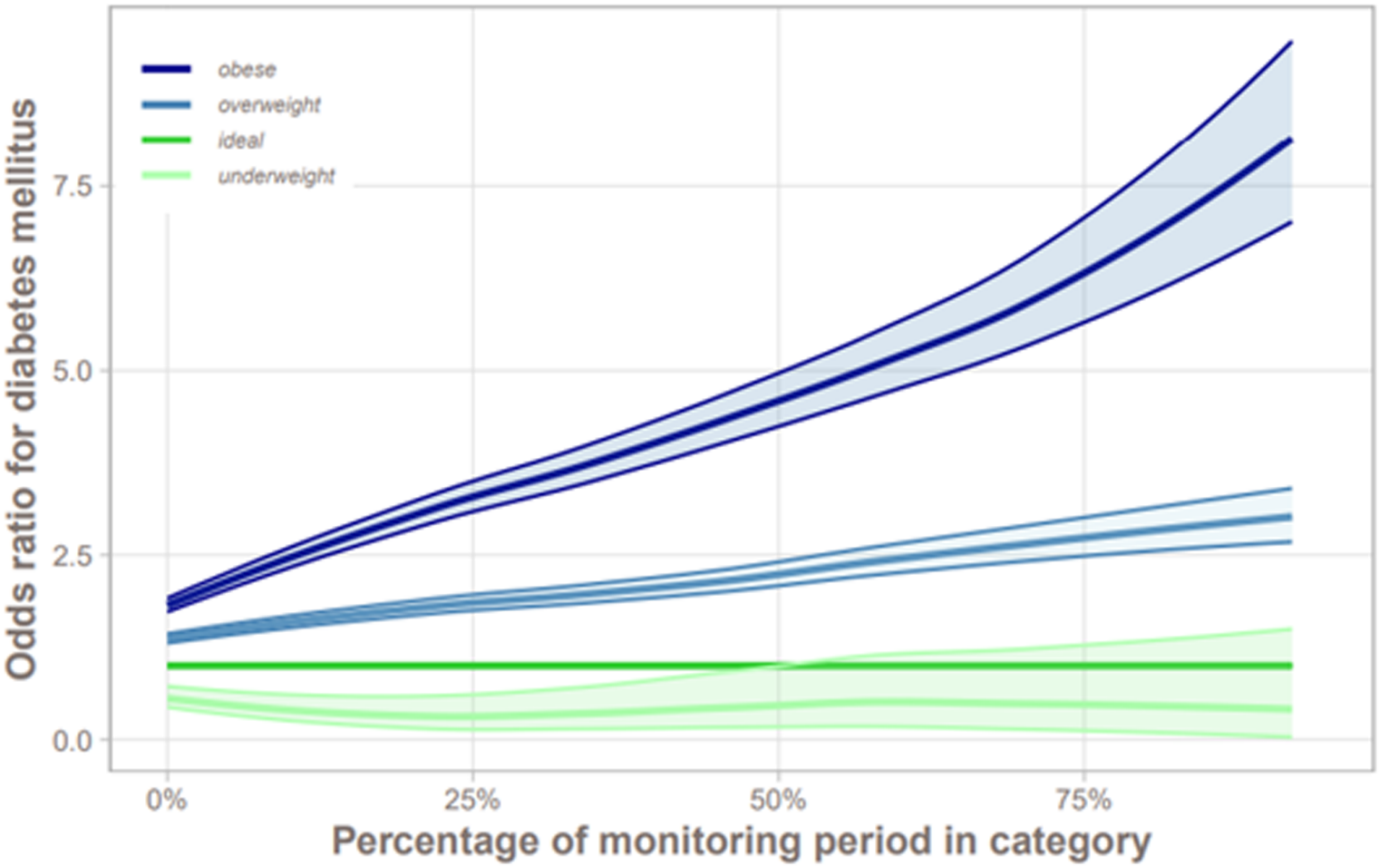
**Figure** Relationship between time spent in different body condition categories and odds ratios for diabetes mellitus in dogs; ideal body condition serves as the reference category.

## 111 ABSTRACT EN07: Endogenous ACTH as an Aid for Primary Hypoadrenocorticism Diagnosis After Glucocorticoid Treatment Beginning

### 111.1 **Álan Pöppl** ^1^, DVM, MSc, DSc; Vitória Wodzik^2^, DVM; Karine Leal^3^, DVM, MSc.; Stefani Carravetta^4^; Andrielly Da Silva^4^; Ana Paula Fogliatto^2^, DVM; Aline Moure^4^

#### 111.1.1 ^1^Professor, Universidade Federal do Rio Grande do Sul, Porto Alegre, Brazil; ^2^Master Degree Student, PPGCV, UFRGS; ^3^PhD Student, PPGCV, UFRGS; ^4^Graduate Student, FAVET, UFRGS

**Background:** ACTH stimulation test (ACTHST) is the gold standard for primary hypoadrenocorticism (PHA) diagnosis. Cortisol/ACTH ratio can be applied alternatively due the high endogenous ACTH (eACTH) levels in those cases. Adrenal crisis severity and eventual difficulties with synthetic ACTH availability and eACTH measurement may lead to the beginning of glucocorticoid therapy (GCT) in suspect cases before PHA confirmation, impairing the interpretation of confirmatory tests.

**Hypothesis/Objectives:** Dogs with PHA don´t suppress eACTH within reference range (5–45 pg/mL) after GCT. Increased eACTH levels in dogs on GCT argues in favor of PHA diagnosis in suspected cases.

**Animals:** Twenty‐three dogs with previous confirmed PHA by the ACTHST and twelve controls submitted to GCT.

**Methods:** Cross‐sectional study of dogs with PHA to measure plasma eACTH (Immulite, Siemens), and prospective comparison of eACTH before (T0) and after 5–10 days (T1) on GCT in the controls. Samples from the PHA group and control dogs in T1 were collected at least 18 h after the last GC administration. Clinical signs, hematological, and serum biochemistry analysis were also compared. Results are expressed as median and range.

**Results:** Median eACTH (485; 21.5–1250 pg/mL) from PHA dogs was higher (*p* < 0.0001) than controls in T1 (9.63; 1–34.4 pg/mL). The GCT significantly decreased eACTH from T0 (20.3; 1–142 pg/mL, *p* = 0.0488). The PHA dogs were receiving less (*p* < 0.0001) prednisone (0.075; 0–1.78 mg/kg/q24h) than controls (0.5; 0.35–1.29 mg/kg/q24h).

**Conclusions and Clinical Importance:** Low‐dose GCT doesn´t normalize eACTH in PHA, a finding that may aid confirming this suspicion in dogs already on GCT.

**Figure:**
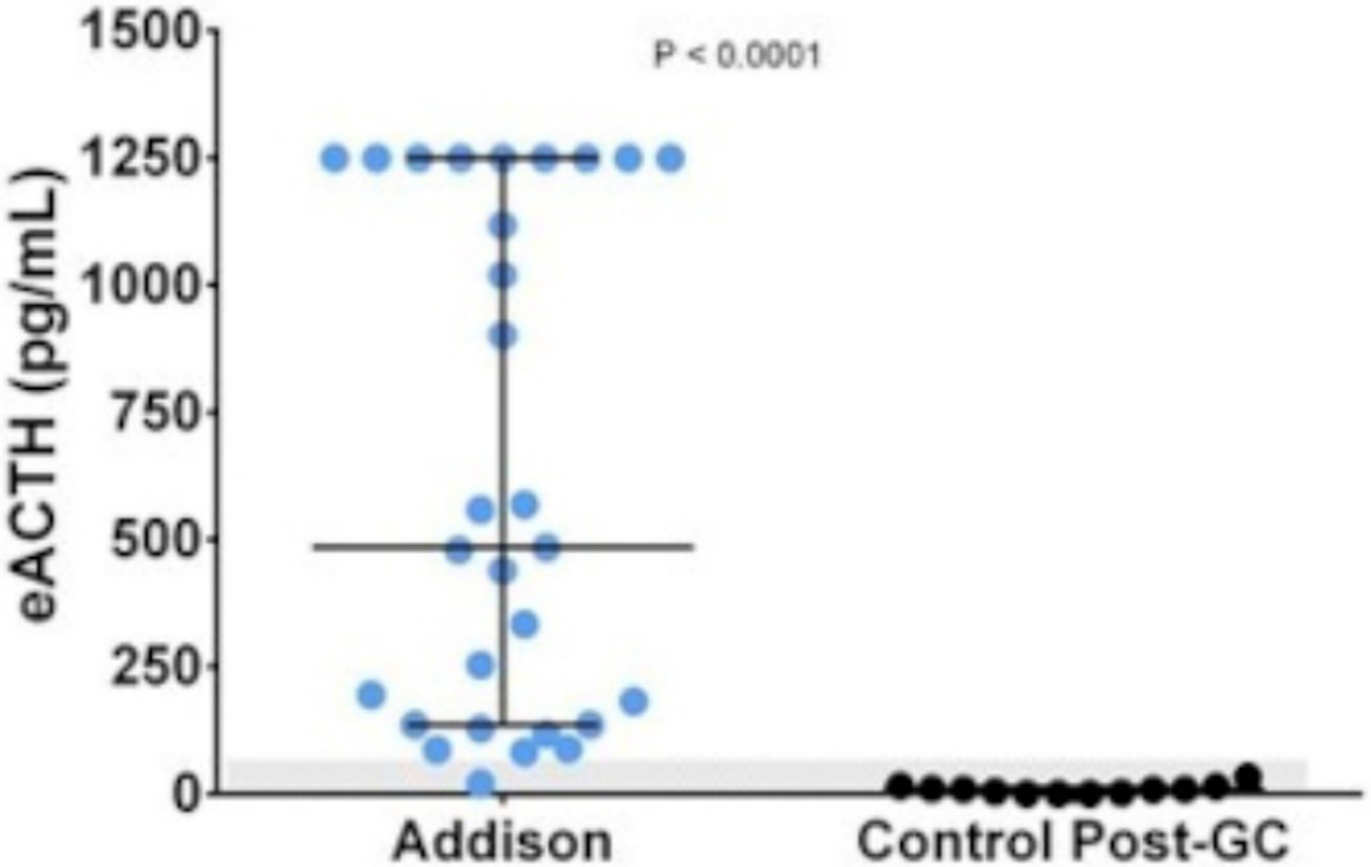
**Figure 1**. Endogenous ACTH in dogs with previously confirmed primary hypoadrenocorticism receiving glucocorticoid treatment (median prednisone dose: 0.075 mg/kg q 24 h, range: 0–1.78) and in control dogs exposed to glucocorticoid treatment (median prednisone dose: 0.5 mg/kg q 24 h, range 0.35–1.29) *Gray zone represents reference interval for eACTH

## 112 ABSTRACT EN08: Free Thyroxine Concentration by Chemiluminescent Immunoassay and Equilibrium Dialysis in Euthyroid Dogs with Nonthyroidal Illness

### 112.1 **Maxime Derre** ^1^, DVM; Timothy Bolton^2^; Christina Marino^3^; George Moore^2^

#### 112.1.1 ^1^Third Year Internal Medicine Resident, Purdue University, West Lafayette, IN, USA; ^2^Purdue University, West Lafayette, IN, USA; ^3^Small Animal Veterinary Specialists

**Background:** Free thyroxine (fT4) measured by chemiluminescent immunoassay (fT4CLIA) and equilibrium dialysis (fT4ED) in known euthyroid dogs with nonthyroidal illness (NTI) needs investigation.

**Objectives:** (1) To determine the agreement between fT4CLIA and fT4ED in serum samples from known euthyroid dogs with NTI, (2) To evaluate the diagnostic sensitivity (correctly classify a dog as euthyroid) of each fT4 assay when TT4 concentration is normal and low, and 3) to evaluate concordant and discordant fT4CLIA and fT4ED results.

**Animals:** Thirty‐eight euthyroid dogs with NTI yielding 116 serum samples.

**Methods:** Method comparison study. In 116 serum samples, serum fT4 concentration was measured by fT4CLIA and fT4ED, and agreement was assessed by a Bland‐Altman plot. In 38 dogs, the diagnostic sensitivity of each fT4 assay was calculated as the proportion of dogs having a normal serum fT4 concentration when the serum TT4 concentration was normal and low.

**Results:** Assay comparison showed agreement, with no significant systematic or proportional bias (Figure 1). The diagnostic sensitivities of the fT4CLIA and fT4ED were 94% (95% confidence interval (CI), 69.8%–99.8%) and 100% (95% CI, 79.4%–100%), respectively, when the serum TT4 concentration was normal, and 45% (95% CI, 24.4%–67.8%) and 41% (95% CI, 20.7%–63.6%), respectively, when the serum TT4 concentration was low. The fT4CLIA and fT4ED were concordant in 77% of dogs (Figure 1).

**Conclusions and Clinical Importance:** In dogs with NTI having a low serum TT4 concentration, fT4CLIA and fT4ED should be interpreted cautiously as they are likely to be low and incorrectly classify a dog as hypothyroid.

Assay comparison showed agreement, with no significant systematic or proportional bias. The diagnostic sensitivities of the fT4CLIA and fT4ED were 94% (95% confidence interval (CI), 69.8%–99.8%) and 100% (95% CI, 79.4%–100%), respectively, when the TT4 concentration was normal, and 45% (95% CI, 24.4%–67.8%) and 41% (95% CI, 20.7%–63.6%), respectively, when the TT4 concentration was low. The fT4CLIA and fT4ED were concordant in 77% of dogs.

**Figure:**
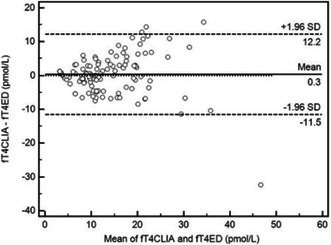
**Figure 1. Bland‐Altman plot depicting the comparison of means to differences between methods (ED and CLIA) of free thyroxine measurement**. The open circles represent the 116 serum samples. The solid line represents the arithmetic mean difference. The dashed lines represent the 95% limits of agreement. The dotted line would indicate perfect agreement between methods. ED, equilibrium dialysis; CLIA, chemiluminescent immunoassay.

**Figure:**
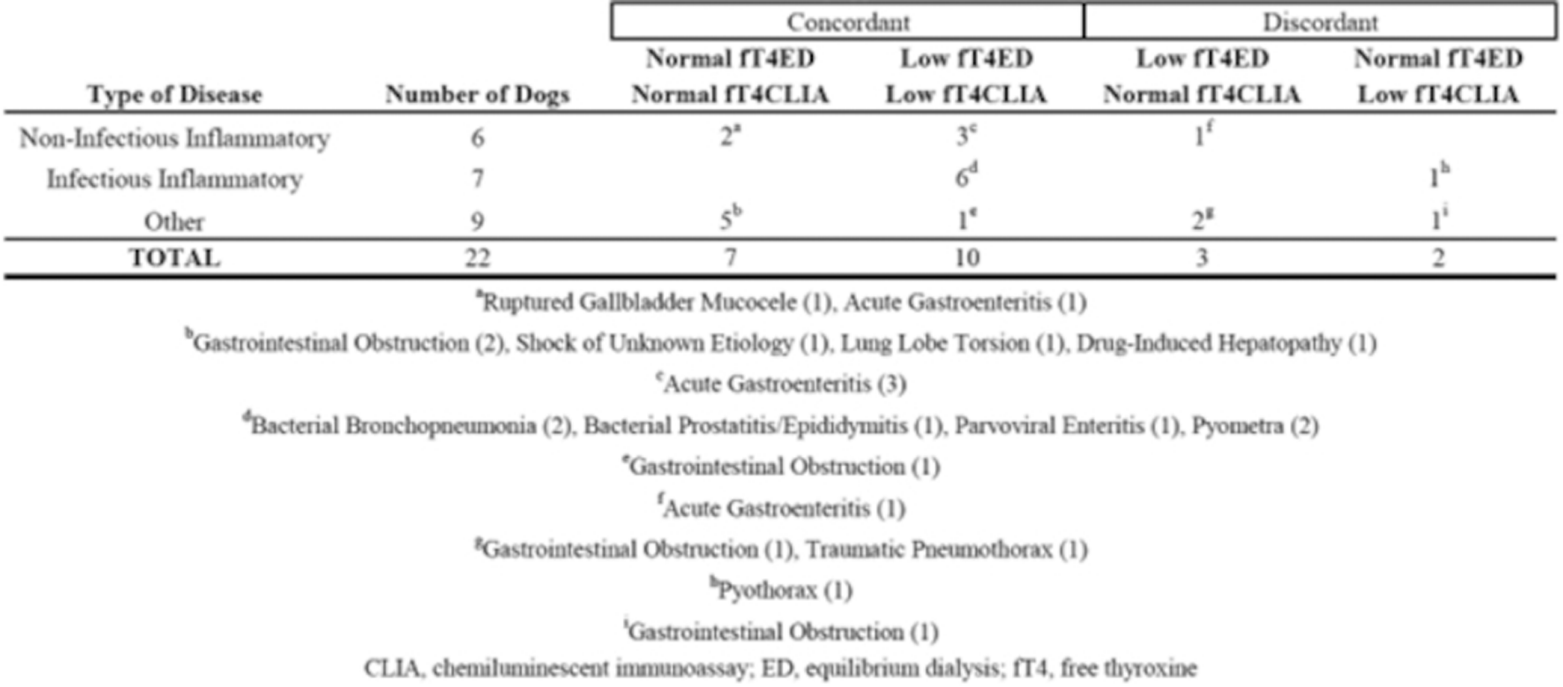
Table 1. Concordant and discordant fT4CLIA and fT4ED results, stratified by type of disease, in dogs with a low serum TT4 concentration

## 113 ABSTRACT EN09: Hypersomatotropism Low‐Prevalence Among Diabetic Cats from Porto Alegre City in Southern Brazil: Preliminary Report

### 113.1 **Álan Pöppl** ^1^, DVM, MSc, DSc; Fernanda Gouvêa^2^; Raquel Guarise^3^, DVM; José Lucas Lopes^4^, DVM; Bruna Machado^2^; Tais Nogueira^5^, DVM, MSc.; Luana Rodrigues^4^, DVM; Diana Tramujas^6^, DVM

#### 113.1.1 ^1^Professor, Universidade Federal do Rio Grande do Sul; ^2^Graduate Student, FAVET, UFRGS; ^3^CEO, SoundVet Solutions; ^4^Master Degree Student, PPGCV, UFRGS; ^5^PhD Student, PPGCV, UFRGS; ^6^SoundVet Solutions

**Background:** Hypersomatotropism (HST) is documented in ~15–33% of diabetic cats in different studies. The first Latin American epidemiological trial on HST prevalence found 14.9% HST prevalence among diabetic cats from Buenos Aires (Argentina).

**Hypothesis/Objectives:** The HST prevalence among diabetic cats from Porto Alegre city, Southern Brazil, correlates with the reported prevalence in Buenos Aires city, about 850 km distant.

**Animals:** A sample size of 33 patients was calculated (Epi Info Calculator) to find a HST prevalence of 15% among diabetic cats considering a 95% alfa and 80% power.

**Methods:** Diabetic cats on insulin therapy for at last two months, without Cushing´s syndrome or hyperthyroidism were eligible. Patients were recruited by convenience among feline and endocrinology practitioners and submitted to a clinical, phenotypical, and laboratorial evaluation. Total T4 and IGF‐1 were measured by the Immulite 2000 system (Siemens). A cut‐off of IGF‐1 > 746 ng/mL was assumed for HST diagnosis. Cats with IGF‐1 higher than the upper limit of the reference interval (670 ng/mL) are invited to perform a pituitary CT scan.

**Results:** Until this moment, 26 cats were evaluated, being 92.3% mixed breed and 65.4% males (90% neutered). The mean age was 10.7±4.28 (2.5–22) years, and mean weight 5.4±1.42 (2.85–9.4) kg. Mean IGF‐1 among this population was 277±134 (79.5–497) ng/mL.

**Conclusions and Clinical Importance:** So far, the HST lower prevalence in this population raises questions regarding geographical differences in disease distribution, eventually due to different genetic and/or environmental backgrounds.

## 114 ABSTRACT EN10: Risk Factors Associated with Elevated Serum Specific Canine Pancreatic Lipase in Dogs with Hypercortisolism

### 114.1 **Chaeyeon Park** ^1^, DVM; Minju Baek^2^; Yeon Chae^2^; Taesik Yun^2^; Byeong‐Teck Kang^2^; Hakhyun Kim^2^

#### 114.1.1 ^1^Veterinary Student, Laboratory of Veterinary Internal Medicine, College of Veterinary Medicine, Chungbuk National University, Cheongju, South Korea; ^2^Laboratory of Veterinary Internal Medicine, College of Veterinary Medicine, Chungbuk National University, Cheongju, South Korea

**Background:** Hypercortisolism (HC) is a common endocrine disorder in dogs that is often associated with pancreatitis. However, the relationship between cortisol concentration, pancreatic injury (PI) markers, and serum specific canine pancreatic lipase (Spec cPL) elevation remains unclear.

**Objectives:** To investigate the prevalence of PI, identify risk factors for elevated Spec cPL, and evaluate changes in Spec cPL levels following treatment.

**Animals:** Seventy‐one client‐owned dogs diagnosed with naturally‐occurring HC between 2015 and 2024.

**Methods:** Dogs were classified as PI (Spec cPL ≥ 400 μg/L) or non‐PI (Spec cPL < 400 μg/L). Clinical and laboratory parameters, including abdominal ultrasonography findings, were analyzed. Risk factors were identified using logistic regression analysis.

**Results:** The PI comprised 16 dogs (22.5%), with 7 (43.7%) showing ultrasonographic signs of pancreatitis. Univariate analysis showed significant inter‐group differences in gamma‐glutamyl transferase, blood urea nitrogen (BUN), symmetric dimethylarginine, cholesterol, C‐reactive protein, urine protein‐to‐creatinine ratio (UPC), and post‐low‐dose dexamethasone suppression test (LDDST) cortisol levels. Multivariate analysis further identified BUN (odds ratio [OR], 1.051; *p* = 0.015), UPC (OR, 1.853; *p* = 0.021), and post‐LDDST cortisol (OR, 1.560; *p* = 0.011) as risk factors. Trilostane treatment significantly reduced the post‐ACTH cortisol and Spec cPL levels.

**Conclusions and Clinical Importance:** PI is common in dogs with HC, and elevated post‐LDDST cortisol, BUN, and UPC levels are significant risk factors. Chronic HC may cause PI by reducing pancreatic perfusion and inducing inflammation. Elevated Spec cPL concentrations, even without clinical or ultrasonographic evidence of pancreatitis, require cautious interpretation along with clinical signs and imaging to mitigate misdiagnosis.

## 115 ABSTRACT EN11: Machine Learning Algorithms as a Screening Tool for Hypercortisolism in Dogs

### 115.1 **Yunjin Sung** ^1^, DVM; Young‐Jae Yoo^2^; Kyungchang Jeong^3^; Hanbit Seo^3^; Ha‐Suk Nam^2^; Yeon Chae^2^; Taesik Yun^2^; Byeong‐Teck Kang^2^; Euijong Lee^3^; Hakhyun Kim^2^

#### 115.1.1 ^1^Veterinary Student, Laboratory of Veterinary Internal Medicine, College of Veterinary Medicine, Chungbuk National University, Chungcheongbuk‐do, South Korea; ^2^Laboratory of Veterinary Internal Medicine, College of Veterinary Medicine, Chungbuk National University, Chungcheongbuk‐do, South Korea; ^3^School of Computer Science, Chungbuk National University, Chungcheongbuk‐do, South Korea

**Background:** Hypercortisolism (HC) is a common endocrine disorder in dogs that can significantly reduce the quality of life in affected animals. However, diagnosing HC is often challenging due to its variable clinical presentation, making it difficult to identify suitable candidates for further diagnostic tests for HC could be difficult.

**Hypothesis/Objectives:** To develop machine learning algorithms to assist in the diagnosis of HC using readily available routine tests.

**Animals:** 153 client‐owned control dogs (suspected of HC but later excluded based on hormonal test results) and 152 dogs diagnosed with confirmed HC.

**Methods:** This case‐control study utilized routine laboratory data, including complete blood count (CBC), serum chemistry panel, and urinalysis parameters such as urine specific gravity (USG) and urine protein‐to‐creatinine ratio (UPC). A boosted tree algorithm (Gradient Boosting) was trained using 80% of the collected data, while the remaining 20% was used for test data performance evaluation.

**Results:** The developed model demonstrated an accuracy of 88.5% (95% CI, 80.5–96.5%), a sensitivity of 83.3% (95% CI, 70.7%–96.7%), a specificity of 93.5% (95% CI, 84.9%–100%), and an area under the receiver operating characteristic curve (AUC) of 0.912 (95% CI, 0.835%–0.988), indicating excellent discriminatory ability between HC and controls. A user‐friendly graphical interface was developed to enable practitioners to apply this tool for HC screening easily.

**Conclusions and Clinical Importance:** The developed machine learning algorithms have the potential to improve diagnostic efficiency and owner satisfaction by identifying appropriate candidates for HC testing and reducing unnecessary diagnostic procedures.

## 116 ABSTRACT EN12: Evaluation of Serum Insulin Measurement in Dogs: A New Perspective

### 116.1 **Priscila Viau** ^1^, MV, MSc, PhD; Fernanda Martins^2^; Rodrigo Dos Santos^3^; Debora Cattaruzzi^4^, MV, MSc, PhD

#### 116.1.1 ^1^CEO, Hormonalle; ^2^Technician, Laboratory, Hormonalle; ^3^Veterinary, Laboratory, Pesquisas Hormonais; ^4^CFO, Laboratory, Pesquisas Hormonais

Accurate insulin quantification is essential in veterinary endocrinology for diagnosing insulinoma and assessing insulin resistance in dogs. The gold standard, radioimmunoassay (RIA), offers high analytical precision but requires up to 48 h for results, delaying clinical decisions. Faster methodologies could enhance diagnostic efficiency and patient management. This study validated a modified magnetic immunoassay (PMI) for canine insulin measurement by comparing its analytical performance with RIA and assessing its reliability for same‐day results. Fifty‐one dogs were categorized into two groups: 42 healthy dogs (normal insulin range: 6–32 μU/mL) and 9 dogs with confirmed insulinoma (insulin ≥ 70 μU/mL; blood glucose < 50 mg/dL). Serum samples were collected from clinical evaluations at the Hormonalle® Laboratory (São Paulo, Brazil). Insulin concentrations were determined using RIA (Millipore Sigma Human Insulin Kit, St. Louis, MO, USA) and PMI (magnetic immunoassay system, DiaSorin, Saluggia, Italy). For healthy dogs, mean insulin concentrations were 19.71±6.13 μU/mL (RIA) and 18.64±5.94 μU/mL (PMI; *p* = 0.47, *r* = 0.98). In insulinoma cases, RIA measured 99.76±24.35 μU/mL and PMI 96.12±23.5 μU/mL (*p* = 0.19, *r* = 0.94). No statistically significant differences were observed. PMI showed concordance with RIA while offering faster processing. Same‐day results enhance clinical workflows and enable timely decisions, particularly for urgent cases such as insulinoma diagnosis. These advantages position PMI as a practical and reliable tool for routine endocrine assessments in veterinary practice.

**Table:** 

Group	Mean ± SD (RIA)	Mean ± SD (PMI)	Min**–**max (RIA)	Min**–**max (PMI)	*p*	Correlation (r)
Normal range *n* = 42	19.71±6.13	18.64±5.94	11.76±31.63	11.20±31.90	0.47	0.98
Insulinoma range *n* = 9	99.76±24.35	96.12±23.5	63.74±130.40	70.90±134.98	0.19	0.94

## 117 ABSTRACT EN13: Dapagliflozin as Once‐Daily Therapy for Feline Diabetes Mellitus

### 117.1 **Viviani De Marco** ^1^, DVM, PhD; Archivaldo Reche Junior^2^; Gabrieli da Silva^3^; Denise Simões^3^; Gabriela Moreno^3^; Caroline Gonçalves^4^; Flavia Tavares^5^; Chen Gilor^6^

#### 117.1.1 ^1^Veterinary Endocrinologist, VetUnity Specialties, MV Minds; ^2^Vet Masters, FMVZ USP; ^3^FMVZ USP; ^4^VetUnity Specialties; ^5^E+Vet Endocrinology Veterinary; ^6^University of Florida, Gainesville, FL, USA

Bexagliflozin and velagliflozin, two sodium‐glucose co‐transporter‐2 inhibitors (SGLT2i), are effective in managing feline diabetes mellitus (FDM), but they are not available worldwide. The objective of this retrospective study was to evaluate the use of the SGLT2i dapagliflozin in FDM, either as monotherapy or in combination with insulin. Twenty‐seven diabetic cats (20 neutered males, 7 spayed females; mean age 11.9±3.5 years) were included. Twelve cats were newly diagnosed diabetics (ND) and 15 were insulin‐treated (IT) for 3 to 96 months (14 received glargine 100 U/mL, 1 received glargine 300 U/mL). Initial dapagliflozin doses were 10 mg q 24 h (*n* = 9), 5 mg q 24 h (*n* = 13), and 2.5 mg q 24 h (*n* = 5). Monitoring included q 12 h blood glucose (BG), continuous interstitial glucose (IG) measurements (FreeStyle Libre), and beta‐hydroxybutyrate (BHB) levels q 24 h during the first week, and then every 48 to 96 h. Normoglycemia was defined as BG or IG consistently below 180 mg/dL. Dapagliflozin doses were adjusted according to glycemic response. In cases of hypoglycemia (< 60 mg/dL), the dose was reduced or dosing frequency modified. After 30 days, 92.6% of cats achieved normoglycemia. In the IT group, insulin therapy was reduced (*n* = 4) or discontinued (*n* = 11) within 14 days. Dapagliflozin was discontinued in 9 cats: 6 due to remission (ND group), 1 due to severe hypoglycemia (IT group), and 2 due to euglycemic diabetic ketoacidosis (IT group). In the remaining 18 cats, dosing was individualized, ranging from 2.5 mg twice weekly to 5 mg q 24 h. Follow‐up ranged from 3 to 12 months. Clinical signs resolved completely in 25 cats (92.6%) and partially in 2 cats with comorbidities (hypersomatotropism and Cushing's syndrome). Dapagliflozin appears to be effective as monotherapy in feline diabetes and allowed insulin withdrawal in most cases.

## 118 ABSTRACT EN14: Serological Evidence of Pancreatic Inflammation in Dogs with Diabetes Mellitus

### 118.1 **Arielle Johnson‐Pitt** ^1^, BSc (Hons), BVetMed, MRCVS; Brian Catchpole^2^, BVetMed, MSc, PhD, FRCVS; Lucy Davison^3^, MA, VetMB, PhD, DSAM, DECVIM‐CA, MRCV

#### 118.1.1 ^1^PhD Student, Royal Veterinary College, London, UK; ^2^Professor, Companion Animal Immunology, Department of Pathobiology and Population Sciences, Royal Veterinary College, London, UK; ^3^Professor of Veterinary Clinical Genetics, Department of Clinical Science and Services, Royal Veterinary College, London, UK

**Background:** Canine diabetes mellitus (DM) is characterised by progressive beta‐cell loss and eventual insulin deficiency, but mechanisms involved remain uncertain. Pancreatic inflammation might be an under‐recognised contributor.

**Hypothesis/Objectives:** Evaluation of an acute pancreatic inflammatory marker (1,2‐o‐dilauryl‐rac‐glycero‐3‐glutaric‐acid‐(6’‐methylresorufin)‐ester (DGGR) lipase) in archived canine DM, sera will reveal evidence of pancreatic inflammation in some dogs.

**Animals:** Sera from 280 diabetic dogs, archived at −25°C (November 2009 to January 2019) with owner consent. Diabetic dogs were selected and grouped using hierarchical criteria related to potential pancreatitis risk: pancreatitis history (*n* = 29), diagnosed with DM < 7 days (*n* = 16), pure‐breed with known pancreatitis risk (*n* = 137), crossbreed with no reported history of pancreatitis (*n* = 98). Dogs with diagnosis of Cushing's syndrome or prednisolone usage were excluded.

**Methods:** Retrospective cohort study; DGGR lipase was measured by a colorimetric method (reference range 5.6–90 U/L).

**Results:** Elevated DGGR lipase was documented in 55 dogs (19.6%) (Figure 1). Although the highest proportion of positive cases was observed in the recently diagnosed group, there were no statistical differences among groups: *n* = 4/29 (13.8%) dogs with pancreatitis history, *n* = 6/16 (37.5%) DM < 7 days, *n* = 24/137 (17.5%) pancreatitis risk‐breed, and *n* = 21/98 (21.4%) crossbreed. There was no correlation between DGGR lipase and fructosamine or sample archive date.

**Conclusions and Clinical Importance:** Elevations in DGGR lipase can be detected in diabetic dogs with no history or known risk factors for pancreatitis. This might represent active causal or consequential pancreatic inflammation. Evaluation for pancreatic inflammatory markers should be considered in all canine DM patients, particularly around the time of diagnosis.

**Figure:**
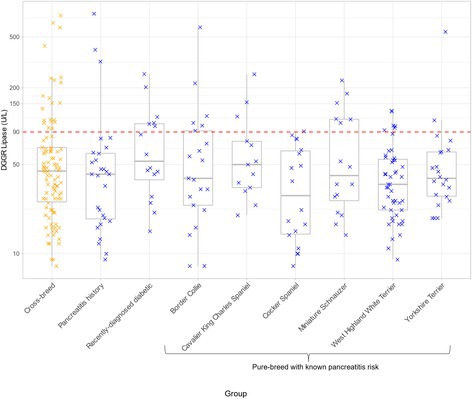
**Figure 1. Boxplot and scatter plat of DGGR lipase values measured in a cohort of diabetic dogs**. DGGR lipase measurements across selected diabetic dog groups: colour coded by potential pancreatitis risk (unlikely = orange, likely = blue).

## 119 ABSTRACT EN15: Comparison of Thyroid‐Stimulating Hormone Measurements Between Chemiluminescent Immunoassay and Bulk Acoustic Wave Assay

### 119.1 **Florence Leduc** ^1^, DACVIM (SAIM) Candidate; Petra Cerna^2^; Michael Lappin^3^

#### 119.1.1 ^1^SAIM Resident, Veterinary Teaching Hospital, Colorado State University, Fort Collins, CO, USA; ^2^Colorado State University, Fort Collins, CO, USA; ^3^Veterinary Teaching Hospital, Colorado State University, Fort Collins, CO, USA

**Background:** Thyroid‐stimulating hormone (TSH) is currently measured in cats in some laboratories using a chemiluminescent immunoassay (CLIA) optimized for canines (Immulite 2000; Siemens). An in‐clinic assay using acoustic wave technology (Truforma® TSH‐BAW; Zomedica) for use with feline sera is now available.

**Hypothesis/Objectives:** To evaluate the stability of TSH in serum following long‐term storage and to compare feline TSH concentrations determined by the CLIA and TSH‐BAW assays.

**Animals:** Stored (−80°C) sera from hyperthyroid cats (*n* = 13) before (week 0) and after (week 4) treatment with I‐131.

**Methods:** The 26 samples were stored for up to 24 months prior to this study. The week 0 samples had all been assayed by CLIA during the initial workup. Pre‐ and post‐storage CLIA results and CLIA and TSH‐BAW results before and after I‐131 treatment were compared

**Results:** Of the 7 samples initially positive by CLIA, 6 were positive after storage. One sample that was originally below the cutoff (< 0.03 ng/mL) was positive after storage (0.033 ng/mL). Of the 26 samples before and after I‐131, 8 were positive by TSH‐BAW and 7 were positive by CLIA; all but 1 positive were post‐treatment samples. The one discordant sample was post I‐131 and had values of < 0.030 ng/mL by CLIA and 0.028 ng/mL by TSH‐BAW.

**Conclusions and Clinical Importance:** Feline TSH appears to change minimally when stored frozen. In some situations, results of the TSH‐BAW may classify the status of a cat with thyroid illness more accurately than the CLIA.

## 120 ABSTRACT F01: Dual‐Center Retrospective Review of 204 Transfusions in Camelids

### 120.1 **Caitlyn Mullins** ^1^; Rachel Oman^2^; Jordan Gebhardt^3^; Emily Reppert^4^

#### 120.1.1 ^1^Resident, Large Animal Internal Medicine (Livestock Emphasis), Kansas State University; ^2^Associate Professor, Farm Animal Medicine and Surgery, Midwestern University; ^3^Assistant Professor, Diagnostic Medicine/Pathobiology, Kansas State University; ^4^Professor, Clinical Sciences, Kansas State University

**Background:** Transfusions in camelid species are performed without evidence‐based guidelines for both prevention and treatment of disease. Safe and recommended practices for administering blood products with minimal risk of reactions are needed.

**Hypothesis/Objectives:** To describe the indications for, methods of, and outcomes associated with transfusions in camelids. To identify risk factors for transfusion reactions and non‐survival; to evaluate the change in blood proteins with plasma transfusions.

**Animals:** Two hundred and four transfusions performed in 169 camelids.

**Methods:** A dual‐center retrospective review of medical records from 2010 to 2024. Signalment, indication for transfusion, pre‐ and post‐transfusion bloodwork values, administration of pre‐medication, presence of transfusion reactions, and survival to discharge were reviewed. Data were analyzed with descriptive statistics, a generalized linear model, and linear regression.

**Results:** Presence of transfusion reactions resulted in a lower probability of survival to discharge (*P* = 0.048). Animals less than 1 day of age had a greater probability of survival compared to patients > 1 year of age (*P* = 0.046). There was no statistically significant difference in probability of a transfusion reaction with respect to administration of pre‐medication, rate of transfusion administration, or previous transfusions (*P* > 0.10). Administration of plasma resulted in a statistically significant increase in total protein, albumin, and globulin levels (*P* ≤ 0.003).

**Conclusions and Clinical Importance:** The use of pre‐medications for the sole purpose of preventing or limiting transfusion reactions is likely not justified. Having received prior transfusions does not significantly affect the future risk of a transfusion reaction.

## 121 ABSTRACT F02: Pharmacokinetics of Lidocaine and Its Metabolites Following Constant Rate Infusion in Healthy Goats

### 121.1 **Florence Dupuis‐Dowd** ^1^; Munashe Chigerwe^2^; Heather Knych^2^

#### 121.1.1 ^1^Large Animal Internal Medicine Resident, University of California, Davis; ^2^University of California, Davis

**Background:** Continuous rate infusion (CRI) of lidocaine is commonly used as an analgesic in hospitalized goats, but no pharmacologic data is available in this species. The dosage used, often extrapolated from equine studies, might lead to suboptimal dosage and pain control in goats.

**Objectives:** To determine the plasma concentrations achieved during a three‐day administration of a CRI of lidocaine at 0.05 mg/kg/min in healthy goats.

**Animals:** Eight healthy adult does from a teaching herd.

**Methods:** Nine goats were enrolled for this clinical trial and were administered a CRI of lidocaine, intravenously, at 0.05 mg/kg/min for three consecutive days. Samples were collected before lidocaine administration, 0.25, 0.5, 1, 3, 6, 12, 24, 36, 48, and 72 h following the beginning of the CRI, and 0.25, 0.5, 1, 3, 6, and 12 h after discontinuation. Liquid chromatography tandem mass spectrometry was performed to determine serum concentration of lidocaine and its metabolites (OH‐lidocaine, glycinexylidide, and monoethylglycinexylidide). Pharmacokinetic analysis was performed using non‐compartment approach.

**Results:** Transient self‐limiting collapse was observed in three goats. Serum lidocaine concentrations reached a steady state 6 h after starting the CRI. Mean maximal serum lidocaine concentration was 1425.2 ng/mL (range 527.2–2930.8). Clearance was 4.2 L/h/kg, with a terminal half‐life of 1.4 h. Only OH‐lidocaine was correlated to serum lidocaine concentration (*P* = 0.01).

**Conclusions and Clinical Importance:** Lidocaine metabolism is highly variable among healthy individuals, with some goats possibly requiring higher doses. Lidocaine as a CRI should not be administered to goat undiluted due to increased likelihood of adverse event.

## 122 ABSTRACT F03: Impact of Nebulizer Device on Aerosol Drug Delivery in Ruminants

### 122.1 **Elizabeth Rumfola** ^1^; Dusty Nagy^2^, DVM, PhD, DACVIM (LAIM); Rebecca Legere^2^, DVM, PhD, DACVIM (LAIM); Rebecca Legere^2^, DVM, PhD, DACVIM (LAIM)

#### 122.1.1 ^1^Veterinary Resident, Texas A&M University; ^2^Texas A&M University

**Background:** Treating severe respiratory disease in ruminants is often challenging, especially for judicious antimicrobial stewardship and appropriate treatment duration. Nebulized therapy in ruminants delivers intrapulmonary aerosolized drugs anecdotally with reported success, but safety and efficacy of commonly used therapeutic medications with commercially available veterinary nebulizers is unexplored.

**Hypothesis/Objectives:** To evaluate effects of the type of nebulization device and different drug formulations on drug flow rate and clinically relevant particle size distribution parameters correlated with extent of intrapulmonary drug delivery, including mass mean aerodynamic diameter (MMAD), fine particle fraction (FPF), and geometric standard deviation (GSD).

**Animals:** No animals used.

**Methods:** Ampicillin, tulathromycin, dexamethasone, and albuterol sulfate solutions were prepared, viscosity measured, and then tested with 4 nebulizers through an inhalation cell with in‐line particle size analysis via laser diffraction analysis. Flow rate was measured by changes in drug volume in reservoir cup over time of nebulization. Each drug × device pairing was tested in triplicate; MMAD, FPF, and GSD values calculated from serial 30‐second measurement windows in triplicate within each data capture. Data were tested for normality using Shapiro‐Wilks test and compared with ANOVA or Kruskal‐Wallis test.

**Results:** Initial analysis demonstrates significant variation in particle size distribution factors among drugs when using the same nebulization device and among devices using the same drug formulation.

**Conclusions and Clinical Importance:** Considerable and clinically relevant differences between nebulization devices and drugs used for treating respiratory disease in ruminants warrant further investigation to optimize treatment efficacy while enhancing patient safety and clinical outcomes.

## 123 ABSTRACT F04: Pharmacokinetics of Famotidine in Goats: Subcutaneous and Intravenous Administration

### 123.1 **Olivia Escher‐Price** ^1^; Joe Smith^2^, DVM, MPS, PhD, DACVIM, DACVCP; Kamryn Christopher^3^, BS; Bryan Hogan^3^, BS; Lainey Harvill^3^, BS; Makenna Hopson^3^, BS; Madeline Duncan^4^; Kya Logan^5^; Hannah Luu^3^, BS; Keren Hernandez^3^, BS; Jessica Gebert^6^, DVM; Srimona Bandyopadhyay^7^, DVM; Sherry Cox^8^, MS, PhD

#### 123.1.1 ^1^DVM‐PhD Student, UTKCVM; ^2^Assistant Professor, LACS, UTKCVM; ^3^Student Assistant, LACS, UTKCVM; ^4^BDS, UTKCVM; ^5^Student, LACS, UTKCVM; ^6^DVM Student, LMU; ^7^Intern, LACS, UTKCVM; ^8^Clinical Professor, BDS, UTKCVM

**Background:** Goats can develop abomasal ulceration due to inappetence, adverse reactions to medications, and stress. There is currently no FDA approved drug for prevention or treatment of abomasal ulceration in goats. Famotidine, a histamine type‐2 (H‐2) receptor antagonist is often used in veterinary medicine to treat gastric ulceration in dogs and in human medicine to treat duodenal ulceration.

**Objectives:** The objectives of these studies were to determine the pharmacokinetics of a single dose of famotidine in goats via subcutaneous (SC) (1.2 mg/kg) and intravenous (IV) (0.6 mg/kg) administration.

**Animals:** Six healthy goats were used in each study.

**Methods:** Plasma samples were collected and analyzed using reverse phase high performance liquid chromatography (HPLC) for famotidine concentration.

**Results:** Elimination half‐life for IV was 0.31 h while SC was 1.31 h. Mean residence time for IV was estimated at 0.33 h and for SC was estimated at 1.62 h. Maximum plasma concentration for IV administration was 5476 ng/mL and for SC administration was 735 ng/mL. SC Bioavailability was calculated at 63%.

**Conclusions and Clinical Importance:** Compared to other studies in horses and cattle, famotidine appears to be more rapidly eliminated in goats. However, comparing the two routes of administration, SC administration of famotidine may be a useful way of maintaining plasma concentration over time for goats. Future studies investigating pharmacodynamics, withdrawal period, and microbiome effects would be beneficial.

## 124 ABSTRACT F05: The Effect of Antigen Characteristics on Camelid Immunoglobulin Isotype Responses

### 124.1 **Fionna Lehmann** ^1^; Daniela Bedenice^2^, DVM, DACVIM (LA), DACVECC (Eq); Jacque Tremblay^3^; Melissa Mazan^4^, DVM, DACVIM (LA); Charles Shoemaker^5^, PhD

#### 124.1.1 ^1^Resident in Large Animal Internal Medicine, Cummings School of Veterinary Medicine, Tufts University; ^2^Professor, Clinical Sciences, Cummings School of Veterinary Medicine, Tufts University; ^3^Senior Research Technician, Department of Infectious Disease and Global Health, Tufts University; ^4^Professor; Associate Dean of Professional Education, Clinical Sciences, Cummings School of Veterinary Medicine, Tufts University; ^5^Professor, Department of Infectious Disease and Global Health, Tufts University

**Background:** Camelids are the only mammalian family to produce both conventional antibodies, with a paired heavy and light chain (IgG1), and two heavy‐chain only antibody (HcAb) types (IgG2 and IgG3). The binding domains of these antibodies (called VHHs or nanobodies) have significant potential as therapeutic agents due to their small size and durability. However, the factors that drive conventional versus HcAb response in camelids are currently unknown.

**Hypothesis:** Relative serum concentration of HcAbs versus conventional antibodies is influenced by antigen size and type, as well as individual immune response.

**Animals:** Banked sera from 8 research alpacas, immunized with 24 antigens.

**Methods:** IgG production was quantified in sera using dilution ELISA. Limit titers were compared between groups by univariate analysis, based on data normality.

**Results:** HcAbs accounted for only ~5% of total IgG (range 0.03%–24%), with small antigens (< 50 kDa) inducing a lower overall IgG response than medium (50–100 kDa; *p* = 0.027) and large (> 100 kDa; *p* = 0.004) antigens. More specifically, significantly less IgG2 and IgG3 were produced in response to small versus medium (*p* = 0.047) and small compared to large antigens (*p* = 0.039), respectively. Descriptive assessment suggested that carbohydrate and glycolipid antigens induced a lower antibody response than protein‐based antigens, but statistical analysis was limited by sample size. Additionally, inter‐individual variation in immune response was observed between animals.

**Conclusions and Clinical Importance:** Antigen size affected relative HcAb and conventional antibody responses in alpacas. Although further assessment is necessary to characterize the immune response to non‐protein antigens, these data may direct epitope selection for novel immunotherapeutic development.

## 125 ABSTRACT F06: Bacteremia Is Uncommon in Systemically Ill Goats Presented to a Tertiary Referral Hospital

### 125.1 **Flavie Payette** ^1^; Clarisa Romero Hernandez^2^, MVZ, DACVIM (LA); Marie‐Eve Fecteau^3^, DVM, DACVIM (LA); Daniela Luethy^4^, DVM, MPH, DACVIM (LAIM)

#### 125.1.1 ^1^LAIM Resident, University of Pennsylvania; ^2^Large Animal Internist, Montana Equine; ^3^Professor of Farm Animal Medicine and Surgery, University of Pennsylvania; ^4^Assistant Professor of Large Animal Medicine, University of Pennsylvania

**Background:** Sepsis in goats is not well described. A better understanding of which diseases and factors are associated with bacteremia and sepsis in systemically ill goats could help improve outcome and guide antimicrobial stewardship.

**Hypothesis/Objectives: (**1) Evaluate frequency of bacteremia in healthy and systemically ill goats; (2) Determine clinical parameters associated with a positive blood culture.

**Animals:** 84 systemically ill and 19 healthy adult goats.

**Methods:** Prospective clinical study. Blood cultures were collected aseptically and clinicopathological parameters, diagnosis and outcome were recorded. Groups of goats were compared based on SIRS criteria extrapolated from other species, blood culture results, outcome, and systems affected, using Student's T test or Fisher's exact test.

**Results:** Ten goats (11.9%; 9 sick, 1 healthy) had positive blood cultures, yielding 11 different isolates (8 Gram‐positive and 3 Gram‐negative). When analyzed by disease category, 33.3% (2/6) of hepatic, 28.5% (2/7) of respiratory, 18% (2/11) of neurologic, 9% (2/21) of gastrointestinal, and 3% (1/29) of urinary cases had positive blood cultures. 47.6% (40/84) of sick goats fit SIRS criteria. Half of the goats with positive blood cultures met SIRS criteria, but SIRS was not associated with a positive blood culture (*p* = 0.7). Goats with a positive blood culture had higher rectal temperatures than those that did not have positive blood cultures (102.9±0.3F vs 101.6±0.2F; *p* = 0.01).

**Conclusion and Clinical Importance:** In this cohort of adult goats, bacteremia was documented infrequently. Blood cultures might be a useful adjunctive diagnostic in goats with hepatic or pulmonary disease.

## 126 ABSTRACT F07: Cerebrospinal Nematodiasis in 8 Goats (2011–2024)

### 126.1 **Jessica Garcia** ^1^; Joe Smith^2^, DVM, MPS, PhD, DACVIM, DACVCP; Deanna Schaefer^3^, DVM, MS, MLS (ASCP), DACVP; Pierre‐Yves Mulon^4^, DVM, DES, DACVS; Pierre‐Yves Mulon^4^, DVM, DES, DACVS

#### 126.1.1 ^1^LAIM Resident, UTKCVM; ^2^Assistant Professor, LACS, UTKCVM; ^3^Associate Professor, BDS, UTKCVM; ^4^Associate Professor, LACS, UTKCVM

**Background:** Cerebrospinal nematodiasis due to *Parelaphostrongylus tenuis* is described in South American Camelids in North America, but information regarding the condition in goats is limited.

**Hypothesis/Objectives:** This case series describes clinical presentation, diagnostics, treatment, and outcome for goats with presumptive cerebrospinal nematodiasis.

**Animals:** Medical records for goats presented to the University of Tennessee from 2011–2024. A presumptive diagnosis was made based on the presence of an eosinophilic pleocytosis.

**Methods:** Retrospective case series.

**Results:** Eight goats were identified that met inclusion criteria. Cerebrospinal fluid analysis showed: total nucleated cell count: 140±157/μL (range: 12–430/μL); % eosinophils: 53.4±19% (range: 33%–89%); and total protein: 88±63 mg/dL. All 8 goats were treated with fenbendazole and anti‐inflammatory drugs (NSAIDs ± corticosteroids), in addition to other therapies and 4 received physical rehabilitation therapy. At discharge or follow‐up, 7 goats were ambulatory and had minimal neurologic deficits. One goat was euthanized due to lack of response to treatment for concurrent anemia.

**Conclusions and Clinical Importance:** In this population, goats with presumptive cerebrospinal nematodiasis due to *P. tenuis* seem to respond well to therapies described for South American Camelids, with a higher percentage surviving to discharge than reported for llamas and alpacas. Clinicians and producers should be aware of the potential for a decreased prognosis with concurrent disease.

## 127 ABSTRACT F08: A Retrospective Look at the Efficacy of Proctoclysis in Hospitalized Swine

### 127.1 **Jessica Lynch** ^1^; Meggan Graves^2^, DVM; Joe Smith^3^, DVM, MPS, PhD, DACVIM, DACVCP

#### 127.1.1 ^1^Veterinary Student, University of Tennessee; ^2^Associate Clinical Professor, LACS, University of Tennessee; ^3^Assistant Professor, LACS, UTKCVM

**Background:** Intravenous (IV) fluid administration can be challenging in swine. Rectal fluid administration (proctoclysis) has been used successfully in other species, and is commonly utilized in hospitalized pigs with limited IV access. Recently, a 2022 study in healthy swine reported that proctoclysis did not significantly affect serum biochemical analytes compared to IV fluids, casting doubt to the efficacy of proctoclysis. However, this has not been the clinical impression at the University of Tennessee College of Veterinary Medicine (UTCVM).

**Hypothesis/Objectives:** The objective of this study was to evaluate the efficacy of proctoclysis in an unhealthy swine population. We hypothesized there would be no changes before and after proctoclysis.

**Animals:** We retrospectively evaluated pigs receiving proctoclysis at UTCVM from 2016 to 2024. Twenty‐five cases fit the admission criteria.

**Methods:** Patient demographics and signalment were identified, and medical records were scoured for electrolyte (Na, K, and Cl), hydration (HCT, TS, and Lactate) and renal (BUN and Creatinine) parameters recorded before and after proctoclysis in hospitalized pigs. Data was statistically compared for differences (*p* < 0.05).

**Results:** Statistically significant changes were identified after proctoclysis for hematocrit (*p* = 0.0009); and creatinine (*p* = 0.0002). Mean reductions and p values were NA (1.39 mmol/L; *p* = 0.4262), K (0.33 mmol/L; *p* = 0.4464), Cl (2.26 mmol/L; *p* = 0.0680), HCT (8.79%; 0.0009), TS (0.415 g/dL; 0.0576), Lactate (1.77 mmol/L, *p* = 0.4609), Creatinine (1.19 mg/dL; *p* = 0.0002), and BUN (6.46 mg/dL; *p* = 0.1297).

**Conclusions and Clinical Importance:** Proctoclysis appears to be beneficial in unhealthy swine patients, but additional research utilizing uniform treatment strategies is indicated to further evaluate its efficacy.

## 128 ABSTRACT F09: Fecal Microbiota Dynamics in Calves During the First 24 Days in a Veal Unit

### 128.1 **Marcio Costa** ^1^, DVM, DVSc, PhD; Sarah Crespo^2^; Sebastien Buckzinski^2^; Diego Gomez^3^; Gilles Fecteau^2^

#### 128.1.1 ^1^Associate Professor, University of Montreal; ^2^Faculty of Veterinary Medicine, University of Montreal; ^3^Ontario Veterinary College, University of Guelph

**Background:** The intestinal microbiota plays a crucial role in health, with early‐life disruptions potentially affecting long‐term outcomes. Various factors influence its composition, particularly during critical periods such as veal calf rearing.

**Objectives:** To evaluate fecal microbiota changes in calves during the first 24 days after entering a veal rearing unit.

**Animals:** Fifty male calves (8–14 days old) purchased from a single auction market but originating from different farms.

**Methods:** Fecal samples were collected at arrival (D0) and on days 4, 10, and 24. Fecal scores and antibiotic use were recorded. Microbiota composition was analyzed via 16S rRNA sequencing (V4 region). ANOVA and PERMANOVA tests (*p* < 0.05) determined statistical significance.

**Results:** Richness and diversity were lowest at arrival and changed significantly over time (*p* < 0.001). However, microbiota composition was not associated with diarrhea incidence or antibiotic treatment. Specific bacterial taxa were linked to each time point (e.g., *Bifidobacterium* and *Veillonella* at D0, *Lactobacillus* and *Bacteroides* at D10). Diarrhea occurrence remained stable over time (*p* = 0.0037).

**Conclusions and Clinical Importance:** Significant microbiota shifts occurred over time, independent of diarrhea or antibiotic use, suggesting a strong environmental influence. Stress, transportation, dietary transitions, and housing conditions likely contributed to these changes. Further research is needed to determine the long‐term impact on calf health and performance and to identify microbiota modulation strategies during early rearing.

## 129 ABSTRACT F10: Insights from Hong Kong's First Ruminant Ambulatory Service

### 129.1 Eloi Guarnieri, DVM, MSc, DACVIM (LAIM)

#### 129.1.1 Clinical Assistant Professor, City University of Hong Kong

**Background:** Veterinary care for domestic ruminants in Hong Kong has historically been limited. In August 2023, the first non‐governmental ambulatory veterinary service for domestic ruminants was established, offering free services.

**Objectives:** To describe the farms visited and the services requested from the Ruminant Ambulatory Service (RAS).

**Animals and Methods:** This retrospective observational study reviewed reports of veterinary visits to local farms from August 1, 2023, to December 31, 2024. The description and number of farms, animals, and consultations were recorded.

**Results:** The RAS had 32 clients (goats, *n* = 30; sheep, *n* = 3; cattle, *n* = 3; alpacas, *n* = 2), including 93.8% (30/32) of leisure or educational farms. The median goat herd size was 8.5 (range: 1–250). A total of 439 consultations were conducted (herd health visits, *n* = 108; individual animal visits, *n* = 331). Goat herds accounted for 90.7% (98/108) of herd health visits, which included vaccinations, deworming, and pregnancy diagnoses. The most common reasons for individual animal visits were skin conditions (excluding foot conditions; 20.8%, 69/331) and animal health certification (16.6%, 55/331). In goats, the most frequent individual dermatological diagnoses were wounds (*n* = 16), pediculosis (*n* = 11), and myiasis (*n* = 7).

**Conclusions and Clinical Importance:** Local farmers have preferentially shown an interest in preventive medicine and care of animals showing obvious clinical signs. Future studies will involve questionnaires to investigate the cultural motivations and expectations of Hong Kong ruminant farmers regarding veterinary care.

## 130 ABSTRACT F11: Influence of Sample Collection Site on Blood Glucose Measurements in Goats

### 130.1 **Jennifer Halleran** ^1^, PhD, DACVIM (LAIM); Sara Fitzgerald^2^; Camryn Kline^2^; Danielle Mzyk^3^, DVM, PhD; Madelyn Schwartz^2^, MS; Dileydis Soto Montes^2^

#### 130.1.1 ^1^Assistant Professor, Ruminant Medicine, North Carolina State University; ^2^North Carolina State University; ^3^Clinical Veterinarian, North Carolina State University

**Background:** Portable blood glucose (BG) monitors are used for stall side assessment of BG. In small ruminants, serially measuring BG is necessary for certain disease processes. Pending the size, behavior and available help, measuring the BG from the jugular vein may be challenging.

**Hypothesis/Objective:** The overall object was to compare BG measurements in the jugular vein and ear vein in three different metabolic states. We hypothesize that the ear vein would not be a suitable alternative collection site to assess BG measurements.

**Animals:** Five intact male Boer Goats (8–9 months old) were used for this study. Prior to the study, they were deemed healthy by physical examination.

**Methods:** Blood glucose measurements were assessed in three induced metabolic states: healthy, hyperglycemia (0.2 mL/kg of 50% dextrose intravenously) and hypoglycemia (0.1 units/kg of short acting insulin intramuscularly). Blood glucose samples were collected from the jugular vein and ear, and were measured using the AlphaTrak3 glucose monitor. All goats were enrolled concurrently and had a 2 day wash out period in between treatments.

**Results:** All of the goats handled each of the treatments well. A liner mixed model was used. Model assumptions were satisfied. It was found that the sample site did not have an effect on the BG, whereas the treatment did.

**Conclusions and Clinical Importance:** This trial indicates that the ear vein can serve as a suitable alternative sample collection site to assess BG measurements. This will allow for ease of measurement for clinical patients.

## 131 ABSTRACT F12: The Pharmacokinetics and Pilot Pharmacodynamics of Subcutaneous Levamisole Through a Combination Product in Domestic Goats

### 131.1 **Grace Malla** ^1^, BS; Joe Smith^2^, DVM, MPS, PhD, DACVIM, DACVCP; Meggan Graves^3^, DVM; Lisa Ebner^4^, DVM, MS, DACVAA; Ryan Branham^5^; Jessica Lynch^6^, BS; Laura Gilliard^7^; Julia Cutchin^8^; Madeline Duncan^8^; Rebecca Rahn^9^, BS; Michelle Buckley^10^, DVM, PhD; Andrew Ursini^11^, BS; Cassandra Klostermann^12^, DVM, MPH, DES; Jessica Shanks^13^, MS; Sherry Cox^14^, MS, PhS

#### 131.1.1 ^1^DVM‐MS Student, UTKCVM; ^2^Assistant Professor, LACS, UTKCVM; ^3^Associate Clinical Professor, LACS, UTKCVM; ^4^Clinical Professor, Texas Tech University; ^5^Student Assistant, LACS, UTCVM; ^6^DVM Student, UTKCVM; ^7^Student Assistant, LACS, UTKCVM; ^8^Student Assistant, BDS, UTKCVM; ^9^Veterinary Student, LACS, UTKCVM; ^10^Assistant Professor, Texas Tech University; ^11^Laboratory Technologist, BDS, UTKCVM; ^12^Clinical Assistant Professor, LACS, UTKCVM; ^13^Extension Specialist, UTK; ^14^Clinical Professor, BDS, UTKCVM

**Background:** Anthelmintic resistance is a worldwide problem, and few products are FDA‐approved for use in goats. Pharmacokinetic data is sparse for levamisole in goats despite it being an efficacious anthelmintic.

**Hypothesis/Objectives:** The purpose of this study was to generate the levamisole pharmacokinetic parameters from a levamisole‐doramectin combination when given subcutaneously to goats. A secondary objective was to gather pilot pharmacodynamic data for this product.

**Animals:** Six healthy goats from a university teaching herd were used.

**Methods:** An injectable levamisole formulation was administered subcutaneously to six goats. Blood samples were drawn at 14 time points. This process was repeated using a commercially available oral drench after a 14‐day washout period. The drug present in the samples was quantified using high performance liquid chromatography. Standard pharmacokinetic parameters were generated. Fecal egg count reduction testing was performed pre‐ and post‐treatment with the injectable product.

**Results:** After one subcutaneous injection, the average maximum plasma concentration (Cmax) was 468.57 ng/mL, time to Cmax (Tmax) was 2.24 h, area under the curve (AUC) was 3206.42 h*ng/mL, and elimination half‐life (T1/2) was 2.36 h. The relative bioavailability of the subcutaneous formulation as compared to the oral formulation was 185%. No adverse effects were observed. Reduction in fecal egg count was observed.

**Conclusions and Clinical Importance:** The tested levamisole product represents a possible choice as an anthelmintic in goats. Further field trials are needed, but the preliminary data outlined in this study is suggestive of the product's efficacy and safety.

## 132 ABSTRACT F13: Impact of Storage Duration on Erythrocyte Health and Biochemical Analytes in Caprine Blood Products

### 132.1 **Lanie Phillips** ^1^; Erika Gruber^2^; Hannah Dion^2^; Anna Flynn^3^; Hakeem Jenkins^2^; Lisa Gamsjäger^2^

#### 132.1.1 ^1^DVM Student, North Carolina State University; ^2^North Carolina State University; ^3^Department of Agriculture

**Background:** The effect of storage on hematologic and biochemical properties of caprine blood products is not well‐understood.

**Objectives:** Assess the impact of storage duration on erythrocyte fragility, packed cell volume (PCV), hemolysis and biochemical composition in caprine whole blood (WB) and packed red blood cells (pRBCs).

**Animals:** 6 healthy Boer × Kiko goats.

**Methods:** Repeated measure cohort study. The WB and pRBC were stored in CDPA‐1 (citrate, dextrose, phosphate, adenine) or CPD (citrate, phosphate, and dextrose) with Optisol, respectively, at 4°C–7°C for seven weeks. The PCV, osmotic fragility, glucose, and lactate concentrations were measured weekly, and hemolysis and potassium concentrations were measured bi‐weekly. Changes from baseline were assessed with ANOVA or Friedman's test and Dunnett's or Dunne's correction for multiple comparisons. *p* values <0.05 were considered significant.

**Results:** Median PCV decreased from 31% to 21.5% after two weeks in WB (*P* = 0.002), with no change in pRBC. Significant hemolysis was observed after two weeks in WB (*P* = 0.04) and pRBC (*P* = 0.02), while osmotic fragility did not increase in either product. Baseline glucose (~500 mg/ml) decreased by two and three weeks in pRBC (*P* = 0.002) and WB (*P* = 0.0005), respectively, but remained substantially higher (> 300 mg/dL) than in normal blood. Lactate concentrations increased after one and three weeks in WB (*P* = 0.045) and pRBC (*P* = 0.03), respectively. Potassium concentrations increased substantially after two weeks (*P* = 0.001) in both products.

**Conclusions and Clinical Importance:** Hemolysis and biochemical changes in caprine blood products develop as early as one week. The use of fresh blood may be preferable for critically‐ill patients.

## 133 ABSTRACT F14: The Pharmacodynamics and Pharmacokinetics of Acepromazine in Domestic Goats

### 133.1 **Joe Smith** ^1^, DVM, MPS, PhD, DACVIM, DACVCP; Laura Gilliard^2^; Matthew Aviles^3^, DVM; Julianne Nussbaum^3^, DVM; Marc Harper^3^, DVM, MS; Ryan Branham^2^; Aidan Smith^2^; Cassandra Klostermann^4^, DVM, MPH, DES; Jessica Lynch^5^, BS; Heather Knych^6^, DVM, PhD, DACVCP; Chiara Hampton^2^, DVM, MS, DACVAA

#### 133.1.1 ^1^Assistant Professor, UTKCVM; ^2^Student, LACS, UTKCVM; ^3^Intern, LACS, UTKCVM; ^4^Clinical Assistant Professor, LACS, UTKCVM; ^5^DVM Student, LACS, UTKCVM; ^6^Professor, UC Davis; ^7^Assistant Professor, LACS, UTKCVM

**Background:** Acepromazine is used as a tranquilizer in goats. However, there is pauce information available on its pharmacodynamic effects (PDE) and pharmacokinetic profile (PK) in this species.

**Objectives:** The primary objective of this study was to investigate the PDE of intravenous (IV) acepromazine in goats. A secondary objective was to describe its PK.

**Animals:** Six healthy adult goats (1 castrated male and 5 females) of various breeds with weight 64.43 kgs.

**Methods:** Blood samples were collected at 0, 2, 5, 10, 15, 30 min and at 1, 2, 4, 8, 12, 24 h after administration of 0.1 mg/kg of IV acepromazine. Packed cell volume (PCV), total solids (TS), and onset and time spent in recumbency were the PDE measured variables of interest. Scores from a standardized tranquilization scale were assigned pre‐administration and subsequently, at every collection timepoint. PCV, TS and tranquilization were tested via RM‐ANOVA. Significance was set at a *p* < 0.05. PK parameters were determined via a compartmental analysis after plasma acepromazine concentration determination by liquid chromatography‐tandem mass spectrometry.

**Results:** No adverse effects were observed. PCV decreased significantly at the 1 (*p* = 0.0481) and 2‐h (*p* = 0.0088) timepoints, with no changes in TS. Tranquilization was greater at 0.25 (*p* = 0.0026), 0.5 (*p* = 0.0101) and 1 (*p* = 0.0101) hour after administration. Recumbency was observed in 5/6 goats (duration range: 8–237 min). Pharmacokinetic parameters were modelled.

**Conclusions and Clinical Importance:** Clinicians should be aware of changes to PCV and the potential for variable recumbency in goats administered intravenous acepromazine.

## 134 ABSTRACT F15: The Effects of Polyclonal IgY Supplementation on the Health of Pre‐Weaned Dairy Calves

### 134.1 **Luisa De La Cuadra Rojas** ^1^, DVM; Catie Cramer^2^, MS, PhD; Sarah Raabis^3^, DVM, PhD, DACVIM

#### 134.1.1 ^1^Veterinarian/Master Student and Livestock Intern, Colorado State University; ^2^Associate Professor, Animal Sciences, Colorado State University; ^3^Associate Professor, Clinical Science, Colorado State University

**Background:** Diarrhea (DIA) is prevalent in preweaned dairy calves. Non‐antimicrobial treatments, such as immunoglobulin Y (IgY), may reduce pathogen colonization and reduce the risk of antimicrobial resistance on farms.

**Hypothesis/Objectives:** Polyclonal IgY oral supplementation is associated with reduced duration of DIA, pathogen shedding and improved average daily gain (ADG) in dairy calves.

**Animals:** 187 newborn dairy calves.

**Methods:** Sample size (*n* = 80) was calculated to detect a 20% difference in days with diarrhea between groups (1−*β*=0.8; *α*=0.05). A randomized controlled clinical trial allocated calves to treatment (TX; received a single dose of IgY at birth; *n* = 91) or control (CON; no IgY administered; *n* = 96) groups over 4 months from 12/2023. Fecal scores (FS) and body weights were collected, and fecal pathogen shedding (*Salmonella* culture, and BCoV, BRV, *C. parvum* qPCR). Multivariable models assessed associations between treatment group, median (Q1, Q3) days with DIA (FS ≥ 2) during days 1–14, pathogen shedding, and ADG.

**Results:** The median number of days with DIA was 2 (1, 3) for CON and 1 (1,3) for TX calves (*p* = 0.68). No significant differences were detected in the odds of pathogen shedding (*P* > 0.2): BCoV (OR: 2.43, CI: 0.7–8.88), BRV (OR: 1.08, CI: 0.30–3.85). TX was not associated with a difference in ADG (*p* = 0.69; 0.81 kg ± 0.05; 88).

**Conclusion and Clinical Relevance:** Polyclonal IgY supplementation at birth was not associated with improved clinical health, reduced pathogen shedding or increased ADG. Further studies investigating multi‐day polyclonal IgY supplementation is indicated.

## 135 ABSTRACT F16: The Impact of Stress on Bovine Pulmonary Mucosal Immunity

### 135.1 **Grace Jakes** ^1^, BS; Dylan Ammons^2^, PhD, Veterinary Student; Randy Hunter^3^, DVM; Ediane Silva^4^; Steven Dow^5^, DVM, PhD, DACVIM (SAIM); Sarah Raabis^1^, DVM, PhD, DACVIM (LAIM)

#### 135.1.1 ^1^Graduate Student, Colorado State University, Fort Collins, CO, USA; ^2^Microbiology, Immunology, Pathology, Colorado State University, Fort Collins, CO, USA; ^3^Hunter Cattle Company, Brooklet, GA, USA; ^4^United States Department of Agriculture, Agriculture Research Service, Washington, DC, USA; ^5^Clinical Sciences, Colorado State University, Fort Collins, CO, USA

**Background:** Bovine respiratory disease (BRD) is a leading cause of morbidity and mortality in cattle. Stress due to transport and weaning increases BRD risk, yet mechanisms of immune susceptibility following stress are less clear. Here we evaluated host‐microbe interactions from bronchoalveolar lavage fluid (BALF) to understand immune responses to stress.

**Hypothesis/Objectives:** We hypothesized that stress would result in pulmonary immunomodulation, and that microbial populations would shift between stress and acclimation.

**Animals:** Stocker calves were purchased at auction and transported to a commercial backgrounder. A subset of healthy calves (rectal temperature <103.5°F, no lung consolidation on lung ultrasound) were sampled for BALF within 24 h (**Stressed**; *n* = 12) or acclimated to the backgrounding facility for 2–3 months before sampling (**Acclimated**; *n* = 7).

**Methods:** In this case‐control study, BALF sampling was performed at the backgrounding facility, and BALF samples were processed for bulk transcriptomic (RNA‐seq, STAR alignment pipeline) and 16S rRNA sequencing (Qiime2 analysis pipeline) separately. Gene expression data was analyzed in Cibersortx to predict abundance of major cell populations.

**Results:** RNA‐seq profiles of stressed calves demonstrated over 350 differentially expressed genes. Key pathways upregulated included neutrophil migration, with an increase in neutrophil numbers predicted in **stressed** calves (2.5‐fold increase, *p* < 0.01). Microbiome analysis demonstrated no difference in diversity metrics (*p* > 0.05) despite the apparent immune activation.

**Conclusions and Clinical Importance:** In contrast to our hypothesis, these data demonstrate that healthy stocker calves at arrival have an increase in neutrophil‐associated gene expression relative to acclimated calves, even as microbial diversity metrics demonstrate no differences.

## 136 ABSTRACT GI01: Analytical Validation of a Point‐of‐Care Assay for Pancreatic Lipase in Dogs and Cats

### 136.1 **Joerg Steiner** ^1^; Yu‐An Wu^2^, DVM, MS, GStat; Phillip Guadiano^2^; Kathleen Aicher^2^; Jonathan Lidbury^2^; Sue Yee Lim^2^

#### 136.1.1 ^1^University Distinguished Professor of Small Animal Internal Medicine | Director of the Gastrointestinal Laboratory, Texas A&M University; ^2^Gastrointestinal Laboratory, Texas A&M University

**Background:** The diagnosis of pancreatitis in dogs and cats can be challenging. Traditional serum lipase activity assays lack specificity for the measurement of pancreatic lipase, limiting their clinical utility. Measurement of serum pancreatic lipase immunoreactivity (PLI) has been analytically and clinically validated for the diagnosis of pancreatitis in dogs and cats. A new point‐of‐care lipase activity assay that aligns with Spec PL® measurements and specifically measures pancreatic lipase would further facilitate the diagnosis of pancreatitis.

**Objective:** To analytically validate a point‐of‐care assay (Catalyst® PL, IDEXX Laboratories, Westbrook, Maine), for the measurement of pancreatic lipase in serum from dogs and cats.

**Samples:** Surplus serum samples from dogs and cats.

**Methods:** Dilutional parallelism, spiking recovery, intra‐ and inter‐assay variabilities, effect of interfering substances, and sensitivity to storage at various temperatures were assessed. Specificity was evaluated by measuring serum samples from dogs with suspected pancreatic acinar atrophy (PAA).

**Results:** Analytical validation showed good linearity, accuracy, precision (mean [max.] intra‐assay variability: dog: 5.2% [8.1%], cat: 5.2% [8.6%]), and repeatability (mean [max.] inter‐assay variability: dog: 6.6% [8.1%], cat: 6.1% [10.4%]). Results of the Catalyst PL were not affected by hemolysis, icterus, or lipemia, even when extreme. Measurements were stable for a minimum of 1 week when samples were stored at room temperature, and for 3 weeks when stored at 4°C, −20°C, or −80°C. Serum lipase activity was negligible in dogs with PAA.

**Conclusions and Clinical Importance:** The Catalyst® PL was shown to be analytically valid for use in dogs and cats.

## 137 ABSTRACT GI02: Calcifediol Supplementation Safely Increases 25‐Hydroxyvitamin D Concentrations in Dogs with Protein‐Losing Enteropathy

### 137.1 **Lauren Reynolds** ^1^; Adam Rudinsky^2^, DVM, MS, DACVIM (SAIM); Jenessa Winston^2^, DVM, PhD, DACVIM (SAIM); Valerie Parker^2^, DVM, DACVIM (SAIM, Nutrition)

#### 137.1.1 ^1^Small Animal Internal Medicine Resident, The Ohio State University; ^2^The Ohio State University

**Background:** Dogs with protein losing enteropathy (PLE) have decreased 25‐hydroxyvitamin D [25(OH)D] concentrations, presumably partly due to malabsorption. It is unknown how dogs with PLE compare to healthy dogs when receiving calcifediol [25(OH)D] supplementation.

**Objectives and Hypothesis:** To compare the short‐term effects of 25(OH)D supplementation in healthy dogs and dogs with PLE. Our hypothesis is that supplementation will increase serum 25(OH)D concentrations in dogs with PLE to a lesser extent than in healthy dogs.

**Animals:** Client‐owned dogs with PLE (*n* = 9) and healthy dogs (*n* = 10).

**Methods:** All dogs received a 25(OH)D supplement at a dose of 2.3 mcg × (BWkg)^0.75^ once daily for 21 days. Ionized calcium, chemistries, and 25(OH)D concentrations were measured at baseline, after 21 days of supplementation, and 21 days after the supplement was discontinued.

**Results:** Mean + SD serum concentrations of 25(OH)D are presented. At baseline, 25(OH)D was 30.2+21.1 nmol/L in dogs with PLE, compared to 78.9+19.6 nmol/L in healthy dogs. After supplementation, 25(OH)D significantly increased to 127.1+75.3 nmol/L in dogs with PLE and 276.4+66.5 nmol/L in healthy dogs, respectively (*p* < 0.01). The total increase in 25(OH)D concentrations was greater in healthy dogs compared to PLE dogs (*p* < 0.01). 25(OH)D concentrations decreased in both groups after cessation of supplementation. No adverse effects related to supplementation were identified.

**Conclusions and Clinical Importance:** Compounded 25(OH)D was effective at increasing serum 25(OH)D concentrations in dogs with PLE and healthy dogs, albeit with a lower total increase in dogs with PLE.

**Figure:**
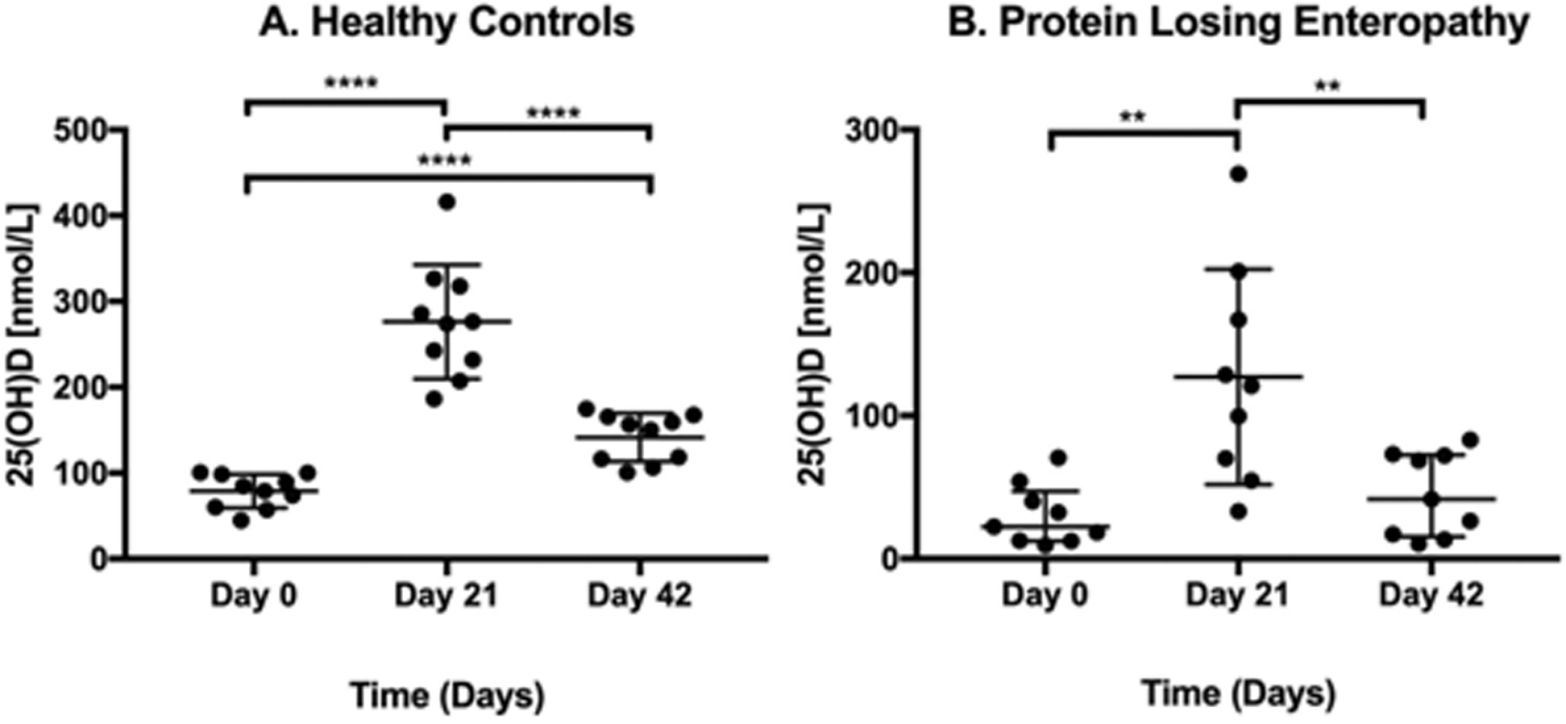
**Figure 1** (A) Vitamin [25(OH)D] in healthy dogs at baseline (Day 0), after 21 days of supplementation (Day 21), and 21 days after discontinuing supplementation (Day 42). (B) Vitamin [25(OH)D] in dogs with protein losing enteropathy (PLE) at baseline (Day 0), after 21 days of supplementation (Day 21), and 21 days after discontinuing supplementation (Day 42).

## 138 ABSTRACT GI03: Gastrointestinal Ulcers and Masses Diagnosed by Video Capsule Endoscopy in 26 Dogs

### 138.1 **Andrew Poon** ^1^; Julia Luca^2^, BSc, MSc; Alice Defarges^3^, DVM, MSc, DACVIM

#### 138.1.1 ^1^Resident in Small Animal Internal Medicine, VCA Canada and University of Guelph; ^2^Veterinary Student, Clinical Studies, Ontario Veterinary College, University of Guelph; ^3^Associate Professor, Clinical Studies, Ontario Veterinary College, University of Guelph

**Background:** Video capsule endoscopy (VCE) is a non‐invasive screening tool for gastrointestinal (GI) signs in dogs.

**Objective:** Evaluate the diagnostic value of VCE for identifying GI ulcers and masses in dogs.

**Methods:** Retrospective study of 26 dogs with VCE findings of GI masses or ulcers (March 2020 to April 2024). Data collected included signalment, VCE findings, follow‐up testing, and clinical outcomes based on medical record when available.

**Results:** Indications for performing VCE included GI signs, overt GI bleeding and anemia. The median weight and age were 17.8 kg (range, 7.4–40.5) and 7.5 years (range, 0.25–14), respectively. Twenty‐three of 26 dogs (88%) had GI ulcers: 13 gastric, 5 duodenal, 3 mid‐SI, and 2 colonic. Nineteen of 23 dogs (83%) reported prior abdominal imaging, showing no ulcers. Three of 26 dogs (12%) were diagnosed with masses on VCE: 2 jejunal, 1 gastric. Nine of 26 (35%) cases performed follow‐up testing after VCE study for persistent GI signs or GI bleeding. Conventional endoscopy was done on 9 dogs within 59 days (range, 0–196), confirming a large gastric ulcer in 1 dog. A vascular mass and jejunal mass were confirmed by endoscopic and surgical biopsies respectively in 2/3 (67%) cases with GI masses. In 17/26 cases (65%) without follow‐up testing, 10 treated with gastroprotectants had favorable outcomes (59%, 95% CI: 36%–78.4%), 4 were euthanized and 3 were lost to follow‐up.

**Conclusions and Clinical Importance:** VCE could be considered as an adjunct screening tool to detect GI ulcers or mid small intestinal masses in dogs.

## 139 ABSTRACT GI04: Effect of Low Dose Azithromycin on the Fecal Microbiome and Fecal Bile Acids in Dogs

### 139.1 **Colin Smith** ^1^; Roman Husnik^2^; Frederic Gaschen^3^; Claudia Kirk^2^; Jan Suchodolski^4^

#### 139.1.1 ^1^Internal Medicine Resident, University of Tennessee; ^2^University of Tennessee; ^3^Louisiana State University; ^4^Texas A&M University

**Background:** Azithromycin has prokinetic effects at low doses in people, cats, and anecdotally, in dogs; however, antibiotics are known to negatively impact the fecal microbiome and metabolome.

**Hypothesis/Objectives:** To prospectively evaluate the effect of azithromycin at a low dose on the fecal microbiome and fecal bile acids over time.

**Animals:** Eleven healthy adult research dogs.

**Methods:** Dogs received azithromycin (2 mg/kg PO q 8 h) for 8 days and then were monitored for 35 days. Fecal samples were collected before (day 1), during (day 6), and post‐azithromycin administration (days 11, 26, 36, 45), and fecal scored daily. The microbiome was assessed using qPCR to determine the abundance of 16 core bacterial taxa and the Dysbiosis Index (DI). Fecal concentrations of conjugated and unconjugated bile acids (UBAs) were measured on day 1, 11, and 45.

**Results:** Fecal scores and the DI increased during azithromycin administration (*p* < 0.001 and *p* = 0.068, respectively). Most bacterial taxa showed decreased abundance on day 6, including *Peptacetobacter hiranonis* (*p* < 0.001), with most recovering by day 45. Only *Enterococcus* increased on day 6 (*p* < 0.001). An unexpected decrease in *Escherichia coli* and *Streptococcus* abundances occurred during azithromycin administration (*p* = 0.009 and *p* = 0.053, respectively). Secondary UBAs decreased on day 11 (*p* = 0.006), but recovered by day 45.

**Conclusions and Clinical Importance:** Low dose azithromycin causes a mostly reversible dysbiosis and decrease in secondary fecal UBAs. Decreased abundance of *E. coli* and *Streptococcus* may also occur, which differs from other broad‐spectrum antibiotics.

## 140 ABSTRACT GI05: Evaluation of an Outpatient Protocol for the Management of Acute Pancreatitis in Dogs

### 140.1 **Caylen Erger** ^1^; Katie Mauro^2^, DVM, DACVECC; Isabelle Mendoza‐White^3^; Marilyn Brink^4^, DVM; Susanne Heartsill^5^, DVM, DABVP (Canine and Feline Practice); Harry Cridge^6^, MVB, MS, PG Cert Vet Ed, DACVIM (SAIM), DECVIM‐CA, FHEA, MRCVS

#### 140.1.1 ^1^Internal Medicine Resident, College of Veterinary Medicine, Michigan State University; ^2^Assistant Professor of Emergency and Critical Care, College of Veterinary Medicine, Michigan State University; ^3^DVM Student, College of Veterinary Medicine, Michigan State University; ^4^Medical Director, Animal Emergency & Specialty Hospital; ^5^Director of Companion Animal Veterinary Services, Ceva Animal Health, LLC; ^6^Associate Professor of Small Animal Internal Medicine, College of Veterinary Medicine, Michigan State University

**Background:** Inpatient care for pancreatitis is costly and can be prohibitive. Development of an outpatient protocol may reduce cost of care.

**Hypothesis/Objectives:** Evaluate the feasibility and analgesia afforded by a standardized outpatient protocol for pancreatitis in dogs.

**Animals:** 11 client‐owned animals with suspected pancreatitis.

**Methods:** Prospective observational study. All dogs received the following protocol: fuzapladib sodium (0.4 mg/kg IV q 24 h for 3 days), subcutaneous fluids (up to 20 mL/kg SQ q 8 h), hydromorphone (0.01 mg/kg/h delivered by a wearable subcutaneous infusion device), anti‐emetics (maropitant citrate 2 mg/kg PO q 24 h &/or ondansetron 0.5 mg/kg PO q 8 h), and nutrition (low‐fat diet). Dogs were assessed every 24 h for 3 days via physical examination and calculation of modified clinical activity index (MCAI) and Glasgow pain scores (GPS).

**Results:** All dogs survived during study follow‐up. One dog was suspected to develop Well's syndrome, but a drug adverse effect could not be ruled out. MCAI decreased from day 0 (median: 7, IQR: 7) to day 3 (median 3.5, IQR: 5). GPS decreased from day 0 (median: 6, IQR: 7) to day 3 (median 0, IQR: 0). One dog was recommended to be hospitalized on day 3 due to vomiting and dehydration. A second dog was hospitalized at the owner's request.

**Conclusions and Clinical Importance:** Dogs with suspected pancreatitis can be treated on an outpatient basis with only 1 dog receiving a recommendation for hospitalization during the study. Randomized controlled studies are needed to compare this protocol to inpatient management and to allow for cost comparison.

## 141 ABSTRACT GI06: Tolerability of At‐Home Nasoesophageal and Nasogastric Tube Feedings in Dogs and Cats

### 141.1 **Christina Mangan** ^1^; Julie Churchill^2^, DVM, PhD, DACVIM (Nutrition); Tracy Hill^3^, DVM, PhD, DACVIM (SAIM); Brian Husbands^4^, DVM, DACVIM (Oncology); Laura Motschenbacher^5^, DVM, DACVIM (SAIM); Kathy Stuebner^5^; Rachel Raser^5^; Jennifer Novak^5^; Abigail Leon^5^; Kelly Makielski^5^, DVM, MS, DACVIM (SAIM); Emily Coffey^5^, DVM, PhD, DACVIM (SAIM)

#### 141.1.1 ^1^Small Animal Internal Medicine Resident, University of Minnesota; ^2^University of Minnesota; ^3^Ethos Veterinary Health; ^4^The Ohio State University; ^5^University of Minnesota

**Background:** Nasoesophageal (NE) and nasogastric (NG) feeding tubes are an option for enteral nutrition in patients unwilling/unable to eat on their own, yet many veterinarians remain hesitant about their outpatient use. Increased understanding of their tolerability and complications could improve comfort with outpatient NE/NG tube use amongst veterinarians.

**Hypothesis/Objectives:** To describe the use and complications of at‐home NE/NG feeding tubes in dogs and cats. We hypothesized these tubes offer a well‐tolerated method for at‐home nutritional support.

**Animals:** 42 patients (19 dogs, 23 cats) with NE or NG feeding tubes placed for at‐home nutritional support.

**Methods:** Medical records for dogs and cats with NE or NG tubes placed for outpatient use in 2023 were retrospectively reviewed. Indication for tube placement, feeding plan details, maximum RER achieved, and complications were recorded.

**Results:** Most tubes placed were NG (*n* = 40, 95%), and used in‐hospital prior to discharge (*n* = 36, 86%). The most common primary disease process was pancreatitis (*n* = 12, 33%). The median tube placement duration was 6 days (range 1–10 days). Tube feedings achieved an average maximum RER of 85% (sd ±34%). The most common owner‐reported complications were diarrhea (*n* = 10, 28%) and vomiting (*n* = 9, 25%). Aspiration pneumonia was documented in one dog with vomiting.

**Conclusions and Clinical Importance:** Outpatient NE/NG tubes were generally well‐tolerated. Diarrhea and vomiting were reported complications, though whether these resulted from tube feedings or the primary disease is unknown. NE and NG tubes are a viable option for at‐home enteral nutritional support in select patients.

## 142 ABSTRACT GI07: Establishment and Characterization of Canine Pancreatic Organoids

### 142.1 **Meg Nakazawa** ^1^; Yoko Ambrosini^2^, DVM, MPVM, PhD, DACVIM (SAIM); Harry Cridge^3^, MVB, MS, PG Cert Vet Ed, DACVIM (SAIM), DECVIM‐CA, FHEA, MRCVS

#### 142.1.1 ^1^Postdoctoral Researcher, Washington State University; ^2^Assistant Professor, Washington State University; ^3^Associate Professor, Michigan State University

**Background:** Disorders of the exocrine pancreas, such as pancreatitis, exocrine pancreatic insufficiency, and pancreatic neoplasia, lack adequate models to study their molecular pathophysiology. Canine pancreatic organoids provide a promising platform for investigating disease mechanisms and evaluating therapeutic interventions, with significant translational potential.

**Hypothesis/Objectives:** The aims of the study were to (i) establish a reproducible protocol for generating canine pancreatic organoids, (ii) assess their viability following 1–3 days of shipment on ice, and (iii) validate their utility as an *in vitro* model that mimics the molecular complexity of native pancreatic tissue.

**Animals:** Residual pancreatic tissue from 4 dogs that were recently euthanized due to non‐pancreatic disorders.

**Methods:** Pancreatic tissue was shipped on ice and used for organoid generation. Cells were embedded in 3D matrices and cultured in optimized media. The expression of endocrine (glucagon, somatostatin), exocrine (pancreatic α‐amylase), and ductal cell marker (cytokeratin 19: KRT19) was evaluated in organoids using RT‐qPCR and compared with pancreatic tissue.

**Results:** Organoids were successfully generated from all samples and exhibited cystic or bleb‐like morphologies, requiring passaging every 7–8 days. Apical‐in polarity, lumenal F‐actin staining, and E‐cadherin expression confirmed structural integrity and epithelial polarization. Organoids showed a 7‐fold increase in ductal marker KRT19 expression but reduced endocrine and exocrine marker expression compared to donor tissue.

**Conclusions and Clinical Importance:** Canine pancreatic organoids can be reproducibly generated from post‐euthanasia tissue and are primarily composed of ductal cells in Wnt‐rich media. These organoids provide a foundation for creating advanced models to study pancreatic biology and disease mechanisms.

## 143 ABSTRACT GI08: Viable Bacteria Isolated From Lyophilized Stool Used for Fecal Microbiota Transplantation

### 143.1 **Holly Ganz** ^1^; Nancy Alvarado^2^; Guillaume Jospin^2,3^; Ruchita Uttarwar^2^; Niokhor Dione^2^, DVM, PhD

#### 143.1.1 ^1^Chief Science Officer, AnimalBiome; ^2^AnimalBiome; ^3^UC Davis

**Background:** Fecal microbiota transplantation (FMT) is used to address gut dysbiosis‐related diseases in companion animals. Lyophilization and powdering of fecal material offer a convenient alternative to fresh transfers, but the mechanisms of action remain poorly understood. This study aimed to identify viable bacterial species in lyophilized FMT powder obtained from cats and dogs contributing to a stool bank.

**Objectives:** To determine the diversity and viability of bacterial species in lyophilized fecal material from domestic cats and dogs.

**Animals:** Lyophilized fecal samples were collected non‐invasively from five healthy cats and five healthy dogs enrolled in a stool bank in Northern California.

**Methods:** Fecal samples were mixed with glycerol before freeze drying and powdering. We employed culturomics techniques, including direct inoculation in various culture media and pre‐incubation in blood culture bottles, to isolate anaerobic, aerobic, fastidious, and slow‐growing bacteria. Identification was performed using MALDI‐TOF mass spectrometry, 16S rRNA gene sequencing, and whole genome sequencing of unique isolates.

**Results:** In cats, 72 bacterial species were isolated, including 32 from the core microbiome, 17 novel species, and seven novel genera. In dogs, more than 70 species were identified, including short‐chain fatty acid (SCFA) producers, inflammation reducers, and known probiotics for companion animals.

**Conclusions:** Lyophilized stool retains a diverse assemblage of viable bacteria, particularly spore formers capable of surviving freeze‐drying. These findings support the potential of lyophilized FMT as a viable therapeutic option for gut microbiome restoration.

## 144 ABSTRACT GI09: Localization of Glucose Transporter 2 RNA in Small Intestine of Dogs With Chronic Inflammatory Enteropathy

### 144.1 **Chih‐Chun Chen** ^1^; Madison Mascorro^2^; Amanda Blake^2^; Rachel Pilla^2^; Katherine Tolbert ^2^; Jan Suchodolski^2^; Paula Giaretta^2^

#### 144.1.1 ^1^Graduate Research Assistant, Gastrointestinal Laboratory, Texas A&M; ^2^Gastrointestinal Laboratory, Texas A&M University

**Background:** Soluble carbohydrates provide nutritional value and are digested and absorbed mainly in the small intestine. Previous studies showed increased fecal concentrations of glucose and other carbohydrates in a subset of dogs with chronic inflammatory enteropathy (CIE) compared to healthy controls (HC). Therefore, malabsorption was suspected with dysregulation of the intestinal carbohydrate transporter possibly playing a role. However, the localization of carbohydrate transporters has not been described in dogs.

**Objective:** Describe intestinal RNA localization of *SLC2A2* (gene encoding the glucose transporter 2) in dogs with CIE and HC.

**Animals:** Duodenum biopsies from 8 HC and 23 dogs with CIE; ileum biopsies from 9 HC and 19 dogs with CIE.

**Methods:** Retrospective cross‐sectional study. RNA expression of SLC2A2 in duodenum and ileum was assessed by *in situ* hybridization and quantified by QuPath as dots per 2.37 mm^2^.

**Results:** SLC2A2 RNA expression was identified only in the duodenum of both HC and CIE dogs. The nucleus and cytoplasm of epithelial cells in villi and crypts were positive. No expression was identified in the lamina propria, submucosa, and muscularis. The RNA expression of *SLC2A2* in villi and crypts did not differ between HC and CIE (Mann‐Whitney U tests, *P* = 0.34 and *P* = 0.67, respectively).

**Conclusions and Clinical Importance:** RNA expression of *SLC2A2* is distributed in duodenal epithelial cells but absent in ileum of dogs. As expression did not differ between HC and dogs with CIE, the cause of increased fecal glucose concentrations in the latter remains to be determined.

## 145 ABSTRACT GI10: Exploring the Gut Microbiome of Ferrets: Associations With Health Status and Microbial Composition

### 145.1 **Elisa Scarsella** ^1^; Zara Marfori^2^; Guillaume Jospin^2^; Niokhor Dione^2^; Holly Ganz^2^

#### 145.1.1 ^1^Director, Molecular Biology, AnimalBiome; ^2^AnimalBiome

**Background:** The gut microbiome supports the health and well‐being of all animals. Still its composition and diversity in relation to health status remain underexplored in many animals, particularly for less well studied exotic species, such as the domestic ferret.

**Objectives:** The objective of this study was to characterize and compare the fecal microbiome of healthy ferrets and those reported to have chronic enteropathy (CE) in order to identify insights that may enhance the management of these conditions.

**Methods:** Fecal samples were collected from a cohort of 45 healthy ferrets and 25 ferrets presenting with various gastrointestinal diseases. Microbial DNA was extracted from the samples, and full‐length 16S rRNA gene sequencing was performed using PacBio technology to provide species‐level resolution.

**Results:** The Shannon index was used to assess alpha diversity and did not differ significantly between the two groups (*p* = 0.39). Beta diversity was assessed using the Bray‐Curtis dissimilarity matrix, which revealed distinct clustering of samples based on health status (PERMANOVA, *p* = 0.04). Spearman rank correlation was used to generate a pattern plot that highlighted the features correlated with key taxa of interest associated with health status. *Enterococcus hirae* and *Streptococcus lutetiensis* were correlated with the CE group, while *Enterococcus faecium*, *E. faecalis*, *Streptococcus equorum*, and *S. saprophyticus* were correlated with the healthy group.

**Conclusion:** These research findings enhance our understanding of the fecal microbiome of domestic ferrets and highlight the need for species‐level resolution when characterizing bacterial composition, particularly members of *Enterococcus* and *Streptococcus*.

## 146 ABSTRACT GI11: Effect of Amoxicillin‐Clavulanate Treatment on the Fecal Lipidome in Healthy Adult Cats

### 146.1 **Teresa Schmidt** ^1^; Jamie Hui^2^; Paula Giaretta^3^; Jan Suchodolski^3^; Frederic Gaschen^2^

#### 146.1.1 ^1^Post‐Doctoral Research Associate, Gastrointestinal Laboratory, Texas A&M University; ^2^Louisiana State University; ^3^Gastrointestinal Laboratory, Texas A&M University

**Background:** Recent evidence indicates that enteropathies are linked to changes in intestinal lipid metabolism, which correspond to alterations observed in the fecal lipid profiles of cats and dogs with acute and chronic enteropathies. Changes in specific fecal lipids (e.g., arachidonic acid, nervonic acid, cholesterol) have been recently reported as potential indicators of intestinal damage in companion animals. This study aimed to evaluate whether antibiotics, commonly given to animals with enteropathies, affect markers of lipid metabolism.

**Objective:** Assessing the effect of amoxicillin‐clavulanate (AMC) treatment on the fecal lipidome in cats.

**Animals:** 13 healthy adult colony cats.

**Methods:** Previous prospective longitudinal study. Cats on a standardized diet were treated with AMC (12.5–15 mg/kg BW PO q 12 hrs) for 10 days. Fecal long‐chain fatty acid and sterol profiles from baseline (day 0) and five follow‐up samples (days 10–66) were assessed using a previously validated GC‐MS assay. Friedman test and Dunn's test for multiple comparisons were utilized for statistical analysis.

**Results:** AMC induced no significant changes in the feline fecal lipidome. None of the individual lipid profiles differed in response to the treatment. The markers previously reported as potential indicators of mucosal damage, nervonic acid (*p* > 0.9999), cholesterol (*p* > 0.9999), and arachidonic acid (*p* = 0.7111), were not significantly altered after treatment (day 10).

**Conclusions and Clinical Importance:** The investigated fecal lipids were not affected by AMC. In the future, these biomarkers could therefore be useful in cats with intestinal diseases that have received antibiotics, as they remain unaffected by a common treatment.

## 147 ABSTRACT GI12: Microbiome and Liver Enzymes in Dogs with Chronic Enteropathies Receiving Fecal Microbiota Transplantation

### 147.1 **Maria Chiara Sabetti** ^1^, DVM, PhD; Milena Brigano^2^; Mariangela Colosini^2^; Tommaso Danese^2^; Amanda Blake^3^; Cecilia Quintavalla^4^; Andrea Corsini^2^; Jan Suchodolski^3^; Rachel Pilla^5^

#### 147.1.1 ^1^ECVCP (Resident), University of Parma, Italy; ^2^University of Parma, Italy; ^3^Department of Small Animal Clinical Sciences, Gastrointestinal Laboratory, Texas A&M University, College Station, TX, USA; ^4^Department of Veterinary Sciences, University of Parma, Italy; ^5^Department of Veterinary Medicine and Animal Sciences, University of Lodi, Milan

**Background:** In chronic enteropathies (CE), intestinal dysbiosis and loss of colonization by *Clostridium* (*Peptacetobacter*) *hiranonis* leads to decreased production of secondary bile acids (BA), which may impact the liver due to BA dysmetabolism. Fecal microbiota transplantation (FMT) can restore BA conversion by reintroducing *C. hiranonis* and, therefore, may impact the liver.

**Objective:** Characterize the response of fecal microbiome and serum liver enzymes to administration of FMT in dogs.

**Animals:** 10 dogs with CE with no or incomplete response to dietary changes.

**Methods:** Prospective observational study. CIBDAI, liver enzymes (ALT, AST, SAP), dysbiosis index (DI) were monitored before (T0), 7 (T1), and 30 days (T2) after FMT. Statistics were performed with Friedman's test.

**Results:** Significant CIBDAI improvement was observed at T1 (1.5, range 0–7) and T2 (1, range 0–9) vs T0 (3.5, range 2–10) (*P* = 0.02). No statistically significant differences were observed in liver enzyme activities which remained within the range of normal. DI decreased at T2 (–4.2, range: –3.1 to 1.9) vs T0 (–2.3, range –5.5 to 5) and T1 (–2.2, range –5.5 to 3.3) (*P* = 0.02).

**Conclusions and Clinical Importance:** FMT improved the clinical course of gastrointestinal signs without affecting liver enzymes. However, at baseline only 3/10 dogs had decreased abundance of *C. hiranonis*, none had increased liver enzymes, and clinical activity was low (median CIBDAI 3.5) suggesting most dogs in this cohort had mild dysbiosis and disease. Measuring fecal and serum BA may help identify smaller effects not captured by liver enzyme measurements

## 148 ABSTRACT GI13: Correlation Between Serum Cobalamin Concentration, Histopathologic Changes, and Outcome in Cats With Chronic Enteropathy

### 148.1 **Mina Park** ^1^; Joao Cavasin^2^, DVM, DACVP (Anatomic); Paula Giaretta^2^, MedVet, MS, PhD, DACVP (Anatomic); Nicole Dausend^3^, DVM; Chee‐Hoon Chang^4^, DVM, MS; Stefan Keller^5^, DVM, DrMedVet, PhD, DECVP; Judit Magnusson Wulcan^6^, DVM, MSc, DACVP; Valerie Freiche^7^, DMV, PhD, DESV (MI); Sina Marsilio^8^, DrMedVet, PhD, DACVIM (SAIM), DECVIM (Companion Animals)

#### 148.1.1 ^1^DVM Candidate, School of Veterinary Medicine, University of California Davis, CA, USA; ^2^Clinical Assistant Professor, Department of Small Animal Clinical Sciences, School of Veterinary Medicine and Biomedical Sciences, Texas A&M University, TX, USA; ^3^Doctoral Candidate, Department of Veterinary Medicine and Epidemiology, School of Veterinary Medicine, University of California Davis, CA, USA; ^4^PhD Candidate, Department of Small Animal Clinical Sciences, School of Veterinary Medicine and Biomedical Sciences, Texas A&M University, TX, USA; ^5^Assistant Professor, Department of Pathology, Microbiology, and Immunology, School of Veterinary Medicine, University of California Davis, CA, USA; ^6^PhD Candidate, Department of Pathology, Microbiology, and Immunology, School of Veterinary Medicine, University of California Davis, CA, USA; ^7^L'école Nationale Vétérinaire d'Alfort; ^8^Assistant Professor, Department of Veterinary Medicine and Epidemiology, School of Veterinary Medicine, University of California Davis, CA, USA

**Background:** Hypocobalaminemia is commonly seen as a surrogate marker for intestinal lesions and as a diagnostic guide for biopsy collection of the ileum. However, the correlation between serum cobalamin concentration and histopathologic lesions in the small intestine has not been investigated in cats.

**Hypothesis/Objectives:** To determine the correlation between serum cobalamin concentration, histopathologic lesions, and outcome in cats with chronic enteropathy.

**Animals:** Formalin‐fixed, paraffin‐embedded duodenum and ileum samples from 73 cats with chronic enteropathy (CE).

**Methods:** Retrospective study. Tissue archives were systematically searched for cases of CE with serum cobalamin results. Samples were scored by a pathologist blinded to clinicopathological data using a modified World Small Animal Veterinary Association (WSAVA) scoring system. Outcomes were assessed using medical records and client questionnaires utilizing a modified Feline Chronic Enteropathy Activity Index (FCEAI) and 4‐point outcome scale (0, worse; 1, unchanged, 2; improved; 3, remission).

**Results:** A total of 73 cases were identified and analyzed. Serum cobalamin concentrations were significantly negatively correlated with total WSAVA scores (*P* < 0.001) and cellular infiltrate scores (*P* < 0.001) but not with architectural scores. Out of 51 cases with hypocobalaminemia, 5 cases showed histopathologic lesions exclusively in the duodenum, while the ileum remained unaffected. Serum cobalamin concentration was not associated with the FCEAI or clinical outcome.

**Conclusions and Clinical Importance:** Serum cobalamin concentrations in cats with CE showed a significant negative correlation with histopathologic lesions, particularly cellular infiltration in the duodenum and the ileum. However, hypocobalaminemia may not always be indicative of ileal lesions in cats.

## 149 ABSTRACT GI14: Blood Reticulocyte Hemoglobin Concentration as a Marker of Iron‐Deficient Erythropoiesis in Canine Chronic Inflammatory Enteropathy

### 149.1 **Romy Heilmann** ^1^; Els Acke^2^, DECVIM, PhD, MANZCVS; Emma Edelmann^3^; Florentine Stacho^3^

#### 149.1.1 ^1^Professor of Small Animal Internal Medicine, College of Veterinary Medicine, University of Leipzig, Germany; ^2^Idexx Laboratories; ^3^College of Veterinary Medicine, University of Leipzig, Germany

**Background:** Non‐regenerative normocytic normochromic anemia is documented in some dogs with chronic inflammatory enteropathy (CIE), but iron deficiency anemia (IDA) is rare. Decreased reticulocyte hemoglobin (retic‐HgB) concentration is an indicator of iron‐deficient erythropoiesis (IDE), which precedes the development of IDA.

**Objectives:** To investigate the occurrence of IDE in canine CIE by routine hematological parameter assessment, including blood retic‐HgB concentrations.

**Animals:** 156 pet dogs with CIE (43 with protein‐losing enteropathy [PLE]), 34 dogs with portosystemic shunt (PSS, disease controls), and 50 blood donors (healthy controls).

**Methods:** Retrospective case‐control study, including patient and clinicopathologic data extracted from electronic medical records. Results of retic‐HgB and other variables were compared and tested for possible associations with clinical and clinicopathologic variables. Delta‐HgB was calculated as [(retic‐HgB in pg) – (MCH in pg)].

**Results:** 13% of dogs with CIE were anemic (PLE: 37%, non‐PLE: 4%), but erythrocyte indices were not different from healthy dogs. Retic‐HgB concentrations in CIE patients differed significantly from PSS but not from healthy controls, and correlated with MCH (*ρ* = 0.55), MCV (*ρ* = 0.43), and reticulocyte count (*ρ* = 0.28). Retic‐HgB concentrations did not differ between PLE and non‐PLE cases, regardless of anemia presence, but PLE dogs had higher platelet counts (*P* = 0.0002), erythrocytes of smaller volume (*P* = 0.0246) and less hemoglobin (*P* = 0.0008), and less frequently a negative delta‐HgB.

**Conclusions and Clinical Importance:** The overall prevalence of IDE was low (8%) in CIE patients in this study. However, an iron‐restricted state appears more common in PLE cases, which highlights the need to monitor these cases and carefully consider iron supplementation.

## 150 ABSTRACT GI15: Parasite and Pathogen Incidence and Frequency of Testing in a Companion Animal Stool Bank

### 150.1 **Holly Ganz** ^1^; Rebecca Oliver^2^; Rebecca Smith^2^

#### 150.1.1 ^1^Chief Science Officer, AnimalBiome; ^2^AnimalBiome

**Background:** Stool banks provide screened material for fecal microbiota transplantation (FMT), yet optimal donor screening and testing frequency guidelines in veterinary medicine are not well defined. Using six years of data, we estimated the probability of new parasite or pathogen infections arising in a companion animal stool bank and assessed whether this differed between cat and dog donors.

**Objectives:** We aimed to determine: (1) the parasites and pathogens found in stool bank donors, (2) their incidence, and (3) how undetected infections vary with different testing frequencies. We hypothesized that reduced testing frequency increases the probability of undetected infections and that testing recommendations are similar for cats and dogs.

**Animals:** We analyzed records from 158 healthy cats and dogs enrolled in a stool bank in California (March 2018–December 2023). Fecal samples were obtained noninvasively.

**Methods:** Monthly fecal tests for parasites and pathogens were conducted using Antech KeyScreen and the IDEXX diarrhea panel. Incidence rates were calculated, and probabilities of undetected infections were modeled for different testing intervals (monthly, every three, six, or 12 months).

**Results:** A total of 34 positive parasite/pathogen cases were identified from 1,275 dog tests and 506 cat tests. The most common pathogens were FeCoV, *Giardia*, and tapeworms. Probability modeling showed increased risk of undetected infections with less frequent testing.

**Conclusions:** Monitoring frequency should align with acceptable risk levels. Given the observed parasite/pathogen incidence in this region, monthly testing may be warranted for both cat and dog donors to minimize transmission risk to FMT recipients.

**Figure:**
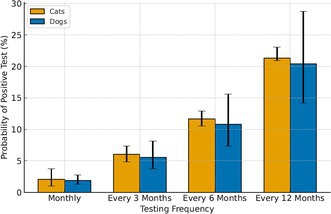
Figure 1. The probability of a positive test result is expected to increase as testing frequency decreased in cat and dog donor populations (based on data from a stool bank in Northern California)

## 151 ABSTRACT GI16: Safety, Tolerability, and Pharmacokinetics of a Canine Monoclonal Antibody for Treatment of Chronic Inflammatory Enteropathy

### 151.1 **Kristy Longpre** ^1^; Stephanie Pierce^2^, DVM, DACVIM (SAIM); Eden Bermingham^3^, DVM, MS, DACVCP; Caryn Thompson^4^, PhD; Hangjun Zhan^5^, Denise Bevers^6^, MBA

#### 151.1.1 ^1^Director of Operations, VETmAb Biosciences, Inc.; ^2^Director, Clinical Development, ACI Biosciences; ^3^Executive Director Product Development, Pharmacology, ACI Biosciences; ^4^President, Vista Research, LLC; ^5^VETmAb Biosciences, Inc.; ^6^Cofounder, President and CEO, Board Member, VETmAb Biosciences, Inc.

**Background:** Chronic inflammatory enteropathy (CIE) is characterized by recurrent clinical signs of vomiting, diarrhea, weight loss, and decreased appetite. There are no monoclonal antibody (mAb) therapies available for treatment of CIE in veterinary medicine. VMB‐C001a is a canine‐specific mAb under evaluation for treatment of CIE in dogs.

**Hypothesis/Objective:** To evaluate safety, tolerability, bioavailability, and pharmacokinetics (PK) of VMB‐C001a administered IV as a single dose or SC for 3 monthly doses.

**Animals:** 20 healthy beagles.

**Methods:** Dogs were randomized into one of 5 treatment groups as presented in Table 1. Physical examinations occurred monthly. CBC and serum chemistry were evaluated every two weeks or monthly. PK samples were collected at 0, 8, 24, 36, 48, 56 h and on days 3, 4, 7, 14, 21, 28 following each treatment.

**Table:** **Table 1.** Treatment groups

Treatment group	Number of dogs	Route	Dose frequency	Dose (mg/kg)	Test article	End of study date
1	4	SC	3× (day 0, 28, 56)	(volume matching 18 mg/kg VMB‐C001a)	PBS	Day 84
2	4	SC	3× (day 0, 28, 56)	3 mg/kg	VMB‐C001a	Day 84
3	4	SC	3× (day 0, 28, 56)	6 mg/kg	VMB‐C001a	Day 84
4	4	SC	3× (day 0, 28, 56)	18 mg/kg	VMB‐C001a	Day 84
5	4	IV	1× (day 0)	6 mg/kg	VMB‐C001a	Day 28

IV, intravenous; SC, subcutaneous

**Results:** There were no clinically significant adverse events in any treatment group. Clinical pathology parameters showed no clinically significant abnormalities or differences between treatment groups. VMB‐C001a concentrations following one IV or three SC doses are presented in Figure 1. The mean 6 mg/kg IV and SC half‐life was approximately 9.25 and 11.93 days, respectively. The accumulation ratio was 1.20, 1.30, and 1.50 for the 3, 6, and 18 mg/kg treatment groups. Absolute bioavailability of the 6 mg/kg SC treatment group was 89%.

**Figure:**
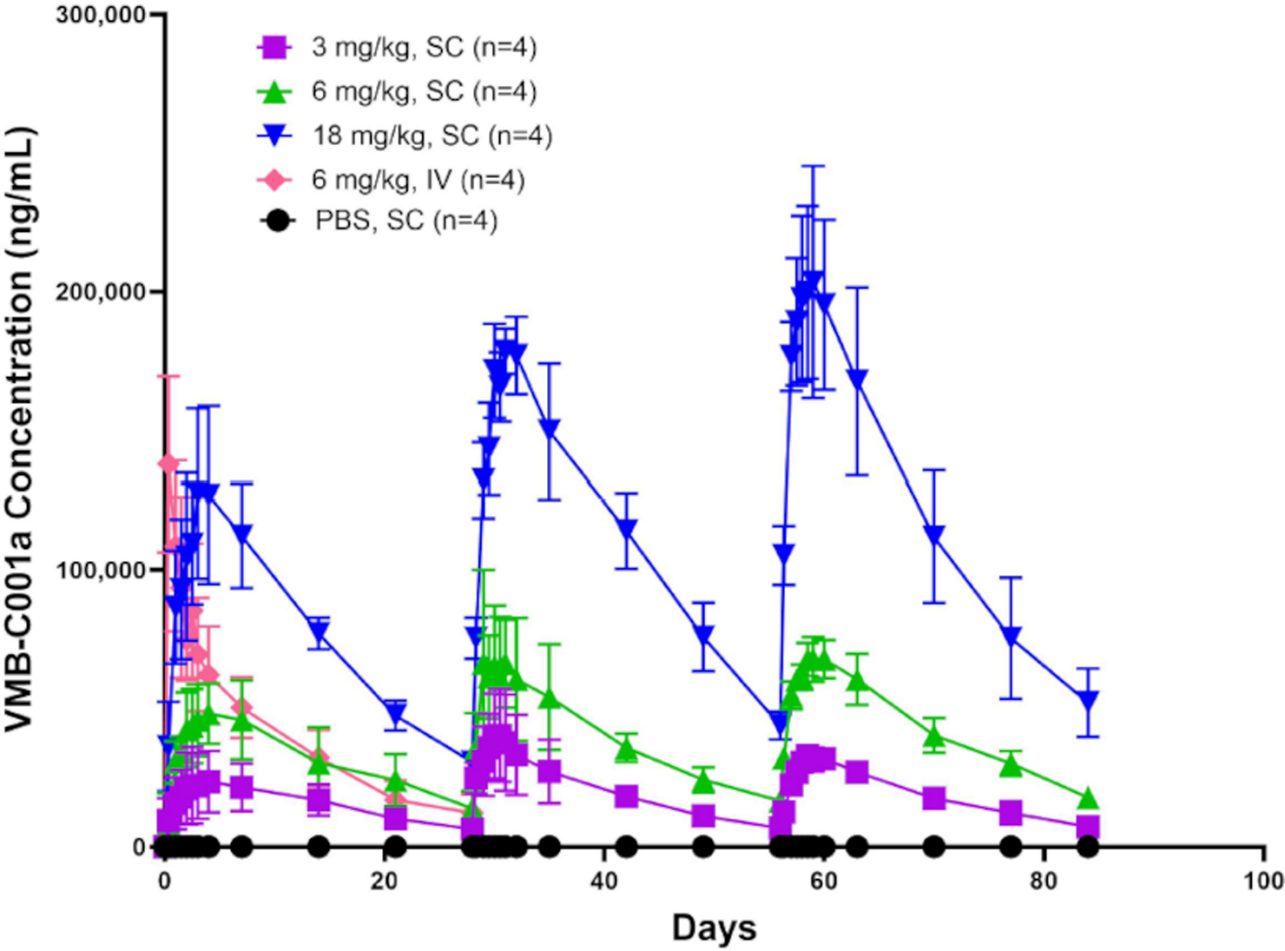
**Figure 1. Mean serum concentrations of VMB‐C001a following one (IV) or three (SC) doses**. Mean ± SD VMB‐C001a serum concentrations over time following: a single IV 6 mg/kg dose of VMB‐C001a; three monthly SC doses of VMB‐C001a at 3, 6, or 18 mg/kg; three monthly SC doses of PBS at the same volume as the 18 mg/kg treatment group.

**Conclusion and Clinical Importance:** Three monthly doses of VMB‐C001a were tolerated and safe at all dose levels and routes evaluated. PK results support monthly SC dosing.

## 152 ABSTRACT GI17: Daily Monitoring of Fecal Microbiota Transplantation Response in Dogs: Preliminary Insights From the Owners' Perspective

### 152.1 **Maria Chiara Sabetti** ^1^, DVM, PhD; Silvia Scorza^2^; Mariangela Colosini^2^; Tommaso Danese^2^; Cecilia Quintavalla^2^; Andrea Corsini^2^; Jan Suchodolski^3^; Rachel Pilla^4^

#### 152.1.1 ^1^ECVCP (Resident), University of Parma, Italy; ^2^University of Parma, Italy; ^3^Gastrointestinal Laboratory, Department of Small Animal Clinical Sciences, Texas A&M University, College Station, TX, USA; ^4^Department of Veterinary Medicine and Animal Sciences, University of Lodi, Milan, Italy

**Background:** Fecal microbiota transplantation (FMT) represents a promising therapeutic option for chronic enteropathy (CE) in dogs. Clinical response was assessed in various studies through CIBDAI scores calculated by veterinarians during follow‐up visits, however, day‐to‐day variations remain largely unexplored.

**Objective:** Assess clinical response to a single FMT enema through daily CIBDAI evaluations performed by owners.

**Animals:** 10 adult dogs with CE with no or incomplete response to dietary changes undergo to FMT.

**Methods:** Prospective observational study. CIBDAI scores were calculated by the attending veterinarian before (D0), 7 days (D7) and 30 days (D30) after FMT. CIBDAI scores were recorded by owners the day post‐FMT (D1) and daily for 30 days after the procedure, using a short questionnaire. Friedman's test was used to evaluate changes in CIBDAI over time, and descriptive statistics to describe the results.

**Results:** The daily questionnaire was well accepted by owners, with no complaints and almost no missing values. A significant improvement in veterinary‐calculated CIBDAI scores was observed from D0 to days 7 (D7), and 30 (D30) post‐FMT (*P* = 0.008). At D1, 8/10 dogs had reached CIBDAI<2, indicating clinically insignificant disease. The median number of relapse days, defined as days with a CIBDAI score ≥ D0, was 5 days (range: 0–13 days). Two dogs had no relapse days, while three dogs experienced > 10 non‐consecutive relapse days.

**Conclusions and Clinical Importance:** Our results suggest that continuous monitoring over multiple days, rather than on visit days, may provide a more accurate assessment of FMT efficacy.

## 153 ABSTRACT GI18: Retrospectively Characterizing Fecal Microbiota in Dogs With Acute Diarrhea Using qPCR, Metagenomics, and Machine Learning

### 153.1 **Jonathan Turck** ^1^; Stefan Unterer^2^, DVM, DrMedVet, Dr.habil, DECVIM (Companion Animals); Kathrin Busch^3^, DVM, DrMedVet, DECVIM (Companion Animals); Rachel Pilla^4^, DVM, PhD; Ivan Ivanov^5^, PhD; Jan Suchodolski^6^, MedVet, DrVetMed, PhD, AGAF, DACVM

#### 153.1.1 ^1^Graduate Research Assistant, Texas A&M University, TX, USA; ^2^Clinic for Small Animal Internal Medicine, Vetsuisse Faculty, University of Zurich, Switzerland; ^3^Clinic of Small Animal Medicine, Centre for Clinical Veterinary Medicine, Ludwig‐Maximilians‐Universität, Munich, Germany; ^4^Department of Veterinary Pathology, Hygiene and Public Health, University of Milan, Italy; ^5^Department of Veterinary Physiology and Pharmacology, Texas A&M University, TX, USA; ^6^Gastrointestinal Laboratory, Department of Small Animal Clinical Sciences, Texas A&M University, TX, USA

**Background:** Acute diarrhea (AD) is a common, clinically self‐limiting condition in dogs, often associated with increased abundance of pathobionts, but limited information is available on overall microbiome shifts.

**Objective:** Evaluate fecal microbiota in dogs with AD using qPCR and shotgun metagenomic sequencing.

**Animals:** Multiple timepoints from 78 dogs with AD treated without antibiotics and one sample from 79 healthy controls (HC).

**Methods:** Retrospective analysis was performed on feces using qPCR (AD, *n* = 78; HC, *n* = 79) for specific bacterial taxa at baseline and up to 1 month. Shotgun metagenomic sequencing was performed on 17 AD and 31 HC. A deterministic machine learning (ML) model was trained to distinguish AD from HC on the qPCR data.

**Results:** On qPCR, AD showed increased *E. coli*, *C. perfringens*, *Streptococcus*, and *Fusobacterium* (*p* < 0.05). *Faecalibacterium*, *Bifidobacterium*, and *Bacteroides* were decreased (*p* < 0.01). *C. difficile* was detected in 10.6% (5/47) of samples tested via qPCR. Only 4/78 (5%) dogs with AD had decreased *P. hiranonis*. Sequencing results were similar: *C. perfringens* and *E. coli* increased, while *P. hiranonis* remained unchanged (*p* = 0.6). The ML model accurately classified HC or AD 92% of the time (AUC: 0.97, 95% CI: 0.94–0.99), driven by increased *C. perfringens* and decreased *Bifidobacterium*. The abundance of *C. perfringens* decreased significantly over time (*p* < 0.01) in dogs with AD.

**Conclusions and Clinical Importance:** Acute diarrhea is characterized by transient, self‐limiting bacterial shifts, including increases in *C. perfringens*, with *C. difficile* playing no significant role. *P. hiranonis* was not significantly reduced, indicating preservation of bile acid conversion.

## 154 ABSTRACT GI19: Concurrent Management of Behavioral and Gastrointestinal Disorders in Dogs With Early‐Life Trauma Improves Clinical Outcomes

### 154.1 **Reagan Jennings** ^1^; M. Leanne Lilly^2^; Kyle Bohland^2^; Adam Rudinsky^2^; Jenessa Winston^2^

#### 154.1.1 ^1^Graduate Student, College of Veterinary Medicine, The Ohio State University, OH, USA; ^2^College of Veterinary Medicine, The Ohio State University, OH, USA

**Background:** In humans, early‐life trauma (ELT), including neglect, abuse, and social isolation, are linked to development of disorders of brain‐gut interaction (DBGI) which present with signs of both behavioral and gastrointestinal dysfunction. Similarly, behavioral signs often co‐occur with chronic gastrointestinal signs in dogs, indicating presumptive DBGI which may also be associated with ELT in this species.

**Hypothesis/Objectives:** This retrospective study investigated the relationship between ELT, chronic gastrointestinal signs, and behavioral disorders in dogs, hypothesizing that multimodal management of presumptive DBGI will improve clinical outcomes.

**Animals:** Twenty‐four client‐owned dogs evaluated by the behavioral medicine service and referred to internal medicine for comanagement of chronic gastrointestinal signs were included.

**Methods:** Data collected from 167 patient encounters included demographics, ELT history, GI signs, diagnostic findings, behavioral assessments, and treatments. Descriptive statistics were performed.

**Results:** Twenty‐four dogs were presumptively diagnosed with a DBGI. Nineteen dogs received concurrent behavioral (environmental management, behavior medications, training techniques, behavior modification, and/or safety equipment) and gastrointestinal (diet modification, fiber supplementation, probiotics, and/or fecal transplant) interventions. Of which, 76% (13/17) showed clinical improvement in both areas once comanaged. Eighty‐six percent (21/24) of dogs experienced ELT, with 90% (19/21) experiencing trauma between birth and 1 year; and 48% (10/21) during a critical neurodevelopmental window (birth to 12 weeks).

**Conclusions and Clinical Importance:** This is the first description of concurrent behavioral and gastrointestinal management of presumptive canine DBGI with clinical improvement. It also identifies ELT in this population. Further research is required to develop diagnostic/treatment protocols for DBGI in dogs.

## 155 ABSTRACT GI20: Serum and Fecal Bile Acid Profiles in Dogs With Chronic Enteropathy

### 155.1 **Amanda Blake** ^1^; Floris Dröes^2^; Joao Cavasin^2^; Robert Phillips^2^; Adrian Tinoco‐Nájera^2^; Joerg Steiner^2^; Jan Suchodolski^2^; Jonathan Lidbury^2^

#### 155.1.1 ^1^Associate Research Scientist, Gastrointestinal Laboratory, Texas A&M University, TX, USA; ^2^Gastrointestinal Laboratory, Texas A&M University, TX, USA

**Background:** Bile acids (BA) aid in lipid absorption and are key microbial metabolites, acting as signaling molecules in the intestines. Serum BA are poorly characterized in dogs with chronic enteropathy (CE).

**Objective:** Compare concurrently collected serum and fecal BA profiles between healthy control dogs (HC) and dogs with CE.

**Animals:** 26 HC and 8 dogs with CE (all privately owned).

**Methods:** Observational cross‐sectional study. Fecal and serum concentrations of 28 BA, including unconjugated, taurine‐ and glycine‐conjugated, and iso‐ and oxo‐BA were measured with a previously validated LC‐MS/MS method. Concentrations were compared between groups with Mann‐Whitney tests. Spearman's correlations were performed for each BA in serum and feces, adjusted by Benjamini‐Hochberg procedure (FDR 5%).

**Results:** Cholic acid and its epimer 7‐oxo‐deoxycholic acid were increased in serum of dogs with CE (median [min‐max] ng/mL; 34 [1–74] and 1.5 [0.1–8.5]) compared to HC (8.6 [0.4–239] and 0.5 [0.1–4.5]; *P* < 0.04). Deoxycholic acid and its epimer 12‐oxo‐lithocholic acid were decreased in feces of dogs with CE (ng/mg fecal dry matter; 80 [9–5,680] and 0.5 [0–1,316]) compared to HC (4618 [45–7,501] and 527 [1–3,140]; *P* < 0.04). Secondary BA deoxycholic (rho=0.608, *P* = 0.001) and hyodeoxycholic (rho=0.712, *P* = 0.001) acids were strongly positively correlated between serum and feces.

**Conclusions and Clinical Importance:** Dogs with CE had altered serum and fecal BA profiles compared to HC. Some secondary BA and their epimers are decreased, and some primary BA and their epimers are increased in CE compared to HC.

## 156 ABSTRACT GI21: Comparison of Gallbladder Volume and Emptying Between Treated Hypothyroid Dogs and Healthy Dogs

### 156.1 **Monique Paludetti** ^1^; Janaína Corrêa^2^; Carla Lorigados^2^; Fabio Teixeira^2^

#### 156.1.1 ^1^Doctor of Veterinary Medicine Student, School of Veterinary Medicine and Animal Science, University of São Paulo, São Paulo, São Paulo, Brazil; ^2^School of Veterinary Medicine and Animal Science

**Background:** Endocrine disorders are risk factors for gallbladder diseases, including bile sludge, mucocele, and cholelithiasis.

**Objectives:** To compare gallbladder volume (GBV) and emptying between controlled hypothyroid dogs and healthy dogs of the same age and diet.

**Animals:** Nine treated and controlled hypothyroid dogs without comorbidities (body condition score: 4–7, nine‐point scale) and 21 healthy age‐matched dogs.

**Methods:** All dogs were fed the same extruded diet for 60 days. After a 12‐h fast, initial GBV (V0) was measured via ultrasonography. Dogs then received levothyroxine sodium and 50% of their daily food intake. GBV was reassessed at 30, 60, and 120 min postprandially (Vx), using the formula: GBV = 0.52 × Length (cm) × Height (cm) × Width (cm), indexed to body weight (kg). Gallbladder ejection fraction (GBEF) was calculated as GBEF = [(V0–Vx)/V0] × 100. All measurements were performed in triplicate. Statistical analysis was conducted using t‐tests, Wilcoxon, and Mann‐Whitney tests (*p* < 0.05).

**Results:** No significant differences were observed in GBV and GBEF between groups at any time point (*p* < 0.05). However, hypothyroid dogs exhibited slower emptying postprandially compared to the fasted state, whereas healthy dogs showed a more rapid response (Table 1).

**Table:** **Table 1.** Comparison of mean volume (standard deviation) and gallbladder emptying in hypothyroid (*n* = 9) and healthy (*n* = 21) dogs.

Time after meal (mins)	Gallbladder volume (mL/kg)			Ejection fraction (%)		
	Hypothyroid	Healthy	*p*	Hypothyroid	Healthy	*p*
0	0.65 (0.34)	0.68 (0.30)	0.72^w^			
30	0.57 (0.24)	0.53 (0.25)	0.65^t^	5.70 (35.7)	22.98 (19.3)	0.42^w^
60	0.52 (0.25)	0.49 (0.25)*	0.66^w^	13.20 (39.8)	27.86 (20.2)	0.27^w^
120	0.45 (0.24)*	0.45 (0.26)*	0.86^w^	26.64 (32.0)	35.31 (32.0)	0.79^w^

*p* value obtained using^w^ the Wilcoxian test and using^t^ t‐test. *represents a difference (*p* < 0.05) in relation to time 0 in the same column.

**Conclusions and Clinical Importance:** Hypothyroid dogs have similar GBV but exhibit delayed GBEF compared to healthy dogs, suggesting an altered postprandial gallbladder response in hypothyroid dogs.

## 157 ABSTRACT GI22: Outcome of Protein‐Losing Enteropathy in Pugs in First‐Opinion Practice: 69 Cases

### 157.1 **Aarti Kathrani** ^1^; David Church^2^; David Brodbelt^2^; Dan O'Neill^2^

#### 157.1.1 ^1^Associate Professor of Gastroenterology, Internal Medicine and Nutrition, Royal Veterinary College, UK; ^2^Royal Veterinary College, UK

**Background:** Pugs with protein‐losing enteropathy (PLE) are reported to have a worse outcome versus other breeds in referral practice.

**Objectives:** To report the clinicopathologic findings, treatment, and outcomes of pugs with PLE in first‐opinion practice.

**Animals:** Sixty‐nine pugs diagnosed with PLE between 2017 to 2024 in first‐opinion practices in the United Kingdom.

**Methods:** Retrospective VetCompass study of first‐opinion practice records documenting clinicopathologic, treatment and outcome in pugs diagnosed with PLE.

**Results:** Of 69 pugs diagnosed with PLE, 38 (55%) died or were euthanized. Thirty‐two (84%) deaths were presumed due to PLE with 17 (53%) of these occurring within 3 months of diagnosis. The following were recorded at the time of death or euthanasia presumed due to PLE: lethargy and/or weakness (14), vomiting (11), diarrhea (6, 2 with melena or fresh blood), pale mucous membranes and/or anemia (7), reduced appetite (10), seizures (4), aspiration pneumonia (4), labored breathing (3), collapse (4), sepsis (1), pancytopenia (1) and foam from nose following dead on arrival (1). Dogs that died within 3 months of diagnosis versus those that were still alive for at least 2 years following diagnosis (*n* = 10; median follow‐up 35 months, range 24–62) were older at diagnosis (*P* = 0.047, mean 103.8 vs. 79.5 months) and had decreased use of chlorambucil (0/17 vs. 3/10, *P* = 0.041) and clopidogrel (1/17 vs. 5/10, *P* = 0.015) at diagnosis.

**Conclusions and Clinical Importance:** Further studies will help to define the subset of pugs with PLE that will likely benefit from chlorambucil and clopidogrel treatment to increase their long‐term prognosis.

## 158 ABSTRACT GI23: Serum Cobalamin and Methylmalonic Acid Concentrations in Emaciated Dogs With Long‐Term Inadequate Nutrition

### 158.1 Sylvia Lesnikowski^1^; Emily Patterson‐Kane^2^, PhD; Robert Reisman^3^, DVM, MS; M. Katherine Tolbert^4^, DVM, PhD, DACVIM (SAIM, SA Nutrition); Ashley Self^5^, LVT, VTS (Nutrition)

#### 158.1.1 ^1^Director of Internal Medicine and Clinical Research, ASPCA Animal Hospital; ^2^Director, Research, Strategy and Research, ASPCA; ^3^Supervisor of Forensic Sciences NYC, Forensic Sciences, ASPCA; ^4^Associate Professor, Gastrointestinal Laboratory, School of Veterinary Medicine and Biomedical Sciences, Texas A&M, TX, USA; ^5^Gastrointestinal Laboratory, School of Veterinary Medicine and Biomedical Sciences, Texas A&M, TX, USA

**Background:** Cobalamin status in emaciated dogs with long‐term inadequate nutrition (LTIN) secondary to neglect is unknown.

**Hypothesis/Objectives:** Evaluate serum cobalamin and methylmalonic acid (MMA) concentrations in emaciated dogs with LTIN.

**Animals:** Serum samples from 27 emaciated dogs with LTIN presenting for forensic evaluation and 29 control dogs with adequate body condition score.

**Methods:** Single‐center, prospective, observational, cross‐sectional study measuring serum cobalamin and MMA concentrations in emaciated dogs with LTIN, compared to control animals with adequate body condition score.

**Results:** 6/26 (23%) dogs with LTIN had hypocobalaminemia (< 251 ng/L) and 15/26 (58%) dogs were in the low‐normal range (< 400 ng/L). The 26 dogs that were emaciated had significantly lower serum B_12_ concentrations (mean 459, median 341, SD=52.2) compared to the 29 control dogs (mean 647, median 610, SD=3151.1; *p* < 0.01). Although 6 of 27 (23%) dogs with LTIN had increased serum MMA, none of the dogs with hypocobalaminemia had elevated MMA concentrations. The was no significant correlation (Spearman *r* = –0.0513, *p* = 0.79) between serum cobalamin and MMA concentrations in emaciated dogs.

**Conclusions and Clinical Importance:** Hypocobalaminemia was present in some emaciated dogs presenting with LTIN. However, none of the hypocobalaminemic dogs had a concurrent MMA elevation, suggesting that their cobalamin deficiency was not found at the cellular level. This study represents the first known report of hypocobalaminemia secondary to starvation in dogs. We recommend evaluation and cobalamin supplementation on intake to all emaciated dogs presenting with LTIN.

**Figure:**
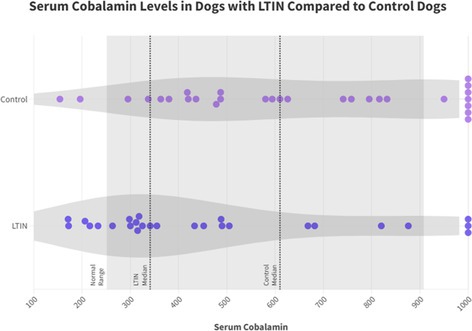
Figure 1

## 159 ABSTRACT GI24: Fecal Concentrations of Carbohydrates in Cats with Chronic Enteropathy

### 159.1 **Chih‐Chun Chen** ^1^, DVM; Katerina Moraiti^2^; Maria Giordano^3^; Amanda Blake^4^; Rachel Pilla^4^; Paula Giaretta^4^; Katherine Tolbert^4^; Jörg Steiner^4^; Panagiotis Xenoulis^2^; Paolo Crisi^3^; Andrea Boari^3^; Jan Suchodolski^4^

#### 159.1.1 ^1^Graduate Research Assistant, Gastrointestinal Laboratory, Texas A&M University, College Station, TX, USA; ^2^Small Animal Clinic, University of Thessaly; ^3^Department of Veterinary Medicine, University of Teramo; ^4^Gastrointestinal Laboratory, Texas A&M University, College Station, TX, USA

**Background:** Carbohydrates are not considered required nutrients in the diet of cats but provide nutritional value. Previous targeted and untargeted metabolomics studies found that a subset of dogs with chronic enteropathy (CE) had increased fecal concentrations of glucose and other carbohydrates associated with increased abundance of fecal microbial genes related to carbohydrate metabolism. Therefore, we suspected that carbohydrate dysmetabolism may also be present in cats with CE.

**Objective:** To describe fecal concentrations of selected carbohydrates in cats with CE and healthy control cats (HC).

**Animals:** Feces from 73 cats with CE and 52 HC.

**Methods:** Retrospective cross‐sectional study. Fecal concentrations of glucose, fructose, mannose, galactose, xylose, ribose, arabinose, and rhamnose were measured by an analytically validated GC‐MS assay. Fecal microbiota was evaluated by qPCR‐based feline dysbiosis index (DI).

**Results:** Fecal carbohydrate profiles differed significantly between HC and cats with CE on multivariant analysis (permutational multivariate analysis of variance [PERMANOVA]: *p* = 0.001). Cats with CE showed significantly decreased concentrations of some carbohydrates that are absorbed in the intestine by simple diffusion (rhamnose, xylose, and arabinose) (Mann‐Whitney U test, adjusted p value < 0.001). A subset of cats with CE (9/73; 12%) had glucose concentrations above the 95th percentile observed in HC; of these, 9/9 (100%) had an increased DI (Fisher's exact test, *P* = 0.049).

**Conclusions and Clinical Importance:** Altered fecal carbohydrate profiles were observed in a subset of cats with CE in association with intestinal dysbiosis.

## 160 ABSTRACT GI25: Duodenum Mucosa Genome‐Wide DNA Methylation Profiling in Canine Inflammatory Enteropathy

### 160.1 **Albert Jergens** ^1^, DVM, PhD, MS, BS, AGAF, DACVIM (SAIM); Dipak Sahoo^1^; Romy Heilmann^2^; Valerie Parker^3^; Adam Rudinsky^3^; Jenessa Winston^3^; Agnes Bourgoise‐Mochel^4^; Karin Allenspach^4^; Jonathan Mochel^4^; Kaylee Shaner^1^

#### 160.1.1 ^1^Professor of Internal Medicine, Iowa State University, Ames, IA, USA; ^2^University of Leipzig, Germany; ^3^The Ohio State University, Columbus, OH, USA; ^4^University of Georgia, Athens, GA, USA

**Background:** Recent studies indicate that dogs exposed to enteropathogens early in life are at increased risk for developing CIE as adults, suggesting that epigenetic mechanisms contribute to disease development.

**Objective:** To investigate DNA methylation (DNAm) in duodenal biopsies of dogs with CIE pre‐ versus post‐treatment.

**Animals:** Twenty dogs diagnosed with CIE.

**Methods:** Dogs were randomized to receive an elimination diet ± synbiotic for 6 weeks. Endoscopic duodenal biopsies were collected pre‐ and post‐treatment. An untargeted approach using whole genome bisulfite sequencing was used to examine methylation‐specific patterns of tissue DNAm across the whole genome. Total reads were mapped to a reference *Canis lupus familiaris* genome, and the methylation level was calculated for each methylation site. Enrichment of DMR‐involved genes was evaluated in KEGG pathways and the genomic locations of DMRs were annotated. The mucosal microbiota was investigated using FISH.

**Results:** Non‐CG sites (CHG, CHH) exhibited distinct relationships with methylation levels compared to CG sites in synbiotic‐ vs. diet‐treated dogs. Differentially expressed genes (DEGs, *p* < 0.05) included hyper‐methylation genes: *MAPK*, *Wnt*, *Ras*, *cGMP*‐*PKG*, and *cAMP* signaling pathways, inflammatory mediator regulation of *TRP* channels/*MAPK* signaling pathway, lysine degradation, *cAMP* and *Rap1* pathways (*CHH*). Hypo‐methylation genes: metabolic pathways, protein digestion and absorption, cell adhesion molecules, focal adhesion and *Rap1*, PI3K‐*Akt*, *TGFβ*, *cGMP*‐*PKG*, *Ras*, *FoxO*, *AGE*‐*RAGE*, *NF*‐κB, *NOD*‐like receptor and *AMPK* pathways, pathways regulating stem cell pluripotency, endocytosis and gap junctions (CG).

**Conclusions and Clinical Importance:** Genome‐wide DNAm analysis identified unique functional patterns associated with hyper‐ and hypo‐methylation and microbial imbalances in dogs with CIE.

## 161 ABSTRACT GI26: Clinical Efficacy and Safety of Fexuprazan in Canine Chronic Gastritis

### 161.1 **Joonghyun Song** ^1^, DVM, PhD; Seonggyu Jeong^2^; Daegeon Kim^3^

#### 161.1.1 ^1^Assistant Professor, Chungnam National University; ^2^Department of Veterinary Internal Medicine, College of Veterinary Medicine, Chungnam National University, Daejeon, Korea; ^3^Drug Discovery Center, Daewoong Pharmaceutical Co., Ltd., Yongin, Republic of Korea

**Background:** Fexuprazan, a novel potassium‐competitive acid blocker, may benefit patients with gastric mucosal injury.

**Objectives:** To evaluate the efficacy and safety of fexuprazan in canine chronic gastritis.

**Animals:** Twenty‐seven client‐owned dogs diagnosed with chronic gastritis.

**Methods:** A randomized active‐controlled trial. Chronic gastritis was diagnosed in 20 dogs based on a comprehensive evaluation of clinical manifestations (CM), endoscopic findings (EF), and histopathological examination (HE). Patients were orally administered one of the following treatments once daily for four weeks: low‐dose fexuprazan (group 1; 0.5 mg/kg, *n* = 5), high‐dose fexuprazan (group 2; 0.5 mg/kg; *n* = 5), famotidine (group 3; 0.5 mg/kg, *n* = 5), or esomeprazole (group 4; 0.5 mg/kg, *n* = 5).

**Results:** Both fexuprazan groups (group 1 and 2) and the groups treated with other medications (group 3 and 4) showed significant post‐treatment improvements in CM (*P* = 0.010; *P* = 0.048, respectively) and EF (*P* = 0.011; *P* = 0.019, respectively) scores. In each group, significant improvement in CM scores were observed in group 1 (*P* = 0.042), and significant improvement in the EF scores were observed in group 1 (*P* = 0.039) and group 4 (*P* = 0.038). No significant differences in post‐treatment CM (*P* = 0.080), EF (*P* = 0.515), and HE (*P* = 0.623) scores were identified between the groups. No significant adverse drug reactions were observed during the experimental period.

**Conclusions and Clinical Importance:** Fexuprazan is considered a potential alternative to other acid‐suppressing drugs and is regarded as safe for managing canine chronic gastritis.

**Figure:**
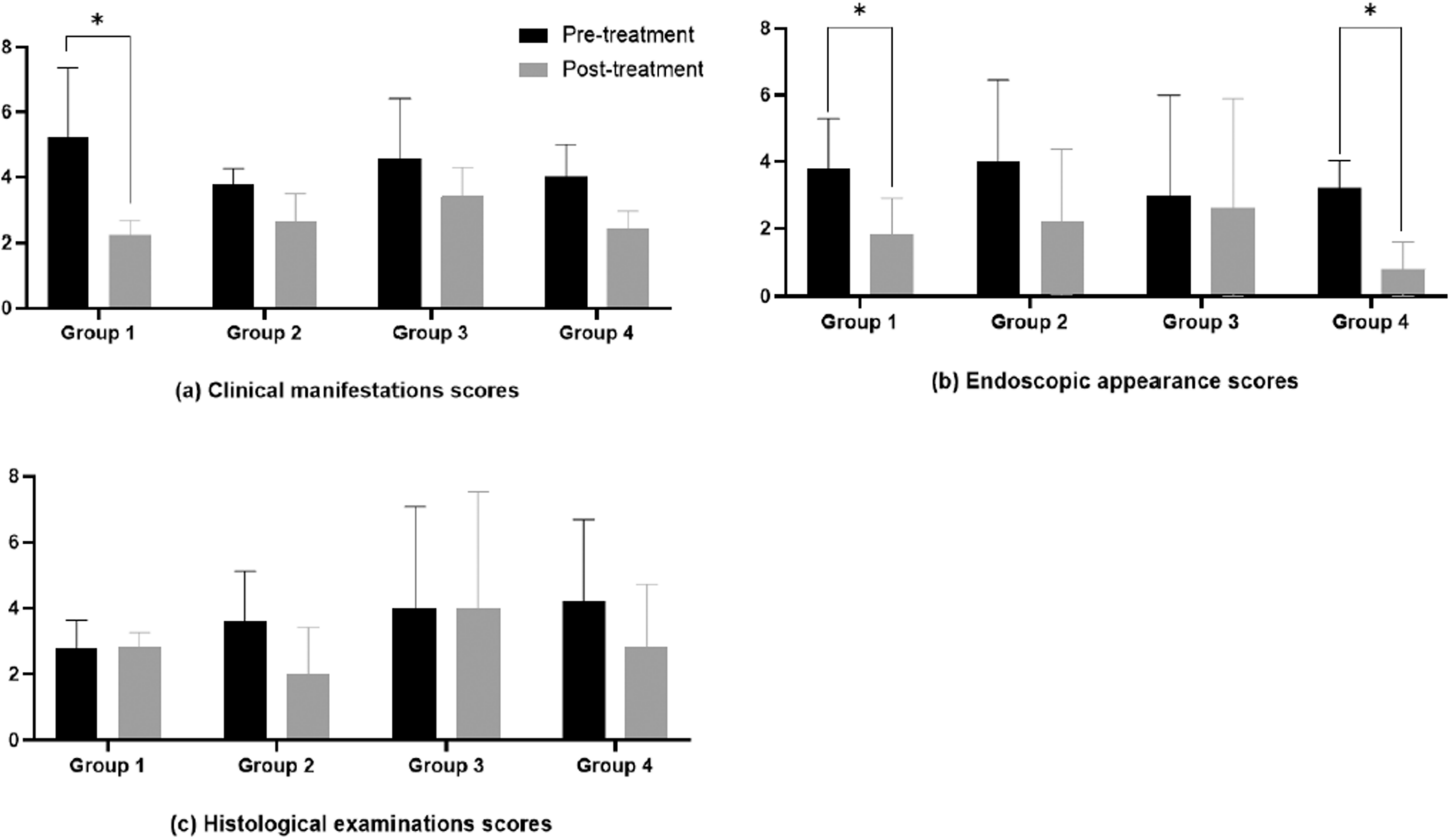
Figure 1. A comparison of clinical manifestations scores (a), endoscopic appearances scores (b), and histological examinations scores (c) between pre‐ and post‐treatment. Statistical analysis was performed using the Kruskal‐Wallis test. Significant differences are indicated with P < 0.05 (*).

## 162 ABSTRACT GI27: AI‐Assisted Meta‐Analysis on the Use of Hypoallergenic Diets for the Treatment of Canine Chronic Enteropathies

### 162.1 **Ricardo Duarte** ^1^, DVM, MSC, PHD; Ana Rita Pereira^2^, DVM, MSC; Ana Claudia Balda^1^, PhD, DVM, MS

#### 162.1.1 ^1^Faculdades Metropolitanas Unidas, São Paulo, Brazil; ^2^GastroVet Vet Med

**Background:** Canine chronic enteropathies (CCE) are inflammatory gastrointestinal diseases commonly encountered in veterinary practice, characterized by persistent vomiting, diarrhea, and weight loss. Among the subtypes, diet‐responsive enteropathy stands out, accounting for 50%–65% of cases and responding exclusively to hypoallergenic diets based on hydrolyzed or novel proteins.

**Objective:** To evaluate the efficacy of hypoallergenic diets as an initial monotherapy for treating CCE in dogs and their potential to reduce the use of immunosuppressants and antibiotics.

**Methods:** A meta‐analysis was conducted, including eight clinical studies. Statistical analysis was performed using artificial intelligence (AI) tools and further validated by an independent statistician to ensure robustness and reliability.

**Results:** The findings demonstrated that 60.8% of dogs with CCE responded positively to hypoallergenic diets, reinforcing their efficacy as a first‐line treatment. Despite limitations, such as methodological and sample heterogeneity, the data support hypoallergenic diets as safe and effective interventions, playing a crucial role in CCE management.

**Conclusion:** Hypoallergenic diets have been shown to be effective and safe for treating CCE, highlighting the importance of personalized approaches to optimize clinical outcomes and reduce costs. The use of AI in research enabled efficient analysis of large datasets, identifying relevant patterns. However, the accuracy of findings depends on the quality and structure of input data, emphasizing the need to minimize biases and inconsistencies in future studies.

## 163 ABSTRACT GI28: Postmortem Evaluation of Esophageal Diameters in Dogs and Cats: A Descriptive Study in 352 Animals

### 163.1 **Abby Goldberg** ^1^, VMD; Chick Weisse^2^, VMD, DACVS (SA); Taryn Donovan^3^, DVM, DACVP; Paul Christos^4^, DrPH, MS

#### 163.1.1 ^1^Resident Veterinarian in Internal Medicine, Schwarzman Animal Medical Center, New York, NY, USA; ^2^Senior Veterinarian, Specialist in Surgery, Interventional Radiology Service, Schwarzman Animal Medical Center, New York, NY, USA; ^3^Senior Veterinarian, Specialist in Anatomic Pathology, Anatomic Pathology, Schwarzman Animal Medicine Center, New York, NY, USA; ^4^Professor of Research in Population Health Sciences, Population Health Sciences, Weill Cornell Medical College, New York, NY, USA

**Background:** Standard therapy for esophageal strictures typically involves dilation using balloon catheters, bougies, or endoscope tips; however, there are no standard recommendations for dilation diameter. To our knowledge, there is no data in the veterinary literature describing esophageal diameters in a population of dogs and cats.

**Hypothesis/Objectives:** To describe the postmortem esophageal diameters of a population of client‐owned dogs and cats without esophageal disease.

**Animals:** 262 dog and 90 cat cadavers euthanized for reasons unrelated to this study and without any history of esophageal disease who had an autopsy performed.

**Methods:** This was a prospective study (February 2013 to September 2020). The cadaver esophagus was removed, and the widths were measured in three regions of the closed esophagus which were then converted to diameters (D) (D = C/3.14).

**Results:** Canine esophageal diameters (median 15.9–17.8 mm; range 8–31 mm) were positively correlated with weight at all 3 locations (*p* < 0.001), but cats (median 9.6–11.1 mm; range 5–17 mm) were not. Among all dog and cat breeds, there was a significant difference between proximal, mid‐ and distal esophageal diameters (*p* < 0.001), with the average esophagus increasing in diameter moving aborally.

**Conclusions and Clinical Importance:** Esophageal diameter varies considerably among both dogs and cats, with body weight, breed, and location being critical determinants. While these findings do not preclude the need for accurate measurements prior to an intervention, the data gathered guides more informed decisions and equipment needs for the management of esophageal strictures.

## 164 ABSTRACT GI29: Evaluation of Immune System Components in Dogs with Protein‐Losing Enteropathy Versus Healthy

### 164.1 **Emily Moore** ^1^, DVM; Kyan Thelen Strong^2^; Sara Jablonski^2^

#### 164.1.1 ^1^SAIM Resident, Michigan State University, East Lansing, MI, USA; ^2^Michigan State University; East Lansing, MI, USA

**Background:** Immune system abnormalities including hypogammaglobulinemia and T‐cell deficiency have been described in humans with protein‐losing enteropathy (PLE). It is unknown whether similar deficiencies exist in dogs with PLE.

**Objective:** To evaluate serum immunoglobulin (Ig) concentrations and immune cell populations in a group of dogs with PLE (secondary to chronic inflammatory enteropathy, intestinal lymphangiectasia, or both) compared to healthy controls (HC).

**Animals:** 18 dogs with PLE and 18 HC dogs.

**Methods:** Prospective study. Dogs with a history of glucocorticoid or immunosuppressive drug administration in the previous 28 days were excluded. Serum IgA, IgG, and IgM levels were measured in well‐characterized PLE dogs and compared to HC dogs. Flow cytometric analysis of T and B cells was planned but unable to be performed due to insufficient cell numbers in PLE dogs. Thus, RNA gene expression of cell surface markers in peripheral blood mononuclear cells was measured by quantitative PCR.

**Results:** Serum IgG (*P* < 0.001) and IgM (*P* = 0.002) concentrations were lower in PLE dogs compared to HC. Serum IgA concentrations were correlated with age in PLE dogs (*P* = 0.008). Additionally, PLE dogs had reduced expression of CD3e (*P* < 0.001), CD5 (*P* < 0.001), and CD8 (*P* = 0.007), while expression of CD4 and CD21 did not differ between groups.

**Conclusions and Clinical Importance:** Dogs with treatment‐naïve PLE have evidence of immunodeficiency. While it is unknown if this represents a cause or consequence of disease, this baseline state of immunodeficiency should be taken into consideration in the management of dogs with PLE.

## 165 ABSTRACT GI30: Fecal Microbiota Transplant as an Adjunctive Treatment of Canine Chronic Pruritic Dermatitis

### 165.1 **Marine Rullier** ^1^, DMV, MS; Marie‐Claude Blais^2^, DACVIM (SAIM); Marcio Costa^2^; Frédéric Sauvé^2^, ACVD; Jan Suchodolski^3^

#### 165.1.1 ^1^PhD Candidate, Dermatology Resident, Université de Montréal, Montreal, QC, Canada; ^2^University of Montreal, Montreal, QC, Canada; ^3^Texas A&M University, College Station, TX, USA

**Background:** Intestinal microbiota imbalances may contribute to the inflammatory process in canine chronic pruritic dermatitis (CPD). Fecal microbiota transplantation (FMT) has been reported to improve atopic signs in both humans and dogs by restoring gut microbiota balance.

**Objectives:** To evaluate the impact of FMT on the intestinal microbiota composition of dogs with CPD.

**Animals:** Fifteen adult dogs with CPD receiving non‐steroidal antipruritics were assigned to either the treatment (*n* = 6) or placebo (*n* = 9) group. Nine healthy dogs served as controls.

**Methods:** The treatment group received an FMT enema from a healthy donor, followed by daily oral lyophilized FMT capsules for 30 days. The placebo group received an autologous FMT enema (self‐derived feces) and placebo capsules (crushed kibbles). All dogs were fed the same diet. Clinical skin lesion scores (CADESI‐04) and microbiota composition (16S rRNA sequencing, V4 region) were assessed before treatment and at day 30 (D30).

**Results:** At D30, no statistically significant differences were observed between groups. No significant changes in microbiota richness, diversity, or composition were detected at any time point (ANOVA and PERMANOVA, *p* > 0.05).

**Conclusions:** The FMT protocol did not alter the fecal microbiota composition in CPD dogs, likely due to the absence of dysbiosis. Despite the small sample size, a trend toward pruritus improvement was noted. Larger studies in dogs with confirmed dysbiosis are necessary to assess FMT's potential for CPD management further.

## 166 ABSTRACT GI31: Description and Insights of the Feline and Canine Gut Mycobiome

### 166.1 **Elisa Scarsella** ^1^, PhD; Guillaume Jospin^2^; Daan Reyes^2^; Holly Ganz^2^

#### 166.1.1 ^1^Director, Molecular Biology, AnimalBiome, Oakland, CA, USA; ^2^AnimalBiome, Oakland, CA, USA

**Background:** The gut mycobiome plays a vital role in maintaining the health and well‐being of cats and dogs. However, little is known about the composition and diversity of the feline and canine gut mycobiomes, especially at the full‐length ITS level.

**Objectives:** This study characterized the gut mycobiome of cats and dogs using full‐length ITS sequencing of fecal samples.

**Methods:** 670 canine fecal samples (297 customer submissions with no conditions, 346 customer submissions with gut, skin or oral issues, and 27 healthy donors) and 139 feline fecal samples (57 customer submissions with no conditions, 65 customer submissions with gut, skin or oral issues, and 17 healthy donors) were sequenced for full‐length ITS.

**Results:** *Cladosporium herbarum*, *Cyphellophora reptans*, *Cyniclomyces guttulatus*, *Issatchenkia orientalis*, *Saccharomyces cerevisiae*, *Candida albicans*, *Malassezia pachydermatis*, and *Trichosporon asahii* were the most abundant fungi in dogs. In cats, the main taxa were *Cyniclomyces guttulatus*, *Saccharomyces cerevisiae*, *Cyphellophora reptans*, *Cryptococcus neoformans*, *Clitopilus baronii*, and *Sarocladium dejongiae*. We found no difference in alpha diversity between healthy and customer‐derived samples from both cats and dogs. Furthermore, a comparative analysis was performed on samples subjected to both 16S and ITS sequencing. The mycobiome's Shannon index was lower in healthy subjects and higher in those with undetermined health. The opposite trend was seen in bacterial community composition.

**Conclusions:** Although limited by a relatively small sample size, this study provides new insights into the composition and diversity of feline and canine gut mycobiomes. Further research is needed for a comprehensive understanding.

## 167 ABSTRACT HM01: Risk Factors Associated With Lipemic Plasma in a Cohort of Healthy Canine Blood Donors

### 167.1 **Corentin Treich** ^1^; Marie‐Claude Blais^2^, DVM, DACVIM (SAIM)

#### 167.1.1 ^1^Internal Medicine Resident, Université de Montréal, QC, Canada; ^2^Full Professor in Small Animal Internal Medicine, Clinical Sciences, Université de Montréal, QC, Canada

**Background:** Plasma products are widely used in veterinary medicine, particularly to treat coagulopathies. However, most transfusion guidelines advise against using lipemic plasma, leading to the loss of valuable blood products.

**Objectives:** To assess the prevalence of lipemic plasma in healthy canine blood donors, identify predisposing factors, including dietary links, and provide recommendations to reduce its occurrence.

**Animals:** A total of 296 healthy canine blood donors were enrolled in a university blood bank and participated prospectively following owner‐signed consent. Health was confirmed through physical examination and complete bloodwork including total T_4_.

**Methods:** After successful blood collection and processing, fresh frozen plasma was assessed blindly by two operators using a turbidity/lipemic index chart (0=clear to 4=opaque with severe lipemia). Plasma graded 3–4 (lipemic) was compared to grades 0–2 (non‐lipemic), and generalized linear models were used to identify risk factors for lipemic plasma.

**Results:** The prevalence of lipemic plasma in the donor cohort was 11.8%. Key associated factors included timing of the last meal (3.64±0.95 h vs. 5.94±0.63 h, *p* = 0.008), especially meals within 4 h of collection (80.56% vs. 47.69%, *p* < 0.001, **or**: 1.15), dietary fat content (46.8 g/1000 kcal±7.93 vs. 40.8 g/1000 kcal±1.09), feeding a raw diet (19.44% vs. 3.88%, *p* = 0.001, OR: 1.37), and triglyceridemia (2.26 mmol/L±0.36 vs. 0.91 mmol/L±0.05, *p* < 0.001).

**Conclusions:** To minimize blood product losses, pre‐collection guidelines could include a 4‐h fasting period, particularly for dogs on raw diets, while avoiding fatty diets.

## 168 ABSTRACT HM02: Plasma Plasminogen Activator Inhibitor (PAI‐1) Activity in Dogs With Immune‐Mediated Hemolytic Anemia

### 168.1 **Carly Iulo** ^1^; Marjory Brooks^2^; Steven Friedenberg^3^; Robert Goggs^2^; Jaspreet Kaur^2^; Dana LeVine^4^; Liming Shen^4^; Tracy Stokol^2^; Austin Viall^5^

#### 168.1.1 ^1^Veterinary Internal Medicine Resident, College of Veterinary Medicine, Auburn University, AL, USA; ^2^College of Veterinary Medicine, Cornell University, NY, USA; ^3^College of Veterinary Medicine, University of Minnesota, MN, USA; ^4^College of Veterinary Medicine, Auburn University, AL, USA; ^5^School of Veterinary Medicine, University of California–Davis, Davis, CA, USA

**Background:** Immune‐mediated hemolytic anemia (IMHA) results in high mortality rates due to fatal thromboemboli. Increased plasminogen activator inhibitor (PAI‐1) gene expression has been reported in IMHA dogs, indicating that hypofibrinolysis may contribute to thrombotic risk. Plasma PAI‐1 activity in dogs with IMHA is unknown.

**Objectives:** Develop and validate a PAI‐1 kinetic activity assay by modifying a human fluorimetric tissue plasminogen activator (tPA) activity assay and measure plasma PAI‐1 activity in dogs with IMHA and healthy controls (HC).

**Animals:** 16 dogs with non‐associative IMHA; 11 HC dogs.

**Methods:** Prospective cross‐sectional study. The PAI‐1 activity assay was configured as a human tPA activity assay modified by the addition of recombinant canine PAI‐1 to generate a PAI‐1 standard curve. Repeatability, reproducibility, recovery, and limits of blank (LoB) and of detection (LoD) were determined according to quality assessment guidelines of the American Society for Veterinary Clinical Pathology. Mann‐Whitney U tests compared median plasma PAI‐1 activity between groups.

**Results:** Assay performance was acceptable including repeatability of 15.8%, reproducibility of 7.9% and observed to expected recovery ratios of 86%–120% for all tested concentrations. LoB and LoD were 10 and 15 ng/mL. Plasma PAI‐1 activity was significantly higher in IMHA (median [range]: 48 [15–439] ng/mL) compared to HC (15 [15–15] ng/mL) dogs (*P* < 0.001).

**Conclusions and Clinical Importance:** An analytically validated PAI‐1 activity assay detected increased plasma PAI‐1 activity in dogs with IMHA compared to HCs. PAI‐1 inhibitors could be novel therapeutics to prevent fatal thrombotic events in IMHA patients.

## 169 ABSTRACT HM03: Evaluation of the Ability of *Babesia rossi* to Induce Neutrophil Extracellular Trap Formation

### 169.1 **Grace Hurley** ^1^; Liming Shen^2^; Andrew Leisewitz^2^; Dana LeVine^2^

#### 169.1.1 ^1^Veterinary Student, College of Veterinary Medicine, Auburn University, AL, USA; ^2^College of Veterinary Medicine, Auburn University, AL, USA

**Background:** *Babesia rossi* (*B. rossi*) causes inflammatory disease in dogs that models malaria, triggering hemolytic anemia and multiple organ failure. Neutrophil extracellular trap (NET) formation drives inflammation in malaria, but its role in babesiosis pathogenesis is unexplored.

**Hypotheses:** *B. rossi* will stimulate NETosis *in vivo*, evidenced by increased plasma cell‐free DNA (cfDNA) in infected dogs. Serum from *B. rossi*‐infected dogs and *B.rossi* parasite lysate will stimulate NETosis *ex vivo* in healthy canine neutrophils.

**Animals:** 9 research dogs (*B. rossi* infections); 10 blood donor dogs (neutrophil isolation).

**Methods:** Plasma was collected from dogs pre‐ and post‐*B. rossi* infection. Plasma cfDNA was quantified with picogreen. Using an *ex vivo* model, isolated donor neutrophils were incubated with pre‐ or post‐infection serum and phorbol‐12‐myristate‐13‐acetate (PMA; NET inducer) or media (unstimulated), stained with dyes labeling extracellular DNA/NETs (SYTOX orange) and intracellular DNA (SYTO green). Percent NET‐forming neutrophils was determined using fluorescence confocal microscopy; a two‐way ANOVA compared volume/treatment effects. Isolated neutrophils were incubated with *B. rossi* lysate or RBC lysate control, fixed, and stained with citrullinated histone antibody and DNA dye for NET identification.

**Results:** Plasma cfDNA was higher post‐infection than pre‐infection (*p* = 0.0312; Wilcoxon signed rank test) (Figure 1). Pre‐ and post‐infection serum did not impact NETosis in unstimulated neutrophils, but unexpectedly inhibited PMA‐induced NETosis (treatment effect; *p* < 0.0001). *B. rossi* parasite lysate induced NETosis.

**Figure:**
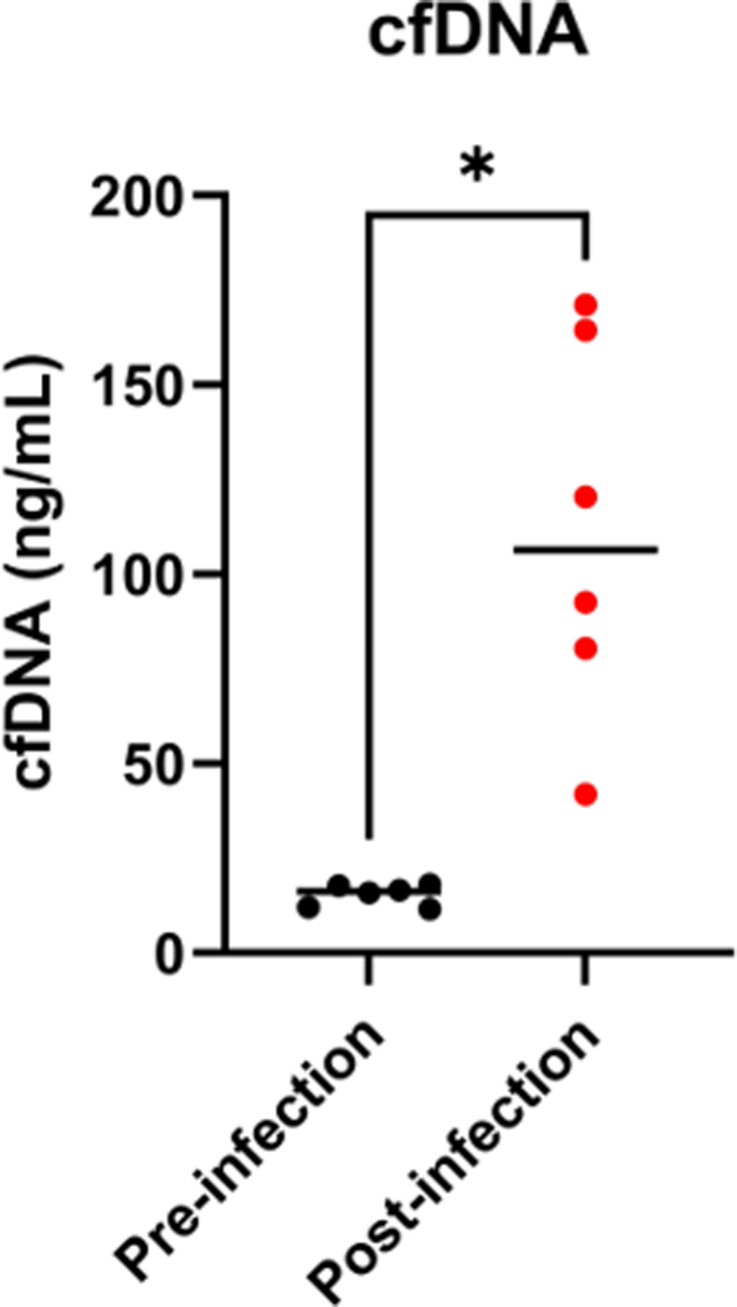
**Figure 1. Plasma cfDNA, a NET marker, was increased in *B. rossi*‐infected dogs**. cfDNA extracted from plasma of dogs infected with *B rossi* was higher (median [range]: 106.5 [41.9‐171.1] ng/mL) than pre‐infection (16.3 [11.6‐18.1] ng/mL) plasma cfDNA (*p* = 0.0312), suggesting *in vivo* NETosis in infected dogs. Lines represent medians.

**Conclusions/Clinical Importance:** *B. rossi* parasite‐induced NETosis and increased plasma cfDNA in infected dogs suggest NETosis contributes to *B. rossi* pathogenesis. Therapeutic regulation of NETosis may improve babesiosis outcomes.

## 170 ABSTRACT HM04: The Use of Cold‐Stored Platelets to Provide Hemostasis in Dogs With Thrombocytopenia

### 170.1 **Jillian Haines** ^1^; Shannon Monaco^2^, DVM; Sarah Guess^2^; Jane Wardrop^2^

#### 170.1.1 ^1^Associate Professor, Washington State University, WA, USA; ^2^Washington State University, WA, USA

**Background:** Dogs with thrombocytopenia are at risk of life‐threatening spontaneous or procedure‐related bleeding. Cold‐stored platelet (CSP) transfusions could reduce this risk.

**Hypothesis/Objectives:** To assess the ability of transfused CSP, stored at 4°C for up to 21 days, to provide hemostasis in dogs with thrombocytopenia using a modified DOGiBAT scoring system, clinical observation, and platelet counts.

**Animals:** Twenty‐four hospitalized thrombocytopenic dogs representing 27 individual transfusion events.

**Methods:** Retrospective study of thrombocytopenic dogs receiving CSP transfusion to treat clinical bleeding or to prevent bleeding secondary to invasive procedures. Information regarding CSP units, platelet counts, and DOGiBAT scores at baseline and then 1 and 4 h post CSP transfusion was collected. Descriptive statistics were performed and Wilcoxon signed‐rank test used for variable comparison.

**Results:** Dogs had a baseline median platelet count of 17 ×10^3^/μL. The reasons for CSP transfusion included surgery (8), invasive procedure (9), or bleeding (10). Dogs received 1 unit/10 kg of body weight of CSP. DOGiBAT scores were significantly lower only at 4 h post CSP transfusion (*p* < 0.05). There was no statistically significant difference in platelet counts. Clinical improvement in bleeding or a lack of noted bleeding following invasive interventions was seen in 19/27 transfusion events. Two dogs developed bleeding complications following splenic fine needle aspiration. Nineteen dogs survived to discharge. There were no transfusion reactions seen.

**Conclusions and Clinical Importance:** Transfusion with CSP is safe in thrombocytopenic dogs and provides short‐term hemostasis allowing for diagnostic and treatment interventions.

## 171 ABSTRACT HM05: Estimation of Red Blood Cell Indices in Dogs and Cats Based on Artificial Intelligence

### 171.1 **Archana Machireddy** ^1^; Liam Cattell^2^; Thanh Le^2^; Bianca Murphy^2^; Kaori Davis^2^

#### 171.1.1 ^1^Computer Vision Scientist, Moichor Inc.; ^2^Moichor Inc.

**Background:** Hematocrit (HCT) and red blood cell (RBC) count are crucial indices that assist in the diagnosis and monitoring of hematological disorders such as anemia and polycythemia. Rapid and accurate assessment of these parameters is essential.

**Hypothesis/Objectives:** The aim of this study is to estimate HCT and RBC count from microscopic blood smear images. It is hypothesized that there is a significant agreement between these indices derived from the images and from the flow cytometry‐based hematology analyzers.

**Animals:** Microscopic blood smear images from 986 dogs and 1522 cats.

**Methods:** Correlational study. Features of a blood smear are used to train a neural network to predict hematocrit and RBC count. The validity and accuracy of the proposed method are assessed by evaluating its correlation with the results from the flow cytometry‐based hematology analyzer (ADVIA® 2120 Hematology System, Siemens Healthcare Diagnostics, Tarrytown, NY, USA).

**Results:** There is a significant correlation between image‐ and ADVIA‐derived hematocrit estimate in dogs (*p* < 0.0001, *r* = 0.71) and cats (*p* < 0.0001, *r* = 0.73). Similarly, a strong positive correlation is observed between image‐ and ADVIA‐derived RBC count estimate in dogs (*p* < 0.0001, *r* = 0.71) and cats (*p* < 0.0001, *r* = 0.66).

**Conclusions and Clinical Importance:** The image‐based indices are in strong agreement with those from flow cytometry analyzers. Image‐based analysis of hematological indices can help clinicians evaluate for RBC disorders rapidly and accurately even in the absence of conventional hematology analyzers.

## 172 ABSTRACT HM06: Evaluation of Cytokine Expression for Predicting Outcome and Adverse Effects in Canine Immune‐Mediated Hemolytic Anemia

### 172.1 **James Swann** ^1^; Gina Parkes^2^; Anna Riddle^2^; Sivert Nerhagen^2^; Balazs Szladovits^2^; Barbara Glanemann^2^

#### 172.1.1 ^1^Damon Runyon Cancer Research Fellow, Columbia University, NY, USA; ^2^Royal Veterinary College, London, UK

**Background:** Immune‐mediated hemolytic anemia (IMHA) is caused by autoimmune destruction of erythrocytes, resulting in severe morbidity. Most dogs are treated with corticosteroids, but few clinical tools exist to predict which dogs will respond to treatment or experience treatment‐related adverse effects.

**Hypothesis/Objectives:** To evaluate novel biomarkers of treatment response and corticosteroid adverse effects in dogs with IMHA.

**Animals:** Thirty‐one client‐owned dogs with nonassociative IMHA, evaluated at diagnosis, 5 days, and 2 weeks after onset of treatment with prednisolone and azathioprine.

**Methods:** Residual blood samples were stimulated with phorbol 12‐myristate 13‐acetate and ionomycin before total RNA was extracted for quantitative reverse transcription (qRT)‐PCR reactions to measure expression of the cytokine genes Il2, Il4, Il10, Il17, and Ifng. Linear regression models were created to evaluate associations between gene expression and clinical outcome variables, including quality‐of‐life parameters from a validated questionnaire and direct measurement of water intake, blood pressure, and 6‐min walk test.

**Results:** All cytokine genes were detectable in stimulated blood at diagnosis, with Il‐10 expression increasing significantly after onset of treatment (*p* = 0.03), whereas Il‐17 decreased (*p* = 0.049). However, expression of Il‐10 or Il‐17 was not associated with packed cell volume at 14 days after diagnosis or duration of hospitalization. Higher Il‐2 expression after starting treatment was a significant predictor of greater water intake measured 2 weeks after diagnosis (*p* = 0.0045), but no other associations were detected between gene expression and quality‐of‐life parameters.

**Conclusions and Clinical Importance:** Expression of cytokine genes, at diagnosis or upon treatment, could represent helpful biomarkers for corticosteroid‐related adverse effects.

## 173 ABSTRACT HM07: Identification of a Novel Common Red Blood Cell Antigen Lacking in a Boykin Spaniel

### 173.1 **Marie‐Claude Blais** ^1^; Céline Athes^2^; Jon Bode^2^; Isabelle Chamberland^3^; Liming Shen^2^; Dana LeVine^4^, DVM, DACVIM (SAIM)

#### 173.1.1 ^1^Full Professor in Small Animal Internal Medicine, Faculté de Médecine Vétérinaire, University of Montreal, QC, Canada; ^2^Department of Clinical Sciences, College of Veterinary Medicine, Auburn University, AL, USA; ^3^Blood Bank Technician, Department of Clinical Sciences, Faculté de Médecine Vétérinaire, Université de Montréal, QC, Canada; ^4^Professor in SAIM, Department of Clinical Sciences, College of Veterinary Medicine, Auburn University, AL, USA

**Background:** Canine blood groups were identified through transfusion‐induced alloantibodies. High‐frequency RBC antigens pose significant clinical challenges, as rare negative patients risk sensitization, making finding compatible blood thereafter difficult.

**Hypothesis:** Suspect identification of a new canine RBC antigen due to alloantibody development in a transfusion‐sensitized Boykin spaniel.

**Animals:** Ten Boykin spaniels; four Dal dogs; 34 canine blood donors.

**Methods:** Serologic tests, including blood typing, crossmatching, agglutination titers and direct Coombs test were performed by standard tube methods and gel column technology.

**Results:** A DEA 1+ Boykin spaniel with Coombs‐negative nonregenerative anemia was first transfused with DEA 1‐packed RBCs (pRBCs). She received a second and third DEA 1+ crossmatch‐compatible pRBC transfusion, both uneventfully, 2 weeks and 11 months later. Nine days later, all major crossmatches with 47 dogs were strongly incompatible (graded 4+; auto‐controls negative), including 9 Boykin spaniels (one half‐sibling), 4 Dal dogs, and 34 blood donors (22 DEA 1+; 12 DEA 1−). The alloantibodies were identified as immunoglobulin G, with agglutination titers of 1:128 on neutral gel but exceeding 1:8,192 on antiglobulin‐enhanced gel. Notably, three dogs exhibited weaker incompatibility (graded weak to 2+; titer < 1:16). The patient was blood typed as DEA 1, 4, 5, 7 and Dal positive. Further typing and crossmatching revealed no clear association with known blood types.

**Conclusions:** A new, presumably common RBC antigen was identified in a Boykin spaniel with an acquired alloantibody likely capable of hemolytic transfusion reactions. Rare weak reactions suggest some antigenic polymorphism. Antiglobulin‐enhanced crossmatches significantly improved sensitivity.

## 174 ABSTRACT HM08: Postoperative Thrombocytosis and Hypercoagulability Based on Thromboelastography Over 28 Days After Splenectomy

### 174.1 **Hyunjin Kim** ^1^, DVM; Joonghyun Song^2^

#### 174.1.1 ^1^Veterinary Student, Chungnam National University, Daejeon, South Korea; ^2^Chungnam National University, Daejeon, South Korea

**Background:** Thrombocytosis and thromboelastographic (TEG) changes indicative of hypercoagulability are commonly observed during the first two weeks post‐splenectomy in dogs. In humans, thrombocytosis typically peaks 7 to 20 days post‐splenectomy and returns to normal values within weeks to months. The timeline for these changes remains unknown in dogs

**Hypothesis/Objectives:** We hypothesize that thrombocytosis and hypercoagulability would resolve by 28 days post‐splenectomy.

**Animals:** Five client‐owned dogs were included in the study, comprising four diagnosed with non‐neoplastic splenic nodules and one with hemangiosarcoma. All dogs underwent splenectomy.

**Methods:** In this prospective study, blood samples were collected on the day of surgery (day 0) and on days 2, 7, 14 and 28 post‐splenectomy. TEG analysis and platelet counts were performed on each of these days.

**Results:** Platelet counts were significantly elevated on days 7, 14 and 28 compared to day 0 (*P* < 0.05), with no significant difference between days 14 and 28 (*P* = 0.54). Both MA and G values were significantly increased on days 7 and 14 compared to day 0 (*P* < 0.05) and significantly decreased on day 28 compared to day 14 (*P* = 0.01).

**Conclusions and Clinical Importance:** Thrombocytosis and hypercoagulability persist up to 28 days post‐splenectomy in most dogs. By day 28, although thrombocytosis remains, the resolution of certain clot strength parameters suggests a gradual recovery from hypercoagulability, emphasizing the need for further long‐term studies.

**Figure:**
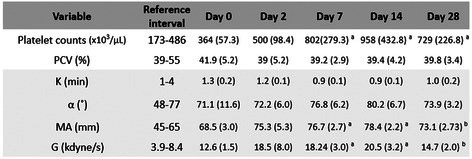
**Table 1. Mean (SD) platelet counts, PCV and TEG values on the day of surgery and 2, 7, 14 and 28 days after splenectomy**. ^a^The mean value at this assessment point differs significantly (*P* < 0.05) from the mean for the same variable on day 0. ^b^The mean value at this assessment point differs significantly (*P* < 0.05) from the mean for the same variable on day 14.

**Figure:**
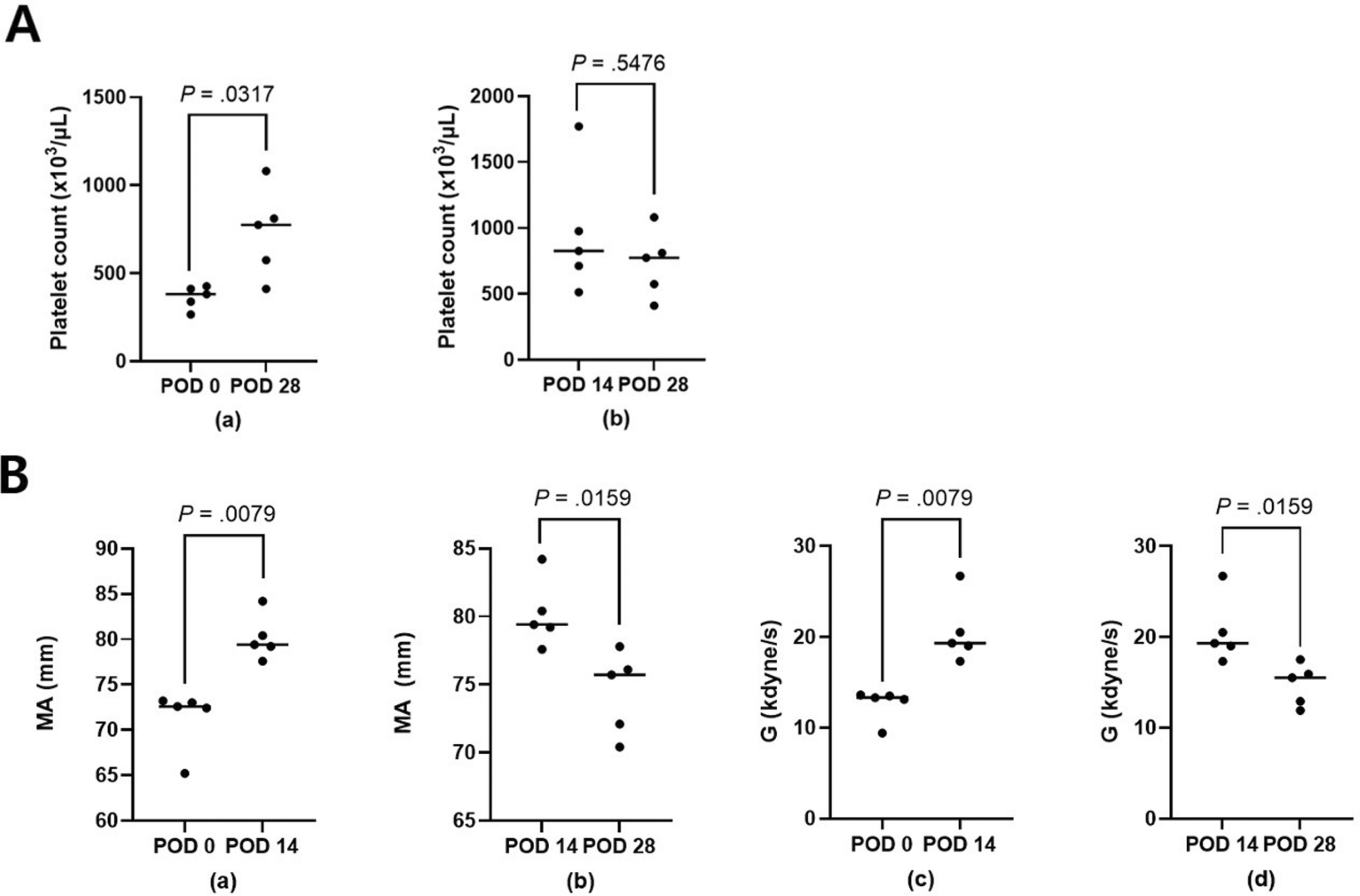
**Figure 1. Platelet counts, MA and G values over 28 days following splenectomy. Data are presented as medians with scatter plots. Statistical analyses were performed using the Mann‐Witney U test, with significant differences indicated at P < 0.05**. **A** Platelet counts were compared between POD 0 and POD 14 and POD 28. **B** MA and G values were compared between POD 0 and POD 14 and POD 28.

## 175 ABSTRACT HM09: Platelet‐Function Analysis Using the PFA‐200 in Geriatric Dogs

### 175.1 **Jaewoo Park** ^1^, MS; Chul Park^2^, PhD

#### 175.1.1 ^1^Veterinarian Internal Medicine, Jeonbuk National University, Jeonbuk, South Korea; ^2^Professor, Veterinary Internal Medicine, Jeonbuk National University, Jeonbuk, South Korea

**Background:** Platelets are essential for primary hemostasis, and their function can be influenced by various factors, including aging. While human studies often show increased platelet activation with age, leading to a prothrombotic state, the effects of aging on platelet function in dogs remain less clear.

**Objectives:** This study aims to compare and evaluate age‐related changes in platelet function in dogs using the Platelet Function Analyzer‐200 (PFA‐200).

**Animals:** Eighty healthy dogs weighing less than 15 kg were divided into a geriatric group (≥ 10 years old, *n* = 48) and a control group (< 10 years old, *n* = 32).

**Methods:** Platelet function was assessed using collagen/adenosine diphosphate (COL/ADP) and collagen/epinephrine (COL/EPI) cartridges. Platelet counts were also measured.

**Results:** Compared to the control group, geriatric dogs exhibited significantly prolonged closure times for both COL/ADP and COL/EPI, with p values of 0.002 and 0.003, respectively. Platelet counts were also significantly higher in the geriatric group than in controls. Pearson correlation analysis showed significant positive correlations between age and closure times for COL/ADP and COL/EPI, also platelet count. Closure times did not differ significantly based on weight, sex, or breed.

**Conclusions:** Our study suggests that as dogs age, their platelet function tends to decline, as supported by prolonged closure times and increased platelet counts. Interestingly, these results differ from human studies, where aging usually makes platelets more reactive. Understanding these species‐specific variations is crucial for improving clinical care in geriatric canine patients.

**Figure:**
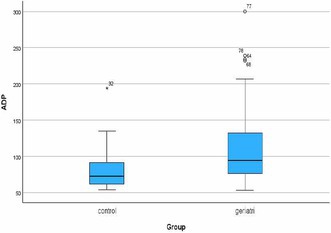
Figure 1. Box plot of COL/ADP closure time for control and geriatric groups

**Figure:**
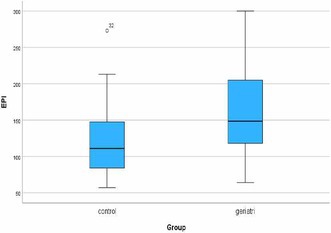
Figure 2. Box plot of COL/ADP closure time for control and geriatric groups

**Figure:**
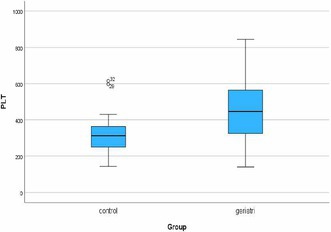
Figure 3. Box plot of PLT for control and geriatric groups

**Figure:**
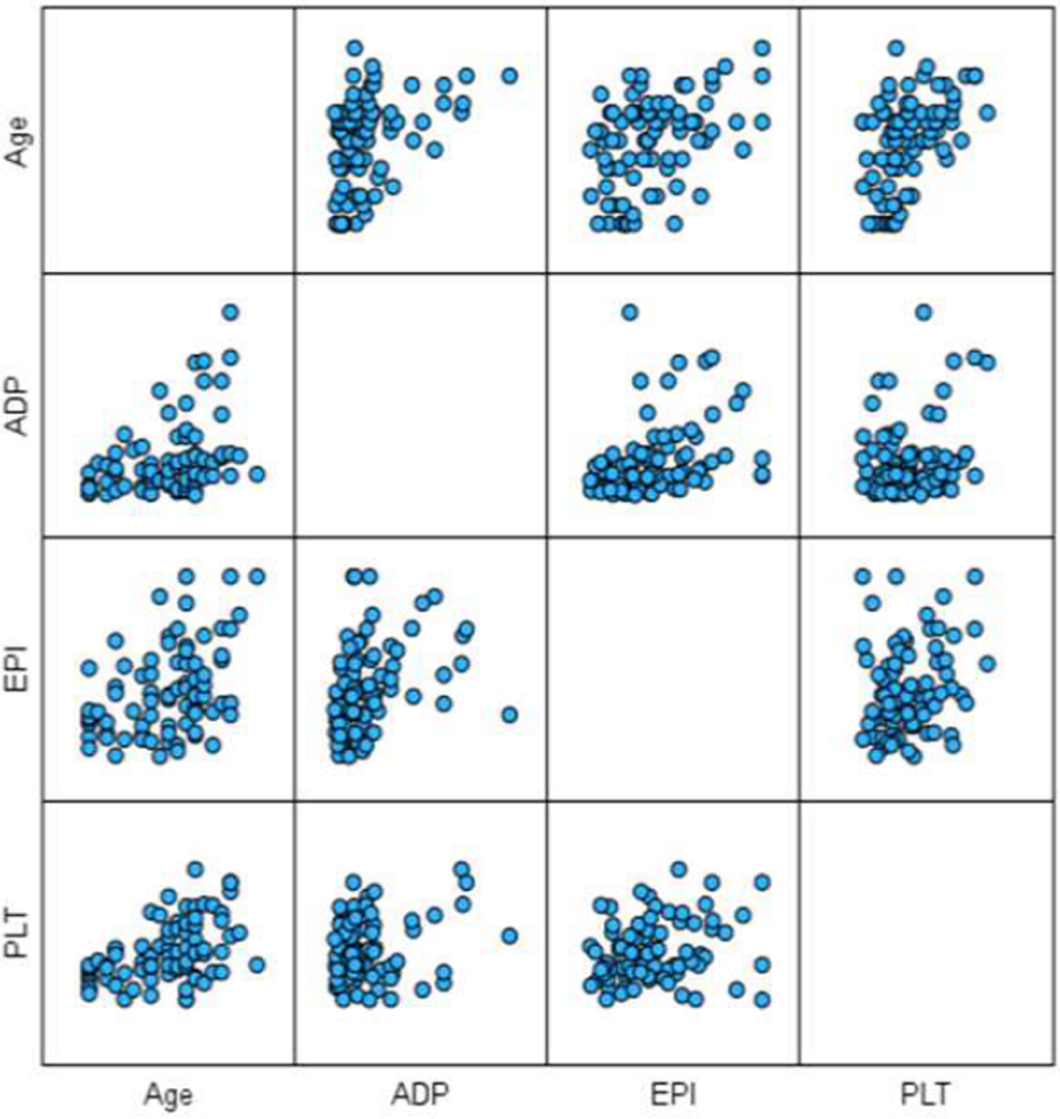
Figure 4. Scatter plot of the correlation between age, ADP, EPI and PLT

**Figure:**
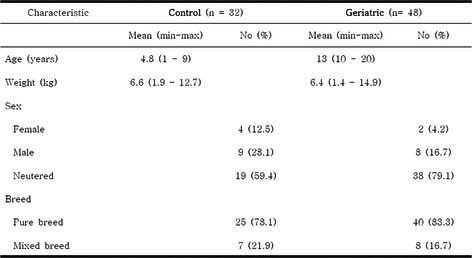
Table 1. Signalment date of control and geriatric groups

**Figure:**
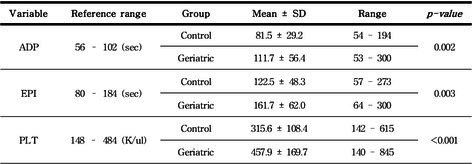
**Table 2. Result of ADP, EPI and PLT of control and geriatric groups**. ADP, closure time of COL/ADP; EPI, closure time of COL/EPI; PLT, platelet count; SD, standard deviation

## 176 ABSTRACT HM10: Indications for Outpatient Packed Red Blood Cell Transfusion in Dogs: 43 Cases (2014–2024)

### 176.1 **Clare Timothy** ^1^, BVSc, DACVECC; Elizabeth Rozanski^2^, DVM, DACVIM, DACVECC; Jennifer Simmons^2^; Natalie Lovelace^2^, DVM

#### 176.1.1 ^1^Clinical Assistant Professor, Tufts University, Medford, MA, USA; ^2^Tufts University, Medford, MA, USA

**Background:** Packed red blood cell (pRBC) transfusions are provided to increase hematocrit in anemic dogs. Anemia may develop from blood loss, hemolysis, bone marrow failure or a combination of causes. In people, outpatient transfusions are provided to stable patients, but the role of outpatient transfusion in dogs is unknown. Due to the scarcity and cost of blood products, appropriate use of blood products is warranted.

**Hypothesis/Objectives:** To retrospectively describe the indications and 30‐day outcome following outpatient transfusion administered to dogs.

**Animals:** 43 dogs receiving pRBC transfusion as outpatients.

**Methods:** The electronic medical records were searched for dogs that received pRBC transfusions and were discharged within 8 h. Dogs that were admitted or died/euthanized before discharge were excluded. The indications were classified as traumatic or surgical blood loss, immune‐mediated disease, blood loss associated with neoplasia, chronic renal failure and unclassified anemias.

**Results:** Indications for transfusion included neoplasia (*n* = 12), immune‐mediated disease (*n* = 12), acute blood loss (*n* = 10), unclassified anemia (*n* = 7), and chronic kidney disease (*n* = 2). Four dogs had transfusion reactions, with 3 febrile/non‐hemolytic and 1 hemolytic, but all were discharged. At 30 days, 19 dogs were dead, 14 dogs were alive and 10 were lost to follow‐up.

**Conclusions and Clinical Importance:** Outpatient transfusions may be performed successfully and be considered in certain cases. Access to care may be improved by limiting costs associated with transfusion therapy.

## 177 ABSTRACT HM12: Comparing Anti‐Globulin‐Enhanced Major Crossmatch Test Methods in Cats

### 177.1 **Allison Collier** ^1^, DVM, DVSc, DACVIM (SAIM); Sebastien Sanz^2^, DVM; Insung Chun^3^, DVM; Shauna Blois^4^, DVM, DVSc, DACVIM (SAIM)

#### 177.1.1 ^1^Assistant Professor, University of Guelph, Guelph, ON, Canada; ^2^Resident, Clinical Studies, University of Guelph, Guelph, ON, Canada; ^3^Intern, Health Sciences Centre, University of Guelph, Guelph, ON, Canada; ^4^Professor, Clinical Studies, University of Guelph, Guelph, ON, Canada

**Background:** It is recommended that all cats, regardless of transfusion history, undergo major crossmatch (CM) prior to blood product administration to decrease the likelihood of immunologic transfusion reactions. Crossmatching can be time consuming and result availability can be delayed, especially when performed at external laboratories. Previous studies of point‐of‐care CM tests have shown variable agreement with the standard tube‐based assay performed at reference laboratories. Accurate CM tests that are accessible in a cost‐effective and rapid manner would increase feasibility of performing CM prior to transfusions in cats.

**Objective:** To assess agreement between two antiglobulin‐enhanced major crossmatch test methods, a laboratory‐based tube (LAB‐CM) and a point‐of‐care gel column (GEL‐CM) method, in cats in need of blood transfusion.

**Methods:** Anemic cats considered likely to need a blood transfusion were prospectively recruited and crossmatched against donors of the same AB blood type. A major CM was performed simultaneously using two antiglobulin‐enhanced **Methods:** a standard tube assay performed at a reference laboratory (LAB‐CM) and a point‐of‐care gel test (GEL‐CM) performed in the clinic.

**Results:** Major crossmatch was performed on 84 unique donor‐recipient pairings, using both methods (see Table 1). Kappa agreement between the LAB‐CM and GEL‐CM was 0.675 (95% confidence interval: 0.468‐0.882).

**Conclusions and Clinical Importance:** The antiglobulin‐enhanced, point‐of‐care gel major CM test had good agreement with the antiglobulin‐enhanced tube CM performed at a reference laboratory. Additional cats will be recruited increase the power of the study.

**Table:** **Table 1.** Agreement between major antiglobulin‐enhanced crossmatch tests in cats using a laboratory tube (LAB‐CM) and a get tube (GEL‐CM) method.

	GEL‐CM incompatible	GEL‐CM compatible
LAB‐CM incompatible	11	6
LAB‐CM compatible	2	65

## 178 ABSTRACT HP01: Do Serum Proinflammatory and Procoagulant Biomarkers in Dogs With Gallbladder Mucoceles Predict Disease Progression?

### 178.1 **Kayla Prentice** ^1^; Cynthia Webster^2^

#### 178.1.1 ^1^Resident in Small Animal Internal Medicine, Tufts University, MA, USA; ^2^Professor, Clinical Science, Cummings School of Veterinary Medicine, Tufts University, MA, USA

**Background:** Gallbladder mucoceles (GBM) are a common biliary disease in dogs. Medical management can be marred by acute decompensation necessitating a high mortality cholecystectomy. A noninvasive biomarker of GBM progression to predict the need for elective cholecystectomy could decrease morbidity/mortality.

**Hypothesis/Objective:** Acute decompensation in dogs with GBM is associated with a proinflammatory and procoagulant state.

**Animals:** Prospectively enrolled 20 clinically asymptomatic dogs with GBM managed medically and 18 symptomatic dogs needing cholecystectomy at an academic hospital.

**Methods:** Inclusion criteria included ultrasonographic diagnosis of GBM, and a biochemical panel within 24 h. Exclusion criteria included medications that affect coagulation or inflammation, greyhounds, and concurrent inflammatory conditions. Blood was obtained for CBC, c‐reactive protein (CRP), fibrinogen, D‐dimers, and thromboelastography (TEG). Groups were compared with Fisher's exact test, with *P* < 0.05 considered significant.

**Results:** Surgically and medically managed dogs were similar in age, weight, and sex distribution. Surgically managed dogs were statistically more likely to have increased CRP (17/18, 94%), neutrophil counts (10/18, 55%) and fibrinogen (9/18, 50%) than medically managed dogs 0/20 (0%), 0/20 (0%) and 4/20 (20%), respectively. Similar proportions of medically (16/20, 80%) and surgically (17/18, 94%) managed dogs had high TEG G values, although statistically more surgically (9/19, 50%) than medically (3/20, 30%) managed dogs had increases in D‐dimers.

**Conclusions/Clinical Importance:** These results suggest that longitudinal studies of increases in serum CRP, fibrinogen and neutrophil counts should be done to determine if they might be early biomarkers of progressive disease in dogs with GBM, warranting consideration of an elective cholecystectomy.

## 179 ABSTRACT HP02: Safety of Chronic Copper‐Restricted Diet (0.11 mg/100 kcal) in Dogs With Congenital Portosystemic‐Vascular Anomalies

### 179.1 **Sharon Center** ^1^; John Randolph^2^, BS, DVM, DACVIM (SAIM); James Flanders^2^, BS, DVM, DACVS; Jay Harvey^2^, BS, DVM, DACVS; Karen Warner^3^, LVT, BS; Andrew Miller^4^, BS, DVM, PhD, DACVP (Anatomic)

#### 179.1.1 ^1^Emeritus James Law Professor of Internal Medicine, Cornell University, NY, USA; ^2^Emeritus Professor, Clinical Sciences, College of Veterinary Medicine, Cornell University, NY, USA; ^3^Research Support Specialist, Clinical Sciences, College of Veterinary Medicine, Cornell University, NY, USA; ^4^Associate Professor, Population Medicine and Diagnostic Sciences, College of Veterinary Medicine, Cornell University, NY, USA

**Background:** Canine risk for liver‐copper (Cu) accumulation from commercial diets supplemented with premix‐micronutrients remains controversial. There is no maximum Cu limit for dogs nor informative Cu labelling requirements. Commercial dog foods (2017–2021 per AAFCO) averaged 20–30 mg Cu/kg dry matter (DM, ≥ 0.5–0.8 mg Cu/100 kcal assuming 3,200–3,600 kcal/kg DM), exceeding minimal allowances of 0.15–0.18mg Cu/100 kcal by 3‐ to 5‐fold. Copper‐restricted (0.10–0.12 mg Cu/100 kcal) protein‐modified prescription liver diets dominate medical management of portosystemic‐vascular anomaly (PSVA) dogs if surgical intervention is not possible.

**Objective:** As portosystemic‐shunting does not alter oral Cu distribution or organ Cu accumulation, we sought to validate safety of chronic feeding Cu‐restricted diets to medically managed PSVA dogs stratified by dietary Cu intake (Cu restricted vs Cu replete [≥ 0.40mg Cu/100kcal]) and against surgically managed dogs.

**Methods:** 377 lifetime‐surveilled extrahepatic‐PSVA dogs (1980–2015) were categorized by ligation status (complete or partial ligation, or medical management stratified by dietary Cu intake; Table. Details evaluated included: age (diagnosis, death), physical/clinical/hematologic features, death cause, and survival duration/curves.

**Table:** **Table**.

Treatment groups	*n*	kg	Death age	PSVA‐related death
Complete ligation	117	5.8 (5.9–8.7)^c,b,h,i^	12.5 (11.0–12.3^k1^	16/117; 14.0%^a,c,e,j^
Partial ligation	74	4.4 (4.5–7.0)^c^	10.5 (8.3–10.2)^a,d,k2^	19/74; 26%^a,e^
Medical management	186	4.2 (4.8–6.1)^a,h^	9.7 (8.4–9.5)^a,k2^	49/186; 26%^e^
0.11 Cu mg/100 kcal	136	4.0 (4.5–5.8)^b,i^	9.7 (8.3–9.5)^k2^	28/136; 21%^g^
≥ 0.4 Cu mg/100 kcal	50	5.2 (4.8–7.3)	7.2 (6.2–8.8)^k2,d^	21/50; 42%^g,j^

**Statistics:** Wilcoxon rank sum test, 2‐by‐2 tables (Fisher's exact test), and Kaplan Meier (Gehan‐Wilcoxon, Log‐rank test) interrogated differences with 2‐sided *P* < 0.05.

**Results:** No coat, neuromuscular, orthopedic, nor hematologic (1‐yr sequential CBCs, *n* = 98 Cu‐restricted dogs) implicated Cu insufficiency. Complete ligation achieved significantly longer survival than partial‐ligation or medically managed dogs. However, survival of medically managed Cu‐restricted dogs did not differ from partially ligated dogs.

**Conclusion:** Findings validate lifetime safety of 0.10–0.12mg Cu/100 kcal in E‐PSVA dogs. While best survival is achieved with complete shunt attenuation, partial ligation and medical management with a Cu‐restricted diet are reasonable alternatives.

**Figure:**
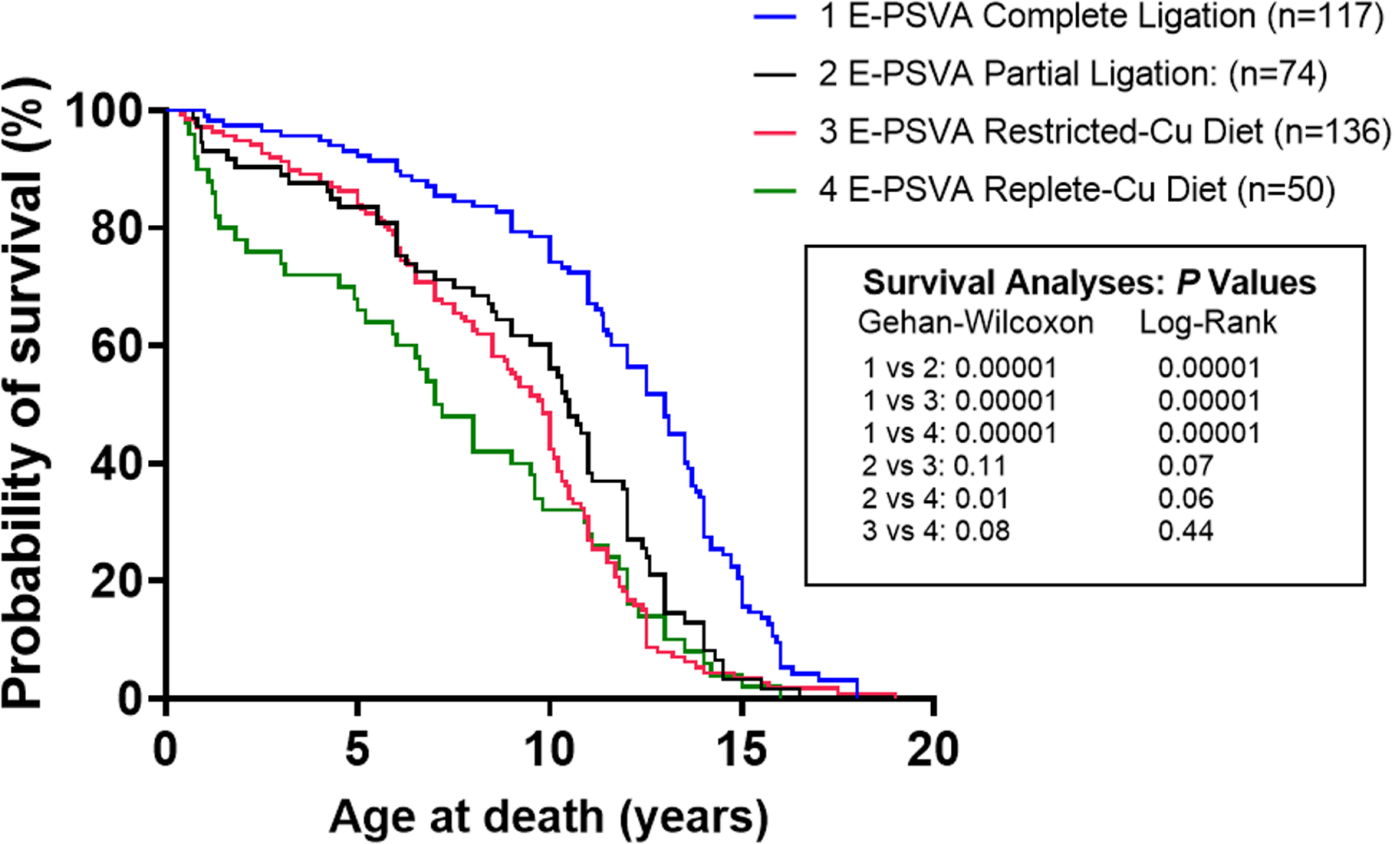
Image

## 180 ABSTRACT HP03: Lower Risk for Liver Copper Accumulation in Dogs Fed ≤ 0.24 mg Copper/100 kcal

### 180.1 **Sharon Center** ^1^; Philip VanVranken^2^; Raymond Glahn^3^, BS, PhD; Jason Wiesinger^4^, BS; Ben Dowdy^5^, BS; John Randolph^6^, BS, DVM, DACVIM (SAIM); Andrew Miller^7^, DVM, PhD, DACVP (Anatomic)

#### 180.1.1 ^1^Emeritus James Law Professor of Internal Medicine, Cornell University, NY, USA; ^2^Veterinarian Associate and Owner, Voyager Pet Foods, Dickman Road Veterinary Clinic; Owner, Voyager Dog Foods; ^3^Research Leader, College of Agriculture and Life Sciences, Robert Holley Center for Agriculture and Health, Agriculture Research Service, USDA; ^4^Research Associate, Robert Holley Center for Agriculture and Health, Agriculture Research Service, USDA; ^5^Director of Operations Voyager Dog Food Company and Brands, Voyager Dog Food Company; ^6^Emeritus Professor, Clinical Sciences, College of Veterinary Medicine, Cornell University, NY, USA; ^7^Associate Professor, Population Medicine and Diagnostic Sciences, College of Veterinary Medicine, Cornell University, NY, USA

**Background:** Canine risk for liver‐copper (Cu) accumulation from commercial diets overformulated with micronutrient premixes, remains controversial. Regulatory agencies express concern that specialty center studies may not reflect pet dog demographics.

**Objective:** Investigate (1) liver‐Cu concentration in end‐of‐life liver samples from humanely euthanized dogs in a primary care hospital and (2) relationship between liver‐Cu and dietary‐Cu intake.

**Methods:** Liver samples from 104 dogs euthanized for geriatric health concerns (owner consent) in a large primary care hospital. Medical records, dietary history, and regional water analyses were acquired. Dietary Cu (mg/100 kcal) was derived from manufacturers or validated inductively coupled‐plasma‐atomic‐emission spectrometry (ICP‐AES) in a USDA micronutrient laboratory. Liver samples underwent histologic evaluation (H&E, Masson's trichrome, rhodanine), rhodanine Cu scoring, and Cu quantification (μg/g dry weight liver [dwl]). Nonparametric statistics defined significant differences (two‐tailed *P* < 0.05).

**Results:** 52 male, 52 female, 34 breeds and 22 mixed‐breeds, had median (95% CI) age, weight, and diet duration of 11.9 (10.9–12.3) years; 22.1 (18.8–24.5) kg, and 8.0 (7.1–8.8) years. No dog fed 0.15–0.24 mg Cu/100 kcal (low‐Cu diets) displayed evidence of Cu insufficiency. Water Cu was 0.12(0.08–0.17) ppm. Common histologic features included: glycogen‐type vacuolation (*n* = 61), reactive‐hepatitis (*n* = 20), and neoplasia (*n* = 18). No histologic findings associated with liver‐Cu.

Thirteen low‐Cu diet dogs had significantly lower liver Cu than 91‐dogs fed higher Cu intakes (0.31–0.39 and ≥ 0.40 mg Cu/100 kcal; Table 1). Low‐Cu diet dogs did not accumulate > 355μg Cu/g dwl whereas in higher Cu‐diet dogs, liver Cu > 400μg Cu/g dwl (upper reference range) occurred in 20/91 (22%) and > 600μg Cu/g dwl in 14/91 (15%).

**Conclusion:** Findings demonstrate significant liver‐Cu accumulation with dietary intake ≥ 0.30 mg Cu/100 kcal vs ≤0.24 mg Cu/100 kcal and exonerate water as a source of Cu loading.

**Table:** **Table 1**.

	Score 0	Score 1	Score 2	Score 3	Score 4	Score 5
n	69	19	10	4	1	1
Median	205	341	635	811	1303	1795
95% CI	191–227	310–376	589–665	457–1295	—	—

**Figure:**
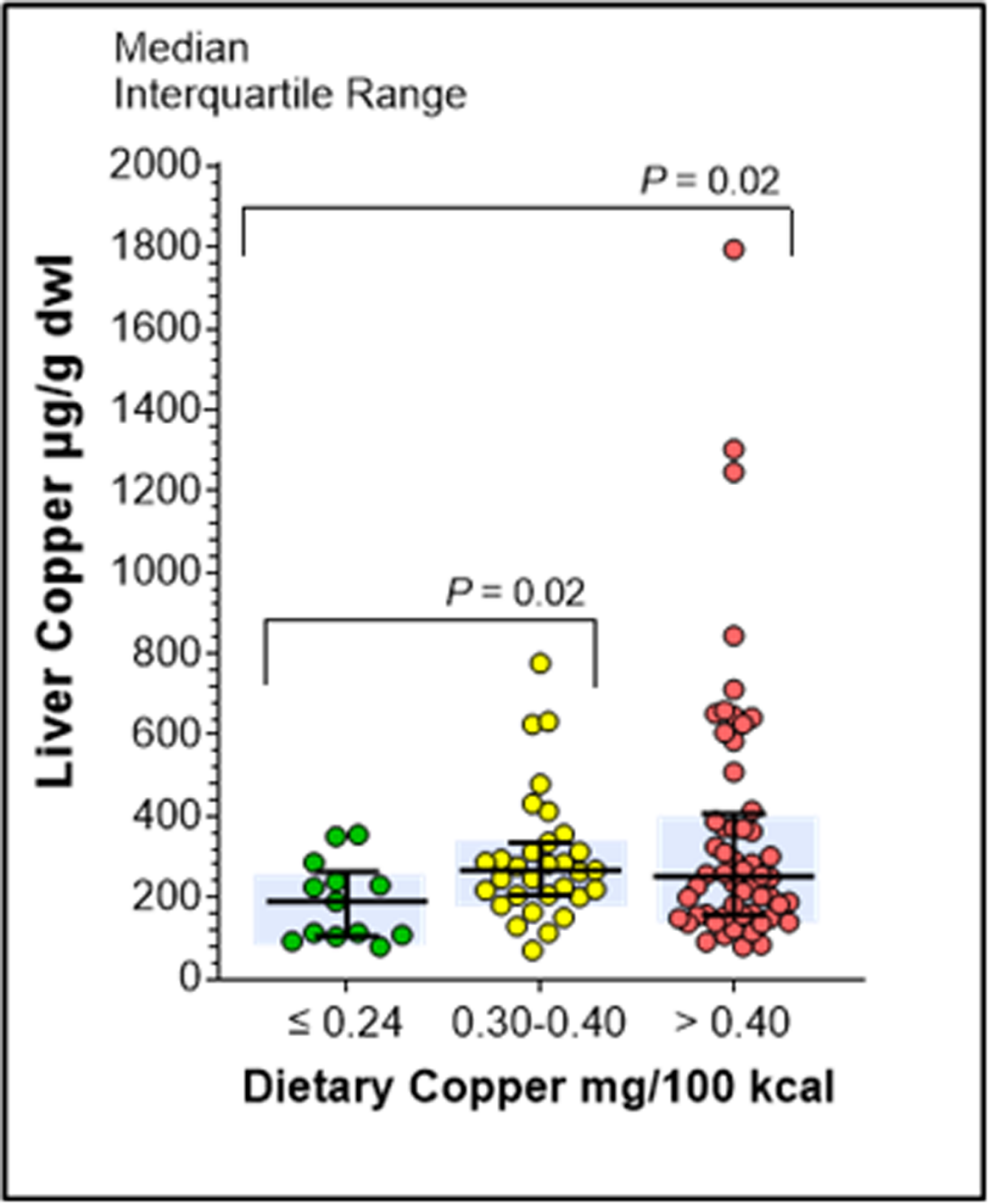
Figure 1

## 181 ABSTRACT HP04: Relative Exchangeable Copper and Ultrafiltered Urine Copper to Creatinine Ratio for Diagnosis of Copper‐Associated Hepatitis in Dogs

### 181.1 **Wei‐Hsiu Chuang** ^1^; João Cavasin^2^; Geoffrey Fosgate^2^; Adrian Tinoco‐Nájera^2^; Floris Dröes^2^; Robert Phillips^2^; John Cullen^3^; Jörg Steiner^2^; Jonathan Lidbury^2^; Kathleen Aicher^2^

#### 181.1.1 ^1^Graduate Assistant (Research), Gastrointestinal Lab, Texas A&M, TX, USA; ^2^Gastrointestinal Laboratory, Texas A&M University, TX, USA; ^3^Department of Public Health and Pathobiology, College of Veterinary Medicine, North Carolina State University, NC, USA

**Background:** Relative exchangeable copper (REC) and urine small copper carrier‐to‐creatinine ratios (Cu/Cr ratio) are increased in humans with Wilson's disease and rodent models.

**Objective:** Determine whether serum REC and 3 kDa ultrafiltered urine Cu/Cr ratio (small copper carrier surrogate) could serve as potential biomarkers for copper‐associated hepatitis (CAH) in dogs.

**Animals:** 16 dogs with CAH, 8 with idiopathic chronic hepatitis (ICH), 18 with congenital portosystemic shunt (CPSS), and 55 healthy controls (HC).

**Methods:** Retrospective cross‐sectional study. Exchangeable copper was measured by incubating serum with 5 g/L EDTA, followed by ultracentrifugation. The REC was calculated as the percentage of exchangeable copper relative to the total serum copper. Urine was ultrafiltered using a 3 kDa filter followed by measurement of copper concentrations normalized to creatinine concentrations. For all samples, copper concentrations were measured by ICP‐MS.

**Results:** REC was higher in CAH dogs than in HC (*P* = 0.03). Ultrafiltered urine Cu/Cr ratio was higher in dogs with CAH and ICH compared to HC (*P* < 0.0001; *P* = 0.01) and compared to dogs with CPSS (*P* = 0.02; *P* = 0.05). There were no other significant differences between groups for either parameter (Table 1). The area under the receiver operating characteristic curve (95% confidence interval) of REC and ultrafiltered urine Cu/Cr ratio for differentiating between dogs with CAH and ICH were 0.59 (0.32–0.86) and 0.61 (0.35–0.86), respectively.

**Table:** **Table 1**.

Group	REC (%)		3 kDa ultrafiltered urine Cu/Cr ratio (ug/mmol)	
	Median	Range	Median	Range
CAH	10.6a	7.1–14.7	11.2a	5.6–57.1
ICH	8.1a	6.9–18.2	15.5a	4.1–50.5
CPSS	NA	NA	6.1b	2.0–13.4
HC	7.8b	5.4–17.8	3.1b	0.7–9.3

Results (median; range) of relative exchangeable copper (REC) and **3 kDa ultrafiltered urine copper** to creatinine ratio (Cu/Cr ratio) in dogs with copper‐associated hepatitis (CAH), dogs with idiopathic chronic hepatitis (ICH), dogs with congenital portosystemic shunt (CPSS), and healthy controls (HC). Medians with the same superscripts are not significantly different at *P* < 0.05.

**Conclusions and Clinical Importance:** Our results do not support the utility of REC or ultrafiltered urine Cu/Cr ratio for differentiating dogs with CAH from ICH.

## 182 ABSTRACT HP05: Coyote Liver Copper Compared to Dogs Implicates Excessive Dietary Copper Supplementation in Commercial Dog Food

### 182.1 **Sharon Center** ^1^, BS, DVM, DACVIM (SAIM); Philip VanVranken^2^, DVM; Andrew Miller^3^, DVM, PhD, DACVP (Anatomic); John Randolph^4^, DVM, DACVIM (SAIM); Cameron Friesen^5^, DVM; Ben Dowdy^6^, BS; Mackenzie Hull^7^

#### 182.1.1 ^1^Emeritus James Law Professor of Internal Medicine, Cornell University, Ithaca, NY, USA; ^2^Associate Veterinarian Dickman Road Veterinary Hospital, Owner Voyager Dog Foods; ^3^Associate Professor, Population Medicine and Diagnostic Sciences, Cornell University, Ithaca, NY, USA; ^4^Emeritus Professor, Clinical Sciences, Cornell University College of Veterinary Medicine, Ithaca, NY, USA; ^5^IVS Imaging; ^6^Director of Operations Voyager Dog Food Company and Brands, Voyager Dog Food; ^7^Vet Assistant, Dickman Road Veterinary Clinic

**Introduction:** Canine risk for liver‐copper (Cu) accumulation from chronic ingestion of commercial diets supplemented with over‐formulated premixes remains controversial.

**Hypothesis:** Liver‐Cu in free‐ranging coyotes (feral wolf‐like‐clade canids consuming a diverse natural diet) may inform this conundrum.

**Animals:** Liver samples: 104 dogs (euthanized for geriatric issues, Michigan primary‐care hospital) and 88 coyotes (70 [Michigan, USA]; 18 [Alberta, Canada], acquired from predator control agents).

**Methods:** Tissues underwent histological evaluation, rhodanine Cu scoring, digital Cu quantification (μg/g dry‐weight liver [dwl]) or bench Cu‐analyses if rhodanine Cu score ≤1. Regional groundwater Cu determinations were acquired. Non‐parametric statistics defined significance (two‐sided P≤0.05).

**Results:** Among 51 male, 53 female, 33 breeds and 28 mixed‐bred dogs median age (11.6, 95% CI: 10.6–12.1) yrs, weight (23.0, 95% CI: 20.1–25.4) kg, and histologic features had no significant association with liver‐Cu. Among 46 male and 42 female coyote samples, all were rhodanine negative, 25 had multifocal eosinophilic granulomas or portal infiltrates, and 7 Alberta coyotes had lymphocytic focal lobular hepatitis. Eosinophilic infiltrates presumably reflect migrating parasites, including *Dirofilaria immitis* for which coyotes are reservoir hosts. As liver Cu in coyote regional groups lacked significant differences, data were consolidated. There was no significant difference between genders (either species) in liver Cu concentrations. Liver Cu was significantly lower in coyotes (vs all dogs [Fig 1]; vs dogs excluding 35 rhodanine‐positive samples [Fig 2]). Groundwater Cu (0.12 mg/L, 95% CI: 0.08–0.17 [Michigan] and 0.006 mg/L, 95% CI 0.07–0.12 [Alberta]) were [sic].

**Conclusions:** Significantly lower liver Cu concentrations in coyotes reconciles with their diverse natural canid diet free of pre‐mix additives, overlaps with liver Cu concentrations in other wolf‐like clade canids and dogs before commercialization of dog food. Findings corroborate concern regarding supraphysiologic Cu supplementation of commercial dog foods and refute groundwater as a relevant Cu source.

**Figure:**
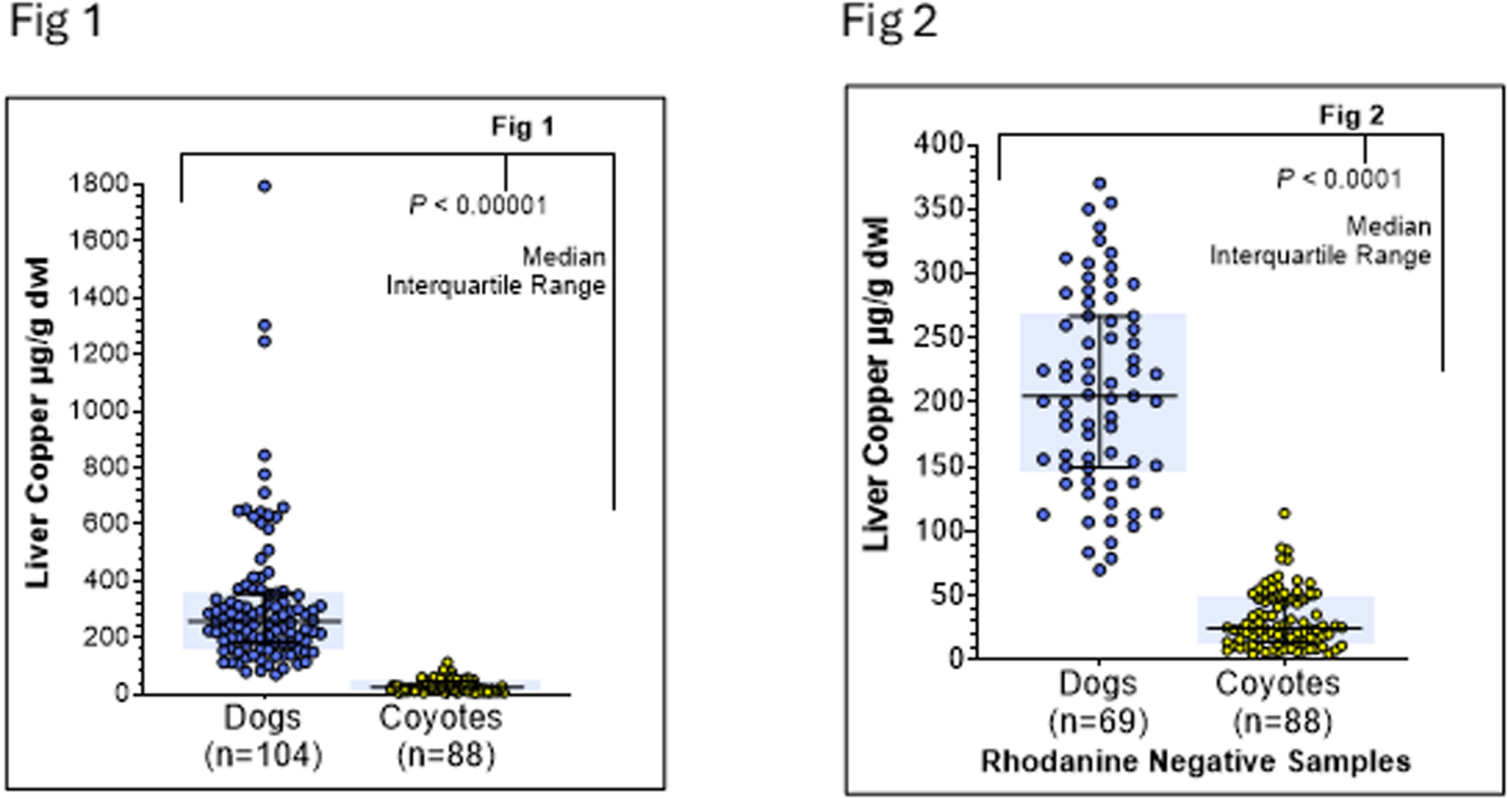
Figure 1. Coyote liver copper compared to dogs implicates excessive dietary copper supplementation in commercial dog food

## 183 ABSTRACT HP06: Transcriptomic Comparison of Liver FNAs and Biopsies in Healthy Cats

### 183.1 **Rommaneeya Leela‐Arporn** ^1^, DVM, PhD; Lyndah Chow^2^; Sarah Shropshire^2^; Catriona MacPhail^2^; Steven Dow^2^; Michael Lappin^2^

#### 183.1.1 ^1^Resident, Small Animal Internal Medicine, Colorado State University, Fort Collins, CO, USA; ^2^Colorado State University, Fort Collins, CO, USA

**Background:** The liver transcriptome can be assessed to identify gene expression patterns, but liver biopsy is invasive. Little is known about whether fine needle aspirate (FNA) samples provide comparable information to biopsies.

**Hypothesis/Objectives:** To define the liver transcriptome of cats using RNA sequencing from liver biopsy and FNA samples obtained from healthy cats and compare findings between the two sample types.

**Animals:** Four healthy purpose‐bred adult cats.

**Methods:** Surgical liver biopsies were obtained during routine ovariectomy with IACUC approval. Fine needle liver aspirates were collected using a 22‐gauge needle from a liver lobe adjacent to the liver biopsy site. RNA was extracted and subjected to Illumina sequencing. Sequence files were aligned to the reference cat genome and assembled using Partek Flow software. Differential analysis was performed with DESeq2, and gene set enrichment analysis (GSEA) was conducted for comparison.

**Results:** A total of 18,502 protein‐coding genes were sequenced. Hepatocyte gene signatures were identified in both biopsies and FNA samples, with high similarity between biological replicates. However, 1,508 genes were significantly upregulated and 1,596 downregulated in FNA samples compared to biopsies. Upregulated pathways were associated with metabolic processes.

**Conclusions and Clinical Importance:** RNA sequencing allows for comprehensive characterization of the feline liver transcriptome. Although hepatocyte gene signatures were detectable in FNAs, significant differential expression suggests potential limitations. Further research, including samples from healthy cats and those with different hepatic disease syndromes, is needed to determine the reliability of FNAs as a less invasive alternative to biopsies.

## 184 ABSTRACT HP07: Signalment, Clinicopathologic, and Imaging Findings in Dogs with Cirrhotic and Non‐Cirrhotic Causes of Portal Hypertension

### 184.1 **Paige Albers** ^1^, VMD (SAIM); Tarini Ullal^2^; Sarah Shropshire^3^; M. Katherine Tolbert^4^; Sara Jablonski^5^

#### 184.1.1 ^1^Resident, Michigan State University, East Lansing, MI, USA; ^2^University of California–Davis, Davis, CA, USA; ^3^Colorado State University, Fort Collins, CO, USA; ^4^Texas A&M University, College Station, TX, USA; ^5^Michigan State University, East Lansing, MI, USA

**Background:** The clinical presentation of hepatic portal hypertension due to cirrhotic vs. non‐cirrhotic causes is similar in dogs. Thus, dogs with portal hypertension due to non‐cirrhotic causes can be misdiagnosed with cirrhosis. Limited information is available comparing clinical and diagnostic findings in dogs with cirrhotic vs. non‐cirrhotic hepatic portal hypertension.

**Objectives:** To retrospectively evaluate for differences in signalment, physical exam, clinicopathologic, and imaging findings between dogs with cirrhotic and non‐cirrhotic causes of hepatic portal hypertension.

**Animals:** 62 dogs with portal hypertension (*n* = 31 due to cirrhosis; *n* = 31 due to non‐cirrhotic causes).

**Methods:** Multi‐institutional retrospective study. Dogs were included if they had clinical evidence of portal hypertension and liver dysfunction. Abdominal imaging and liver biopsy were required. Dogs were identified as having a cirrhotic or non‐cirrhotic cause of portal hypertension based on liver biopsy (WSAVA criteria applied).

**Results:** Dogs with cirrhotic causes of portal hypertension were older (*P* < 0.001) and had higher serum bilirubin (*P* < 0.001) and ALP (*P* < 0.001) concentrations compared to dogs with non‐cirrhotic causes of portal hypertension. Serum albumin concentrations (*P* = 0.04) and platelet count (*P* = 0.004) were lower in dogs with cirrhotic vs. non‐cirrhotic causes. Dogs with cirrhotic causes of portal hypertension also had nodular change (*P* < 0.001) and heterogeneity (*P* < 0.001) of the liver reported more frequently on abdominal ultrasound.

**Conclusions and Clinical Importance:** Liver biopsy remains the gold standard for diagnosis of the cause of hepatic portal hypertension in dogs. However, clinicopathologic and imaging findings can be used to prioritize between cirrhotic and non‐cirrhotic causes.

## 185 ABSTRACT HP08: Serum and Fecal Bile Acid Profiles in Dogs with Liver Disease

### 185.1 **Floris Dröes** ^1^, DVM, MRCVS, PGCert, VMS; Amanda Blake^2^, MS, PhD; João Cavasin^3^, DVM, DACVP; Robert Phillips^4^, MS, PhD; Adrian Tinoco Najera^1^, PhD, MVZ, EMCPG, MMVZ; Joerg Steiner^5^, MedVet, DrMedVet, PhD, DACVIM, DECVIM‐CA, AGAF; Jan Suchodolski^6^, MedVet, DrVetMed, PhD, AGAF, DACVM; Jonathan Lidbury^7^, BVMS, MRCVS, PhD, DACVIM, DECVIM‐CA

#### 185.1.1 ^1^Graduate Research Assistant, Gastrointestinal Laboratory, School of Veterinary Medicine and Biomedical Sciences, Texas A&M University, College Station, TX, USA; ^2^Associate Research Scientist, Small Animal Clinical Sciences, Gastrointestinal Laboratory, Texas A&M University, College Station, TX, USA; ^3^Clinical Assistant Professor, Small Animal Clinical Sciences, Gastrointestinal Laboratory, Texas A&M University, College Station, TX, USA; ^4^Postdoctoral Research Associate, Small Animal Clinical Sciences, Gastrointestinal Laboratory, Texas A&M University, College Station, TX, USA; ^5^Regents Professor, University Distinguished Professor, Dr. Mark Morris Chair in Small Animal Gastroenterology and Nutrition, Director of Gastrointestinal Laboratory, Gastrointestinal Laboratory, Small Animal Clinical Sciences, Texas A&M University, College Station, TX, USA; ^6^Professor and Associate Director for Research of the GI Lab, Purina PetCare Endowed Chair for Microbiome Research, Gastrointestinal Laboratory, Small Animal Clinical Sciences, Texas A&M University, College Station, TX, USA; ^7^Associate Professor, Small Animal Internal Medicine, Gastrointestinal Laboratory, Associate Director for Clinical Services, Assistant Department Head for Research and Graduate Studies, Rob and Roxann Bilger Chair in Feline Hepatology, Small Animal Clinical Sciences, Texas A&M University, College Station, TX, USA

**Background:** Synthesis and secretion of bile acids (BA) is a key hepatic function. Altered serum and fecal BA concentrations have been described in humans with liver disease.

**Objective:** Compare serum BA (SBA) and fecal BA (FBA) profiles between healthy control dogs (controls) and dogs with liver disease.

**Animals:** 26 controls, 5 dogs with acute liver disease (ALD), 5 dogs with chronic hepatitis (CH), and 6 dogs with hepatocellular carcinoma (HCC). Controls had not received oral antibiotics for ≥ 6 months, dogs with liver disease for ≥ 3 months. Dogs with liver disease were not receiving any choleretics.

**Methods:** Observational cross‐sectional study. Concentrations of 29 SBA and FBA (including unconjugated, taurine‐ and glycine‐conjugated, and iso‐ and oxo‐BA) were measured by a LC‐MS/MS method. Groups were compared using Kruskal‐Wallis tests adjusted for multiple comparisons. For significant parameters, individual groups were compared with a Dunn's *post hoc* test.

**Results:** In ALD, serum glycolithocholic acid (*p* = 0.0014), serum taurocholic acid (*p* = 0.0019), and fecal 3‐oxo‐chenodeoxycholic acid (*p* = 0.0131) were higher compared to controls. In CH, fecal 3‐oxo‐chenodeoxycholic acid (*p* = 0.0323) was higher compared to controls. In HCC, serum taurocholic acid (*p* = 0.0281) and fecal chenodeoxycholic acid (*p* = 0.0047) were higher compared to controls. Other comparisons between groups were not statistically significantly different.

**Conclusions and Clinical Importance:** Dogs with ALD, CH, and HCC had altered SBA and FBA profiles compared to controls.

## 186 ABSTRACT ID01: *Castellaniella* spp. as a Potential Emerging Pathogen in Dogs

### 186.1 **Lina Crespo Bilhalva** ^1^; Sabrina Manley^2^; Paola Guasch^2^; Janina Krumbeck^3^; Mark Yacoub^3^; Emily Brinker^4^; Esther Gisela Martinez‐Romero^4^; Andrea Santos^5^; Francisco Conrado^4^; Joyce Knoll^4^; Leslie Sharkey^3^

#### 186.1.1 ^1^Clinical Pathology Resident, Cummings School of Veterinary Medicine, Tufts University, MA, USA; ^2^Department of Clinical Sciences, Cummings School of Veterinary Medicine, Tufts University, MA, USA; ^3^MiDOG Animal Diagnostics; ^4^Department of Comparative Pathobiology, Cummings School of Veterinary Medicine, Tufts University, MA, USA; ^5^Department of Comparative Pathobiology, Purdue University College of Veterinary Medicine, IN, USA

**Background:** *Castellaniella* is a genus of gram‐negative bacteria within the family Alcaligenaceae, known for its ability to reduce nitrate. These organisms have been isolated from various environments, demonstrating adaptability to highly human‐impacted areas. Although generally not considered pathogenic, *Castellaniella* spp. have been associated with mass mortality events in Daurian pikas and broilers, as well as one case in a harbor porpoise. To date, no disease caused by *Castellaniella* spp. has been reported in companion animals.

**Hypothesis/Objectives:** To report and describe *Castellaniella* sp. infection in dogs.

**Animals:** In late 2024, two unrelated client‐owned dogs from the same geographic region were presented with pyothorax and suffered a sudden clinical decline, resulting in death within 24 h.

**Methods:** Diagnostic evaluations included fluid analysis, bacterial culture, 16S rRNA gene amplification, and necropsy.

**Results:** Cytologic examination of the exudative effusion identified intracellular short rods, predominantly within macrophages. Bacterial culture yielded pure gram‐negative colonies but failed to determine the organism's species. Amplification of the 16S rRNA gene showed 99–100% similarity to two species within the genus *Castellaniella*. Necropsy findings did not reveal potential inoculation routes.

**Conclusions and Clinical Importance:** These are the first documented cases of *Castellaniella* sp. infection in dogs. The monomorphic bacterial population and its unusual presence within macrophages prompted further investigation. Pending genomic analysis may confirm whether the organisms belong to the same species and help assess the risk of a potential outbreak. The severity and rapid progression of *Castellaniella* spp. infections underscore the critical need for heightened awareness among veterinary professionals.

**Figure:**
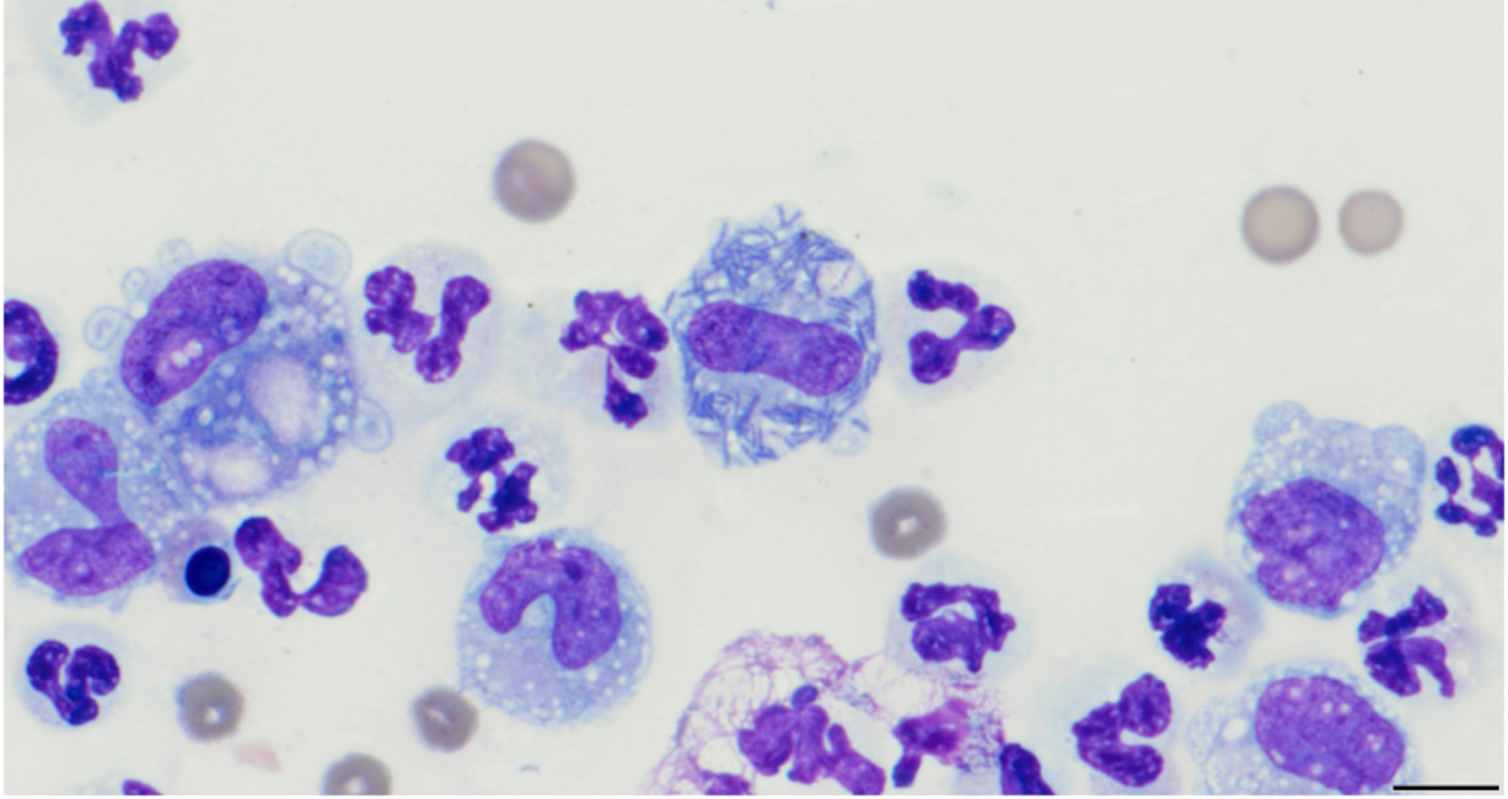
**Figure 1. Photomicrograph of a direct smear from a pleural effusion in dog**. Numerous rod‐shaped bacteria are visible within a macrophage (center). Wright‐Giemsa stain, 100x objective. Bar=10 μg.

## 187 ABSTRACT ID02: Agreement Between Collection Techniques for Detection of *Histoplasma* Antigen in the Urine of Dogs

### 187.1 **Katie Como** ^1^; Kate Aicher^2^, DVM, DACVIM (SAIM); Song Zhang^3^, PhD; Andrew Hanzlicek^4^, DVM, MS, DACVIM (SAIM); Emily Gould^2^, DVM, MS, PhD, DACVIM (SAIM)

#### 187.1.1 ^1^Resident in Small Animal Internal Medicine, College of Veterinary Medicine and Biomedical Sciences, Texas A&M University, TX, USA; ^2^Assistant Professor, Gastrointestinal Laboratory, Department of Small Animal Clinical Sciences, College of Veterinary Medicine and Biomedical Sciences, Texas A&M University, TX, USA; ^3^Peter O'Donnell Jr. School of Public Health, UT Southwestern Medical Center, TX, USA; ^4^Director of Veterinary Medicine and Research, MiraVista Diagnostics

**Background:** A commercially available *Histoplasma* urine antigen EIA (EIA) test is commonly used to support the diagnosis of histoplasmosis in dogs, but a gold standard urine collection technique has yet to be established.

**Objective:** Evaluate the agreement between three urine collection techniques—sterile cystocentesis (cysto), free catch (FC), and contaminated cystocentesis (contam)—using the EIA in dogs in an endemic region.

**Animals:** 30 dogs suspected of histoplasmosis and 30 temporally matched, healthy dogs.

**Methods:** In this 12‐month prospective study, three urine samples (cysto, FC, contam) were collected within a 24‐h period from each dog. Agreement between collection techniques was assessed using McNemar's test and kappa coefficients, with false positive rate (FPR) for all dogs combined calculated using the clinical diagnosis as the reference standard.

**Results:** Three clinically ill dogs were diagnosed with histoplasmosis. Cysto and contam samples had substantial agreement (kappa = 0.68, 95% CI 0.39%–0.97%), while moderate agreement was found between cysto and FC (Kappa = 0.45, 95% CI 0.07%–0.82%) and FC and contam (kappa = 0.42, 95% CI 0.10%–0.75%) samples. The overall FPR was 16% (9/57; 95% CI 6%–25%). Cysto samples had the lowest FPR (4%, 2/57; 95% CI 1%–12%), followed by FC (7%, 4/57; 95% CI 3%–17%), and contam (11%, 6/57; 95% CI 5%–21%).

**Conclusions and Clinical Importance:** While all collection techniques demonstrated reasonable agreement, FC samples might yield false positives. Clinicians should confirm low‐level positive FC results with sterile cystocentesis to improve diagnostic accuracy and clinical decision making for histoplasmosis in dogs.

## 188 ABSTRACT ID03: Negative or Low Levels of Antibodies in 27 Dogs With Clinical Leishmaniosis

### 188.1 **Elizabeth Snyder** ^1^; Jorge Castro^2^; Carolina Arenas^3^; Rodolfo Oliveria Leal^4^; Maria Dolores Tabar^5^; Carles Blasi^6^; Coralie Bertolani^7^; Andres Salas^8^; Ignacio Mesa^9^; Xavier Roura^10^; Paolo Silvestrini^11^

#### 188.1.1 ^1^Internal Medicine Resident, Ryan Veterinary Hospital, University of Pennsylvania, PA, USA; ^2^AniCura Vetsia; ^3^AniCura Valencia Sur; ^4^University of Lisbon, Portugal; ^5^AniCura Hospital Veterinario San Vicente; ^6^University of Dublin, Ireland; ^7^Hospital Veterinari Canis; ^8^Southfields Veterinary Specialists; ^9^Auna Especialidades Veterinarias; ^10^Universitat Autonoma de Barcelona, Spain; ^11^University of Pennsylvania, PA, USA

**Background:** In canine leishmaniosis (CanL), high antibody titer corresponds with clinical signs, except in dogs with papular dermatitis or uveitis where serology is often low or negative.

**Objectives:** To describe the clinical findings of dogs with leishmaniosis but low or negative serology.

**Animals:** 27 client‐owned dogs with leishmaniosis.

**Methods:** Retrospective case series.

**Results:** The 27 dogs were grouped by their main clinical presentation: gastrointestinal (9), dermatologic (8), hematologic (6), musculoskeletal (2), neurological (1), and renal (1). Five dogs were on immunosuppressive therapy. Serology was negative in 15 dogs and low in 12. CanL diagnosis was based on a combination of cytology (lymph nodes=6, spleen = 6, liver=3, cutaneous lesions=2, conjunctiva=2), PCR (blood=3, lymph nodes=2, bone marrow=2, CSF=1, synovial fluid=1), and bone marrow (5), intestinal (2), and skin biopsy (1). Dogs were treated with allopurinol and meglumine antimoniate (21) or miltefosine (5); one dog received only allopurinol. Most (20/27) responded well, and serology remained low or negative in dogs with favorable outcomes.

**Conclusions and Clinical Importance:** Some dogs with clinical active leishmaniosis exhibit low or negative serology, which could be due to concurrent immunosuppression, high numbers of immune complexes, or infections with different *Leishmania* species inducing antibodies not detected by the assays used for *Leishmania infantum*. Additionally, some dogs may develop clinical signs despite mounting a Th1 immune response with low antibody production. When CanL is suspected, but serology is negative or low, further investigations are warranted.

## 189 ABSTRACT ID04: Outbreak of Leptospirosis in Dogs from Los Angeles County, California, USA, 2021

### 189.1 **Max Randolph** ^1^; Jarlath Nally^2^; David Wagner^3^; Nathan Stone^3^; Sean Yoshimoto^4^; Betty Chow^5^; Camila Hamond^6^; Karen LeCount^7^; Hans van der Linden^8^; Krystle Reagan^9^; Jamie Sebastian^10^; Alexander Schreiber^4^; Jane Sykes^9^

#### 189.1.1 ^1^Resident III, University of California–Davis, CA, USA; ^2^USDA; ^3^Northern Arizona University, AZ, USA; ^4^VCA West Los Angeles Hospital, CA, USA; ^5^VCA Animal Specialty and Emergency Center; ^6^University of Connecticut, CT, USA; ^7^USDA; ^8^University of Amsterdam, The Netherlands; ^9^University of California–Davis, CA, USA; ^10^Kansas State University, KS, USA

**Background:** A leptospirosis outbreak was suspected in west Los Angeles county (April–December 2021).

**Hypothesis/Objectives:** To describe signalment, clinicopathologic and microbiologic findings, and diagnostic test results in affected dogs.

**Animals:** Fifty‐seven dogs with leptospirosis evaluated at two Los Angeles county specialty hospitals.

**Methods:** Signalment variables were compared to the hospital population (*n* = 19,870) (Fisher's exact or chi‐square analysis; significance p≤0.05). Clinicopathologic and microbiologic findings were described for cases.

**Results:** Cases were more likely ≤ 5 years old, ≥ 15 kg, and male (odds ratio [95% confidence interval] 8.1 [3.8–17.4], 4.2 [2.3–7.4], and 1.8 [1.1–3.2], respectively) than the hospital population. Siberian huskies, labradoodles, and golden retrievers were overrepresented (7.9 [2.6–23.9], 5.3 [2.3–12.7], and 3.5 [1.5–8.3], respectively). Exposure to boarding/daycare was reported for 26/57 dogs, 19 between 7/15/21 and 9/6/21. Forty‐five of 47 dogs were unvaccinated for leptospirosis. Laboratory findings included anemia (52%), neutrophilia (49%), thrombocytopenia (33%), increased serum creatinine (88%), increased serum liver enzymes (18%), and glucosuria (37%). Diagnostic testing included WITNESS Lepto (Zoetis), SNAP Lepto (IDEXX), acute microscopic agglutination testing, blood and urine PCR (positive in 19/26, 8/9, 22/28, 15/55, and 47/52, respectively). Isolates from urine (4/4 dogs) were serotyped as *Leptospira interrogans* serogroup Canicola serovar Canicola and were clonal using whole genome sequencing.

**Conclusions and Clinical Importance:** Findings supported a leptospirosis outbreak in association with boarding/daycare in a predominantly young‐adult, unvaccinated dog population. Urine PCR was a sensitive diagnostic test. Diagnosis was optimized using a combination of serologic tests and blood and urine PCR assays.

## 190 ABSTRACT ID05: Vector‐Borne Infections in Dogs with Immune‐Mediated Hemolytic Anemia, Thrombocytopenia, or Polyarthritis in Northern California

### 190.1 **Sarah Michalak** ^1^; William Vernau^2^, BSc, BVMS, DVSc, PhD, DAVP (Clin Path); Jane Sykes^3^, BVSc (Hons), PhD, MPH, MBA, FNAP, DACVIM (SAIM)

#### 190.1.1 ^1^Small Animal Internal Medicine Resident, University of California, Davis, CA, USA; ^2^Professor, Pathology, Microbiology, and Immunology, University of California, Davis, CA, USA; ^3^Professor, Medicine and Epidemiology, University of California, Davis, CA, USA

**Background:** Testing and empiric treatment for vector‐borne infections (VBIs) are commonly pursued in dogs with immune‐mediated diseases despite lack of information on regional prevalence.

**Hypothesis/Objectives:** Determine test positivity rate and associated clinicopathologic features of VBIs in dogs with immune‐mediated hemolytic anemia (IMHA), immune thrombocytopenia (ITP), and immune‐mediated polyarthritis (IMPA) at a teaching hospital in Northern California (2017–2022).

**Animals:** Client‐owned dogs meeting inclusion criteria for IMHA (*n* = 94), ITP (*n* = 101), or IMPA (*n* = 100) that underwent testing for VBIs (SNAP 4Dx Plus [IDEXX] with or without PCR or quantitative serology).

**Methods:** Retrospective study. VBI test positivity rate was determined. Clinicopathologic features in test‐positive and test‐negative dogs were compared.

**Results:** Six (2%) of 295 dogs had VBIs; all responded to specific antimicrobial treatment, 5 without immunosuppression. IMHA‐group infections were *Ehrlichia canis* (4Dx+/PCR+) (1) and concurrent *E. canis* (4Dx+/PCR+) and *Anaplasma platys* (4Dx‐/PCR+) (1). ITP‐group infections were *Anaplasma phagocytophilum* (4Dx‐/PCR+/morulae+) (1), and *Babesia conradae* (PCR+/piroplasm+) (1). Two IMPA dogs were diagnosed with Lyme polyarthritis (4Dx+/high QuantC6 [125 and 256 U/mL]). Serum globulin concentration was higher in VBI dogs than in test‐negative dogs (*P* = 0.02); 5/6 dogs with VBIs were hyperglobulinemic versus 33% of test‐negative dogs. Four dogs with VBIs had travel histories to endemic regions.

**Conclusions and Clinical Importance:** VBIs in this population of dogs were rare. For this population, testing for VBIs should be focused on those with hyperglobulinemia and/or relevant travel history; empiric antimicrobial therapy appears unwarranted. Given their acute nature, serologic testing may have limited utility for diagnosis of rickettsioses when compared with PCR.

## 191 ABSTRACT ID06: Canine Blastomycosis: A Retrospective Study of 126 Cases in Tennessee

### 191.1 **Leonardo Spadafino** ^1^; Ashley Hartley^2^, DVM, PhD, DACVIM (SAIM)

#### 191.1.1 ^1^Veterinary Student, College of Veterinary Medicine, University of Tennessee, TN, USA; ^2^Assistant Professor of Internal Medicine, Small Animal Clinical Sciences, College of Veterinary Medicine, University of Tennessee, TN, USA

**Background:** *Blastomyces dermatitidis*, a fungus endemic to Mississippi River Valley, infects both dogs and humans. Canine blastomycosis was last characterized in Tennessee with 78 cases presenting to the University of Tennessee Veterinary Medical Center (UTVMC) during 1977–1999. A *Blastomyces* antigen quantitative (BdAg) test has since emerged as a less invasive yet sensitive diagnostic for blastomycosis.

**Objective:** The objective was to identify cases of canine blastomycosis at UTVMC and characterize the presentation, clinicopathologic and imaging abnormalities, and treatments selected during 2014–2023.

**Animals and Methods:** One hundred thirty‐one (131) cases of canine blastomycosis were identified from a hospital population using laboratory database searches for BdAg and pathology (i.e., cytology, histopathology, and necropsy) submissions.

**Results:** Electronic medical record and imaging report screening confirmed clinical diagnosis in 126 cases. Clinical cases were all ages, gender, and size, yet most often <5 years of age (*n* = 65/126, 52%) and weighing > 10 kg (*n* = 57/126, 45%). Emergency (*n* = 57/126, 45%), ophthalmology (*n* = 28/126, 22%), and internal medicine (*n* = 25/126, 19.8%) services were most visited at initial presentation. Respiratory, ocular, and dermatological manifestations were most commonly involved, both as primary and total organ systems. Notable clinicopathologic abnormalities included leukocytosis, neutrophilia, hyperproteinemia, hypoalbuminemia, hyperglobulinemia, elevated C‐reactive protein, and low iron. Thoracic imaging commonly revealed structured interstitial patterns (50/86, 58.1%), with miliary (20/86, 23.3%) and nodular (38/86, 44.2%) features. Itraconazole was the most frequently prescribed antifungal (89/106, 84%).

**Conclusions:** Veterinarians should consider *Blastomyces* sp. infection for a variety of clinical presentations.

## 192 ABSTRACT ID07: Performance of a Proprietary Silicon Surface Multiplex Immunoassay for *Ehrlichia* spp Antibody Detection in Dogs

### 192.1 **Graham Bilbrough** ^1^; Greg Freeman^2^; Michael Lappin^3^; Christian Leutenegger^2^; Jere Mcbride^4^; Irina Sokolchik^2^; Jennifer Wilcox^2^

#### 192.1.1 ^1^Director Clinical Expertise, Antech/MARS; ^2^Antech/MARS; ^3^Colorado State University, CO, USA; ^4^University of Texas Medical Branch (UTMB)

**Background:** The Companion Animal Parasite Council predicts continued range expansion of United States (U.S.) and Canadian tick vectors. Effective surveillance for tick‐transmitted pathogens in dogs, such as *Ehrlichia* spp. (*E. canis*, *E. chaffeensis*, and *E. ewingii*), is needed. Detection of antibodies (serology) can be an accurate method to test for canine pathogen exposure in the U.S. and Canada.

**Objectives:** To analyze performance of a proprietary silicon surface multiplex immunoassay (SSMIA) using two combined novel markers to detect *Ehrlichia* spp. antibodies (Accuplex®, Antech Diagnostics, Mars Petcare Science and Diagnostics) in canine serum samples compared to indirect fluorescent antibody (IFA) and a commercially available point‐of‐care ELISA (IDEXX 4Dx® Plus‐POC‐ELISA).

**Animals:** Anonymized retention‐released serum samples from dogs in the Southeastern US submitted to a commercial reference laboratory.

**Methods:** Samples were evaluated by SSMIA, IFA, and POC‐ELISA. IFA was used to classify samples (true positive/true negative). SSMIA was evaluated compared to IFA prioritizing specificity [with 95% confidence interval (95% CI)] and kappa statistics (95% CI) for agreement comparing SSMIA and POC‐ELISA to IFA. *Anaplasma* spp. antibody‐detected samples were excluded due to potential IFA cross‐reactivity.

**Results:** For 309 evaluable samples, SSMIA (2 markers combined) compared to IFA yielded a 94% sensitivity (95% CI: 86%–97%), 97% specificity (95% CI: 95%–99%), and kappa of 0.9 (95% CI: 0.84%–0.95) and 0.67 (95% CI: 0.57–0.77) for SSMIA and POC‐ELISA compared to IFA, respectively.

**Clinical Importance:** This study demonstrates SSMIA is an effective and accurate tool for *Ehrlichia* spp. exposure detection in dogs and compares favorably to other commercially available products.

## 193 ABSTRACT ID08: Use of Acute Phase Proteins in Cats With Feline Infectious Peritonitis

### 193.1 **Petra Cerna** ^1^; Jennifer Hawley^2^; Michael Lappin^2^; A. Russell Moore^2^

#### 193.1.1 ^1^PhD Candidate, Colorado State University; ^2^Colorado State University

**Background:** Serum acute phase proteins α‐1 acid glycoprotein (AGP), serum amyloid A (SAA), and albumin:globulin (A:G) change with feline infectious peritonitis (FIP) and may help monitor therapy response and detect FIP relapse.

**Hypothesis/Objectives:** Determine if AGP, SAA, or A:G can aid in monitoring FIP cats on antiviral therapy and identifying relapses.

**Animals:** Cats treated for novel (*n* = 15) or relapsing (*n* = 7) FIP.

**Methods:** A commercial feline AGP assay (Shima, Tokyo, Japan) was modified for an automated biochemistry analyzer and analytic performance was verified. Longitudinal serum samples (T0, T4, T8, and T12 weeks) were evaluated for AGP, SAA, and electrophoresis derived A:G. Friedman's or Mann‐Whitney tests were used.

**Results:** The AGP assay performed acceptably: coefficient of variation was <8.1% and the assay was linear and reportable between 200–2000 mg/L (*r* ^2^=0.99) with acceptable correlation with SAA (*r* ^2^=0.75). SAA (mea*n* = 52.9 mg/L) and AGP (mea*n* = 2405 mg/L) were higher and A:G (mea*n* = 0.52) was lower at novel T0 than novel T4, T8, or T12 (all *p* < 0.04; Figure 1). Similar relationship existed between novel T0 and relapse T0 for SAA (relapse T0 mea*n* = 8.5 mg/L), AGP (relapse T0 mea*n* = 758 mg/L) and A:G (relapse T0 mea*n* = 1.01), all *p* < 0.01. There was no statistical difference between novel T12 and relapse T0 (all *p* > 0.32; Figure 2).

**Conclusions and Clinical Importance:** AGP, SAA, and A:G were associated with therapy response in novel FIP but did not detect FIP relapse.

**Figure:**
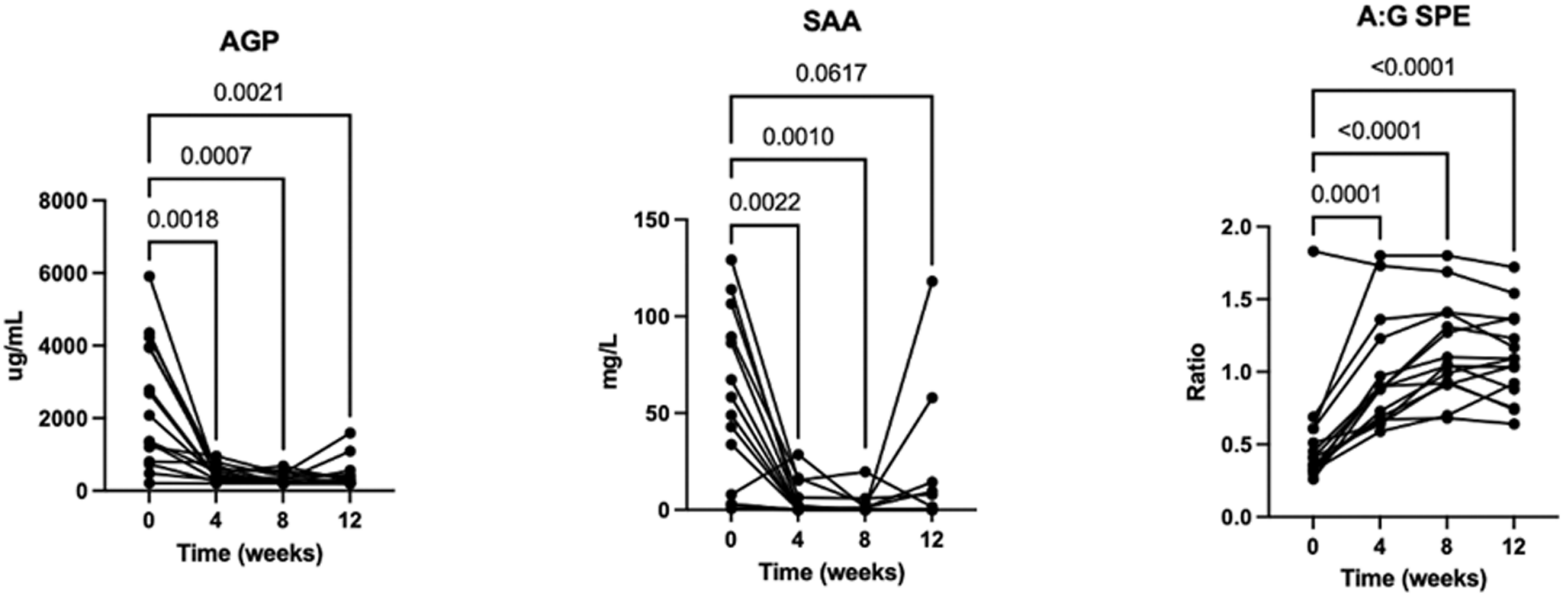
**Figure 1** Serum α1‐acid glycoprotein (AGP; left) and serum amyloid A (SAA; middle) and albumin:globulin by serum electrophoresis (A:G SPE, right) in 15 cats at initial FIP diagnosis (week 0) and at weeks 4, 8, and 12 while on treatment with antiviral (molnupiravir). Clinical improvement was noted in all cats at weeks 4, 8, and 12.

**Figure:**
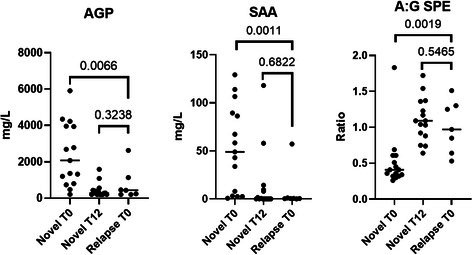
**Figure 2** Serum α1‐acid glycoprotein (AGP; left) and serum amyloid A (SAA; middle) and albumin:globulin by serum electrophoresis (A:G SPE, right) in 15 cats at initial FIP diagnosis at week 0 (T0) and week 12 (T12) and 7 cats diagnosed with relapse FIP at week 0 (T0).

## 194 ABSTRACT ID09: Atovaquone Resistance M121I Polymorphism in *Babesia gibsoni* Isolates From Dogs

### 194.1 **Christian Leutenegger** ^1^; Myriam Belanger^2^; Michelle Evason^2^; Pablo Jimenez Castro^2^; Christian Savard^2^; Jeffrey Tereski^2^

#### 194.1.1 ^1^Vice President, Research and Development, Antech Diagnostics, Inc., Mars Petcare Science and Diagnostics; ^2^Antech/MARS

**Background:** *Babesia gibsoni* is a global pathogen of clinical importance in dogs. Resistance to atovaquone, based on the cytochrome b gene M121I mutation, has been increasingly identified in the United States, Japan, China, and India.

**Objectives:** To determine *B. gibsoni* proportion positive in a canine sample set collected in Hong Kong, and to validate a molecular test in the *B. gibsoni* detected samples for the single nucleotide polymorphism G363T/A causing the amino acid change in codon 121 from methionine to isoleucine (M121I).

**Animals:** A total of 56 anonymized canine remnant whole blood samples were evaluated with a vector‐borne disease (VBD) pathogen qPCR panel, including qPCR tests for *B. gibsoni* and the M121I Atovaquone resistance polymorphism.

**Methods:** Nucleic acid extracts were analyzed using a hydrolysis probe‐based real‐time PCR (qPCR) test for canine VBD pathogens, the M121I polymorphism, and an internal sample control. The M121I qPCR hydrolysis probe was specific for the G363T/A polymorphism. The cytochrome b gene G363T/A polymorphism was confirmed by Sanger sequencing. Frequency calculations were performed for: overall *Babesia* spp., *B. gibsoni*, and the G363T/A polymorphism.

**Results:** *Babesia* spp. were detected in 66.1% (37/56) samples. Of these, the majority detected as *B. gibsoni* (86.5%, 32/37), with 25% (8/32) detected for the G363T/A polymorphism, and confirmed by Sanger sequencing. VBD pathogen detection was described (Table 1).

**Conclusions:** *Babesia gibsoni*, and concurrent atovaquone resistance (M121I polymorphism), were widely detected in this cohort. Our work describes, and validates, a VBD qPCR panel that enables accurate pathogen detection, and targeted antimicrobial treatment, in dogs.

**Table:** **Table 1**.

Pathogen/SNP detected	Count	Frequency
*Babesia* spp.	37	66.1%
*Babesia gibsoni*	32	32/37=86.4%
*B. gibsoni* Atovaquone G363T/A	8	8/32=25.0%
*Babesia canis*	5	5/37=13.5%
*Ehrlichia canis*	3	5.4%
*Hepatozoon canis*	1	1.8%
VBD qPCR undetected	17	30.4%
VBD qPCR detected	40	71.4%
	**56**	

Validation of the Atovaquone resistance M121I polymorphism as part of a broad vector‐borne disease pathogen qPCR panel for dogs.

## 195 ABSTRACT ID10: Comparison of Gastrointestinal Parasite Frequency in Feral and Pet Cats in Houston, Texas

### 195.1 **Michelle Evason** ^1^; Harper Frye^2^; Jeffrey Tereski^3^; Samantha Loo^3^; Christian Savard^3^; Pablo Jimenez Castro^3^; Michelle Frye^2^; Christian Leutenegger^4^, DVM, BSc, PhD, FVH

#### 195.1.1 ^1^Global Director of Veterinary Clinical Education, Antech/Mars Petcare Science and Diagnostics; ^2^Kedi Labs, Inc.; ^3^Antech Diagnostics, Inc.; ^4^Vice President, R&D, Antech Diagnostics, Inc.

**Background:** Gastrointestinal parasites in pet cats are frequently detected in wellness tests. Zoonotic parasites are a One Health concern and early detection and identification reduces exposure and risk of human infection. Intestinal parasite frequency in feral cat populations however is less well characterized and may be a source of continued re‐infection for the pet cat population.

**Objectives:** To compare the frequency and diversity of gastrointestinal parasites in feral and pet cats in Houston, Texas, over a 4‐month period.

**Animals:** Stool samples were collected from 250 feral cats undergoing spay/neuter procedures at a Houston Spay Neuter. The pet cat population consisted of 423 fecal samples collected from pet cats and submitted to a reference lab during the same time frame, March to June 2024, and geographic location.

**Methods:** Fecal samples were analyzed using molecular tests for 20 parasites, benzimidazole resistance markers in the canine hookworm and for *Giardia* with zoonotic potential (KeyScreen® GI Parasite PCR, Antech Diagnostics, Inc. Mars Petcare Science and Diagnostics). Data was compared to a time and geography matched data set from samples submitted to a reference laboratory.

**Results:** The overall frequency of any intestinal parasites detected in feral cats was almost 3‐times higher than in pet cats (91.6% vs 34.8%, Table 1). *Cryptosporidium felis* and *Ancylostoma* spp. were 8–18‐times higher in feral cats.

**Conclusions:** Intestinal parasites frequencies in a feral cat population are substantially higher than in a comparable pet cat population and constitute a risk population from a One Health perspective.

**Table:** **Table 1**.

	Pet cats %	Feral cats %
Parasites found	34.8%	91.6%
Single parasite infections	22.9%	61.2%
Parasitic co‐infections	51.5%	33.2%
**Individual parasites**	**Pet cats %**	**Feral cats %**
*Ancylostoma* spp.	3.1%	55.3%
*Giardia duodenalis*	14.7%	28.3%
*Giardia duodenalis* zoonotic	9.7%	10.4%
*Dipylidium caninum*	10.4%	40.5%
*Cystoisospora* spp.	9.0%	34.6%
*Toxocara* spp.	4.5%	21.1%
*Cryptosporidium felis*	1.7%	13.5%
*Toxoplasma gondii*	5.7%	3.0%
*Taenia* spp.	0.2%	2.5%
*Tritrichomonas blagburni*	0.01%	0.8%

Comparison of gastrointestinal parasitic infections in pet and feral cats.

## 196 ABSTRACT ID11: Molnupiravir Pharmacokinetics Study in Cats With Naturally Occurring Feline Infectious Peritonitis

### 196.1 **Petra Cerna** ^1^; Luke Wittenburg^2^; McKenna Willis^3^; Britta Siegenthaler^3^; Jennifer Hawley^3^; Michael Lappin^3^

#### 196.1.1 ^1^PhD Candidate, Colorado State University; ^2^Department of Surgical and Radiological Sciences, University of California–Davis; ^3^Colorado State University

**Background:** Antiviral drugs like EIDD‐2801 (molnupiravir; MPV) have been successfully used in treatment of feline infectious peritonitis (FIP). Previous study on pharmacokinetics of MPV in healthy cats showed promise for its use and safety.

**Hypothesis/Objectives:** To determine the pharmacokinetics of molnupiravir in cats with naturally occurring FIP by measuring MPV and EIDD‐193 (β‐D‐N4‐hydroxycytidine; NHC) serum levels.

**Animals:** Seven cats diagnosed with naturally occurring FIP treated with MPV.

**Methods:** Blood was collected at 1, 2, 4, 6 and 12 h post oral MPV administration and at 12 h post‐pill administration 7 days later. Serum concentrations of MPV and NHC were determined using a previously published high performance liquid chromatography tandem‐mass spectrometry (HPLC‐MS/MS) method.

**Results:** The mean dose of MPV was 15.44 mg/kg (SD±1.82). The mean peak serum concentration of MPV (Cmax) after a single PO dose of MPV was 6.0 μM (SD±2.8). The mean peak serum concentration of NHC (Cmax) after a single PO dose of MVP was 1551 ng/mL (SD±720). Time to reach Cmax (Tmax) was 2.6 h (SD±1.4); and elimination half‐life was 1.6 h (SD±1.1). No accumulation of drug was seen in trough levels following daily dosing for 7 days. Combined serum MPV and NHC levels for all cats at 1, 2, 4, 6 and 12 h post MPV administration are shown in Figures 1 and 2.

**Conclusions and Clinical Importance:** This study supports the use of MPV in the treatment of FIP. The low MPV levels may be explained by fast conversion to its active metabolite NHC.

**Figure:**
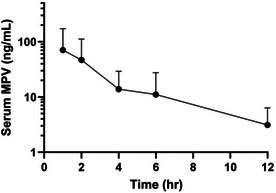
**Figure 1** Combined serum MPV levels in all cats at 1, 2, 4, 6 and 12 h post MPV administration.

**Figure:**
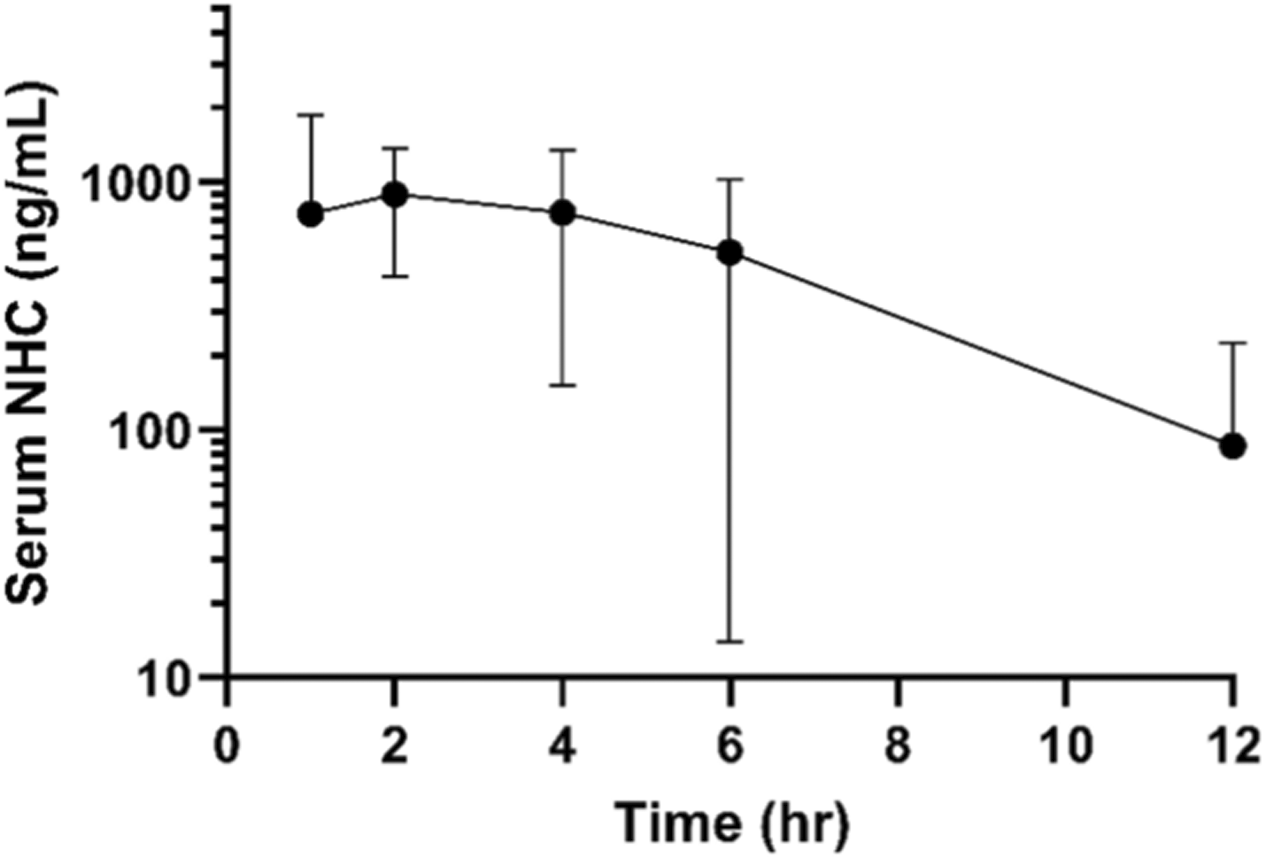
**Figure 2** Combined serum NHC levels in all cats at 1, 2, 4, 6 and 12 h post MPV administration. The mean NHC concentrations at all time points were at least 4 times the reported *in vitro* IC50 for feline coronavirus strains.

## 197 ABSTRACT ID12: Does Virulence Vary Amongst ‘*Candidatus* Mycoplasma haemominutum’ Isolates Within a Geographical Region?

### 197.1 **Keisuke Konishi** ^1^; Jennifer Hawley^2^; Michael Lappin^3^, DVM, PhD, DACVIM (SAIM); Valeria Scorza^4^, DVM, PhD

#### 197.1.1 ^1^Veterinarian, MS Student, Colorado State University; ^2^Research Associate, Colorado State University; ^3^Professor, Colorado State University; ^4^Assistant Professor, Colorado State University

**BACKGROUND:** ‘*Candidatus* Mycoplasma haemominutum’ (CMh) is the most common feline hemoplasma but is generally considered a non‐pathogen. However, in some clinically affected cats, CMh appears to be a primary pathogen.

**Hypothesis/Objectives:** Different strains of CMh exist within regions that vary in virulence.

**Animals:** Stored EDTA blood samples submitted to our laboratory that had been stored at −80°C until assayed in this study. The samples came from healthy cats being screened as blood donors or from clinically ill cats with fever or anemia.

**Methods:** Samples were assessed for DNA of the 3 feline hemoplasmas by PCR assay that targets the 16S rRNA gene. Samples positive for CMh and no other pathogens were sequenced and the data were compared by BLAST analysis. Phylogenetic analysis was performed in Geneious Prime and exported into MEGA 11 for phylogenetic construction. Clinical histories were determined by review of the medical record.

**Results:** Positive results were obtained for 6 healthy cats and 9 clinically ill cats and sequences showing 98–100% homology to other CMh reported in GenBank. When clinical histories were evaluated, 4 of the 9 cats had findings suggesting that CMh was a primary pathogen. However, clustering differences amongst healthy cats and cats with histories suggesting different CMh manifestations were not apparent.

**Conclusions and Clinical Importance:** Clinical findings potentially associated with CMh cases assessed here suggest that virulence can vary amongst strains in a region. Phylogenetic analysis being repeated with a longer segment of the 23S gene.

## 198 ABSTRACT ID13: Comparison of Two In‐House Screening Tests for *Anaplasma* and *Ehrlichia* spp. Antibody Detection in Dogs

### 198.1 **Meera Surendran Nair** ^1^, DVM, MS, PhD, DACVM; Chinta Lamichlane^2^; Xiuli Yang^3^; Haichen Song^4^; Irina Sokolchik^5^; Michelle Evason^6^; Graham Bilbrough^7^; Pablo Jimenez Castro^8^; Jennifer Willcox^9^; Christian Leutenegger^10^

#### 198.1.1 ^1^Director, Infectious Disease, Antech/MARS; ^2^Senior Vice President, Laboratory Technologies Department, Antech/MARS; ^3^Senior Manager, Manufacturing Operations, Antech/MARS; ^4^Senior Manager, R&D and Quality Control, Antech/MARS; ^5^Senior Manager, Immunology, Antech/MARS; ^6^Global Director, Veterinary Clinical Education, Antech/MARS; ^7^Director, Clinical Expertise and Medical Marketing Support, Antech/MARS; ^8^Director, Parasitology, Antech/MARS; ^9^Director, Clinical Studies, Antech/MARS; ^10^Vice President, R&D Reference Assay Development, Antech/MARS

**Background:** Veterinary surveillance for *Anaplasma* and *Ehrlichia* spp. in dogs is essential to assess human exposure risk. Annual testing is recommended in endemic regions of North America.

**Objective:** Compare the performance of screening tests for *Anaplasma* and *Ehrlichia* spp. antibodies in canine serum, plasma, or whole blood to an indirect fluorescent antibody test (IFA‐REFERENCE) for *A. phagocytophilum*, *A. platys*, *E. canis*, *E. chaffeensis*, a species‐specific ELISA test for *E. ewingii*, and a commercial in‐hospital test (SNAP® 4Dx® Plus, IDEXX).

**Animals:** Anonymized (IFA‐characterized) remnant serum samples (*n* = 786; *Anaplasma* spp. *n* = 358, *Ehrlichia* spp. *n* = 428) were obtained from veterinary reference laboratories and included client‐owned dogs.

**Methods:** Samples were processed (*Anaplasma* spp. *n* = 220, *Ehrlichia* spp. *n* = 200) using the following tests to evaluate the performance metrics: 1. trūRapid FOUR® (Antech Diagnostics, Mars Petcare Science and Diagnostics, 3. IFA (REFERENCE), and 4. species‐specific in‐house ELISA test for *E. ewingii*. Kappa statistics was calculated to evaluate test agreement between trūRapid FOUR® and SNAP 4Dx® (*Anaplasma* spp. *n* = 138, *Ehrlichia* spp. *n* = 228).

**Results:** Table 1 shows performance metrics for trūRapid FOUR® using REFERENCE as standard and Kappa agreement between trūRapid FOUR® and SNAP 4Dx®. The lower agreement for trūRapid FOUR® in *Ehrlichia* testing with SNAP 4Dx® is due to SNAP 4Dx's low specificity observed compared to REFERENCE test.

**Clinical Importance:** The trūRapid FOUR® is an accurate option for in‐house *Anaplasma* and *Ehrlichia* spp. antibody detection in dogs. This work offers veterinary teams essential clinical information on tick‐borne pathogen diagnostics to support One Health.

**Table:** **Table 1a.** Performance metrics (specificity and sensitivity) compared to the REFERENCE tests

Species‐specific REFERENCE tests	trūRapid FOUR® *Anaplasma* spp. antibody test (95% confidence interval)	trūRapid FOUR® *Ehrlichia* spp. antibody test (95% confidence interval)
Sensitivity	96.5% (91.3%–99.0%)	93.5% (87.0%–97.3%)
Specificity	98.1% (93.4%–99.8%)	98.9% (94.2%–100.0%)

**Table:** **Table 1b.** Agreement (kappa) between trūRapid FOUR® and SNAP 4Dx® were evaluated

**Kappa statistics—trūRapid FOUR® vs SNAP 4Dx®**	*Anaplasma* spp.—98.2% (95% CL 94.68% to 100%)	*Ehrlichia* spp.—91.06% (95% CL 85.64% to 96.47%)

## 199 ABSTRACT ID14: Field Validation of an *Anaplasma* and *Ehrlichia* spp. Antibody Screening Test in Dogs

### 199.1 **Irina Sokolchik** ^1^; Christian Leutenegger^2^; Isabelle Caya^2^; Michelle Evason^2^; Pablo David Jimenez Castro^2^; Jere McBride^3^; Christian Savard^2^

#### 199.1.1 ^1^Senior Manager, Immunology R&D, Antech Diagnostics/Mars Science and Diagnostics; ^2^Antech/MARS; ^3^UTMB

**Background:** Antibody testing for tick‐borne pathogens is the main screening method used by veterinarians in dogs. With the expanding range and abundance of tick vectors, and the incursion of new tick‐borne pathogen species and strains into the United States (U.S.) and Canada, screening tests need to be continuously updated and evaluated for performance.

**Objectives:** To perform a field validation of updated *Anaplasma* and *Ehrlichia* spp. peptides on a commercially available silicon surface multiplex immunoassay (SSMIA) screening platform (Accuplex®, Antech Diagnostics, Inc., Mars Petcare Science and Diagnostics), and compare SSMIA to public prevalence data obtained from the Companion Animal Parasite Council (CAPC) website.

**Animals:** A total of 2,031 anonymized canine remnant serum samples from five Northeastern U.S. states, submitted to a veterinary reference laboratory, were evaluated.

**Methods:** CAPC data sets from the capcvet.org website for 5 U.S. states were obtained, and these pathogen prevalence results compared to samples evaluated by the SSMIA platform with updated peptides detecting antibodies for *A. phagocytophilum*, *A. platys* and *E. canis*, *E. chaffeensis*, and *E. ewingii* from the same five U.S. states and time frame.

**Results:** The updated SSMIA platform detected higher proportion positives for *Anaplasma* and *Ehrlichia* spp. as compared to CAPC data (Table 1).

**Conclusions:** The SSMIA screening platform with updated *Anaplasma* and *Ehrlichia* spp. peptides detected higher tick‐borne pathogen antibodies in dogs as compared to those reported through CAPC. These results highlight the need for continuous performance evaluation, and field validation, of screening tests, to reflect current tick‐borne pathogens in the U.S. and Canada.

**Table:** **Table 1**.

State	*Anaplasma* spp.			*Ehrlichia* spp.		
	SSMIA	CAPC	Difference	SSMIA	CAPC	Difference
Pennslyvania	16.1%	13.2%	3.0%	2.6%	1.1%	1.6%
New York	20.7%	15.8%	4.9%	4.3%	1.5%	2.8%
New Jersey	13.8%	10.9%	2.9%	5.3%	3.7%	1.6%
Massachusetts	26.1%	23.7%	2.4%	4.3%	1.9%	2.4%
Connecticut	24.8%	27.1%	−7.1%	4.3%	1.7%	2.6%
**Average**	20.1%	18.1%	**2.16%**	4.2%	2.0%	**2.19%**

Field validation of an updated canine screening platform for tick‐borne pathogen antibody detection and comparison to prevalence data obtained from capcvet.org.

## 200 ABSTRACT ID15: *Echinococcus multilocularis* in Three Dogs in Boulder County, Colorado

### 200.1 **Hanna Kiryluk** ^1^; Jennifer Weng^2^; Terry Watrous^3^; G.A. Khalsa^4^; Alison Manchester^2^; Christian Leutenegger^5^; Michael Lappin^2^

#### 200.1.1 ^1^DVM Student, Colorado State University; ^2^Colorado State University; ^3^South Boulder Animal Hospital; ^4^VCA Broadway Animal Hospital and Pet Center; ^5^Antech Diagnostics

**Background:** *Echinococcus multilocularis* is a zoonotic tapeworm with re‐emerging public health significance. Wild canids are the natural definitive hosts, experience subclinical infection from adult worms, and pass eggs in their feces. Intermediate hosts like rodents ingest the eggs, and the larvae migrate from the intestines predominantly to the liver, where multiple cysts develop. Rarely, domestic dogs and people serve as aberrant hosts and develop life‐threatening multiple hepatic cysts (alveolar echinococcosis).

**Objectives:** To describe the clinical findings associated with *E. multilocularis* in client‐owned dogs on the front range of Colorado.

**Animals:** Three domestic dogs living in Boulder County, Colorado in 2023 and 2024.

**Methods:** *Echinococcus multilocularis* DNA was amplified by a commercially available PCR (KeyScreen; Antech Diagnostics). The medical records from positive dogs were reviewed and discussed with the respective clinicians.

**Results:** Two dogs with mild diarrhea were positive (confirmed) for *E. multilocularis* DNA in feces once. These dogs were negative one week after praziquantel administration and there was no evidence of systemic illness. The third dog has multiple hepatic masses/cysts that have been present for over a year; fine‐needle aspirates of the liver confirmed the presence of *E. multilocularis* DNA. This dog was negative for the parasite in feces, alveolar echinococcosis was diagnosed, and the dog is under long‐term fenbendazole treatment. To our knowledge, no human family members have been infected.

**Conclusions and Clinical Importance:** The zoonotic nature of this parasite's life cycle and frequent contact between humans and domestic dogs highlight the public health relevance of these findings.

## 201 ABSTRACT ID16: Plasma and Complement Proteins Are Essential for the Antimicrobial Activity of Canine Platelet Lysate

### 201.1 **Melika Sadat Mollabashi** ^1^; Alonza Klopfer^2^; Thaina Lunardon^3^; Matt Murray^3^; Nikolia Darzenta^3^; Emily Davis^3^; Scarlett Sumner^3^; Maria Naskou^3^

#### 201.1.1 ^1^Graduate Student, College of Veterinary Medicine, Auburn University; ^2^Auburn University; ^3^College of Veterinary Medicine, Auburn University

**Background:** Platelets play an important role during the inflammatory phase of wound healing due to the release of growth factors. They also have antimicrobial peptides which contribute to their antimicrobial properties. These features make platelet‐derived products suitable biological therapies specifically against bacterial infections.

**Hypothesis:** Canine platelet lysate will display antimicrobial impacts against common bacteria found in canine skin wounds, while the leukocyte concentration, presence of plasma and complement will affect this antimicrobial activity.

**Animals:** Blood was collected from nine purpose‐bred dogs.

**Methods:** Platelet‐rich plasma was generated using both leukocyte‐reduced and leukocyte‐rich centrifugation methods, while a portion of the formulations were depleted from plasma while others underwent complement inactivation via heat. The antimicrobial activity of the above formulation was tested against (ADD bacteria) using a bacteria spiking assay.

**Results:** After 3 h, platelet lysate showed a statistically significant reduction in *E. coli* and *S. aureus* bacteria cultures. No significant differences were observed between leukocyte‐rich and leukocyte‐reduced lysate formulation. Plasma depletion increased the growth of *S. pseud* but reduced the growth of *E. faecalis* after 3 h. In comparison to platelet lysate, *E. faecalis* growth was significantly higher due to heat‐induced deactivation of plasma complement proteins after 24 h.

**Conclusions and Clinical Importance:** Complement proteins and plasma are critical for inhibiting the growth of some bacterial strains, although leukocyte concentration does not appear to affect this activity. These findings highlight the potential of platelet lysate to be used as an antibiotic substitute or supplement for clinical use.

## 202 ABSTRACT ID17: *Giardia Duodenalis* in U.S. Dogs and Cats: Differences in Positive Proportion and Zoonotic Potential

### 202.1 **Pablo Jimenez Castro** ^1^, DVM, PhD, DACVM (Parasitology); Michelle Evason^2^, DVM, DACVIM, MRCVS; Christian Leutenegger^3^; Heather Martinez^3^; Frances Moore^3^; Haresh Rochani^3^; Jennifer Wilcox^3^

#### 202.1.1 ^1^Director of Parasitology, Antech Diagnostics; ^2^Global Director of Veterinary Clinical Education, Antech Diagnostics; ^3^Antech Diagnostics

**Background:** *Giardia duodenalis* is the most frequently reported protozoan of dogs and cats and can have zoonotic potential. Molecular diagnostics have increased sensitivity for detecting parasites as compared to traditional fecal tests.

**Objectives:** To evaluate dog and cat positive proportion, relative risk, and adjusted odds ratio for demographic categories (age, sex, and geographic location), for *G. duodenalis* and *Giardia* with zoonotic potential (assemblages A/B).

**Animals:** Anonymized dog and cat fecal samples (*n* = 1,768,322) from the United States submitted to a reference laboratory for KeyScreen® GI Parasite PCR panel testing between March 2022 and December 2023.

**Methods:** Samples were evaluated by qPCR detecting *G. duodenalis* and *G. duodenalis* with zoonotic potential (assemblages A/B). Logistic regression was performed to investigate strength and direction of the adjusted odds ratio (AOR) between independent and dependent variables.

**Results:** The positive proportion of *G. duodenalis* and *Giardia* with zoonotic potential was 13.15% and 1.67% for dogs and 6.96% and 17.92% for cats, respectively. Demographic categories were described (Table). Dogs had a higher likelihood of *G. duodenalis* detection as compared to cats (AOR=3.12, 95% CI: 2.86, 3.42). Cats were more likely than dogs to have zoonotic potential *Giardia* detected (AOR=1.96 95% CI: 1.47, 2.61).

**Clinical Importance:** Our work provides needed clinical insights on *G. duodenalis* and *Giardia* with zoonotic potential, positive proportions and demographic characterizations. These findings highlight detection differences between cats and dogs, particularly for *Giardia* with zoonotic potential, that will assist veterinarians with management, and guide One Health communications.

**Figure:**
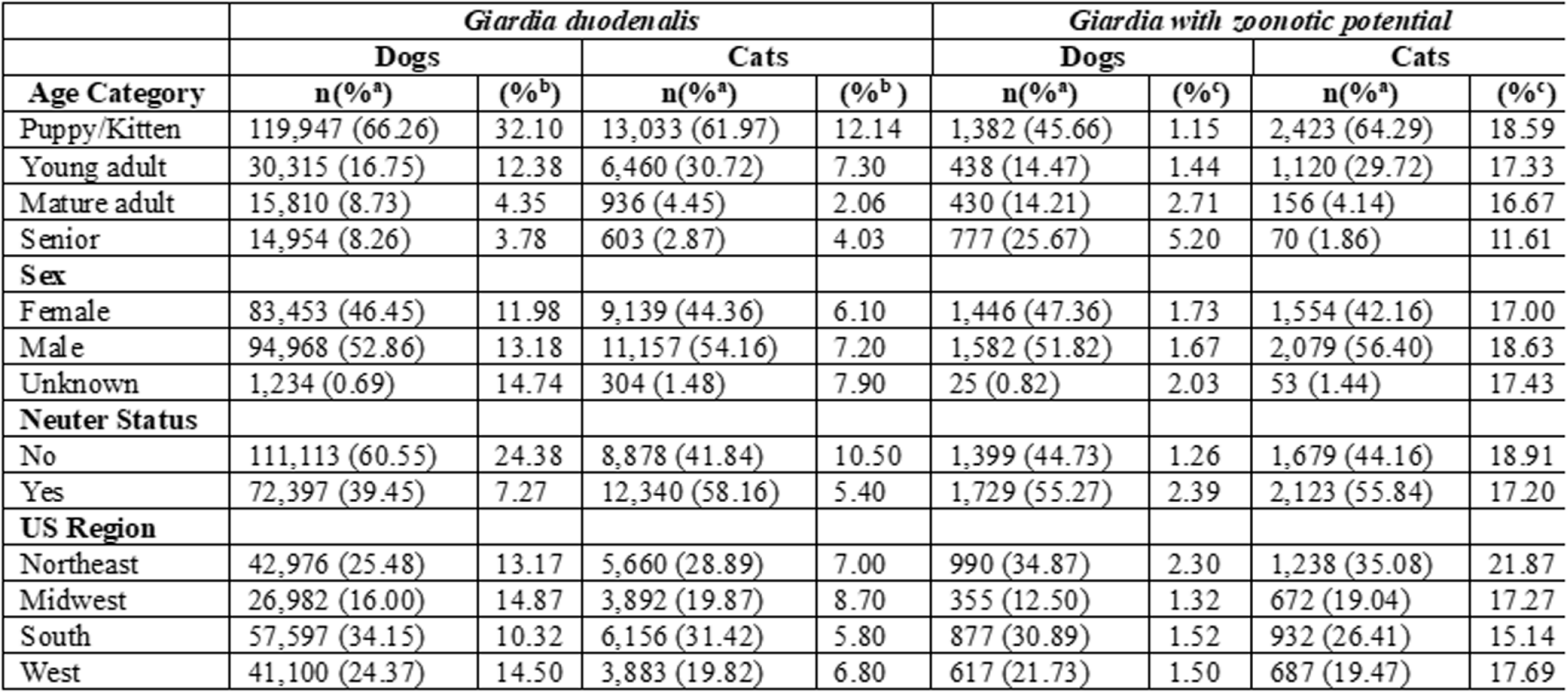
**Table legend. Distribution of U.S. *G. duodenalis* and *Giardia* with zoonotic potential in dogs and cats by demographic categories**. ^a^ % of cases relative to the total number of submissions for each respective category. ^b^ % of the specific age category, sex, neuter status, and region for *Giardia duodenalis* out of the total number of submissions in that category. ^c^ % of the specific age category, sex, neuter status, and region of *Giardia* with zoonotic potential out of the total number of *G. duodenalis* within that category.

## 203 ABSTRACT ID18: Identifying Dogs at Risk of Antimicrobial‐Resistant Infections with Machine Learning: *Escherichia coli* Bacteriuria in Dogs

### 203.1 **Peyton Wagner** ^1^; Krystle Reagan^2^; Tessa LeCuyer^3^; Xinyi Cen^2^; Amanda Gonzalez^4^; Hubert Ngyuen^2^

#### 203.1.1 ^1^Student, University of California–Davis, Davis, CA, USA; ^2^Department of Veterinary Medicine and Epidemiology, University of California–Davis, Davis, CA, USA; ^3^Department of Medicine and Pathology, Microbiology, and Immunology, University of California–Davis, Davis, CA, USA; ^4^VCA West Los Angeles Animal Hospital, Los Angeles, CA, USA

**Background:** *Escherichia coli* is the most commonly isolated organism from dog urine cultures. Treatment guidelines advocate for amoxicillin as an empiric first‐line treatment for sporadic cystitis. However, with rising antimicrobial resistance rates, it is critical to identify dogs at high risk for resistance to first‐line antimicrobials so culture and susceptibility can be prioritized.

**OBJECTIVE:** To determine if machine learning tools applied to clinicopathologic features can predict ampicillin‐resistant (*AmpR*) *Escherichia coli* bacteriuria in dogs, a surrogate for amoxicillin resistance.

**Animals:** 538 dogs.

**Methods:** Dogs with *Escherichia coli* bacteriuria and concomitant urinalysis from 2015 to 2021 were included. The *Escherichia coli* isolate was classified as resistant or susceptible to ampicillin based on VET01SCLSI breakpoints. Clinicopathologic features were collected (Table 1). These data were split into 80% training and 20% testing sets. Gradient‐boosted tree machine learning models (MLM) were trained to predict *AmpR*, and performance on the test set was determined.

**Results:** *Escherichia coli* isolates were ampicillin susceptible in 398 and resistant in 120 dogs. The MLM accurately categorized 86.0% isolates in the test set, with a sensitivity of 93.9% (95% CI; 88.2%–99.7%) and specificity of 66.7% (95% CI; 48.9%–84.5%) for detecting *AmpR*. The area under the receiver operating characteristic curve was 0.956 (95% CI; 0.917–0.994). The most contributive features included pyuria, antimicrobial administration in the preceding 30 days, and clarity of the urine.

**Conclusion and Clinical Importance:** MLMs can predict the presence of *AmpR Escherichia coli* bacteriuria with high sensitivity using features available the day of clinical presentation. This may assist veterinarians in therapeutic and diagnostic decision‐making.

**Figure:**
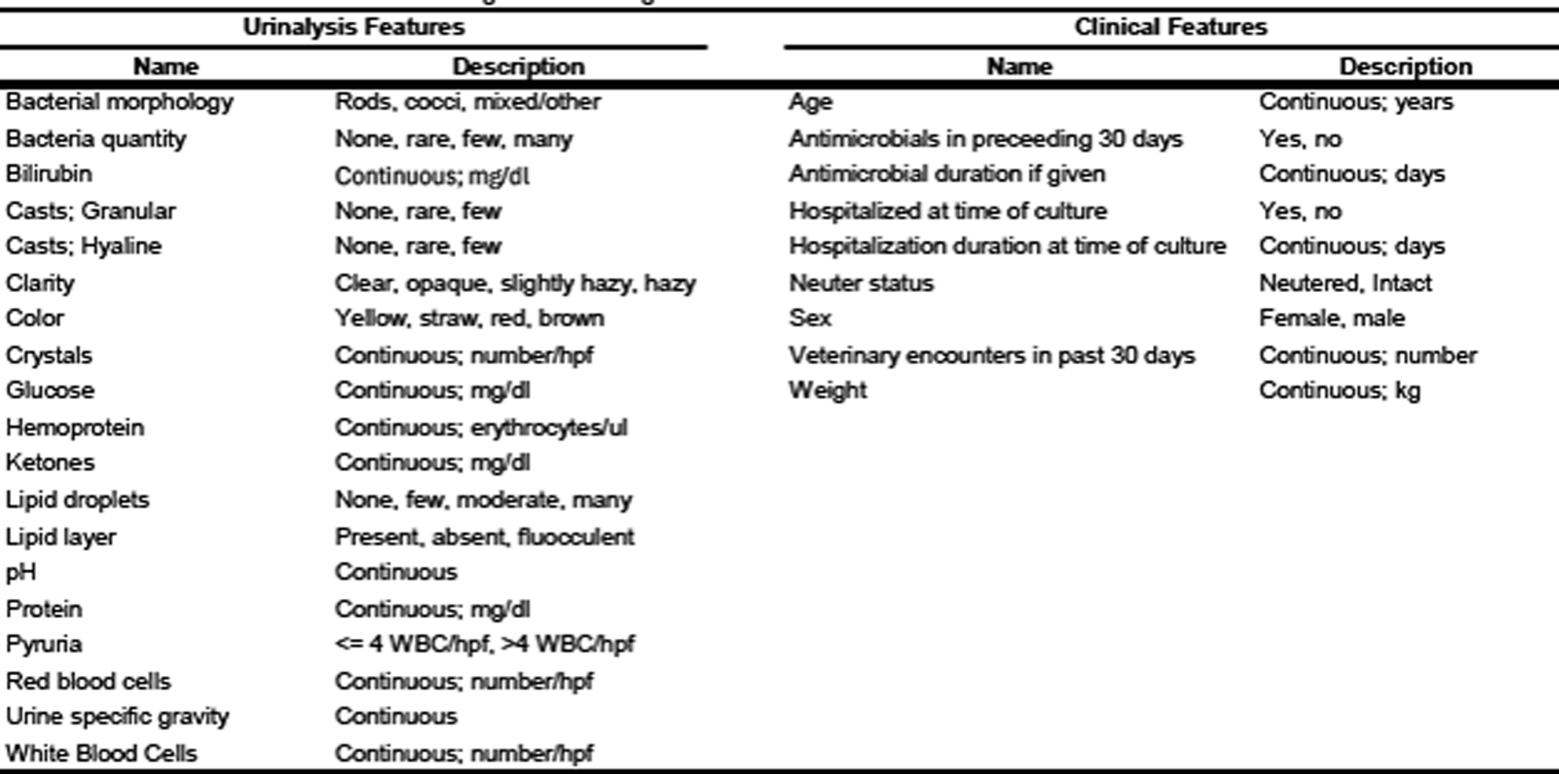
**Table 1. Features utilized in machine learning model training**. Hpf = high power field, WBC = white blood cells

## 204 ABSTRACT ID19: Lungworm Species Typing Using Molecular Diagnostics in an International Sample Cohort

### 204.1 **Pablo Jimenez Castro** ^1^, DVM, PhD, DACVM (Parasitology); Donato Traversa^2^; Michelle Evason^3^; Anastasia Diakou^4^; Elizabeth Redman^5^; John Gilleard^5^; Christian Leutenegger^3^; Samatha Loo^3^; Christian Savard^6^; Jeffrey Tereski^3^

#### 204.1.1 ^1^Director of Parasitology, Antech Diagnostics; ^2^Unite; ^3^Antech Diagnostics; ^4^Aristotle University of Thessaloniki, Thessaloniki, Greece; ^5^University of Calgary, Calgary, AB, Canada; ^6^BioVet

**Background:** Infection with lungworm species may cause clinical disease in dogs and cats. As an example, *Angiostrongylus vasorum* is endemic in Europe, Africa, and South America, and has been reported in Canada and the United States (U.S.). Molecular diagnostics allow the detection and typing of lungworm species, which are difficult to identify by conventional microscopy.

**Objectives:** To validate a 7‐species lungworm molecular diagnostics panel for dogs and cats and inform geographic distribution.

**Animals:** A total of 387 microscopically identified samples from different animals from Italy and Greece were included to determine the clinical performance of the 7‐species molecular lungworm panel. A cohort of 5,765 canine and feline fecal samples submitted for conventional zinc‐sulfate centrifugation or molecular qPCR testing from the U.S. and Canada were tested to identify lungworms.

**Methods:** Total nucleic acid extracts were analyzed using hydrolysis probe‐based real‐time PCR (qPCR) tests for 7 lungworm species and one internal sample control. Canine lungworm species included *A. vasorum*, *Oslerus osleri*/*Filaroides hirthi*, and *Crenosoma vulpis*. Feline lungworm species included *A. chabaudi*, *Aelurostrongylus abstrusus*, *O. rostratus*, and *Troglostrongylus brevior*. Discrepant results were confirmed using next‐generation sequencing methods.

**Results:** When compared to the Baermann test, ZCF, and morphologically identified specimens, the PPA and NPA values were 74% (0.58–0.86) and 99% (0.96–1), 89% (0.79–0.96) and 95% (0.89–0.98), and 100% (0.74–1) and 100% (0.59–1), respectively. Sixty sequence‐confirmed cases were detected in Canada and the U.S. (Figure).

**Conclusions:** Molecular methods for the detection and differentiation of lungworm species of global concern provide efficient and affordable alternatives to conventional identification methods. Accurate identification of lungworm species supports timely clinical management, reduces unnecessary treatments, and mitigates risks associated with misdiagnosis or spurious parasitism.

**Figure:**
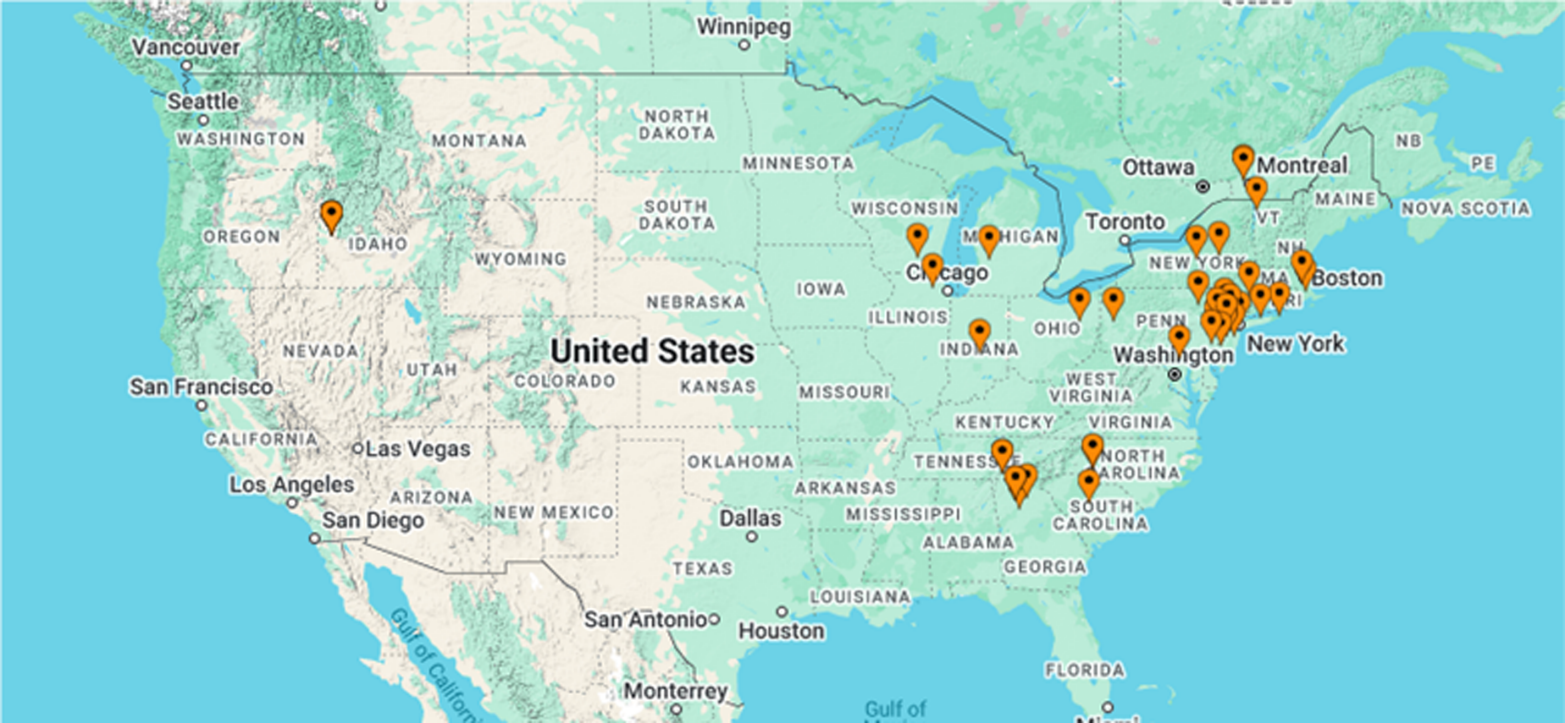
Figure legend. Geographic map of identified and confirmed lungworms in cats and dogs

## 205 ABSTRACT ID20: Machine Learning Tools to Identify Cats With Bacterial Cystitis When Exhibiting Lower Urinary Tract Signs

### 205.1 **Peyton Wagner** ^1^; Xinyi Cen^2^; Bradley Phyo^2^; Krystle Reagan^2^; Savannah Tobin^2^; Jodi Westropp^2^

#### 205.1.1 ^1^Student, University of California Davis, CA, USA; ^2^Department of Veterinary Medicine and Epidemiology, University of California, Davis, CA, USA

**Background:** Feline idiopathic cystitis (FIC) is the leading cause of cats with lower urinary tract signs (LUTS). Although bacterial urinary tract infections (UTI) are less common, antimicrobial therapy is often prescribed. Methods to identify cats with a high likelihood of UTI may improve antimicrobial stewardship.

**Hypothesis:** Machine learning models (MLM) trained with clinical data and urinalysis results can predict which cats with LUTS have a high risk of UTI.

**Animals:** 425 cats.

**Methods:** Records from cats with LUTS and FIC or UTI from 2009–2020 were reviewed; urinalysis and contemporaneous urinary culture were recorded. Cats with urolithiasis, masses, or other causes for LUTS were excluded. These data were split into 80% training and 20% test sets. Body weight, age, sex, and urinalysis results were used to train a gradient‐boosted tree MLM, and feature importance was determined. The performance statistics from the test set are presented.

**Results:** 230 cats with FIC and 195 with UTI were identified. The MLM had a sensitivity of 84.8% (95% confidence interval [CI]; 74.4%–94.0%) and specificity of 81.8% (95% CI; 67.6%–93.9%) for predicting UTI. The area under the receiver operating characteristic curve was 0.885 (95% CI; 0.802–0.955). The positive predictive value was 82.3% (95% CI; 66.5%–93.9%), and the positive likelihood ratio was 4.6. The top contributing features to the MLM were urine white blood cell concentration, specific gravity, and age.

**Conclusion and Clinical Importance:** MLMs can help identify cats at high risk of UTI when exhibiting LUTS and may aid in clinical decision‐making.

## 206 ABSTRACT ID21: *Baylisascaris procyonis* (Raccoon Roundworm) Detection in Dogs and Cats From the United States and Canada

### 206.1 **Emily Teng** ^1^, DVM; Pablo Jimenez Castro^2^; Marie Kerl^3^; Anne Kimmerlein^2^; Jeffrey Tereski^2^; Samantha Loo^2^; Jennifer Wilcox^2^; Michelle Evason^2^; Christian Leutenegger^2^

#### 206.1.1 ^1^Internal Medicine Resident, VCA; ^2^Antech; ^3^Mars

**Background:** *Baylisascaris procyonis* is a zoonotic roundworm. Clinical disease, occasionally fatal, has been reported in dogs and humans. There are limited studies on the epidemiology or clinical features of *Baylisascaris procyonis* in dogs and cats from the United States (U.S.) and Canada.

**Objectives:** To describe the geographic distribution and clinical features of fecal qPCR‐detected *Baylisascaris procyonis* in dogs and cats.

**Animals:** A total of 74 dogs and 3 cats from the U.S. (*n* = 55) and Canada (*n* = 22) detected with *Baylisascaris procyonis* by the KeyScreen® GI Parasite PCR.

**Methods:** Questionnaires regarding individual dog and cat demographics, clinical signs, and treatment were distributed to the veterinarian who submitted fecal samples with detected *Baylisascaris procyonis* on the KeyScreen® GI Parasite PCR. Descriptive statistics were calculated.

**Results:** Detected *Baylisascaris procyonis* dogs and cats (Figure 1) were reported from the Midwestern (35%) and Northeastern U.S. (22%) and Ontario, Canada (23%). Most U.S. (69%) *Baylisascaris procyonis* detected animals resided in a suburban environment (69%), while the majority (80%) from Canada were reported to reside in an urban environment. Most (62%) animals did not have reported clinical signs; however, 36% had gastrointestinal signs reported. Most treated animals (72.9%) were reported to have responded well to anthelmintic treatment.

**Figure:**
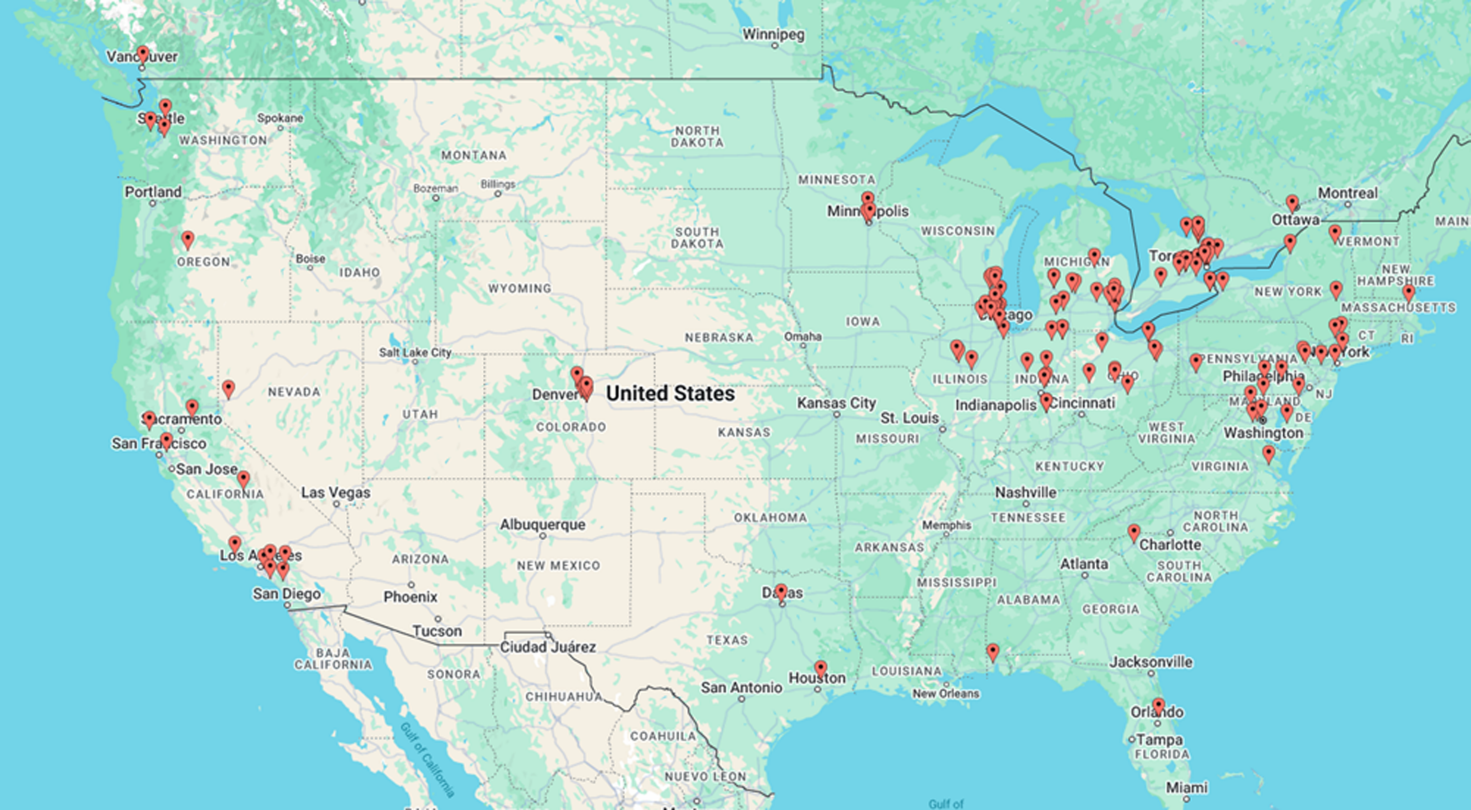
**Figure 1. *Baylisascaris procyonis* detected on KeyScreen® GI Parasite PCR, March 2022–January 2025**. Geographic map of identified and confirmed *Baylisascaris procyonis* in cats and dogs with KeyScreen® GI Parasite PCR test, between March 2022–January 2025, *n* = 155.

**Conclusions and Clinical Importance:** The geographic distribution of detected *Baylisascaris procyonis* in dogs and cats is similar to that reported in raccoons. Our work raises awareness of zoonotic risk, informs clinical management, and provides valuable One Health information.

## 207 ABSTRACT ID22: Assessing Fecal Lipidome in Cats Recovering From Feline Infectious Peritonitis After Antiviral Therapy

### 207.1 **Petra Cerna** ^1^, PhD, DACVIM (SAIM), MANZCVS (Medicine of Cats), CertAVP (SAM‐F), MRCVS, AFHEA, AdvCertFB; Jan Suchodolski^2^; Teresa Schmidt^2^; Jennifer Hawley^3^; Michael R Lappin^3^

#### 207.1.1 ^1^PhD Candidate, Colorado State University, CO, USA; ^2^Texas A&M University, TX, USA; ^3^Colorado State University, CO, USA

**Background:** Gastrointestinal symptoms are reported with SARS‐CoV‐2 due to increased production of inflammatory cytokines in the gut, leading to damage of the mucous membrane barrier. Studies have shown changes in long‐chain fatty acids, sterols, and bile acids in cats with chronic enteropathy, but studies of cats with FIP have not been completed.

**Hypothesis/Objectives:** Determine fecal lipid profiles in cats recovering from feline infectious peritonitis (FIP) after antiviral therapy and compare the results to healthy controls (HC).

**Animals:** Fecal samples from 34 cats without gastrointestinal signs after being treated for 12 weeks with antiviral therapy (EIDD‐2801) for FIP and 21 HC.

**Methods:** Long‐chain fatty acid and sterol profiles were measured in all fecal samples using a previously validated gas chromatography‐mass spectrometry assay. Mann‐Whitney test was utilized for statistical analysis.

**Results:** Cats with FIP had increased concentrations of arachidonic acid (p≤0.001) and nervonic acid (p≤0.001) in feces compared to HC (Figure 1). Those 2 long‐chain fatty acids have been consistently shown to be increased in cats with acute and chronic enteropathy in previous studies, suggesting that the FIP group has alterations in the intestinal epithelium despite not having gastrointestinal signs.

**Figure:**
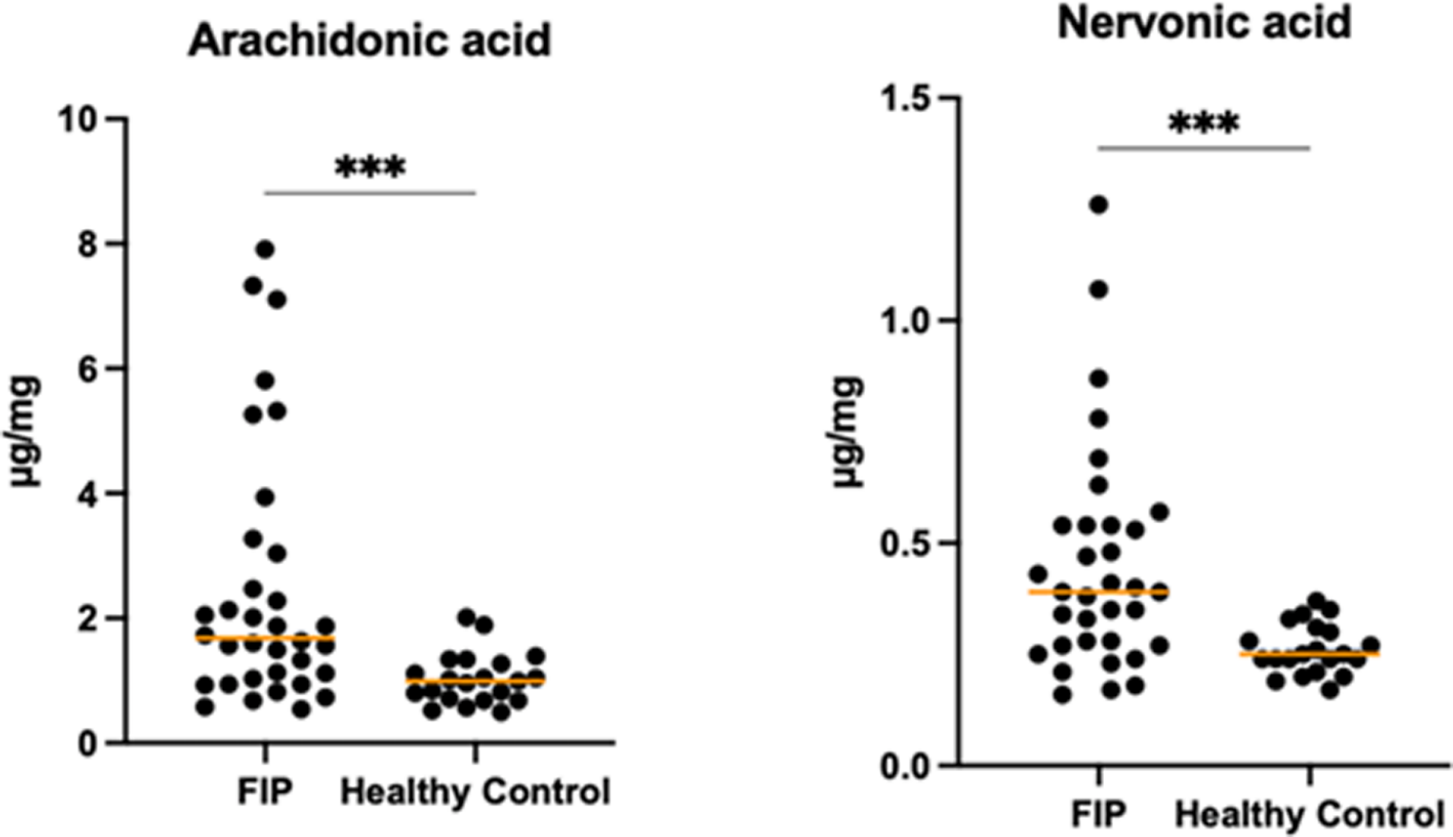
**Figure 1. Fecal concentration of the significant increased fecal concentrations of two long‐chain fatty acids μg/mg in cats with FIP (*n* = 34) and healthy control cats (*n* = 21)**. Orange lines represent the median. ***indicates *P* < 0.001.

**Conclusions and Clinical Importance:** This preliminary data suggests that cats with treated FIP have gastrointestinal alterations in fatty acid metabolism and more studies are needed to see if these are related to the drug or the viral infection and if this can lead to development of gastrointestinal disease in some cats after recovery from FIP.

## 208 ABSTRACT ID23: Development of Direct Whole‐Genome Sequencing of Feline Coronavirus for Understanding Pathogenesis and Drug Resistance

### 208.1 **Izumi Kida** ^1^, DVM; Keita Matsuno^2^, DVM, PhD

#### 208.1.1 ^1^PhD Student, International Institute for Zoonosis Control, Hokkaido University, Japan; ^2^Associate Professor, Division of Risk Analysis and Management, International Institute for Zoonosis Control, Hokkaido University, Japan

**Background:** Feline coronavirus (FCoV) is the causative agent of feline infectious peritonitis (FIP). Despite its clinical significance, the molecular mechanisms underlying FIP pathogenesis remain poorly understood due to the limited availability of complete genomes. Advances in genome sequencing methods of FCoV are, therefore, essential to expand the landscape of viral pathogenicity.

**Objectives:** To develop and validate a protocol for obtaining complete FCoV genomes directly from clinical samples, enabling routine genomic analysis in clinical settings.

**Animals:** Ten client‐owned cats diagnosed with effusive FIP.

**Methods:** Viral RNA extracted from peritoneal and pleural effusions underwent targeted enrichment using multiplex PCR. Sequencing was performed on the Illumina platform. Sequence reads were trimmed, mapped to reference genomes, and assembled into consensus sequences using CLC Genomics Workbench.

**Results:** Nearly complete FCoV genomes with ≥ 80% coverage were successfully obtained from 9 out of 10 clinical samples. Notably, key genes associated with pathogenicity (3c, 7b, and spike) and drug resistance (RdRp) were reliably sequenced.

**Conclusions and Clinical Importance:** We present a robust, clinically applicable protocol for whole‐genome sequencing of FCoV from effusive FIP cases. This method improves the availability of FCoV genome sequences that can be used for clinical diagnosis and offers a valuable tool for advancing our understanding of FIP pathogenesis and tracking drug resistance in FCoV.

## 209 ABSTRACT ID24: Validation of an In‐House Diagnostic Screening Test for *Borrelia burgdorferi* Antibodies in Naturally Exposed Dogs

### 209.1 **Chinta Lamichlane** ^1^, BVSc & AH, PhD; Graham Bilbrough^2^; Michelle Evason^2^; Pablo Jimenez Caastro^2^; Christian Leutenegger^2^; Irina Sokolchik^2^; Meera Surendran‐Nair^2^

#### 209.1.1 ^1^Senior VP, Laboratory Technologies Department, Antech/MARS; ^2^Antech/MARS

**Background:** Routine veterinary serologic screening for *Borrelia* antibodies in dogs is recommended in Lyme endemic regions.

**Objective:** To determine: 1. The performance of trūRapid FOUR® lateral flow immunoassay (trūRapid FOUR®), Antech Diagnostics, Mars Petcare Science & Diagnostics, for detection of C6 antibodies in dogs as compared to an indirect fluorescent antibody (IFA) test, and 2. Agreement between trūRapid FOUR® and a point‐of‐care (POC) test (SNAP® 4Dx® Plus, IDEXX).

**Animals:** Anonymized remnant serum samples submitted to a veterinary reference laboratory for wellness testing from individual client‐owned dogs in the United States.

**Methods:** Operators processed samples (*n* = 270) using all three tests: 1. trūRapid FOUR®, 2. POC, and 3. IFA, following manufacturers’ instructions and blinded to previous results. All samples underwent analysis with trūRapid FOUR® and IFA, while a subset of 128 samples, selected based on sample volume, were also tested with POC. Sensitivity and specificity were calculated for trūRapid FOUR® using IFA as the standard. Kappa was calculated for trūRapid FOUR® and IFA, and trūRapid FOUR® and the POC test.

**Results:** trūRapid FOUR® demonstrated a sensitivity of 99.0% (95% CI: 94.8%–100%) and specificity of 98.2% (95% CI: 94.8%–99.6%) compared to IFA, with a high level of agreement (Kappa) between: 1. trūRapid FOUR® and IFA 0.97 (95% CI: 0.94–0.99) and 2. trūRapid FOUR® and POC 0.97 (95% CI: 0.94–0.99).

**Clinical Importance:** The trūRapid FOUR® is a highly accurate tool for identifying canine antibodies to the C6 peptide of *Borrelia burgdorferi*, supporting its use in veterinary tick‐borne pathogen surveillance.

## 210 ABSTRACT ID25: Performance of a Silicon Surface Multiplex Immunoassay for *Anaplasma* spp. Antibody Detection in Dogs

### 210.1 Graham Bilbrough^1^, MA, VetMB, MRCVS, CertVA; Michelle Evason^2^; Greg Freeman^2^; Christian Leutnegger^2^; Irina Sokolchik^2^; Jennifer Wilcox^2^

#### 210.1.1 ^1^Director, Clinical Expertise, Antech/MARS; ^2^Antech/MARS

**Background:** With the continued expansion of tick vectors and transmitted pathogens across the United States (U.S.) and Canada, effective surveillance for *Anaplasma* spp. (*A. phagocytophilum* and *A. platys*) in dogs is essential. Serology‐based antibody screening remains a reliable and recommended method for identifying exposure to tick‐borne pathogens of One Health significance.

**Objectives:** To evaluate the performance of two commercially available screening tests: 1. A proprietary silicon surface multiplex immunoassay (SSMIA, Accuplex®, Antech Diagnostics, Mars Petcare Science & Diagnostics) and 2. A point‐of‐care (POC) test (SNAP® 4Dx® Plus, IDEXX). Both were compared to indirect fluorescent antibody (REFERENCE) as the standard. POC was used as no validation publications for Lab 4Dx Plus® were available.

**Animals:** Anonymized remnant serum samples from dogs in the U.S. submitted to a veterinary reference laboratory.

**Methods:** Samples (*n* = 310) were processed using SSMIA, POC, and REFERENCE, following manufacturers’ protocols for SNAP and REFERENCE and reference laboratory standard operating procedures for SSMIA. Operators were blinded to previous results. Performance metrics—sensitivity, specificity, and Kappa statistics—were calculated using REFERENCE as the standard.

**Results:** Both SSMIA and POC demonstrated comparable performance metrics (Table).

**Conclusions:** SSMIA is a highly accurate option for detecting *Anaplasma* spp. exposure in dogs, supporting its use in routine veterinary tick‐borne pathogen surveillance. This study provides clinicians with comparative performance data to inform decision‐making and support client communication.

**Table:** 

Indirect fluorescent antibody; REFERENCE	Accuplex®, Antech Diagnostics (95% confidence interval)	SNAP® 4Dx® Plus, IDEXX (95% confidence interval)
Positives	133	122
Negatives	177	188
Total	310	310
Sensitivity	98% (93–99%)	94% (88–97%)
Specificity	96% (92–98%)	99% (97–100%)
Kappa	0.93 (0.89–0.97)	0.94 (0.90–0.98)

Table legend: Comparative performance of two veterinary screening tests for *Anaplasma* spp. antibodies in dogs. (*n* = 310; REFERENCE identified 129 positives [42%] and 181 negatives [58%]).

## 211 ABSTRACT ID26: Effects of Molnupiravir on Clinical Remission and Calicivirus Shedding in Cats With Chronic Gingivostomatitis

### 211.1 **Jean Carlos Alvarado Colon** ^1^, MS; Petra Cerna^2^, DVM, PhD, DACVIM; Christine Cordova^3^, RVT; Jennifer Hawley^4^; Michael Lappin^5^, DVM, PhD, DACVIM

#### 211.1.1 ^1^DVM Student, College of Veterinary Medicine and Biomedical Sciences, Colorado State University, CO, USA; ^2^Veterinary Resident, Small Animal Internal Medicine, Clinical Sciences, College of Veterinary Medicine and Biomedical Sciences, Colorado State University; ^3^The Cat House on The Kings; ^4^Senior Research Assistant, Clinical Sciences, College of Veterinary Medicine and Biomedical Sciences, Colorado State University, CO, USA; ^5^Professor, Clinical Sciences, College of Veterinary Medicine and Biomedical Sciences, Colorado State University, CO, USA

**Background:** Feline chronic gingivostomatitis (FCGS) is a debilitating condition of cats. Feline calicivirus (FCV) is an RNA virus that is overrepresented in affected cats. Current treatment options, such as full‐mouth dental extractions, are costly and only moderately effective. Molnupiravir inhibits replication of RNA viruses and recently has been widely used to treat SARS‐CoV‐2 and feline coronaviruses.

**Hypothesis/Objectives:** The hypotheses were that administration of molnupiravir would decrease FCV shedding and induce clinical improvement in cats with FCGS that had failed full‐mouth dental extractions.

**Animals:** A cat sanctuary provided review board approval to enroll 8 FeLV/FIV‐negative, FCV‐positive cats with FCGS in this pilot.

**Methods:** The cats had no significant findings on a serum biochemical profile and were divided into a treatment group (*n* = 5) and a control group (*n* = 3). Stomatitis scores assigned using a standardized rubric, body weight, oral swabs for FCV PCR, and lesion photographs were collected prior to starting the study and then weekly. The treatment group was administered molnupiravir (10 mg/kg) orally twice daily for 4 weeks.

**Results:** By week 4, improved lesion scores (4 of 5 cats) and decreased FCV shedding (2 of 5 cats) were noted in some of the molnupiravir‐treated cats but none of the untreated cats (0/3). No side effects were noted.

**Conclusion and Clinical Importance:** This molnupiravir protocol shows promise as a noninvasive treatment for FCV‐positive cats with FCGS, potentially improving quality of life. Further research is needed to confirm its efficacy and broader applications in cats with FCV.

## 212 ABSTRACT ID27: Diagnostic Application of Machine Learning Models for Predicting Feline Infectious Peritonitis

### 212.1 **Sarah Eusebi** ^1^; Petra Černá^2^, PhD, DACVIM (SAIM), MANZCVS (Medicine of Cats), CertAVP (SAM‐F), MRCVS, AFHEA, AdvCertFB; Emi Barker^3^, BSc (Hons), BVSc (Hons), PhD, PGCertTLHE, DECVIM (CA), FRCVS, RCVS Specialist in Small Animal Internal Medicine, EBVS® European Veterinary Specialist in Small Animal Internal Medicine; Sally Coggins^4^, BVSc (Hons I), MANZCVS (Medicine of Cats), PhD; Samantha Evans^5^, DVM, PhD, DACVP, DACVM; Laksha Karthikeyan^6^; Christiana Koo^7^; Bradley Phyo^8^; Nile Stolz^9^; Krystle Reagan^10^, DVM, PhD, DACVIM (SAIM)

#### 212.1.1 ^1^DVM Candidate, School of Veterinary Medicine, University of California, Davis, CA, USA; ^2^PhD Candidate, College of Veterinary Medicine and Biomedical Sciences, Colorado State University, CO, USA; ^3^Senior Clinician in Internal Medicine, Clinical Lead in Infectious Diseases, Langford Vets, University of Bristol; ^4^Postdoctoral Research Fellow (Diseases and Treatment of Cats), Sydney School of Veterinary Science, The University of Sydney; ^5^Assistant Professor, Clinical Pathology, College of Veterinary Medicine and Biomedical Sciences, Colorado State University, CO, USA; ^6^Research Data Analyst, Department of Veterinary Medicine and Epidemiology, University of California, Davis, CA, USA; ^7^DVM Candidate, Department of Veterinary Medicine and Epidemiology, School of Veterinary Medicine, University of California; ^8^Research Data Analyst, Department of Veterinary Medicine and Epidemiology, University of California, Davis; ^9^Student Research Assistant, College of Veterinary Medicine and Biomedical Sciences, Colorado State University, CO, USA; ^10^Director and Associate Professor, Department of Veterinary Medicine and Epidemiology, School of Veterinary Medicine, University of California, CA, USA

**Background:** Antemortem diagnosis of feline infectious peritonitis (FIP) often requires diagnostics that may be costly, invasive, and delay curative treatment. Affordable techniques enabling rapid and confident identification of affected cats are essential for timely initiation of antiviral therapy, enhancing cat welfare by minimizing treatment delays.

**Objective:** To develop machine learning models (MLM) trained on clinicopathologic data to predict a diagnosis of FIP.

**Animals:** 133 cats with histopathology and FCoV‐immunohistochemistry confirmed FIP and 133 age‐matched and effusion‐matched control cats suspected of FIP with alternative diagnoses.

**Methods:** A multi‐institutional, retrospective, case‐control study was conducted using records from 2002–2024. Clinical features, normalized hematology, and serum biochemistry were collected. 5‐fold cross‐validation was used to split, train, and test the data to evaluate logistic regression (LR), support vector machine (SVM), decision tree (DT), and random forest (RF) classification models. Model performance statistics on the test were evaluated, and feature importance was determined where possible.

**Results:** MLMs demonstrated strong predictive performance for identifying cats with FIP, with area under the receiver operating curves of 0.809–0.908 (Table 1). The top‐performing model, random forest, achieved a sensitivity of 83.46% (95% confidence interval [CI]; 74.81–88.85%), specificity of 83.46% (95% CI; 73.55–95.43%), and harmonic mean of precision and recall (F1 score) of 0.8355 for predicting FIP. Serum globulin concentration, eosinophil count, and serum bilirubin concentration were consistently the top contributing features for evaluated models.

**Conclusion:** MLMs trained on clinicopathologic data from cats with a high pretest probability of FIP can accurately predict a diagnosis and may aid in clinical decision‐making.

**Table:** **Table 1.** Performance statistics of machine learning models for predicting a diagnosis of feline infectious peritonitis

Model name	Sensitivity	Specificity	AUC
Logistic regression	81.3% (72.5–98.6%)	80.6% (75.1–88.7%)	0.896 (0.856–0.933)
Support vector machine	84.0% (67.6–96.1%)	78.5% (69.7–88.4%)	0.890 (0.864–0.923)
Decision tree	81.3% (70.5–89.6%)	77.8% (69.6–88.4%)	0.809 (0.786–0.836)
Random forest	80.6% (70.6–88.9%)	84.0% (76.6–92.1%)	0.901 (0.849–0.933)

All values presented with a 95% confidence interval in parentheses. AUC = area under the receiver operating characteristic curve.

## 213 ABSTRACT ID28: Resistance Against Allopurinol in Three Dogs Infected With *Leishmania infantum*

### 213.1 **Ingo Schäfer** ^1^, MSc (SA), ECVCP (Resident); Mathieu Faucher^2^; Yaarit Nachum‐Biala^3^; Lluis Ferrer^4^; Marina Carrasco^4^; Alexandra Kehl^1^; Elisabeth Müller^1^; Torsten Naucke^1^; Gad Baneth^3^

#### 213.1.1 ^1^Laboklin GmbH & Co. KG; ^2^Clinique Veterinaire Alliance; ^3^Koret School of Veterinary Medicine, The Hebrew University; ^4^Nano1Health SL

**Background:** Rising numbers of autochthonous infections with *Leishmania infantum* are described in the U.S. Allopurinol is the main drug for long‐term management.

**Hypothesis/Objectives:** Resistance to allopurinol was associated with reduction in S‐adenosylmethionine synthetase (METK) gene copy numbers (CNs) *in vitro*, but knowledge regarding clinical significance is limited.

**Animals**/**Methods:** Samples of 3 dogs diagnosed with leishmaniasis were submitted due to clinical suspicion of allopurinol resistance. All dogs were tested for **METK** gene CNs by PCR.

**Results:** The dogs originated from Israel, Southern France, and Germany (imported from Spain 2 years before). The dogs were treated with allopurinol for 7 (one additional 3‐week course of miltefosine and one‐month course of domperidone), 3.5 (two additional cycles of meglumine antimonate), and 2 years (two additional 4‐week courses of miltefosine). Steroids were applied in 2 dogs shortly before relapsing. Relapses were suspected by reoccurrence of clinical signs and/or results of laboratory workups (mild/moderate anemia (*n* = 3), mild thrombocytopenia (*n* = 2), hyperproteinemia with hyperglobulinemia (*n* = 3), mild azotemia (*n* = *n* = 1), and marked elevation of the c‐reactive protein (*n* = 2)) and were confirmed by positive PCR. Testing for resistance revealed decreased **METK** gene CNs <3 in all dogs.

**Conclusions and Clinical Importance:** Relapse of *L. infantum* infection after treatment with allopurinol for a minimum of 2 years due to resistance was suspected clinically, backed by the reduction in the METK gene CNs and laboratory workup results. Recognizing drug resistance in relapses is crucial for further management. Allopurinol‐resistant *L. infantum* strains represent a great risk for infection of naïve dogs, cats, and humans.

## 214 ABSTRACT IM01: Plasma ADAMTS13 Activity in Dogs With Severe Thrombocytopenia and Presumed Immune Thrombocytopenia

### 214.1 **Sierrah Travis** ^1^; Ashley Wilkinson^2^; Stefanie DeMonaco^2^; Audrey Keebaugh^2^; Timothy Bolton^3^

#### 214.1.1 ^1^Small Animal Internal Medicine Resident, Virginia‐Maryland College of Veterinary Medicine; ^2^Virginia‐Maryland College of Veterinary Medicine; ^3^College of Veterinary Medicine, Purdue University

**Background:** In humans, deficiency of ADAMTS13, a von Willebrand factor cleaving enzyme, is an important cause of thrombocytopenia and difficult to differentiate from immune thrombocytopenia (ITP).

**Hypothesis/Objectives:** Dogs with presumed ITP have reduced ADAMTS13 activity compared to healthy dogs and bleeding assessment scores (DOGiBAT) negatively correlate with ADAMTS13 activity.

**Animals:** Fifteen privately owned dogs with presumed ITP and 40 healthy dogs enrolled in a separate study recruited from a hospital population.

**Methods:** Prospective observational study. Dogs (≥ 1 year of age) with severe thrombocytopenia (≤20,000 platelets/uL) underwent diagnostic evaluation and were categorized as having presumed primary ITP (pITP) or ITP with concurrent disease. All dogs received a DOGiBAT score. Plasma ADAMTS13 activity was assessed using the Diapharma® RUO Technozyme® ADAMTS13 Activity ELISA kit. Wilcoxon rank sum test was used to determine if ADAMTS13 was significantly different in dogs with ITP compared to healthy controls. Spearman's rank correlation rho test was used to determine if ADAMTS13 activity correlates with bleeding scores.

**Results:** Preliminary **Results:** dogs with presumed ITP have reduced plasma ADAMTS13 activity compared to healthy controls (*P* = 0.039). Individually, plasma ADAMTS13 activity in dogs with pITP or ITP with concurrent disease is not different from healthy controls (*P* = 0.428, *P* = 0.095, respectively). ADAMTS13 activity does not correlate with DOGiBAT scores (*P* = 0.069).

**Conclusions and Clinical Importance:** ADAMTS13 deficiency may be a mechanism for thrombocytopenia in some dogs with severe thrombocytopenia that are labeled as having ITP.

## 215 ABSTRACT IM02: Canine Autoimmune Lymphoproliferative Syndrome in Four Sibling Boerboel Puppies

### 215.1 **Linda J. Tong** ^1^; Steven G. Friedenberg^2^; Jonah N. Cullen^2^; Hayden Hamsher^2^; Katie M. Minor^2^; Eva Furrow^2^; William Vernau^3^ Lisa Horgan^4^; Danijela Kocic^5^; Jason Stayt^6^; Adrien Hespel^1^; George Reppas^7^; Richard Malik^8^

#### 215.1.1 ^1^Perth Veterinary Specialists, Osborne Park, WA, Australia; ^2^Department of Veterinary Clinical Sciences, College of Veterinary Medicine, University of Minnesota, St. Paul, MN, USA; ^3^Department of Pathology, Microbiology and Immunology, School of Veterinary Medicine, University of California–Davis, Davis, CA, USA; ^4^Department of Immunology, Royal Prince Alfred Hospital, Camperdown, NSW, Australia; ^5^St. Vincent's Pathology, St. Vincent's Hospital, Darlinghurst, NSW, Australia; ^6^Novavet, Bayswater, WA, Australia; ^7^Vetnostics, Macquarie Park, NSW, Australia; ^8^Centre for Veterinary Education, Sydney School of Veterinary Science, University of Sydney, NSW, Australia

**Background:** Autoimmune lymphoproliferative syndrome (ALPS) is a rare genetic disorder characterized by FAS‐mediated defective lymphocyte apoptosis, and consequent chronic lymphadenomegaly, splenomegaly and autoimmune cytopenias. While documented in humans and British Shorthair cats, ALPS has not previously been reported in dogs.

**Hypothesis/Objectives:** To report the clinical presentation, laboratory findings, treatment and genetic basis of ALPS in a family of Boerboels.

**Animals:** Four affected Boerboel puppies and three unaffected relatives (littermate, dam and sire).

**Methods:** Prospective case series. Medical histories, clinicopathologic findings, imaging results, treatment and outcomes were analyzed. Whole genome sequencing was conducted on two affected puppies. Variants present in the puppies and absent in a database of 3,023 dog genomes were evaluated.

**Results:** Affected puppies presented with lymphadenomegaly, splenomegaly and various cytopenias, including anemia and thrombocytopenia. Lymph node and spleen cytology showed reactive lymphoid hyperplasia with expanded large lymphocytes. Molecular clonality PCR indicated polyclonal lymphoproliferation in lymph nodes and spleen. Lymph node immunocytochemistry and flow cytometry revealed CD3+/CD4−/CD8− (double negative) T‐cell proliferation. Treatment with prednisolone and sirolimus led to significant clinicopathologic improvement in all affected puppies. Whole genome sequencing in two puppies identified a 14‐base pair insertion in exon two of the FAS gene. The variant was absent in the 3,023 database dogs, including 4 Boerboels. Genotyping of relatives is ongoing.

**Conclusions and Clinical Importance:** This report describes a canine homolog of human ALPS and highlights the importance of considering non‐neoplastic lymphoproliferative disorders in young dogs with generalized lymphadenomegaly and splenomegaly.

## 216 ABSTRACT IM03: Antioxidant Effects of Gastric Acid Suppressants on Free Radicals From *Ex Vivo* Dog Neutrophils

### 216.1 **Keerthi Bandaru** ^1^; Angela Bordin^2^; Paula Giaretta^3^; Karin Allenspach^4^; Katie Tolbert^3^; Emily Gould^3^

#### 216.1.1 ^1^PhD Student, GI Lab, Texas A&M University; ^2^College of Veterinary Medicine, Texas A&M University; ^3^GI Lab, Texas A&M University; ^4^College of Veterinary Medicine, University of Georgia

**Background:** Excess free radical production by neutrophils exacerbates tissue damage in GI ulceration. The gastric acid suppressant drugs (ASDs), famotidine and esomeprazole, are commonly used for GI ulcer treatment. These drugs have antioxidant properties in other species.

**Objectives:** Determine if ASDs reduce *in vitro* (2,2‐diphenyl‐1‐picrylhydrazyl [DPPH]) and *ex vivo* (hydrogen peroxide [H_2_O_2_]) free radicals.

**Animals:** Five healthy, client‐owned dogs.

**Methods:** Standard curves (1.25–200 μg/ml of ASDs and vitamin C [positive control]) were used to calculate the inhibitory concentration (IC50; concentration required to scavenge 50% of DPPH) of each compound via spectrofluorometry. Dog neutrophils were treated with famotidine (1, 10, 100 μg/ml), esomeprazole (8, 80, 800 μg/ml), vehicle (saline) or positive (apocynin; free radical scavenger) control, stimulated with phorbol 12‐myristate 13‐acetate, and resultant H_2_O_2_ quantified via relative luminescence units (RLUs). Experiments were repeated a minimum of three times, with triplicate samples per treatment. Normality was assessed (Shapiro‐Wilk), and differences in IC50s or RLUs (mean+standard deviation) between groups analyzed (one way ANOVA, Tukey's multiple comparisons).

**Results:** Both ASDs were equivalent to vitamin C IC50. Only 800 μg/ml esomeprazole significantly reduced H_2_O_2_ RLUs from stimulated neutrophils (53,867+4,957) compared to stimulated, untreated (606,667+81,132) and stimulated, saline‐treated (662,000+28,000) groups (*p* < 0.01 for both). Esomeprazole‐treated neutrophil RLUs were equivalent to that of apocynin.

**Conclusions and Clinical Importance:** While both ASDs showed *in vitro* antioxidant effects, only esomeprazole reduced H_2_O_2_ *ex vivo*. This might be another mechanism by which esomeprazole resolves GI ulceration in dogs, independent of gastric acid suppression.

## 217 ABSTRACT IM04: Defining the Nasal Transcriptome in Cats With Chronic Rhinitis

### 217.1 **Claire Ludwig** ^1^; Lyndah Chow^2^, PhD; Alice Wang^2^, DVM, MS, DACVIM (SAIM); Michael Lappin^2^, DVM, PhD, DACVIM (SAIM); Steven Dow^2^, DVM, PhD, DACVIM (SAIM)

#### 217.1.1 ^1^Small Animal Medicine and Surgery Rotating Intern, Colorado State University; ^2^Colorado State University

**Background:** Chronic rhinitis (CR) is a common and frustrating syndrome in cats, with few broadly effective treatments. At present, little is known regarding nasal immune responses in cats and how they may be altered in animals with CR.

**Objectives:** To help elucidate the pathogenesis of CR in cats, we collected deep nasal swabs and performed full RNA sequencing on collected cells and compared the nasal transcriptomes of healthy cats and cats with CR.

**Animals and Study Design:** Prospective study design, which included 5 healthy cats (no clinical signs) and 3 cats with CR that were evaluated by rhinoscopy and CT imaging to rule out nasal cancer and fungal infection.

**Methods:** Deep nasal swabs were collected under general anesthesia from study animals, with care to avoid nasal bleeding. RNA extracted from nasal swab cells was subjected to full sequencing, using an Illumina platform (Novogene). Sequence files were analyzed using Partek Flow software, including identification of differentially expressed genes and pathway analysis.

**Results:** Transcriptome comparisons revealed 450 significantly upregulated genes in cats with CR and 106 significantly downregulated genes compared to healthy cats. Pathway analysis revealed significant upregulation of pathways associated with innate immunity, including TNF, IFN‐g, IFN‐a and IL‐6 signaling in cats with CR.

**Conclusions and Clinical Importance:** Nasal transcriptome analysis in cats with CR revealed significant upregulation of innate immune pathways associated with responses to viral infections, including interferon pathways. These findings are distinctly different from those of dogs with CR and suggest a possible underlying viral etiology in cats.

**Figure:**
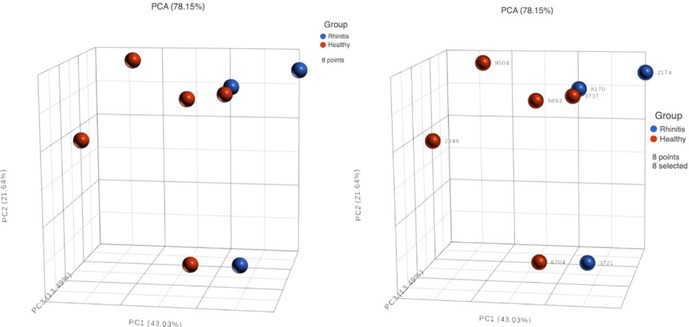
Feline rhinitis results RNA sequencing

**Figure:**
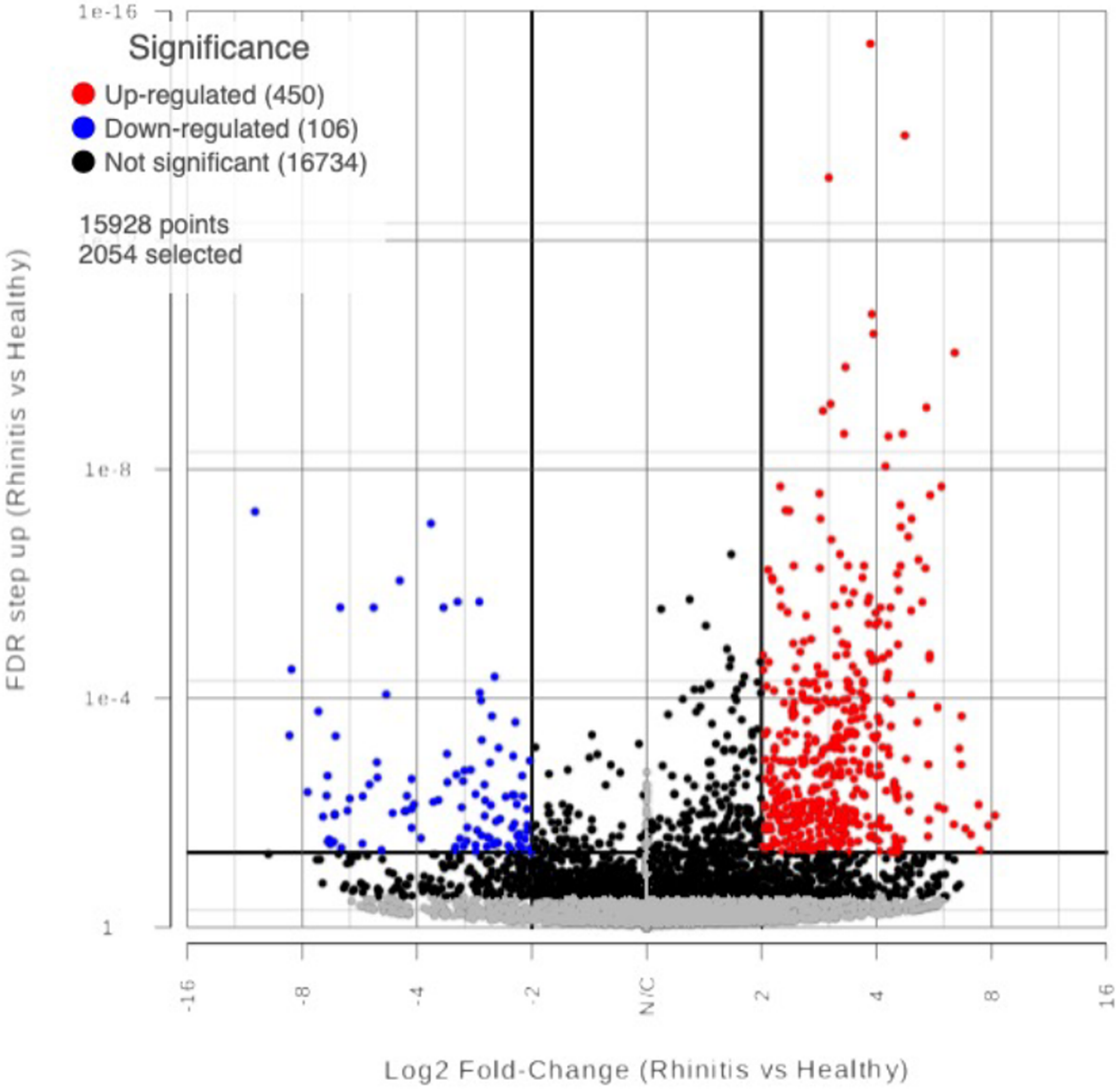
Volcano plot, cat, rhinitis vs. healthy Felis_catus_9.0 Alignment Total ~17000 protein coding genes Differential gene expression analysis (Deseq) Significace FDR <0.05 FC > 2Log2 or <−2Log2 Red=significant upregulated (450) Blue=significant downregulated (106)

**Figure:**
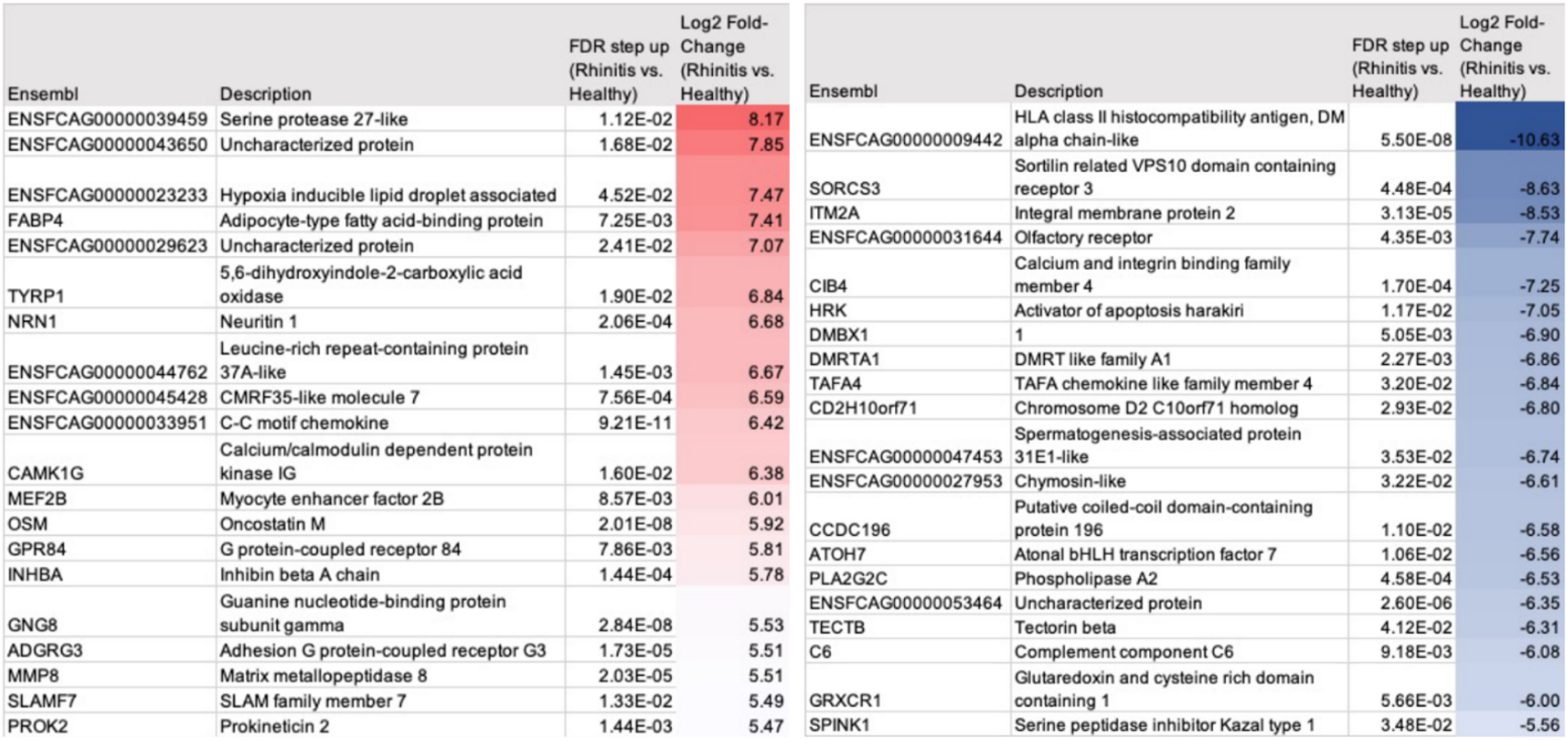
Top 20 up/downregulated genes

**Figure:**
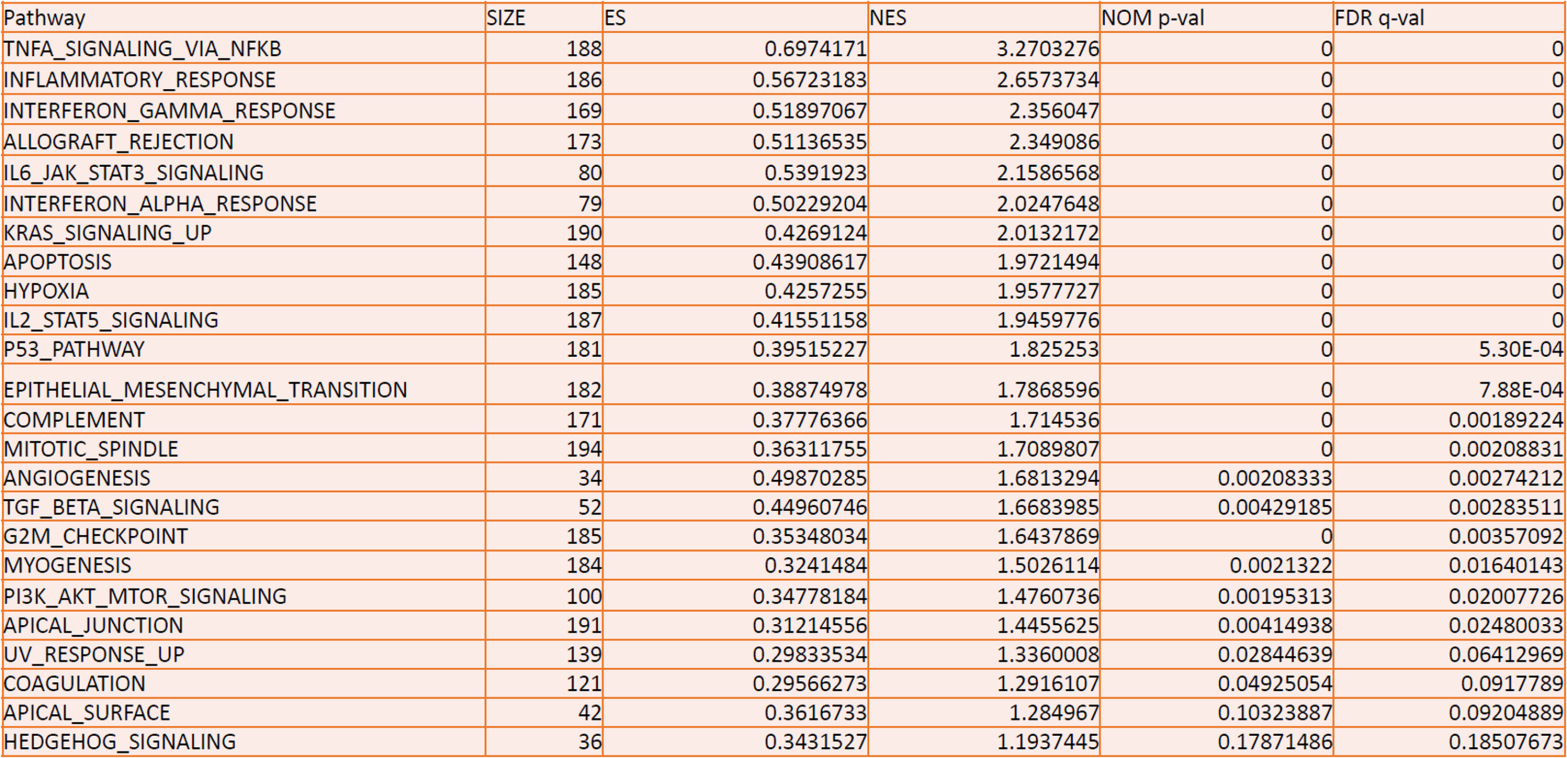
Pathways upregulated in rhinitis

**Figure:**
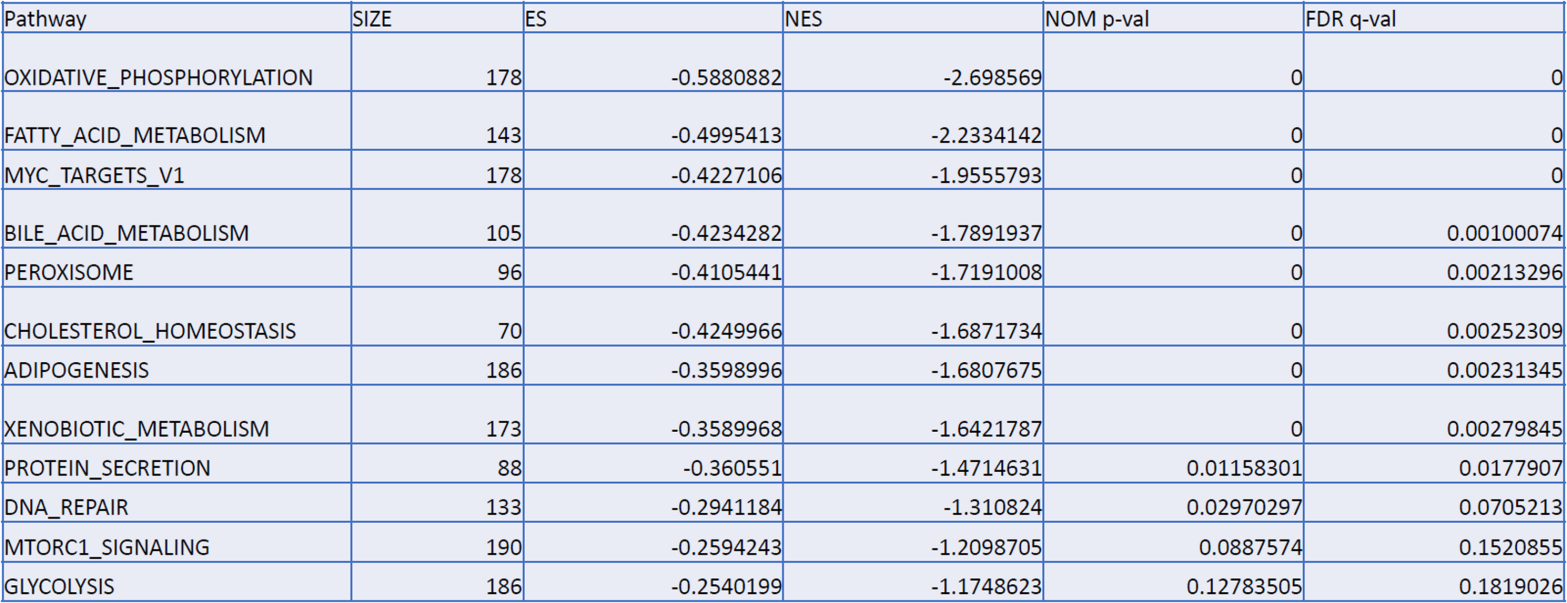
Pathways downregulated in rhinitis

## 218 ABSTRACT IM05: Bulk RNA Sequencing of Blood From Dogs With Immune‐Mediated Polyarthritis Reveals Diverse Inflammatory Pathway Activation

### 218.1 **James Swann** ^1^; Anna Riddle^2^; Samantha Mason^2^; Fergus Allerton^3^; Benjamin Harris^4^; Alenka Hrovat^5^; Andrew Kent^3^; Christopher Scudder^6^; Barbara Glanemann^2^

#### 218.1.1 ^1^Damon Runyon Cancer Research Fellow, Columbia University; ^2^Royal Veterinary College; ^3^Willows Veterinary Centre and Referral Service; ^4^Wear Referrals; ^5^Pride Veterinary Specialists; ^6^Southfields Veterinary Specialists

**Background:** Immune‐mediated polyarthritis (IMPA) causes severe lameness, joint effusions, pyrexia, and lethargy in affected dogs. Clinical features are caused by aberrant immune responses directed at joint antigens, but there is little understanding of the molecular mechanisms driving disease development.

**Hypothesis/Objectives:** To uncover molecular features of the aberrant immune response occurring in dogs with IMPA.

**Animals:** Seventeen client‐owned dogs with primary idiopathic IMPA evaluated at diagnosis and 2 weeks after commencing prednisolone therapy, and 6 healthy controls.

**Methods:** Total RNA was extracted from residual blood samples before bulk library preparation with depletion of ribosomal and hemoglobin transcripts. After sequencing, reads were aligned to the CanFam3.1 reference genome and quantified using Salmon. Differential expression among groups was evaluated with DESeq2 before gene set enrichment analysis and evaluation of transcription factor (TF) activity using the ChEA3 database.

**Results:** At diagnosis, dogs with IMPA had marked upregulation of inflammatory cytokine genes compared to controls (IL1B, IL6, TNF), as well as significant enrichment for pathways related to type I interferon signaling and TNF‐mediated activation of NF‐kB. Moreover, the NF‐kB member RELB was upregulated and predicted to be among the most activated TFs. After treatment, TNF/NF‐kB activity was no longer increased compared to controls, but interferon activity was persistently higher. Additionally, treated dogs showed significant increases in oxidative phosphorylation and reactive oxygen species responses, driven by genes like PRDX2 and ATOX1.

**Conclusions and Clinical Importance:** Dogs with IMPA display dramatic inflammatory responses in peripheral blood, which appear to be incompletely controlled by conventional immunosuppressive therapy.

## 219 ABSTRACT IM06: Enhanced Anti‐Inflammatory Effects of 3D‐Cultured Feline Adipose Tissue‐Derived Mesenchymal Stem Cells

### 219.1 **Sangyoon Choi** ^1^, DVM, MS; Ga‐Hyun Lim^2^; Min‐Ok Ryu^3^; Kyoung‐Won Seo^3^; Hwa‐Young Youn^3^

#### 219.1.1 ^1^Rotating Intern, Ontario Veterinary College, University of Guelph, ON, Canada; ^2^VIP Animal Medical Center; ^3^Seoul National University, South Korea

**Background:** Mesenchymal stem cells (MSCs) exhibit anti‐inflammatory properties in cats. A 3D culture technique has demonstrated enhanced effects compared to conventional 2D cultures.

**Hypothesis/Objectives:** This study aimed to develop a 3D culture method for feline adipose tissue‐derived MSCs (fAT‐MSCs) and to compare their anti‐inflammatory potential with that of 2D‐cultured MSCs.

**Animals:** Feline adipose tissue was collected from healthy spayed and neutered donors at Seoul National University Veterinary Medical Teaching Hospital under an approved protocol.

**Methods:** fAT‐MSC spheroids were generated using an ultra‐low attachment plate. Confocal imaging assessed their structure and hypoxia. Stemness‐related proteins (SOX2, OCT4) were analyzed via immunohistochemistry and western blotting. RT‐PCR evaluated inflammation‐related factors. Conditioned media (CM) from 2D‐ and 3D‐cultured fAT‐MSCs were tested on LPS‐stimulated Raw264.7 macrophages. Inflammatory responses were assessed by flow cytometry and RT‐PCR.

**Results:** Hypoxic conditions in 3D cultures significantly enhanced the expression of stemness‐related proteins (*p* < 0.01). Additionally, inflammation‐related factors, including TGFβ, TNFα, COX‐2, and IL‐10 (*p* < 0.05), were significantly upregulated in 3D‐cultured fAT‐MSC spheroids. CM from 3D cultures significantly reduced M1 polarization in Raw264.7 cells and decreased inflammatory cytokine expression compared to LPS treatment alone.

**Conclusions and Clinical Importance:** This study presents a novel 3D culture method for fAT‐MSCs, demonstrating their superior anti‐inflammatory effects over 2D cultures. These findings suggest that 3D‐cultured fAT‐MSCs may serve as a more effective therapeutic approach for inflammatory diseases in cats.

**Figure:**
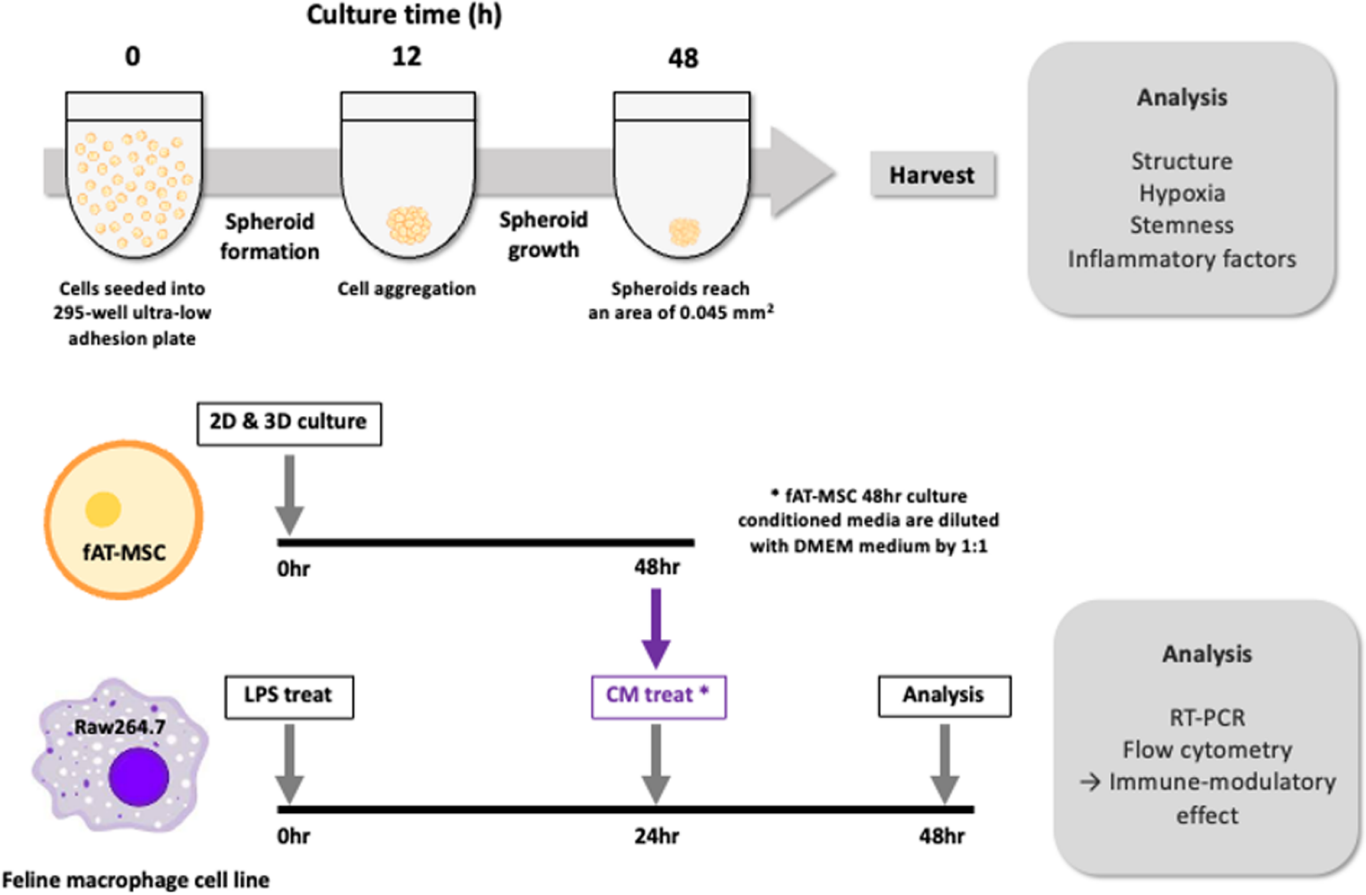
Figure 1

**Figure:**
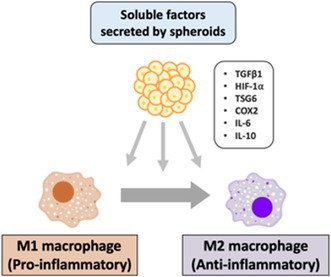
Figure 2

## 220 ABSTRACT IM07: Improved Anti‐Inflammatory Effects of Canine Adipose Tissue‐Derived Mesenchymal Stem Cells Using 3D Culture

### 220.1 **Eunbi Lee** ^1^, DVM; Ga‐Hyun Lim^2^; Min‐Ok Ryu^3^; Kyoung‐Won Seo^3^; Hwa‐Young Youn^3^

#### 220.1.1 ^1^Gradudate, Laboratory of Veterinary Internal Medicine, Department of Veterinary Clinical Science, College of Veterinary Medicine; ^2^VIP Animal Medical Center; ^3^Laboratory of Veterinary Internal Medicine, Department of Veterinary Clinical Science, College of Veterinary Medicine, Seoul National University

**Background:** Mesenchymal stem cells (MSCs) have been utilized in veterinary medicine for their anti‐inflammatory potential. This study explores whether 3D culture improves the anti‐inflammatory effects of canine adipose tissue‐derived MSCs (cAT‐MSCs).

**Hypothesis/Objectives:** This study aimed to optimize the culture conditions for cAT‐MSC spheroids, assess the stemness of the spheroids, and examine the enhancement of anti‐inflammatory factor expression.

**Animals:** Canine adipose tissues were collected from healthy donors that had been spayed and neutered at Seoul National University (SNU) Veterinary Medical Teaching Hospital under a protocol approved by the institutional animal care and use committee of SNU.

**Methods:** cAT‐MSC spheroids were generated using ultra‐low adhesion plates, and their structure and hypoxic conditions were analyzed via confocal imaging. Western blot was used to analyze SOX2 and OCT4 expression. Inflammatory markers were assessed using RT‐PCR and ELISA. Conditioned media from cAT‐MSC spheroids was applied to canine macrophages (DH82), with proinflammatory factor expression and M1 polarization analyzed using RT‐PCR and flow cytometry.

**Results:** 3D culture‐induced hypoxic conditions in cAT‐MSC spheroids and increased their SOX2 and OCT4 expression (*p* < 0.05). Anti‐inflammatory markers, including TGF‐β1, TSG‐6, COX‐2, PGE2, and IL‐10, were elevated in cAT‐MSC spheroids (*p* < 0.05). Conditioned media from cAT‐MSC spheroids reduced expression of proinflammatory factors TNF‐α, IL‐1β, and IL‐6 (*p* < 0.01), and decreased M1 polarization of DH82 cells (*p* < 0.01).

**Conclusions and Clinical Importance:** 3D culture enhances the anti‐inflammatory properties of cAT‐MSCs, suggesting that cAT‐MSC spheroids may provide a more effective therapeutic approach for managing canine inflammatory diseases.

## 221 ABSTRACT IM08: Leptospirosis Vaccine Responses Are Maintained in a Cohort of Alaskan Sled Dogs Aged 8–14

### 221.1 **Lynna Li** ^1^, BSc, MS; John Loftus^2^, PhD, DVM, DACVIM (SAIM, Nutrition)

#### 221.1.1 ^1^College of Veterinary Medicine, Cornell University, NY, USA; ^2^Integrity Veterinary Center

**Background:** Leptospirosis is a zoonotic bacterial disease. Currently, vaccination is the primary method of prevention. Serovars included in vaccines for dogs are Icterohaemorrhagiae, Canicola, Pomona, and Grippotyphosa. The efficacy of this vaccine has not been well‐researched in senior dogs, which may have diminished vaccine responses.

**Hypothesis/Objectives:** The main objective of this study is to use vaccine titers as a marker for amnestic response to understand the relationship between age and immune function within senior dogs.

**Animals:** 103 retired sled dogs (56 males, 47 females) aged 8–11 years old.

**Methods:** Prevaccine blood was collected just before annual 4‐serovar leptospirosis vaccine administration. Post‐vaccine was collected 1 month after vaccination. Leptospira microscopic agglutination test (MAT) titers were performed on sera. Reciprocal titers were compared between age groups and years of vaccine administration using the Kruskal‐Wallis test.

**Results:** There were no significant differences when comparing age groups across all serovars. The titers of different years were also compared to analyze the potential effects of boosters on titer values. Despite significant differences between several‐year comparisons, there was no evident trend seen in any serovar.

**Conclusions and Clinical Importance:** A significant decline in vaccine response within this cohort of senior dogs was not observed. These results suggest that aging in the range assessed may not cause a significant decrease in immune response to the leptospirosis vaccine.

## 222 ABSTRACT IM09: Assessment of NK Cell Activity for Immunity Evaluation in Dogs

### 222.1 **Ga‐Hyun Lim**, DVM, PhD; Ryang‐Hee Kim; So‐Young Jeung; Sung‐Soo Kim

#### 222.1.1 VIP Animal Medical Center

Natural killer (NK) cell activity is crucial for immunity because NK cells can directly eliminate virus‐infected and tumor cells without prior sensitization, providing rapid immune defense. Additionally, they produce cytokines like IFNγ, which enhance adaptive immune responses by promoting macrophage activation and antigen presentation. This study aimed to assess NK cell activity in dogs by measuring plasma IFNγ levels after stimulation with NK cell‐stimulating agent (Promoca, NKMAX). A total of 120 dogs were included, and 1 ml of whole blood was collected from each. The blood was incubated with Promoca at 37°C for 24 h, followed by centrifugation to obtain plasma. IFNγ concentrations were measured using an ELISA kit. The median plasma IFNγ level was 621.8 pg/ml. Healthy dogs exhibited a median level of 1084.9 pg/ml (*n* = 53), whereas diseased dogs showed a lower level of 559.5 pg/ml (*n* = 67). Notably, dogs with tumors had a significantly reduced median IFNγ level of 125.6 pg/ml (*n* = 17). Age also influenced IFNγ levels, with younger dogs (<7 years) showing higher levels (median: 928.9 pg/ml, *n* = 28) compared to older dogs (≥ 7 years, median: 590.25 pg/ml, *n* = 92). These findings suggest that measuring NK cell activity through IFNγ levels may serve as a useful biomarker for canine immune function. The significant differences observed between healthy and diseased dogs, particularly in tumor‐bearing individuals, highlight the potential clinical utility of this test. Further research is needed to validate its application in veterinary medicine.

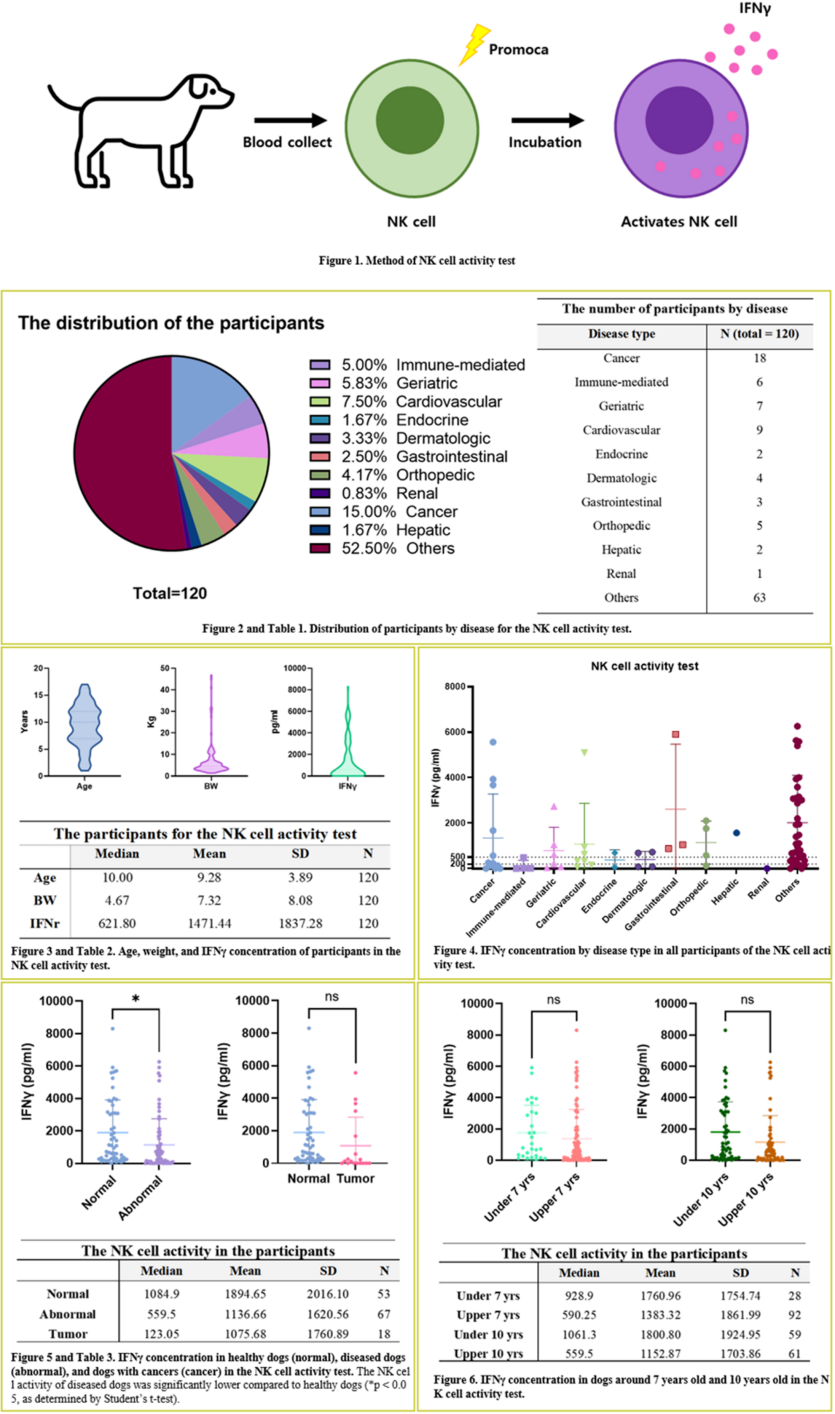

## 223 ABSTRACT IM10: Disease Impact on Prednisolone Pharmacokinetic and Pharmacodynamic Parameters in Dogs with Immune‐Mediated Diseases or Lymphoma

### 223.1 **Kevin Gooch** ^1^, DVM; Beck Mayland^2^, DACVIM (Oncology); Steven Dow^3^, DVM, DACVIM; Daniel Gustafson^3^; Renata Impastato^3^; Jade Kurihara^3^; Chow Lyndah^3^; Sarah Shropshire^3^, DVM, DACVIM; Lana Susan^3^, DVM, DACVIM (Oncology)

#### 223.1.1 ^1^Resident, Small Animal Internal Medicine, Colorado State University, CO, USA; ^2^Southern Colorado Veterinary Internal Medicine; ^3^Colorado State University, CO, USA

**Background:** Prednisolone is used in treating immune‐mediated disease (IMD) and lymphoma (LSA) in dogs. The impact of these two diseases on the pharmacokinetic and pharmacodynamic (PK/PD) parameters of prednisolone in dogs has not been previously investigated.

**Hypothesis/Objectives:** We hypothesized that prednisolone PK parameters would exhibit dose dependence in both diseases, and the impact on modulating immune responses would be greatest in dogs with IMD. Objective 1: Determine prednisolone pharmacokinetics at doses of 1 mg/kg and 2 mg/kg in dogs with IMD and LSA. Objective 2. Assess prednisolone effects on immune transcriptomes and cytokines.

**Animals:** Dogs with IMD (*n* = 10) or lymphoma (*n* = 10) were enrolled to determine prednisolone PK/PD parameters at doses of 1 mg/kg/day and 2 mg/kg/day.

**Methods:** Prednisolone concentrations were determined using standard methodology, leukocyte transcriptome analysis was performed using a Nanostring platform, and plasma cytokines were done by multiplex assay.

**Results:** Prednisolone concentrations exhibited minimal dose dependence in dogs with IMD. There was evidence of decreased absorption in the 1mg/kg LSA group. Transcriptome analysis of circulating leukocytes revealed suppression of the G2M checkpoint and IFN‐α in IMD dogs, and suppression of IFN‐α in LSA dogs.

**Conclusions and Clinical Importance:** Both PK and PD parameters associated with oral prednisolone administration were significantly affected by disease status in dogs. The immune‐modifying activity of prednisolone was also different between dogs with IMD and lymphoma. We conclude that disease status can impact prednisolone absorption in dogs with IMD or LSA.

**Figure:**
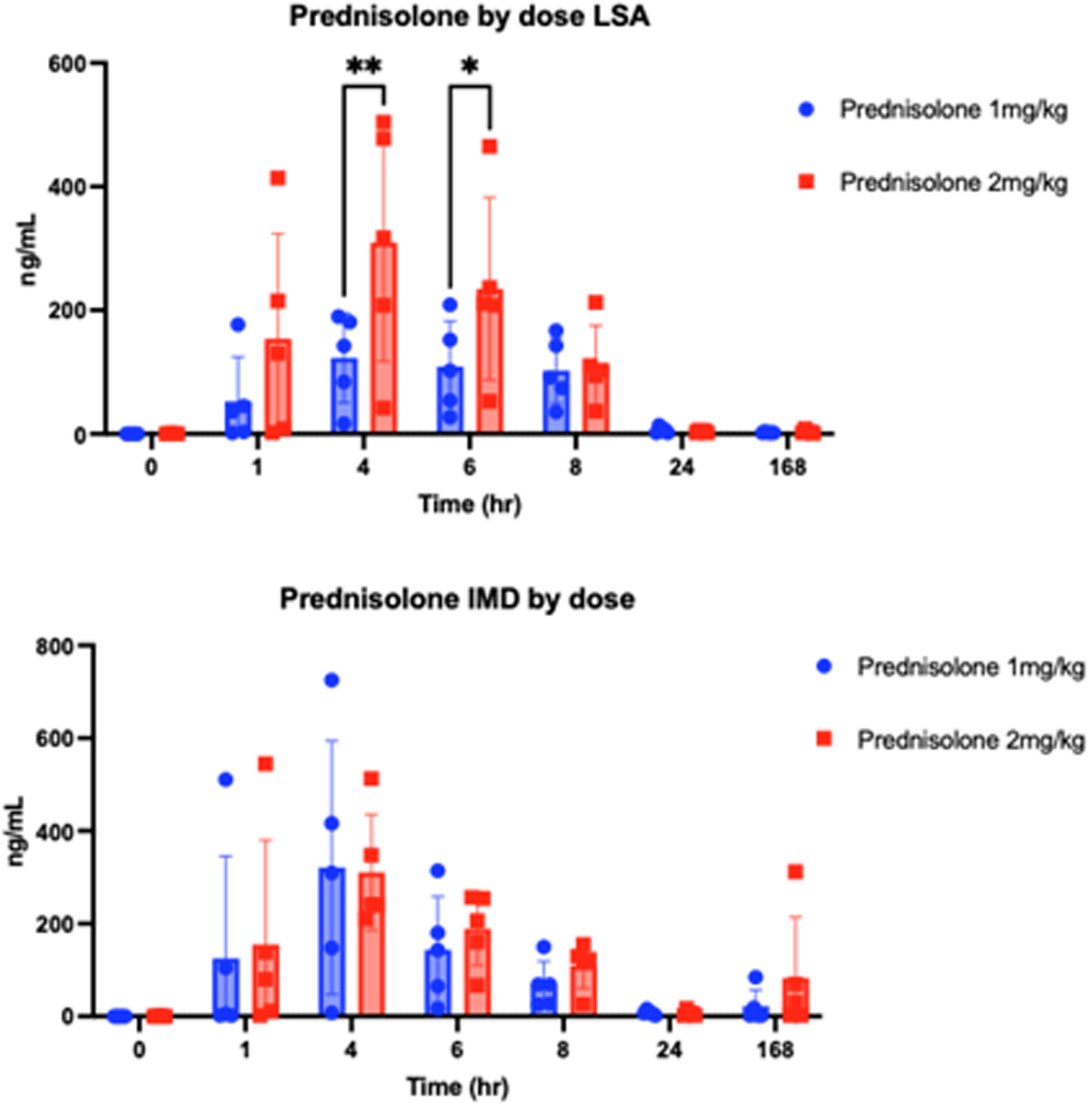
Figure 1

**Figure:**
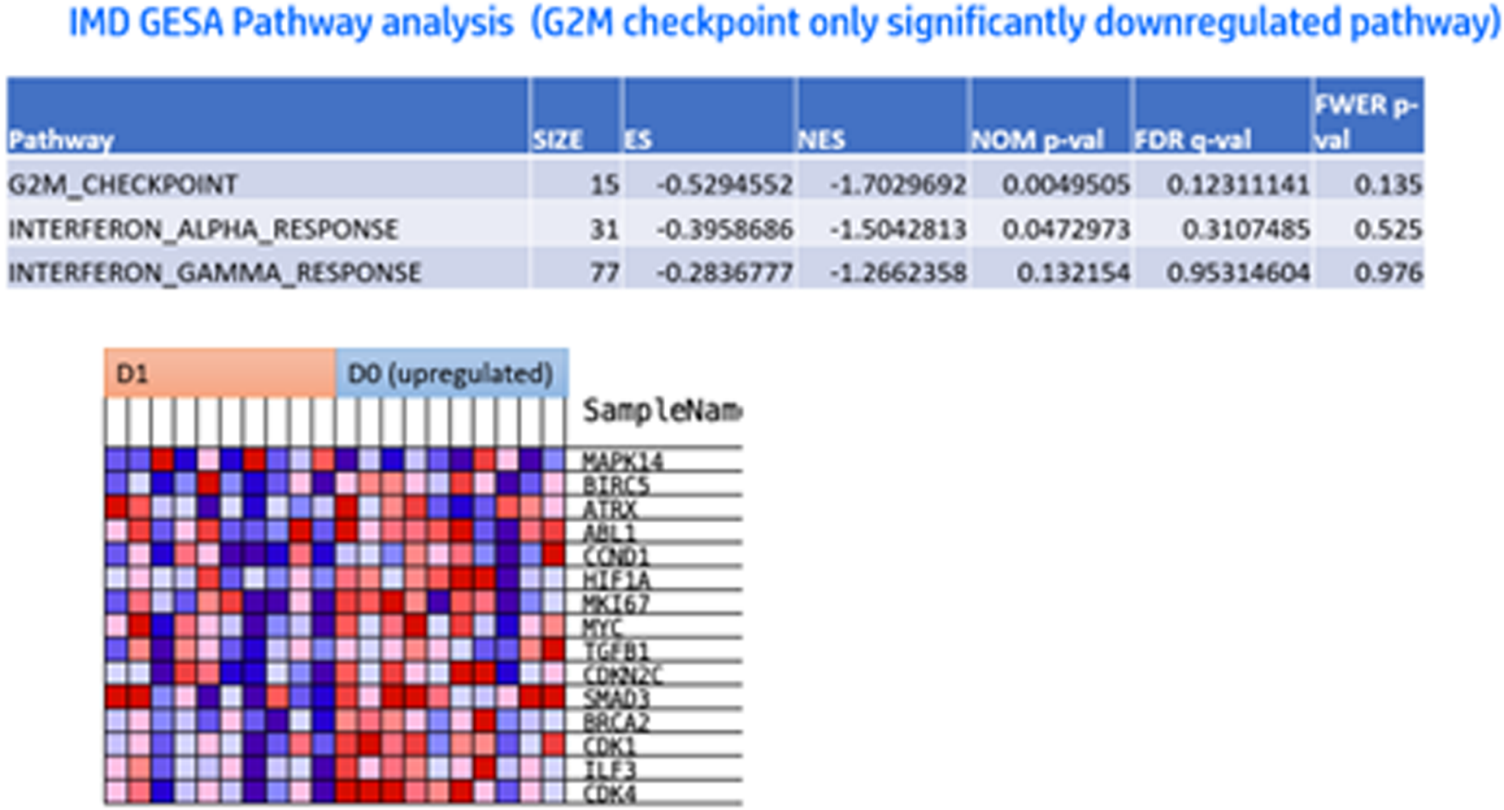
Figure 2

## 224 ABSTRACT N01: Case‐Control Investigation of Clinician‐Determined Variables in Progressive Myelomalacia After Disc Herniation in Dogs

### 224.1 **Eileen Donoghue** ^1^; Joseph Mankin^2^; Nick Jeffery^2^

#### 224.1.1 ^1^Neurology Resident, Veterinary Medical Teaching Hospital, Texas A&M University; ^2^Professor, Neurology, Veterinary Medical Teaching Hospital, Texas A&M University

**Background:** Progressive myelomalacia after disc herniation in dogs is frequently fatal, and there is no proven effective treatment. More information regarding potential causes, including replication studies, is required as a first step in reducing its incidence.

**Hypothesis/Objectives:** To investigate the association of progressive myelomalacia with (1) variables under veterinary control; and (2) patient characteristics.

**Animals:** 233 dogs presenting to a university neurology clinic with absent hindquarter pain perception following acute thoracolumbar disc herniation over a 10‐year period.

**Methods:** Case‐control analysis of association between progressive myelomalacia and putative causal factors using univariable and multivariable logistic regression. Pre‐specified sample size appropriate to replicate previously implicated associations.

**Results:** 45 (19%) dogs were diagnosed with progressive myelomalacia. None of the exposure factors under veterinary control, including pre‐operative administration of corticosteroids or non‐steroidal anti‐inflammatory drugs, time from presentation to completion of surgery, high or low blood pressure events during anesthesia, 4‐vertebral length durotomy, surgery time and extent of decompressive surgery appeared to be associated with progressive myelomalacia. The analysis supports the previously suggested higher risk for progressive myelomalacia associated with lesions affecting the lumbosacral intumescence versus T3–L3 lesions (OR=3.96; 95% CI: 1.35–11.63).

**Conclusions and Clinical Importance:** Veterinarians currently do not have reliable means to influence the incidence of progressive myelomalacia in a susceptible population of dogs, emphasizing the need for further therapeutic investigations (including formal trials of extensive durotomy). The replication of a high incidence of progressive myelomalacia with lesions of the lumbar intumescence suggests that it is a robust association.

## 225 ABSTRACT N02: Mechanical Sensory Threshold in Neurologically Normal French Bulldogs With Congenital Vertebral Malformations

### 225.1 **Karina Pinal** ^1^; Natalia Zidan^2^, DACVIM; Starr Cameron^2^, DACVIM (Neurology)

#### 225.1.1 ^1^Neurology/Neurosurgery Resident, University of Wisconsin–Madison Veterinary Care; ^2^Faculty, Neurology, University of Wisconsin–Madison

**Background:** French bulldogs are the most common dog breed in the U.S. and they appear to be overrepresented for congenital vertebral malformations. These malformations are often considered an incidental finding on radiographs, but may result in spinal deformities, such as kyphosis or scoliosis. In people, these changes can result in spinal pain.

**Hypothesis/Objectives:** To evaluate paraspinal mechanical sensory thresholds (MST) in French bulldogs with confirmed vertebral malformations and compare with healthy control dogs, to determine if overlooked paraspinal pain is present. We hypothesized there will be no difference in MST measurements between the two groups, despite the presence of congenital vertebral malformations in the French bulldogs.

**Animals:** Twenty‐five client‐owned French bulldogs perceived neurologically normal by their owners were recruited for the study. Twelve dogs of any breed, weighing 8–15 kg, with no history of neurologic disease, were used as control dogs.

**Methods:** Vertebral column radiographs were performed in all dogs. MST was measured by an algometer applied to the paraspinal muscles at three vertebral sites and compared between groups. Algometer maximal force was 11 lbs. *P* < 0.05 was considered significant.

**Results:** The difference in MST measurements between the French bulldog and control groups were not statistically significant at any vertebral sites tested. The mean paraspinal MST in the French Bulldogs and control dogs were 10.9 and 10.6 (scapula), 10.8 and 10.2 (thoracolumbar) and 10.8 and 10 (lumbar), respectively.

**Conclusions:** Presence of vertebral malformations alone in French bulldogs are unlikely to result in secondary paraspinal pain and do not warrant therapeutic intervention.

**Figure:**
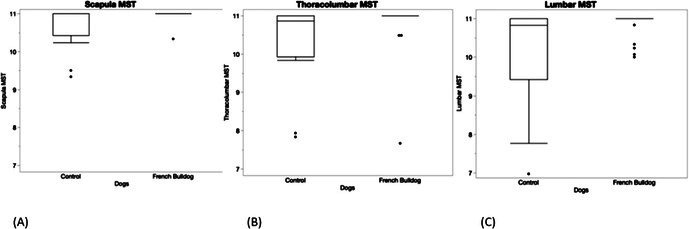
**Figure 1** Box plot graphs of the distribution of mechanical sensory thresholds (MST) in the control and French Bulldog group. Each value is in pounds (lbs). (A) Scapula MST, (B) thoracolumbar MST, (C) lumbar MST.

## 226 ABSTRACT N03: Clinical and Imaging Findings in Fourteen Dogs With Surgically Treated Tethered Cord Syndrome

### 226.1 **Elina Kalamkarova** ^1^; Rachel Lampe^2^, BVetMed, MS, DACVIM (Neurology); Nicholas Sharp^2^, BVM, DACVS, DACVIM

#### 226.1.1 ^1^Neurology Resident, Canada West Veterinary Specialists; ^2^Neurologist, Canada West Veterinary Specialists

**Background:** Tethered cord syndrome (TCS) results from tissue attachments that limit the movement of the conus medullaris or dural sac. This can lead to pain, bladder or bowel dysfunction, and lower limb sensorimotor problems in humans. TCS remains an under‐recognized condition in dogs, with no consensus on diagnosis and treatment.

**Hypothesis/Objective:** To detail the clinical signs, advanced imaging, surgical procedures, and outcomes of dogs diagnosed with suspected TCS.

**Animals:** Fourteen client‐owned dogs.

**Methods:** Medical records were reviewed retrospectively for dogs that underwent imaging and surgery for suspected TCS. Dogs were diagnosed based on a combination of clinical signs and dynamic MRI or CT.

**Results:** Median age of onset of initial signs was 21 months and median age at presentation was 51 months. The most commonly reported clinical signs for all dogs were lumbar pain and exercise intolerance. Each dog underwent either dynamic MRI or CT, which revealed minimal craniocaudal movement of the dural sac, conus medullaris, or both between flexion and extension. In all dogs, a taught external or internal filum terminale, extradural adhesions, or a combination of these structures were found and transected during surgery. All dogs showed subsequent improvement in discomfort and exercise intolerance.

**Conclusion/Clinical Importance:** Dogs with unexplained lumbar pain and exercise intolerance should be evaluated using dynamic imaging. Surgery to remove the tethering structure(s) is indicated when imaging shows minimal movement of the dural sac and conus medullaris.

## 227 ABSTRACT N04: Caretaker‐Reported Quality of Life, Functionality, and Complications Associated With Assistive Mobility Cart Use in Animals

### 227.1 **Melissa Narum** ^1^; Jennifer Repac^2^, DVM, DACVSMR; Erin Miscioscia^3^, DVM, DACVSMR

#### 227.1.1 ^1^Neurology/Neurosurgery Resident, University of Florida; ^2^Clinical Assistant Professor, Integrative and Mobility Medicine, Comparative, Diagnostic and Population Medicine, University of Florida; ^3^Service Chief, Clinical Assistant Professor, Integrative Medicine, Comparative, Diagnostic and Population Medicine, University of Florida

**Background:** Cart use is frequently recommended for patients experiencing mobility impairment secondary to neurologic disease or other health conditions. There are no pre‐existing studies that have explored the impact of assistive mobility carts on companion animals or caretaker quality of life.

**Objective:** To evaluate the impact of assistive mobility carts on companion animals and caretakers’ quality of life by investigating factors pertaining to caretaker satisfaction, the ability to perform daily tasks, and complication rates.

**Animals:** Inclusion and analysis of 954 dog, 219 cat, and 46 rabbit caretaker survey responses.

**Materials and Methods:** A 23‐question survey was distributed to caretakers of animals using carts to evaluate the animal and caretakers’ quality of life, acceptance, ability to complete functional tasks, and complications. Data from canine, feline, and rabbit responses were analyzed separately.

**Results:** Dogs and cats had improved quality of life in 62 and 57% of responses and 61 and 60% for their caretakers, respectively. There was no improvement in the quality of life of rabbits or their caretakers. Regarding the complication rate, 64% were reported to have at least one complication associated with cart use, 53% of which were wounds. Across all species, there was a reported improvement in ability to perform daily tasks and activities.

**Conclusions and Clinical Relevance:** Caretakers reported that assistive mobility carts improve both companion animals’ and caretakers’ quality of life, despite high prevalence of complications, including wounds. Future studies exploring specific disease conditions and long‐term outcomes will be useful for guiding clinical recommendations.

**Figure:**
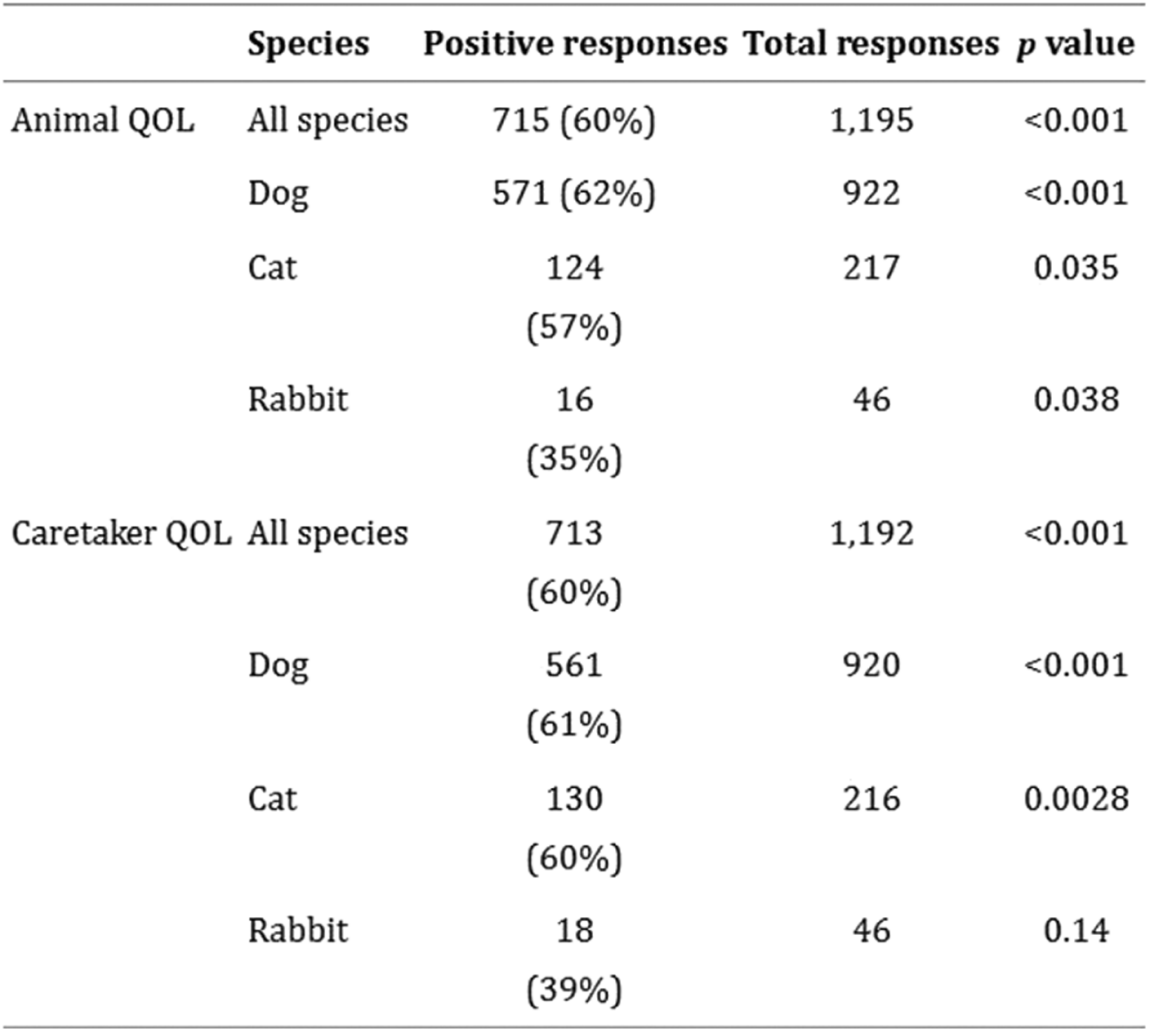
**Table 2** Impact of assistive mobility cart use on animal and caretaker quality of life (QOL) by species.

**Figure:**
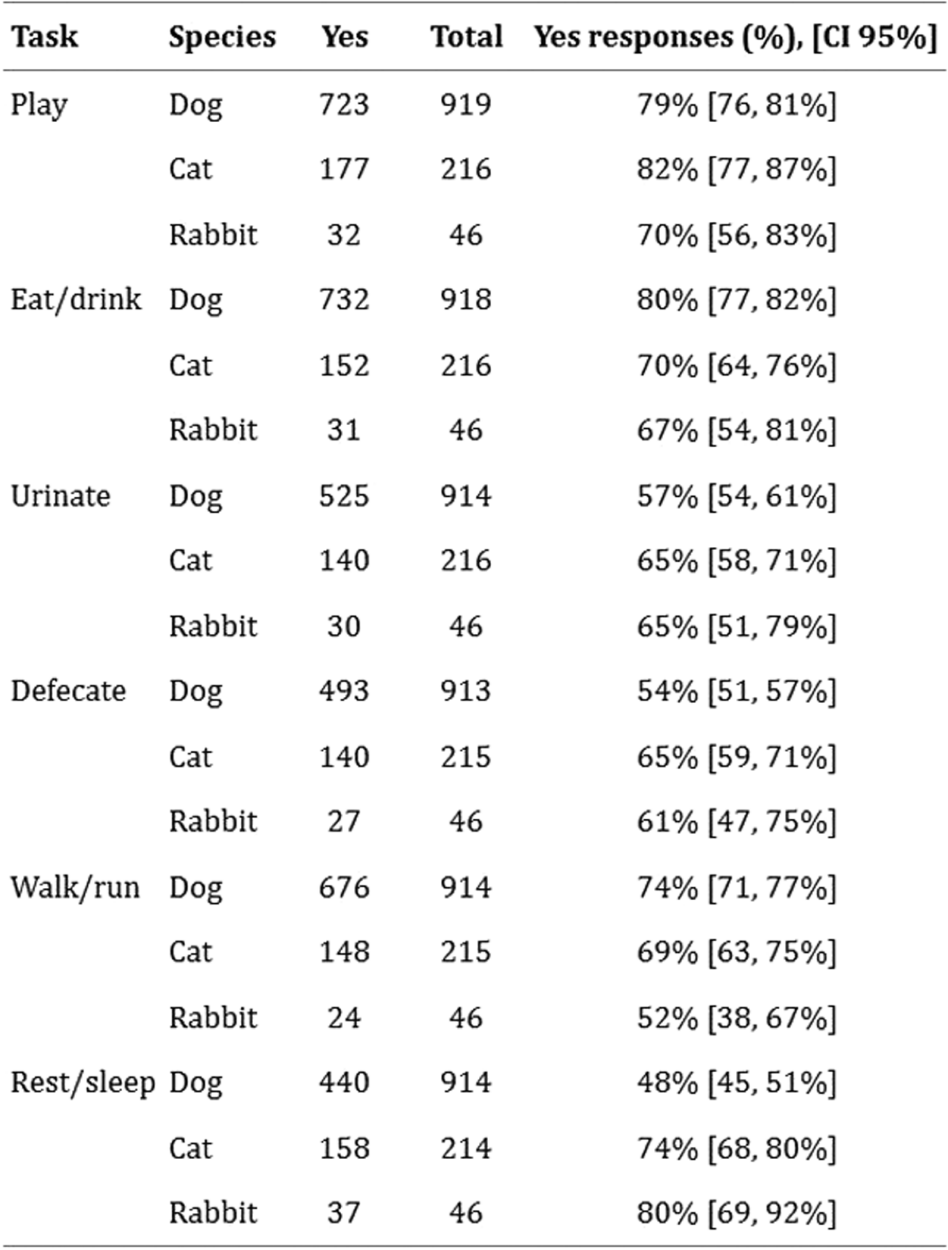
**Table 6** Improvement in functional task performance across species using assistive mobility carts.

## 228 ABSTRACT N05: Breed Predispositions for Canine Otitis Media With Intracranial Extension

### 228.1 **Clifford Petit** ^1^; Vishal Murthy^2^, DVM, DACVIM (Neurology); Karen Vernau^3^, DVM, MAS, DACVIM (Neurology); Peter Dickinson^4^, BVSc, PhD, DACVIM (Neurology)

#### 228.1.1 ^1^Neurology and Neurosurgery Resident, Veterinary Medical Teaching Hospital, School of Veterinary Medicine, University of California−Davis; ^2^Assistant Professor, Clinical Neurology/Neurosurgery, Department of Surgical and Radiological Sciences, School of Veterinary Medicine, University of California−Davis; ^3^Professor, Clinical Neurology/Neurosurgery, Department of Surgical and Radiological Sciences, School of Veterinary Medicine, University of California−Davis; ^4^Professor, Neurology/Neurosurgery, Department of Surgical and Radiological Sciences, School of Veterinary Medicine, University of California−Davis

**Background:** Intracranial extension of otitis media (OM) infection is an infrequent, yet severe complication in dogs. The prevalence and breed predispositions for this complication have yet to be described.

**Hypothesis:** Prevalence of otitis media with intracranial extension (OMIE) is higher in French Bulldogs than other breeds.

**Animals:** 314 client‐owned dogs with definitively diagnosed OM from January 1990–August 2024.

**Methods:** Retrospective study. Medical records were reviewed and cases with a histopathologic and/or microbiological diagnosis of OM were included. Extension of OM was based on histopathology, advanced cross‐sectional imaging, or cerebrospinal fluid analysis. Fisher's exact tests were performed to compare each breed with at least 5 cases against the hospital population and OM population. Holm‐Sidak correction for multiple comparisons was performed.

**Results:** OMIE and OM without extension was identified in 43 and 271 dogs, respectively. Predisposition for OM was identified in French Bulldogs (OR 12.12 [95% CI: 8.92–16.46]), American Cocker Spaniels (8.59 [6.06–12.17]), Pugs (6.40 [4.07–10.09]), Bulldogs (4.70 [2.84–7.78]), Cavalier King Charles Spaniels (6.07 [3.23–11.43]) (*P* < 0.0001 each), and Standard Poodles (4.82 [2.56–9.06]; *P* = 0.0005). Only French Bulldogs were predisposed to OMIE both relative to the hospital (51.50 [28.19–94.10]; *P* < 0.0001) and OM populations (6.36 [0.08 to 0.32]; *P* < 0.0001). The most common organism in the OMIE group was *Staphylococcus* sp. 30‐day survival was excellent (35/38; 92%).

**Conclusions and Clinical Importance:** French Bulldogs are predisposed to the development of OMIE which does not reflect a higher incidence of middle ear disease alone. This may reflect anatomical variations in skull morphology associated with routes of extension.

## 229 ABSTRACT N06: Dissolution Characteristics of Split and Crushed Levetiracetam Extended‐Release Tablets in Comparison With Immediate‐Release Formulation

### 229.1 **Alejandra Mondino Vero** ^1^; Julie Nettifee^2^; Mark Papich^3^; Karen Munana^3^

#### 229.1.1 ^1^Neurology Resident, Department of Clinical Sciences, College of Veterinary Medicine, North Carolina State University; ^2^Department of Clinical Sciences, North Carolina State University; ^3^North Carolina State University

**Background:** Levetiracetam (LEV) is a commonly used antiseizure medication in dogs, available as immediate‐release (LEV‐IR) and extended‐release (LEV‐XR) formulations. LEV‐XR improves compliance by reducing dosing frequency but has limited utility in small dogs based on the minimum tablet concentration of 500 mg. Some clinicians advocate modifying LEV‐XR tablets for administration during cluster seizures, but this practice has not been validated.

**Objectives:** To evaluate the effect of modifying LEV‐XR tablets on *in vitro* dissolution rates and compare to that of intact LEV‐IR.

**Methods:** Dissolution testing followed United States Pharmacopeia (USP) guidelines for LEV‐XR. Intact, split, and crushed LEV‐XR 500‐mg tablets and intact LEV‐IR 500‐mg tablets were each placed in a pH 6.0 buffer at room temperature and agitated at 100 rpm, with experiments run in triplicate. Samples were collected at 0, 0.5, 2, 4, 6, and 8 h and analyzed using high‐performance liquid chromatography.

**Results:** Percentage of drug release over time is depicted in Figure 1. At 30 min, LEV‐IR was completely dissolved; dissolution was 12.9±0.6% for intact, 17.6±0.7% for split, and 89.2±4.6% for crushed LEV‐XR. At 8 h, intact, split, and crushed LEV‐XR tablets reached 72.4±2.4%, 88.8±5.8%, and 95.5±1.8% dissolution, respectively.

**Conclusions and Clinical Importance:** Splitting LEV‐XR minimally affects extended‐release properties, suggesting it may be a practical option for small dogs. Crushing LEV‐XR mimics LEV‐IR, indicating this modification may be useful for cluster seizures. Pharmacokinetic studies are needed to validate *in vivo* application.

**Figure:**
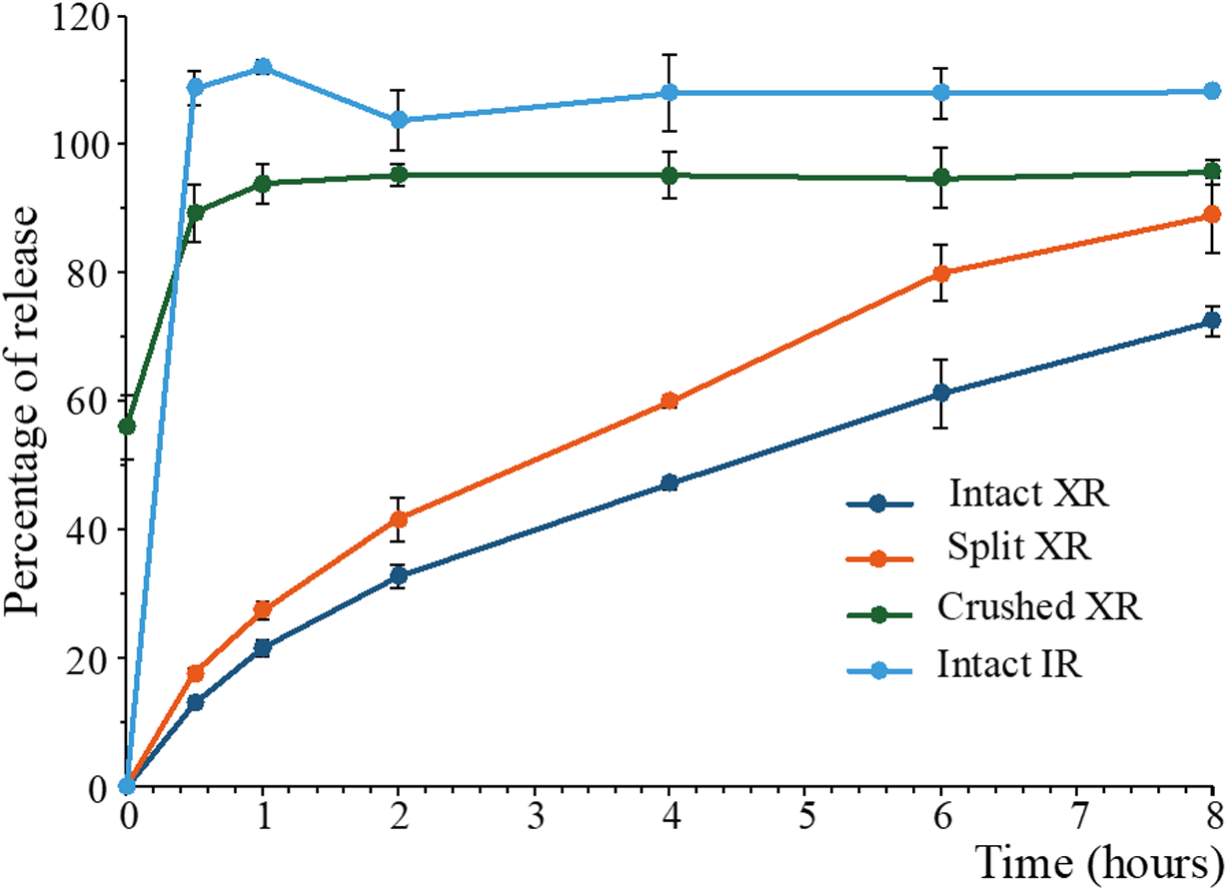
**Figure 1** Comparison of the release profiles over time for intact LEV‐XR, split LEV‐XR, crushed LEV‐XR, and intact LEV‐IR. Error bars represent standard deviations at each time point.

## 230 ABSTRACT N07: Treatment and Prognosis for Canine Calvarial Multilobular Tumor of Bone: 19 Cases (2012–2024)

### 230.1 **Maureen Bennett** ^1^; Renee Barber^2^, PhD, DACVIM (Neurology); Elizabeth Howerth^2^, PhD, DACVP; Marc Kent^2^, DACVIM (Neurology, SAIM)

#### 230.1.1 ^1^Neurology Resident, University of Georgia; ^2^University of Georgia

**Background:** There are no large‐scale analyses of canine calvarial multilobular tumor of bone (MLT); the majority are single case reports or small case series describing novel surgical techniques. Exclusion of mandibular, maxillary, and zygomatic‐derived MLT when considering survival time, risk of tumor recurrence, disease morbidity, and metastasis is critical when considering surgical challenges of calvarial neoplasm.

**Objective:** To evaluate survival time, time to recurrence, and determine possible prognostic factors of calvarial multilobular tumor of bone in treated patients.

**Animals:** Client‐owned companion canines who underwent surgical debulking and stereotactic radiation therapy or surgical debulking alone with histologic diagnosis.

**Methods:** Retrospective cohort study, single center, cases referred between 2012–2024.

**Results:** Histologic grade, duration of clinical signs prior to surgery, and neurologic status prior to surgery had no significant effect on median survival time. Radiation therapy post‐operatively was the only factor evaluated which significantly prolonged both survival time (*p* = 0.0279) and disease‐free interval (*p* = 0.0195). Median survival time with surgery alone 291 days (*n* = 14). Median survival time with surgery and radiation therapy 1543 days (*n* = 5). 78% of patients with surgery alone had tumor regrowth; median days post‐operative regrowth was diagnosed 311 days (98–1651). 1/19 patients diagnosed with metastasis. Median weight 23.7 kg (4.3–42.5).

**Clinical Importance:** Median time to recurrence and median survival time with surgery alone shorter than previously reported literature which grouped all locations of MLT of the skull, though rates of metastasis are significantly lower. Radiation therapy following surgical debulking significantly prolongs survival time and disease‐free interval.

## 231 ABSTRACT N08: Spinal‐Only Meningomyelitis of Unknown Etiology: Clinical Presentation, Diagnosis, Treatment, and Outcome in 23 Dogs

### 231.1 **Jessica Linder** ^1^; Kimberly Anderson^2^, DVM, PhD, DACVIM (Neurology); Sasha Dixon^3^, DVM, MS, DACVIM (Neurology); George Moore^4^, DVM, PhD; Stephanie Thomovsky^4^, DVM, MS, DACVIM (Neurology), CCRP, cVMA

#### 231.1.1 ^1^Neurology and Neurosurgery Resident, College of Veterinary Medicine, Purdue University; ^2^University of Tennessee; ^3^MedVet; ^4^Purdue University

**Background:** Meningoencephalomyelitis of unknown etiology (MUE) is a common cause of neurologic disease in dogs. A paucity of reports exist describing spinal‐only MUE.

**Hypothesis/Objectives:** To evaluate the clinical presentation, diagnosis, treatment, and outcomes of dogs with spinal‐only MUE.

**Animals:** Twenty‐three client‐owned dogs diagnosed with spinal‐only MUE between 2005–2023.

**Methods:** Multi‐center, retrospective, observational study. Dogs with a spinal cord neurolocalization, increased cerebrospinal fluid nucleated cell count, and MRI ruling out extradural spinal cord compression were included. Patient signalment, clinical signs, neurologic examination, diagnostic results, treatment, and outcome were reported.

**Results:** Modified Frankel Score on presentation was grade 2 in 11 dogs, grade 3 in 5 dogs, grade 4 in 4 dogs, and grade 5 in 3 dogs. The median ratio comparing length of T2W hyperintensity to length of L2 vertebral body was 5.6 (range 1.6–15.3). All 23 dogs were treated with prednisone (dose 0.7–3.0 mg/kg/day); 18 dogs received additional immunosuppressive medications. Seven dogs (30.4%, 95% CI: 13.2–52.9%) were alive at study completion; 8 dogs (34.8%, 95% CI: 16.4–57.3%) were euthanized, 8 dogs (34.8%, 95% CI: 16.4–57.3%) were lost to follow‐up. For patients with serial neurologic examinations, 5 had improvement, 9 were unchanged, and 1 had worsening of neurologic grade. At time of study completion, 9 dogs (39.1%, 95% CI: 19.7–61.5%) were euthanized or worsened neurologically.

**Conclusions and Clinical Importance:** Prognosis for spinal‐only MUE is guarded long term.

## 232 ABSTRACT N09: Lipemia Interference in Automated Immunoassay Measurement of Plasma Phenobarbital Concentration

### 232.1 **Phabpim Thumarat** ^1^; Crisanta Cruz‐Espindola^2^, MS; Alex Jenkins^2^, PharmD; Tom Jukier^3^, DVM, MS, DACVIM (Neurology); Kamoltip Thungrat^2^, DVM, PhD, DACVCP

#### 232.1.1 ^1^Small Animal Rotating Intern, Medicine and Surgery, Auburn University; ^2^Auburn University; ^3^Clinical Sciences, College of Veterinary Medicine, Auburn University

**Background:** Phenobarbital is a commonly used antiseizure medication with a narrow therapeutic window, utilizing Therapeutic Drug Monitoring (TDM) to guide appropriate dosing. Lipemia may interfere with automated immunoassay (IA) results, potentially leading to inaccurate phenobarbital concentrations and inappropriate dose adjustments. However, this interference has not been directly evaluated against a reference method in veterinary medicine.

**Objectives:** To evaluate the impact of lipemia on plasma phenobarbital concentrations measured by IA, using high‐performance liquid chromatography (HPLC) as the reference method.

**Animals:** Archived canine plasma samples (*n* = 80) submitted to the Auburn University TDM service, including non‐lipemic, lipemic, and simulated lipemic plasma spiked with 20% Intralipid®.

**Methods:** Phenobarbital concentrations were measured using IA and HPLC. Percent bias was calculated as the difference between quantitated phenobarbital concentration and the reference method. Non‐parametric statistics were applied due to skewed distribution. Lipemia indices were assigned using an automated analyzer, with index 4–6 representing moderate to marked lipemia.

**Results:** HPLC demonstrated consistent specificity, accuracy, and precision across all lipemia levels. In simulated lipemic plasma, percent bias increased with lipemia severity, with the highest median bias at index 6 (34.4%, 13.6–132.5; *P* < 0.001). In clinical samples, plasma with index 4–6—thresholds where turbidity and analytical interference are more likely—showed significantly higher median bias (23.8%, −13.9–89.9) than non‐lipemic samples (1.2%, −27.1–17.1; *P* < 0.001).

**Conclusions and Clinical Importance:** Lipemia significantly interferes with IA‐based phenobarbital measurements. IA results should be interpreted cautiously in lipemic patients, and confirmation with HPLC considered.

## 233 ABSTRACT N10: Owner‐Observed Sleep Disturbances Between Cavalier King Charles Spaniels With and Without Symptomatic Chiari‐Like Malformation

### 233.1 **Amanda Valentino** ^1^; Natasha Olby^2^, VetMB, PhD, DACVIM (Neurology)

#### 233.1.1 ^1^PhD Student, North Carolina State University; ^2^Professor, Neurology and Neurosurgery, North Carolina State University

**Background:** Chiari‐like malformation (CM) and syringomyelia (SM) are prevalent among Cavalier King Charles Spaniels (CKCS). Sleep disturbances are common in people with this malformation; however, no studies have investigated whether symptomatic CKCS experience these.

**Hypothesis/Objectives:** We hypothesized that CKCS with symptomatic CM±SM exhibit sleep disturbances.

**Animals:** Thirty‐nine CKCS with and without symptomatic CM±SM were included. All dogs slept in the same room as their owners.

**Methods:** Questionnaire‐based study conducted using 2 validated questionnaires: CHASE for severity of CM±SM signs, and SNoRE 3.0 for evaluation of sleep disturbances. Dogs with a CHASE score of 7 or greater were classified symptomatic for CM±SM. SNoRE 3.0 scores were compared between symptomatic and asymptomatic dogs using the Wilcoxon rank sum test.

**Results:** There were 19 symptomatic dogs aged 3–10 years (8 males, 11 females), and 20 asymptomatic dogs aged 1–10 years (8 males, 12 females). The mean CHASE score for symptomatic dogs was 12.32 (median: 13.0, range: 7–18) and asymptomatic dogs was 1.85 (median: 1, range: 0–6). The total SNoRE score for symptomatic dogs had a median of 10 (range: 6–30), while asymptomatic dogs had a median of 6 (range: 6–23). Significant differences were found between the groups comparing total SNoRE score, sleep quality, and sleep interruptions (*p* = 0.0016, 0.0052, and 0.0009, respectively).

**Conclusions and Clinical Importance:** CKCS with symptomatic CM±SM exhibit owner‐reported signs of sleep disturbances. Confirmation using polysomnography is warranted and sleep should be considered in the therapeutic plan.

## 234 ABSTRACT N11: Platelet‐to‐Lymphocyte Ratio as a Potential Biomarker in Dogs With Idiopathic Epilepsy

### 234.1 **Sungjae Lee** ^1^; Juwon Wang^2^; Yeon Chae^2^; Hakhyun Kim^2^; Taesik Yun^2^; Byeong‐Teck Kang^2^

#### 234.1.1 ^1^Veterinary Student, Laboratory of Veterinary Internal Medicine, College of Veterinary Medicine, Chungbuk National University; ^2^Laboratory of Veterinary Internal Medicine, College of Veterinary Medicine, Chungbuk National University

**Background:** The platelet‐to‐lymphocyte ratio (PLR) has been identified as a biomarker in various inflammatory diseases and has shown changes in human epilepsy patients.

**Hypothesis/Objectives:** This study aimed to evaluate the diagnostic utility of PLR in dogs with idiopathic epilepsy (IE).

**Animals:** This study included 46 dogs with IE, 24 dogs with hydrocephalus, 39 dogs with meningoencephalitis of unknown etiology (MUE), 17 dogs with brain tumors (BT), and 55 clinically healthy dogs.

**Methods:** This was a retrospective study. PLR was calculated using the formula: platelet count (×10^3^/dL)÷lymphocyte count (×10^3^/dL).

**Results:** The median PLR was significantly higher in IE dogs (132.3) compared to healthy dogs (94.92, *P* < 0.0001), but lower than in dogs with MUE (240.8, *P* < 0.0001), BT (246.5, *P* < 0.0001), and hydrocephalus (164.0, *P* < 0.005). The area under the receiver operating characteristic curve was 0.74 for differentiating IE from healthy dogs, 0.78 for IE vs. structural brain diseases, and 0.84 for IE vs. structural brain diseases with seizures. The optimal PLR cutoff was 111.0 (*P* < 0.0001) to distinguish IE from healthy dogs (sensitivity 69.57%, specificity 76.36%), 161.9 to differentiate IE from structural brain diseases (sensitivity 76.09%, specificity 71.25%), and 167.7 to differentiate IE from structural brain diseases with seizures (sensitivity 81.63%, specificity 80.49%).

**Conclusions and Clinical Importance:** PLR shows promise as a biomarker for diagnosing idiopathic epilepsy in dogs and distinguishing it from structural brain diseases and structural brain diseases with seizures.

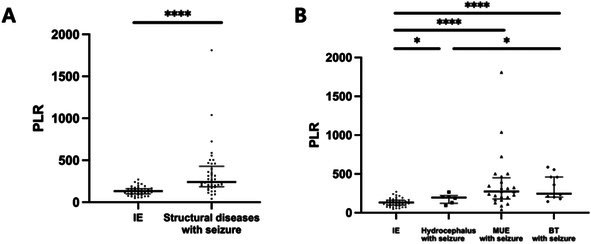

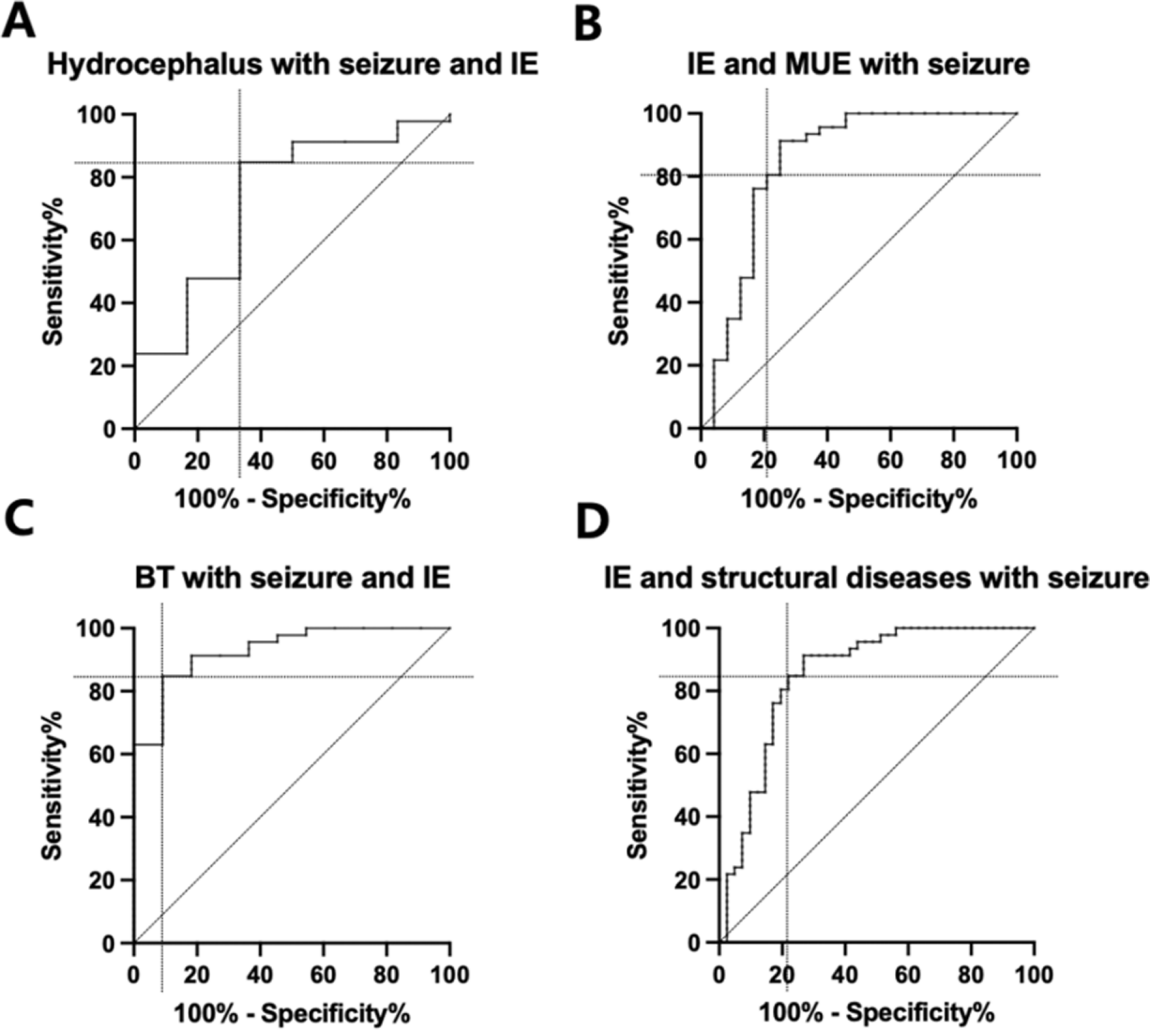

## 235 ABSTRACT N12: The Relationship Between Cognition and Urinalysis Abnormalities in Aging Dogs

### 235.1 **Shaghayegh Rafatpanah Baigi** ^1^; Beth Case^2^; Kate Simon^3^; Amanda Valentino^2^; Telea Wade‐LaHart^4^, DVM; Marco Ruggeri^2^; Christine Whitley^5^; Margaret Gruen^6^; Natasha Olby^7^, VetMB, PhD

#### 235.1.1 ^1^Neurology Research Intern, College of Veterinary Medicine, North Carolina State University; ^2^ College of Veterinary Medicine, North Carolina State University; ^3^DMV PhD Student, College of Veterinary Medicine, North Carolina State University; ^4^Postdoctoral Research Scholar, College of Veterinary Medicine, North Carolina State University; ^5^Research Specialist, College of Veterinary Medicine, North Carolina State University; ^6^Associate Professor, College of Veterinary Medicine, North Carolina State University; ^7^Professor, College of Veterinary Medicine, North Carolina State University

**Background:** In elderly people, especially those with dementia, risk of urinary tract infections (UTI) and bacteriuria is high. Additionally, urine specific gravity (USG) decreases with age. The prevalence of urinalysis (UA) abnormalities and their relationship with cognitive decline has not been described in aging dogs.

**Hypothesis/Objectives:** Cognitive decline in aging dogs is associated with UA abnormalities.

**Animals:** 83 client‐owned dogs, aged 8 to 18 years enrolled in the Longitudinal Study of Neuroaging underwent biannual assessments.

**Methods:** Owner responses from the house soiling domain of Canine Dementia Scale: CADES were recorded. UA abnormalities were documented: pyuria (> 5 wbc/hpf); hematuria (> 5 rbc/hpf); bacteriuria (> trace). In‐house cognitive testing included inhibitory control and detour. The relationship between UA findings, age, sex, CADES soiling score, and cognitive test results was examined with mixed effect models.

**Results:** Of 216 urinalyses in 83 dogs, 33 had pyuria (15), bacteriuria (15), or hematuria (14). Age was associated with a significant decrease in USG (*p* < 0.0001) and correlated with CADES soiling score (*p* < 0.0001). Pyuria was associated with higher CADES soiling score (*p* = 0.0225). Dogs with higher CADES soiling scores showed lower inhibitory control (*p* = 0.0010) and performed worse in detour (*p* = 0.0203). UA abnormalities were not associated with cognitive test performance.

**Conclusions and Clinical Significance:** Aging in dogs is associated with decreased urine specific gravity. House soiling, as described by the owners on the CADES questionnaire, was associated with both urinary tract health (pyuria) and cognition, highlighting the importance of a complete diagnostic workup when owners report house soiling.

## 236 ABSTRACT N13: Post‐surgical Outcomes in Paraplegic Dogs With Asymmetrical Loss of Nociception Secondary to Intervertebral Disc Herniation

### 236.1 Stephanie Chang, DVM

#### 236.1.1 Neurology Resident, BluePearl Pet Hospital (North Dallas), Lewisville, TX, USA

**Background:** Presence of nociception is an important prognostic factor in dogs diagnosed with thoracolumbar intervertebral disc herniation (IVDH) undergoing surgical decompression (hemilaminectomy). Absence of nociception in both pelvic limbs is associated with a worse prognosis. There is little published data on paraplegic dogs with asymmetrical incomplete loss of nociception i.e., nociception present in at least 1 pelvic limb or tail but absent elsewhere.

**Hypothesis/Objectives:** To investigate paraplegic dogs with asymmetrical loss of nociception (classified as Modified Frankel Score; MFS 4b) secondary to thoracolumbar IVDH, and their prognosis of regaining ambulation and continence following hemilaminectomy, in comparison to dogs with absent nociception in both pelvic limbs and tail (MFS 5).

**Animals:** 76 client‐owned dogs with thoracolumbar IVDH with MFS of 4b and 5 treated with hemilaminectomies met the inclusion criteria.

**Methods:** Retrospective cohort study.

**Results:** At the time of recheck (≥ 30 days postoperatively), 80% (16/20) of dogs with presurgical MFS 4b regained ambulation, compared to 27% (15/56) of dogs with presurgical MFS 5. The probability of regaining ambulation was 3× higher in MFS 4b dogs (RR (95% CI) = 3.0(1.9–5.0), *p* < 0.001), and median MFS scores were significantly better at recheck (*p* < 0.001). The probability of having incontinence at recheck was 70% less in MFS 4b dogs (RR (95% CI) = 0.30(0.10–0.63), *p* < 0.001).

**Conclusions and Clinical Importance:** Paraplegic dogs with asymmetrical loss of nociception secondary to thoracolumbar IVDH are likely to have a successful outcome with hemilaminectomy. This information can better guide clients on the decision to pursue surgery.

## 237 ABSTRACT N14: The Relationship Between Owner Assessments of Cognition and Quality of Life in Senior Companion Dogs

### 237.1 **Katherine Simon** ^1^; Katharine Russell^2^, DVM; Beth Case^3^; Margaret Gruen^3^, DVM, MVPH, PhD, DACVB; Natasha Olby^3^, MB, PhD, MRCVS, DACVIM

#### 237.1.1 ^1^Graduate Student, North Carolina State University, Raleigh, NC, USA; ^2^Southeast Veterinary Neurology, Miami, FL, USA; ^3^Neurology, College of Veterinary Medicine, North Carolina State University, Raleigh, NC, USA

**Background:** Age‐related cognitive decline is well recognized in dogs. Quality of life (QoL) is another critical consideration for owners of aging pets. However, little is known regarding the relationship between owner assessed cognitive changes and quality of life.

**Hypothesis/Objectives:** We hypothesize that owner assessments of senior dog cognition and QoL will correlate.

**Animals:** Fifty‐seven senior dogs that completed a 6‐month clinical trial.

**Methods:** Owners completed the Canine Cognitive Dysfunction Rating (CCDR) and a modified Canine Owner Reported Quality of Life (CORQ) questionnaire during the trial. The cross‐sectional relationship between CCDR and CORQ at baseline was evaluated. To investigate the longitudinal relationship, difference in CCDR and CORQ score between baseline and month 6 was calculated. Bivariate analyses were performed and Spearman correlation coefficients (r) were obtained to quantify the relationship between CCDR and CORQ. P values <0.05 were significant.

**Results:** Dogs represented a variety of breeds, ages (mean: 13.03 years, sd: 1.54) and weights (median: 23 kg, range: 6.5–39.5). At baseline, there was a significant correlation (*p* = 0.03, *r* = ‐0.29) between CCDR (median: 38, range: 34–58) and CORQ (median: 5.35, range: 3.06–6.53) scores. Owner assessments of cognition and QoL remained stable across six months (CCDR difference [median: ‐1, range: ‐29–16], CORQ difference [mean: 0.35, sd: 1.08]). However, there was a significant (*p* < 0.0001) correlation (*r* = ‐0.51) between CCDR and CORQ differences.

**Conclusions and Clinical Importance:** Owners’ perception of their pet's cognitive status is influencing their assessment of quality of life both cross‐sectionally and over time.

## 238 ABSTRACT N15: Quantification of Effective Implant Size on CT for Cortical Stainless‐Steel Screws

### 238.1 **Stephanie Fonseca** ^1^, DVM; Peter Early^2^; Melissa Lewis^2^; Karl Kraus^3^; Kim Love^4^; Nathan Nelson^2^

#### 238.1.1 ^1^Neurology Resident, Neurology, North Carolina State University, Raleigh, NC, USA; ^2^North Carolina State University, Raleigh, NC, USA; ^3^Iowa State University, Ames, IA, USA; ^4^K. R. Love Quantitative Consulting and Collaboration, Athens, GA, USA

**Background:** Stainless steel cortical screws (CS) are commonly used in veterinary spinal stabilization procedures with computed tomography (CT) used postoperatively to evaluate implant placement.

**Hypothesis/Objectives:** This study evaluated the impact of metallic artifact on CT measurements of CS using an *in vitro* model.

**Methods:** Eighteen screws (6 each of 2.0 mm, 2.7 mm, and 3.5 mm) were placed in ballistic gel and the construct underwent a CT scan. For each screw, three blinded reviewers measured maximum screw width using a bone window (W 2000, L 500) while a non‐blinded reviewer performed the same measurements using an ultrawide window (W 20,000, L 3000). The difference between measured and actual screw size (mean gross difference) was compared between screw sizes and window level.

**Results:** The mean gross difference across all screw sizes ranged from 1.291 to 2.788 mm with a percentage overestimation of 64.6 to 82.5%. The largest mean gross difference was seen in 3.5 mm screws. A positive correlation was found between implant size and gross difference (*p* < 0.001). The mean gross difference using the bone window was significantly larger than the ultrawide window for 2.7 mm (*p* = 0.059) and 3.5 mm (*p* = 0.011) screws.

**Conclusions:** Metallic artifact affects CT measurements of CS, with greater overestimation in bone window when compared to ultrawide window. These findings suggest that using an ultrawide window may improve measurement of screws in challenging clinical cases.

## 239 ABSTRACT N16: Objective Assessment of a Novel Hearing Protection Device in Dogs

### 239.1 **Joshuah Klutzke** ^1^, DVM; George Moore^2^, DVM, PhD; Stephanie Thomovsky^3^, DVM, DACVIM

#### 239.1.1 ^1^Neurology/Neurosurgery Resident, Purdue University, West Lafayette, IN, USA; ^2^Purdue University, West Lafayette, IN, USA; ^3^Neurology, Purdue University, West Lafayette, IN, USA

**Background:** Prolonged exposure to loud noises has been shown to affect hearing acuity in dogs. Affordable, adjustable, and easy‐to‐fit hearing protection devices (HPD) are needed for dogs.

**Hypothesis/Objectives:** Determine hearing threshold in the same population of dogs without and while wearing novel HPD when frequency of sound is held constant and decibel levels are incrementally decreased.

**Animals:** 22 healthy shelter dogs were recruited over a 4‐month period.

**Methods:** Prospective study. Healthy dogs between the ages of 4 months and 69 months were anesthetized for routine surgical alteration. Hearing thresholds (AS and AD) were determined using Brainstem Auditory Evoked Response (BAER) and over the ear headphones (OTEH) in the same population of dogs prior to and while wearing a novel HPD, which fits over the ears and fastens into place.

**Results:** Median hearing thresholds for dogs without the HPD, were 40 dB AS (range 5–70 dB) and 50 dB AD (range 20–70 dB). All dogs with the novel HPD in place had hearing thresholds greater than 70 dB, except one dog whose threshold was 65 dB AD (*p* < 0.0001).

**Conclusions and Clinical Importance:** The novel HPD effectively raised hearing thresholds in dogs, blocking sound of at least 65 dB in all dogs. This HPD may help preserve hearing acuity in dogs exposed to loud noises.

## 240 ABSTRACT N17: Evaluation of Hemoglobin, Albumin, Lymphocyte, and Platelet Score for Predicting Short‐Term Mortality in Canine MUE

### 240.1 **Jungmin Lym** ^1^, DVM; Sejung Ahn^1^; Yeon Chae^1^, PhD; Hakhyun Kim^2^, PhD; Byeong‐Teck Kang^2^, PhD; Taesik Yun^2^, PhD

#### 240.1.1 ^1^Veterinary Student, Laboratory, Veterinary Internal Medicine, College of Veterinary Medicine, Chungbuk National University, Chungcheongbuk‐do, South Korea; ^2^Professor, Laboratory of Veterinary Internal Medicine, College of Veterinary Medicine, Chungbuk National University, Chungcheongbuk‐do, South Korea

**Background:** Meningoencephalitis of unknown etiology (MUE) is a common inflammatory condition of the CNS in dogs, with high mortality. The hemoglobin, albumin, lymphocyte, and platelet (HALP) scores are a novel prognostic tool for predicting short‐term mortality in human diseases. However, its utility in MUE dogs remains unexplored.

**Objectives:** To evaluate the utility of the HALP score as a predictor of short‐term mortality in MUE dogs.

**Animals:** Thirty‐six dogs diagnosed with MUE.

**Methods:** Retrospective study. Medical records were reviewed to confirm the diagnosis of MUE and determine HALP scores.

**Results:** The HALP score was significantly higher in the survival group compared to the non‐survival group for both three‐month (*P* = 0.0025) and one‐year mortality (*P* = 0.0004). The area under the receiver operating characteristic curve of the HALP score for predicting mortality was 0.7906 for three‐month and 0.8016 for one‐year mortality. An optimal cut‐off of the HALP score 12.25 had 50.00% sensitivity and 95.00% specificity for predicting three‐month mortality. An optimal cut‐off of the HALP score 34.33 had 66.67% sensitivity and 95.24% specificity for predicting one‐year mortality. Dogs with HALP score ≤12.25 had a shorter median survival time (MST) of 31 days, compared to 283 days for those with HALP score > 12.25 (*P* = 0.0007). Similarly, dogs with a HALP score ≤34.33 had a shorter MST of 67.5 days, compared to 971 days for those with HALP scores > 34.33 (*P* = 0.0030).

**Conclusions and Clinical Importance:** The HALP score could be a useful predictor of short‐term mortality in MUE dogs.

## 241 ABSTRACT NM01: Increasing Dietary Fiber Intake Improves Fecal and Clinical Activity Scores in Dogs With Chronic Enteropathy

### 241.1 **Dana Ford** ^1^; Adam Rudinsky^2^; Isabel Muirragui^2^; Elena Landau^2^; Jenessa Winston^2^; Valerie Parker^2^

#### 241.1.1 ^1^Small Animal Internal Medicine Resident, The Ohio State University; ^2^The Ohio State University

**Background:** Nutrition is a mainstay of management of canine chronic enteropathy (CE). While many veterinarians prioritize elimination diet trials, there seems to be less consideration paid to fiber‐responsive enteropathies.

**Hypothesis:** Modifying dietary fiber intake can improve objective clinical markers in dogs with CE.

**Animals:** Dogs with CE presented to the Nutrition service (*n* = 32).

**Methods:** Retrospective case series of CE dogs for whom dietary fiber intake was intentionally increased or altered as the primary nutritional goal. Purina fecal scores, canine inflammatory bowel disease activity index (CIBDAI) scores, and fiber concentrations (g per 100 kilocalories [kcal]) of diets fed at time of initial nutrition consult (baseline) and at time of follow‐up were compared. Data were analyzed using a Wilcoxon matched‐pairs signed rank test with significance defined as *P* < 0.05.

**Results:** Median (range) crude fiber and total dietary fiber increased from 0.8 g/100 kcal (0.2–2.5 g/100 kcal) and 1.7 g/100 kcal (0.1–6.6 g/100 kcal) at baseline to 1.8 g/100 kcal (0.3–3.0 g/100 kcal) and 4.6 g/100 kcal (0.5–6.6 g/100 kcal) at follow‐up. Median (range) time from baseline to follow‐up was 21 weeks (4–82 weeks). Fecal scores significantly decreased from baseline to follow‐up with a median (range) of 4 (2–7) to 2 (2–3), respectively (*p* < 0.0001). CIBDAI scores significantly decreased from baseline to follow‐up with a median (range) of 4 (1–8) to 1 (0–4), respectively (*p* < 0.0001). At follow‐up, 27/32 (84%) of dogs had improved fecal scores and 29/32 (90%) had improved CIBDAI scores.

**Conclusion and Clinical Importance:** Increased dietary fiber intake can improve fecal and clinical activity scores in dogs with CE.

**Figure:**
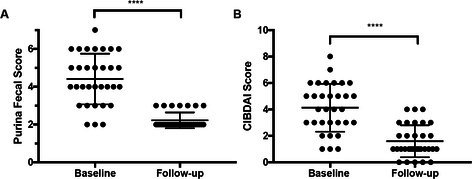
**Figure 1** (A) Purina fecal scores between baseline and follow‐up. (B) Canine inflammatory bowel disease activity index (CIBDAI) scores between baseline and follow‐up.

## 242 ABSTRACT NM02: Exploring Veterinary Professionals’ Strategies for Communicating and Documenting Canine Obesity

### 242.1 **Shawna Morrow** ^1^; Sarah K. Abood^2^, DVM, PhD; Caitlin Grant^3^, DVM, DVSc, DECVCN; Deep Khosa^4^, BSc, BVMS, MANZCVS (Small Animal Medicine), PhD; Adronie Verbrugghe^2^, DVM, DVSc, DECVCN

#### 242.1.1 ^1^Graduate Student, Ontario Veterinary College, University of Guelph; ^2^Associate Professor, Clinical Studies, Ontario Veterinary College, University of Guelph; ^3^Board Certified Veterinary Nutritionist, Clinical Studies, Ontario Veterinary College, University of Guelph; ^4^Associate Professor, Population Medicine, Ontario Veterinary College, University of Guelph

**Background:** Research on how veterinarians and technicians communicate, and document canine obesity diagnoses is limited.

**Objective:** This study explores communication techniques and medical record reporting of veterinarians and technicians regarding canine obesity.

**Methods:** A descriptive survey was conducted in Ontario, Canada between January and August 2022; 152 veterinarians and 196 technicians participated. Descriptive statistics and T‐tests identified differences in communication and documentation.

**Results:** Both groups ranked demonstrating visual and palpation tools as their top method for communicating obesity diagnoses. On a scale from 0 to 10, both groups were most likely to record bodyweight (mean rank±SD: veterinarians: 8.29±2.27; technicians: 8.93±2.05) and body condition score (veterinarians: 8.09±2.31; technicians: 8.25±2.40). Morphometric measurements and body fat index were least likely to be recorded. Veterinarians were more likely to record muscle condition score (t(263)=3.69, *p* = 0.0003) and morphometric measurements (t(214)=2.88, *p* = 0.004), and technicians more likely to record bodyweight (t(341)=−2.72, *p* = 0.006). When concerned about a dog's weight, most participants reported discussing it with guardians and recommending a weight management plan but believed less than half followed through. More veterinarians than technicians reported having these discussions (t(340)=4.05, *p* = 0.00006), and providing recommendations (t(341)=3.51, *p* = 0.0005).

**Conclusion and Clinical Importance:** Despite use of appropriate communication techniques, improvements are needed to help guardians complete weight loss plans. Enhanced documentation, further technician training, and better use of diagnostic tools could improve support.

## 243 ABSTRACT NM03: Validation of a Food Frequency Questionnaire (FFQ) for Use in Dog Nutrition Research

### 243.1 **Janice O’Brien** ^1^; Audrey Ruple^2^, DVM, MS, PhD, DACVPM, MRCVS, FNAP; M. Tolbert^3^, DVM, PhD, DACVIM (SAIM, Board Certified Veterinary Nutritionist)

#### 243.1.1 ^1^PhD Student, Virginia‐Maryland College of Veterinary Medicine; ^2^Dorothy A. and Richard G. Metcalf Professor of Veterinary Medical Informatics, Population Health Sciences, Virginia‐Maryland College of Veterinary Medicine; ^3^Associate Professor, Gastrointestinal Laboratory, Texas A&M University

**Background:** A Food Frequency Questionnaire (FFQ) is a tool used by human nutrition researchers to estimate the average intake of food in a population of people. One has never been validated in veterinary research.

**Hypothesis/Objectives:** To validate the Semi‐Quantitative Food Frequency Questionnaire used by the Dog Aging Project (DAP) to collect dietary information for the prospective cohort study.

**Animals:** 1236 DAP participating dogs.

**Methods:** A stratified random sample of 3043 Dog Aging Project participants consuming eight different diet types was taken. Participants who had completed a dietary questionnaire (FFQ) were invited to retake it (FFQ retest) and then document their pet's food consumption over three days using a food diary (FD). The food items reported on the FDs were categorized according to daily equivalent frequency (DEF), and the DEFs for the FFQ, retest, and food diary were compared using Spearman rank coefficient, intraclass correlation coefficient, and the Wilcoxon Rank Sum test.

**Results:** Of the 3043 participants invited, 1236 completed both the retake questionnaire and the 3‐day food diary. The FFQ accurately records Primary Diet Component, Secondary Diet Component, meats, fruits, and treats. Reported vegetable and dairy intake was less reliable.

**Conclusions and Clinical Importance:** This is the first time a FFQ has been validated for dogs using the same methods and standards applied in human nutrition epidemiology. This work will be instrumental for any future researchers using DAP data, and this FFQ will have applications beyond research.

**Figure:**
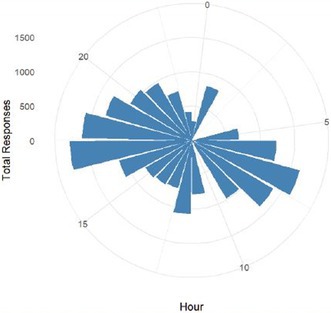
The reported hours at which dogs were fed, according to their food diary—on a 24‐h clock.

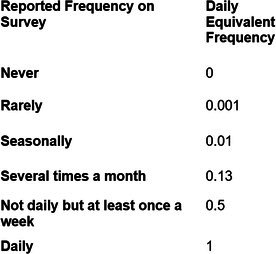

## 244 ABSTRACT NM04: Home‐Prepared Diets for Companion Dogs Are Made From Diverse Ingredients and Few Are Balanced

### 244.1 **Janice O’Brien** ^1^; Audrey Ruple^2^, DVM, MS, PhD, DACVPM, MRCVS, FNAP; Kath Tolbert^3^, DVM, PhD, DACVIM (SAIM, Board Certified Veterinary Nutritionist)

#### 244.1.1 ^1^PhD Student, Virginia‐Maryland College of Veterinary Medicine; ^2^Dorothy A. and Richard G. Metcalf Professor of Veterinary Medical Informatics, Population Health Sciences, Virginia‐Maryland College of Veterinary Medicine; ^3^Associate Professor, Gastrointestinal Laboratory, Texas A&M University

**Background:** Past research on the dietary adequacy of home‐prepared diets has concentrated on individual dogs, highlighting severe deficiencies in case reports or evaluating online and book recipes. There is a lack of research about how pet owners are actually feeding their dogs at home.

**Objectives:** To describe home‐prepared diets for pet dogs, focusing on typical ingredients and their compliance with Association of American Feed Control Officials (AAFCO) standards for maintenance. Can a complete diet be achieved based on the owner's listed ingredients?

**Animals:** 1765 pet dogs enrolled in the Dog Aging Project.

**Methods:** 1765 free‐text responses provided by dog owners from 1/1/2023 to 12/31/2023 as part of a diet survey were evaluated by two independent coders to describe dietary ingredients. The ingredients were analyzed using Balance.it to assess dietary balance. Diets were categorized as unbalanced (10+ nutrient imbalances), partially balanced (1–10 imbalances), or completely balanced (no imbalances).

**Results:** The most common ingredients were: meat (contained in 89% of diets), vegetables (64%), organs/offal (26%), fruit (25%), non‐meat proteins (22.6%), fats/oils (14%), and nuts/seeds (6%). Of the meat and offal categories, the most common animal sources reported by owners were: chicken, beef, turkey. Due to insufficient information, approximately 17% of diets could not be categorized according to balance. 5% of diets were possibly complete, 25% were partially balanced, and 52% were unbalanced.

**Conclusions:** The ingredients owners choose to feed their dogs in home‐prepared diets are diverse. Unfortunately, only 5% could possibly have met AAFCO standards for maintenance diets.

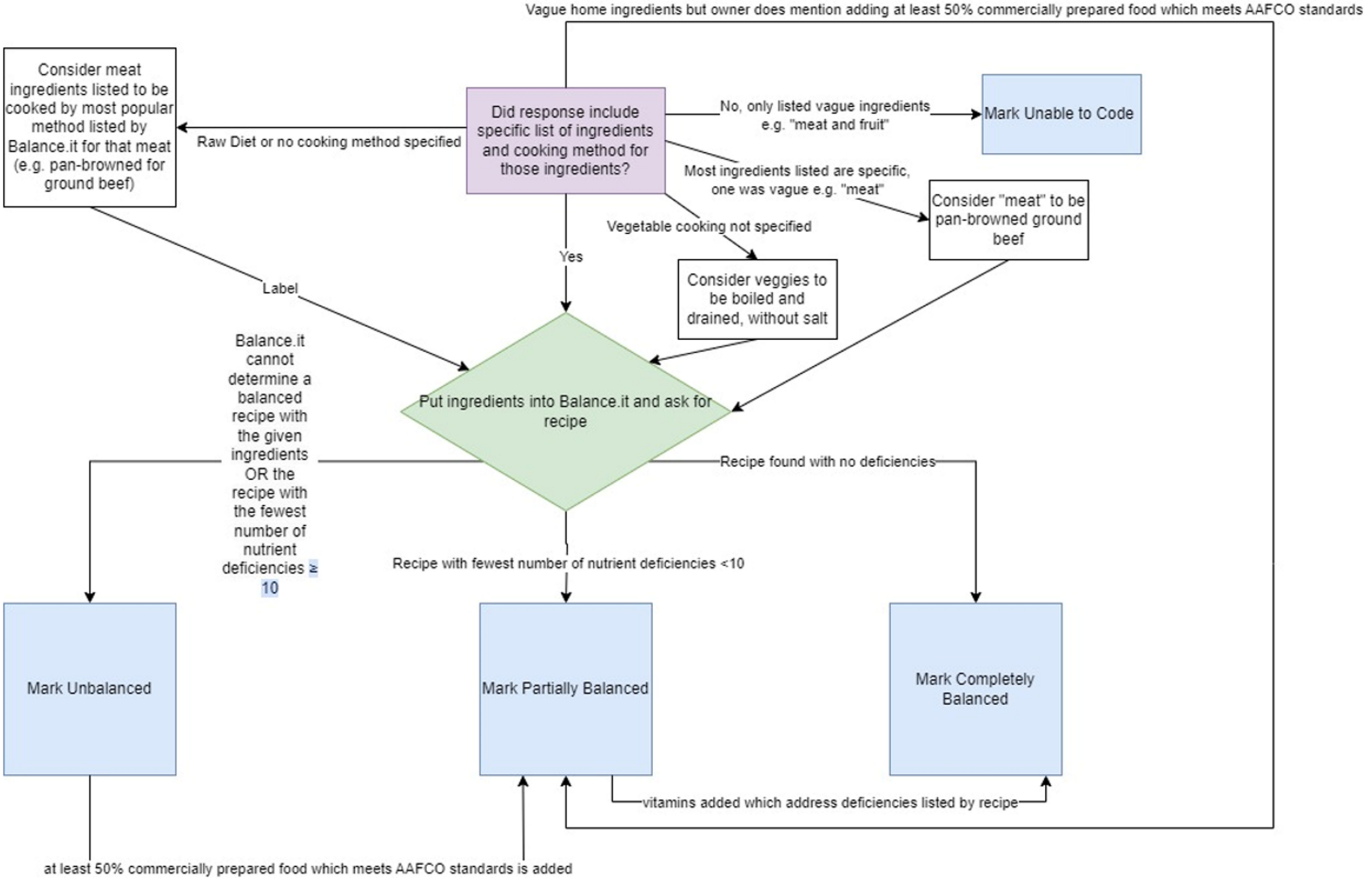

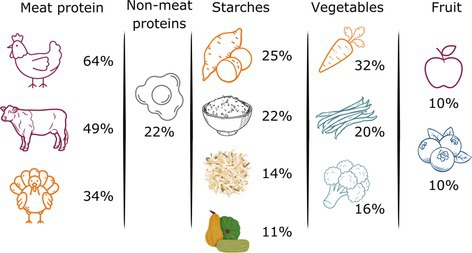

## 245 ABSTRACT NM05: Effects of Dietary Medium Chain Fatty Acids on Feline Renal Function

### 245.1 **Brielle Gordon** ^1^, BS; Kiran Panickar^2^; Regina Hollar^2^; Selena Tavener^2^; Dennis Jewell^3^; Matthew Jackson^2^

#### 245.1.1 ^1^Associate Scientist, Hill's Pet Nutrition; ^2^Hill's Pet Nutrition; ^3^Kansas State University, KS, USA

**Background:** Kidneys require energy to maintain function; renal disease (CKD) manifests decreased oxidative phosphorylation and mitochondrial function. Medium‐chain fatty acids are more efficiently digested, transported to the liver, and absorbed into mitochondria than longer fatty acids. They also generate higher levels of ketone bodies such as beta‐hydroxybutyrate (BHB), which is the kidneys’ preferred energy source. Inflammation is another predominant feature of renal disease, and complex lipids are important mediators of inflammation.

**OBJECTIVE:** Assess circulating markers of energy status and inflammation in felines with renal disease.

**Animals:** Cats with naturally occurring CKD (IRIS stage at least 1 and elevated SDMA; *n* = 16) housed at Hill's Pet Nutrition Center.

**Methods:** Based on gender and age, cats were assigned to the order of feeding of both a control (dry food for management of CKD) or test (control plus 8% C8 medium‐chain triglyceride (MCT)) food, fed for eight weeks in a crossover study design. Analyses included plasma metabolomics (Metabolon), serum biochemistries (in‐house laboratory), and symmetric dimethylarginine (IDEXX). Statistical analyses performed by linear mixed model analysis and MANOVA (JMP).

**Results:** Fasting levels of circulating BHB and metabolites of BHB (e.g., carnitine adducts) were increased by the test food, as compared to control food (*p* < 0.05). There was also a decrease in inflammatory lipid mediators, including dihydrosphingomyelins, plasmalogens, and lysoplasmalogens with test food (*p* < 0.05 vs control).

**Conclusions:** The results indicate feeding MCTs to cats improves energy status and reduces proinflammatory lipids. Feeding MCTs may benefit management of renal disease by correcting energy deficiencies and reducing inflammation.

## 246 ABSTRACT NM06: Changes in Circulating MicroRNAs and Their Potential Beneficial Effects in Canines Fed Dietary Vitamin D

### 246.1 **Kiran Panickar** ^1^, PhD; Selena Tavener^2^, BS; Dennis Jewell^3^, PhD

#### 246.1.1 ^1^Director, Science and Technology Center, Hills Pet Nutrition Center; ^2^Scientist, Science and Technology Center, Hills Pet Nutrition Center; ^3^Professor, Grain Science and Industry, Kansas State University, KS, USA

**Background:** 25‐(OH) D provides nutrient and hormonal properties. Adult dogs may benefit from circulating concentrations above 100 ng/mL to support optimal health.

**Objectives:** Assess the miRNA profile in the plasma of dogs fed different concentrations of vitamin D to understand the relationship between dietary vitamin D and circulating miRNAs.

**Animals:** Adult beagles housed at the Hill's Pet Nutrition Center animal colony.

**Methods:** Five groups of dogs (*n* = 8/group) were fed food of approximately 4000 kcal/kg according to AAFCO maintenance protocols for 6 months, containing one of the concentrations of vitamin D: 796, 3087, 5511, 7314, and 9992 IU/kg (dry matter). Blood samples were taken at baseline and at the end of the study period. Plasma miRNA was assessed using Qiagen's miRCURY PCR array and statistical analysis performed using their GeneGlobe portal.

**Results:** There was a significant increase in the levels of selected miRNAs with the highest dose of vitamin D when compared to the controls (796 IU), including let‐7b, let‐7g, miR‐16, miR‐17, miR‐18a, miR‐20a, miR‐31, miR‐499a (*p* < 0.05). Several of these miRNAs attenuate the expression of proinflammatory genes, including IL‐12, IL‐13, IL‐23, TLR‐4, NFkB pathway genes, and NLRP3 inflammasome. There was a significant downregulation of miRNAs, including miR‐34b and miR‐96 with the highest dose (*p* < 0.05) which could result in decreased levels of selected proinflammatory cytokines.

**Conclusion:** The highest concentration of dietary vitamin D likely has a potential anti‐inflammatory effect and may be beneficial for canine health. An anti‐inflammatory effect would benefit dogs in aging and in conditions associated with excessive inflammation.

## 247 ABSTRACT NM07: Serum Vitamin D Metabolites in Diabetic Dogs

### 247.1 **Camila Torelli** ^1^, MS; Robert Backus^2^; Juliana Jeremias^3^; Erika Pereira^4^; Cristiana Pontieri^3^; Tatiane Pooli^4^; Fabio Teixeira^4^; Solangie Villar^4^

#### 247.1.1 ^1^Professor, UNIFACCAMP; ^2^University of Missouri, Columbia, MO, USA; ^3^Nutritional Development Center, PremieR Pet, Dourado, Sao Paulo, Brazil; ^4^School of Medicine Veterinary and Animal Science, University of Sao Paulo, Sao Paulo, Brazil

**Background:** Hypovitaminosis D is associated with insulin resistance and an increased risk of type 1 and type 2 diabetes mellitus (DM) in humans. In dogs, its role in autoimmune and inflammatory diseases is well established, but its association with DM remains unclear.

**Objectives:** This study aimed to assess whether controlled diabetic dogs exhibit alterations in serum vitamin D status.

**Animals:** Twenty‐seven diabetic dogs, free from other diseases, and 19 healthy dogs were included.

**Methods:** Diabetic dogs underwent a 60‐day stabilization period with NPH insulin and a diet formulated for diabetic dogs. After stabilization, all dogs received the same diet for two months. Healthy dogs were fed the same diet for 60 days. Serum 25‐hydroxyvitamin(OH)D3 (calcidiol) and 24,25‐dihydroxyvitamin(OH)2D3 were analyzed. Wilcoxon or t‐tests compared diabetic and healthy dogs.

**Results:** No significant differences were found in age or median serum calcidiol concentrations between groups (*p* = 0.09). However, diabetic dogs had significantly higher mean serum 24,25(OH)2D3 concentrations than healthy dogs (*p* = 0.007).

**Conclusions and Clinical Importance:** This is the first study standardizing diet to evaluate vitamin D metabolism in diabetic dogs, revealing alterations distinct from human type 1 DM, which is linked to hypovitaminosis D. However, our findings align with juvenile insulin‐dependent diabetes in humans, where increased calcidiol and 24,25(OH)2D3 are also observed. The elevated 24,25(OH)2D3 may result from mitochondrial stimulation of 24‐hydroxylase by DM metabolites, as reported in insulin‐deficient rats.

## 248 ABSTRACT NU01: Evaluation of Three Urinary Biomarkers for the Detection of Meloxicam‐Induced Kidney Injury in Cats

### 248.1 **Matthew Wun** ^1^; Julianne Hwang^2^, PhD; Nicolas Villarino^3^, DVS, PhD, DACVCP

#### 248.1.1 ^1^Small Animal Internal Medicine Resident, College of Veterinary Medicine, Washington State University; ^2^Department of Veterinary Clinical Sciences, College of Veterinary Medicine, Washington State University; ^3^Associate Professor, Department of Veterinary Clinical Sciences, College of Veterinary Medicine, Washington State University

**Background:** Urinary tissue inhibitor of metalloproteinase‐2 (TIMP‐2), insulin‐like growth factor binding protein‐7 (IGFBP‐7), and kidney injury molecule‐1 (KIM‐1) are tubular injury biomarkers used in human medicine. These urinary proteins are found in renal tubular cells and leak into the urine during acute kidney injury (AKI); in addition, KIM‐1 is up‐regulated during tubular injury. The utility of these urinary biomarkers in the prediction and diagnosis of feline AKI remains largely unknown.

**Objectives:** To establish the time course profile of urinary TIMP‐2, IGFBP‐7, and KIM‐1 during repeated administrations of a nephrotoxic dose of meloxicam, and determine whether these profiles differ from saline‐treated cats.

**Animals:** Twelve healthy adult purpose‐bred female cats.

**Methods:** Controlled experimental design. Cats were randomly allocated to control and treatment groups. Cats in the treatment group received meloxicam 0.3 mg/kg subcutaneously every 24 h for 31 days. Cats in the control group received saline. Urinary TIMP‐2, IGFBP‐7, KIM‐1, and creatinine were measured every 4 days. The concentration of urinary biomarkers between groups was compared by calculating the area under the curve (AUC) for each biomarker normalized for urinary creatinine.

**Results:** The AUCs for urinary IGFBP‐7/UC and KIM‐1/UC were higher in the treatment groups compared to the control groups. An increase in urinary TIMP‐2/UC was detected in only 50% of the treatment group.

**Conclusions and Clinical Importance:** Urinary IGFBP‐7, KIM‐1, and TIMP‐2 may be reliable markers of renal tubular injury and diagnosing and monitoring feline AKI.

**Figure:**
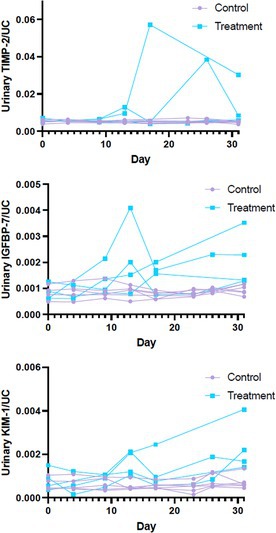
**Figure 1** Urinary tissue inhibitor of metalloproteinase‐2 (TIMP‐2), insulin‐like growth factor binding protein‐7 (IGFBP‐7), and kidney injury molecule‐1 (KIM‐1) normalized by urine creatinine (UC) concentration vs time profile for six cats in the control group and four cats in the meloxicam‐treated renal injury group. During this period, cats in the renal injury group were administered meloxicam 0.3 mg/kg subcutaneously (SC) every 24 h, and cats in the control group were administered saline 0.1 mL/kg SC every 24 h.

**Figure:**
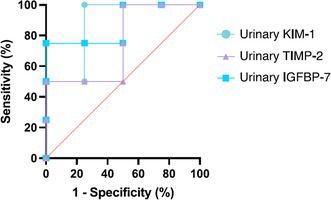
**Figure 2** Receiver operating characteristic (ROC) curves for urinary tissue inhibitor of metalloproteinase‐2 (TIMP‐2), insulin‐like growth factor binding protein‐7 (IGFBP‐7), and kidney injury molecule‐1 (KIM‐1) using the maximum values obtained between days 4 and 13.

## 249 ABSTRACT NU02: Urinary Calcium Excretion and Bone Turnover in Dogs With Calcium Oxalate Urolithiasis: Preliminary Results

### 249.1 **Ammanuel Ouba**, DVM; Matheus Leite; Victoria Gomes; Clara Mori; Danielle Santos; Giovanna Ribeiro; Juliana Carvalho; Marcos Heinemann, DMV, PhD; Fernanda Chacar, DMV, PhD

#### 249.1.1 ^1^School of Veterinary Medicine and Animal Science, University of Sao Paulo, São Paulo, Brazil

**Background:** Hypercalciuria in calcium oxalate (CaOx) urolithiasis may be related to increased bone turnover.

**Objectives:** To evaluate the urinary calcium excretion and bone turnover in dogs with CaOx urolithiasis.

**Animals:** Breed‐ and age‐matched dogs with CaOx stones (*n* = 12) and non‐stone formers (NF; *n* = 12) were prospectively recruited. Dogs using calcium or vitamin D supplements were excluded.

**Methods:** All dogs were fed commercial diets designed for struvite urolith dissolution and CaOx prevention. Fasting serum and urine samples were collected at two time‐points (T1 and T2, with twenty days of interval) to perform the bone marker β‐crosslaps (RI 0.36–2.50 ng/mL) and urine calcium‐to‐creatinine ratio (uCa:Cr; RI ≤0.05). Repeated measures two‐way ANOVA was used for data analysis (p≤0.05).

**Results:** All dogs with CaOx urolithiasis had hypercalciuria, with higher uCa:Cr levels than NF at T1 (*p* = 0.0141). Until now, β‐crosslaps have been assessed in seven dogs. CaOx group (*n* = 4) showed 1.30–8.68; 1.91 ng/mL at T1, and 1.36–2.54; 1.53 ng/mL at T2; NF (*n* = 3) had 0.44–1.88; 1.27 ng/mL at T1, and 0.50–1.77; 0.58 ng/mL at T2. There was a trend towards β‐crosslaps concentrations to be higher in CaOx group than NF. 2/4 dogs with CaOx urolithiasis showed hypercalciuria and high levels of β‐crosslaps at least once.

**Conclusions and Clinical Importance:** Dogs with CaOx and hypercalciuria may have increased bone turnover. Although preliminary, our results highlight the importance of assessing the extent of bone remodeling in hypercalciuric CaOx urolithiasis.

**Figure:**
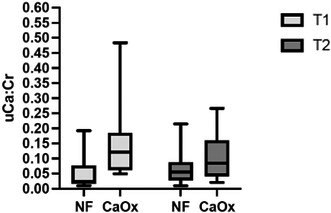
**Figure 1** Urine calcium‐to‐creatinine ratio in dogs with calcium oxalate urolithiasis and non‐stone formers at two time points of evaluation.

**Figure:**
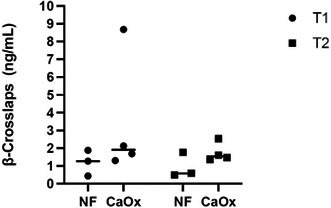
**Figure 2** β‐Crosslaps concentrations (mg/dL) in dogs with calcium oxalate urolithiasis and non‐stone formers at two time points of evaluation.

## 250 ABSTRACT NU03: Effect of Sedation on Kidney Function Markers in Cats with Chronic Kidney Disease

### 250.1 **Zhan Hong Chew** ^1^; Joanna White^2^, BVSc, MVS (Epi), DACVIM (SAIM), PhD

#### 250.1.1 ^1^Small Animal Internal Medicine Resident, Small Animal Specialist Hospital; ^2^Small Animal Specialist Hospital

**Background:** Previous studies had shown that sedation does not adversely affect kidney function in healthy cats, but the effect of study protocol on cats with chronic kidney disease (CKD) is unknown.

**Objectives:** To determine the short‐term effects of sedation on markers of glomerular filtration rate (GFR) in cats with CKD.

**Animals:** Client‐owned cats with CKD undergoing routine abdominal sonography.

**Methods:** Prospective observational study. Each cat received a standard sedation protocol of oral gabapentin, butorphanol, and midazolam intramuscularly, followed by alfaxalone intravenously to effect. Pre‐sedation blood tests included haematology and biochemistry. A second blood test for GFR markers (symmetric dimethylarginine [SDMA] and creatinine) only was collected at the point of maximum sedation (20 cats) or 2 h post‐sedation (12 cats).

**Results:** Thirty‐two cats (age 15 years median, range 7–20) had pre‐sedation SDMA and creatinine concentrations of 19 μg/dL median (Q1–Q3 14.5–23) and 200 μmol/L median (Q1–Q3 147.5–260) respectively. There was no significant difference between pre‐sedation and samples obtained at the point of maximal sedation (SDMA 19 μg/dL median, Q1–Q3 15–24.5, *p* = 0.31; creatinine 200 μmol/L median, Q1–Q3 147.5–255, *p* = 0.71) or 2 h post‐sedation (SDMA 25 μg/dL median, Q1–Q3 20–26, *p* = 0.51; creatinine 200 μmol/L median, Q1–Q3 175–280, *p* = 0.06). The main adverse effect was prolonged sedation after discharge in some cats.

**Conclusion:** Sedation with a combination of gabapentin, butorphanol, midazolam, and alfaxalone appeared adequate for cats with underlying CKD to complete routine, non‐invasive diagnostic procedures and is not associated with short term changes in markers of GFR.

**Figure:**
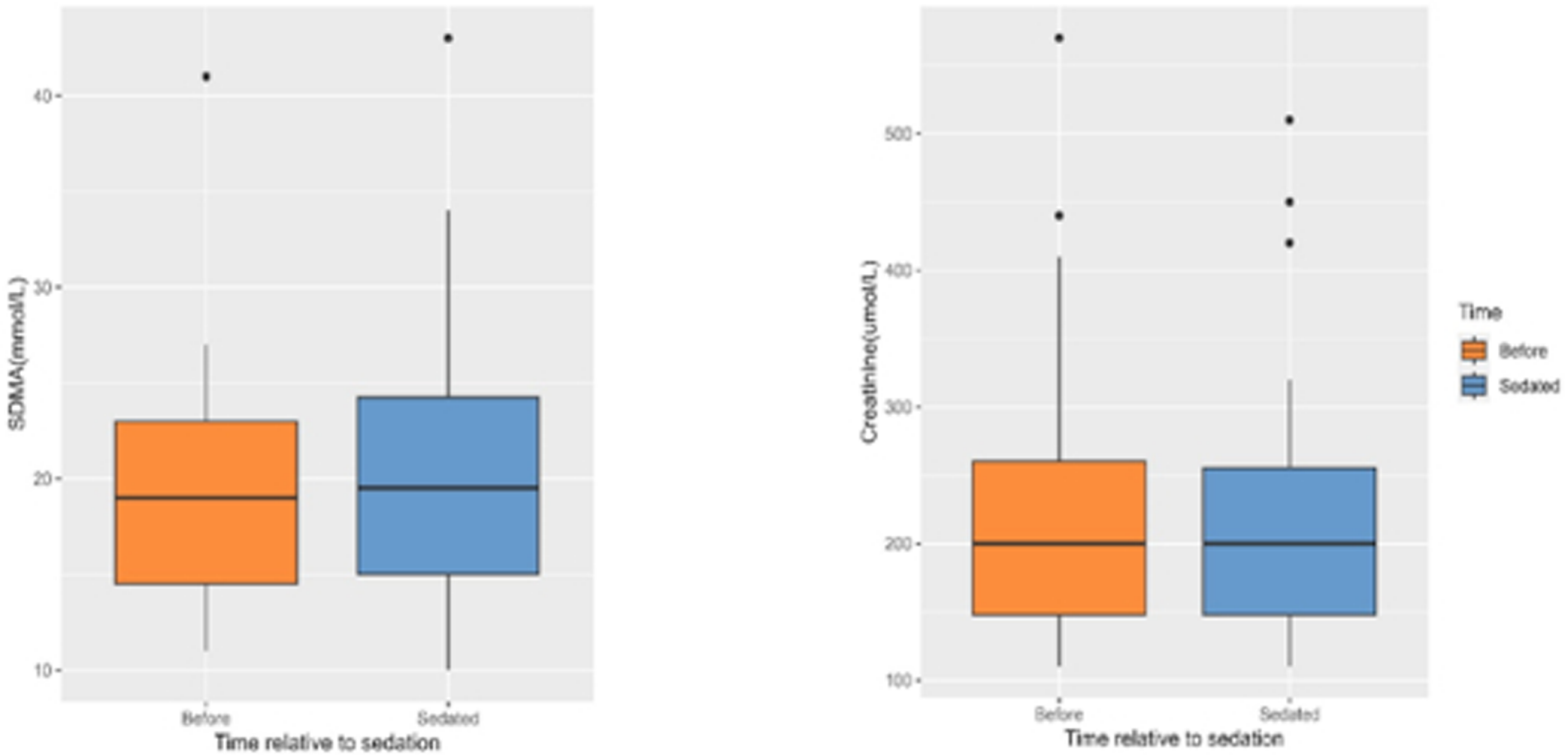
**Figure 1** Box plots comparing symmetric dimethylarginine (SDMA) and creatinine concentrations in 20 cats before and at maximal sedation.

**Figure:**
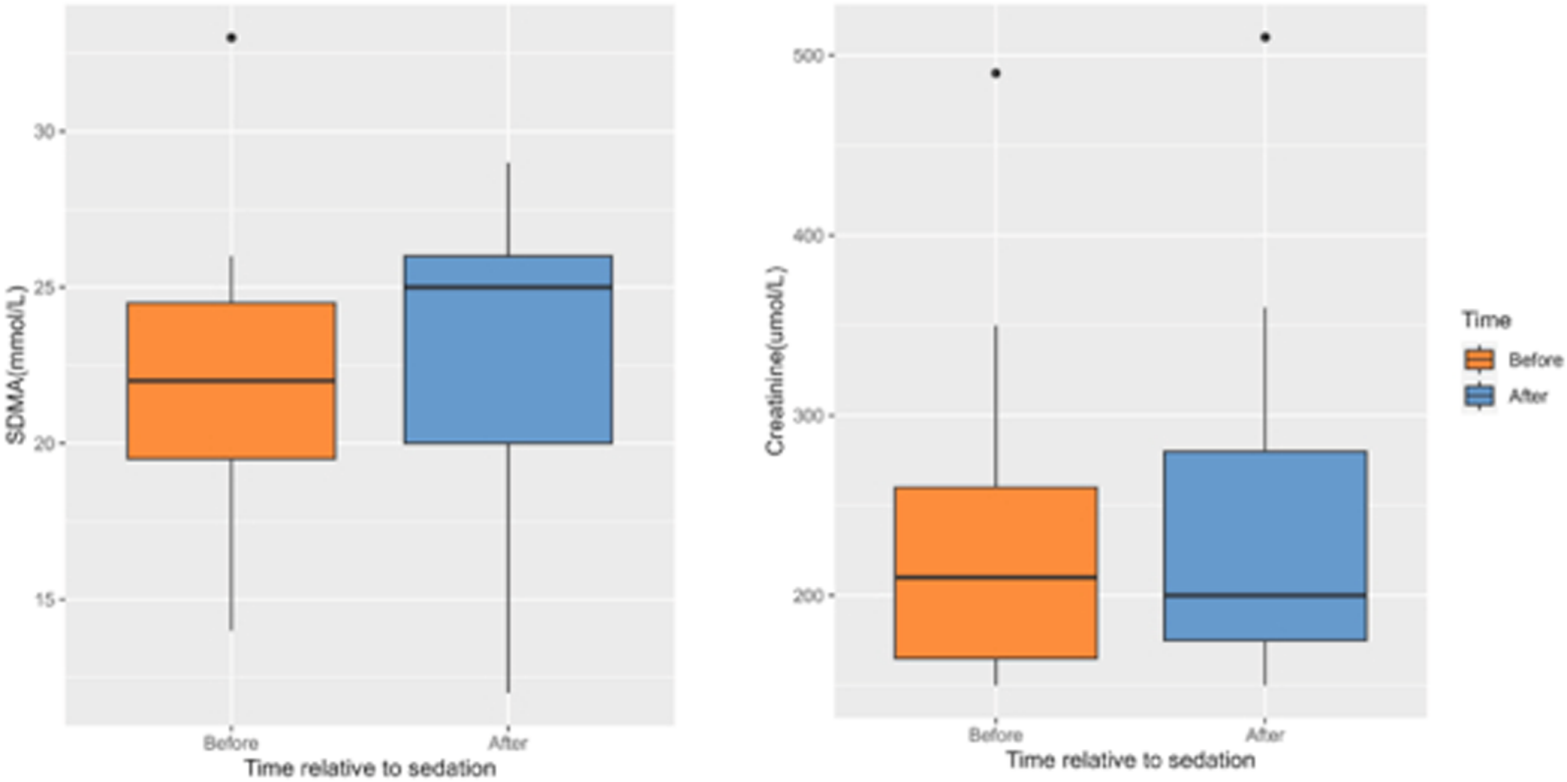
**Figure** Box plots comparing symmetric dimethylarginine (SDMA) and creatinine concentrations in 12 cats before and 2 h after sedation.

## 251 ABSTRACT NU04: Serum Triglyceride and Total Cholesterol in Dogs With Calcium Oxalate Urolithiasis

### 251.1 **Ammanuel Ouba** ^1^, DVM; Giovanna V. Ribeiro^2^; Danielle Santos^2^; Matheus Leite^2^; Victoria Gomes^2^; Juliana De Carvalho^2^; Fernanda Chacar^2^

#### 251.1.1 ^1^School of Veterinary Medicine and Animal Science, University of Sao Paulo, São Paulo, Brazil; ^2^University of Sao Paulo, São Paulo, Brazil

**Background:** Dyslipidemia may be associated with calcium oxalate (CaOx) urolithiasis in rats and humans.

**Objective:** To evaluate serum triglyceride and total cholesterol concentrations in dogs with CaOx urolithiasis.

**Animals:** Breed‐ and age‐matched dogs with CaOx stones (*n* = 12) and non‐stone formers (NF; *n* = 12) were prospectively recruited from the Veterinary Teaching Hospital, University of Sao Paulo. The exclusion criteria were endocrinopathies or the use of n‐3 PUFAs or fibrates.

**Methods:** All dogs were fed commercial diets for struvite urolith dissolution and CaOx prevention. The Body Condition Score (BCS) was assessed, and 12‐h fasting blood samples were collected at two time points with a twenty‐day interval to measure blood glucose (RI 60–130 mg/dL), serum triglyceride (RI 40–168 mg/dL) and total cholesterol (RI 125–270 mg/dL) concentrations. Repeated measures two‐way ANOVA was used for data analysis (p≤0.05).

**Results:** Hypertriglyceridemia was found in 5/12 NF dogs and 6/12 dogs with CaOx urolithiasis at least one time point. Hypercholesterolemia was observed in 3/12 NF dogs and 7/12 dogs with CaOx urolithiasis at least one time point. No differences were found between groups regarding BCS, blood glucose and serum triglycerides at any time point. A main effect of group (*p* = 0.0132) and time (*p* = 0.0132) was observed for total cholesterol, but no interaction between group and time was found.

**Conclusions:** Dogs with CaOx urolithiasis should be screened for hypercholesterolemia. Further studies with proper design are needed to investigate a possible cause‐and‐effect relationship.

**Figure:**
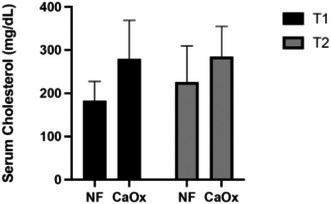
**Figure 1** Serum cholesterol (mg/dL) in dogs with calcium oxalate urolithiasis and non‐stone formers.

## 252 ABSTRACT NU05: Investigating the Microbiome of the Canine Struvite Urolith and Its Link to the Oral Cavity

### 252.1 **Ashleigh Flores** ^1^, DVM; Rodrigo Profeta^2^, PhD; Jodi Westropp^3^, PhD, DVM, DACVIM (SAIM); Maria Soltero‐Rivera^4^, DVM, DACVIM (AVDC); Bart Weimer^5^, PhD

#### 252.1.1 ^1^Student/PhD Candidate, School of Veterinary Medicine, University of California‐Davis, Davis, CA, USA; ^2^Postdoctoral Scholar, Weimer Lab, Population Health and Reproduction, School of Veterinary Medicine, University of California‐Davis, Davis, CA, USA; ^3^Professor, Medicine and Epidemiology, School of Veterinary Medicine, University of California‐Davis, Davis, CA, USA; ^4^Assistant Professor, Surgical and Radiological Sciences, School of Veterinary Medicine, University of California‐Davis, Davis, CA, USA; ^5^Professor, Population Health and Reproduction, School of Veterinary Medicine, University of California‐Davis, Davis, CA, USA

**Background:** A link between the oral microbiome and urolith formation has been investigated in humans, associations in dogs are lacking. While canine struvite urolithiasis is traditionally associated with urease producing organisms, the urolith microbiome and its role in stone formation remains largely unexplored.

**Hypothesis/Objectives:** Canine struvite uroliths contain a diverse microbiome that share genomically indistinguishable organisms to those in the oral cavity.

**Animals:** Eleven dog struvite uroliths submitted to the UC Davis Stone Analysis Laboratory were examined directly. Buccal mucosal swabs from seven healthy dogs were obtained.

**Methods:** A retrospective, cross sectional, non‐case controlled, microbiome study was conducted with total RNA sequencing. Taxonomic and functional profiles of the uroliths and oral microbiomes were examined for common organisms. Comparisons to aerobic urine/urolith cultures, obtained at the time of stone removal, were done.

**Results:** Uroliths contained 693 microbes, of which 322 were shared with the oral swabs. Dogs with positive urine (*n* = 6)/urolith (*n* = 8) cultures yielded *Staphylococcus* or *Enterococcus*. While metatranscriptomic analysis detected abundant *Staphylococcus* species in the stone, it also found numerous microbes, including multiple species of *Enterococcus* and *Porphyromonas* that were shared with oral samples. These organisms can produce ammonia via urease and other metabolic routes including nitrate/nitrite reduction and arginine catabolism.

**Conclusions and Clinical Importance:** Using metatranscriptomics, we found many highly active microbes that are known to produce ammonia through various mechanisms. The numerous shared strains suggest that oral health may be important in urolith formation. Additional studies to characterize microbial community metabolic function will further elucidate the oral/urolith axis.

## 253 ABSTRACT NU06: Incidence of Acute Kidney Injury in Dogs Treated With Extracorporeal Shockwave Lithotripsy

### 253.1 **William Whitehouse** ^1^; Larry Adams^2^, DVM, PhD, DACVIM (SAIM)

#### 253.1.1 ^1^Assistant Professor, Purdue University, West Lafayette, IN, USA; Purdue University, West Lafayette, IN, USA

**Background:** Acute kidney injury (AKI) may result from the treatment of nephrolithiasis with extracorporeal shockwave lithotripsy (SWL), but the incidence and outcome in dogs is unknown.

**Objectives:** To report the incidence and outcome of AKI in dogs treated with SWL and to identify any risk factors.

**Animals:** Fifty‐one dogs, 57 treatments.

**Methods:** Retrospective, single center case series. Medical records from dogs with nephroliths and/or proximal ureteroliths treated with SWL between May 2011 and May 2024 were reviewed. Incidence of AKI was compared between water bath and dry SWL. Cases treated with dry SWL were assessed for risk factors of AKI.

**Results:** Incidence of AKI was significantly higher with dry SWL (10/32 treatments, 31%) versus water bath SWL (2/25 treatments, 8%; *p* = 0.049). AKI was graded as International Renal Interest Society grade I (*n* = 7), grade II (*n* = 2), and grade III (*n* = 3). Serum creatinine returned to baseline in 10/12 (83%) cases. In the cohort treated with dry SWL, dogs with AKI had significantly lower pretreatment creatinine concentrations and lower maximum number of shocks on one kidney compared to dogs without AKI (*p* = 0.003 and *p* = 0.007, respectively). No difference was found with total number of shocks, power setting, maximum stone size, unilateral vs. bilateral treatment, pre‐existing chronic kidney disease, hypotension, or anesthesia duration.

**Conclusions and Clinical Importance:** AKI is more likely with dry SWL but is usually transient. Further studies are needed to see if the relationship between AKI and pretreatment creatinine and number of shocks on one kidney persist in other cohorts.

## 254 ABSTRACT NU07: Early Characterisation of Autophagy and Antioxidant Response in Progressive Feline Chronic Kidney Disease

### 254.1 **Michael Frill** ^1^; Elisavet Vasilopoulou^2^, PhD, PGCHE; Jonathan Elliott^3^, MA, Vet MB, PhD, Cert SAC, DECVPT, MRCVS; Rosanne Jepson^4^, BVSc, MVetMed, PhD, DACVIM, DECVIM, PGCertVetEd, FHEA, MRCVS

#### 254.1.1 ^1^Veterinary Surgeon and PhD Candidate, Royal Veterinary College, London, UK; ^2^Lecturer in Basic Sciences, Comparative Biomedical Sciences, Royal Veterinary College, London, UK; ^3^Professor of Veterinary Clinical Pharmacology, Comparative Biomedical Sciences, Royal Veterinary College, London, UK; ^4^Professor in Small Internal Medicine & Nephrology, Clinical Science and Services, Royal Veterinary College, London, UK

**Background:** Mitochondrial dysfunction leads to excess production of reactive oxygen species (ROS) in renal tubular epithelial cells contributing to inflammation, fibrosis, and progression of chronic kidney disease (CKD). Superoxide Dismutase 1 (SOD1), an antioxidative enzyme, and microtubule‐associated protein 1A/1B light chain 3 (LC3), a marker of autophagy, are cellular defense mechanisms regulating ROS.

**Hypothesis:** Autophagy and antioxidant mechanisms are reduced in progressive CKD.

**Animals:** Snap‐frozen and formalin‐fixed paraffin‐embedded (FFPE) kidney tissue from healthy cats (> 9 years) and those with stable and progressive CKD (*n* = 7/group) euthanized for health reasons.

**Methods:** LC3 activation (LC3ii/LC3i) and SOD1 protein expression were semi‐quantified by Western blot, normalizing to the loading control (β‐actin) in kidney lysates. LC3 and SOD1 localization was assessed by immunohistochemistry using FFPE sections. Kruskal‐Wallis with Dunn correction was used to determine significance between groups.

**Results:** Relative SOD1 protein expression was lower in progressive (median = 0.54 [0.33, 0.83]) compared to stable (median = 1.15 [0.97, 1.17], *p* = 0.013) and control cats (median = 1.49 [1.30, 1.75], *p* < 0.001); Figure 1). LC3 activation was not significantly different between the control (median = 2.05 [1.95, 3.69]) and stable (median = 1.94 [1.71, 2.56], *p* = 0.32) group but those with progressive CKD (median = 1.68 [1.36, 2.36]) trended towards reduced activation compared to control (*p* = 0.06) group. LC3 and SOD1 (Figure 2) were localized to tubular epithelial cells.

**Conclusions and Clinical Importance:** The prominent SOD1 antioxidant response in healthy tubular epithelial cells is diminished in CKD, with lower levels in progressive compared to stable cats. Better understanding factors limiting antioxidant defense in progressive CKD may provide novel therapeutic options.

**Figure:**
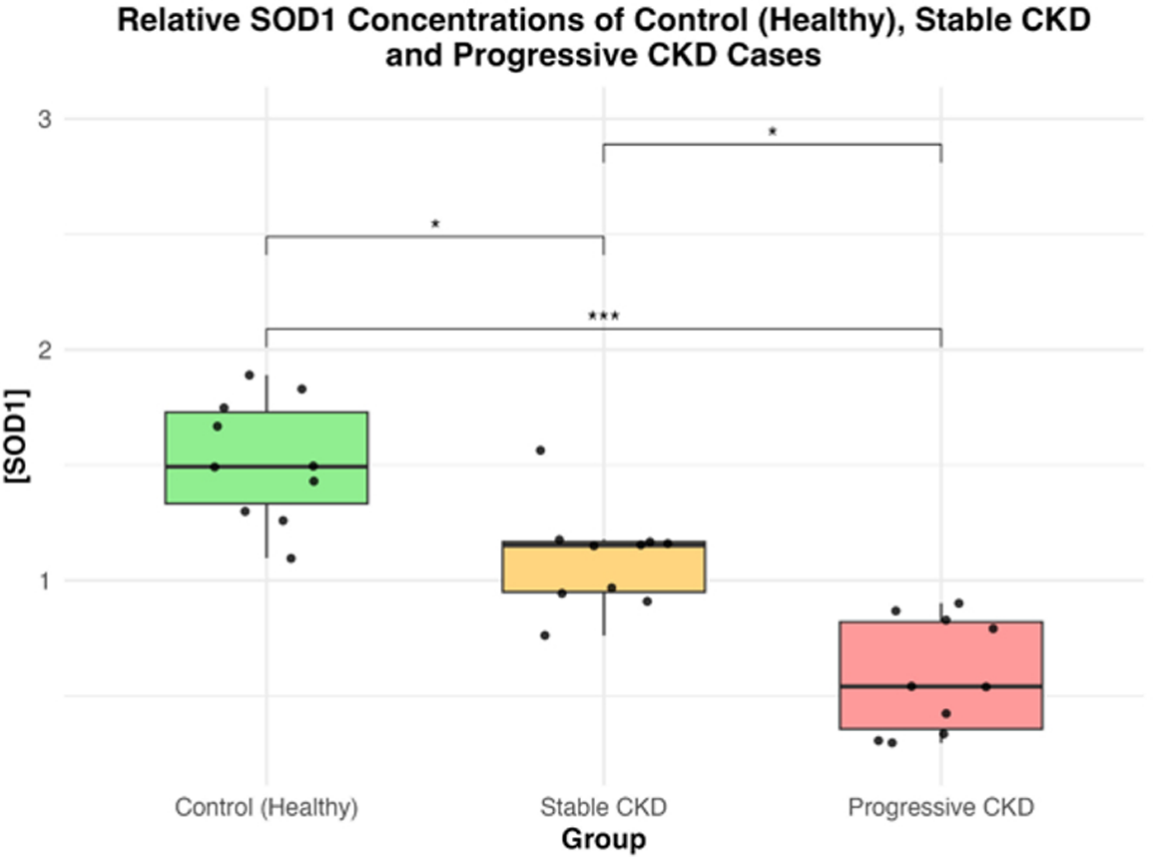
**Figure 1** Relative superoxide dismutase 1 (SOD 1) protein expressions in control, stable CKD, and progressive CKD cats, as determined by Western blotting.

**Figure:**
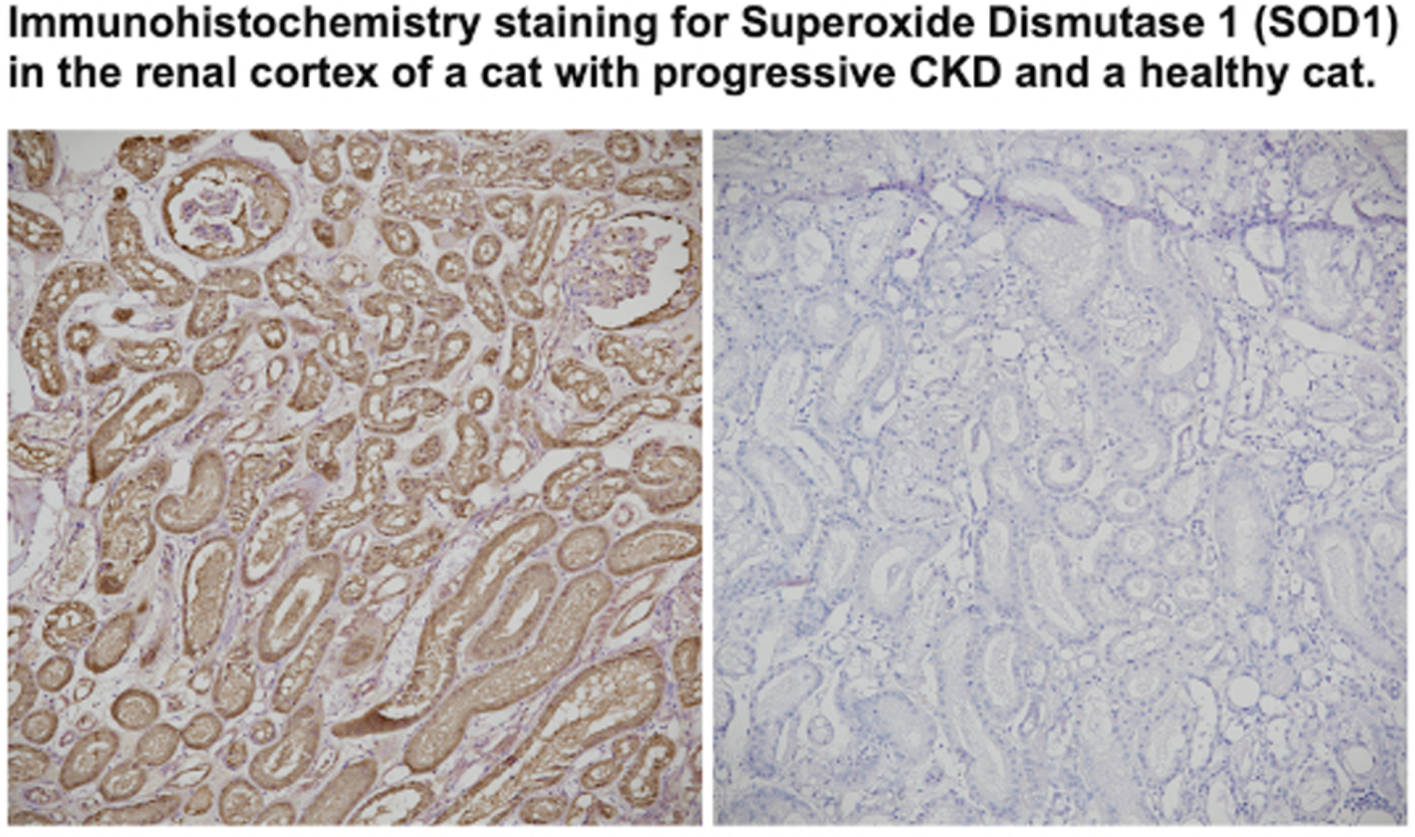
**Figure 2** 2A Immunohistochemistry staining reveals tubular epithelial superoxide dismutase 1 (SOD1) in renal cortex of a cat with progressive CKD. 40x magnification. 2B Negative control in the same section. 40x magnification.

## 255 ABSTRACT NU08: Age, Signalment, and Renal Markers Affect Risk of Increased Cystatin B in Dogs and Cats

### 255.1 **Helen Michael** ^1^; Edie Pallozzi^2^; Sarah Sweet^2^; Corie Drake^2^; Richard Mellanby^2^

#### 255.1.1 ^1^Director of Global Medical Affairs, IDEXX; ^2^IDEXX

**Background:** Accumulation of renal injury contributes to the development of chronic kidney disease. Renal functional markers, including creatinine and symmetric dimethylarginine (SDMA), are insensitive to kidney injury. Urinary cystatin B (cysB) is a marker of renal injury independent of functional changes.

**Objective/Hypothesis:** Evaluate effect of signalment and of renal functional markers on the likelihood of increased cysB in dogs and cats.

**Animals:** Retrospective data study of 208,440 dog and 237,500 cat clinical samples with cysB, creatinine, and SDMA results and signalment information submitted to IDEXX Laboratories submitted from September 1, 2023 to September 1, 2024.

**Methods:** ORs for predicting the likelihood and probability of increased cysB were calculated using logistic regression. ORs were calculated with young animals (dogs 1–3 years, cats 1–6 years), medium‐sized breeds (dog), male, and neutered animals as the reference groups.

**Results:** Increased cysB was more likely in adult (OR=1.47) and senior (OR=3.32) dogs, small (OR=1.3) and toy (OR=1.77) breeds, females (OR=1.16), and intact (OR=1.36) dogs. Increased SDMA (OR=3.89), increased creatinine (OR=3.11), and both (OR=15.3) were associated with high cysB (Figure 1). In cats, risk was higher in adults (OR=1.21), seniors (OR=1.90), females (1.11), and intact cats (1.27). Increased SDMA (OR=2.99) and concurrent SDMA and creatinine (OR=5.5) but not increased creatinine (OR=0.97) were associated with high cysB (Figure 2).

**Conclusions and Clinical Importance:** Age, sex, and neuter status impact likelihood of increased cysB. Increased creatinine was only associated with high cysB risk in dogs but SDMA and concurrent increases predicted higher risk in both.

**Figure:**
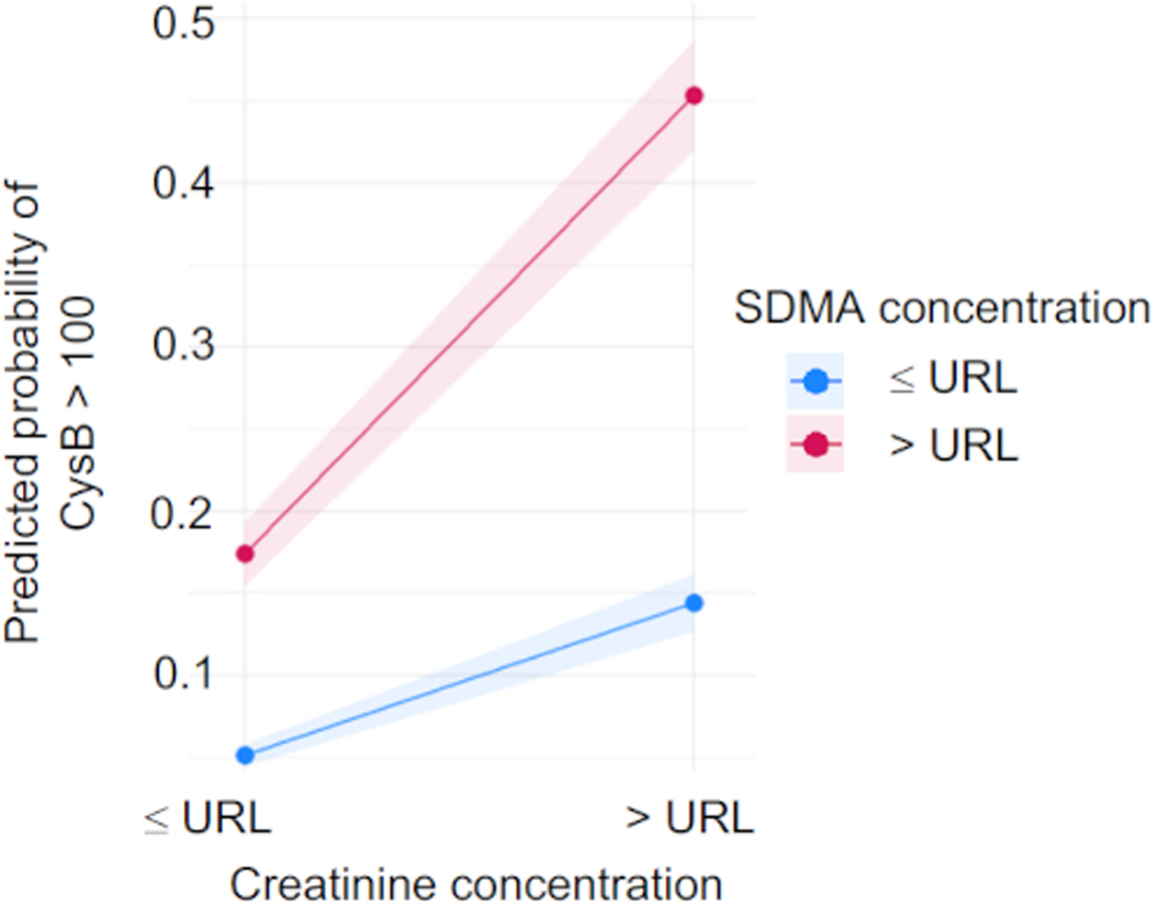
**Figure 1** Predicted probability of increased cystatin B (CysB) in dogs based on creatinine and SDMA concentration was ≤ or > upper reference limit (URL). Dogs had a higher predicted probability of increased CysB with isolated increases in SDMA and creatinine, and a markedly higher probability with concurrent increased SDMA and creatinine.

**Figure:**
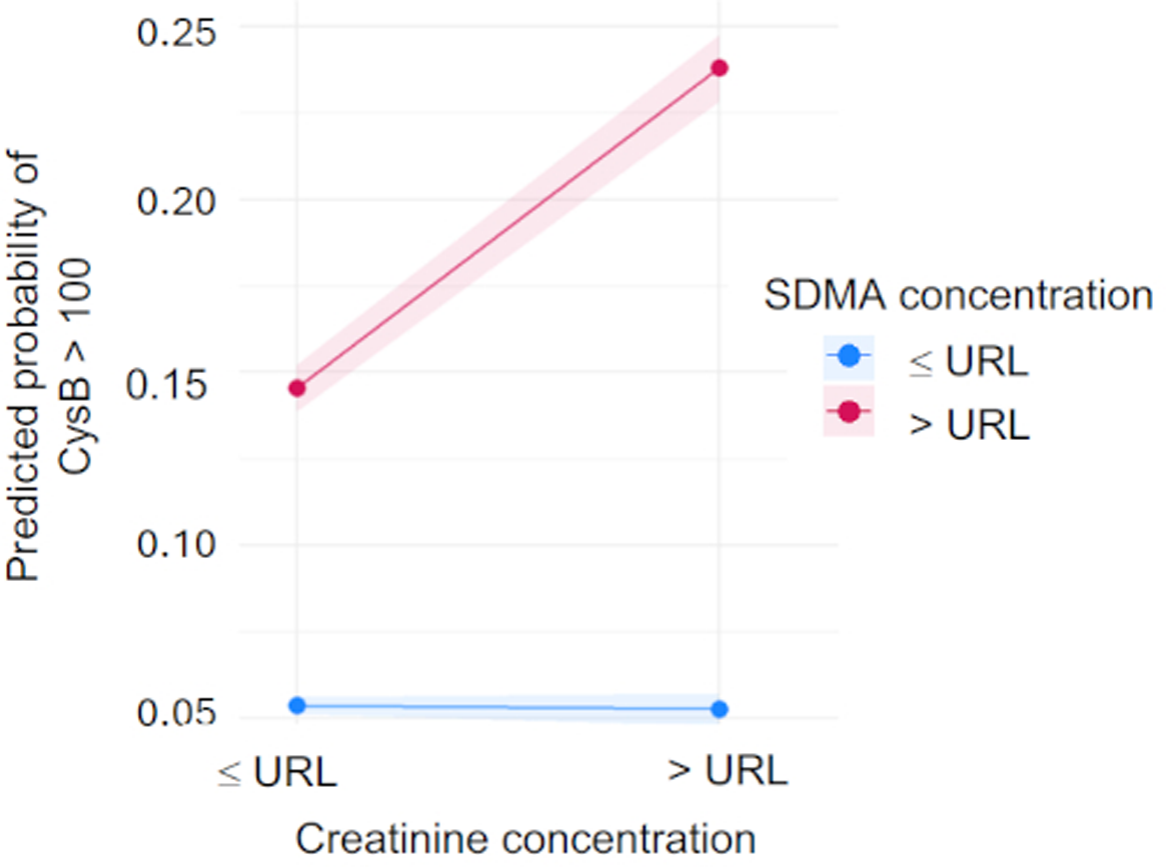
**Figure 2** Predicted probability of increased cystatin B (CysB) in cats based on creatinine and SDMA concentration was ≤ or > upper reference limit (URL). Increased SDMA with normal creatinine and concurrent increased SDMA and creatinine were associated with higher probability of increased CysB, but increased creatinine alone was not.

## 256 ABSTRACT NU09: Urinary Ammonia Excretion is Impaired in Dogs With Kidney Disease

### 256.1 **Autumn Harris** ^1^; Alexis Cooper^2^; Rebeca Castro^3^; Andrew Specht^4^, DVM, DACVIM (SAIM); Allison Kendall^3^, DVM, MS, DACVIM (SAIM); Shelly Vaden^5^, DVM, PhD, DACVIM (SAIM), Founding Member ACVNU; Kirsten Cooke^4^, DVM, DACVIM (SAIM)

#### 256.1.1 ^1^Associate Professor Nephrology‐Urology, North Carolina State University, Raleigh, NC, USA; ^2^University of Florida, Gainesville, FL, USA; ^3^Department of Clinical Sciences, North Caroline State University, Raleigh, NC, USA; ^4^Department of Small Animal Clinical Sciences, University of Florida, Gainesville, FL, USA; ^5^Professor Nephrology‐Urology, Department of Clinical Sciences, North Caroline State University, Raleigh, NC, USA

**Background:** Metabolic acidosis is common in dogs with chronic kidney disease (CKD). Inadequate ammonia excretion drives the development of metabolic acidosis in people with CKD and is correlated with worse clinical outcomes. Limited information exists about ammonia excretion in dogs with CKD.

**Hypothesis/Objectives:** Our objective was to determine if urine ammonia‐to‐creatinine ratio (UACR) correlates with serum creatinine as a renal function marker in healthy dogs and dogs with CKD and whether UACR is related to the presence of CKD.

**Animals:** The study population comprised 46 healthy and 49 stable IRIS stage II‐IV CKD dogs.

**Methods:** Serum biochemistry parameters were measured. Urinary ammonia and creatinine concentrations were measured using commercially available assays and used to calculate UACR. Group comparisons were made using the Mann‐Whitney Test. Correlation between UACR concentrations and serum renal and electrolyte values used Spearman's correlation test. Relationships between UACR, renal parameters, electrolytes, urine specific gravity, age, and body weight were explored with multiple linear regression.

**Results:** CKD dogs (median 2.2; range 0.9–5.8) had lower UACR than healthy dogs (median 7.1; range 0.7–40.0) (*p* < 0.0001). UACR was inversely correlated with creatinine concentration (*p* < 0.0001, rs=‐0.524). The relationship between UACR and creatinine persisted after controlling age, body weight, electrolytes, and urine‐specific gravity.

**Conclusions and Clinical Importance:** These findings suggest that ammonia excretion becomes impaired with declining renal function in dogs with kidney disease. Future research is warranted to determine if impaired ammonia excretion in dogs with kidney disease can provide prognostic information or become a therapeutic target.

## 257 ABSTRACT NU10: The Circulating and Intrarenal Renin‐Angiotensin(‐aldosterone) Systems in Cats With Renal Ischemia‐Induced Chronic Kidney Disease

### 257.1 **Jane Huang** ^1^, Bianca Lourenço^2^, DVM, MSc, PhD, DACVIM (SAIM); Chad Schmiedt^2^, DVM, DACVS; Amanda Coleman^2^, DVM, DACVIM

#### 257.1.1 ^1^PhD Student, University of Georgia, Athens, GA, USA; ^2^University of Georgia, Athens, GA, USA

**Background:** The relationship between the intrarenal renin‐angiotensin system (iRAS) and markers of the circulating renin‐angiotensin‐aldosterone system (cRAAS) in cats with chronic kidney disease (CKD) is understudied.

**Objectives:** To describe the correlation between iRAS and cRAAS markers in cats with surgically induced CKD.

**Animals:** 11 purpose‐bred cats with unilateral renal ischemia‐induced CKD and an undisturbed (*n* = 5) or nephrectomized (*n* = 6) contralateral kidney; and 8 healthy cats. All cats were aged 0.6–2.4 years.

**Methods:** Observational case‐control study. Serum and renal tissue equilibrium concentrations of angiotensin peptides and serum equilibrium concentrations of aldosterone were evaluated using liquid chromatography‐tandem mass spectrometry. Renal transcription levels of angiotensin‐converting enzyme (ACE), angiotensinogen (AGT), angiotensin II type 1 receptor (AT1R), and renin (REN) were quantified using reverse‐transcription polymerase chain reaction. Data were log‐transformed and correlations between each cRAAS and iRAS component were evaluated using Pearson correlation analysis.

**Results:** No correlation was found between circulating and tissular concentrations of angiotensin peptides (*r* = 0.36 for angiotensin I; *r* = 0.36 for angiotensin II; *r* = 0.07 for angiotensin III). Circulating angiotensin II was positively correlated with renal transcription levels of AGT (*r* = 0.51, *p* = 0.045), circulating angiotensin I was positively correlated with renal angiotensin II (*r* = 0.51, *p* = 0.044), and circulating aldosterone was negatively correlated with renal transcription levels of ACE (*r* = ‐0.60, *p* = 0.014).

**Conclusions and Clinical Importance:** Equilibrium concentrations of circulating angiotensin peptides or aldosterone did not reflect those of renal tissular angiotensin peptides or ACE, AGT, AT1R, and REN transcription levels. Evaluation of the iRAS requires renal tissue specimens or the development of other non‐invasive biomarker(s).

**Figure:**
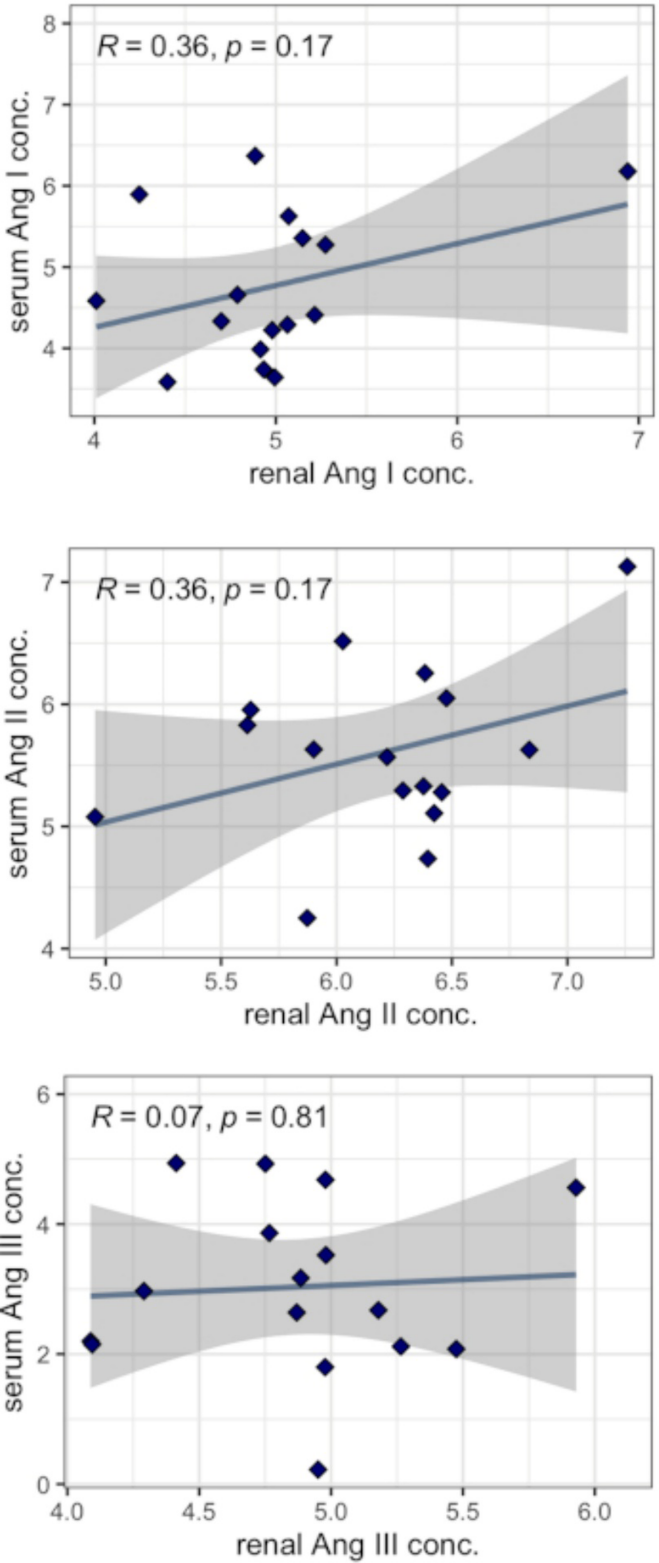
**Figure** Correlations between angiotensin equilibrium concentrations in serum and renal tissue. All values were log‐transformed. Correlation coefficient (R) and *p* values were calculated with Pearson's correlation analysis. Abbreviations: Ang, angiotensin; conc., concentration.

## 258 ABSTRACT NU11: Circulating Renin‐Angiotensin‐Aldosterone System Markers in Cats With Non‐hypertensive Chronic Kidney Disease or Systemic Arterial Hypertension

### 258.1 **Bianca Lourenço** ^1^; Jane Huang^2^, DVM, MSc; Lisa Reno^3^; Carl Toborowsky^3^, DVM, DACVIM (Cardiology); Amanda Coleman^4^, DVM, DACVIM (Cardiology)

#### 258.1.1 ^1^Assistant Professor of Internal Medicine, College of Veterinary Medicine, University of Georgia, Athens, GA, USA; ^2^PhD Student, Small Animal Medicine and Surgery, College of Veterinary Medicine, University of Georgia, Athens, GA, USA; ^3^College of Veterinary Medicine, University of Georgia, Athens, GA, USA; ^4^Small Animal Medicine and Surgery, College of Veterinary Medicine, University of Georgia, Athens, GA, USA

**Background:** Information on the classical and alternative pathways of the circulating renin‐angiotensin‐aldosterone system (cRAAS) in cats is limited.

**Objectives:** To describe and compare serum equilibrium concentrations of angiotensin peptides and aldosterone in apparently healthy cats and cats with azotemic, non‐hypertensive CKD (Azo‐NHT‐CKD) or untreated systemic arterial hypertension (SAH); and to characterize changes in these concentrations in hypertensive cats treated with amlodipine.

**Animals:** Client‐owned cats with Azo‐NHT‐CKD (serum creatinine ≥ 1.6 mg/dL, systemic arterial blood pressure [SBP] <160 mm Hg; *n* = 17), SAH (SBP ≥ 160 mm Hg or ≥ 150 mm Hg with hypertensive ocular lesions; *n* = 6), or normal SBP (<140 mm Hg) and kidney function (*n* = 17).

**Methods:** Prospective, single‐center, observational study. Cats underwent indirect SBP measurement and hematologic, serum, and urinary analyses to determine group assignment. Serum was obtained contemporaneously in all cats, and after 2–4 weeks of amlodipine therapy in cats with SAH, for evaluation of equilibrium concentrations of angiotensin I, II, III, IV, 1–5, and 1–7, and aldosterone using liquid chromatography‐tandem mass spectrometry.

**Results:** No differences in mean serum equilibrium concentration were detected between the Azo‐NHT‐CKD and control groups for any angiotensin peptide. Cats with untreated SAH had significantly lower mean concentrations of angiotensin I, II, III, IV, and 1–5 versus controls, and of angiotensin I and III versus the Azo‐NHT‐CKD group. Hypertensive cats treated with amlodipine experienced a significant increase in all angiotensin peptides. Serum aldosterone did not differ between groups, or before and after amlodipine in hypertensives.

**Conclusions and Clinical Importance:** Evidence of cRAAS activation in cats with Azo‐NHT‐CKD or untreated SAH was not found.

## 259 ABSTRACT NU12: Cats With a Subcutaneous Ureteral Bypass Device Locked With Saline or t‐EDTA

### 259.1 **Grégor Boot** ^1^; Marilyn Dunn^2^, DMV, MVSc, ACVIM, Fellow IR; Catherine Vachon^3^, DMV, DVSc, DACVIM, Fellow IR

#### 259.1.1 ^1^Specialized Intern in Interventional Medicine, Veterinary Teaching Hospital of Montreal University, Saint‐Hyacinthe, QC, Canada; ^2^Professor in Interventional Medicine, Interventional Medicine, Veterinary Hospital of the University of Montreal, Saint‐Hyacinthe, QC, Canada; ^3^Clinician in Internal Medicine and Interventional Medicine, Internal Medicine/Interventional Medicine, Veterinary Hospital of the University of Montreal, Saint‐Hyacinthe, QC, Canada

**Background:** Tetra EDTA (tEDTA), used as a locking solution in people with intravenous catheters decreases luminal obstruction and infection rates and may have similar effects following routine SUB flushes in cats.

**Hypothesis/Objective:** To compare the rate of positive urine culture, luminal obstruction and outcome in cats with SUBs undergoing routine flushing with sterile saline or tEDTA.

**Animals:** Cats with benign ureteral obstruction treated with a SUB between 2012 and 2024.

**Methods:** Retrospective study. Cats were evaluated every 3–4 months to monitor SUB patency by ultrasound‐guided flushes. Saline (2012–2017) or tEDTA 4% (2017–2024) was used as a locking solution.

**Results:** 81 cats with 116 bypassed ureters (BU) were included. 36 cats with 47 BU received saline and 45 cats with 69 BU received tEDTA. No significant difference was found for luminal obstruction between the saline (11/47, 23%) and tEDTA groups (25/69, 36%; *p* = 0.112). Median time to obstruction (418 and 469 days, respectively; *p* = 0.798) and rate of positive urine culture (12/36, 33% and 11/45, 24%, respectively; *p* = 0.67) were similar among the two groups. Total median follow‐up was 581 days for saline and 898 days for tEDTA (*p* = 0.068). In cats with luminal obstruction, an increase in serum creatinine was observed in 7/30 (23%) and a dilated renal pelvis in 10/36 (28%).

**Conclusions:** Routine use of tEDTA as a locking solution in cats with SUBs did not significantly decrease the rate of luminal obstruction nor positive urine cultures. Luminal obstruction was uncommonly associated with an increased creatinine or dilated renal pelvis.

## 260 ABSTRACT NU13: Subcutaneous Ureteral Bypass Explantation in 21 Cats (2015–2023): Outcomes and Owner Perceptions

### 260.1 **Sophie Broughton** ^1^; Anna Frykfors von Hekkel^2^, BVetMed (Hons), PGDVCP, MVetMed, PGCertVetEd, DECVS, FHEA, MRCVS; Matteo Rossanese^2^, DVM, SPSA, CertAVP, MSc, DECVS, FRCVS; Lynda Rutherford^2^, BVM&S, MVetMed, DECVS, PGCertVetEd, FHEA, MRCVS; Rebecca Geddes^2^, MA, VetMB, GPCert(FelP), MVetMed, PhD, DACVIM, FHEA, MRCVS; Jack Lawson^2^, BVetMed, MVetMed, PhD, DACVIM, DECVIM, PgCertVetEd, MRCVS

#### 260.1.1 ^1^PhD Student, Royal Veterinary College, London, UK; ^2^Royal Veterinary College, London, UK

**Background:** Subcutaneous ureteral bypass (SUB) is the gold standard for feline ureteric obstruction management, but SUB explantation is little documented.

**Hypothesis/Objectives:** Describe reasons for SUB explantation, clinical outcomes and owner experiences.

**Animals:** Twenty‐one cats that underwent SUB explantation.

**Methods:** Retrospective case‐series. Data from 21 cats that underwent SUB explantation between 10/15–08/23 analysed. Twenty owners contacted for follow‐up.

**Results:** SUB placement was mostly associated with ureterolithiasis (16/21). Explantation was performed a median of 1.8 y (range 0.2–4.1) later, most commonly for positive urine culture (15/21) followed by SUB obstruction (8/21), sterile cystitis (4/21), suspected SUB failure (2/21), and one transmural migration to the gastrointestinal tract (two further cats had transmural migration noted intraoperatively). Contrast studies documented that 26/31 ureters had regained patency. Seventeen cats had all implants removed (9 unilateral, 8 bilateral). Two underwent unilateral explantation and ureteronephrectomy. One underwent unilateral explantation despite bilateral SUBs and bilateral ureteral patency, and one underwent unilateral explantation and contralateral replacement. Median follow‐up was 596 days (3–2310). Four cats had ureteral re‐obstruction (one had a partial obstruction at explantation). Ten cats did not re‐obstruct and the remainder were undetermined. Seven cats died, at a median time of 614 days (405–2200). Four deaths were related to renal disease (two re‐obstructions). Questionnaire response was 45% (9/20), and 8/9 owners would consider explantation if in the same position in future.

**Conclusions and Clinical Importance:** For SUB cats with complications, explantation appears a viable option. Most ureters re‐gained patency before explantation with re‐obstruction only documented in 4/21. Most responding owners were satisfied with the outcome.

## 261 ABSTRACT NU14: Investigating Serum Growth Differentiation Factor‐8 Concentrations in Cats With Early Chronic Kidney Disease

### 261.1 **Kerrigan Fleming** (Veterinary Student); William Whitehouse

#### 261.1.1 Purdue University, IN, USA

**Background:** Growth differentiation factor‐8 (GDF‐8) is a negative regulator of muscle growth and is increased in people with chronic kidney disease (CKD). It has not been evaluated in cats with CKD.

**Objectives:** The objective of this study is to evaluate if the concentrations of GDF‐8 in cats with early International Renal Interest Society (IRIS) stages of CKD are increased compared to healthy cats.

**Animals:** Fifteen cats with CKD (5 IRIS stage 1, 10 IRIS stage 2) and 10 healthy control cats.

**Methods:** Retrospective cross‐sectional study. Serum GDF‐8 was measured using a commercially available multispecies sandwich enzyme‐linked immunosorbent assay. Associations of GDF‐8 concentrations with age, sex, body weight, body condition score, muscle condition score (MCS), creatinine, blood urea nitrogen, symmetric dimethylarginine, phosphorus, and urine specific gravity were also examined.

**Results:** GDF‐8 was not different amongst healthy cats (2137±740 pg/mL) and cats with IRIS stage 1 (1785±530 pg/mL) and IRIS stage 2 (1961±638 pg/mL; *P* = 0.608) CKD. GDF‐8 was negatively correlated to MCS (*r* = –0.517, *P* = 0.049), but no association was found between GDF‐8 and the other selected renal parameters. However, age was significantly higher in IRIS stage 2 CKD cats compared to the healthy cats (*P* = 0.036), and GDF‐8 was negatively correlated with age (*r* = –0.429, *P* = 0.032).

**Conclusions and Clinical Importance:** GDF‐8 could be a marker for muscle mass. Further evaluation of the functional role of GDF‐8 with age and CKD in cats is warranted.

## 262 ABSTRACT NU15: Renal Cortical Thickness to Aortic Diameter Ratio: A Novel Ultrasound Marker for Feline Kidney Disease

### 262.1 **Andre Meneses** ^1^, DVM, PhD; Adriana Belas^2^, Maria Catarina Caldeira^3^; Vanessa Duarte^2^; Joana Fonseca^2^; Sabrina Legatti^2^; Joana Oliveira^2^; Ana Santana^2^; Isabella Sposito^3^; Rute Teixeira^2^

#### 262.1.1 ^1^Auxiliary Professor, Faculty of Veterinary Medicine, Lusofona University of Lisbon, Lisbon, Portugal; ^2^Professor, Faculty of Veterinary Medicine, Lusofona University, Lisbon, Portugal; ^3^Student, Faculty of Veterinary Medicine, Lusofona University, Lisbon, Portugal

Feline kidney disease, including chronic kidney disease (CKD) and acute kidney injury (AKI), is a major concern in veterinary medicine. Despite the availability of diagnostic tools such as serum biomarkers and ultrasound, there is a need for novel, practical, and reliable methods to distinguish between healthy cats and those with renal disease. This study introduces the renal cortical thickness to abdominal aortic diameter ratio (RCA/Ao) as a noninvasive, quantitative ultrasound marker for CKD and AKI in cats. To evaluate the RCA/Ao ratio as a diagnostic marker for renal disease and assess its correlation with serum biomarkers. A total of 105 client‐owned cats were enrolled: Healthy (*n* = 50), CKD (*n* = 44), and AKI (*n* = 11). Sagittal ultrasound images of the kidneys and aorta were obtained. Renal cortical thickness was measured at three locations, while aortic diameter was measured at systole, caudal to the left renal artery bifurcation. Serum biomarkers (BUN, phosphorus, creatinine, SDMA) were analyzed. The RCA/Ao ratio was: 1.04±0.32 cm (healthy), 1.90±0.77 cm (CKD), and 1.97±0.85 cm (AKI). A cutoff of ≥ 1.4 cm had 81.82% sensitivity and 86% specificity for detecting renal disease. This study introduces RCA/Ao as a novel, quantitative, noninvasive diagnostic tool for feline kidney disease. Its simplicity, reproducibility, and strong correlation with renal biomarkers support its inclusion in veterinary diagnostic protocols, aiding early detection and disease management.

## 263 ABSTRACT NU16: Retrospective Study of Cystoscopic‐Guided Laser Ablation of Intramural Ectopic Ureters in Male Dogs

### 263.1 **Josh Chang**, DVM (Resident); Allyson Berent, DVM, DACVIM; Chick Weisse, DVM, DACVS

#### 263.1.1 Schwarzman Animal Medical Center

**Background:** To report the use of cystoscopic‐guided laser ablation (CLA) for ectopic ureters (EU) in male dogs.

**Hypothesis/Objective:** To retrospectively describe the long‐term outcomes using CLA for correction of intramural EU in a larger cohort of male dogs than previously reported.

**Animals:** Eighteen client‐owned male dogs

**Methods:** Medical records of male dogs that had CLA‐EU performed were retrospectively reviewed. Continence scores were assigned (out of 10) before and after the procedure, and after additional intervention. Data collected included signalment, neuter status, age of onset of urinary incontinence, pre‐ and post‐operative continence scores, complete blood count, serum biochemistry, urinalysis and microbiological culture, preoperative abdominal imaging, EU laterality, concurrent urinary tract anatomic anomalies, laser type/size used, procedure time, complications, and follow‐up time.

**Results:** The CLA‐EU procedure was technically successful in all dogs. Four (22%) dogs were continent at the time of presentation. Postoperative continence scores were significantly improved from preoperative (*P* = 0.001). Of the 14 that were initially incontinent, 11(79%) became continent without any additional intervention, and 86% with the addition of medications, resulting in 89% of dogs completely continent at the end of the study period. The presence of preoperative EU orifice stenosis (*P* = 0.04), hydronephrosis (*P* = 0.02), hydroureter (*P* = 0.03), or a normal urethral diameter (*P* = 0.0009) were significantly associated with a better postoperative continence score. The median follow‐up time was 1789 days (range, 98–3560).

**Conclusion and Clinical Importance:** CLA‐EU in male dogs was a safe, minimally invasive, and effective procedure with good short‐ and long‐term outcomes, achieving continence in most dogs.

**Table:** **Table 1.** Continence score pre vs post CLA‐EU

Male dogs	Continence score pre‐CLA‐EU	Continence score post CLA‐EU without any intervention	Continence score with addition of meds	Continence score with additional U‐Coil procedure
1	5	10		
2	5	10		
3	10	10		
4	1	10		
5	10	10		
6	2.5	2.5	9	
7	10	10		
8	1	10		
9	2.5	2.5	2.5	2.5
10	7.5	10		
11	5	10		
12	2.5	10		
13	10	10		
14	5	10		
15	5	10		
16	5	10		
17	1	1		
18	7.5	10		

Abbreviations: CLA; cytoscopic‐guided lase ablation, EU; ectopic ureter, U‐Coil; urethral coil.

**Table:** **Table 2.** Urinary continence scoring system (Berent et al. 2012, Burdick et al. 2014)

Urinary continence score	Description
1	Leaking always and does not accumulate urine in the bladder at rest. Fully incontinent.
2.5	Leaking at all times: running, playing, or lying down. Can urinate a stream of urine and has urine accumulating in the bladder at rest.
5	Leaking mainly when lying down, minimally when walking or playing, may leak immediately before or after urination.
7.5	Only leaking when resting and lying down.
9	No puddles or urine noted. Urine only detected to leak on fur/around prepuce few times a week.
10	No leaking at all. Fully continent

## 264 ABSTRACT NU17: Evaluation of Oxidative Stress in Nonazotemic Cats With Increased Symmetric Dimethylarginine Concentrations

### 264.1 **William Whitehouse** ^1^, DVM, DACVIM (SAIM); Nicky Cassel^2^, BVSc, MMedVet, DECVDI

#### 264.1.1 ^1^Assistant Professor, Purdue University, IN, USA; ^2^Kansas State University, KS, USA

**Background:** Oxidative stress might be present in cats with early chronic kidney disease (CKD), but it has not been evaluated in cats with increased symmetric dimethylarginine (SDMA) concentrations as the primary index of early CKD.

**Objectives:** The primary objective was to determine if urinary F2‐Isoprostanes (F2‐IsoPs) are increased in cats with a normal serum creatinine but increased SDMA compared to healthy controls. Secondary objectives were to evaluate plasma F2‐IsoPs and renal resistive index (RRI) in these cohorts.

**Animals:** Eight cats with early CKD and 14 healthy control cats (> 8 years old).

**Methods:** Urinary and plasma F2‐IsoP concentrations were measured by gas chromatography/negative ion chemical ionization mass spectrometry. Spectral Doppler was used to acquire RRI values.

**Results:** Median absolute urinary F2‐IsoPs were significantly lower in cats with early CKD (0.402 ng/mL, range 0.113–3.306) compared to controls (1.738 ng/mL, range 0.770–3.183, *P* = 0.029). When urinary F2‐IsoPs were normalized to urine creatinine, there was no difference between early CKD cats (0.286 ng/mg, range 0.163–1.45) and controls (0.667, range 0.416–1.43, *P* = 0.057). There was no difference in median free plasma F2‐IsoPs in cats with early CKD (0.038, range 0.003–0.086) compared to controls (0.106 ng/mL, range 0.003–0.198, *P* = 0.111). Left kidney RRI was higher in early CKD cats (*P* = 0.026) but not with right kidney or averaged kidney RRI values compared to controls.

**Conclusions and Clinical Importance:** Urinary F2‐IsoPs are not increased in cats with a normal serum creatinine but increased SDMA. RRI does not reliably detect early CKD.

## 265 ABSTRACT NU18: Quantitative and Qualitative Analysis of Renal Proteinuria in 25 Dogs With Spontaneous Chronic Kidney Disease

### 265.1 **Luciano Giovaninni** ^1^, DVM, PhD; Douglas Caragelasco^2^; Fernando Carvalho^3^; Cinthia Martorelli^3^

#### 265.1.1 ^1^Veterinary, UnicPet Nefrology and Urology in Dogs and Cats; ^2^Faculdade de Medicina Veterinária, Pontificie Universidade Católica de Campinas; ^3^UnicPet Nephrology and Urology

In dogs, glomerular proteinuria may result from systemic hypertension, glomerular hyperfiltration, or glomerulonephritis—conditions typically managed with angiotensin‐converting enzyme inhibitors or angiotensin receptor blockers. However, the urinary protein‐to‐creatinine ratio (UPC), widely used in clinical settings, cannot differentiate the origin of renal proteinuria (RP) —whether glomerular, tubular, or mixed. SDS‐PAGE urinary protein electrophoresis has emerged as a promising tool to qualitatively identify the predominant origin of RP by distinguishing glomerular (GEP), tubular (TEP), or mixed (MEP) electrophoretic patterns, potentially supporting more appropriate therapeutic decisions. This study aimed to demonstrate that relying solely on UPC may result in suboptimal treatment of RP. Twenty‐five dogs with renal proteinuria and chronic kidney disease (CKD), stages 1 to 4 (11 males, 14 females; mean age 9.8±2.9 years; various breeds), were evaluated. UPC values were compared to SDS‐PAGE electrophoretic patterns. MEP was the most frequently observed pattern (68.0%, *n* = 17), with a mean UPC of 8.8±8.4. TEP and GEP were each observed in four dogs (16.0%), with mean UPCs of 6.7±3.5 and 4.3±3.2, respectively. In GEP cases, the predominant protein band was observed between 60–70 kDa, likely representing albumin. Importantly, no bands were detected between 90–100 kDa, which could correspond to Tamm‐Horsfall protein. These findings suggest that MEP can be predominant in dogs with RP. Therefore, a more accurate identification of RP origin may require both quantitative (UPC) and qualitative (SDS‐PAGE) assessments. This combined approach could enhance the localization of nephron damage and guide more effective treatment strategies in dogs with renal proteinuria.

## 266 ABSTRACT NU19: Elevated Trimethylamine N‐Oxide Levels in Canine Chronic Kidney Disease: TMAO as a Potential Renal Biomarker

### 266.1 **Seungju Kang** ^1^; Chang‐Hyeon Choi^2^, DVM; Yoon Jung Do^2^; Keon Kim^2^, DVM; Wan‐gyu Kim^2^, DVM; Chang‐min Lee^2^ (Professor); Jong‐Hwan Park^2^ (Professor); Woong‐bin Ro^2^, (Professor)

#### 266.1.1 ^1^Graduate Student of Veterinary Medicine, Veterinary Researcher, Veterinary Medicine, Chonnam National University, South Korea; ^2^Chonnam National University, South Korea

**Background:** Trimethylamine N‐oxide(TMAO) is known as a biomarker for cardiovascular diseases in humans. Recently, it has been reported to increase in cases of renal failure in humans and is recognized as a biomarker of kidney injury in mouse model studies.

**Objective:** To evaluate the utility and potential of TMAO as a supportive biomarker for the diagnostic and prognostic of canine chronic kidney disease (CKD).

**Animals:** The CKD group (*n* = 26) consisted of dogs with CKD as the chief complaint, selected retrospectively. For detailed analysis, the CKD group was classified into stage 2 (*n* = 11), Stage 3 (*n* = 8), and stage 4 (*n* = 7) according to the IRIS guidelines. The control group (*n* = 25) consisted of healthy dogs with no evidence of CKD, confirmed through medical history, physical examination, blood tests, and imaging studies.

**Methods:** Serum TMAO levels were quantified using enzyme‐linked immunosorbent assay (ELISA) methods.

**Results:** Dogs with CKD exhibited significantly higher circulating TMAO levels compared to the control group (*p* < 0.0001). As CKD stage advanced, median TMAO concentrations tended to increase, and each stage showed a statistically significant difference compared to healthy controls (control vs CKD 2; *p* < 0.0015, control vs CKD 3; *p* < 0.0001, control vs CKD 4; *p* < 0.0001). Additionally, at the 6‐month follow‐up, TMAO concentrations were significantly higher in non‐survivors compared to survivors within the CKD group (*p* = 0.0020).

**Conclusions and Clinical Importance:** This pilot study demonstrates the potential diagnostic and prognostic utility of TMAO as a novel biomarker in canine CKD.

## 267 ABSTRACT NU20: Urinary Calcium and Urinary Magnesium in Cats: A Validation Study

### 267.1 **Victória Gomes** ^1^; Ammanuel Ouba^2^; Matheus Leite^2^; Clara Mori^2^; Juliana Conceição^2^; Danielle Santos^2^; Juliana de Carvalho^2^; Fernanda Chacar^2^

#### 267.1.1 ^1^Veterinary, School of Veterinary Medicine and Animal Science, University of Sao Paulo, Sao Paulo, Brazil; ^2^Faculty of Veterinary Medicine and Animal Science (FMVZ), University of Sao Paulo, Sao Paulo, Brazil

**Background:** The measurement of electrolytes in urine may be useful in the diagnosis and management of urolithiasis or tubular disorders; however, a validation study in cats is needed.

**Objective:** To validate a colorimetric method for measuring urinary calcium and magnesium concentrations in cats.

**Animals:** Urine samples of cats treated at the Veterinary Teaching Hospital of the University of Sao Paulo.

**Methods:** Urine samples were stored at –80°C. After thawing, samples were pooled (50 mL), and an acidified solution (AS; 1 mL urine:10 μL of 10% nitric acid) and a non‐acidified solution (NAS) were prepared. Ten consecutive measurements of calcium and magnesium were performed for intra‐assay; measurements along five consecutive days were performed for inter‐assay. Coefficient of variation (CV) <10% was considered satisfactory.

**Results:** Inter‐assay CVs were: NAS magnesium (16,53%; 3,73%; 1,72%; 1,73% 1,29%), AS magnesium (2,63%, 1,69%, 2,42%, 1,90%, 2,40%), NAS calcium (6,92%; 1,79%; 4,72%; 2,61%; 2,65%) and AS calcium (1,81%; 2,30%; 2,46%; 1,67%; 1,40%). Differences were found in the intra‐assay of magnesium using AS (*P* < 0.05). There were no differences in the inter‐assay of urinary magnesium between AS and NAS. Regarding urinary calcium, differences were found in the inter‐assay between AS and NAS (*P* < 0.0001).

**Conclusions and Clinical Importance:** Urinary calcium measurement should be performed in acidified samples, and magnesium measurement in non‐acidified samples. The colorimetric method is inexpensive and widely used, which may support the use of urinary electrolyte evaluation as a diagnostic and therapeutic monitoring tool.

**Figure:**
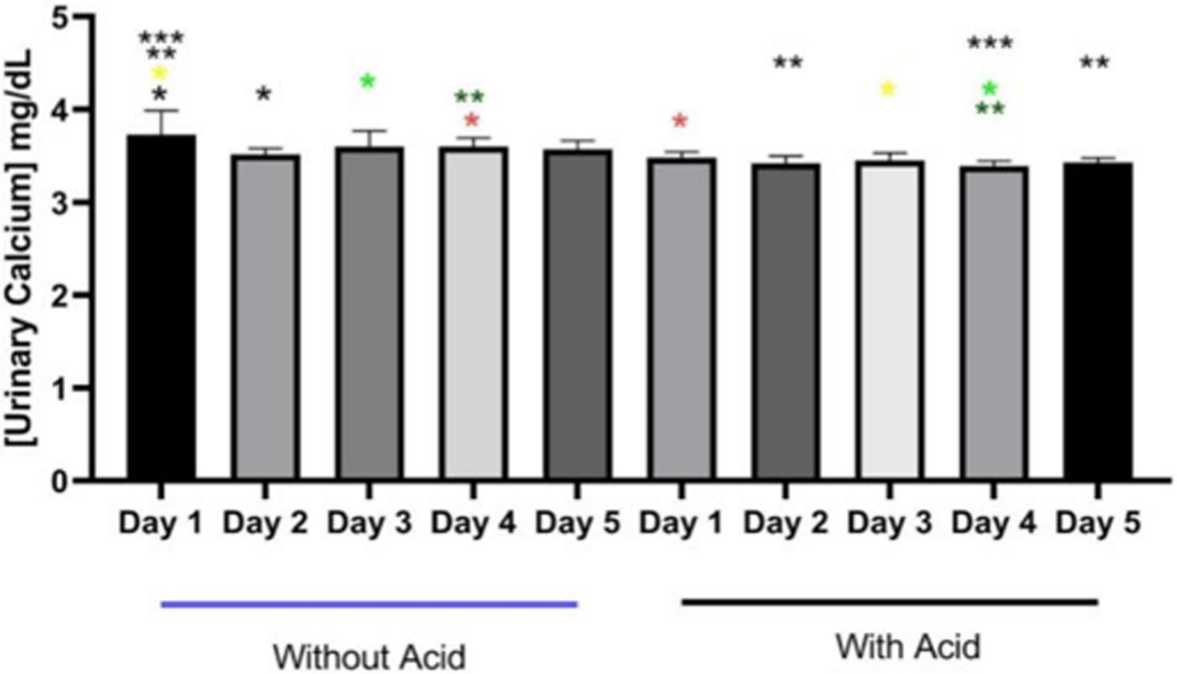
**Figure 1. Mean, standard deviation and statistical difference of urinary calcium measurement in non‐acidified (NAS) and acidified (AS) samples on days 1 to 5**. *p < 0.05; **p < 0.01; ***<0.0001

**Figure:**
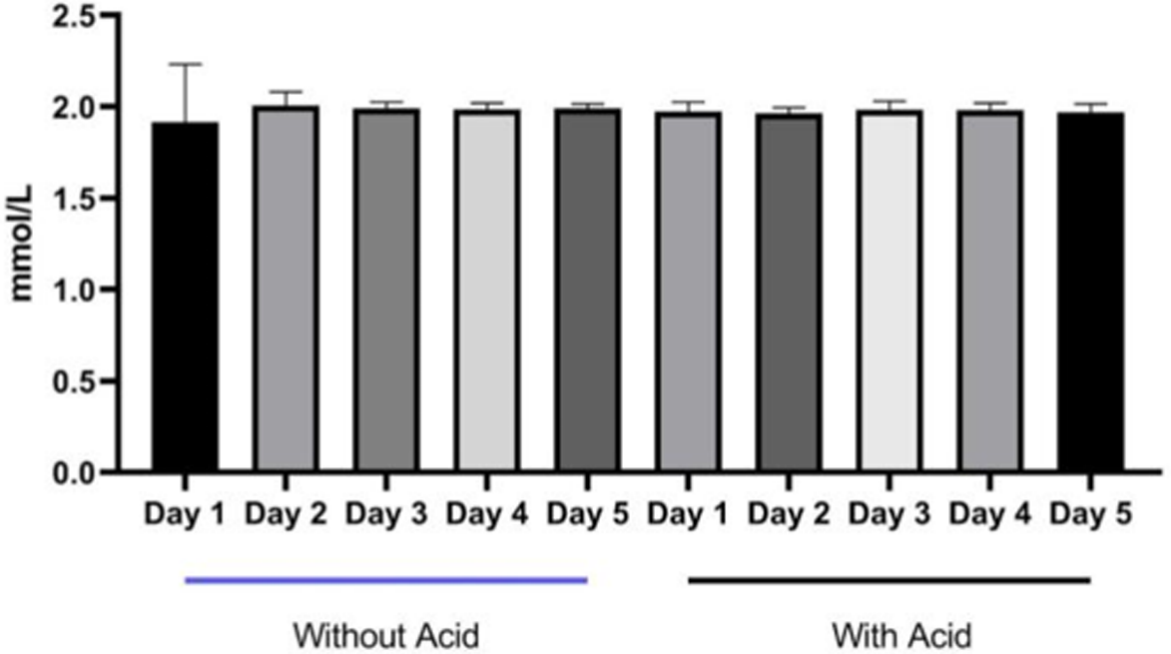
Figure 2. Mean, standard deviation and statistical difference of urinary magnesium measurement in non‐acidified (NAS) and acidified (AS) samples on days 1 to 5

## 268 ABSTRACT NU21: The Co‐occurrence Between Nephrocalcinosis and Nephrolithiasis in Feline Kidneys

### 268.1 **Nuttha Hengtrakul**, DVM; Jody Lulich

#### 268.1.1 Graduate Student, University of Minnesota

**Background:** Nephrocalcinosis and nephrolithiasis are common findings in feline kidneys, but their relationship to each other remains poorly understood. Understanding these associations is essential for developing effective therapeutic approaches to feline nephrocalcinosis and nephrolithiasis.

**Hypothesis/Objectives:** To evaluate the co‐occurrence between nephrocalcinosis and nephrolithiasis in feline kidneys.

**Animals:** Kidneys from 175 client‐owned cats were collected post‐mortem within 24 h of euthanasia.

**Methods:** In this cross‐sectional study, high‐resolution microradiography was used to classify nephrocalcinosis patterns in kidneys with and without nephroliths. Nephroliths from select kidneys were retrieved for validation and compositional analysis (IR). Fisher's exact and chi‐square tests were used to assess associations. Logistic regression was used to evaluate predictors of nephrolithiasis.

**Results:** Nephrocalcinosis was observed in 84% (147/175) of cats, and nephroliths were identified in 54.3% (95/175). 59.5% of cats with nephrocalcinosis had nephroliths, compared to 25% of cats without nephrocalcinosis (χ^2^=10.2, *p* = 0.001). 350 kidneys were independently evaluated; 92.7% (267) had striated nephrocalcinosis, 26% (75) had punctate nephrocalcinosis, and 17.7% (62) without nephrocalcinosis; nephroliths were identified in 49%, 53%, and 12.9% respectively. In univariate analysis, nephrocalcinosis was associated with a high risk of nephrolithiasis (OR=3.45, 95% CI=1.60–7.44, *p* = 0.002, *n* = 175). Cat age was a significant risk factor for nephrolithiasis (OR=1.15, 95% CI=1.05–1.25, *p* = 0.001, *n* = 115).

**Conclusions and Clinical Importance:** Nephrocalcinosis is a significant risk factor for nephrolithiasis. The underlying shared pathology requires further study.

**Figure:**
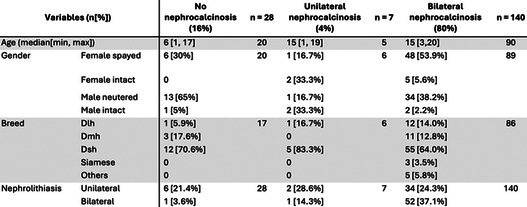
Table 1. Characteristics of cats with no nephrocalcinosis, unilateral nephrocalcinosis, and bilateral nephrocalcinosis

**Figure:**
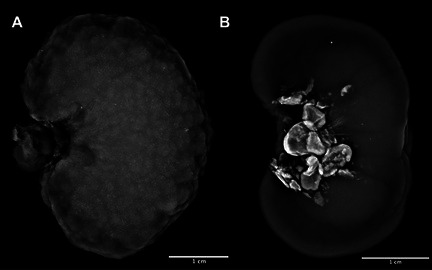
**Figure 1. Microradiographs of feline kidneys displaying distinct mineralization patterns and nephroliths**. (A) Kidney with diffuse punctate mineralization as the major pattern, characterized by scattered radiopaque foci throughout the parenchyma. (B) Kidney with diffuse striation pattern, exhibiting parallel radiopaque lines along the renal parenchyma and multiple nephroliths localized in the renal pelvis.

## 269 ABSTRACT NU22: Urinary Calcium, Magnesium, and Phosphorus in Dogs: A Validation Study

### 269.1 **Matheus Leite**; Ammanuel Ouba; Victoria Gomes; Clara Mori; Danielle Santos; Juliana de Carvalho; Fernanda Chacar

#### 269.1.1 School of Veterinary Medicine and Animal Science (FMVZ), University of São Paulo (USP)

**Background:** Urinary calcium, magnesium, and phosphorus evaluation may be useful in diagnosing urinary tract disorders. However, to the best of our knowledge, validation of the colorimetric assay is not currently available.

**Objective:** To validate a colorimetric method for measuring urinary calcium, magnesium, and phosphorus in dogs.

**Animals:** Urine samples of dogs treated at the Veterinary Teaching Hospital of the University of São Paulo. Samples with color abnormalities or cloudiness were excluded.

**Methods:** Samples were stored at −80°C, thawed, pooled, then acidified (AC) with 10 μl of 10% nitric (calcium; magnesium) or hydrochloric acid (phosphorus) per 1 ml of urine; or kept non‐acidified (NA). Ten measurements were performed daily over five consecutive days using an automatic biochemistry analyzer.

**Results:** All protocols showed satisfactory coefficients of variation (<10%): NA calcium (0.69%, 2.02%, 3.31%, 2.83%, 2.07%), magnesium (1.11%, 1.06%, 1.01%, 1.22%, 0.55%), phosphorus (1.92%, 3.34%, 2.42%, 1.19%, 1.51%); AC calcium (1.49%, 1.22%, 1.44%, 1.72%, 3.86%), magnesium (4.59%, 0.92%, 0.98%, 0.72%,1.00%), phosphorus (2.05%, 1.91%, 2.33%, 2.87%, 2.67%). Inter‐assay analysis revealed differences across days in all subgroups: NA and AC calcium (*p* < 0.0001); NA and AC magnesium (*p* < 0.0001); NA and AC phosphorus (*p* < 0.001).

**Conclusions and Clinical Importance:** AC and NA samples may be used to measure urinary calcium, magnesium, and phosphorus by the colorimetric method in dogs. This method is inexpensive and widely used, which may facilitate dissemination of urinary electrolyte assessment as a diagnostic and therapeutic monitoring tool.

**Figure:**
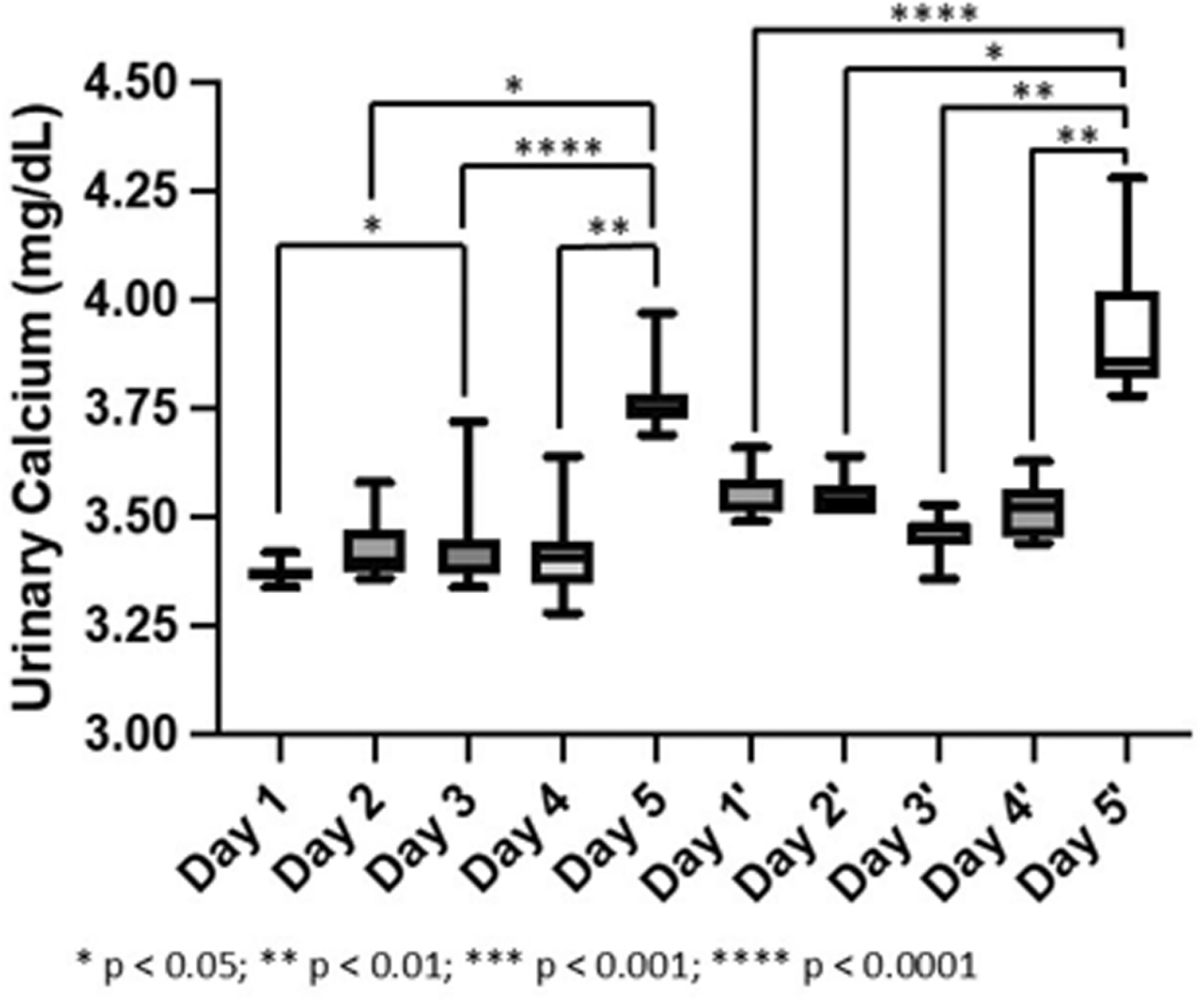
Urinary calcium measurements in non‐acidified and acidified (with apostrophe) samples with statistical differences

**Figure:**
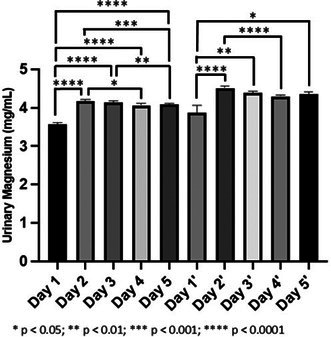
Urinary magnesium measurements in non‐acidified and acidified (with apostrophe) samples with statistical differences

## 270 ABSTRACT NU23: Assessment of an Evolved Deep Learning Artificial Intelligence Algorithm for Canine/Feline Urine Sediment Samples

### 270.1 **Cory Penn** ^1^, DVM; Mary Lewis^2^; Anonda Haskin^2^; Dan Lin^3^; Eric Morissette^2^; Kristin Owens^2^

#### 270.1.1 ^1^Vetscan Imagyst, Immunoassay, and Virtual Laboratory, Medical Affairs, Zoetis Global Diagnostics; ^2^Zoetis; ^3^Analitix Giant Clinical Research Co. Ltd

**Background:** Urine sediment exam is vital to a complete urinalysis. A deep learning artificial intelligence (AI) tool for consistent evaluation of urine sediment samples can provide rapid, in‐clinic results.

**Hypothesis/Objectives:** Vetscan Imagyst® AI Urine Sediment v2.0 will accurately identify red blood cells, white blood cells, struvite crystals, calcium oxalate dihydrate crystals, and rod‐shaped and cocci bacteria in agreement with digital review by ACVP‐boarded clinical pathologists (ACVP‐CPs).

**Animals:** Urine samples included client‐owned dogs and cats undergoing urinalysis for any reason submitted to Zoetis Reference Laboratories. Some samples were artificially created by spiking donor urine with necessary elements (e.g., RBC from whole blood). A total of 175 urine samples (canine, 56%; feline, 44%) were evaluated.

**Methods:** Samples were prepped via the Vetscan Imagyst® preparation method, scanned, and evaluated digitally by 3 blinded ACVP‐CPs who recorded results for urine elements as an average of 10, 40X fields. Algorithm performance and agreement were calculated as compared to ACVP‐CP results.

**Results:** Accuracy, sensitivity, and specificity for urine sediment object classes ranged from 91–95%, 89–95%, and 85–96%, respectively (Table 1). Agreement between Vetscan Imagyst® AI Urine Sediment v2.0 and ACVP‐CPs was good to excellent (Table 2).

**Conclusions and Clinical Importance:** Vetscan Imagyst® AI Urine Sediment v2.0 was comparable to ACVP‐CPs in the identification and semi‐quantitative agreement of elements in urine sediment. In‐clinic utilization of Vetscan Imagyst® AI Urine Sediment v2.0 can provide a diagnostic tool for urine sediment evaluation.

**Figure:**
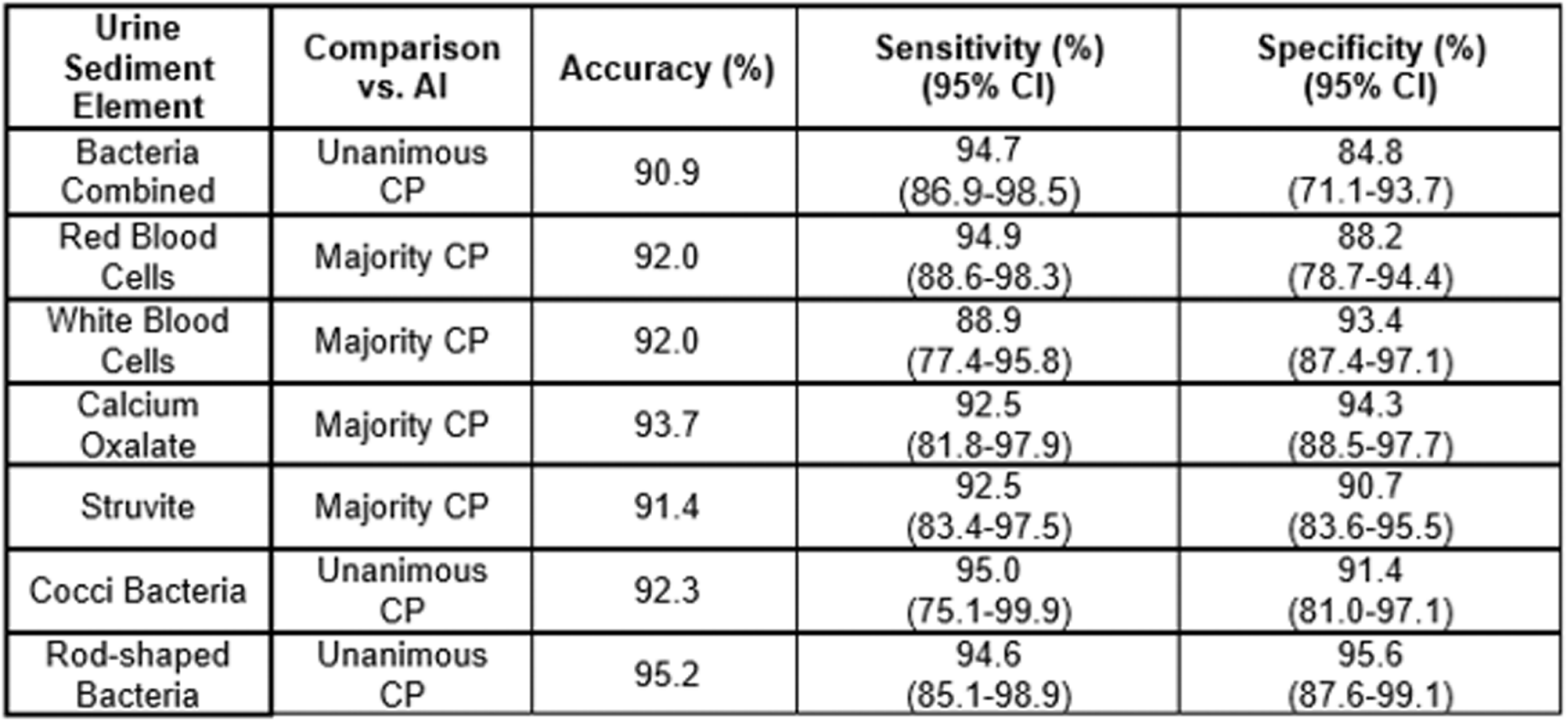
**Table 1. Accuracy, sensitivity, and specificity of version 2.0 of the Vetscan Imagyst AI Urine Sediment (AI) compared to clinical pathologists (CP) results**. Person‐Clopper

**Figure:**
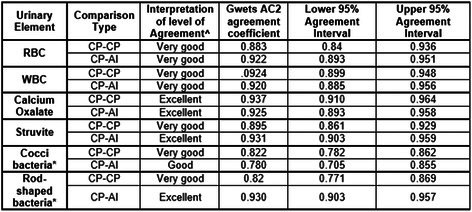
Table 2. Semi‐quantitative agreement between consensus clinical pathologists (CP) results* and version 2.0 of the Vetscan Imagyst AI Urine Sediment AI. CP‐CP = CP to CP comparison; CP‐AI = CP to AI comparison; *unanimous CP results were used for bacteria Landis JR, Koch GG. The measurement of observer agreement for categorical data. *Biometrics*. 1977;22:159–174. https://doi.org/10.2307/2529310

## 271 ABSTRACT NU24: Deep Learning Algorithm for Evaluation of Less Common Crystals and Spermatozoa in Canine/Feline Urine Sediment

### 271.1 **Mary Lewis** ^1^, MS, DVM, DACVP (Clinical); Anonda Haskin^2^; Dan Lin^3^; Eric Morissette^2^; Kristin Owens^2^; Cory Penn^2^

#### 271.1.1 ^1^Veterinary Clinical Pathologist, Imagyst Development, Medical Affairs, Zoetis; ^2^Zoetis; ^3^Analitix Giant Clinical Research Co. Ltd

**Background:** Urine sediment exam is vital to a complete urinalysis. A deep learning artificial intelligence (AI) tool for identification of less common crystals and spermatozoa at the point of care is currently lacking.

**Hypothesis/Objectives:** Vetscan Imagyst® AI Urine Sediment v2.0 will accurately identify cystine, bilirubin, and ammonium biurate crystals, and spermatozoa in agreement with digital review by ACVP‐boarded clinical pathologists (ACVP‐CPs).

**Animals:** Urine samples included those from client‐owned dogs and cats undergoing urinalysis for any reason submitted to Zoetis Reference Laboratories. A total of 30 samples (canine, 77%; feline, 23%) were evaluated.

**Methods:** Ten repeats of these 30 samples were prepped by the Vetscan Imagyst® preparation method, scanned, and converted to WSIs for review by three, blinded ACVP‐CPs and Vetscan Imagyst® AI Urine Sediment v2.0 algorithm for a total of 319 scans. Some samples contained more than one element of interest and only 9 repeats were possible for one sample. Algorithm performance and agreement was calculated as compared to ACVP‐CP results recorded as an average of 10, 40X fields.

**Results:** Accuracy, sensitivity, and specificity for urine sediment object classes ranged from 86–99%, 76–100%, and 81–99%, respectively (Table 1). Agreement between Vetscan Imagyst® AI Urine Sediment v2.0 and ACVP‐CPs was good to excellent (Table 2).

**Conclusions and Clinical Importance:** Vetscan Imagyst® AI Urine Sediment v2.0 was comparable to ACVP‐CPs in the identification and semi‐quantitative agreement of less common crystals and spermatozoa in urine sediment.

**Figure:**
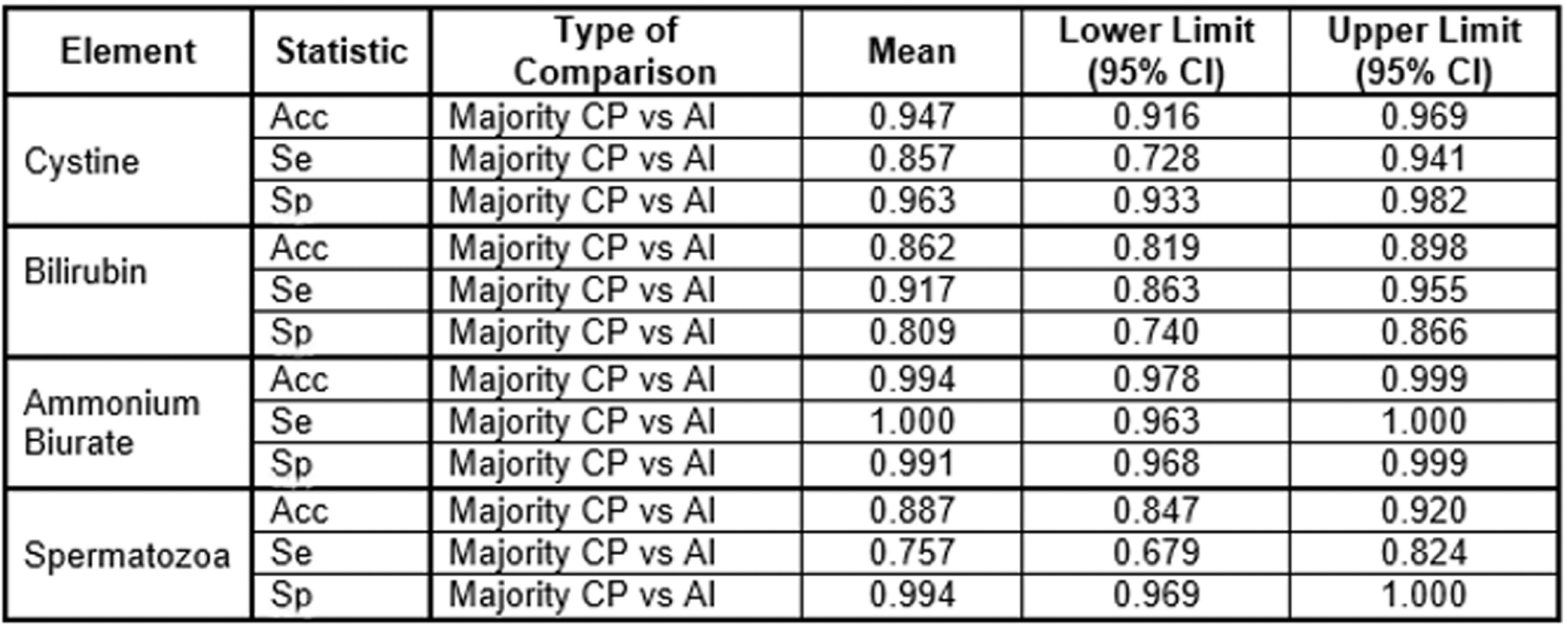
**Table 1. Accuracy, sensitivity, and specificity of Vetscan Imagyst AI Urine Sediment Version 2.0 compared to majority CP results**. Acc = accuracy; Se = sensitivity; Sp = specificity; CI = confidence interval. Pearson‐Clopper

**Figure:**
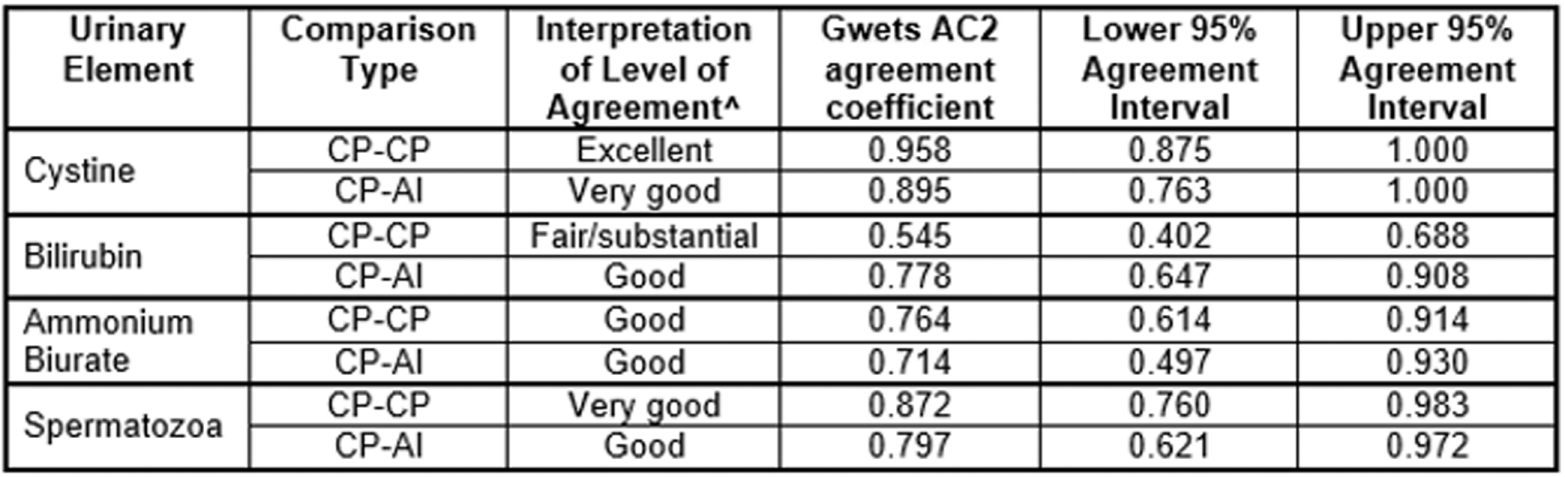
**Table 2. Semi‐quantitative agreement between consensus clinical pathologists (CP) results and version 2.0 of the Vetscan Imagyst AI Urine Sediment (AI)**. CP‐CP = CP to CP comparison; CP‐AI = CP to AI comparison. Landis JR, Kock GG. The measurement of observer agreement for categorical data. *Biometrics*. 1977;22:159–174. https://doi.org/10.2307/2529310

## 272 ABSTRACT NU25: Modulation of Fecal Microbiota in Dogs With Chronic Kidney Disease Supplemented With Probiotics

### 272.1 **Ignacio Lopez** ^1^, DVM, PhD; Antonio Camargo^2^, PhD; Carmen Pineda^3^, DVM, PhD; Carolina Arenas^4^, DVM, PhD, DECVIM; Escolastico Aguilera‐Tejero^5^, DVM, PhD

#### 272.1.1 ^1^Professor, University of Cordoba; ^2^Department of Internal Medicine, Lipids and Atherosclerosis Unit, Reina Sofia University Hospital; ^3^Animal Medicine and Surgery, University of Cordoba; ^4^Internal Medicine, Hospital Veterinario AniCura Valencia Sur; ^5^Animal Medicine and Surgery, University of Cordoba

**Background:** Probiotic supplementation has been suggested as an adjuvant therapy in patients with chronic kidney disease (CKD).

**Hypothesis/Objectives:** The aim of this study was to investigate the effects of a commercial probiotic formulation for companion animals on fecal microbiota in dogs with CKD.

**Animals:** Sixteen dogs with CKD (IRIS stage II and III) were recruited to a double‐blind, placebo‐controlled, randomized study. Either placebo (*n* = 8) or probiotic (Fortiflora® Nestle Purina PetCare) (*n* = 8) were added to their renal diets for 60 days. Feces were collected at the beginning and the end of the study. In addition, ten clinically normal dogs fed a standard commercial diet donated feces that were used as a control.

**Methods:** Fresh stool samples were processed to determine the intestinal microbiota profile by sequencing the microbial rRNA on the Illumina MiSeq. Microbiome Analyst 2.0 was used to compare groups at baseline and to visualize the results, using a taxonomic heat tree and the Wilcox test as statistical analysis.

**Results:** Higher abundance of Lachnospiraceae family and *Blautia* bacterial species and *Ruminococcus gnavus* group and lower abundance of Gammaproteobacteria class, Burkholderiales order, Sutterellaceae family, and *Sutterella* bacterial species was observed in dogs with CKD when compared with healthy dogs (Figure 1). Probiotic treatment resulted in a significant reduction in the population of *Ruminococcus gnavus* group but did not influence the other bacterial taxa (Figure 2).

**Conclusions:** In conclusion, feeding the probiotic Fortiflora® to dogs with CKD resulted in attenuation of uremia‐induced dysregulation of the gut microbiota.

**Figure:**
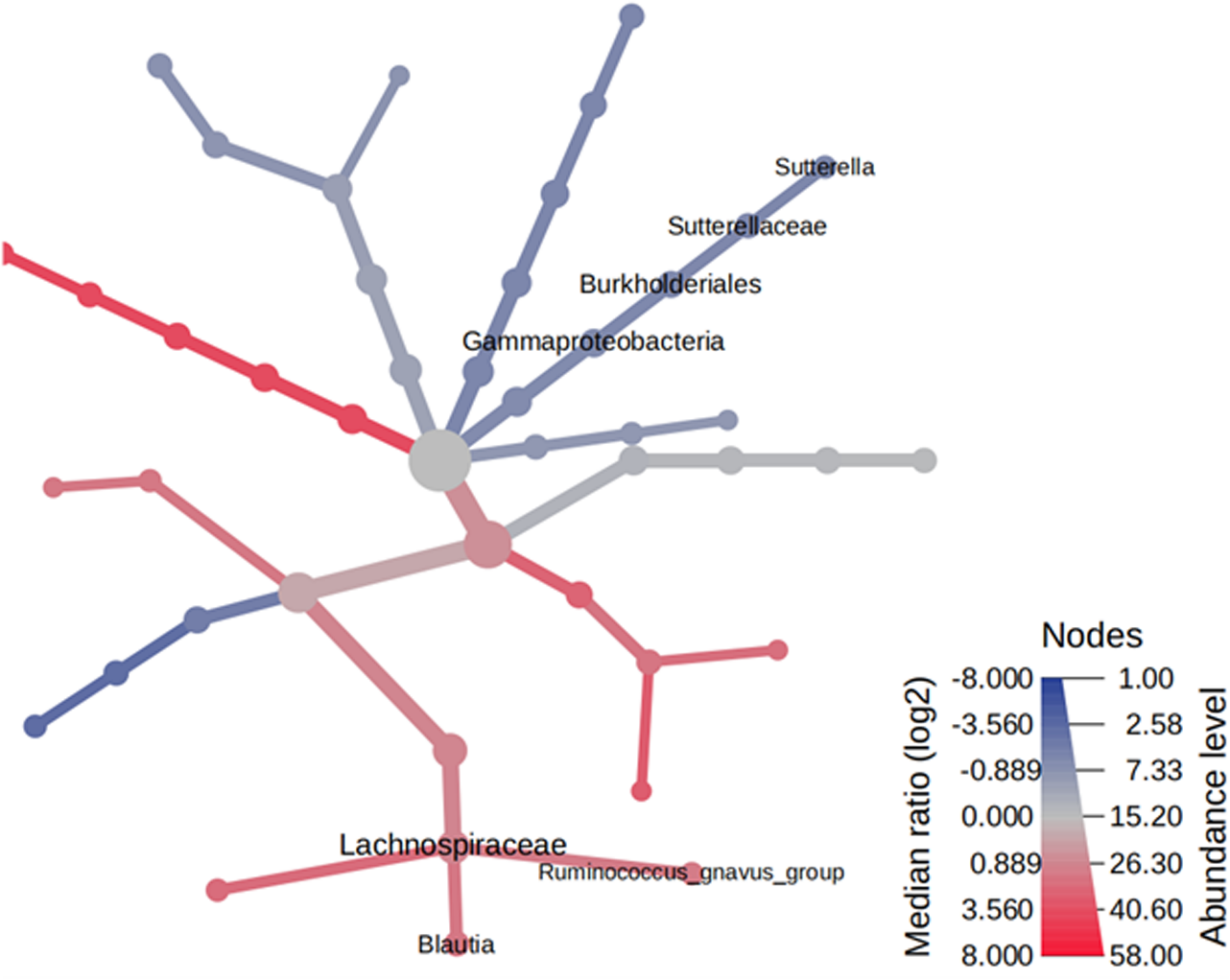
Figure 1. Taxonomic heat tree, to compare abundant taxa of the intestinal microbiota at baseline between dogs with chronic renal disease and a control healthy dogs group

**Figure:**
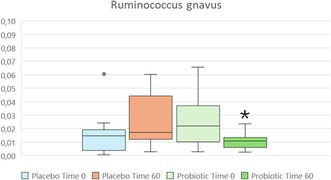
**Figure 2. Abundance changes (whiskers boxes) of *Ruminococcus gnavus* in the dogs of the study with CKD associated to the consumption of probiotics and placebo**. Dots represent disperses values. *p < 0.05 vs. probiotic time 0

## 273 ABSTRACT NU26: Sparse Sampling Pharmacokinetics of Pregabalin in Cats With Chronic Kidney Disease

### 273.1 **Jessica M. Quimby** ^1^, DVM, PhD, DACVIM (SAIM); Bianca Lourenco^2^; Meghan Lancaster^2^; Stacie Summers^3^; Lauren Trepanier^4^; Luke Wittenburg^5^; Chad Schmiedt^2^

#### 273.1.1 ^1^Professor, The Ohio State University; ^2^University of Georgia; ^3^Oregon State University; ^4^University of Wisconsin–Madison; ^5^University of California–Davis

**Background:** Pregabalin is utilized to help alleviate anxiety and fear associated with veterinary visits. Its published mean half‐life in healthy cats is 14.7 h. Appropriate dosing in cats with chronic kidney disease (CKD) is unknown, as pregabalin is cleared by renal excretion.

**Objective:** Assess the half‐life of two formulations of pregabalin in cats with surgically induced CKD using a sparse sampling approach.

**Animals:** 10 purpose‐bred cats in IRIS CKD stage 2 (*n* = 9) or 3 (*n* = 1).

**Methods:** In two sequential studies three months apart, two formulations of pregabalin were assessed: compounded capsules and oral solution (Bonqat®, 50 mg/mL). Both formulations were administered orally once at an intended dose of 2.5 mg/kg, and two blood samples per cat were obtained at differing time points post administration (3, 12, 16, 20, 24, 28, 36, and 48 h). Degree of sedation was scored. Serum pregabalin concentrations were measured via HPLC‐MS‐MS. Data were plotted on semi‐log graphs for visual inspection and analyzed using a non‐compartmental, sparse sampling method with uniform weighting. Elimination rate and half‐life predictions were made using at least 4 time points in the terminal phase.

**Results:** Administration of the oral solution was subjectively more difficult than that of capsules, and visual inspection of serum concentration‐time graphs showed more variability in this formulation. The predicted terminal half‐lives of the compounded and Bonqat® formulations of pregabalin were 24.8 and 26.3 h, respectively. No cat experienced excessive sedation.

**Conclusions:** The clearance of pregabalin is delayed in cats with CKD.

## 274 ABSTRACT NU27: Acid‐Base and Electrolyte Disorders in Dogs With Leptospirosis: Preliminary Results

### 274.1 **Danielle Santos** ^1^; Ammanuel Ouba^2^; Matheus Leite^1^; Victória Gomes^1^; Bruna Coelho^1^; Silvia Lucas^1^; Bruno Miotto^3^; Marcos Heinemann^4^; Fernanda Chacar^1^

#### 274.1.1 ^1^Clínica Médica (VCM), Faculdade de Medicina Veterinária e Zootecnia, Universidade de São Paulo; ^2^Mestrando, Clínica Médica (VCM), Faculdade de Medicina Veterinária e Zootecnia, Universidade de São Paulo; ^3^Programa de Pós‐Graduação em Saúde Única, Universidade Santo Amaro; ^4^Medicina Veterinária Preventiva e Saúde Animal, Faculdade de Medicina Veterinária e Zootecnia, Universidade de São Paulo

**Background:** Acid‐base and electrolyte disorders may occur in dogs with acute kidney injury due to leptospirosis.

**Objective:** To evaluate the occurrence of acid‐base and electrolyte disorders in dogs with leptospirosis.

**Animals:** Data from 18 dogs with leptospirosis treated at the Veterinary Teaching Hospital of the University of São Paulo.

**Methods:** Venous blood gas analysis was used to evaluate the acid‐base status. Serum concentrations of total calcium (Ca), total magnesium (Mg), and phosphorus (P) were also assessed. Data are presented as mean ±SD (min–max).

**Results:** Serum levels of Ca, Mg, and P were 8.98±1.49 mg/dL (6.69–10.73), 2.9±0.93 mg/dL (2.22–4.2), and 21.25±4.67 mg/dL (3.0–24.05), respectively. The occurrence of hypocalcemia and hyperphosphatemia was found in 50% of the cases; 33.3% of dogs had hypermagnesemia. Acid‐base disturbances were observed in 33.3% of dogs, with 50% presenting metabolic acidosis and 50% mixed disturbances. The mortality rate was higher than 80% in dogs with acid‐base disturbances.

**Conclusions:** The assessment of acid‐base status and electrolyte profile should be recommended in dogs with leptospirosis.

## 275 ABSTRACT NU28: Electrophoretic Urine Protein Banding Patterns in Healthy Cats and Cats With Kidney Disease

### 275.1 **Kate Sarkan** ^1^, DVM; Catherine Yeoman^3^; Jessica Quimby^2^, DVM, PhD, DACVIM; Katelyn Brusach^2^; Sarah Shropshire^4^, DVM, PhD, DACVIM; Jessica Hokamp^3^

#### 275.1.1 ^1^Combined Veterinary Pathology Resident/PhD Student, Department of Veterinary Clinical Sciences, The Ohio State University Veterinary Medical Center; ^2^Veterinary Clinical Sciences, The Ohio State University Veterinary Medical Center; ^3^Veterinary Pathobiology, Texas A&M University College of Veterinary Medicine; ^4^Clinical Sciences, Colorado State University College of Veterinary Medicine & Biomedical Sciences

**Background:** Determining the cause of proteinuric renal disease in cats presents a diagnostic challenge due to the necessity of renal biopsy for definitive diagnosis. Use of urine electrophoretic protein banding patterns to determine origin and cause of renal proteinuria is underexplored in cats.

**Objective:** Determine the expected urine protein banding patterns of clinically healthy cats and compare with cats with biopsy‐confirmed tubulointerstitial (TI) and glomerular diseases.

**Animals:** Banked urine supernatant from 15 clinically healthy cats and 29 cats with biopsy‐confirmed kidney disease: 10 stable chronic kidney disease (CKD) with TI damage; 12 immune complex‐mediated glomerulonephritis (ICGN); 7 non‐ICGN glomerular disease (amyloidosis, podocytopathy, focal segmental glomerulosclerosis).

**Methods:** Urine proteins were separated with sodium dodecyl sulfate polyacrylamide gel electrophoresis (SDS‐PAGE). Low (LMW, <36.5 kDa), intermediate (IMW, 36.5–66.3 kDa), high (HMW, 66.3–200 kDa), and very high (VHMW, > 200 kDa) molecular weight bands were enumerated.

**Results:** Healthy cat urine contained protein bands consistent with albumin, cauxin, and uromodulin. LMW bands were significantly greater in all diseases than in healthy cats and greater in ICGN and non‐ICGN glomerular diseases than CKD. IMW, HMW, and VHMW bands were significantly increased in ICGN and non‐ICGN cats compared to healthy and CKD cats. ICGN cats had the highest median number of VHMW bands; ≥ 3 VHMW bands was 83% sensitive and 91% specific for ICGN. (Table 1)

**Conclusions:** Urine SDS‐PAGE differentiates cats with glomerular disease from healthy cats and cats with TI CKD and has modest to high sensitivity and specificity for identifying ICGN cats.

**Figure:**
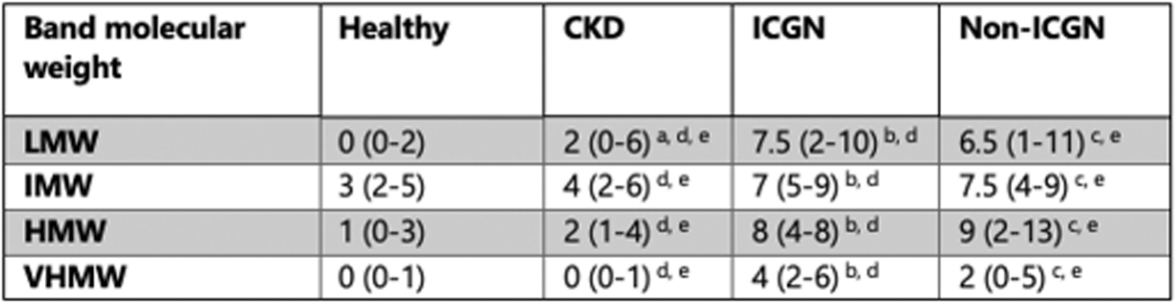
**Table 1. Median number and range of low (LMW), intermediate (IMW), high (HMW), and very high (VHMW) molecular weight protein bands on gel electrophoresis for healthy, CKD, ICGN, and non‐ICGN cats**. Statistical significance (*p* < 0.05). a. Healthy‐CKD. b. Healthy‐ICGN. c. Healthy‐non‐ICGN. d. CKD‐ICGN. e. CKD‐non‐ICGN. f. ICGN‐non‐ICGN

## 276 ABSTRACT O01: Minimally Invasive Sampling for Transcriptomic Profiling of the Immune Tumor Microenvironment in Canine B‐Cell Lymphoma

### 276.1 **Dillon Didehvar** ^1^; Jennifer Lenz^2^; Brandon Peng^2^; Lang Jiang^2^; Audrey Ghanian^2^; Matthew Atherton^2^

#### 276.1.1 ^1^Resident in Medical Oncology, PennVet, University of Pennsylvania, Philadelphia, PA, USA; ^2^PennVet, University of Pennsylvania, Philadelphia, PA, USA

**Background:** Despite initial favorable responses to cytotoxic chemotherapy, most canine patients with B‐cell lymphoma relapse within a year. However, a subset experience prolonged remission.

**Objectives:** We aimed to discover putative transcriptional and circulating biomarkers associated with remission duration.

**Animals:** Eighteen client‐owned canine patients with a confirmed diagnosis of naïve, intermediate‐to‐large, B‐cell lymphoma undergoing CHOP chemotherapy (vincristine, cyclophosphamide, doxorubicin, prednisone).

**Methods:** Patients were prospectively enrolled. Plasma, whole blood, and RNA from nodal aspirates were acquired prior to CHOP. Immune transcriptional profiling was undertaken utilizing the NanoString nCounter Canine IO panel, and data were analyzed on the ROSALIND platform. Serologic profiling was undertaken using ELISA and Luminex assays. Remission duration and outcome were recorded, establishing a minimally invasive biobank with linked hematologic, cytologic, immunophenotypic, and clinical metadata.

**Results:** Median time to progression (TTP) was 262.5 days for all patients, with 5 patients retaining remission over 2 years. Immune transcriptional profiling identified 47 differentially expressed genes (*p* < 0.05) between the five patients with significantly prolonged remission compared to the five patients with the shortest remission durations (log‐rank *p* = 0.0017). T‐cell transcripts and angiogenic markers were enriched in dogs with prolonged and short remissions respectively. Increased circulating VEGFA, IL‐6, and neutrophil: lymphocyte were documented in dogs with short remissions.

**Conclusions:** Our data revealed early evidence supporting favorable associations of T‐cell signatures with longer remissions and pro‐angiogenic activity associated with shorter remissions following CHOP for canine B‐cell lymphoma.

## 277 ABSTRACT O02: Optimization of Low‐Volume Blood Biopsy in Feline Cancer

### 277.1 **Katilee Jacobs** ^1^; Kate Megquier^2^, DVM; Tanya Karagiannis^3,4^, PhD; Carrie Wood^5^, DVM, DACVIM (Oncology); Heather Gardner^5^, DVM, PhD, DACVIM (Oncology)

#### 277.1.1 ^1^Veterinary Medical Oncology Resident, Cummings School of Veterinary Medicine, Tufts University, North Grafton, MA, USA; ^2^Broad Institute of MIT and Harvard, Cambridge, MA, USA; ^3^Institute for Clinical Research and Health Policy Studies, Boston, MA, USA; ^4^Department of Medicine, Tufts Medical Center, Tufts University, North Grafton, MA, USA; ^5^Foster Hospital for Small Animals, Cummings School of Veterinary Medicine, Tufts University, North Grafton, MA, USA

**Background:** The detection of cell‐free DNA (cfDNA) from plasma (e.g., liquid biopsy) is an emerging technique with many potential applications to facilitate cancer diagnosis and disease monitoring. Liquid biopsy approaches have been studied in canine cancer, but it is unknown if similar approaches can be used to facilitate staging and diagnosis of feline cancers.

**Hypothesis/Objectives:** It was hypothesized that liquid biopsy could be used to differentiate plasma samples originating from cats with and without cancer.

**Animals:** 19 client owned cats with oral squamous cell carcinoma (*n* = 5), gastrointestinal lymphoma (*n* = 9), or cats with no clinical evidence of cancer (*n* = 5).

**Methods:** Blood samples (<5 ml) were collected from cats into an EDTA or Streck cell‐free collection tube. Isolated cfDNA underwent ultra‐low‐pass whole genome sequencing with the resultant fastq files aligned with the feline genome (felis_catus_9.0). Copy number alterations and tumor fraction was determined using ichorCNA.

**Results:** cfDNA yield is variable, but not statistically different across diagnoses (*p* > 0.5). Somatic copy number changes were detected in samples taken from cats with known cancer, but not in the non‐cancer group.

**Conclusions and Clinical Importance:** cfDNA can reliably be isolated from feline plasma, and exhibits biologic variability consistent with other species. Tumor fraction was higher in cats with known cancer, supporting further optimization of liquid biopsy approaches in cats with cancer.

## 278 ABSTRACT O03: Comparison of Pooled vs. Single Samples Measuring Urine Protein: Creatinine Ratio in Dogs Receiving Toceranib Phosphate

### 278.1 **Kayla Neiderfer** ^1^; Kaitlin Curran^2^, DVM, MS, DACVIM (Oncology); Haley Leeper^3^, DVM, DACVIM (Oncology)

#### 278.1.1 ^1^Medical Oncology Resident, Oregon State University, Corvallis, OR, USA; ^2^Assistant Professor, Oncology, Clinical Sciences, Oregon State University, Corvallis, OR, USA; ^3^Clinical Associate Professor, Oncology, Clinical Sciences, Oregon State University, Corvallis, OR, USA

**Background:** It has been previously established that the measurement of urine protein: creatinine ratios (UPC) in pooled samples is superior in assessing proteinuria as compared to individual samples. However, the mainstay of monitoring proteinuria in patients receiving toceranib currently relies on analysis of a single urine sample. This practice lends to the potential for underestimating proteinuria.

**Hypothesis/Objectives:** The aim of this study is to evaluate the agreement between pooled and single sample UPC in dogs receiving toceranib.

**Animals:** As of abstract submission, 23 “events” are evaluable. Each visit for client‐owned dogs receiving toceranib was considered one “event.”

**Methods:** This is a prospective observational study of dogs receiving toceranib for the treatment of neoplasia. Urine samples were collected for three days prior to scheduled visits. An individual UPC measurement was obtained from the sample taken the morning of the visit, as well as from the pooling of all three samples. Additional diagnostics include a complete blood count, serum biochemistry, urinalysis, and blood pressure measurement.

**Results:** The Bland‐Altman plot was used to describe the agreement between pooled and single UPC measurements. Using this method, the average difference was ‐0.02 with 95% confidence interval of ‐0.316–0.273.

**Conclusions and Clinical Importance:** There is adequate agreement in measurements of UPC for single versus pooled samples for dogs receiving toceranib. Thus, collection of a single urine sample is appropriate for monitoring of non‐proteinuric dogs. A prospective study evaluating agreement in a population of proteinuric dogs would be needed to further validate these findings.

## 279 ABSTRACT O04: MicroRNAs as Prognostic Markers of Canine Splenic Hemangiosarcoma

### 279.1 **Laura Machado Ribas** ^1^; Mary Nowak^2^, DVM; Kerstin Muner^3^, DVM, MS; Elizabeth Rozanski^4^, DVM, DACVIM, DACVECC; Francisco Conrado^5^, DVM, MS, DACVP; Luis Felipe Dos Santos^6^, DVM, PhD, DACVIM (Cardiology); Andrea Pires dos Santos^7^, DVM, MS, PhD, DACVP

#### 279.1.1 ^1^Graduate Student, Purdue University, West Lafayette, IN, USA; ^2^Purdue University, West Lafayette, IN, USA; ^3^PhD Student, Comparative Pathobiology, Purdue University, West Lafayette, IN, USA; ^4^Associate Professor, Clinical Sciences, Tufts University, West Lafayette, IN, USA; ^5^Associate Professor, Comparative Pathobiology, Tufts University, West Lafayette, IN, USA; ^6^Assistant Professor, Department of Veterinary Clinical Sciences, Purdue University, West Lafayette, IN, USA; ^7^Associate Professor, Comparative Pathobiology, Purdue University, West Lafayette, IN, USA

**Background:** Splenic hemangiosarcoma (sHSA) is a highly aggressive cancer of endothelial cells. Due to its invasive nature, metastatic potential, and advanced stage at diagnosis, prognosis for affected dogs is poor, despite surgical resection and chemotherapy. Although histologic evaluation aids in diagnosis, grading systems often do not reflect tumor behavior, highlighting the urgency for accurate prognostic markers for sHSA.

**Hypothesis/Objectives:** We hypothesize that microRNA‐based biomarkers can assist in the prognostication of sHSA in dogs.

**Animals:** Archived tissue samples from 17 dogs diagnosed with sHSA were grouped based on overall survival (OS): group 1 (G1) <90 days (*n* = 6), group 2 (G2) 90 to 180 days (*n* = 6), and group 3 (G3) > 180 days (*n* = 5).

**Methods:** Total RNA was extracted from formalin‐fixed, paraffin‐embedded splenic samples and subjected to small RNA sequencing. Differentially expressed microRNAs identified in sequencing analysis were validated by qPCR.

**Results:** Sequencing revealed seven microRNAs differentially expressed across the groups: miR‐9–5p, miR‐155, miR‐184, miR‐335, miR‐483, miR‐542, and miR‐8865. MiR‐184 was excluded from the further analysis due to inconsistent amplification by qPCR. In addition, miR‐9‐5p, miR‐155, miR‐335, miR‐483, miR‐542, and miR‐8865 showed no significant differences (*p* > 0.05) by qPCR, while miR‐503 was downregulated in dogs with longer OS (*p* = 0.0147).

**Conclusions and Clinical Importance:** miRNomics suggests potential prognostic markers based on OS. The qPCR‐confirmed downregulation of miR‐503 was associated with longer survival times, indicating a potential role in sHSA progression. Ongoing studies aim to expand the sample size and explore these miRNAs in liquid biopsy samples to develop non‐invasive prognostic markers of sHSA.

## 280 ABSTRACT O05: Validating Antibodies for Cross‐Species Applications in Murine, Human, and Canine Osteosarcoma

### 280.1 **Kate Silver** ^1^; Jessica Beck^2^, DVM, PhD, DACVP; Donna Butcher^3^, BS; Elijah Edmondson^3^, DVM, PhD; Lauren McGee^2^, PhD; Christina Mazcko^2^, BS; Amy LeBlanc^2^, DVM

#### 280.1.1 ^1^Postbaccalaureate Fellow, Comparative Oncology Program, National Cancer Institute, National Institutes of Health, Bethesda, MD, USA; ^2^Comparative Oncology Program, National Institutes of Health, Bethesda, MD, USA; ^3^Molecular Histopathology Laboratory, Frederick National Laboratory for Cancer Research, Frederick, MD, USA

**Background:** Pet dogs develop osteosarcoma approximately 10 times as frequently as human patients, providing a crucial source of biospecimen and allowing researchers to fill critical knowledge gaps. However, comparative studies are constrained by the lack of appropriately validated antibodies across murine, canine, and human samples, which may limit the translational value of model species.

**Objective:** To curate a panel of antibodies that identify targets across murine, canine, and human tissues with a focus on the pulmonary metastatic osteosarcoma microenvironment.

**Animals:** Murine, canine, and human osteosarcoma and non‐tumor control tissues (lung, brain, liver, and spleen) were collected and processed using a standard operating procedure.

**Methods:** Immunohistochemical labeling was performed for each antibody in all three species using the same concentration and antigen retrieval conditions. Results were examined by a board‐certified pathologist to confirm appropriate immunolabeling. Antibodies were further validated using western blots.

**Results:** We identified a panel of antibodies that detect components of the microenvironment of metastatic osteosarcoma across murine, canine, and human lung tissues, including immune cells (CD3, CD20, CD204, FOXP3, IBA1), pneumocytes (TTF‐1), endothelium (CD31), lymphatics (PROX1), and osteosarcoma cells (ALPL, RUNX2, SAT2B).

**Conclusions and Clinical Importance:** Murine models and tumor bearing pet dogs are critical components of comparative oncology that aid in the advancement of human osteosarcoma therapies. This project identified a panel of antibodies that can be applied to metastatic osteosarcoma within murine, canine, and human lung. Validating cross‐species antibodies ensures that generated data achieves its full translational value while being efficient, cost effective, and precise.

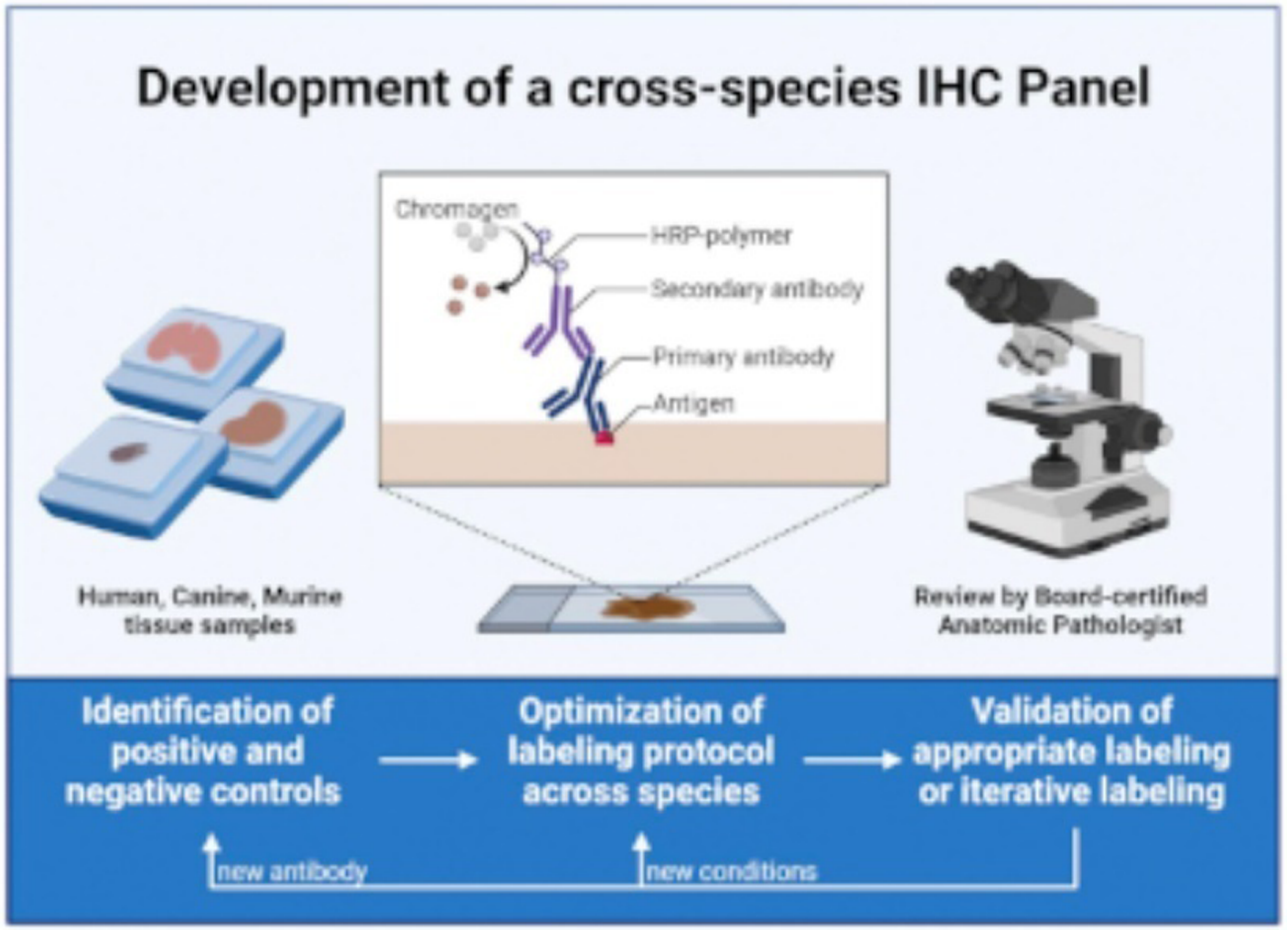

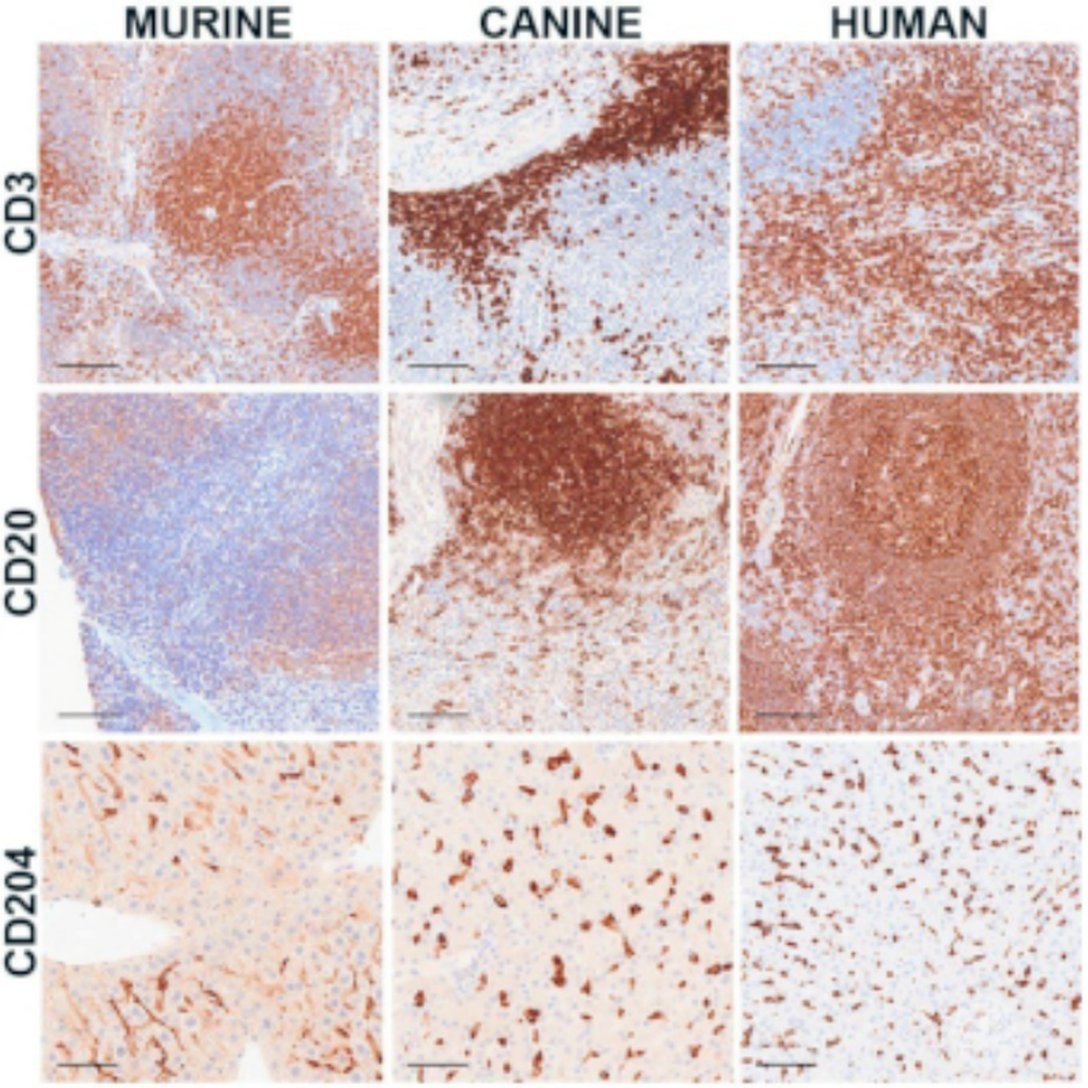

## 281 ABSTRACT O06: USA: Adoptive Cell Therapy with Chemotherapy Improves Canine Osteosarcoma Outcomes Compared to Standard of Care Chemotherapy

### 281.1 **Noe Reyes** ^1^; Tammie Wahaus^2^; Zachary Wright^3^, DVM, DACVIM (Oncology); Sharon Shor^4^, DVM, MS, DACVIM (Oncology); Amy Back^5^; Tariq Shah^6^, BSc; Gary Wood^7^, PhD; Wayne Carter^8^, DVM, PHD; Barry Skikne^9^, MD, FACP, FCP (SA); Jeffrey Bryan^10^, DVM, MS, PHD, DACVIM (Oncology)

#### 281.1.1 ^1^Chief Medical Officer, ELIAS Animal Health; ^2^CEO, ELIAS Animal Health; ^3^VCA Animal Diagnostic Clinic; ^4^BluePearl Pet Hospital Tacoma, Lakewood, WA, USA; ^5^MedVet Dayton, Moraine, OH, USA; ^6^Oncologize, Columbia, MO, USA; ^7^CSO, TVAX Biomedical, Lenexa, KS, USA; ^8^CEO, TVAX Biomedical, Lenexa, KS, USA; ^9^Kansas University Medical Center, Kansas City, KS, USA; ^10^Professor, College of Veterinary Medicine, University of Missouri, Columbia, MO, USA

**Background:** There is growing evidence that combined chemo‐immunotherapy improves outcomes for cancer patients. Combining the initial cytotoxic effect of chemotherapy with the immunological effects of adoptive cell therapy (ACT) aims to increase overall response and survival rates.

**Methods:** This prospective clinical trial evaluated overall survival times of companion dogs (*n* = 17) newly diagnosed with osteosarcoma (OSA) receiving 1–4 doses of carboplatin followed by ACT initiated 21 days following the final chemotherapy dose. After amputation, autologous cancer cell vaccinations were administered to generate neoantigen‐specific T cells which were later harvested, *ex vivo* expanded/activated, and intravenously administered. Survival time, tumor location, and other demographic data were collected. A set of matching control dogs (*n* = 17) were selected from a published dataset in the National Cancer Institute's Canine Data Commons. Kaplan‐Meier analysis was conducted with survival data compared to historical published results of dogs receiving chemotherapy only as therapy.

**Results:** Dogs in ACT group had a median survival time (MST) of 486 days compared to 208 days for matched controls. One‐year survival rates were 53% and 18%, respectively. No statistical difference was observed between groups (*p* > 0.2).

**Conclusion:** The initial analysis of this study, dogs with OSA treated with ACT and chemotherapy showed improved outcomes compared to carboplatin alone and this therapeutic approach was well‐tolerated. Clinical relevance was demonstrated through improved MST and 1‐year survival rates. Results suggest chemo‐immunotherapy may improve outcomes in canines with OSA. The translational impact of this research may have important implications for treatment of OSA in human medicine.

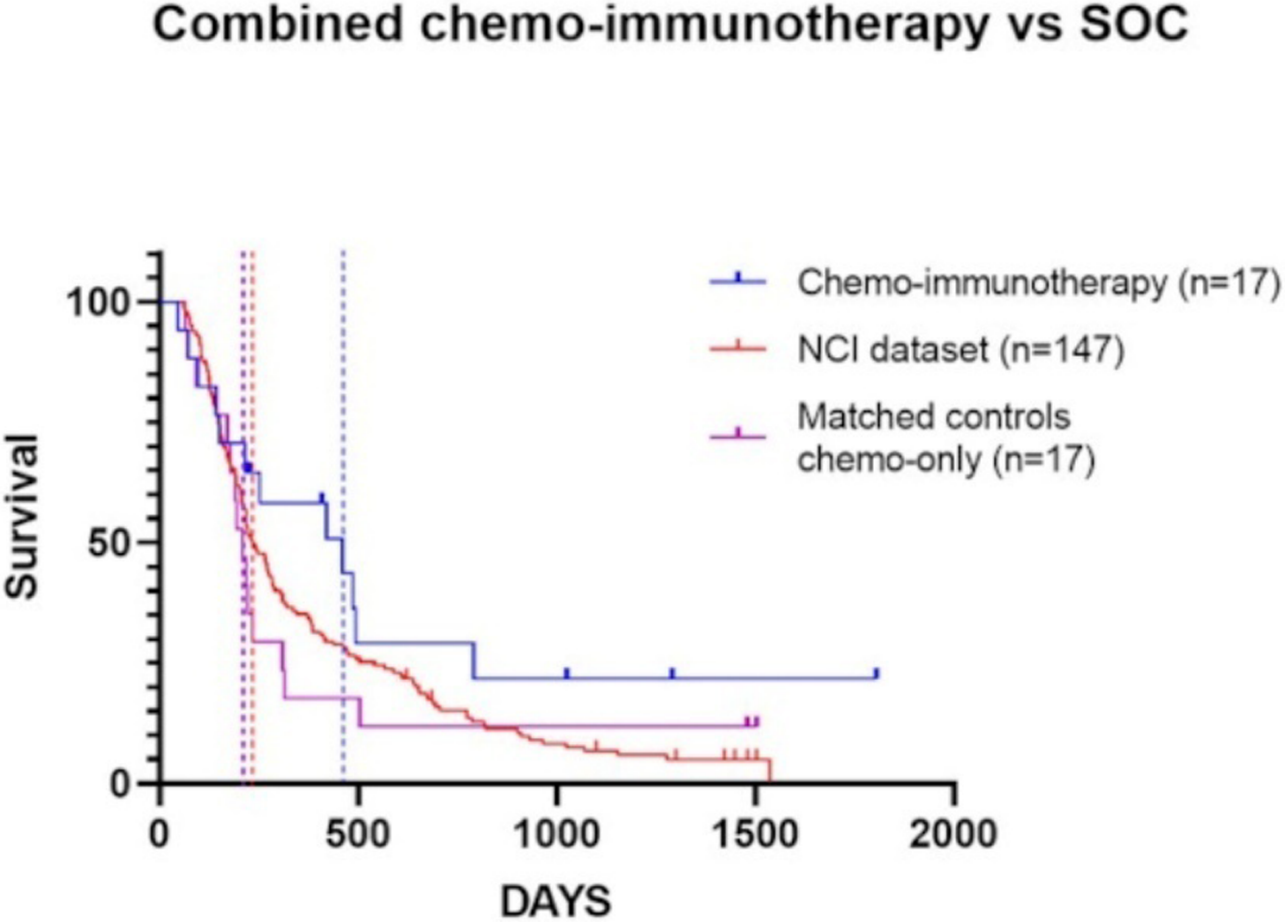

## 282 ABSTRACT O07: Plasma Extracellular Vesicles in Non‐Cancerous Bone Disease: Implications for Osteosarcoma Liquid Biopsy

### 282.1 **Meagan Wojtysiak** ^1^; Jaron Magstadt^2^; Courtney Labé^2^; Rachel Bolle^2^; Andrea Chehadeh^2^; Sara Pracht^2^; Amber Winter^2^; Kelly Reid^2^; Michelle Buettner^2^; Caitlin Feiock^2^; Mitzi Lewellen^2^; Adam Cook^2^; Kyle Duval^2^; Julia Miller^2^; Ali Khammanivong^2^; Aaron Rendahl^2^; Brenda Weigel^2^; Logan Spector^2^; Jaime Modiano^2^; Wanda Gordon‐Evans^2^; Kelly Makielski^2^

#### 282.1.1 ^1^DVM Candidate, University of Minnesota, Minneapolis, MN, USA; ^2^University of Minnesota, Minneapolis, MN, USA

**Background:** Many cancers are diagnosed when advanced, highlighting the need for early detection. As part of developing a canine osteosarcoma liquid biopsy test, we examined physical parameters of extracellular vesicles (EVs) in canine cancers and benign musculoskeletal diseases (MD) important comparisons for osteosarcoma due to overlapping clinical signs.

**Hypothesis/Objectives:** We aimed to characterize plasma EV size and concentration from dogs with cancer, dogs with MD, and healthy dogs. We hypothesized that physical EV parameters would be distinct between groups.

**Animals:** Two sets of client‐owned dogs were studied. Set‐1: Healthy dogs (*n* = 26), dogs with cancer (*n* = 29; osteosarcoma *n* = 8). Set‐2: Healthy dogs (*n* = 10), dogs with MD (*n* = 50).

**Methods:** Plasma EVs were enriched by precipitation (Set‐1) or size exclusion chromatography (Set‐2), and characterized by nanoparticle tracking analysis. Medians/IQRs are reported and Wilcoxon tests compared characteristics between groups.

**Results:** Set‐1: osteosarcoma dogs had a smaller median EV size than healthy dogs (osteosarcoma median/IQR: 78.7/24.1 nm; healthy: 113.0/32.8 nm; *p* = 0.0065) and dogs with other cancers (median/IQR: 104.5/36.6 nm, *p* = 0.04). Set‐2: the median EV size in dogs with MD was smaller than healthy dogs (MD median/IQR: 79.9/12.0 nm; healthy: 96.4/18.6 nm; *p* = 0.00025). Median EV concentrations were higher in dogs with osteosarcoma and MD than in their respective healthy dog groups. Importantly, the size and concentration distributions overlapped between groups.

**Conclusions and Clinical Importance:** Physical parameters of canine plasma EVs alone cannot differentiate between benign and malignant conditions. Molecular analysis of EV contents will robustly improve differentiation, creating opportunities to develop clinically useful diagnostic tools.

## 283 ABSTRACT O08: Relationship Between Lactate Dehydrogenase and Substage in Dogs Diagnosed with Multicentric Large B‐Cell Lymphoma

### 283.1 **Mio Ayukai** ^1^; Darren Fry^2^

#### 283.1.1 ^1^Small Animal Medicine Registrar, Queensland Veterinary Specialists; Brisbane Veterinary Specialist Centre, Albany Creek, QLD, Australia

**Background:** Lactate dehydrogenase (LDH) is validated prognostic marker in non‐Hodgkin's lymphoma in humans and a component of the International Prognostic Index for diffuse large B‐cell lymphoma. The significance of serum LDH levels in dogs diagnosed with multicentric large B‐cell lymphoma is unclear.

**Objectives:** The purpose of this study was to determine whether there was a correlation between LDH and substage.

**Animals:** Twenty‐seven dogs diagnosed with multicentric B‐cell lymphoma from a veterinary hospital met inclusion criteria and were included in the study.

**TABLES AND FIGURES:**

**Methods:** In the prospective cohort study, LDH concentrations were measured using an IDEXX Catalyst ONE Veterinary Chemistry analyser prior to commencing CHOP chemotherapy. Group one included subjects with LDH within the reference range (40–400 U/L) and group two, included those above (> 400 U/L). Details of clinical remission, date of cytologically confirmed disease relapse and euthanasia were recorded. Disease free interval (DFI) and mean survival time was calculated.

**Results:** Statistical analysis showed no significant relationship between LDH levels and disease substage (*p* = 0.396). The data indicates that LDH is not a strong predictor of disease substage in the cohort. Median DFI was higher for subjects with LDH > 400 U/L compared to those that were below. However, log rank test revealed that there was no statistically significant difference between the two groups (*p* = 0.96).

**Conclusion:** This study suggests that there is no clear correlation between LDH concentrations and substage, therefore, other factors likely play a role in the biomarker metabolism. Limitations of the study included the small study population.

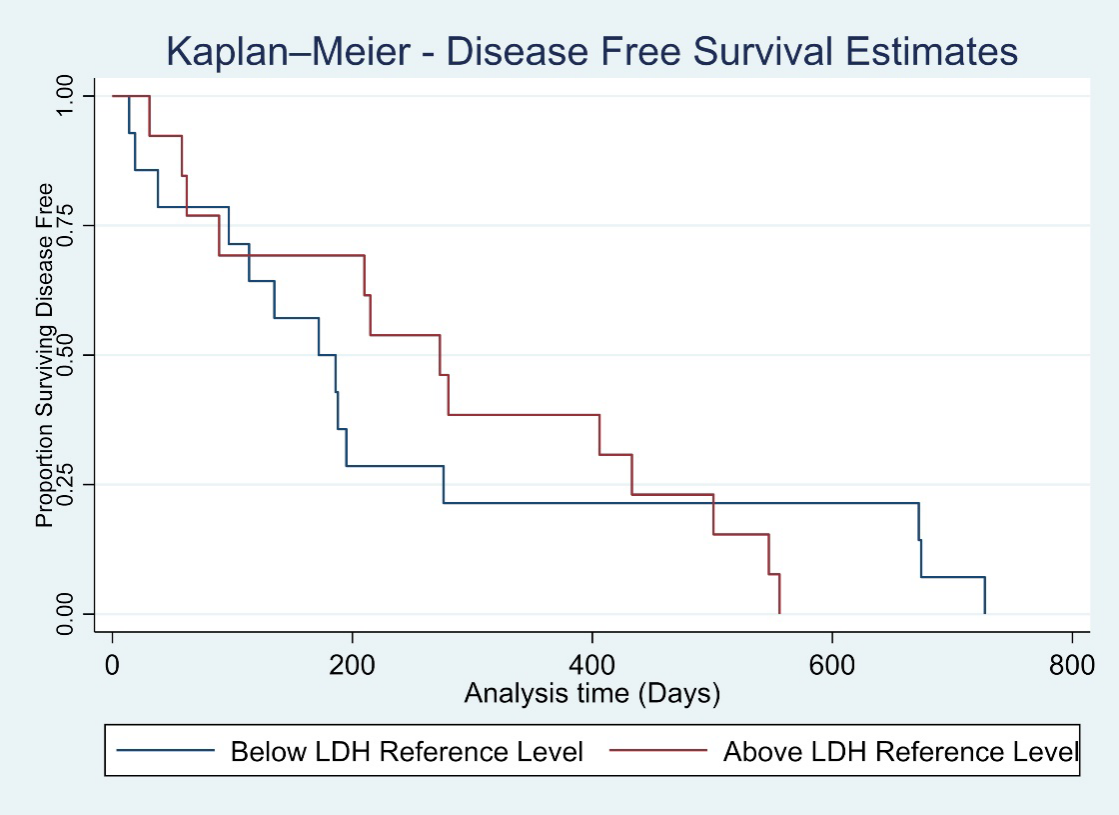

**Table:** 

LDH Ref Level	Time at risk	Incidence rate	Number of subjects	Disease free interval		
				25%	Median	75%
Below	3,507	0.003992	14	97	172	276
Above	3,661	0.0035509	13	89	273	433
Total	7,168	0.0037667	27	89	195	433

**Table:** 

	LDH Reference Range		
Substage	Below	Above	Total
Substage A	10	11	21
	47.62	52.38	100.00
	66.67	84.62	75.00
Substage B	5	2	7
	71.43	28.57	100.00
	33.33	15.38	25.00
Total	15	13	28
	53.57	46.43	100.00
	100.00	100.00	100.00

Fisher's exact Pr = 0.396

Key:

Row 1 = frequency

Row 2 = row percentage

Row 3 = column percentage

## 284 ABSTRACT O09: Organoid‐Based Platform for Predicting Anticancer Drug Susceptibility in Animals

### 284.1 **Jeongmin Lee** ^1^; Jung Eun Kim^2^, PhD; Jiah Yeom^2^, PhD; Hyun Jae Kim^3^, DVM

#### 284.1.1 ^1^Director, Korea Animala Medical Center, Cheongju‐si, Ch’ungch’ong‐bukto, Republic of Korea; ^2^Precision Medicine, PODO Therapeutics, Seongnam‐si, Gyeonggi‐do, Republic of Korea; ^3^Seoul Animal Medical Center, Sejong‐si Republic of Korea

**Background:** With the aging of animals, the incidence of cancer has been steadily increasing. Despite the diverse clinical characteristics observed in tumor patients, veterinary oncology often relies on standardized treatment protocols. However, this generalized approach frequently leads to side effects and low therapeutic efficacy.

**Objectives:** To establish a platform that cultures cancer organoids and performs drug sensitivity testing to propose “personalized” anticancer therapies.

**Animals and Methods:** Canine and feline organoids were generated from tumor tissues through surgical excision or fluid samples, such as urine. Tumor origin was confirmed via immunohistochemical staining. Organoids were treated with chemotherapeutic and targeted drugs for 5 days, and cell viability was assessed using microscopy and a luminol‐based assay.

**Results:** Tumor organoids were successfully established from tumor samples from canine and feline. IHC staining confirmed tumor biomarker expression in organoids. Cytotoxicity varied between ineffective and effective drugs within individual organoids upon treatment with chemotherapeutic and targeted anticancer agents. Effective drugs significantly reduced organoid size, number, and luminescence‐based cell viability compared to controls. Notably, drug effectiveness varied even among organoids from the same cancer type. Organoid‐guided therapy improved survival outcomes (*n* = 25, mean: 99.24±58.47 days, median: 78 days) compared to unguided therapy (*n* = 9, mean: 53.22±22.33 days, median: 51 days; *p* = 0.016).

**Conclusions and Clinical Importance:** We developed a novel platform that cultures 3D cancer organoids from canine and feline tumor samples to provide anticancer drug sensitivity results. This platform is expected to make a significant contribution to advancing personalized and precision medicine in veterinary oncology.

## 285 ABSTRACT O10: Assessing Individual Variation in Chemoradiation Responses in Canine Bladder Cancer Organoids

### 285.1 **Karin Allenspach** ^1^, DVM, DECVIM‐CA, PhD; Michael Catucci^2^, BS; Travis Laver^3^, VMD, PhD, ACVVIM; MacKenzie Long^2^, BS; Bryan Melvin^4^, BS; Jonathan Mochel^5^, DVM, PhD, DECVPT; Koichi Nagata^5^; Hannah Nicholson^2^, BS; Maria Orbay‐Cerrato^5^, DVM; Aleksandra Pawlak^6^, DVM, PhD; Jiayi Peng^2^, BS; Emily Rawlings^5^, DVM; Corey Saba^7^, DVM, DACVIM; Christopher Zdyrski^8^, PhD

#### 285.1.1 ^1^Professor of Comparative Medicine, University of Georgia, Athens, GA, USA; ^2^PhD Student Pathology, University of Georgia, Athens, GA, USA; ^3^Clinical Sciences, University of Georgia, Athens, GA, USA; ^4^DVM/PhD Student, University of Georgia, Athens, GA, USA; ^5^University of Georgia, Athens, GA, USA; ^6^Senior Research Scientist, Pathology, University of Georgia, Athens, GA, USA; ^7^Professor of Oncology, Clinical Sciences, University of Georgia, Athens, GA, USA; ^8^Senior Research Scientist, Pathology, University of Georgia, Athens, GA, USA

**Background:** Bladder cancer, particularly high‐grade urothelial carcinoma (UC), poses significant treatment challenges in humans and canines due to its high lethality. Canines are valuable models for studying human UC due to their similar histologic and molecular characteristics and biological behavior. Recent research aims to enhance treatment efficacy by combining radiation therapy with subsequent chemotherapy. With their complex cellular architecture, canine UC urine‐derived organoids are more accurate in predicting treatment responses than conventional 2D cell lines.

**Objective:** This study aimed to evaluate individual *in vitro* variation in cytotoxicity response to sequential radiation and chemotherapy compared to cisplatin monotherapy using urine‐derived canine UC organoid cell lines.

**Methods:** Canine UC organoid cell lines from two patients were isolated, expanded, plated (10,000 cells/well), and irradiated (5, 9, or 15 Gy) 24 h after seeding. Following 72 h of incubation, the organoids were treated with cisplatin (10, 25, 50, or 100 μM) with untreated cells as controls. Metabolic activity was measured after one week using Presto Blue. Histological analysis including immunohistochemistry to assess apoptosis (Caspase 3/7) and proliferation (Ki‐67) were performed.

**Results:** Using radiation with subsequent chemotherapy induced greater cytotoxicity than cisplatin monotherapy, and differential IC50s were observed between the two patients (IC50: ranging from 10–50 μM; 5–9 Gy).

**Conclusion:** Significant individual variation in cytotoxicity was observed in *in vitro* radiation/chemotherapy protocol responses. This highlights the possibility of interpatient variation in therapeutic response and the importance of developing personalized treatment strategies to optimize therapeutic outcomes.

## 286 ABSTRACT O11: Hypoxia Enhances Vasculogenic Mimicry Formation in Canine Mammary Tumor Cell Lines

### 286.1 **Yunho Jeong** ^1^; Jeong‐Ho Ha^2^; Jung Hoon Choi^3^; Jin‐Young Chung^2^; Jin‐Ok Ahn^2^

#### 286.1.1 ^1^DVM/PhD Student, Department of Veterinary Internal Medicine, College of Veterinary Medicine, Kangwon National University, Chuncheon, South Korea; ^2^Department of Veterinary Internal Medicine, College of Veterinary Medicine, Kangwon National University, Chuncheon, South Korea; ^3^Department of Anatomy, College of Veterinary Medicine, Kangwon National University, Chuncheon, South Korea

**Background:** Vasculogenic mimicry (VM) formation is closely associated with drug resistance, tumor invasion, and metastasis in human breast cancer. Hypoxia is recognized as a key driver of VM formation. Similarly, canine mammary gland tumors (CMGT), particularly aggressive phenotypes, exhibit VM characteristics.

**Objectives:** This study aimed to compare genes and proteins associated with VM formation between two CMGT cell lines (CF41.Mg and CMT‐U27) under hypoxic conditions induced by cobalt chloride (CoCl_2_).

**Methods:** Vasculogenic mimicry (VM) formation was assessed using a Matrigel‐based 3D‐culture system under normal and CoCl_2_‐induced hypoxic conditions. RNA sequencing was performed after tubule formation to identify genes associated with VM formation. Quantitative polymerase chain reaction (qPCR) and Western blot analyses were subsequently conducted to validate the findings.

**Results:** Only the CF41.Mg cell line formed tubular structures, with hypoxia significantly enhancing the number of tubules (*p* < 0.05). Gene enrichment analysis revealed differences in gene ontology between the two CMGT cell lines, particularly in pathways related to angiogenesis, cell migration, and extracellular matrix remodeling. Selected genes and associated proteins identified through RNA sequencing exhibited significant upregulation or downregulation following CoCl_2_ treatment (*p* < 0.05).

**Conclusions and Clinical Importance:** CF41.Mg and CMT‐U27 differed in their VM‐forming ability, with hypoxia enhancing VM only in the CF41.Mg cell line. These findings suggest a strong link between hypoxia and VM formation in aggressive CMGT, providing insights into tumor progression and potential targets for future research.

## 287 ABSTRACT O12: Circulating Progranulin: A Promising Biomarker for Diagnosis and Prognostic Prediction in Canine Tumors

### 287.1 **Keon Kim** ^1^, DVM; Chang‐Hyeon Choi^2^; Yeong‐Jun Kim^2^; Jong‐Hwan Park^2^; Yoon Jung Do^3^; Woong‐Bin Ro^2^; Chang‐Min Lee^2^

#### 287.1.1 ^1^PhD Candidate, Graduate Student, Chonnam National University, Gwangju, South Korea; ^2^Chonnam National University, Gwangju, South Korea; ^3^Rural Development Administration

**Background:** Progranulin (PRGN) is a pluripotent growth factor that has shown promise as diagnostic and prognostic biomarker for various neoplastic conditions in human medicine.

**Objective:** To evaluate the applicability of serum PRGN as a biomarker in veterinary oncology, providing novel insights into the clinical management of canine tumors.

**Animals:** The tumor group (*n* = 110) comprised dogs retrospectively selected where neoplastic diseases were the primary concern. For detailed analysis, the tumor group was categorized based on histopathological origin, malignancy, and metastatic status. The control group (*n* = 30) consisted of healthy dogs with no evidence of neoplastic diseases, confirmed through medical history, physical examination, blood tests, and imaging studies.

**Methods:** Circulating PRGN levels were quantified using enzyme‐linked immunosorbent assay (ELISA) methods.

**Results:** Dogs with tumors exhibited significantly elevated circulating PRGN levels compared to control dogs (*p* < 0.0001****), with a high sensitivity of 90.91%. Malignant and metastatic tumors demonstrated markedly higher PRGN levels relative to the control group (*p* = 0.0012** and *p* < 0.0001****, respectively). Additionally, serum PRGN was identified as a significant marker for differentiating metastatic tumors from non‐metastatic ones (*p* = 0.0264*).

**Conclusions and Clinical Importance:** This study underscores the potential of PRGN as a novel biomarker with considerable diagnostic and prognostic value in the evaluation of canine neoplastic diseases.

**Figure:**
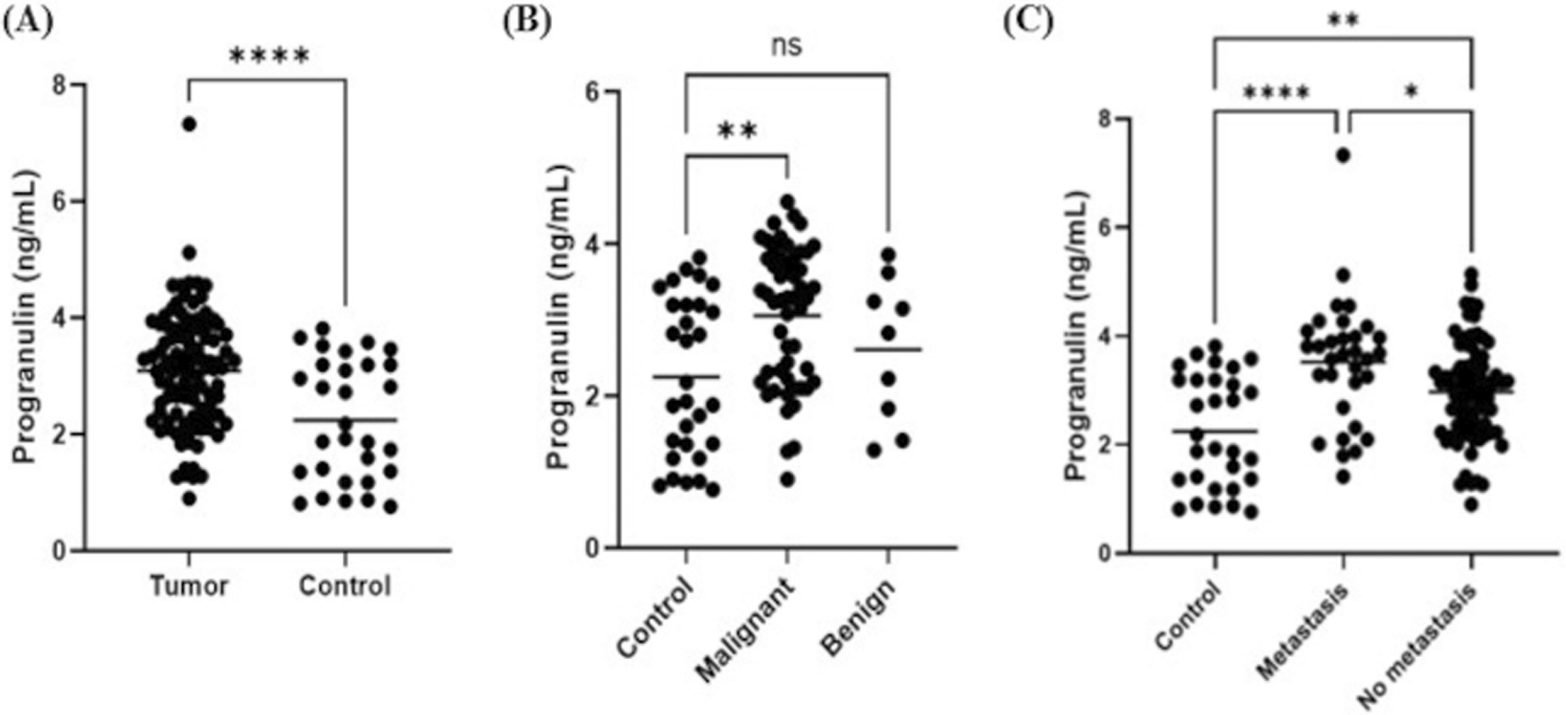
**Figure 1. Scatter dot plots of PRGN concentrations in dogs with tumors and control group**. (A) Dogs with tumors showed significantly higher PRGN concentrations than control group (*p* < 0.0001****). (B) Dogs with malignant tumors showed significantly higher PRGN concentrations than control dogs (*p* = 0.0012**). In contrast, dogs with benign tumors showed no significant difference of PRGN concentrations compared to control dogs. (C) Dogs with tumors showed significantly higher PRGN concentrations than control dogs regardless of the metastasis (*p* < 0.0001**** and *p* = 0.0024**, respectively). Furthermore, in dogs with metastatic tumors, PRGN levels were significantly higher compared to tumors without metastasis (*p* = 0.0264*). PRGN, progranulin.

**Figure:**
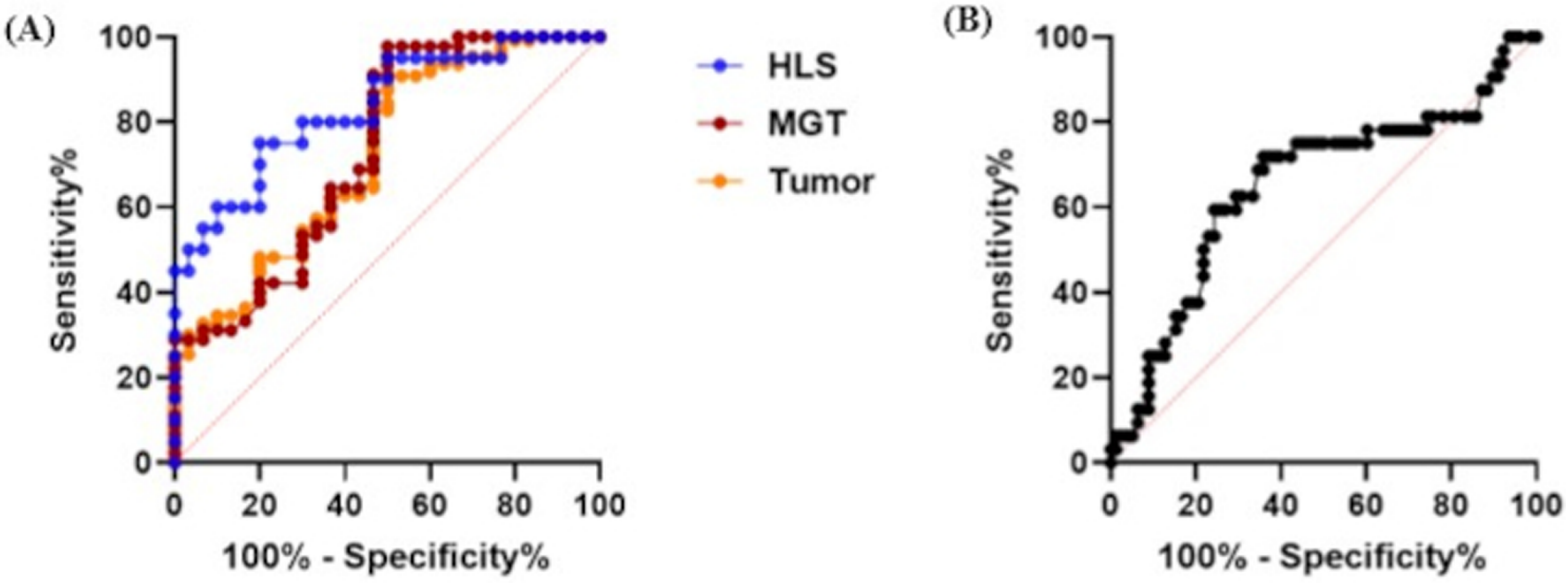
**Figure 2. ROC curve of PRGN concentrations to confirm diagnostic and prognostic accuracy of tumors**. (A) ROC curve for predicting the dogs with tumors (AUC=0.723; *p* = 0.0002***), MGTs (AUC=0.734; *p* = 0.0006***) and HLS tumors (AUC=0.835; *p* < 0.0001****) compared to healthy dogs. (B) ROC curve for predicting the metastasis of canine tumors (AUC=0.651; *p* = 0.0129*) compared to the canine tumors without metastasis. ROC, receiver operating characteristic; PRGN, progranulin; AUC, area under the curve; MGT, mammary gland tumor; HLS, hematopoietic, and lymphoreticular system.

## 288 ABSTRACT O13: 3D Organoid‐Based Drug Sensitivity Analysis for Personalized Chemotherapy in Canine Sarcoma

### 288.1 **Jeongmin Lee** ^1^, DVM, PhD; Woo‐Seok Yang^2^, DVM; Munbin Cho^2^, DVM; Ji‐Hyun Han^2^, DVM; Jung Eun Kim^3^, PhD; Jiah Yeom^3^, PhD

#### 288.1.1 ^1^Director, Korea Animal Medical Center, Seowon‐gu, Cheongju‐si, Republic of Korea; ^2^Korea Animal Medical Center, Seowon‐gu, Cheongju‐si, Republic of Korea; 3Precision Medicine, PODO Therapeutics, Eongnam‐si, Gyeonggi‐do, Republic of Korea

**Background:** In advanced, high‐grade, recurrent, or metastatic sarcomas, chemotherapy is used; however, its effectiveness remains limited. Recently, in human medicine, patient‐derived organoids have gained attention as a promising tool for predicting anticancer drug susceptibility, highlighting the potential of organoid‐based platforms in personalized cancer treatment.

**Hypothesis/Objectives:** This study aims to develop an organoid‐based platform to visually quantify patient‐derived organoid responses to chemotherapy, enabling personalized treatment strategies for canine sarcoma.

**Animals:** 10 dogs diagnosed with sarcoma at Korea Animal Medical Center between June 2024 and January 2025.

**Methods:** Canine organoids were generated from tumor tissues through surgical excision confirmed as sarcoma by histopathology. Organoids were treated with chemotherapeutic and targeted drugs for 5 days, and cell viability was assessed using microscopy and a luminol‐based assay for comparing drug sensitivities including doxorubicin, toceranib, cyclophosphamide, vincristine, epirubicin, and sorafenib.

**Results:** A total of ten cases were identified, including soft tissue sarcoma (*n* = 5), hemangiosarcoma (*n* = 3), osteosarcoma (*n* = 1), and stromal sarcoma (*n* = 1). Among the tested chemotherapeutic agents, doxorubicin (*n* = 6) exhibited the highest response rate, followed by toceranib (*n* = 3) and mitoxantrone (*n* = 1). The most effective drug varied among individual patients, regardless of tumor type, suggesting that treatment response may be highly patient‐specific.

**Conclusions and Clinical Importance:** The evaluation was conducted using a 3D organoid model in canine sarcoma patients. Based on the observed *ex vivo* responses, this approach could be considered as a tool for applying personalized chemotherapy *in vivo*. However, further research is needed to validate its clinical utility.

## 289 ABSTRACT O14: Retrospective Analysis of Canine Splenic Tumors: 54 Cases

### 289.1 **Yiyu Li** ^1^, VMM; Yujia Peng^2^; Gebin Li^3^

#### 289.1.1 ^1^Doctor, Veterinary Teaching Hospital of China Agricultural University, Hedong, Tianjin, China; ^2^Veterinary Teaching Hospital of China Agricultural University, Hedong, Tianjin, China; ^3^Gebin Li, College of Veterinary Medicine, China Agricultural University, Beijing, China

**Background:** Canine splenic tumors are frequently encountered in small animal clinical practice and certain portions of patients have short survival (<2 weeks).

**Hypothesis/Objectives:** The aim of this study was to investigate the clinical behavior of splenic tumors in dogs, and to explore the underlying causes causing short survival.

**Animals:** 54 eligible canine splenic tumor cases were collected from the Veterinary Teaching Hospital of China Agricultural University from 2020 to 2024.

**Methods:** The clinical records were retrospectively reviewed. Clinical presentation, clinicopathological abnormalities, treatment, and histopathology results were recorded.

**Results:** Hemangiosarcoma (72.2%), liposarcoma (7.4%), and lymphoma (5.6%) were the most common types of splenic neoplastic tumors. 41.2% of the neoplastic disease patients had metastasis disease at diagnosis, 15.7% had lung metastasis and 33.3% (17/51) had abdominal metastasis. 18.5% (10/54) of neoplastic splenic tumor patients survived less than 14 days. Thrombocytopenia, hemoabdomen, decreased hematocrit, diagnosis of hemangiosarcoma were associated with an increase in the probability that the patient had short survival, but the influence were not statistically significant (*p* > 0.05). The overall three‐month, half‐year, and one‐year survival rates of affected dogs were 50.0% (21/42), 33.3% (13/39), and 23.1% (9/39), respectively. For those who didn’t have metastasis disease at diagnosis, the one‐year survival rate was 42.9% (9/21).

**Conclusions and Clinical Importance:** A certain amount of patients didn’t survive the first two weeks after surgery, further studies are warranted to investigate the potential cause. The overall prognosis of dogs with splenic tumors was guarded, but for cases without metastasis at diagnosis, the prognosis was good.

## 290 ABSTRACT O15: Innovative Development of Organoids From Canine Mastocytomas: A Novel *In Vitro* Model Background

### 290.1 **Andre Meneses** ^1^, DVM, PhD; Pedro Almeida^2^; Adriana Belas^2^; Jorge Braga^3^; Maria Catarina Caldeira^3^, Sónia Campos^2^, Francisco Cruz^3^, Sabrina Legatti^2^, Pamela Lona^3^, Joana Oliveira^2^

#### 290.1.1 ^1^Auxiliary Professor, Faculty of Veterinary Medicine, Lusofona University of Lisbon, Lisbon, Portugal; ^2^Professor, Faculty of Veterinary Medicine, Lusofona University, Lisbon, Portugal; ^3^Student, Faculty of Veterinary Medicine, Lusofona University, Lisbon, Portugal

Canine mastocytomas are the most common cutaneous malignancy in dogs, providing a valuable model for studying mast cell tumor biology. Organoid technology has revolutionized cancer research by creating *in vitro* models that recapitulate tumor behavior and microenvironments. However, descriptions of organoids from canine mastocytomas are notably absent. This study addresses this gap by isolating and developing a specialized culture medium to sustain those organoids growth and morphology. The objective was to establish a reliable protocol for isolating these organoids and to develop a culture medium that supports their rapid growth and structural integrity. Tumor samples were obtained from three dogs with mastocytomas confirmed by histopathology. Ethical guidelines were followed, and informed owner consent was obtained. Tumor tissues were enzymatically and mechanically dissociated into single‐cell suspensions and embedded in a 3D extracellular matrix. Organoids were cultured in a medium composed of Advanced DMEM/F12 supplemented with B27, Glutamax, N2, Hepes, R‐spondin, Wnt‐CM, FGF, NOG, EGF, A83, Y‐27632, and SB202190. Growth was monitored daily, focusing on time to visible formation, confluence, and morphology. Organoids were visible 48 h post‐culture initiation in all three samples. The tailored medium supported rapid growth, reaching high confluence within 7 days. Morphological assessments revealed that organoids exhibited spheroidal structures and dense cellular clusters under phase‐contrast microscopy. This study represents a pioneering effort to establish organoids from canine mastocytomas, highlighting the novelty and significance of this work, which provides a foundation for future investigations into tumor biology, drug discovery, and personalized treatment approaches in both veterinary and comparative oncology.

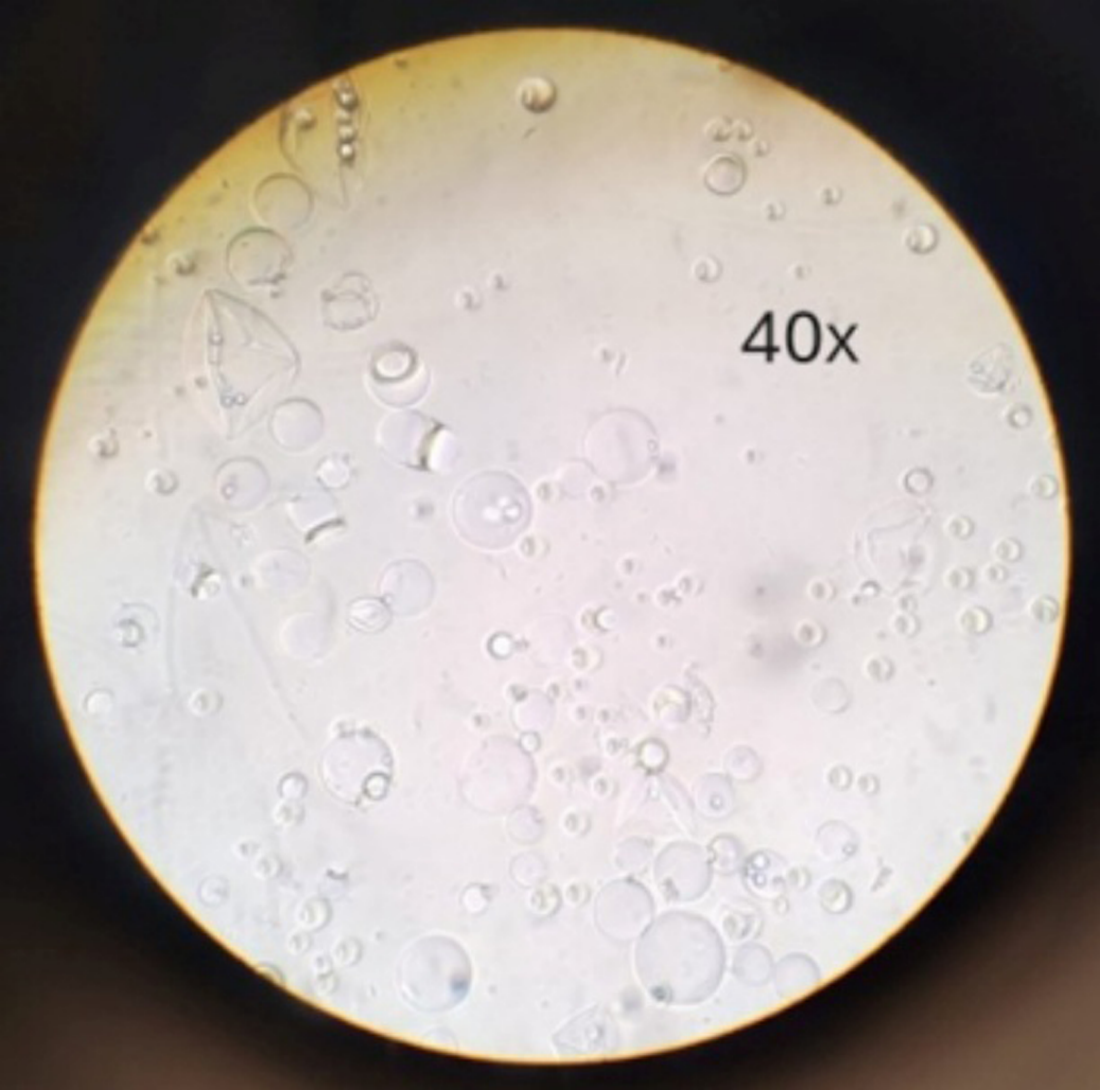

## 291 ABSTRACT O16: Auranofin Induces Apoptosis and Contributes to Paraptosis‐Like Cell Death in Malignant Canine Mammary Tumor Cells

### 291.1 **Yoon‐Ho Suh** ^1^, DVM; Se‐Hoon Kim^2^, DVM, MS; Ki‐Hoon Song^3^, PhD; Jun‐Yeol Choi^2^, DVM; Min‐Ok Ryu^2^, DVM, PhD; Robert Rebhun^4^, DVM, PhD, DACVIM (Oncology); Kyoung‐Won Seo^2^, DVM, PhD, DAiCVIM (Oncology), DKCVIM (SAIM)

#### 291.1.1 ^1^Graduate Student, Laboratory of Veterinary Internal Medicine, Department of Veterinary Clinical Sciences, College of Veterinary Medicine, Seoul National University, Seoul, South Korea; ^2^Laboratory of Veterinary Internal Medicine, Department of Veterinary Clinical Sciences, College of Veterinary Medicine, Seoul National University, Seoul, South Korea; ^3^Research Institute, ViroCure Inc.; ^4^Department of Surgical and Radiological Sciences, School of Veterinary Medicine, University of California‐Davis, Davis, CA, USA

**Background:** Canine mammary tumors (CMT) are common in female dogs, often exhibiting high malignancy and limited responses. Auranofin (AF), an FDA‐approved drug for human rheumatoid arthritis that is also used in dogs to treat idiopathic polyarthritis, has demonstrated anticancer effects in various cancers.

**Objectives:** To evaluate the anticancer effects and mechanisms of AF in five malignant CMT cell lines, focusing on its ability to induce distinct forms of cell death.

**Methods:** Cells were treated with AF under various conditions, and subsequent assessments included cell viability, clonogenicity, thioredoxin reductase (TrxR) activity, and protein expression. Additionally, live‐cell staining was performed to investigate the origin of cytoplasmic vacuoles.

**Results:** AF inhibited TrxR activity, cell proliferation, and colony formation across malignant CMT cell lines. In AF‐sensitive cell lines, 0.5–2 μM AF induced endoplasmic reticulum (ER) stress‐mediated apoptosis, while concentrations above 3 μM caused near‐complete cell death via additional proteasome inhibition. Therefore, we combined sublethal doses of AF (~2 μM) with the proteasome inhibitor Bortezomib (Bz) in AF‐resistant cell lines. The combination exhibited synergistic cytotoxicity and induced extensive cytoplasmic vacuolation originating from the ER. Cycloheximide pretreatment inhibited both vacuolation and AF + Bz‐induced cell death, indicating features of paraptosis. This cell death was related to enhanced ER stress from AF‐induced TrxR inhibition and Bz‐induced proteasome inhibition.

**Conclusions and Clinical Importance:** We propose AF alone or with Bz as a therapeutic strategy for malignant CMT. This study highlights AF's potential to induce ER stress‐mediated apoptosis and paraptosis‐like cell death, expanding options for canine cancer treatment.

**Figure:**
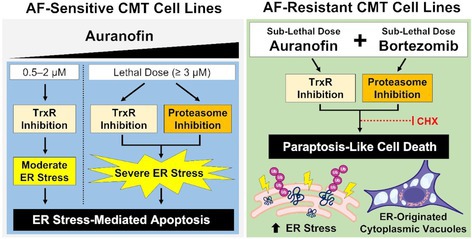
Graphical abstract. A schematic representation summarizes the key findings of the study

## 292 ABSTRACT O17: Environmental Chemical Exposures, Early DNA Damage, and Multicentric Lymphoma in Dogs

### 292.1 **Brian Hansford** ^1^, BS; Ashleigh Tindle^2^; Lauren Trepanier^3^, DVM, PHD, DACVIM(SAIM), DACVCP

#### 292.1.1 ^1^Veterinary Student, University of Wisconsin, Madison, WI, USA; ^2^Research Assistant, Department of Medical Sciences, University of Wisconsin Madison, WI, USA; ^3^Professor, Internal Medicine, Medical Sciences, University of Wisconsin, Madison, WI, USA

Human non‐Hodgkin lymphoma, which resembles canine multicentric lymphoma (CML), is associated with herbicides and volatile organic compounds (VOCs). However, it is not known whether these genotoxic chemicals are associated with CML, or early DNA damage, in dogs. We hypothesized that VOC and herbicide exposures would associate with both early DNA damage and CML in dogs. We utilized banked blood and urine from the Golden Retriever Lifetime Study, in 30 CML cases and 30 matched controls at time of CML diagnosis (T0) and one year prior (T‐1y). We measured urinary concentrations of the herbicides 2,4‐D and glyphosate, and metabolites of the VOCs benzene, xylene, and 1,3‐butadiene in all dogs at T0 and T‐1y. Whole blood DNA strand breaks and oxidized DNA bases were detected with the alkaline comet assay, with or without FpG. DNA damage was compared between cases and controls using Wilcoxon signed ranks tests and was correlated with herbicide and VOC exposures using Spearman rank order. To date, median oxidative DNA damage (as percent comet tail) at T‐1y was 4.6 (0–23.2) in 10 cases and 1.4 (0–14.8) in 10 matched controls (*p* = 0.10). Ongoing assays will further assess oxidative DNA damage in CML prior to diagnosis and its association with herbicide and VOC exposures. If oxidative DNA damage is associated with chemical exposures in dogs that later develop CML, this could support targeted screening and environmental prevention measures in high‐risk breeds.

## 293 ABSTRACT O18: Chimeric Antigen Receptor T Cells Can Be Tracked *In Vivo* and Induce Remission in Dogs

### 293.1 **Jeffrey Bryan** ^1^, DVM, MS, PhD, DACVIM (Oncology); Wesley Wierson^2^, PhD; Alex Abel^3^, PhD; Saad Kenderian^4^, MD, ChB; Elizabeth Siegler^5^, PhD, MS

#### 293.1.1 ^1^Professor, University of Missouri, Columbia, MO, USA; ^2^CEO, LifEngine Animal Health Laboratories Incorporated, Eagan, MN, USA; ^3^CSO, LifEngine Animal Health Laboratories Incorporated, Eagan, MN, USA; ^4^Associate Professor of Medicine, Assistant Professor of Oncology, Assistant Professor of Immunology, T cell Engineering, Division of Hematology, Department of Immunology, Mayo Clinic; ^5^Assistant Professor of Medicine, T cell Engineering, Division of Hematology, Mayo Clinic

**Background:** Chimeric Antigen Receptor (CAR)‐T cell therapy can be curative for human patients with hematologic malignancies and there is great interest in bringing this technology to companion animals with lymphoma. However, considerations in cost, lack of available pre‐conditioning protocols, and potential side effects have limited its application in dogs.

**Objectives:** We describe the use of human T cells engineered with TRAC and B2M CRISPR knockouts, a canine CD20 targeting CAR with the Sodium Iodide Symporter (NIS), and a novel lymphodepletion protocol towards live cell imaging, tracking, and application of xenogeneic CAR‐T cells to spontaneous B cell lymphoma.

**Animals:** Two client‐owned companion dogs with Stage IV‐a CD20+ B cell lymphoma completed this observational protocol.

**Methods:** Dogs received immunosuppressive chemotherapy of 0.5 mg/kg pentostatin for 3 days and, on the 3rd day, 300 mg/m2 cyclophosphamide along with supportive IV fluid and concomitant medications. Three days later, 25 M CAR‐T cells were injected intranodally, and 10 M/kg infused intravenously and later assessed by 18F‐TFB PET 24, 72, and 164 h after treatment.

**Results:** Both dogs reached complete remission during the observation period with resolution of internal and peripheral lymph node enlargement. Moreover, CAR‐T cells are well‐tolerated, and live imaging in dogs is feasible, demonstrating the application of 18F‐TFB PET for future study of CAR‐T dynamics in spontaneous disease.

**Conclusions and Clinical Importance:** Combination cyclophosphamide and pentostatin is a potent yet dose‐limiting lymphodepletion protocol in companion dogs. However, lymphodepletion followed by dual‐administered CAR‐T cells for lymphoma is an effective protocol for both live imaging and clinical response.

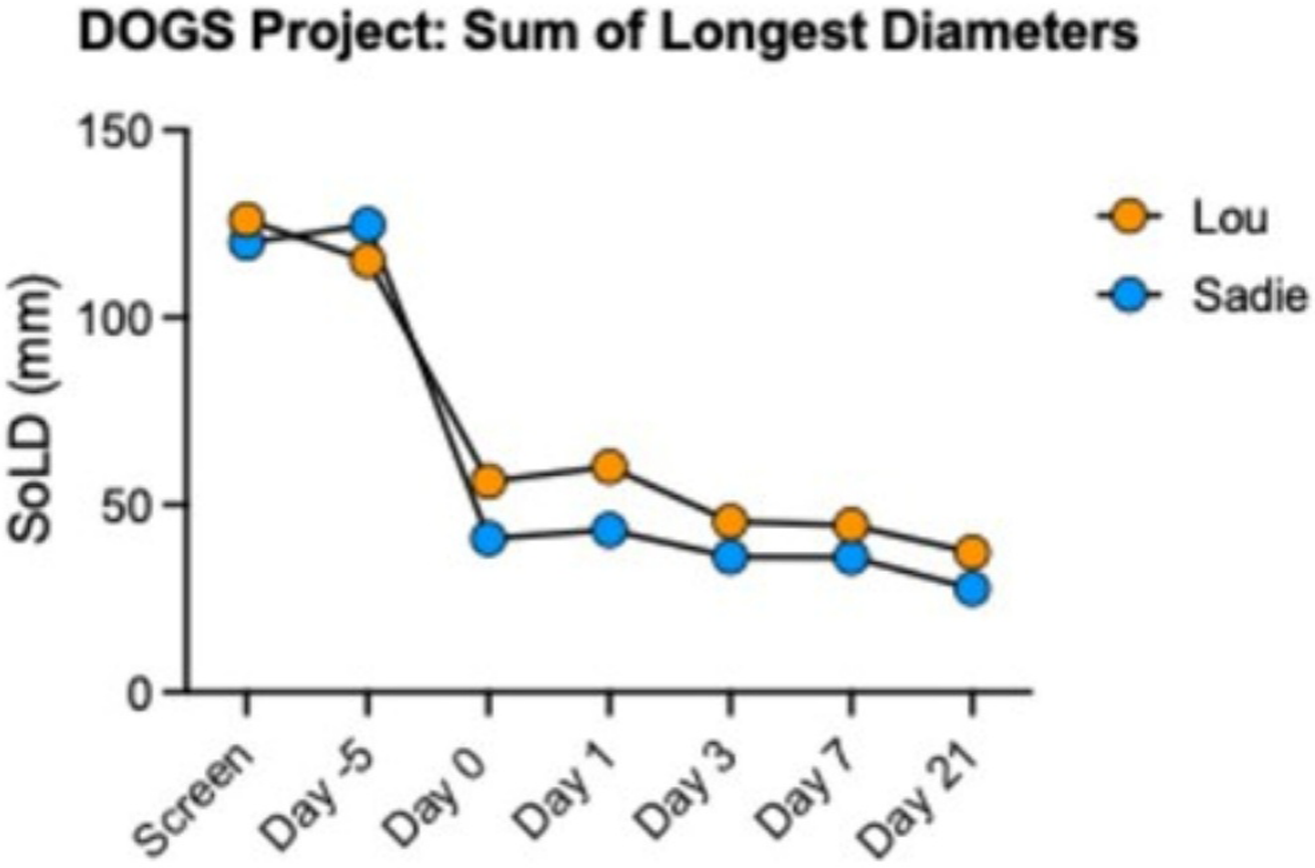

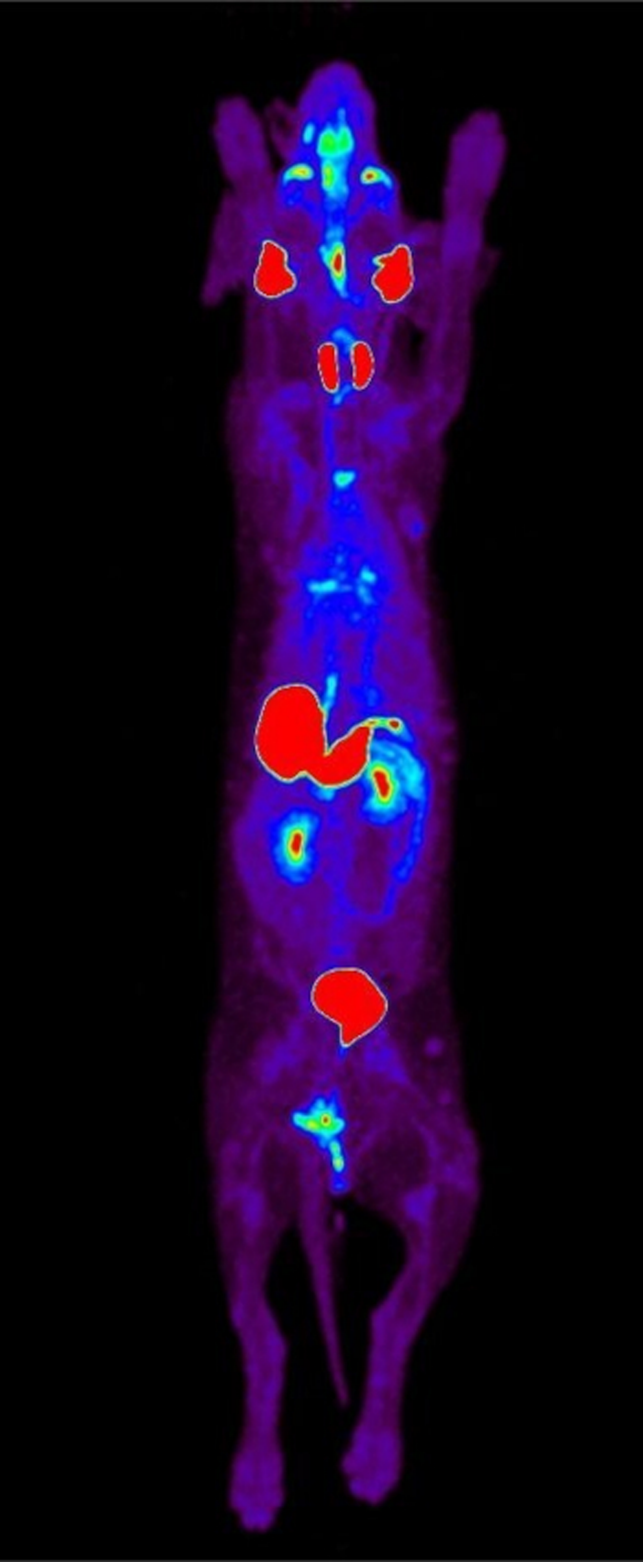

## 294 ABSTRACT O19: Bacterial Extracellular Vesicles Decrease Viability of Canine Osteosarcoma Cells and Modulate T Cell Immune Response

### 294.1 **Christine Chang** ^1^, DVM, DACVIM (Oncology); Aubrie Selmek^2^; Christopher Montgomery^3^, MD; William Kisseberth^2^, DVM, PhD, DACVIM (Oncology); Shay Bracha^2^, DVM, MS, DACVIM (Oncology)

#### 294.1.1 ^1^Graduate Research Associate, Ohio State University, Columbus, OH, USA; ^2^Ohio State University, Columbus, OH, USA; ^3^Nationwide Children's Hospital, Columbus, OH, USA

**Background:** Bacterial infections following limb‐salvage procedure in osteosarcoma patients are associated with improved survival rates, with *Staphylococcus* species being the most common bacterial isolate. Bacterial extracellular vesicles (BEVs) carry cargo including toxins and pathogen‐associated molecular patterns capable of modulating immune responses without causing infection. While BEVs have been studied in infectious diseases, there is limited research on their role in cancer and their therapeutic applications remain underexplored.

**Hypothesis/Objectives:** BEVs from *Staphylococcus aureus* (*S. aureus*) promote osteosarcoma cell death and modulate T cell immune responses.

**Animals:** Blood was collected from three healthy canine donors.

**Methods:** One wild‐type strain of *S. aureus* (USA300) and three engineered strains (agr del, hla del, sae del) engineered to eliminate selected cytotoxins were used. BEVs were isolated using ultracentrifugation and characterized for size and composition with a Nanosight analyzer and immunoblots. Two canine osteosarcoma cell lines (TOB and HMPOS) were incubated with BEVs for 4, 8, and 24 h and subjected to flow cytometry for viability assessment. Peripheral blood mononuclear cells (PBMCs) were isolated, incubated with BEVs for 4, 8, and 24 h, and analyzed for T cell marker expression with flow cytometry.

**Results:** BEVs from the sae del strain decreased viability of canine osteosarcoma cells. Proliferation of the CD8+ T lymphocyte population was seen when PBMCs were incubated with BEVs from the wild‐type USA300 and sae del strain.

**Conclusions/Clinical Importance:** BEVs are capable of decreasing the viability of osteosarcoma cells *in vitro* and should be further investigated as a novel immunotherapy.

## 295 ABSTRACT O20: Innovative Bioanalytical Luminescent Assays for Advancing Veterinary Oncology Therapeutics

### 295.1 **Maria Dashek** ^1^, DVM, PHD, DACPV; Jun Wang^2^; Denise Garvin^2^; Trish Hoang^2^; Rod Flemming^2^; Jeff Nelson^2^; Marjeta Urh^2^; Mei Cong^2^; Jamison Grailer^2^

#### 295.1.1 ^1^Market Development Manager, Animal Health, Promega, Madison, WI, USA; ^2^Promega, Madison, WI, USA

**Background:** The development of biologic drugs for veterinary oncology is increasingly important, requiring precise and reliable bioanalytical tools to ensure their efficacy and safety.

**Objective:** This study aimed to develop *in vitro* bioanalytical assays to support the characterization, potency assessment, and advancement of therapeutic targets in veterinary oncology.

**Methods:** This study utilized cellular and protein engineering approaches to develop two cell‐based reporter bioassays—the Canine ADCC Reporter Bioassay and the Canine PD‐1/PD‐L1 Blockade Bioassay—along with Lumit® Immunoassays for Canine and Feline FcRn receptor binding. These luminescence‐based assays were designed to measure target interactions with high sensitivity and specificity. Analytical performance characteristics, including accuracy, precision, linearity, and specificity, were rigorously assessed following International Council of Harmonization (ICH) guidelines.

**Results:** The cell‐based bioassays demonstrated robust performance, accurately detecting and quantifying the potency of antibodies with high sensitivity and specificity. The Lumit® Immunoassays significantly reduced assay time to less than 70 min compared to traditional ELISAs, which require over 4 h, while maintaining comparable accuracy.

**Conclusions and Clinical Importance:** The advanced bioassays and immunoassays developed in this study provide a reliable, efficient, and comprehensive platform for the characterization and potency assessment of veterinary biologic drugs. By improving the sensitivity, specificity, and reproducibility of bioanalytical tools, these methods enable the development of more effective therapies. This supports advancements in veterinary oncology and better outcomes for companion animals.

## 296 ABSTRACT O21: Pharmacokinetic & Safety Assessment of a Novel Anti‐CD20 mAb

### 296.1 **Aleksandar Masic** ^1^, DVM, PhD; Dalit Frank^2^; Amanda Guth^3^; Chad Johannes^4,5^; Courtney Sparks^2^; Ruhcha Sutavani^2^; Guncha Taneja^2^; Avian Townley^2^; Juexuan Wang^1^; Adam Werts^1^; Andi Flory^3^

#### 296.1.1 ^1^Senior Principal Scientist, Zoetis Inc.; ^2^Principal Scientist, Zoetis Inc.; ^3^Zoetis Inc.; ^4^Colorado State University; ^5^Flint Animal Cancer Center

**Background:** Lymphoma, is one of the most common cancers in dogs. Treatment outcomes of the corresponding human cancer have improved with the addition of B‐cell‐specific, anti‐CD20 monoclonal antibodies (mAbs). Similar to human medicine, a novel canine anti‐CD20mAb could significantly improve treatment options for dogs with B‐cell lymphoma.

**Hypothesis/Objectives:** A novel anti‐CD20 mAb has been developed with specific binding to canine CD20 and potent killing of target cells in in vitro assays. The purpose of this study was to evaluate the safety and PK/PD of novel anti‐CD20mAb in dogs.

**Animals:** Healthy laboratory beagle dogs (*n* = 23), ~4 years of age, were randomly selected to receive single or multiple doses of the anti‐CD20mAb.

**Methods:** In vivo exposure, peripheral B‐cell depletion, CBC, and serum chemistry were assessed post single‐ (0.2–10 mg/kg SC) and multiple‐dose (0.05–0.2 mg/kg SC or IV) administration. Serum concentrations were coupled with B‐cell kinetics to predict drug effect.

**Results:** Detectable levels of drug were observed post SC administration in serum at all dose levels with a mean half‐life of ~9.9 days and mean bioavailability of 79%. Drug was well‐tolerated at the dose levels tested. With single‐dose administration, CD21+ B‐cells started to decline an 1‐h post‐dose and were completely depleted by 24 h in all dose groups with onset of recovery ~50 days.

**Conclusions and Clinical Importance:** This canine anti‐CD20 mAb has good subcutaneous bioavailability and is well‐tolerated in vivo at the doses tested. This drug may add to the arsenal of available therapeutics for management of canine lymphoma.

## 297 ABSTRACT OT01: Do Owners Know Best? Clinical and Laboratory Findings in Reportedly Healthy Geriatric Cats

### 297.1 Amy Nichelason^1^; Zoe Snyder^2^; Karina Herrera^2^; Ben Veenstra^2^; Starr Cameron^3^, BVetMed, MS, DACVIM (Neurology)

#### 297.1.1 ^1^Clinical Assistant Professor, School of Veterinary Medicine, University of Wisconsin‐Madison, Madison, WI, USA; ^2^School of Veterinary Medicine, University of Wisconsin‐Madison, Madison, WI, USA; Clinical Associate Professor, Neurology, School of Veterinary Medicine, University of Madison‐Wisconsin, Madison, WI, USA

**Background:** Owners do not take their cats to the veterinarian as frequently as recommended, citing concerns including cost, stress to their cat, or a perception of health.

**Objectives:** To evaluate clinical and laboratory findings in healthy geriatric cats (> 12 years of age).

**Animals:** 131 geriatric cats reported healthy by owners were enrolled between 2022 and 2024. Cats with known neoplasia, infectious or neurologic disease were excluded. Of those, 61 cats had more comprehensive examinations and owners completed surveys about their cats’ habits.

**Methods:** All cats had a complete blood count, serum biochemistry, T4, blood pressure, and urinalysis performed. Descriptive analyses and comparisons were performed between clinical and laboratory findings and owner‐reported questionnaires.

**Results:** 75% (46/61) of cats had orthopedic changes on exam, despite 73% of clients (39/53) reporting no orthopedic limitations at home. 24% (14/58) of cats had a newly diagnosed arrhythmia or murmur. 69% (37/39) of cats had dental scoring ≥ 3/6, consistent with moderate unstaged periodontal disease and 34% (21/61) had at least one resorptive lesion. Despite only 24.2% (29/120) of owners reporting historic chronic kidney disease and no owners reporting changed thirst or urination, 75% (73/97) of cats had urine specific gravity < 1.035, and 58.5% (69/118) exhibited creatinine levels > 1.6 mg/dL. Additionally, 44.5% (53/119) of cats were hypertensive, with hypertension being severe (> 180 mm Hg) in 37 cats.

**Conclusions:** Our findings demonstrate that apparently healthy geriatric cats may exhibit clinical and laboratory abnormalities warranting intervention. This underscores the importance of routine examinations and laboratory screenings in this population.

## 298 ABSTRACT OT02: Associations of Physical Factors with Gait Speed in Healthy Dogs as a Potential Frailty Evaluation

### 298.1 **Jake Ryave** ^1^; Kelsey Patterson^2^; Kate Creevy^3^, DVM, MS, DACVIM (SAIM); Jessica Hoffman^4^, PhD; Taylor Reiter^3^, DVM; DAP Consortium^3^

#### 298.1.1 ^1^Dog Aging Project Veterinary Fellow, Texas A&M University, College Station, TX, USA; ^2^Augusta University, Augusta, GA, USA; ^3^Texas A&M University, College Station, TX, USA; ^4^Assistant Professor, Department of Biological Sciences, Augusta University, Augusta, GA, USA

**Background:** Gait speed is a marker of frailty and a predictor of mortality in humans. Little information is available on frailty or its predictive value in companion dogs. Variable dog phenotypes complicate assessment of speed.

**Hypothesis/Objectives:** To evaluate gait speed in a population of healthy, adult companion dogs and determine which phenotypic measurements predict gait speed as part of a proposed frailty evaluation.

**Animals:** Reportedly healthy, skeletally mature, client‐owned dogs (≥ 2 years) that completed morphometric and mobility activities as part of the Dog Aging Project (DAP) (*n* = 3,412).

**Methods:** Body condition scores (BCS) were extracted from medical records submitted by DAP participants. Annually, each DAP participant provides their dog's weight and is asked to perform and report 1) eight unique measurements of their dog, and 2) a timed off‐leash ten‐meter run. Pearson's product‐moment correlation was used to assess associations between continuous measurements and speed.

**Results:** Measurements and speed were available for 1374 dogs between 2 and 6 years. Six of the eight unique measurements and body weight were positively correlated with gait speed, with body weight being the primary driver of the relationship. BCS was not correlated with speed. Among all dogs, a broken‐line analysis found a significant decline in gait speed at 8.37 years.

**Conclusions and Clinical Importance:** Owner‐reported gait speed may be a marker of frailty in otherwise healthy dogs. Given variable dog phenotype, the ability to use weight alone to interpret speed simplifies use of this practical, low‐tech assessment.

## 299 ABSTRACT OT03: The Effects of Intraoperative Intraperitoneal Fluid Administration on Perfusion Parameters in Pregnant Queens Undergoing Ovariohysterectomy

### 299.1 **Joseph Frame** ^1^; Nolan Chalifoux^2^, DVM, MSTR, DACVECC; James Fudge^3^, DVM, MPVM, DACVECC; Rebecka Hess^2^, DVM, MSCE, DACVIM (SAIM); Deborah Silverstein^2^, DVM, DACVECC

#### 299.1.1 ^1^Resident, Small Animal Emergency and Critical Care, University of Pennsylvania, Philadelphia, PA, USA; ^2^University of Pennsylvania, Philadelphia, PA, USA; ^3^Hill County Animal League

**Background:** High‐volume spay‐and‐neuter clinics in the United States decrease euthanasia rates and limit further feral population growth. Although intravascular (IV) fluid therapy is ideal during anesthesia and surgery, it is often impractical in high‐volume, low‐cost settings. Intraperitoneal (IP) fluid administration is an alternative fluid route in cats. Effects of IP fluid administration on perfusion parameters in late‐term pregnancy queens undergoing an ovariohysterectomy (OHE) are unknown.

**Hypothesis/Objectives:** Intraoperative IP crystalloid fluid administration reduces the occurrence of postoperative hypotension and improves markers of perfusion in the postoperative period in late‐term pregnant queens undergoing OHE.

**Animals:** 50 client‐owned/feral late‐term pregnant queens; 25 IP fluid and 25 control cats included.

**Methods:** Prospective randomized case‐control study. IP fluids were administered in volumes equal to 25% of the gravid uterus weight intraoperatively. Perfusion parameters, including blood lactate, Doppler blood pressure (DBP), and intra‐abdominal pressure (IAP), were recorded pre‐operatively, intraoperatively, and 10 min postoperatively.

**Results:** No difference was detected between pre‐, intra‐, and postoperative perfusion parameters or IAP between IP and control groups (mean DBP (mm Hg) 84.48 vs. 82.4, 73.83 vs. 70.04, and 76.78 vs. 71.52, median lactate (mmol/L) 1 vs. 1, 0.8 vs. 0.8, and 0.8 vs. 0.8, and median IAP (mm Hg) 9 vs. 10, 7.5 vs. 7.5, and 6.75 vs. 6.5), respectively.

**Conclusions and Clinical Importance:** No significant differences in perfusion parameters were observed, however, IP fluid administration may still be a viable option for fluid administration in high‐volume surgical settings. Further veterinary studies evaluating the potential benefits of the IP fluid route are warranted.

## 300 ABSTRACT OT04: Assessment of *Peptacetobacter hiranonis*’ *In Vitro* Ability to Convert Primary into Secondary Bile Acids

### 300.1 **Bruna Correa Lopes** ^1^; Luis da Costa Medina^2^, PhD; Amanda Blake^2^, PhD; Jonathan Turck^2^; Paula Giaretta^2^, DVM, PhD, DACVP; M. Katherine Tolbert^2^, DVM, PhD, DACVIM (SAIM, SA nutrition); Jan Suchodolski^2^, MedVet, DrVetMed, PhD, AGAF, DACVM; Rachel Pilla^3^, DVM, PhD

#### 300.1.1 ^1^PhD Student, Texas A&M University, College Station, TX, USA; ^2^Gastrointestinal Laboratory, Department of Small Animal Clinical Sciences, Texas A&M University, College Station, TX, USA; ^3^Department of Veterinary Pathology, Hygiene, and Public Health, University of Milan, Milan, Italy

**Background:** Dysmetabolism of bile acids (BAs) and intestinal dysbiosis are associated with lower fecal *Peptacetobacter* (*Clostridium*) *hiranonis* abundance in dogs and cats with chronic enteropathies or following antibiotic treatment. BA dysmetabolism, defined by a reduced conversion of primary (PBAs) to secondary BAs (SBAs), has been correlated with inflammation, increased intestinal permeability, and malabsorption. *P. hiranonis* is the main species converting PBAs into SBAs in dogs and cats. However, the ability of *P. hiranonis* to convert BAs *in vitro* has not been characterized.

**Objective:** Assess the *in vitro* BA conversion ability of *P. hiranonis*.

**Animals:** Fecal canine‐ (*n* = 13) and feline‐derived (*n* = 11) strains of *P. hiranonis*.

**Methods:** *P. hiranonis* strains, identified by qPCR, were incubated in BHI broth with PBAs (cholic acid (CA) and chenodeoxycholic acid (CDCA)) for 24 h at 37°C in anaerobic conditions. Conversion ability was assessed through PBAs and SBAs (deoxycholic acid (DCA), lithocholic acid (LCA)) quantification by LC‐MS/MS assay.

**Results:** All canine‐ and feline‐derived strains were able to convert CA and CDCA into DCA and LCA, respectively. Additionally, conversion to oxidized forms of PBAs, 7‐oxo‐DCA and 7‐oxo‐LCA, was observed.

**Conclusions and Clinical Importance:** All tested strains demonstrated the ability to convert PBAs into SBAs. The *in vitro* BA conversion ability of *P. hiranonis* supports its role in the *in vivo* conversion of BAs. It also strengthens the reported correlation between *P. hiranonis* and SBAs in the feces of companion animals, where conversion of BAs was not identified in dogs and cats lacking *P. hiranonis*.

## 301 ABSTRACT OT05: Analysis of Beta‐Adrenergic Receptor Expression in Canine Splenic Hemangiosarcoma

### 301.1 **Kiyeon Son** ^1^, DVM; Youngmi Youk; Mungyu Song; Hyoyoung Jung; Joonghyun Song

#### 301.1.1 ^1^Student, Chungnam National University; ^2^Chungnam National University

**Background:** Hemangiosarcoma is a highly aggressive vascular tumor in dogs, similar to human angiosarcoma. Beta‐adrenergic receptors (β‐ARs) are known to be significantly expressed in angiosarcoma, and β‐AR blockers are under investigation for therapeutic applications.

**Hypothesis/Objectives:** This study aimed to evaluate the expression of β1‐AR and β2‐AR in canine splenic hemangiosarcoma.

**Animals:** Splenic tissue samples were obtained from ten client‐owned dogs, consisting of four with splenic hemangiosarcoma and six with non‐neoplastic disorders, including nodular hyperplasia, lymphocytic proliferation, and extramedullary hematopoiesis.

**Methods:** Formalin‐fixed, paraffin‐embedded splenic tissues were analyzed using immunohistochemistry (IHC) with primary antibodies for β1‐AR and β2‐AR. Immunostaining was evaluated at 400× magnification by averaging 10 fields per sample. The IHC score was calculated by multiplying the staining intensity with the percentage of positively stained cells. Group comparisons were performed using the Mann‐Whitney U test.

**Results:** The IHC scores for β1‐AR and β2‐AR were significantly higher in splenic hemangiosarcoma tissues compared to non‐neoplastic splenic tissues. Statistical analysis confirmed a significant upregulation of β1‐AR (*P* = 0.0095) and β2‐AR expression (*P* = 0.0048).

**Conclusions and Clinical Importance:** The significant overexpression of β1‐AR and β2‐AR suggests a potential role for β‐AR in canine splenic hemangiosarcoma. These findings provide novel insights into β‐AR‐associated mechanisms underlying the pathogenesis of this malignancy, emphasizing their potential as therapeutic targets for future investigation.

**Figure:**
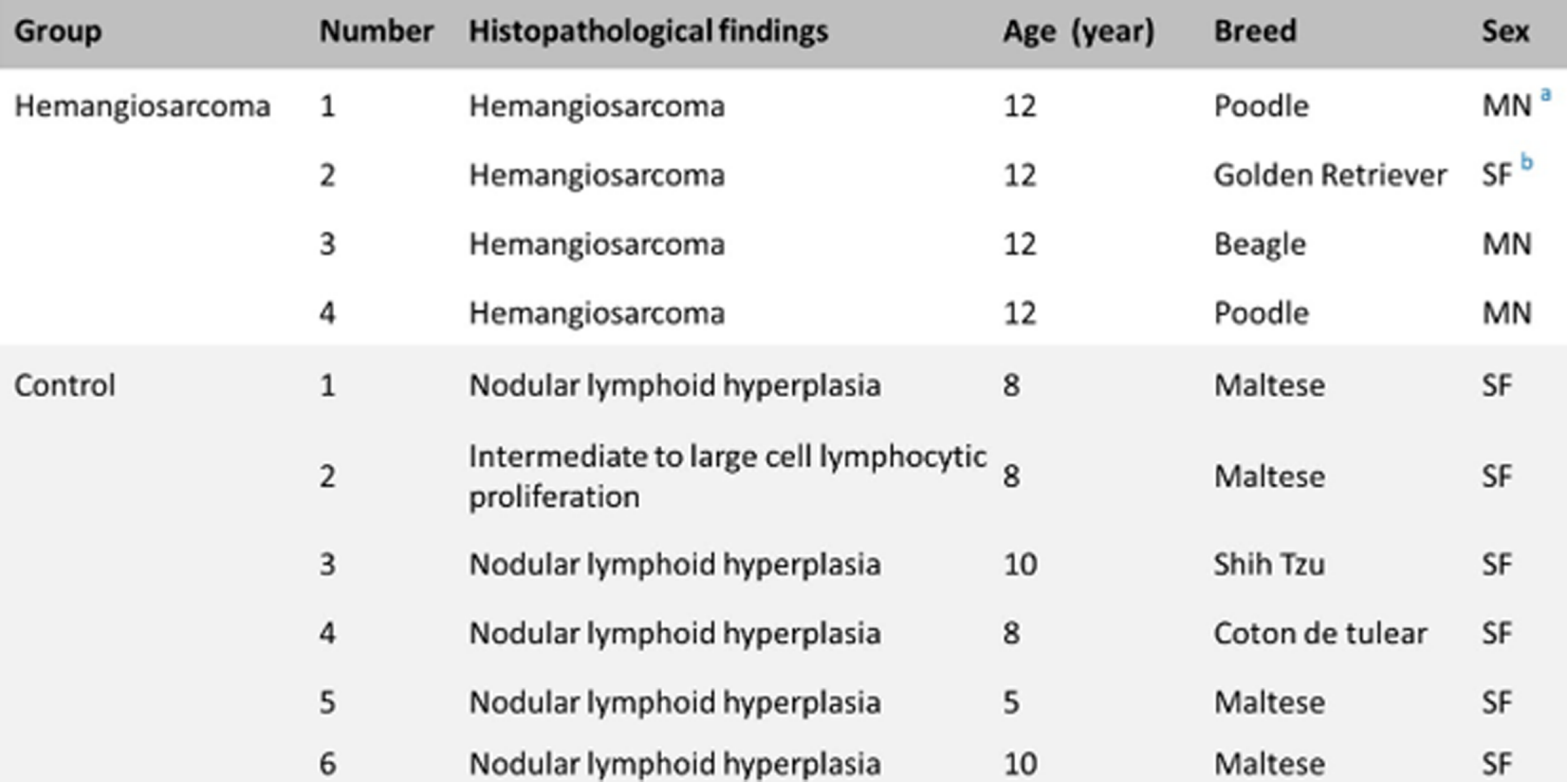
**Table 1. Signalment of dogs with hemangiosarcoma and control dogs**. ^a^MN, neutered male; ^b^SF, spayed female

**Figure:**
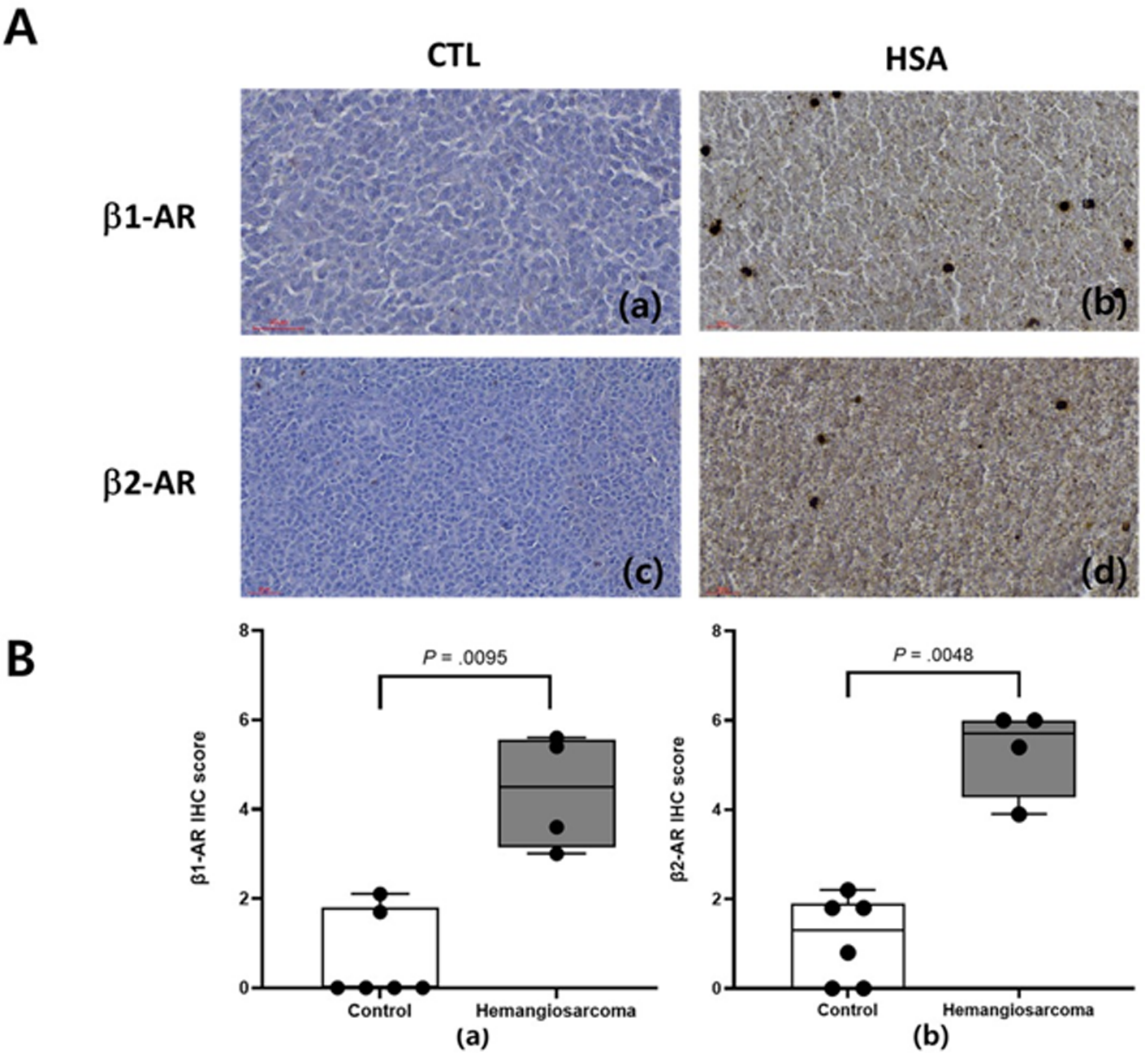
**Figure 1. Immunohistochemical assessment for expression of beta‐adrenergic receptors in canine hemangiosarcoma**. (A) Representative image of β1‐AR (a and b), and β2‐AR (c and d) staining; hematoxylin counterstain; scale bar 30 μm. The staining intensity was evaluated using the following criteria: 0 = undetectable staining, 1 = weak staining, 3 = moderate staining, and 4 = strong staining. (B) Quantitative IHC scoring of β1‐AR and β2‐AR expression in control (CTL) and hemangiosarcoma (HSA) tissues. Data are presented as median with interquartile range. Statistical analysis was performed using the Mann‐Whitney U test. Significant differences are indicated with *p* < 0.01 (**).

## 302 ABSTRACT OT06: Delineating the Fecal Microbiome of Healthy Domestic Short‐Hair Cats in South Korea

### 302.1 **Hyunyoung Cho** ^1^, DVM; Hyung‐Joon Park^2^, PhD; Jin‐Sik Choi^2^; Se‐Hoon Kim^3^, DVM, MS; Min‐Ok Ryu^3^, DVM, PhD; Kyoung‐won Seo^3^, DVM, PhD, DAiCVIM (Oncology), DKCVIM (SAIM)

#### 302.1.1 ^1^Graduate Student, Laboratory of Internal Medicine, College of Veterinary Medicine and Research Institute for Veterinary Science, Seoul National University; ^2^UB Animal R&D Center, GRASSMEDI Co, Ltd., Republic of Korea; ^3^Laboratory of Veterinary Internal Medicine, Department of Veterinary Clinical Sciences, College of Veterinary Medicine, Seoul National University

**Background:** The gut microbiome is essential for metabolism, immune function, and overall balance. In veterinary medicine, it plays a key role in advancing our understanding and management of feline health and disease.

**Hypothesis/Objectives:** This study aimed to characterize the gut microbiota of healthy domestic short‐hair cats in South Korea and evaluate the effects of age, body condition score (BCS), sex, and diet on microbial composition.

**Animals:** Forty healthy domestic short‐haired cats were included.

**Methods:** Fecal samples were analyzed via Quant‐IT PicGreen for DNA quantification. Libraries targeting V3–V4 regions were prepared using the Illumina 16S metagenomic protocol and sequenced on the MiSeq™ platform (2×300 bp). Microbial composition was assessed by age, sex, diet, and BCS at phylum, genus, and species levels.

**Results:** A total of 2,721 bacterial ASV were identified, with Bacillota, Bacteroidota, and Actinomycetota as predominant phyla. Alpha diversity showed no significant age‐related differences but tended to increase in cats aged 7–14 years. *Phocaeicola* abundance correlated with age, and Verrucomicrobiota and *Ruminococcus torque* were associated with higher BCS. Neutered males had elevated Pseudomonadota, *Finegoldia magna*, and *Sutterella massiliensis* levels. Dietary analysis revealed increased *Blautia* and *Lachnoclostridium* in cats fed both dry and wet food.

**Conclusions and Clinical Importance:** This study offers insights into the core microbiota of domestic short‐haired cats in South Korea, highlighting the impact of geographic, physiological, and environmental factors on microbial diversity. These findings underscore the potential of gut microbiome modulation in feline health management, particularly in addressing obesity, aging, and diet‐related health issues.

**Figure:**
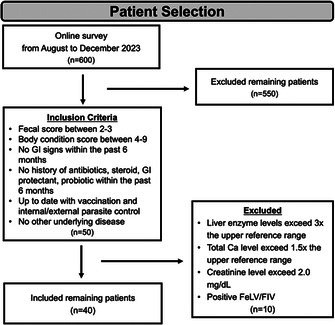
**Figure 1. Scheme of animal selection**. For the body condition score, a 9‐point scale was used, while for the fecal core assessment, a 7‐point scale was utilized. Note: GI, gastrointestinal; BCS, body condition score

**Figure:**
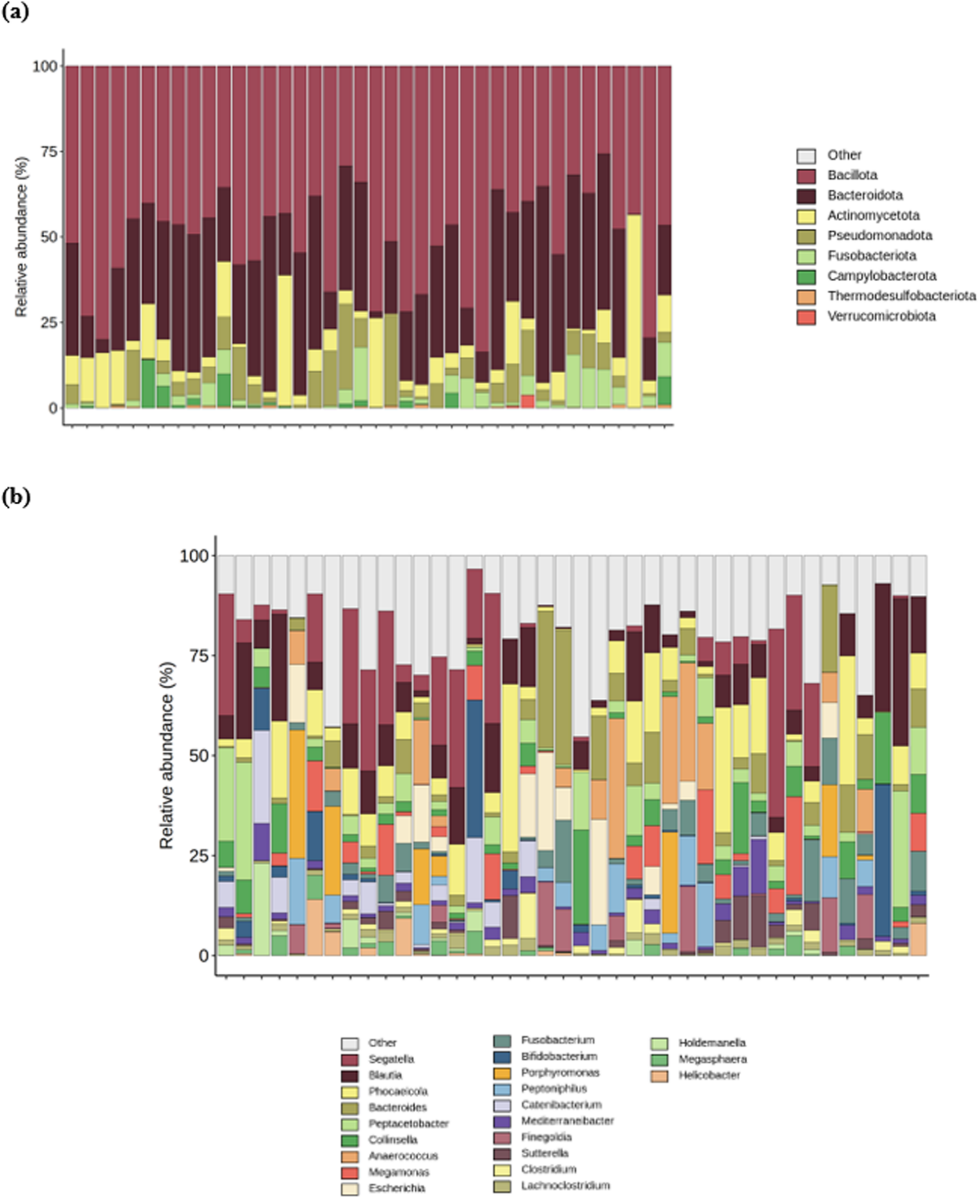
**Figure 2. Comprehensive gut microbiome composition of 40 healthy KSH cats**. Relative abundances of phyla accounting for 0.01% or more of the total sequences. (a) Relative abundances of genera accounting for 1% or more of the total sequences from the eight major phyla. (b)

**Figure:**
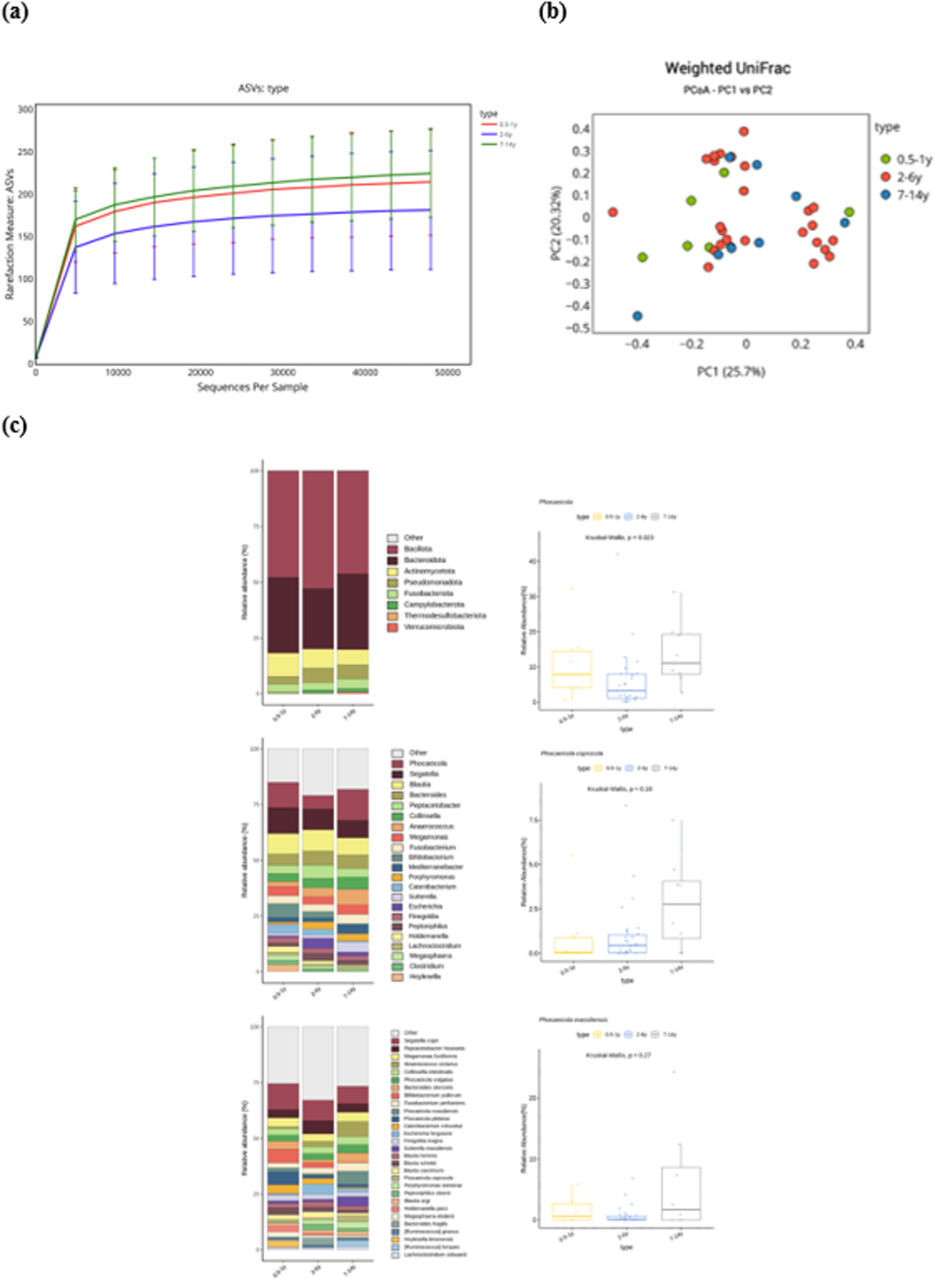
**Figure 3** Alpha and beta diversity were analyzed based on different age groups, with microbial richness assessed through ASV. (a) PCoA was conducted using weighted UniFrac distance. (b) The relative abundances of bacteria at the phylum, genus, and species levels were analyzed based on the age of the cats. Major bacterial groups were represented through chart analysis (phylum ≥ 0.01%, genus ≥ 1.0%, species ≥ 1.0% of total abundance). Box plots were used to illustrate bacterial groups that showed significant differences, and box plots with *p* > 0.05 were shown to indicate the tendency of increase with age. (c)

**Figure:**
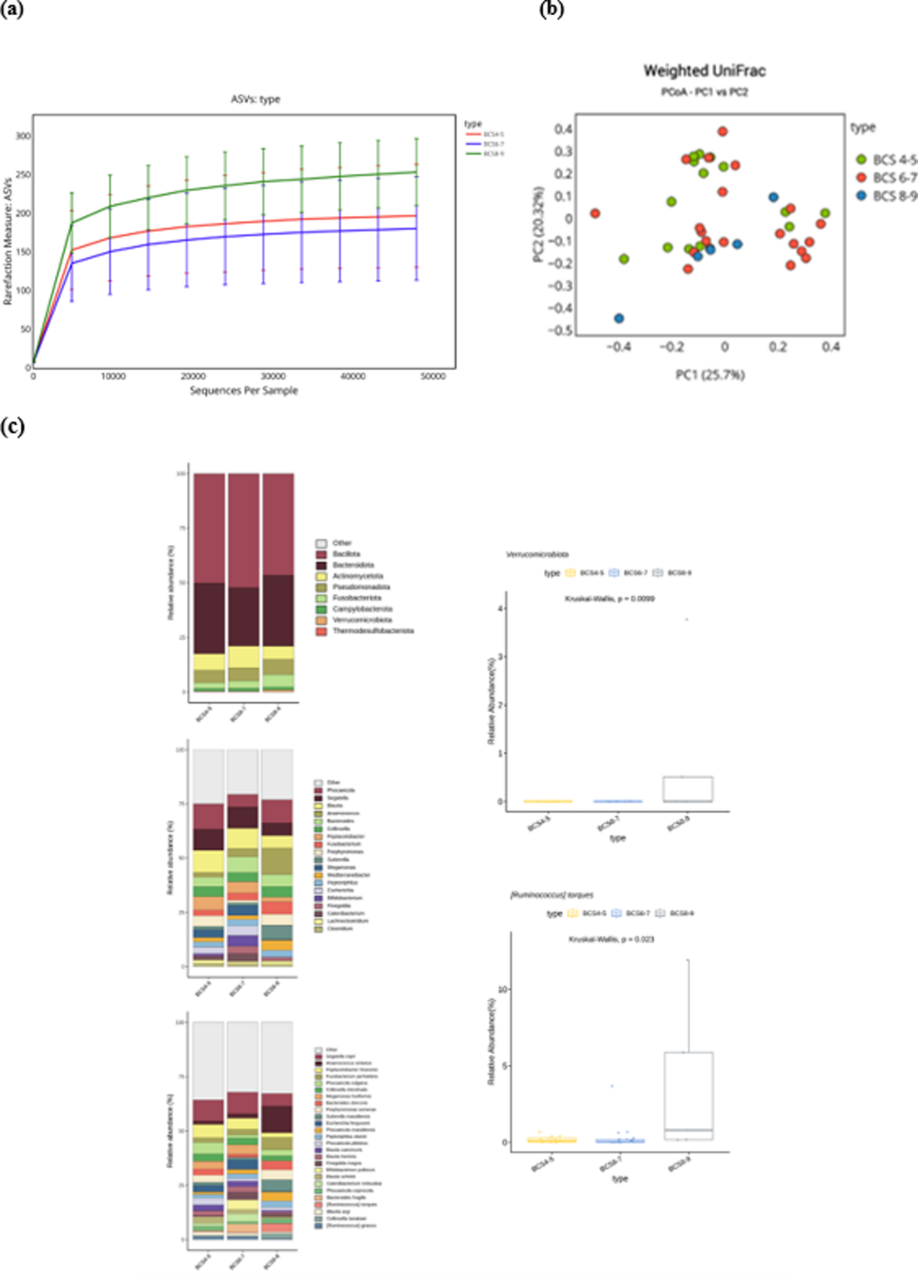
**Figure 4** Alpha and beta diversity were analyzed based on different BCS groups, with microbial richness assessed through ASV (a). PCoA was conducted using weighted UniFrac distance (b). The relative abundances of bacteria at the phylum, genus, and species levels were analyzed based on the BCS of the cats. Major bacterial groups were represented through chart analysis (phylum ≥ 0.01%, genus ≥ 1.0%, species ≥ 1.0% of total abundance). Box plots were used to illustrate bacterial groups that showed significant differences (c).

**Figure:**
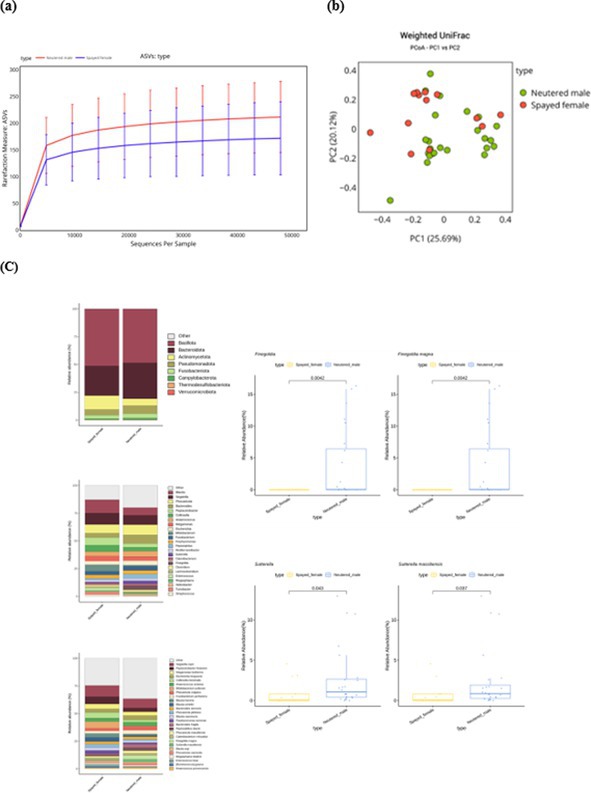
**Figure 5** Alpha and beta diversity were assessed according to different sex groups, with microbial richness evaluated using ASV (a). Weighted UniFrac distance was used to conduct the PCoA analysis (b). The relative abundances of bacteria were evaluated at the phylum, genus, and species levels based on the sex of the cats. Major bacterial groups were visualized through charts, including phyla (≥ 0.01%), genera (≥ 1.0%), and species (≥ 1.0%) representing total abundance. Box plots were utilized to depict bacterial groups that exhibited significant differences (c).

**Figure:**
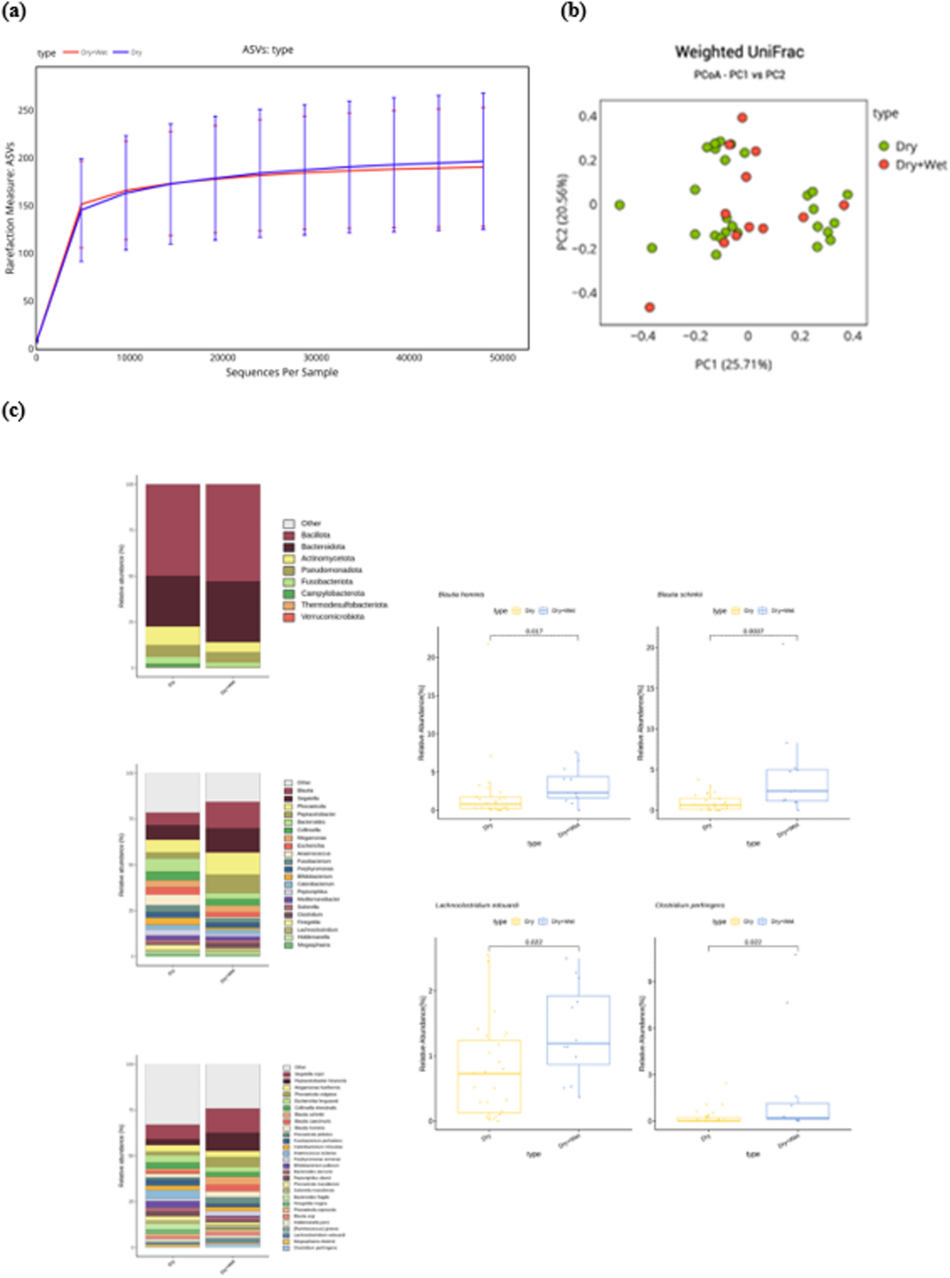
**Figure 6** Alpha and beta diversity were examined across different diet groups, with microbial richness determined through ASV (a). PCoA was performed using weighted UniFrac distance (b). The relative abundances of bacteria were evaluated at the phylum, genus, and species levels based on the diet of the cats. Major bacterial groups were depicted using chart analysis, including phyla (≥ 0.01%), genera (≥ 1.0%), and species (≥ 1.0%) of the total abundance. Box plots were utilized to present bacterial groups that showed significant differences (c).

## 303 ABSTRACT OT07: Participant Compliance With Scheduled Activities in the Test of Rapamycin in Aging Dogs (TRIAD)

### 303.1 **Sydney Holland** ^1^, BS; Anna Ferguson^2^; Shelby Knowles^3^; Rozalyn Anderson^4^; May Reed^5^; Kate Creevy^6^

#### 303.1.1 ^1^Project Manager, Texas A&M University; ^2^Research Study Assistant, Department of Medicine, University of Washington School of Medicine; ^3^Program Operations Specialist, Department of Medicine, University of Washington School of Medicine; ^4^Professor of Medicine, Department of Medicine, University of Wisconsin–Madison; ^5^Associate Professor of Medicine, Department of Medicine, University of Washington School of Medicine; ^6^Professor of Small Animal Internal Medicine, Department of Small Animal Clinical Sciences, Texas A&M University

**Background:** The Dog Aging Project's Test of Rapamycin in Aging Dogs (TRIAD) is a randomized clinical trial of low‐dose rapamycin with lifespan as the primary endpoint.

**Hypothesis/Objectives:** To assess factors that may influence trial protocol compliance.

**Animals:** Healthy companion dogs, ≥ 7 years, 20–55 kg, who have completed the first 12 months of the protocol (*N* = 120).

**Methods:** Completion of trial visits, monthly at‐home observation (MAHO) surveys, biannual quality of life (QOL) surveys, and adherence to study medication protocol within the first year of enrollment were analyzed. Factors including clinical trial site, distance from site, and owner age group at enrollment were assessed to understand possible relationships with compliance. Dogs were censored at the point of withdrawal (*N* = 2) or death (*N* = 9).

**Results:** Mean completion rates of MAHO and QOL surveys were 86.6% (SD 22.5%) and 91.0% (SD 19.4%), respectively. The largest owner age group was 55–64 years (36.7%), with the remainder ranging from 25 to ≥ 75 years. Median distance from site was 106.65 miles (IQR 51.5–185.5). Completion of 6‐month and 12‐month clinical site visits were 98.2% and 97.2%, respectively (*N* = 113, *N* = 106). Examined factors did not reveal an influence on compliance with study protocol within the first year of enrollment in TRIAD.

**Conclusions and Clinical Importance:** Compliance with the TRIAD clinical protocol during the treatment period was very high. Continued study of factors that may influence compliance in the treatment and post‐treatment periods will be beneficial to TRIAD and other long‐term veterinary clinical trials.

## 304 ABSTRACT OT08: Dogs and Cats Have Differential Immunometabolic Responses to Lipopolysaccharide in Cultured Circulating Cells

### 304.1 **Matthew Jackson** ^1^, PhD; Kiran Panickar^2^, PhD; Dennis Jewell^3^, PhD

#### 304.1.1 ^1^Senior Principal Scientist, Hill's Pet Nutrition; ^2^Director, Life Sciences Laboratory, Research and Innovaiton, Hill's Pet Nutrition; ^3^Adjunct Faculty Member, Grain Science and Industry, Kansas State University

**Background:** Immunometabolic responses of cats and dogs to lipopolysaccharide (metabolic endotoxemia; MetTox) is unknown, despite MetTox being associated with disease. To assess responses of cats and dogs without exposure to lipopolysaccharide, cultured whole blood was subjected to lipopolysaccharide challenge *ex vivo* and analyzed by untargeted metabolomics.

**Objective:** Assess *ex vivo* whole blood immunometabolic response of both species to lipopolysaccharide.

**Animals:** Adult dogs (6) and cats (6) housed at Hill's Pet Nutrition Center.

**Methods:** Blood drawn according to animal welfare protocols, incubated ±lipopolysaccharide. After 24 or 48 h, supernatants were collected for metabolomic analysis. Metabolites analyzed using a split‐plot ANOVA with hour (whole‐plot), lipopolysaccharide treatment and interaction (subplot).

**Results:** Metabolites classified into pathway analysis. Fatty acids decreased in response to lipopolysaccharide in cats but not dogs. In response to lipopolysaccharide both species declined in glucose and increased in lactate while pyruvate increased only in dog. With lipopolysaccharide, both species increased in tryptophan metabolites including kynurenine (dogs) and kynurenate (cats). Also, with lipopolysaccharide dogs had decreased betaine and S‐adenosylhomocysteine while cats had only increased S‐adenosylhomocysteine. Both species had decreased pyridoxine in the presence of lipopolysaccharide, while only cats had increased levels of the immune modulator itaconate.

**Conclusions:** The cat is more reliant on fatty acids in a heightened immune response using *ex vivo* challenge and may benefit from decreased dietary carbohydrate and increased fat as compared to the dog. Both dogs and cats have demands on single carbon metabolism and may benefit from increased dietary betaine for optimal immune response.

## 305 ABSTRACT OT09: Multiomic Analysis of Canine Aging Uncovers Conserved Aging Pathways

### 305.1 **Matthew Peloquin** ^1^, BS, MS; Steven Annan^2^; Hubert Chen^2^; Phil Frankino^2^; Jessica Graves^2^; Zane Koch^2^; James McMahon^2^; Ashley Tovar^2^

#### 305.1.1 ^1^Senior Director, Research, Cellular Longevity, Inc. dba Loyal; ^2^Cellular Longevity, Inc. dba Loyal

Aging is a complex process characterized by molecular alterations at numerous biological scales. While these alterations have been extensively studied in humans and rodents, the molecular changes associated with aging in dogs remain less studied. Multiomic profiling methods, such as transcriptomics and proteomics, allow for the interrogation and quantification of transcriptional pathways and bioactive proteins within a desired population. In this study, we sampled 29 female laboratory beagles ages 3 to 14 years old and performed untargeted transcriptomics and proteomics on whole blood and plasma, respectively. In total, 18,451 genes and 7,596 proteins were detected and analyzed. Comparing young (3–5 years old, *N* = 5), old (8–9 years old, *n* = 12), and geriatric (10–14 years old, *n* = 12) dogs, we identified 374 genes and 175 proteins which significantly changed in abundance with age (*p* < 0.05). These changes converged on common pathways, including DNA repair, collagen processing, and metabolic dysregulation. Notably, we observed a significant overlap between age‐associated genes in dogs and those previously identified in humans. In recapitulating key hallmarks of human aging, our findings reinforce the existence of shared aging pathways across species.

## 306 ABSTRACT OT10: Owner‐Reported Health Conditions in UK Miniature Schnauzers

### 306.1 **Arielle Johnson‐Pitt** ^1^, BSc (Hons), BVetMed, MRCVS; Cathryn Mellersh^2^; Brian Catchpole^3^, BVetMed, MSc, PhD, FRCVS; Lucy Davison^4^, MA, VetMB, PhD, DSAM, DECVIM‐CA, MRCVS

#### 306.1.1 ^1^PhD Student, Royal Veterinary College; ^2^Canine Genetics Centre, University of Cambridge; ^3^Professor of Companion Animal Immunology, Department of Pathobiology and Population Sciences, Royal Veterinary College; ^4^Professor of Veterinary Clinical Genetics, Department of Clinical Science and Services, Royal Veterinary College

**Background:** Veterinary epidemiological studies have demonstrated high prevalence of particular health conditions in the miniature schnauzer (MS); however, previous work has not evaluated owner‐reported conditions in this breed.

**Hypothesis/Objectives:** Owner‐reported survey data will reveal novel insights into conditions affecting MS from the owner perspective.

**Animals:** An online survey with specific health‐related questions and opportunity for free‐text reporting was open to owners of UK MS for 1 month in 2023.

**Methods:** Data from 4786 respondents to 21 questions of a MS health survey were analysed. Results are reported as median [range]. Data were cleaned, parsed, and visualised using R through RStudio.

**Results:** Results are illustrated in Figure 1. Dermatological, mass‐associated, and allergic disease were the most highly‐reported conditions in free‐text responses. For questions related to specific conditions, the highest prevalence and youngest age‐of‐onset conditions (Figure 2) were heart murmur 5 y [1 m–14 y] and pancreatitis 4 y [5 m–14 y], reported in 4.0% and 4.6% MS respectively. In contrast to pancreatitis, onset of hyperlipidaemia (reported in 0.6%), was significantly later at 7.5 y [1 y–12 y] (*P* = 1.93e–05). Nephroliths/uroliths were reported in 1.2% with age of onset 5 y [5 m–11 y]. Body condition score was most commonly reported as 3/5 (72.9%).

**Conclusions and Clinical Importance:** MS owners report a relatively high prevalence of pancreatitis and heart murmurs. Recognition of pancreatitis typically precedes hyperlipidaemia diagnosis, where the two diseases are both present. Owner‐reporting of nephrolithiasis/urolithiasis is lower than expected from previous studies. In contrast to previous studies indicating a high obesity prevalence in MS, owners do not commonly self‐report obesity in the breed.

**Figure:**
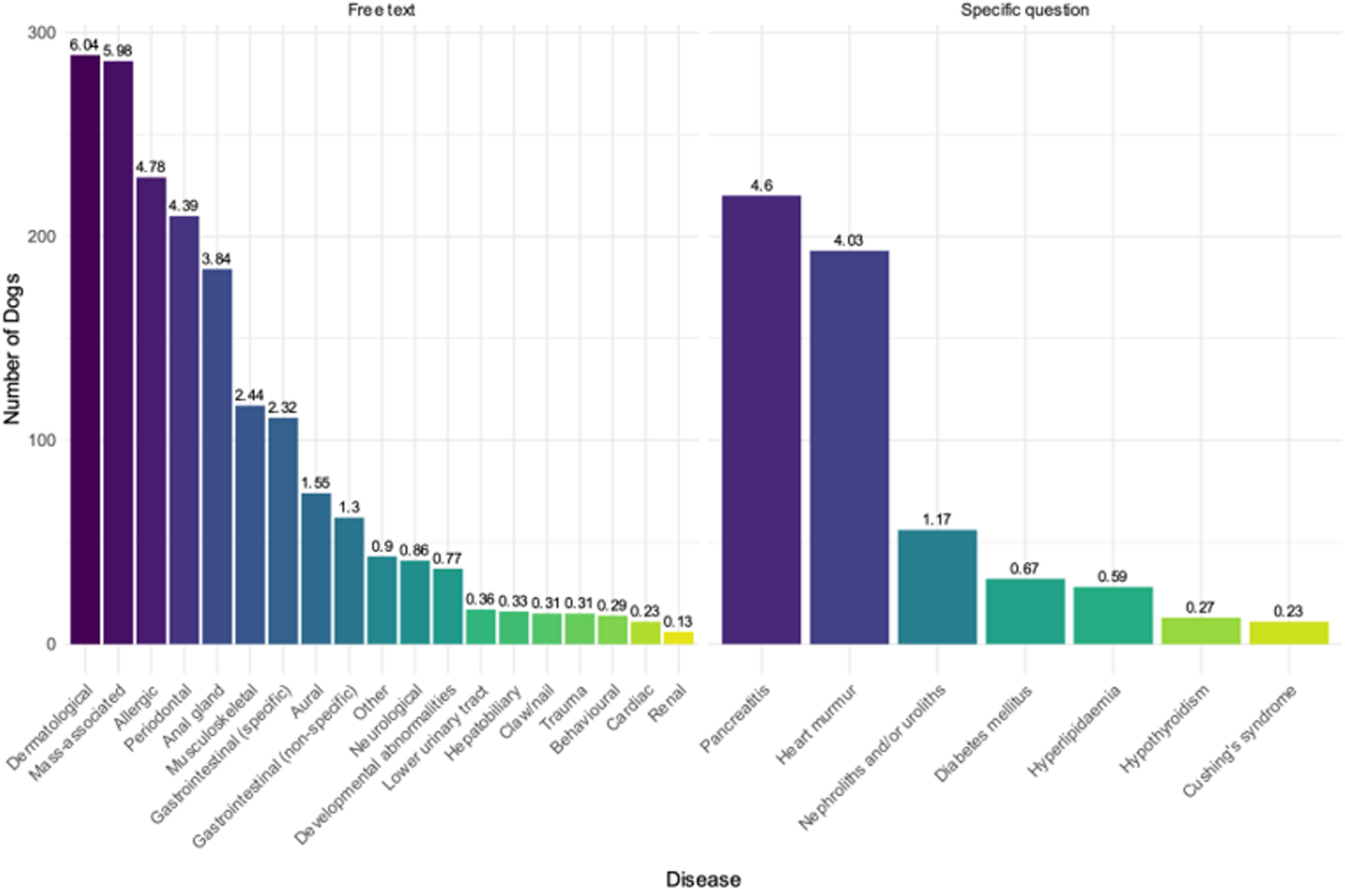
**Figure 1. Comparison of reported disease in miniature schnauzers by specific questioning and free‐text analysis**. Disease frequency reported by miniature schnauzer owners via survey response. Compares disease reports through free‐text analysis (grouped into disease categories) and responses to a specific question format with “Yes”/“No” answers for 7 diseases. Percentages above bars represent the proportion of 4786 total respondents.

**Figure:**
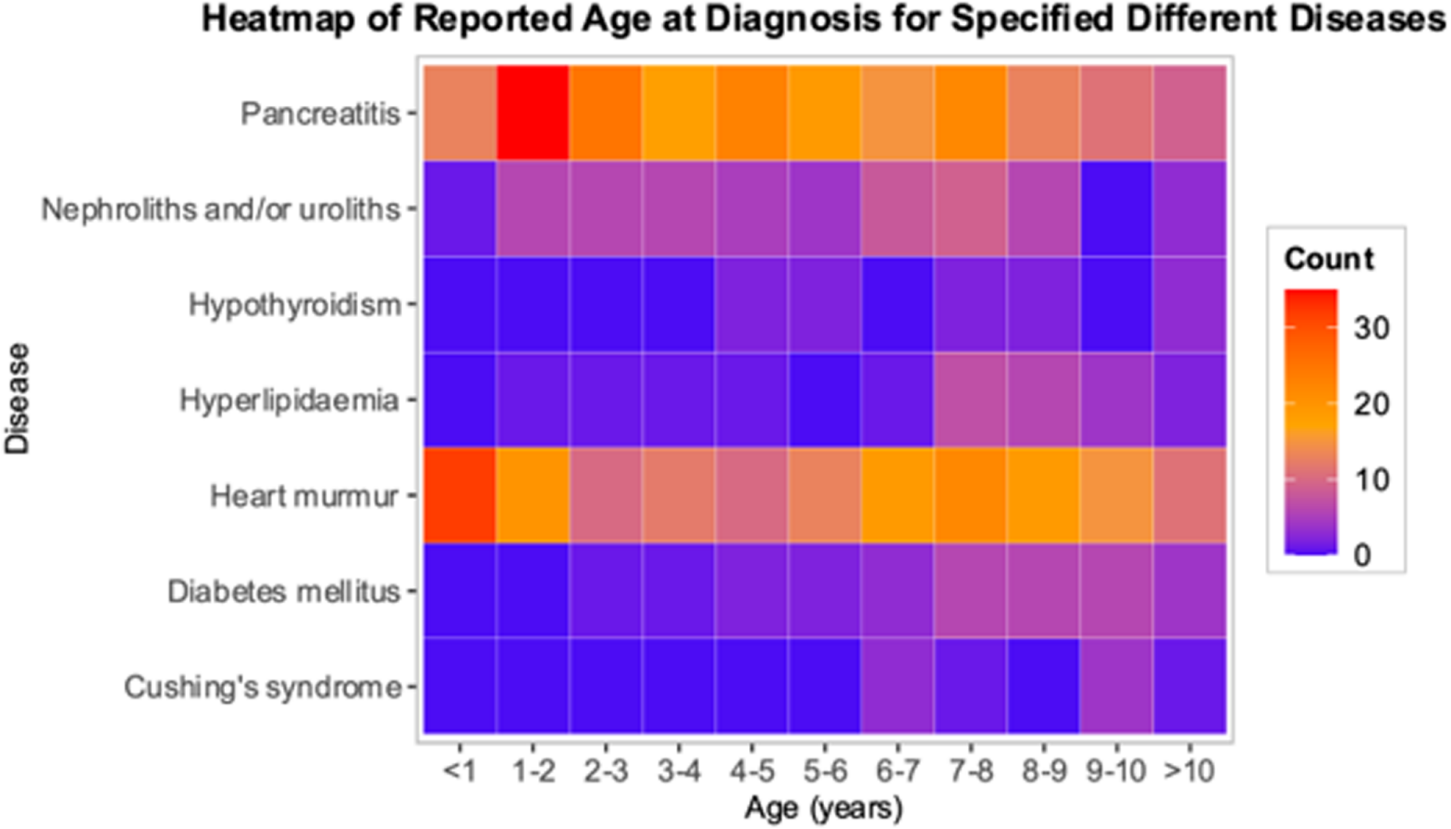
**Figure 2. Heatmap illustrating responses to the “age of onset” question format across 7 specified diseases**. Colours represent response distribution, highlighting variations in reported onset ages for each disease.

## 307 ABSTRACT P01: Effect of Inhaled Albuterol on Whole Blood Potassium and Plasma Insulin Concentrations in Cats

### 307.1 **Jared Jaffey** ^1^; Anderson da Cunha^2^; Tokiko Kushiro‐Banker^2^; Renata Costa^2^; Mark Acierno^2^; Chase Irwin^3^; Lauren Chittick^2^; Natacha Montalvo^4^; Mia Felix^2^; Megan Valentine^2^; Gurpinder Dhaliwal^2^; Jean Brudvig^5^

#### 307.1.1 ^1^Internist/Associate Professor, College of Veterinary Medicine, Midwestern University, Glendale, AZ, USA; ^2^College of Veterinary Medicine, Midwestern University, Glendale, AZ, USA; ^3^Office of Research and Sponsored Programs, Midwestern University, Glendale, AZ, USA; ^4^Office of Clinical Research Services, Midwestern University, Glendale, AZ, USA; ^5^College of Veterinary Medicine, Michigan State University, East Lansing, MI, USA

**Background:** Albuterol delivered by inhalation is a common treatment for hyperkalemia in people but its effect on blood potassium concentrations in cats is unknown.

**Objective:** Characterize the effects of inhaled albuterol on blood potassium and glucose concentrations, heart rate (HR), and plasma insulin levels in normokalemic cats.

**Animals:** Twenty healthy cats.

**Methods:** Prospective, randomized study. Cats received albuterol sulfate at a low‐dose (90 μg) or high‐dose (360 μg). Blood samples and HR were obtained at baseline and then at 10 timepoints after albuterol delivery. Blood potassium and glucose concentrations were measured via blood gas analyzer and plasma insulin levels were measured by radioimmunoassay.

**Results:** Blood potassium concentrations decreased rapidly after albuterol delivery compared to baseline for low‐dose (*p* < 0.001) and high‐dose (*p* < 0.001) within 3 min and remained significantly decreased until 180 min. The maximal decrease in potassium concentration for albuterol at the low‐dose was ‐0.52 mmol/L (95% CI: ‐0.66 to ‐0.37) and high‐dose was ‐0.38 mmol/L (95% CI: ‐0.52 to ‐0.23) and occurred at 30 min for both doses. Plasma insulin levels rapidly increased with peak effect at 30 min for both groups (low‐dose: 5.10 μIU/mL, 95% CI: 2.79‐7.40, *p* < 0.001; high‐dose: 3.86 μIU/mL, 95% CI: 1.49–6.22, *P* = 0.002). No differences in potassium, glucose, insulin, or HR were found between dosing groups.

**Conclusion and Clinical Importance:** Albuterol rapidly decreases blood potassium concentrations in part by stimulating endogenous insulin secretion in cats without dose‐dependent effects or clinically meaningful changes in HR or glucose concentrations.

## 308 ABSTRACT P02: Therapeutic Drug Monitoring of Itraconazole in Dogs With Blastomycosis

### 308.1 **Jennifer Reinhart** ^1^; Florence Leduc^2^, DVM; Andrew Hanzlicek^3^, DVM, MS, DACVIM (SAIM); Todd Marlo^2^, DVM, MS, DACVO; Audrey Billhymer^2^, DVM, DACVR; Alexandria Gochenauer^2^, PharmD, DICVP, FSVHP, FACA; Lauralei Fisher‐Cronhkite^2^, PharmD, DICVP

#### 308.1.1 ^1^Assistant Professor, SA Internal Medicine, University of Illinois Urbana‐Champaign, Champaign, IL, USA; ^2^Veterinary Clinical Medicine, University of Illinois Urbana‐Champaign, Champaign, IL, USA; ^3^MiraVista Diagnostics, Indianapolis, IN, USA

**Background:** Itraconazole is the treatment of choice for canine blastomycosis, but serum concentrations are highly variable. Therapeutic drug monitoring (TDM) could facilitate individualized therapy and optimize outcomes.

**Hypothesis/Objectives:** To establish a protocol for itraconazole TDM for canine blastomycosis.

**Animals:** 13 dogs with blastomycosis treated with itraconazole.

**Methods:** Single‐arm, prospective study. The starting dose of itraconazole was 5 mg/kg/d. TDM was performed at two weeks and then monthly until clinical and antigenic remission of blastomycosis. Itraconazole doses were adjusted to maintain trough serum concentrations within the target range (2–7 mcg/mL) using a standardized formula.

**Results:** Dogs were treated with itraconazole for a median of 7 months (range 2–10). The dose at disease remission (2.8 mg/kg/d, 0.9–5.0) was significantly lower than the initial dose (4.9 mg/kg/d, 4.0–5.4, *p* < 0.001). 11/13 dogs required at least one dose adjustment and 5/11 required three dose adjustments. The first dose adjustment occurred a median of 1 month (0.5–7) after treatment starts and occurred in the first 2 months in 10/11 dogs. 5/11 dogs underwent only dose decreases, while 6/11 required both increases and decreases. All dogs successfully achieved and maintained remission of blastomycosis. The most common adverse effects were decreased appetite (4/11) and increased ALT activity (4/11) and were mild in all cases.

**Conclusions and Clinical Importance:** Most dogs with blastomycosis require dose adjustments to maintain itraconazole concentrations in the target range. We recommend TDM be performed within 2 months of drug initiation and monitoring continued throughout treatment.

## 309 ABSTRACT P03: Comparison of Cyclosporine Pharmacokinetics and Pharmacodynamics in Blood, Plasma, Liver, and Small Intestine in Dogs

### 309.1 **Yi Jen Chang** ^1^; Todd Archer^2^, DVM, DACVIM (SAIM); Barbara Kaplan^3^, PhD; Gregory Pharr^3^, PhD; Matthew Ross^4^, PhD; Melody Whitney^5^, VMD, MS, DACVS (SA); Andrew Mackin^6^, BVMS, MVS, DVSc, FANZCVS, DACVIM (SAIM)

#### 309.1.1 ^1^PhD Student/Resident, Mississippi State University, Mississippi State, MS, USA; ^2^Bluff City Veterinary Specialists, Memphis, TN, USA; ^3^Associate Professor, Department of Comparative Biomedical Sciences, Mississippi State University, Mississippi State, MS, USA; ^4^Professor, Department of Comparative Biomedical Sciences, Mississippi State University, Mississippi State, MS, USA; ^5^Assistant Clinical Professor, Department of Clinical Science, Mississippi State University, Mississippi State, MS, USA; ^6^Professor, Department of Clinical Science, Mississippi State University, Mississippi State, MS, USA

**Background:** Cyclosporine concentrations and pharmacodynamic effects in the canine liver and intestinal tract have not been evaluated.

**Hypothesis/Objectives:** Our objective was to determine drug concentration and pharmacodynamic effects in the blood, plasma, liver, and small intestine in dogs receiving oral cyclosporine. Our hypothesis was that the drug would concentrate in liver and intestine, with associated enhanced T‐cell suppression in these locations.

**Animals:** Six healthy Walker hounds.

**Methods:** Dogs were administered oral cyclosporine 2.5 mg/kg twice daily for 7 days. Before treatment and again 2 h after the final dose, blood samples, laparoscopic liver biopsies, and endoscopic small intestinal biopsies were collected under general anesthesia. Liquid chromatography‐mass spectrometry was used to measure cyclosporine concentrations. Activated T‐cell interleukin‐2 (IL‐2) expression was measured by quantitative reverse transcription PCR. Percentage immune suppression (IS) was calculated by differences in PCR cycle threshold after administration of cyclosporine.

**Results:** Cyclosporine concentrations in liver (2537±1232 ng/mL) and intestine (5323±6697 ng/mL) were markedly higher (*p* < 0.01) than in blood (373±200 ng/mL) and plasma (138±98 ng/mL). In contrast, T‐cell IL‐2 expression was significantly more suppressed in blood (IS 51±17%) than in liver (IS 16±28%) or intestine (IS 2±39%) (*p* < 0.01). No correlation was found between cyclosporine concentration and IS between tissues (*p* = 0.724).

**Conclusions and Clinical Importance:** Surprisingly, pharmacodynamic effects in intestine and liver do not correlate with high cyclosporine concentrations. Further studies are needed to determine why high tissue concentrations do not lead to enhanced local T‐cell suppression.

## 310 ABSTRACT P04: Fluoroquinolones are a Risk Factor for Cranial Cruciate Ruptures in Retrievers

### 310.1 **Kate KuKanich** ^1^, DVM, PhD, DACVIM (SAIM); Jimena Kilian^2^; Jessica Bunch^3^; Butch KuKanich^2^; Alexa Korsberg^3^; Steve Martinez^3^; Richard Evans^4^

#### 310.1.1 ^1^Professor, Kansas State University, Manhattan, KS, USA; ^2^Kansas State University, Manhattan, KS, USA; ^3^Washington State University, Pullman, WA, USA; ^4^University of Minnesota, Minneapolis, MN, USA

**Background:** Cranial cruciate ligament rupture (CCLR) is common in retrievers, producing lameness and often requiring surgery and rehabilitation. In humans, fluoroquinolones and glucocorticoids have been associated with tendon rupture and ligament damage.

**Hypothesis:** A higher proportion of retrievers with CCLR would have previous exposure to fluoroquinolones or glucocorticoids than retrievers without CCLR.

**Animals:** Labrador and golden retrievers and retriever‐crosses, 2–12 years‐old, from Kansas State University and Washington State University with CCLR (*n* = 218, cases) and without CCLR (*n* = 203, healthy controls).

**Methods:** Retrospective, multi‐institutional case‐control study from 2019–2023. Medical records were reviewed for exposure to fluoroquinolones or glucocorticoids within 6 months of injury or visit (control) or anytime throughout life prior to injury or visit. Data were analyzed using Fisher's exact test, Wilcoxon rank sum test, and logistic regression.

**Results:** CCLR dogs (37 kg) weighed more than controls (32 kg) (*p* < 0.001). Labradors had a higher rate of CCLR (118/218) than goldens (36/218), (*p* = 0.019). Lifetime fluoroquinolone exposure was reported from 33/218 (15%) cases compared with 10/203 (4.9%) controls (*p* < 0.001). The odds of CCLR after exposure to fluoroquinolones was 3.063 times (CI 1.283–7.312) higher than the odds of CCLR without fluoroquinolone exposure. Lifetime glucocorticoid exposure was reported from 125/218 (57%) cases compared with 93/203 (46%) controls (*p* = 0.019, but not significant with multivariate analysis). No association was identified between CCLR and use of fluoroquinolones or glucocorticoids within 6 months.

**Conclusions and Clinical Importance:** These data suggest fluoroquinolones are a risk factor for CCLR in retrievers and should be used cautiously in these breeds.

## 311 ABSTRACT R01: Using Histologic Examination to Determine If Chronic Bronchitis is a Cause of Bronchomalacia in Dogs

### 311.1 **Briana Lippert** ^1^; Aida Vientós‐Plotts^2^, DVM, PhD, DACVIM (Small Animal Internal Medicine); Carol Reinero^2^, DVM, DACVIM (Small Animal Internal Medicine), PhD

#### 311.1.1 ^1^Resident Veterinarian, University of Missouri, Columbia, MO, USA; University of Missouri, Columbia, MO, USA

**Background:** The etiology of canine bronchomalacia (BM), defined as segmental and subsegmental airway collapse, is unknown. Chronic bronchitis (CB), a non‐infectious inflammatory airway disorder, is speculated to cause BM. If so, BM and CB should always be present as comorbid disorders. Using the criterion standard of histologic evaluation of lung tissue could help answer this question.

**Hypothesis/Objectives:** We hypothesized not all dogs with BM would have histologic evidence of CB.

**Animals:** Thirty client‐owned dogs with BM having lung histology.

**Methods:** Dogs with BM confirmed by bronchoscopy or paired inspiratory:expiratory breath‐hold thoracic CT scans having histologic evaluation between January 2015‐October 2024 were retrospectively evaluated. Chronic bronchitis was diagnosed by cough > 2 months, mucus hypersecretion (productive cough, visually evident luminal mucus on bronchoscopy or CT, or Curshmann's spirals on bronchoalveolar lavage (BAL) cytology), and airway neutrophilia (BAL or histology). Histology documented the presence or absence of CB and, along with the entire diagnostic picture, comorbid disorders.

**Results:** Only 7/30 dogs with BM had histologic evidence of CB. Based on clinical picture without histology, 10 dogs were diagnosed with CB, but 5/10 lacked histologic evidence of CB, having alternative explanations for BAL neutrophilia. Dogs were diagnosed on average with 5 comorbid conditions including cardiac, vascular, aerodigestive, and respiratory diseases.

**Conclusion and Clinical Importance:** Results of this study support that CB does not cause BM. Whether dogs with BM have defects in airway clearance or protection that might predispose to CB in a subpopulation of cases remains to be determined.

## 312 ABSTRACT R02: Prevalence of Tracheal Collapse (CTCS), Congenital Portosystemic Shunts (CPSS), or Both in Yorkshire Terriers

### 312.1 **Chick Weisse** ^1^; Allyson Berent^2^; SinYin Kwok^2^; Caroline Andy^3^

#### 312.1.1 ^1^Staff Surgeon/Director of IR Service, Animal Medical Center; ^2^Animal Medical Center; ^3^Weill Cornell Graduate School of Medical Sciences, New York, NY, USA

**Objective:** The incidence of tracheal collapse (CTCS) and congenital portosystemic shunts (CPSS) in Yorkshire terriers is currently not understood. CPSS may reduce the likelihood of CTCS development which would be suggested if the observed prevalence of both conditions is lower than the predicted prevalence of both conditions (based on the observed prevalence of each individual condition in a given population of dogs).

**Study Design:** Retrospective, single institutional study.

**Animals:** Yorkshire terriers with a diagnosis of CTCS, CPSS, or both seen at the Animal Medical Center (AMC) in New York City.

**Methods:** The AMC medical record system was used to recruit all available Yorkshire terriers. Data collected included patient DOB, sex, weight, date of last visit, and reported diagnoses of CTCS and/or CPSS. Medical records were reviewed to confirm diagnosis of CTCS or CPSS. Observed and predicted prevalences were compared statistically.

**Results:** 11,061 Yorkshire terriers were identified in the medical record system with age at last visit available in 7263 patients. The observed incidence of confirmed CPSS was 0.8% (93 dogs), confirmed severe CTCS was 6.7% (740 dogs), and both conditions was 0.05% (6 dogs). The median age at diagnosis of CPSS was 2.7 yrs (1.0, 5.7) and CTCS was 10.0 (8.0, 13.0). When comparing the prevalence of each condition by patient age, the observed presence of both conditions was typically lower than the predicted incidence, however, this was not significant (*p* = 0.16).

**Conclusions:** Further investigation is needed to confirm is there is a link between CPSS and the development of CTCS in Yorkshire terriers.

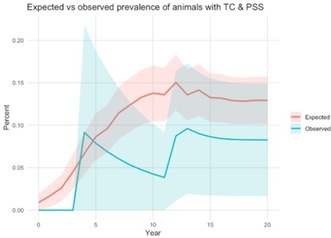

## 313 ABSTRACT R03: Unraveling Canine Infectious Respiratory Disease: Nasal Microbiome Differences in Healthy vs. Symptomatic Dogs

### 313.1 Janina Krumbeck^1^; Mark Yacoub^2^, PhD; Kaylie Zapanta^2^, PhD

#### 313.1.1 ^1^CEO and Co‐Founder, MiDOG Animal Diagnostics, Tustin, CA, USA; ^2^Microbiome Scientist, MiDOG Animal Diagnostics, Tustin, CA, USA

**Background:** The canine infectious respiratory disease complex (CIRDC) is highly contagious and challenging to treat. Initially linked to bacteria like *Mycoplasma*, *Bordetella*, and *Streptococcus* in the lung microbiome, limited research has explored the nasal microbiome. PCR studies identified *Mycoplasma canis* and *Mycoplasma cynos* as more abundant in the nasal microbiome of dogs with CIRDC. However, no studies have examined bacterial pathogens at the species level or bacterial‐fungal interactions related to CIRDC using NGS.

**Hypothesis/Objectives:** Using NGS, we analyzed the nasal bacterial and fungal microbiome in healthy and CIRDC‐affected dogs to identify contributing microbes and explore bacterial‐fungal interactions influencing CIRDC.

**Animals:** Dogs from veterinary clinics, either healthy (*n* = 63) or CIRDC‐affected (*n* = 119).

**Methods:** The nasal microbiome of each dog was examined with NGS. Linear discriminant analysis effect size (LeFSE) compared bacterial and fungal genera and species, while co‐occurrence analysis identified significant microbial interactions between bacterial and fungal species (> 1% relative abundance and > 25% frequency) in the CIRDC‐affected dogs.

**Results:** Healthy dogs had higher levels of (f) Morcellate sp., *Streptococcus canis*, and (o) Pseudomonadales sp., while CIRDC‐affected dogs had more *Staphylococcus*, *Pasteurella canis*, and *Mycoplasma cynos*. Fungal microbiome analysis showed healthy dogs had more *Aureobasidium* sp. and *Botrytis*, whereas CIRDC‐affected dogs had more Dothideomycetes, *Curvularia*, and Hypocreales spp. Co‐occurrence analysis revealed positive interactions between bacterial and fungal species (e.g., *Peptostreptococcus canis* and *Malassezia restricta*) and negative ones (e.g., *Conchiformibius steedae* and *Aspergillus* sp.).

**Conclusion and Clinical Importance:** These findings provide insights for veterinary professionals to improve CIRDC diagnosis and treatment in dogs.

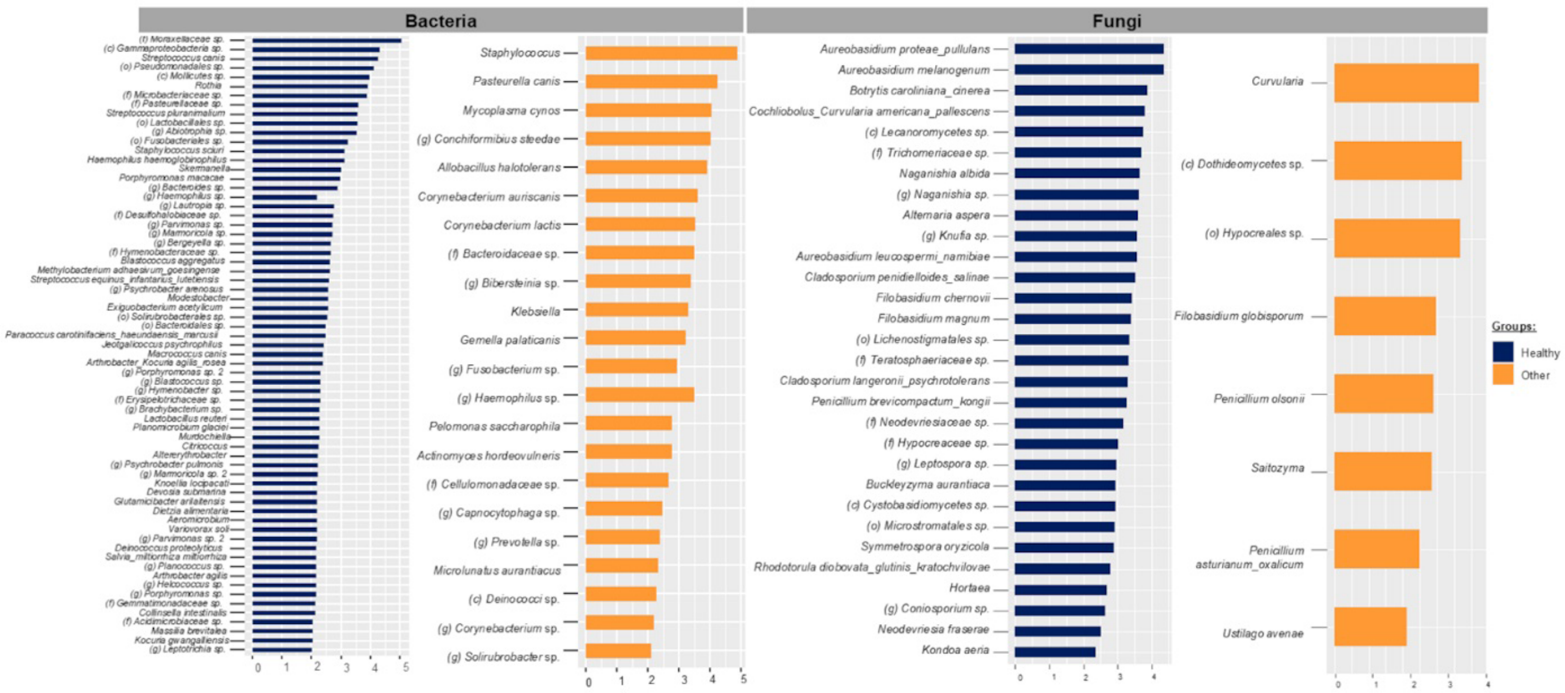

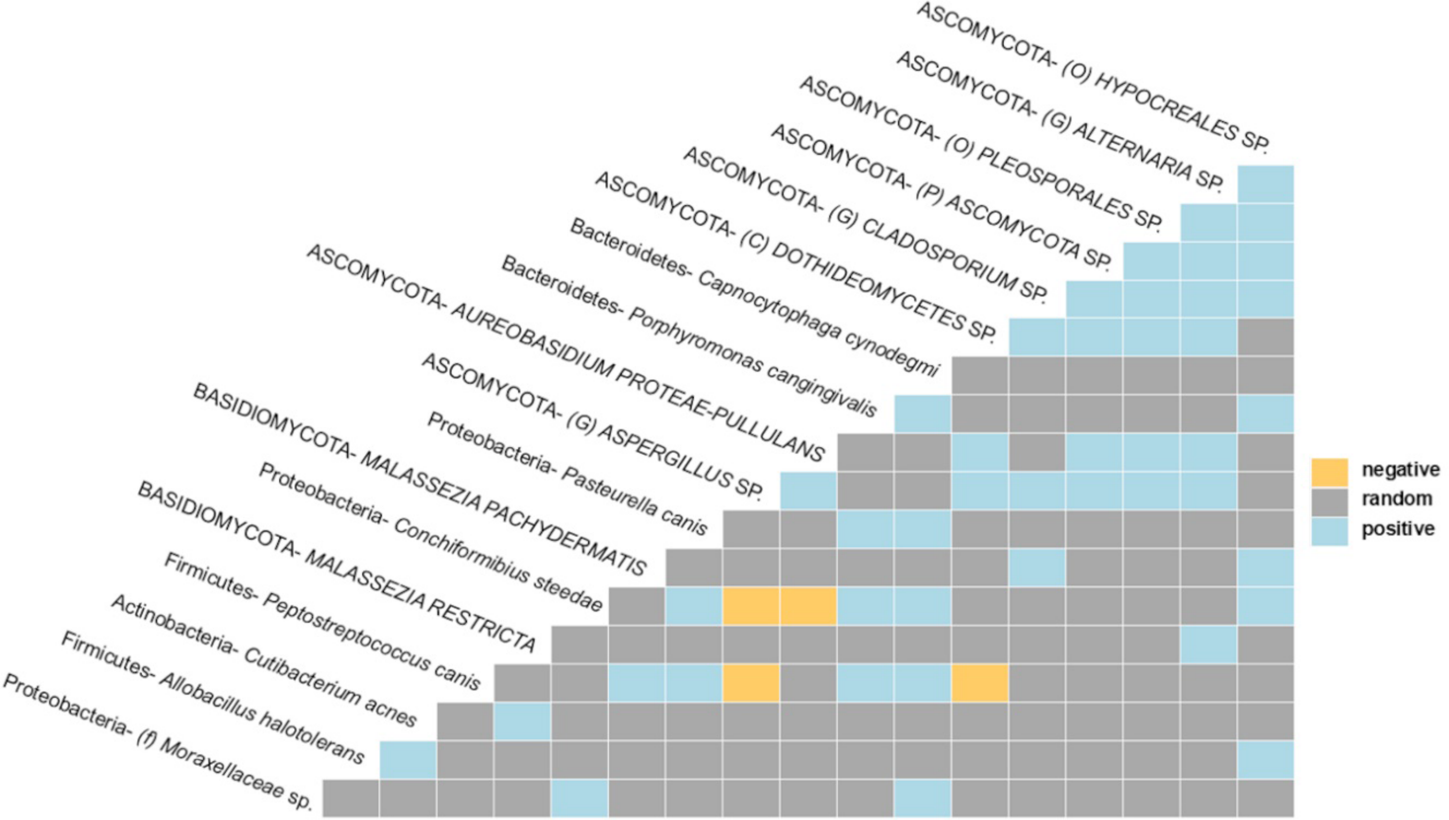

## 314 ABSTRACT R04: Effect of Chest Confirmation on Measured Expiratory Volumes and Lung Mechanics in Healthy Dogs

### 314.1 Elizabeth Rozanski^1^; Ian DeStefano^2^; Junes Ossman^3^, DVM; Noa Berlin^4^, DVM, DACVECC

#### 314.1.1 ^1^Associate Professor, Tufts University, Medford, MA, USA; ^2^Clinical Assistant Professor, Tufts University, Medford, MA, USA; ^3^Resident, Tufts University, Medford, MA, USA; ^4^Assistant Professor, Tufts University, Medford, MA, USA

**Background:** In people with acute respiratory distress syndrome (ARDS), ventilation with lower tidal volumes has been associated with improved outcome. However, chest conformation in dogs is more variable than in people and it is unclear if different chest confirmations can influence lung mechanics.

**Hypothesis/Objectives:** To prospectively evaluate lung volumes and dynamic compliance in healthy dogs with different chest conformation.

**Animals:** Healthy dogs undergoing anesthesia for treatment of a non‐pulmonary condition.

**Methods:** Dogs were classified as having a keel (e.g., setter), barrel (e.g., Labrador), or flat (e.g., bulldog) chest based upon measurement of the thoracic depth:width ratio. Dogs were anesthetized, connected to a critical care ventilator and provided pressure‐limited ventilation to a peak inspiratory pressure of 25 cm H2O to measure expiratory volume. Next, lung mechanics were measured with volume‐limited ventilation. Continuous data for each group were compared with one‐way ANOVA and Tukey *post hoc* test for multiple comparisons.

**Results:** 42 dogs were enrolled, 14 in each group. The mean expiratory volume was significantly lower in flat‐chested dogs (28.1 ±0.4 mL/kg) compared to that of keel‐chested dogs (31.5 ±5.9 mL/kg, *p* = 0.041). Flat‐chested dogs also had significantly lower dynamic compliance (1.39 ±0.23 mL/cm H2O/kg) than keel‐chested (1.50 ±0.41 mL/cm H2O/kg, *p* = 0.023) and barrel‐chested dogs (1.705 ±0.21 mL/cm H2O/kg, *p* = 0.011).

**Conclusions and Clinical Importance:** Flat‐chested dogs have a lower lung volume for a given inflation pressure and subsequently lower compliance. Chest conformation impact lung mechanics, and separate guidelines should exist for ventilatory settings.

## 315 ABSTRACT R05: Comparison of Conventional Oxygen Therapy and High‐Flow Nasal Cannula in Dogs With Non‐Cardiogenic Pulmonary Edema

### 315.1 **Hye‐In Park** ^1^; Se‐Hoon Kim^2^; Min‐Ok Ryu^2^; Kyoung‐Won Seo^2^; Woo‐Jin Song^3^

#### 315.1.1 ^1^Graduate Student, Laboratory of Internal Medicine, College of Veterinary Medicine and Research Institute for Veterinary Science, Seoul National University, Seoul, South Korea; ^2^Seoul National University, Seoul, South Korea; ^3^Jeju National University, Jeju City, South Korea

**Background:** In veterinary medicine, the use of high‐flow nasal cannula (HFNC) has been increasing, but research data on the efficacy of HFNC in treating non‐cardiogenic pulmonary edema (NCPE) remains limited.

**Hypothesis/Objectives:** To determine whether HFNC is more effective than conventional oxygen therapy (COT) in managing dogs with NCPE, based on respiratory rate (RR), peripheral capillary oxygen saturation (SpO2), dyspnea scores, and survival rate.

**Animals:** Twenty‐six client‐owned dogs diagnosed with NCPE and treated with either COT or HFNC.

**Methods:** Retrospective case series. Medical records were searched from September 2022 to September 2024. Dogs were divided into two treatment groups: COT (*n* = 13) and HFNC (*n* = 13). Changes in RR, SpO2 and dyspnea scores were obtained at the pre‐ and post‐treatment. The data gathered in this study were compared using non‐parametric tests as the Wilcoxon signed rank test and the Mann Whitney U test at a significance level of *p* < 0.05.

**Results:** Both COT and HFNC lead to improvement of RR and SpO2, but significantly higher enhancement in HFNC (*p* < 0.05). Additionally, the dyspnea scores significantly decreased only in the HFNC group (*p* < 0.01). There were no surviving patients in COT group, whereas 2 patients in HFNC group were alive at the end of the study. There were no serious adverse events associated with the use of HFNC, except for two cases of aerophagia.

**Conclusions and Clinical Importance:** HFNC can be a life‐saving tool compared to COT, resulting in improvements in respiratory parameters (RR, SpO2, and dyspnea score) in dogs with NCPE.

**Table:** **Table 1.** Dyspnea scoring system

Score	Parameter
0	Normal respiratory rate, no to mild effort
1	Respiratory rate ~40–48, able to eat/sleep/rest, no use of accessory muscles
2	Respiratory rate ~40–48 and/or: focused on respirations, mild abdominal component to breathing, occasionally will lie down, ± willing to eat
3	Respiratory rate 48–60 and/or: lips retracted, neck extension present, moderately increased respiration with abdominal component, paradoxical breathing
4	Respiratory rate > 60 with marked respiratory effort and/or: extreme restlessness, abducted elbows, unwilling to lie down despite sedation

**Table:** **Table 2.** Demographics and vital signs on the first day of hospitalization

Demographic variables	COT (*n* = 13)	HFNC (*n* = 13)	*p*
Age (years)	10 (4–16)	12 (7–18)	0.604
Weight (kg)	4.13 (2.67–12.2)	4.86 (2.33–14.4)	0.505
Body condition score	5 (4–7)	6 (3–8)	0.632
Sex			
Male intact	1	1	
Male castrated	6	8	
Female intact	1	0	
Female spayed	5	4	
Temperature (°C)	38.2 (37.0–40.2)	38.4 (37.3–39.5)	0.316
Heart rate (/min)	150 (100–174)	126 (98–180)	0.338
Respiratory rate (/min)	60 (28–150)	66 (24–120)	0.758
Systolic blood pressure (mm Hg)	140 (80–190)	140 (110–180)	0.868

* Data are presented as median (range)

COT, conventional oxygen therapy

HFNC, high‐flow nasal cannula

**Table:** **Table 3.** Concurrent diseases with non‐cardiogenic pulmonary edema

Concurrent disease	COT (*n* = 13)	HFNC (*n* = 13)	Total (*n* = 26)
Malignancy	3	3	6 (23.1%)
Pancreatitis	2	3	5 (19.2%)
Cushing's disease	3	2	5 (19.2%)
Acute on CKD	3	1	4 (15.4%)
Seizure	2	2	4 (15.4%)
Upper respiratory tract obstruction	1	2	3 (11.5%)
SIRS	0	2	2 (7.7%)
AKI	0	1	1 (3.8%)

AKI, Acute kidney injury

CKD, Chronic kidney disease

SIRS, Systemic inflammatory response syndrome

**Figure:**
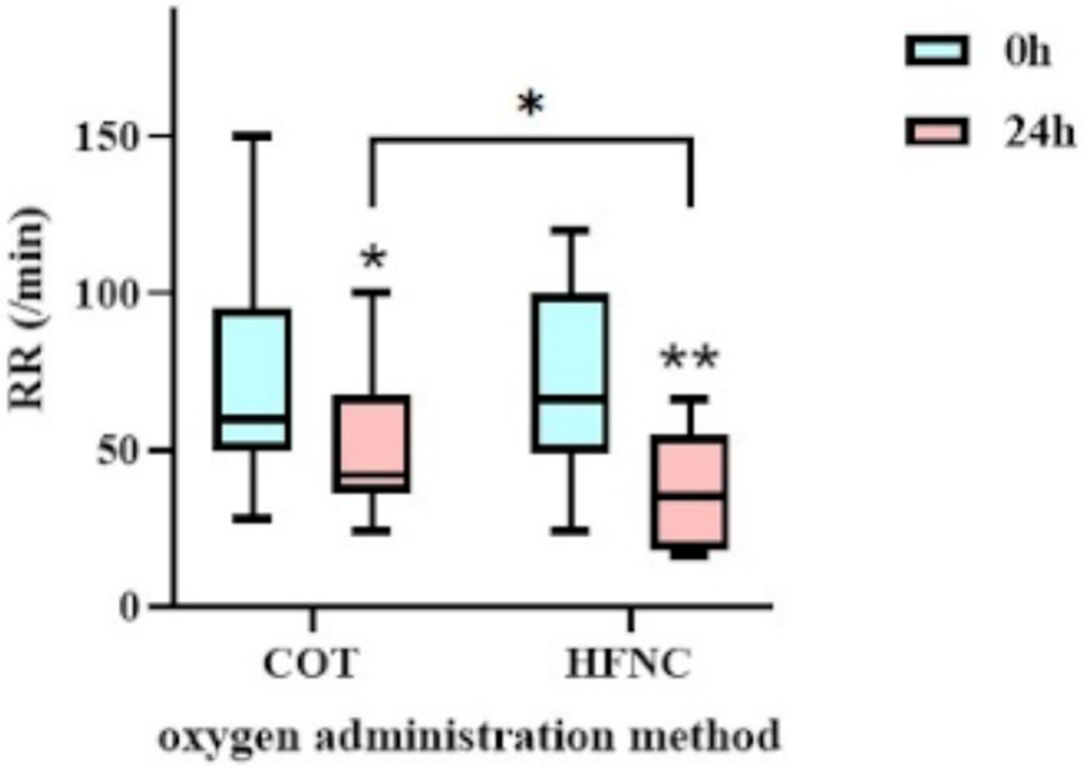
**Figure 1. Box‐and‐whisker plot of RR for dogs treated with COT (left) and HFNC (right)**. Each box represents the second and third quartiles, the whiskers indicate the range, and the bar within each box represents the median. The plots show a statistically significant decrease in RR in the HFNC group compared to that in the COT group. *p < 0.05, **p < 0.01. COT, conventional oxygen therapy. HFNC, high‐flow nasal cannula. RR, respiratory rate

**Figure:**
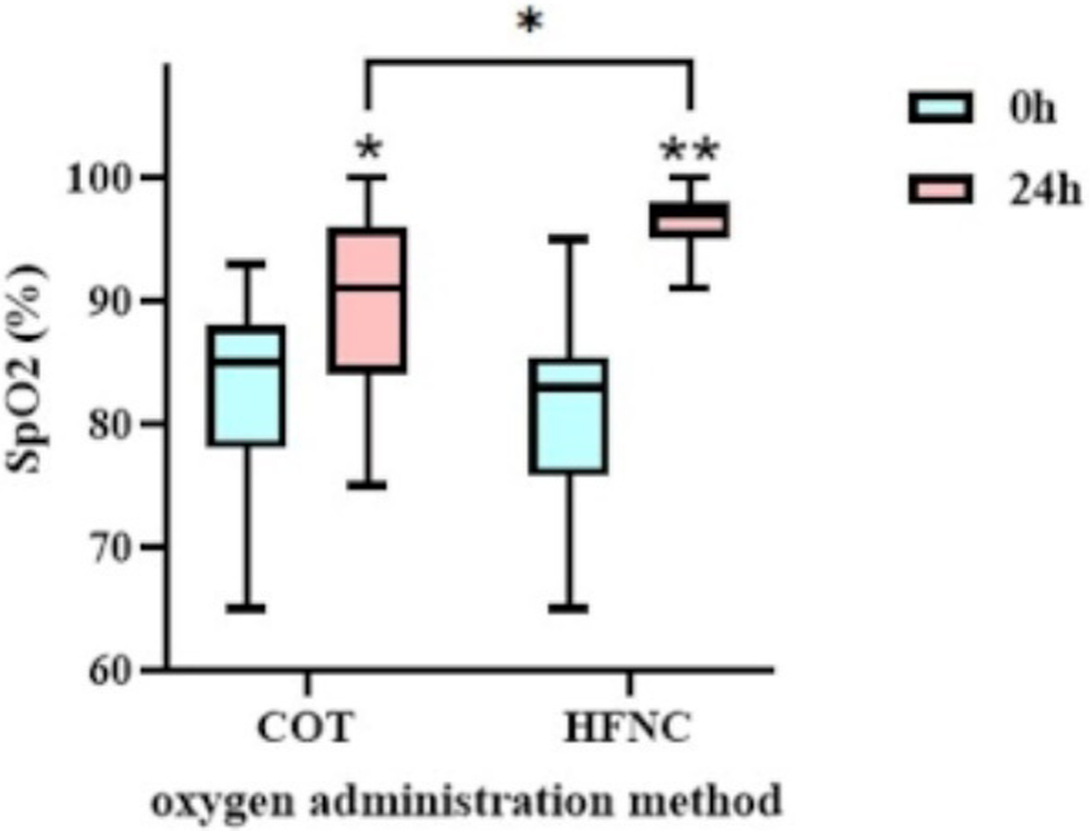
**Figure 2. Box‐and‐whisker plot of SpO2 for dogs treated with COT (left) and HFNC (right)**. Each box represents the second and third quartiles, the whiskers indicate the range, and the bar within each box represents the median. The plots show a statistically significant increase in SpO2 in the HFNC group compared to that in the COT group. *p < 0.05, **p < 0.01. COT, conventional oxygen therapy. HFNC, high‐flow nasal cannula. SpO2, peripheral capillary oxygen saturation

**Figure:**
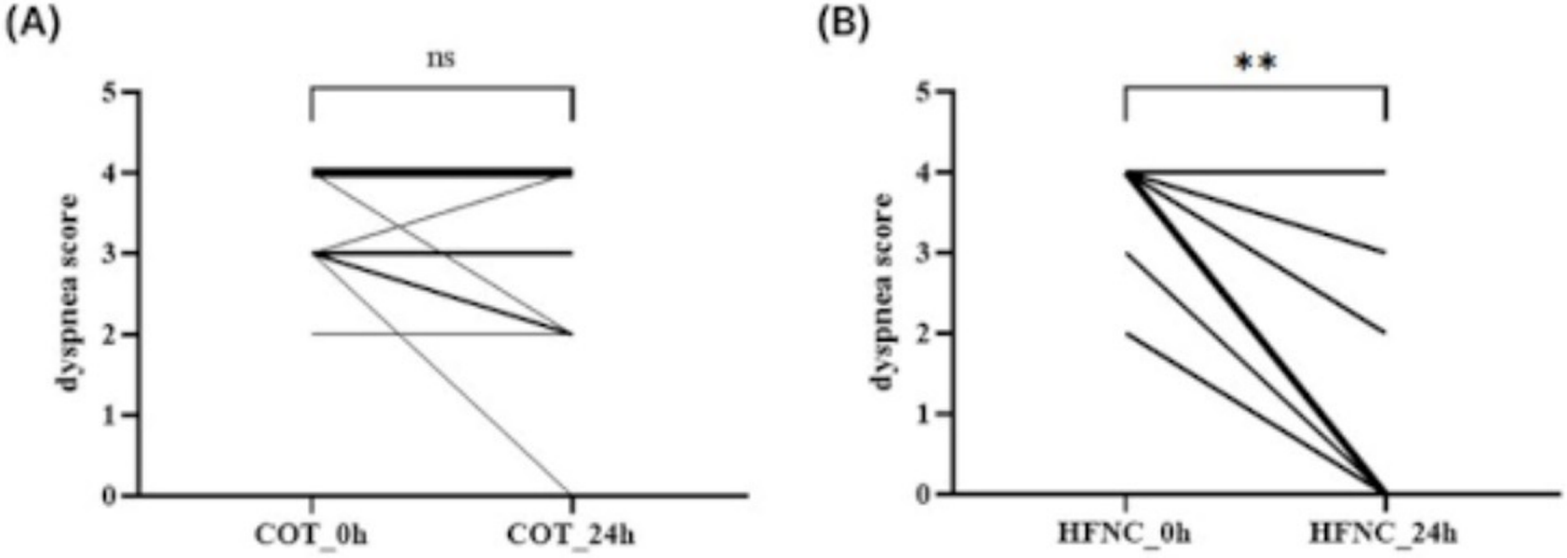
**Figure 3. Paired line plots showing changes in dyspnea scores at 0 h and 24 h for dogs treated with COT (A) and HFNC (B)**. (A) The thickest line (dyspnea score remaining at 4) represents 5 dogs, whereas the moderately thick lines (dyspnea score changing from 3 to 3, and from 3 to 2) each represent 2 dogs. All other thinner lines each represent 1 dog. (B) The thick line (dyspnea score changing from 4 to 0) represents 3 dogs, whereas all other lines each represent 2 dogs. The plots show a statistically significant reduction in dyspnea scores in the HFNC group, whereas the COT group did not exhibit a significant reduction. ns = non‐significant, *p < 0.05, **p < 0.01. COT, conventional oxygen therapy. HFNC, high‐flow nasal cannula

**Figure:**
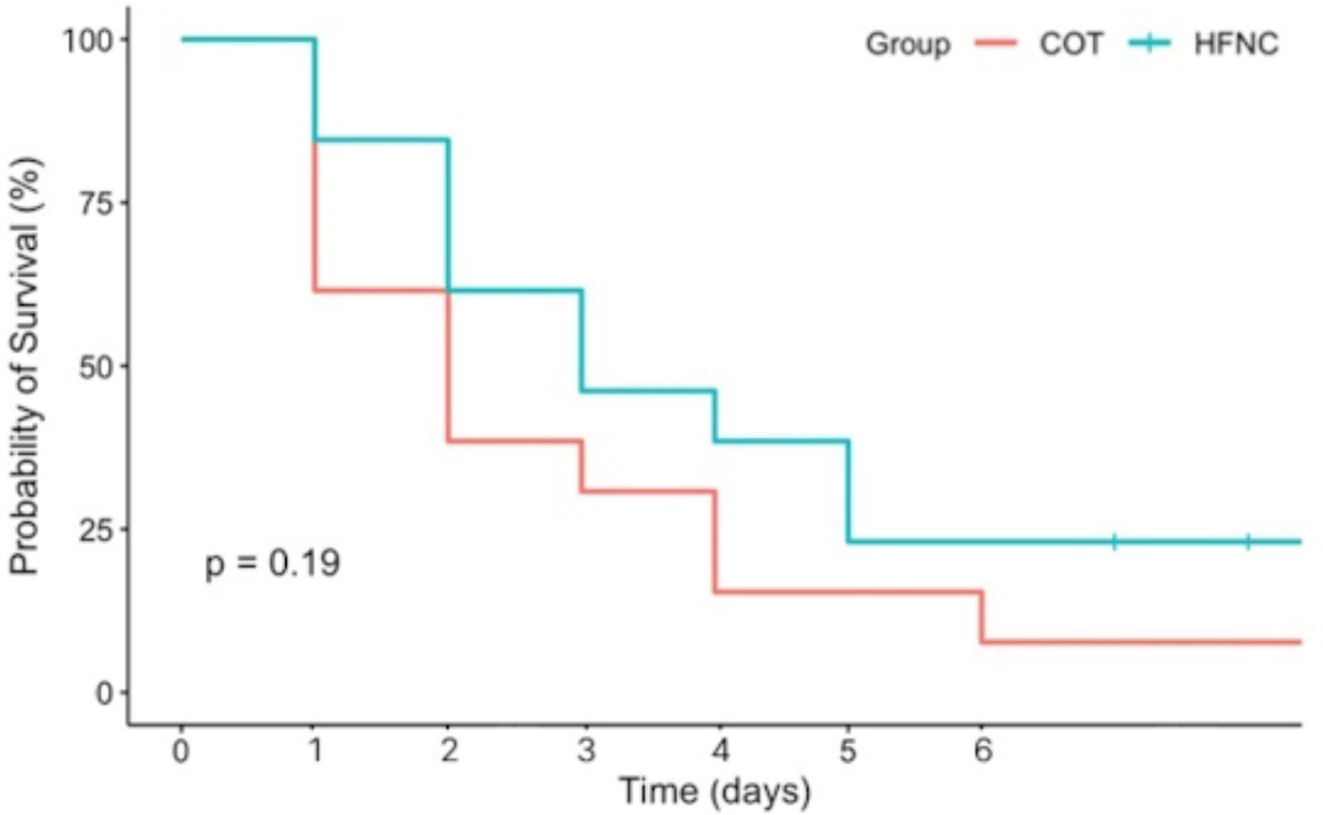
**Figure 4. Kaplan–Meier survival curves comparing the survival probabilities of dogs treated with COT and HFNC**. The x‐axis represents time (in days), and the y‐axis represents survival probability. The log‐rank test revealed no statistically significant difference (*p* = 0.19) in survival between the two groups. Censored data points are indicated by vertical ticks on the HFNC curve. COT, conventional oxygen therapy. HFNC, high‐flow nasal cannula

## 316 ABSTRACT R06: Soft Palate Defects as a Cause of Chronic Nasal Signs in 3 Cats

### 316.1 **Jennifer Weng** ^1^, DVM, BS; Catriona MacPhail^2^, DVM, PhD, DACVS; Michael Lappin^3^, DVM, PhD, DACVIM (SAIM)

#### 316.1.1 ^1^Small Animal Internal Medicine Resident, Colorado State University, Fort Collins, CO, USA; ^2^Professor, Small Animal Surgery, General Surgery, Veterinary Teaching Hospital, Colorado State University, Fort Collins, CO, USA; ^3^Professor, Clinical Sciences, Small Animal Internal Medicine, Veterinary Teaching Hospital, Colorado State University, Fort Collins, CO, USA

**Background:** Chronic nasal discharges are common in cats and when the character of the nasal discharges is mucopurulent in appearance, infectious etiologies are often suspected. Frequently, antibiotics or anti‐viral therapies are prescribed empirically without a complete diagnostic workup.

**Hypothesis/Objectives:** To describe 3 cases of chronic nasal signs that resulted from unusual defects of the soft palate in otherwise healthy cats.

**Animals:** Three client‐owned cats with chronic nasal signs.

**Methods:** Case series. Clinical and medical records evaluation of the 3 clinical cases.

**Results:** Three young cats (age, 4 months to 3 years) were initially suspected of having community acquired bacterial or viral infection leading to mucopurulent nasal discharges and intermittent sneezing. Several antibiotics and anti‐viral therapies failed to control clinical signs, so referral to a veterinary speciality hospital was recommended. No abnormalities were noted on awake oral examinations but after induction of anesthesia, defects within the soft palate (5×5 mm penetrating defect through the soft palate; 5 cm penetrating defect through the soft palate; 1.5 cm congenital midline cleft palate extending the entire soft palate) were appreciated. Surgical repair resulted in significant improvement to resolution of clinical signs.

**Conclusions and Clinical Importance:** These 3 cases emphasize that a sedated oropharyngeal examination should be performed early in the clinical evaluation of young cats to detect palatal and other abnormalities before attempting multiple antibiotic or antiviral trials for the treatment of nasal signs.

## 317 ABSTRACT SC01: ENaC Blockade Enhances Circadian Variation in Systolic Blood Pressure Independent of Diabetic Status

### 317.1 **Geoff Culshaw** ^1^; Kevin Stewart^2^, BSc (Hons); Natalie Jones^3^, BSc (Hons) MScR PhD; Matthew Bailey^4^, BSc (Hons) PhD

#### 317.1.1 ^1^Professor in Small Animal Cardiology, University of Edinburgh, Edinburgh, Scotland; ^2^ *In Vivo* Laboratory Manager, Centre for Cardiovascular Science, Queen's Medical Research Institute, Edinburgh, Scotland; ^3^U‐Care Project Manager, Centre for Inflammation Research, Queen's Medical Research Institute, Edinburgh, Scotland; ^4^Professor, Centre for Cardiovascular Science, Queen's Medical Research Institute, Edinburgh, Scotland

**Background:** Deranged glucose metabolism has deleterious cardiovascular and renal consequencies, predicted by disrupted circadian variation in blood pressure (BP). However, SGLT2 inhibitors (SGLTi), now veterinary‐licensed, reduce cardiovascular risk in people and are renoprotective. The mechanism is unknown. We modelled natriuresis and glucosuria from SGLTi and assessed cardiovascular risk in rodents.

**Hypothesis:** Combining natriuresis with glucosuria enhances circadian variation in BP.

**Animals:** Sprague Dawley rats (*n* = 16, 250–300 g) were maintained on standard chow and water *ad libitum* (12‐h light cycle, 21±1°C, 50% humidity). Diabetes mellitus (T1DM) was induced (35 mg/kg streptozotocin, intraperitoneal) or rats received vehicle (*n* = 8 both groups).

**Methods:** BP was measured continuously for 2 weeks with radiotelemetry devices (DSI‐HD‐S10, distal aorta). After baseline (week 1), rats ate oral amiloride (ENaC antagonist, 1 mg/kg q24) in week 2. Data (mesor±SD) were assessed by cosinor analysis and 2‐way repeated‐measures ANOVA.

**Results:** There were complete data from 5 controls and 6 T1DM rats. Sinusoidal variation in systolic BP (127±8 mm Hg) and heart rate occurred in all rats. Amiloride did not modify systolic mesor but increased amplitude and dip by ~25% to 5.5±1.2 mm Hg and 4.7%±0.5%. Diastolic BP (87±3 mm Hg) was unaffected. T1DM reduced heart rate by ~40 beats/min to 340.8±34.2 beats/min, while amiloride reduced it in controls and T1DM rats by ~20 beats/min without changing amplitude or dip (40.3±7.4 beats/min, 11.2%±1.9%).

**Conclusions and Clinical Importance:** Natriuresis by ENaC blockade reduced heart rate and enhanced circadian variation in BP, regardless of diabetic status. Contrary to our hypothesis, natriuresis from SGLT2i, rather than natriuresis/glucosuria combined, may offer cardiovascular benefits in small animals.

## 318 ABSTRACT SC02: First Step Toward Precision Veterinary Clinical Decision‐Support Development Incorporating Physiological and Electronic Health Records

### 318.1 **Manlik Kwong** ^1^, BSEE, BSCS; Thomas Jacob^2^, MBA, MS; Micah Hernandez^3^, BSEE; Michael Jacob^4^, MS; Vicky Yang^5^, DVM, PhD, DACVIM; Hannah Melhorn^6^, DVM

#### 318.1.1 ^1^Scientist/Engineer, Senior Informatics Advisor, Research Instructor, Tufts Medical Center, Tufts CTSI, Tufts School of Medicine; ^2^CEO, Dextronix, Inc.; ^3^Systems Engineer, Dextronix, Inc.; ^4^Software Engineer, Dextronix, Inc.; ^5^Associate Professor, Department of Clinical Sciences, Cummings School of Veterinary Medicine, Tufts University; ^6^Cardiology Resident, Foster Hospital for Small Animals, Cummings School of Veterinary Medicine, Tufts University

Interest and work in using machine learning and artificial intelligence (ML/AI) to develop and improve clinical decision‐making is an active area within human medicine. Examples in human medicine include clinical decision‐support in intensive care unit (ICU) sepsis infections [1] to computerized interpretation of electrocardiographic conditions such as left ventricular dysfunction [2] and arrhythmias detection using deep learning methods. [3] These approaches and methods can be translated to veterinary medicine to improve pre‐surgery assessment for fitness to reduce unnecessary death and adverse outcome or arrhythmia diagnosis to initiate appropriate management strategies. [4] Real‐time monitoring and assessment of physiological signals including multi‐lead electrocardiographic signals during interventions such as surgery could provide early indicators of emergent adverse events. To enable the development of new veterinary clinical decision support tools and methods to facilitate and support complex ECG interpretation across the care spectrum, high fidelity data must be acquired. Further such data must be reviewed and annotated and linked to medical histories and outcomes to form reference datasets from which clinical decision aids can be derived and dissemination issues identified to translate such tools from bench to bedside. The standardization of electronic health record data is now becoming readily available (e.g., Observational Health Data Science and Informatics’ Observational Medical Outcomes Partnership—OHDSI OMOP common data model) and the integration of high fidelity physiological data will further expand the capacity of research and discovery.

**Figure:**
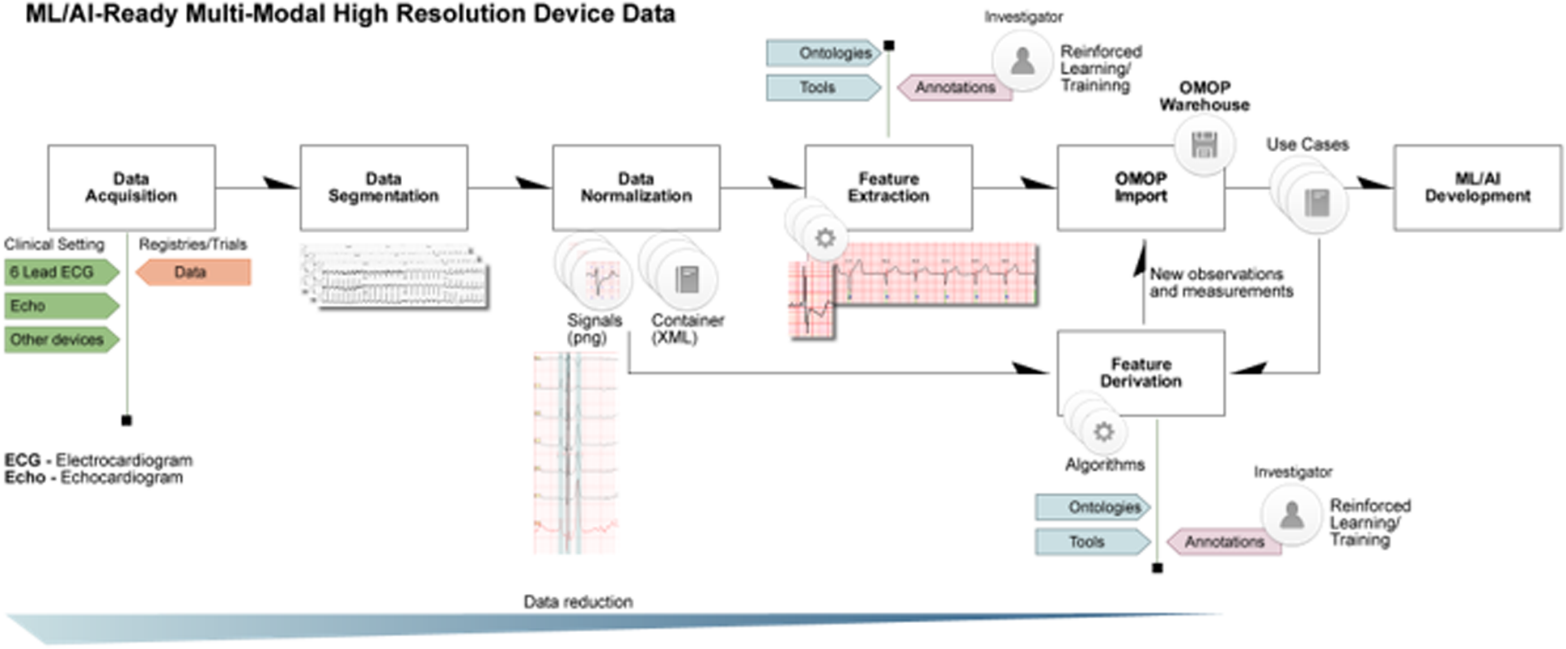
Figure 1. Integration of physiological data into an OMOP research warehouse

## 319 ABSTRACT SC03: CD40‐CD40 Ligand‐Mediated Cardiorenal Injury in Canine Myxomatous Mitral Valve Disease

### 319.1 **Seung‐Bum Cho** ^1^, DVM; Jungwoo Han^2^; Suyeon Kim^2^; Geunwoo Jeon^2^; Minseok Kim^2^; Yu‐Tae Jang^2^; Hee‐Woo Cha^2^; Ah‐Yeon Jeong^2^; Ha‐Jung Kim^2^

#### 319.1.1 ^1^Student, Chonnam National University; ^2^Chonnam National University

**Background:** CD40‐CD40 ligand (CD40L) interaction, one of the inflammatory mechanisms, is known to play a pathological role in heart failure in human medicine. However, its role in dogs with myxomatous mitral valve disease (MMVD) remains unexplored.

**Hypothesis:** CD40‐CD40L interaction may play a role in myocardial and renal tubular injury in dogs with MMVD.

**Animals:** Twenty client‐owned healthy dogs and forty client‐owned dogs with MMVD, categorized into B1, B2, C, and D groups according to the American College of Veterinary Internal Medicine guidelines.

**Methods:** A prospective study with cross‐sectional and longitudinal analyses. Serum soluble (s) CD40L, N‐terminal pro‐B‐type natriuretic peptide (NT‐proBNP), and urinary neutrophil gelatinase‐associated lipocalin/creatinine ratio (uNGALCR) were measured. Longitudinal analysis in MMVD C and D groups compared values at baseline (day 0) and during medical therapy at 6 weeks and 3 weeks, respectively.

**Results:** Serum NT‐proBNP (*P* = 0.0001), sCD40L (*P* < 0.0001), and uNGALCR (*P* = 0.0004) were significantly elevated in dogs with MMVD. Medical treatment significantly reduced serum sCD40L levels in the stage C group (*P* = 0.0001). Serum sCD40L positively correlated with NT‐proBNP (*r* = 0.2784, *P* = 0.014) and uNGALCR (*r* = 0.3025, *P* = 0.039).

**Conclusions and Clinical Importance:** Serum sCD40L levels are elevated in dogs with MMVD and positively correlate with biomarkers of cardiac and renal tubular injury. These findings suggest that enhanced CD40‐CD40L interactions contribute to the pathogenesis of canine MMVD.

**Figure:**
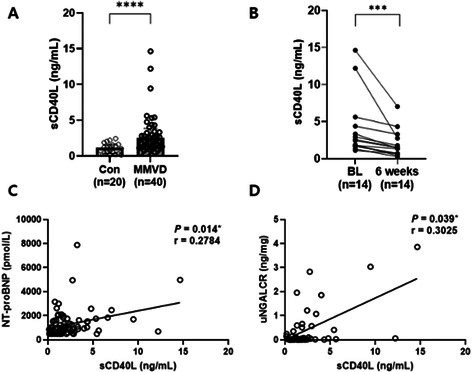
**Figure 1** (A) Bar plots of serum sCD40L levels comparing healthy controls (Con, *n* = 20) and MMVD patients (*n* = 40). The bars represent the median values, and the error bars represent the interquartile ranges (25th–75th percentile). Individual data points are shown for each group. (B) Paired dot plots showing serum sCD40L levels at baseline (BL) and while receiving treatment in dogs with MMVD stages C (*n* = 14). (C, D) Correlation of sCD40L with NT‐proBNP (C), UNCR (D) in healthy controls and MMVD patients. Scatter plots show the correlation between sCD40L levels and the corresponding variables. Solid line represents a trendline of the data *P < 0.05, **P < 0.01, ***P < 0.001, and ****P < 0.0001. sCD40L, soluble CD40 ligand; NT‐proBNP, N‐terminal pro‐B‐type natriuretic peptide; MMVD, myxomatous mitral valve disease; uNGALCR, urinary neutrophil gelatinase‐associated lipocalin/creatinine ratio.

**Figure:**
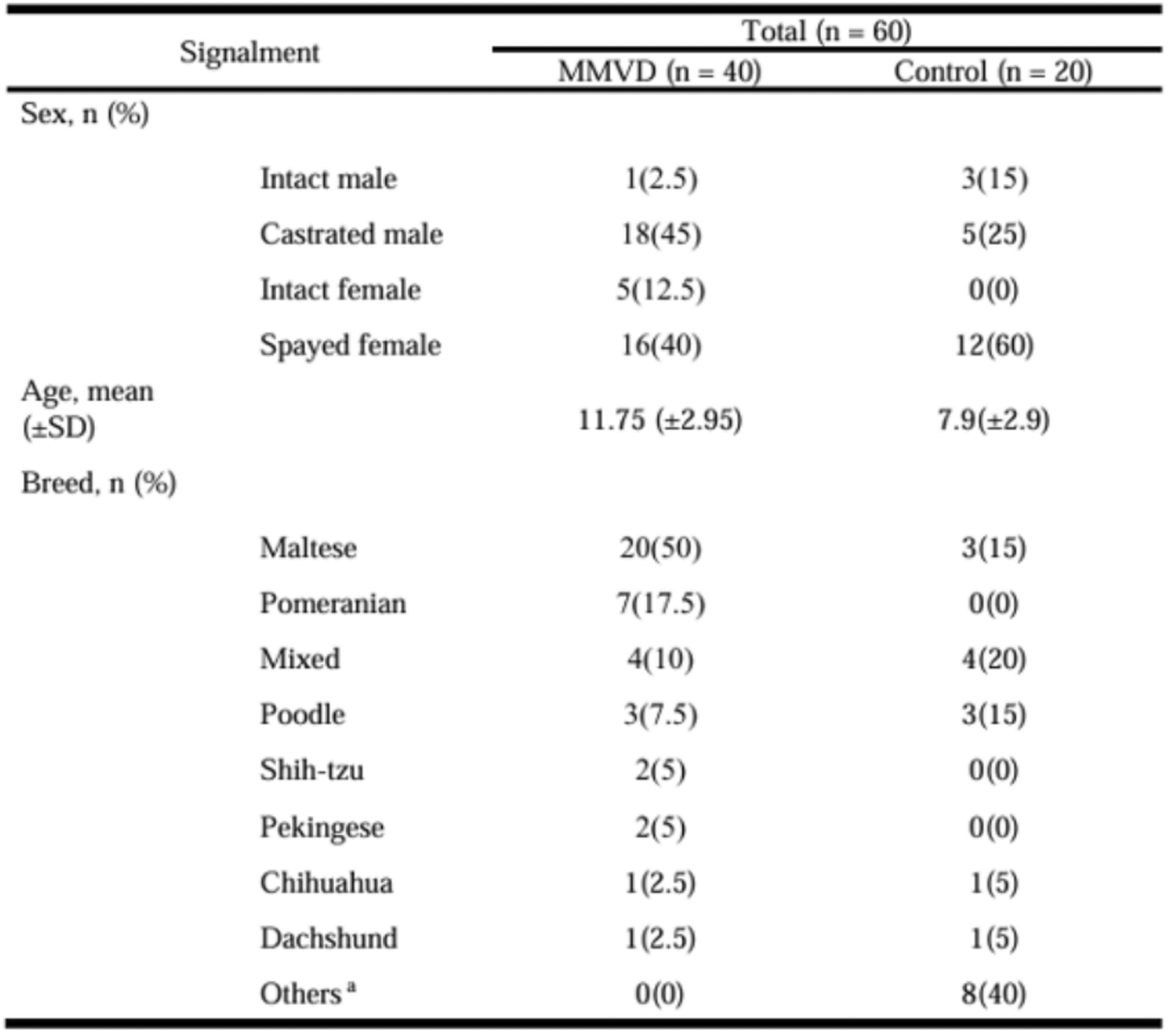
**Table 1. Epidemiological characteristics of total participants**. a. Others: beagle, bichon frise, cocker spaniel, Jindo, Shiba Inu, spitz, Yorkshire terrier

## 320 ABSTRACT SC04: Impact of Pulmonary Hypertension for Hepatobiliary System: Retrospective Study in 33 Dogs

### 320.1 **Jae Hun Kim** ^1^, DVM, MS; Min Suk Kim^2^, DVM; Chul Park^3^

#### 320.1.1 ^1^PhD Candidate, Jeonbuk National University Animal Hospital; ^2^Small Animal Internal Medicine, Jeonbuk National Animal Hospital; ^3^Professor, Small Animal Internal Medicine, Jeonbuk National Animal Hospital

**Background:** According to the American College of Veterinary Internal Medicine, pulmonary hypertension (PH) in dogs is defined by increased pulmonary arterial pressure, which can result in venous congestion and affect the hepatic vein. Understanding the relationship between PH and the hepatobiliary system is critical for disease management.

**Hypothesis/Objectives:** This study hypothesizes that dogs with PH exhibit significant alterations in liver enzyme levels. The objective is to investigate cardio‐hepatic relationship in dogs with PH.

**Animals:** 64 dogs were included in this retrospective study: 33 dogs with PH diagnosed via echocardiographic criteria and 31 healthy controls with no clinical abnormalities.

**Methods:** Clinical data were collected from physical examinations, blood analyses, radiography, and echocardiography. Liver enzyme levels (ALT, ALP, AST, GGT, and total bilirubin) were analyzed and compared between groups. Statistical analyses included t‐tests, Mann‐Whitney U tests, and Spearman's rank correlation to assess relationships between liver enzyme levels and cardiac indices such as pulmonary velocity (PV vel), pulmonary arterial‐to‐aortic diameter (PA/Ao), tricuspid regurgitation velocity.

**Results:** Dogs with PH had higher ALT and ALP levels compared to controls (median [IQR][95% CI]: 102.5 [49–157] [85.15–137.14], 214 [128.5–505.75][231.12–557.58], respectively; *p* < 0.05). ALT correlated with PV vel (correlation coefficient=0.345, *p* = 0.008) and PA/Ao (correlation coefficient=0.276, *p* = 0.028). ALP correlated with PA/Ao (correlation coefficient=0.399, *p* = 0.001).

**Conclusions and Clinical Importance:** These findings suggest that PH may affect the hepatobiliary system. Regular monitoring of liver enzymes in dogs with PH could facilitate early detection and management of systemic complications.

**Figure:**
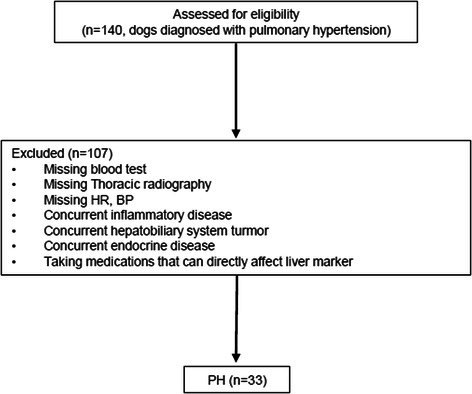
Figure 1. Flow diagram describing case selection

## 321 ABSTRACT SNM01: Temporal Variability of the Canine Fecal Microbiome

### 321.1 **Connie Rojas** ^1^; Holly Ganz^2^, PhD; Becky Smith^3^, LVMT, VTS (ECC)

#### 321.1.1 ^1^Senior Scientist, AnimalBiome, Oakland, CA, USA; ^2^Chief Science Officer, R&D, AnimalBiome, Oakland, CA, USA; ^3^Veterinary Care Specialist, AnimalBiome Veterinary, AnimalBiome, Oakland, CA, USA

**Background:** To date, no studies have evaluated the stability of the fecal microbiome of healthy dogs over six months. Knowledge of microbiome dynamics and stability could enhance its use as a diagnostic and therapeutic tool.

**Hypothesis/Objectives:** Determine the degree of temporal variability observed in canine fecal microbiomes and identify the bacterial species that are the most dynamic over time.

**Animals:** Thirty‐five healthy pet dogs with no history of clinical signs, physical conditions, or medication use were sampled longitudinally over a median of 1.07 years (0.3–2.64 years), with samples collected every 30 days.

**Methods:** Full‐length PacBio 16S rRNA gene sequence data from fecal samples were analyzed in R. Microbiome variability was assessed using sample distance to centroid, which captures how much a dog's microbiome deviates from its compositional center. Temporal variability of 28 core bacterial species was quantified using coefficients of variation.

**Results:** Fecal microbiome composition was highly variable over time in the surveyed dogs and also showed individual‐specific signatures. All 28 bacterial species exhibited substantial temporal fluctuations (median CV: 80–323%). *Megamonas funiformis*, *Prevotella copri*, *Peptacetobacter hiranonis*, *Blautia marasmi*, and *Collinsella intestinalis* were the most stable, while *Escherichia coli*, *Streptococcus gallolyticus*, *Enterococcus faecium*, and *Enterococcus hirae* were the most dynamic.

**Conclusions:** The fecal microbiomes of healthy dogs vary considerably over time, indicating that temporal variability is an inherent characteristic of canine microbial communities. These findings highlight the need for longitudinal sampling in microbiome studies, particularly for therapeutic interventions.

## 322 ABSTRACT SNM02: Defining the Core Microbiome in Healthy Cats

### 322.1 **Connie Rojas** ^1^; Holly Ganz^2^, PhD; Brian Park^3^, PhD

#### 322.1.1 ^1^Senior Scientist, AnimalBiome, Oakland, CA, USA; ^2^Chief Science Officer, R&D, AnimalBiome, Oakland, CA, USA; ^3^Bioinformatician, R&D, AnimalBiome, Oakland, CA, USA

**Background:** A core microbiome at the species level has been defined for healthy dogs but is lacking for healthy cats. Knowledge of the exact bacterial species associated with health is imperative for restoring dysbiotic microbiomes.

**Hypothesis/Objectives:** Identify the bacterial species that constitute the core microbiome in healthy cats and examine the effects of age, sex, body weight, and diet on this core.

**Animals:** Fecal samples were collected from 104 healthy cats living in homes with no current or past physical conditions, no clinical signs, and no reported medication use. In this reference population, 20% were verified healthy via veterinary records and monthly parasite and pathogen testing.

**Methods:** Full‐length PacBio16S rRNA gene sequence data from fecal samples were analyzed in R. Core bacterial species were present in at least 38% of cats. Generalized linear models were used to correlate two core microbiome metrics with host factors. PERMANOVAs identified significant factors driving microbiome beta‐diversity.

**Results:** 22 bacterial species formed part of the core microbiome in healthy cats, and among the most prevalent were: *Blautia hansenii*, *Collinsella intestinalis*, *Collinsella tanakaei*, *Clostridium hiranonis*, unclassified Lachnospiraceae, and *Clostridium perfringens*. Neither the total number of core taxa, or proportion of microbiome composed by core taxa differed between cats of different ages, sexes, diets, or body weights. However, diet category (kibble vs. raw vs. canned) and age were significant drivers of microbiome beta‐diversity.

**Conclusions:** A common core group of 22 bacterial species were identified, and fecal microbiome profiles varied with diet and age.

## 323 ABSTRACT SNM03: Establishing a Reference Interval for Postprandial Triglyceride Concentrations in Healthy Adult Dogs

### 323.1 **Deborah Linder** ^1^, DVM, MS, DACVIM (Nutrition); Rachel Hanford^2^, DVM; Jenn Hergert^1^; Sara Huebner^1^; Kady Marino^1^; Kristen Thane^1^; Leslie Sharkey^1^; Claire Fellman^1^

#### 323.1.1 ^1^Tufts University; ^2^Medical Oncology Resident, University of California–Davis

**Background:** Current recommendations for dogs suggest measuring triglyceride concentrations after a minimum 12‐h fast. However, reference values for postprandial triglyceride concentrations in healthy adult dogs receiving a non‐standardized diet remain unknown.

**Hypothesis/Objectives:** To establish a reference interval for postprandial triglyceride concentrations in healthy dogs fed non‐standardized diets and investigate the effect of body condition score (BCS) on serum triglyceride concentrations.

**Animals:** 57 healthy, client‐owned dogs were enrolled. Breeds known to have dyslipidemias and dogs receiving medications and supplements known to affect lipid metabolism were excluded.

**Methods:** Cohort study. Triglyceride concentrations were measured after a minimum 12‐h fast (T0) and repeated four (T4) and six (T6) h after the dog's typical meal. Reference intervals were calculated for all dogs and separately for ideal (BCS 4–5), overweight (BCS 6–7), and obese (BCS 8–9) dogs using nonparametric methods. For groups with less than 20 individuals, the range of triglyceride concentrations are presented. Multivariable linear regression using maximum triglyceride concentration at 4 or 6 h (MaxTg) as the outcome was performed.

**Results:** The median (reference interval) triglyceride concentrations at times T0, T4, and T6 are summarized in Table 1. Patient BCS was significantly associated with MaxTg, adjusting for T0 triglyceride concentration. Subjects with overweight or obese BCS exhibited MaxTg values that were on average 38 mg/dL higher than subjects with ideal BCS (*p* = 0.039).

**Conclusions and Clinical Importance:** The proposed triglyceride reference interval for postprandial dogs will improve recognition of lipid abnormalities and increase the diagnostic yield of non‐fasted bloodwork.

**Figure:**
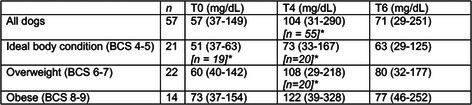
**Table 1. Serum triglyceride concentrations expressed as median (reference interval)**. Serum triglyceride concentration reference intervals for healthy dogs fed a non‐standardized diet calculated using nonparametric methods or expressed as the range for group sizes less than 20. BCS=body condition score; T0=triglyceride concentration after a minimum 12‐h fast; T4=triglyceride concentration 4 h after feeding; T6=triglyceride concentration 6 h after feeding. *n accounting for missing data and/or after excluding outliers.

## 324 ABSTRACT SNM04: Case Description of Canine Gallbladder Mucocele: Retrospective Study of 60 Cases

### 324.1 **Monique Paludetti** ^1^, DVM; Crislauana Garcia^2^, DVM; Alessandra Vargas^3^, MSc; Fabio Teixeira^4^, PhD

#### 324.1.1 ^1^Doctor of Veterinary Medicine Student, School of Veterinary Medicine and Animal Science, University of São Paulo, São Paulo, São Paulo, Brazil; ^2^National Association of São Paulo for Small Animal Practice (ANCLIVEPA‐SP); ^3^EndocrinoVet—Veterinary Endocrinology and Metabolism; ^4^School of Veterinary Medicine and Animal Science, University of São Paulo

**Background:** Limited information exists regarding the pathogenesis and treatment of gallbladder mucocele (GBM) in dogs.

**Objective:** To analyze epidemiological data and evaluate remission possibilities in dogs diagnosed with GBM.

**Animals:** Dogs treated at an endocrinology service (October/2017–May/2023).

**Methods:** A retrospective descriptive analysis of medical records from dogs diagnosed with GBM.

**Results:** Out of 945 dogs examined, 63 were diagnosed with GBM (three cases excluded due to missing data). Among the remaining 60 cases, 65% were female, with an average diagnosis age of 12.3 years. Mixed breed dogs (30%), Lhasa apso (13.3%), and shih tzu (13.3%) were most commonly affected. GBM occurred without comorbidities in 35% of cases. Comorbidities included hypercortisolism (28.3%), diabetes mellitus (8.3%), hypothyroidism (5.0%), adrenal neoplasia (10.0%), obesity (3.3%), and other conditions (9.8%). Cholecystectomy was performed in 29 dogs (62% elective, 34.5% emergency), with no bacterial growth in 70% of cases and one postoperative death. Conservative treatment approach was used in 31 cases. One dog received only bezafibrate, while the others were treated with varying combinations of fish oil, ezetimibe, S‐adenosylmethionine (SAMe), silymarin, ursodeoxycholic acid, and hepatoprotectors. Resolution occurred in 25.8% (8/31), with two recurrences at 5 and 36 months. Six dogs achieved complete remission (three with GBM alone, one with diabetes, one with hypercortisolism, and one with adrenal neoplasia) following 3 to 11 months of conservative treatment.

**Conclusion:** Although surgical treatment remains the definitive approach for GBM, comprehensive protocols incorporating lipid‐lowering agents, choleretics, and hepatoprotectors may offer a viable alternative, with a remission probability of 19%.

